# Effects of Gut Microbiota and Metabolites on Heart Failure and Its Risk Factors: A Two-Sample Mendelian Randomization Study

**DOI:** 10.3389/fnut.2022.899746

**Published:** 2022-06-20

**Authors:** Qiang Luo, Yilan Hu, Xin Chen, Yong Luo, Jie Chen, Han Wang

**Affiliations:** ^1^Department of Cardiology, Affiliated Hospital of Southwest Jiaotong University, The Third People's Hospital of Chengdu, Chengdu, China; ^2^Department of Laboratory, Affiliated Hospital of Southwest Jiaotong University, The Third People's Hospital of Chengdu, Chengdu, China

**Keywords:** gut microbiota, mendelian randomization, heart failure, gut metabolites, risk factors for heart failure

## Abstract

**Introduction:**

Previous observational studies have indicated that gut microbiota and metabolites may contribute to heart failure and its risk factors. However, with the limitation of reverse causality and confounder in observational studies, such relationship remains unclear. This study aims to reveal the causal effect of gut microbiota and metabolites on heart failure and its risk factors.

**Methods:**

This study collected summary statistics regarding gut microbiota and metabolites, heart failure, diabetes, hypertension, chronic kidney disease, myocardial infarction, atrial fibrillation, hypertrophic cardiomyopathy, dilated cardiomyopathy, coronary heart disease, valvular heart disease, and myocarditis. Two-sample Mendelian randomization analysis was performed using MR-Egger, inverse variance weighted (IVW), MR-PRESSO, maximum likelihood, and weighted median.

**Results:**

Results from gene prediction showed that among all gut microbiota, *candida, shigella*, and *campylobacter* were not associated with higher incidence of heart failure. However, genetic prediction suggested that for every 1 unit increase in *shigella* concentration, the relative risk increased by 38.1% for myocarditis and 13.3% for hypertrophic cardiomyopathy. Besides, for every 1 unit increased in *candida* concentration, the relative risk of chronic kidney disease increased by 7.1%. As for intestinal metabolites, genetic prediction results suggested that for every 1 unit increase in betaine, the relative risk of heart failure and myocardial infarction increased by 1.4% and 1.7%, separately.

**Conclusions:**

This study suggested new evidence of the relationship between gut microbiota and heart failure and its risk factors, which may shed light on designing microbiome- and microbiome-dependent metabolite interventions on heart failure and its risk factors in clinical trials in the future.

## Introduction

Heart failure is an intractable disease referring to ventricular dysfunction caused by cardiac structure and function changes and is a crucial part of the global prevention and treatment of chronic cardiovascular diseases (1). Studies have shown that the approximate prevalence of heart failure is 1% to 2%, and it may increase continuously with age (2, 3). Patients with heart failure are always accompanied by dyspnea, decreased exercise tolerance, systemic fluid retention and other symptoms, resulting in a serious decline in quality of life and even death (4). Although a growing number of medications have been used for heart failure currently, existing treatments target only a fraction of the putative pathophysiological pathways, thus the overall prognosis of heart failure remains unclear (5, 6). Therefore, early prevention and diagnosis are the key to improving prognosis among patients with heart failure.

Gut microbiota, as the most important active components in the intestinal microecosystem, can not only participate in the digestion of food and absorption of nutrients in order to provide energy for the host, but act as endocrine organs to produce various substances and then involve in various physiological regulation of the host (7). In recent years, more and more studies have proved that the intestinal tract plays an essential role in the pathogenesis of heart failure. An observational study from Pasini et al. reported that compared with the control group, heart failure patients had more *candida, campylobacter, shigella, and yersinia*, and these gut florae were closely associated with the development and progression of the disease (8). Furthermore, there is evidence that intestinal metabolites are strongly associated with heart failure. For example, compared with healthy population, trimethylamine N-oxide (TMAO) levels were higher in chronic heart failure patients and were associated with the New York Heart Association (NYHA) grades, ischemic etiology and adverse outcomes (9). One meta-analysis of 19 prospective studies in 19,256 subjects indicated that elevated plasma TMAO levels were related to an increasing relative risk of major adverse cardiovascular events after adjusting for BMI, diabetes, history of cardiovascular disease, renal dysfunction and other variables (10). At present, most investigators are trying to use gut hypothesis to explain the relationship between gut microbiota and metabolites and heart failure (11). However, research studies supporting the “gut hypothesis” thus far are associative in nature. In addition, the current association was mainly based on observational studies with limited sample size and the presence of confounders.

Mendelian randomization (MR) has emerged as a powerful method for identifying the causation between risk factors and diseases using genetic variants as instrument variables (IVs) (12). Genetic variation can be identified at conception and is generally not susceptible to non-differential measurement error or confounding, while MR meets the condition of causal consequence which is particularly fundamental in inferring causal inference (12). In this study we performed two-sample MR analysis based on public data from genome-wide association study (GWAS), so as to reveal the causal effect of gut microbiota and metabolites on heart failure and its risk factors (i.e., type 2 diabetes (T2DM), hypertension, chronic kidney disease (CKD), coronary heart disease (CAD), myocardial infarction (MI), atrial fibrillation (AF), hypertrophic cardiomyopathy, dilated cardiomyopathy, valvular heart disease, and myocarditis).

## Methods and Materials

### Study Design

**Figure 1:**
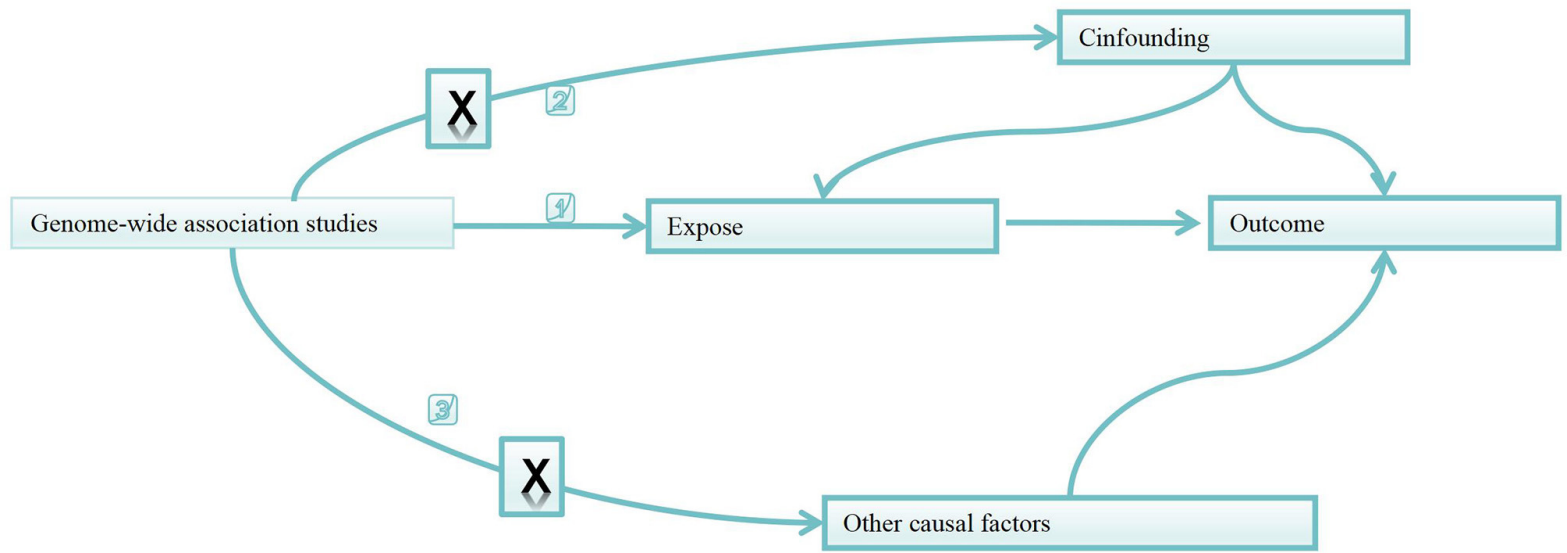
Directed acyclic graphs for the classical Mendelian randomization designs. The arrows denote causal relations between two variables, pointing from the cause to the effect. The causal pathway is blocked if “X” is placed in the arrowed line. MR, Mendelian randomization.

As shown in Figure 1, based on two-sample MR approach, this study aims to investigate the causality between gut microbiota and metabolites and heart failure and its risk factors (13).

### Data Collection for Gut Microbial Metabolites, Gut Microbiota, and Heart Failure and Its Related Risk Factors

Pooled data of gut microbial metabolites (i.e., beta-hydroxy butyric acid, betaine, TMAO, carnitine, choline, glutamate, kynurenine, phenylalanine, propionic acid, serotonin, tryptophan, tyrosine) was from GWAS which includes summary data of human metabolome in 2,076 European participants from Framingham Heart Study (14). Since gene loci identified by GWAS for intestinal metabolites rarely reach genome-wide significance levels, single-nucleotide polymorphisms (SNPs) with suggestive genome-wide significance thresholds (i.e., *P* < 5^*^10^−5^) were selected as instrumental variables (IVs) in this study (Table 1).

**Table 1:** Gut microbiota and metabolites and heart failure and heart failure risk factors summary data sources.

**Trait**	**Year**	**Sample**	**Case**	**Control**
Gut Metabolites	2013	2,076	NA	NA
Gut Microbiota	2021	18,340	NA	NA
Heart failure	2020	977,323	47,309	930,014
Type 2 diabetes	2018	659,316	62,892	596,424
Atrial fibrillation	2018	1,030,836	60,620	970,216
Coronary artery disease	2018	396,525	34,541	261,984
Myocardial infarction	2018	171,875	43,676	128,199
Chronic kidney disease	2016	117,165	12,385	104,780
Hypertension	2018	757,601	NA	NA
Heart valve disease	2020	647,853	27,065	620,788
Myocarditis	2020	605,758	735	605,023
Hypertrophic cardiomyopathy	2020	667,855	890	666,965
Dilated cardiomyopathy	2020	533,543	1,861	531,682
Valvular heart disease	2020	485,153	27,065	458,088

Summary statistics of gut microbiota were from a large-scale multiracial GWAS meta-analysis which consists of 18,340 individuals from 24 cohorts, with 211 taxa (i.e., 131 genera, 35 families, 20 orders, 16 classes, and 9 phyla) (15). *Shigella, campylobacter and candida* were included in this study, and SNPs with suggestive genome-wide significance thresholds (i.e., *P* < 5^*^10^−5^) were selected as IVs (Table 1).

Leading single-nucleotide polymorphisms (SNPs) as genetic IVs were from the current largest available GWAS meta-analysis on heart failure among individuals with European ancestry, performed by the Heart Failure Molecular Epidemiology for Therapeutic Targets Consortium. This GWAS meta-analysis included 47,309 heart failure cases and 930,014 controls from 26 studies with adjustments for age, gender and other principal components (16). Heart failure identification was from at least one of the following databases in all cohort studies: discharge registries, cause of death registries, and physician adjudication/diagnosis. In this study, T2DM, hypertension, CKD, CAD, MI, AF, hypertrophic cardiomyopathy, dilated cardiomyopathy, valvular heart disease, and myocarditis were considered as risk factors of heart failure. Summary-level data was extracted from the Diabetes Genetics Replication and Meta-analysis (DIAGRAM) Consortium for T2DM (*n* = 149,821) (17), the Atrial Fibrillation Consortium (AFGen) for AF (60,620 patients and 970,216 controls) (18), the Coronary Artery Disease Genomewide Replication and Meta-analysis (CARDIoGRAM) plus the Coronary Artery Disease (C4D) Genetics (CARDIoGRAMplusC4D) Consortium for CAD (60,801 patients and 123,504 controls) and MI (43,676 patients and 128,197 controls) (19), the Chronic Kidney Disease Consortium (CKDGen) for CKD (*n* = 133,814) (20), the International Consortium of Blood Pressure-Genome Wide Association Studies (ICBP) for hypertension (*n* = 299,024) (21), the heart valve disease GWAS for valvular heart disease (*n* = 178,726) (22), the myocarditis GWAS for myocarditis (*n* = 177,847) (22), the hypertrophic cardiomyopathy GWAS for hypertrophic cardiomyopathy (*n* = 177,745) (22), and the dilated cardiomyopathy GWAS for dilated cardiomyopathy (*n* = 353,937) (22) (Table 1).

### Selection of Instrumental Variables

First, we used Plink Software to screen SNPs with *P* < 5^*^10^−5^, a genetic distance of 10,000 kb and a linkage disequilibrium parameter (r^2^) of <0.001, from GWAS of gut microbiota and metabolites. Second, we used catalog and PhenoScanner to explore whether the above SNPs were associated with the known confounding (obesity, dyslipidemia), and if yes, the SNP would be excluded. Last, F statistic was calculated for each SNP to test the weak IV bias in this study (23). F statistic of SNP <10 indicated the potential weak IV bias, and then such SNP was eliminated to avoid its influence on results (24).

### Statistical Analysis

This study focused primarily on inverse variance weighted (IVW) approach (25), with IVW fixed-effect model used in the absence of any potential horizontal multiplicity heterogeneity and random-effect model used in the presence of heterogeneity. As for the secondary analysis, MR-Egger, maximum likelihood, weighted median and MR-PRESSO were conducted for sensitivity analysis of IVW results (26–29). Maximum likelihood approach estimates causal effect by the direct maximization of the likelihood given the SNP-exposure and SNP-outcome effects, and assumes a linear relationship between the exposure and outcome. MR-Egger approach is based on the assumption of InSIDE in order to perform weighted linear regression of exposure results, but it is susceptible to IVs. Weighted median approach can significantly improve the detection ability of causal effects as well as reduce type I errors. To account for multiple testing in our study, the Bonferronicorrected significance level of *P* < 3.57×10^−3^ (0.05 divided by 18 risk factors) was used. *P-*value between 3.57×10^−3^ and 0.05 were considered as potential associations.

### Pleiotropy and Heterogeneity Analysis

First, we used MR-PRESSO approach to detect outliers (29) and re-analyzed after removal of outliers. The leave-one-out sensitivity analysis was implemented by removing a single SNP each time to assess whether the variant was driving the association between the exposure and the outcome variable. Second, MR-Egger regression test was also performed to determine the horizontal multiplicity in MR analysis if the intercept term had statistical significance (30). Last, Cochran Q statistic was calculated to detect heterogeneity (31).

The threshold for significance was *P* = 0.05. All statistical analysis were conducted using R, Version 4.1.2 with the two-sample MR and MRPRESSO packages.

## Results

### Participants and Genetic Instrumental Variables for Gut Microbiota and Metabolites

In this study, we found 9SNPs were found to be closely related to obesity, diabetes and dyslipidemia, so they were excluded (see Supplementary 1). Finally, 631 independent genome-wide significant SNPs associated with gut microbe-dependent metabolites and gut bacteria traits were selected for the construction of IVs, including 24 associated with beta-hydroxy butyric acid, 41 associated with betaine, 29 associated with carnitine, 29 associated with choline, 23 associated with glutamate, 47 associated with kynurenine, 39 associated with phenylalanine, 24 associated with propionic acid, 44 associated with serotonin, 56 associated with TMAO, 30 associated with tryptophan, 38 associated with tyrosine, 57 associated with *candida*, 60 associated with *campylobacter*, and 90 associated with *shigella*. Total variance of gut microbe-dependent metabolites and gut bacteria value explained by genetic instruments was 37.14%-87.70%%, respectively. F values were all greater than 18.77, indicating the research was not susceptible to weak IVs. See Supplementary 1 for details of each IV.

### MR Analysis of Gut Microbiota and Heart Failure and Its Risk Factors

**Figure 2:**
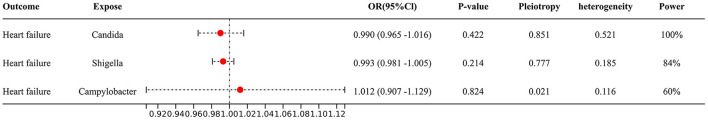
Forest plot to visualize the causal effect of Gut microbiota on the risk of heart failure by inverse variance weighted method.

No significant differences were found between *candida* (OR 0.990, 95% Cl: 0.965–1.016, *P* = 0.422), *shigella* (OR 0.993, 95% Cl: 0.981–1.005, *P* = 0.214) and *campylobacter* (OR 1.012, 95% Cl: 0.907–1.129, *P* = 0.824) and heart failure from IVW results (Figure 2), and sensitivity analysis (see Supplementary 1) yielded similar results.

**Figure 3:**
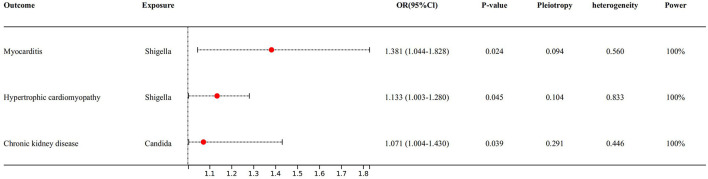
Forest plot to visualize the causal effect of Gut microbiota on the risk of heart failure risk factors by inverse variance weighted method.

We also found that some gut microbiota link strongly with risk factors of heart failure (Figure 3), for example, for every 1 unit increase in *shigella concentration*, relative risk increased by 38.1% (OR 1.381, 95% Cl: 1.044–1.828, *P* = 0.024) for myocarditis, and sensitivity analysis (see Supplementary 1) yielded similar results. Besides, for every 1 unit increase in *shigella concentration*, relative risk increased by 13.3% (OR 1.133, 95% Cl: 1.003–1.280, *P* = 0.045) for hypertrophic cardiomyopathy; for every 1 unit increased in *candida concentration*, relative risk of chronic kidney disease increased by 7.1% (OR 1.071, 95% Cl: 1.004–1.430, *P* = 0.039). However, these two associations were not confirmed in MR-Egger and weighted median method.

There were no directional pleiotropies but potential heterogeneities for the analysis results (see Supplementary 1). We replaced with random-effect model on heterogeneous results for further analysis and found consistent results as before (see Supplementary 1). MR-PRESSO test displayed that there were significant outliers between some gut microbiota and CAD, diastolic blood pressure, systolic blood pressure and T2DM, and after removing those outliers, re-analysis revealed consistency with the previous results (see Supplementary 1). In addition, the leave-one-out analysis reported that the IVs did not have significant impact on the results (see Supplementary 2–5). Funnel plots suggested that points representing causal association effects were symmetrically distributed when single SNP was used as IV, indicating that causal associations were less likely to be affected by potential biases (see Supplementary 2–5).

### MR Analysis of Gut Microbial Metabolites and Heart Failure and Its Risk Factors

**Figure 4:**
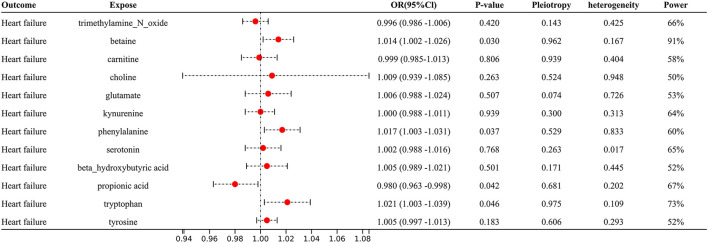
Forest plot to visualize the causal effect of Gut metabolites on the risk of heart failure by inverse variance weighted method.

**Figure 5:**
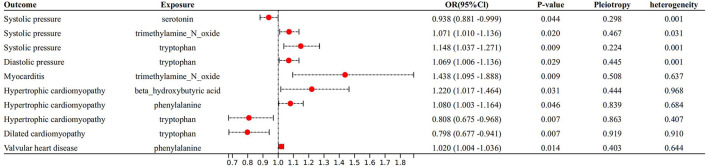
Forest plot to visualize the causal effect of Gut metabolites on the risk of heart failure risk factors by inverse variance weighted method.

**Figure 6:**
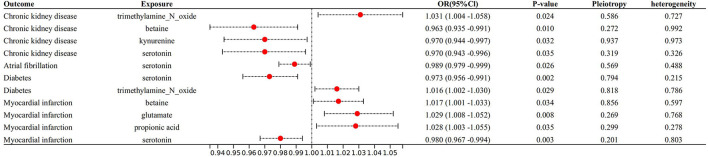
Forest plot to visualize the causal effect of Gut metabolites on the risk of heart failure risk factors by invers variance weighted method.

With IVW approach, we observed some gut microbial metabolites are not only closely related to heart failure (Figures 4–6), but to its risk factors. (a) For every 1 unit increase in betaine, relative risk of heart failure and MI increased by 1.4% (OR 1.014, 95% Cl: 1.002–1.026, *P* = 0.030) and 1.7% (OR 1.017, 95% Cl: 1.001–1.033, *P* = 0.034), separately, yet relative risk of CKD decreased by 3.7% (OR 0.963, 95% Cl: 0.934–0.991, *P* = 0.010). (b)For every 1 unit increase in phenylalanine, relative risk of heart failure, hypertrophic cardiomyopathy and valvular heart disease increased by 1.7% (OR 1.017, 95% Cl: 1.003–1.031, *P* = 0.037), 8.0% (OR 1.080, 95% Cl: 1.003–1.164, *P* = 0.046) and 2.0% (OR 1.020, 95% Cl: 1.004–1.1036, *P* = 0.014), respectively. (c) For every 1 unit increase in tryptophan, relative risk of heart failure, elevated systolic pressure and diastolic pressure increased by 2.1% (OR 1.021, 95% Cl: 1.003–1.039, *P* = 0.046), 14.8% (OR 1.148, 95% Cl: 1.037–1.271, *P* = 0.009) and 6.9% (OR 1.069, 95% Cl: 1.006–1.136, *P* = 0.029), respectively, while relative risk of hypertrophic cardiomyopathy and dilated cardiomyopathy decreased by 19.2% (OR 0.808, 95% Cl: 0.675–0.968, *P* = 0.007) and 20.2% (OR 0.798, 95% Cl: 0.677–0.941, *P* = 0.007). (d) For every 1 unit increase in propionic acid, relative risk of heart failure decreased by 2.0% (OR 0.980, 95% Cl: 0.963–0.998, *P* = 0.042) while relative risk of MI increased by 2.8% (OR 1.028, 95% Cl: 1.003–1.055, *P* = 0.035). (e) For every 1 unit increase in TMAO, relative risk of elevated systolic blood pressure, chronic nephritis and T2DM increased by 7.1% (OR 1.071, 95% Cl: 1.010–1.136, *P* = 0.020), 3.1% (OR 1.031, 95% Cl:1.004–1.058, *P* = 0.024) and 1.6% (OR 1.016 95% Cl: 1.002–1.033, *P* = 0.029), respectively. (f) For every 1 unit increase in beta–hydroxy butyric acid, relative risk of hypertrophic cardiomyopathy increased by 22% (OR 1.220, 95% Cl: 1.017–1.464, *P* = 0.031). (g) For every 1 unit increase in glutamate, relative risk of MI increased by 2.9% (OR 1.029, 95% Cl: 1.008–1.052, *P* = 0.008). (h) For every 1 unit increase in serotonin, relative risk of CKD, AF, T2DM, MI and elevated systolic blood pressure decreased by 3.0% (OR 0.970, 95% Cl: 0.943–0.996, *P* = 0.035),1.1% (OR 0.989, 95% Cl: 0.979–0.998, *P* = 0.026), 2.7% (OR 0.973, 95% Cl: 0.956–0.991, *P* = 0.002), 2.0% (OR 0.980, 95% Cl: 0.967–0.994, *P* = 0.003) and 6.2% (OR 0.938, 95%Cl: 0.881–0.999, *P* = 0.044). (i) For every 1 unit increase in kynurenine, relative risk of CKD decreased by 3.0% (OR 0.970, 95% Cl: 0.944–0.997, *P* = 0.032). Some above associations were proved by MR–Egger, MR–PRESSO, maximum likelihood, weighted median (see Supplementary 1).

Pleiotropy and heterogeneity analysis reported the presence of potential heterogeneities but absence of directional pleiotropies for the results. In view of some heterogeneous results, we changed to random–effect model and found consistent results except for the relationship between phenylalanine and systolic blood pressure, and between beta–hydroxy butyric acid and diastolic blood pressure (see Supplementary 1). In addition, MR–PRESSO test revealed that there are no obvious outliers for the instrumental variables in this study, and the leave–one–out sensitivity alco confirmed the above conclusion (see Supplementary 2–5). Funnel plots suggested that SNPs are symmetrically distributed, indicating that causal associations were less likely to be affected by potential biases (see Supplementary 2–5).

## Discussion

This is the first MR analysis to examine the genetically predictive ability of gut microbiota and metabolites on heart failure and its risk factors. Our findings suggested a partial gut microbiota and metabolites could promote or prevent heart failure or its risk factors.

To draw causality conclusions from MR studies, it is crucial to determine the bias introduced by potential violations of MR assumption and to assess the result consistency with observational literature. Therefore, we compared results from previous observational studies with the purpose of reliability assessment of our MR results. Betaine, a metabolic imperative, was found to be strongly associated with MI in this study which was generally consistent with the prior observational study with 1,876 participants who confirmed a dose–dependent relationship (32). Animal studies have reported that the mechanism of betaine on MI may refer to its up–regulation promotion of various macrophage clearance receptors related to atherosclerosis, but the exact mechanism required further exploration (32). In addition, we observed a suggestive association of genetically increased betaine with higher risk of heart failure and it has been proved by studies of Michael et al. where plasma betaine concentrations were independently associated with incidence of heart failure (33, 34). Another PREDIMED study with 326 patients documented the close association between betaine and heart failure even after adjusting for classical risk factors (OR 1.65, 95% Cl: 1.00–2.71) (35). At present, mechanisms of how betaine acts on the occurrence and development of heart failure are still rudimentary understood. Tang et al. has reported the increased betaine level can lead to further deterioration of left ventricular diastolic and systolic function among heart failure patients, while no clear link was found between betaine and inflammatory and endothelial biomarkers (36). It was speculated that betaine may mediate the occurrence of heart failure through an independent metabolic pathway instead of inflammation and endothelial injury pathways (36). Since another important function of betaine is to maintain the relative stability of cellular osmotic pressure, it has been pointed out that when betaine concentration in the human body is too high, it will lead to errors in the folding of cell membrane proteins, which in turn affects the distribution of intracellular and extracellular fluids (37). Whether there is a link between such mechanism and heart failure is not clear and a large number of studies are still needed to prove it. In conclusion, we found that betaine can not only directly affect the occurrence of heart failure, but indirectly promote it through myocardial infarction. The phenomenon implied that in the treatment of MI or suspected heart failure, we should particularly pay attention to patient's diet in terms of avoiding high betaine diet, so as to reduce the incidence rate, and then reduce the mortality and disability rate and improve the prognosis. This study also found metabolites of betaine (i.e., TMAO) is strongly associated with an increase in systolic blood pressure, which is similar as a previous meta–analysis (38). However, the mechanism of TMAO leading to elevated blood pression remains poorly understood, Brunt et al. explained such relationship through the inhibition ability of TMAO on the activity of eNOS and induced oxidative stress, which contributed to impaired endothelial cell function (39). Besides betaine, the most mentioned gut metabolites are tryptophan and phenylalanine, which, in our study, both elevated the risk of heart failure. This is similar to previous findings. For example, using metabolomics, Tang and colleagues found that tryptophan was strongly associated with the development of heart failure (40), which was also confirmed by other studies, and it was thought that the link between the two was mainly dependent on inflammation and oxidative stress (41). In a longitudinal study, Cheng et al. used metabolomics and demonstrated that phenylalanine levels were significantly elevated in patients with progressive heart failure (42). Furthermore, phenylalanine was also found to be an independent predictor of heart failure in an analysis of data from the PROSPER and FINRISK cohorts, even after adjustment for confounders (43). Authors speculated that phenylalanine may mediate inflammation to elevate the risk of heart failure. However, phenylalanine may also directly predict death of heart failure independent of traditional risk factors and inflammation (44).

This study also showed that some gut microbial metabolites, such as phenylalanine, tryptophan, propionic acid, beta–hydroxy butyric acid, glutamate, serotonin, *shigella*, and *candida* are closely related to the occurrence of heart failure and its risk factors, but the exact mechanism remained unclear, which needs further studies to verify.

Regrettably, *candida, shigella, and campylobacter* are not associated with heart failure, although they may affect chronic kidney disease and myocarditis in our study. This has some conflict with previous findings (8). However, that study was only an observational study, and a causal relationship between the two could not be concluded. Moreover, it also suggested that single bacteria may not affect the onset of HF, and the overall role of the gut microbiota may have more important roles on heart failure risk. Additionally, the role of gut metabolites associated with heart failure also needs to be reconsidered. Therefore, the incorporation of more potential bacteria to explore the risk of heart failure should be needed by MR.

Our study has a few limitations. Firstly, although the GAWS associated with heart failure that we included in this study had the largest known sample size, the GWAS had a drawback in that the study also included a small Asian population, which may have affected the accuracy of our results. Secondly, we could not completely rule out the possible interaction between diet–gene or gene–environment, which might have an impact on our results. Thirdly, most of the studies we included had case and control groups. However, not all the participants included in the study have been subject to strict quality control. And, unlike other cardiovascular diseases, heart failure was not a single homogenous cardiovascular disease. Moreover, the heart failure risk factors may change with age, and the composition of gut microbiota and metabolites may also change with age. Therefore, we cannot ignore the critical role of age, which may affect the stability of our results. Fourthly, after Bonferroni corrected, we did not find a clear causal relationship between gut flora and metabolites and heart failure and heart failure risk factors, suggesting that more studies are still needed to confirm the relationship between them. Fifthly, just as other MR studies, we could not address unobserved pleiotropies. Last, it should be acknowledged that IVW effect estimates are liable to be biased when some instrumental SNPs exhibit horizontal pleiotropy (e.g., when we have genetically determined factors which are associated with heart failure). Despite these limitations, our study has several advantages. First, a large amount of data in this study allowed us to perform comprehensive analysis for incident heart failure and well–powered GWAS to obtain genetic instruments for MR analyses. Besides, the consistent causal estimation across five methods (i.e., MR–Egger, IVW, MR–PRESSO, maximum likelihood, weighted median) suggests robustness of our findings.

## Conclusion

In conclusion, we lent potential evidence for the first time to the causal effect of gut microbiota and metabolites on heart failure and its risk factors. However, more original studies are still needed to explore the exact relationship between gut flora and metabolites and heart failure and heart failure risk factors. And further studies should conduct a more thorough review of the exact mechanism of such association.

## Data Availability Statement

The original contributions presented in the study are included in the article/Supplementary Material, further inquiries can be directed to the corresponding author.

## Author Contributions

HW and QL: study design. QL, YH, and XC: data collection and data analysis. HW, QL, YH, and JC: data interpretation. HW, QL, and YL: drafting manuscript. All authors take responsibility for the integrity of the data analysis. All authors participated and approved the final version of manuscript.

## Funding

This work was supported by National Natural Science Foundation of China (Grant 81300243), Sichuan Administration of Traditional Chinese Medicine (2020JC0010), Chengdu Health Commission Medical Research Project (2021206), Project of Sichuan Science and Technology Department (19YYJC0580), Chengdu High–level Key Clinical Specialty Construction Project, and Chengdu Science and Technology Bureau Technology Innovation Project (2019–YF05–00523–SN).

## Conflict of Interest

The authors declare that the research was conducted in the absence of any commercial or financial relationships that could be construed as a potential conflict of interest.

## Publisher's Note

All claims expressed in this article are solely those of the authors and do not necessarily represent those of their affiliated organizations, or those of the publisher, the editors and the reviewers. Any product that may be evaluated in this article, or claim that may be made by its manufacturer, is not guaranteed or endorsed by the publisher.
