# Supplementary material for: Effects of Gut Microbiota and Metabolites on Heart Failure and Its Risk Factors: A Two-Sample Mendelian Randomization Study
Source: Front Nutr. 2022 Jun 20;9:899746. doi: 10.3389/fnut.2022.899746 (PMC9253861; doi:10.3389/fnut.2022.899746)

Supplementary Information

Effects of Gut Microbiota and Metabolites on Heart Failure and Its Risk Factors: A Two-sample Mendelian Randomization Study

Qiang Luo[a], Yilan Hu[a], Xin Chen[a], Yong Luo[a], Jie Chen[a], Han Wang[a, *]

Figure 1-24: Effects of gut metabolites and heart failure.

Figure 25-48: Effects of gut metabolites and atrial fibrillation

Figure 49-72: Effects of gut metabolites and hypertrophic cardiomyopathy

Figure 73-96: Effects of gut metabolites and coronary heart disease

Figure 97-120: Effects of gut metabolites and dilated cardiomyopathy

Figure 121-144: Effects of gut metabolites and chronic kidney disease

Figure 145-168: Effects of gut metabolites and systolic blood pressure

Figure 169-192: Effects of gut metabolites and diastolic blood pressure

Figure 193-216: Effects of gut metabolites and diabetes

Figure 217-240: Effects of gut metabolites and myocardial infarction

Figure 241-254: Effects of gut metabolites and myocarditis

Figure 255-278: Effects of gut metabolites and valvular heart disease

Figure 279-284: Effects of gut microbiota and atrial fibrillation

Figure 285-290: Effects of gut microbiota and hypertrophic cardiomyopathy

Figure 291-296: Effects of gut microbiota and coronary heart disease

Figure 297-302: Effects of gut microbiota and dilated cardiomyopathy

Figure 303-308: Effects of gut microbiota and chronic kidney disease

Figure 309-314: Effects of gut microbiota and systolic blood pressure

Figure 315-320: Effects of gut microbiota and diastolic blood pressure

Figure 321-326: Effects of gut microbiota and diabetes

Figure 327-322: Effects of gut microbiota and valvular heart disease

Figure 333-338: Effects of gut microbiota and myocardial infarction

Figure 339-342: Effects of gut microbiota and myocarditis

Figure 343-348: Effects of gut microbiota and heart failure

Figure 1: Leave-one-out plot to visualize causal effect of beta_hydroxybutyric acid on the risk of heart failure when leaving one SNP out.

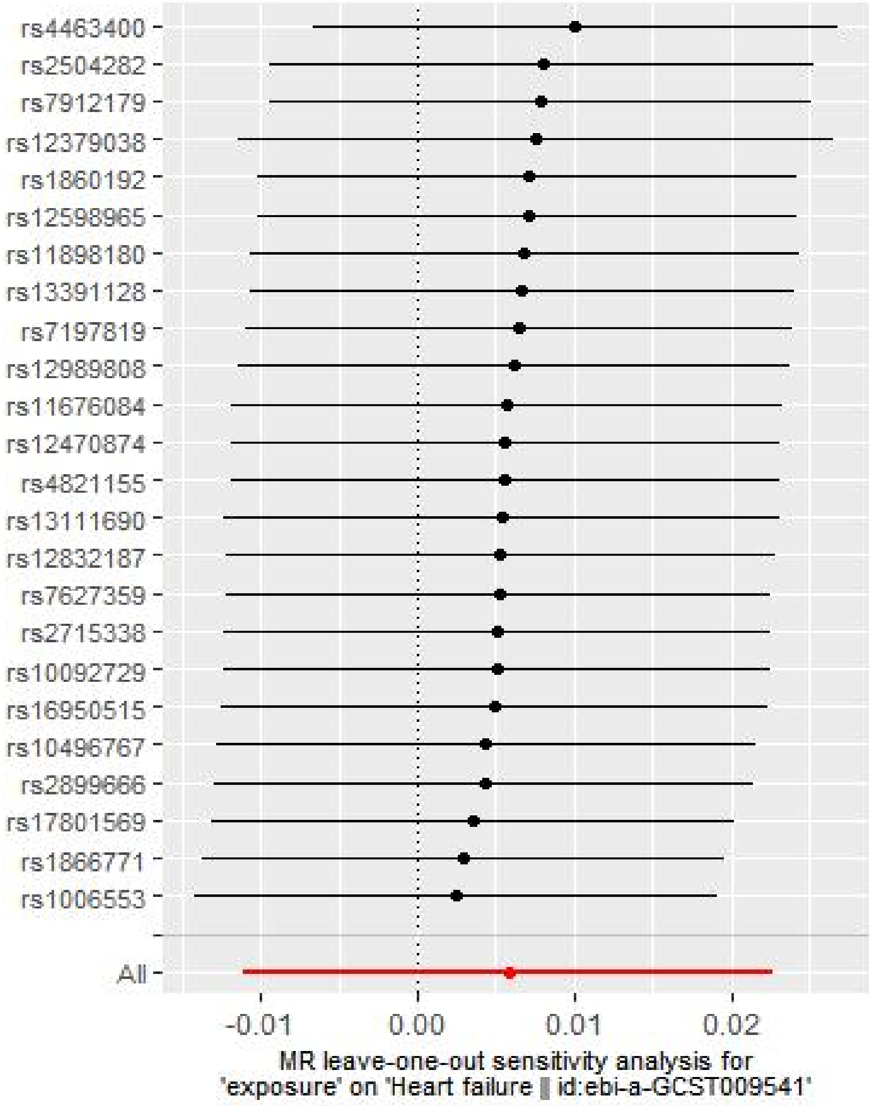

Figure 2: Funnel plots to visualize overall heterogeneity of Mendelian randomization (MR)

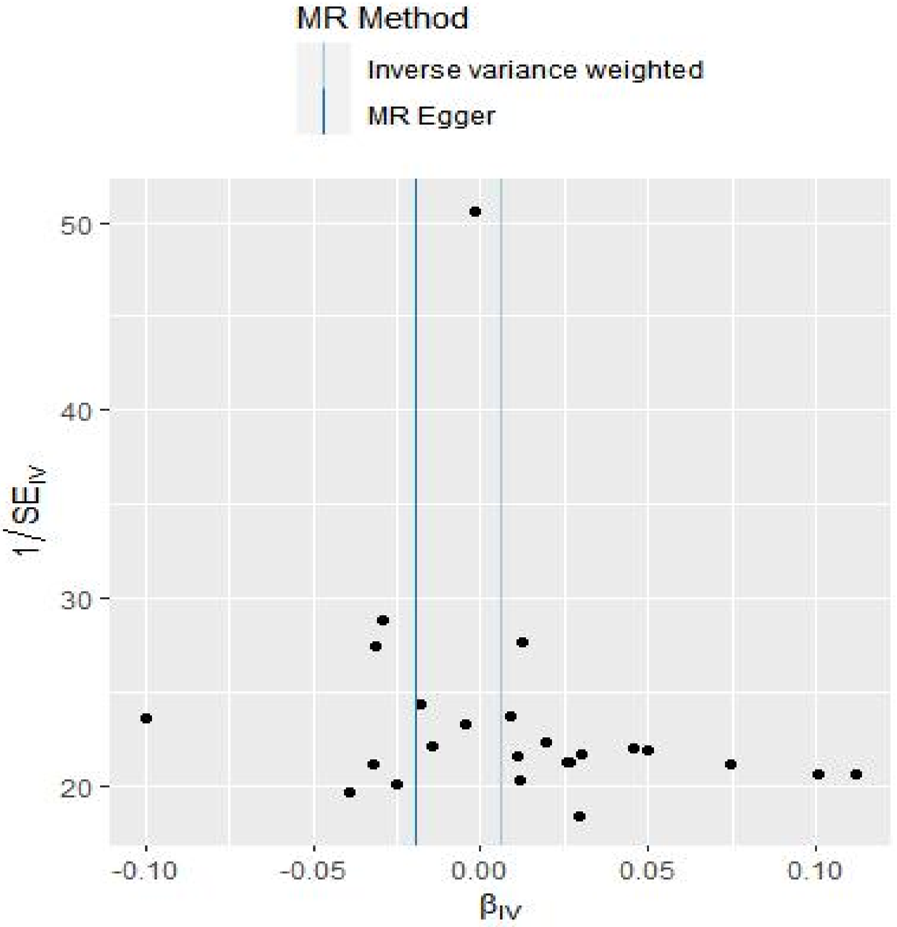

Figure 3: Leave-one-out plot to visualize causal effect of betaine on the risk of heart failure when leaving one SNP out.

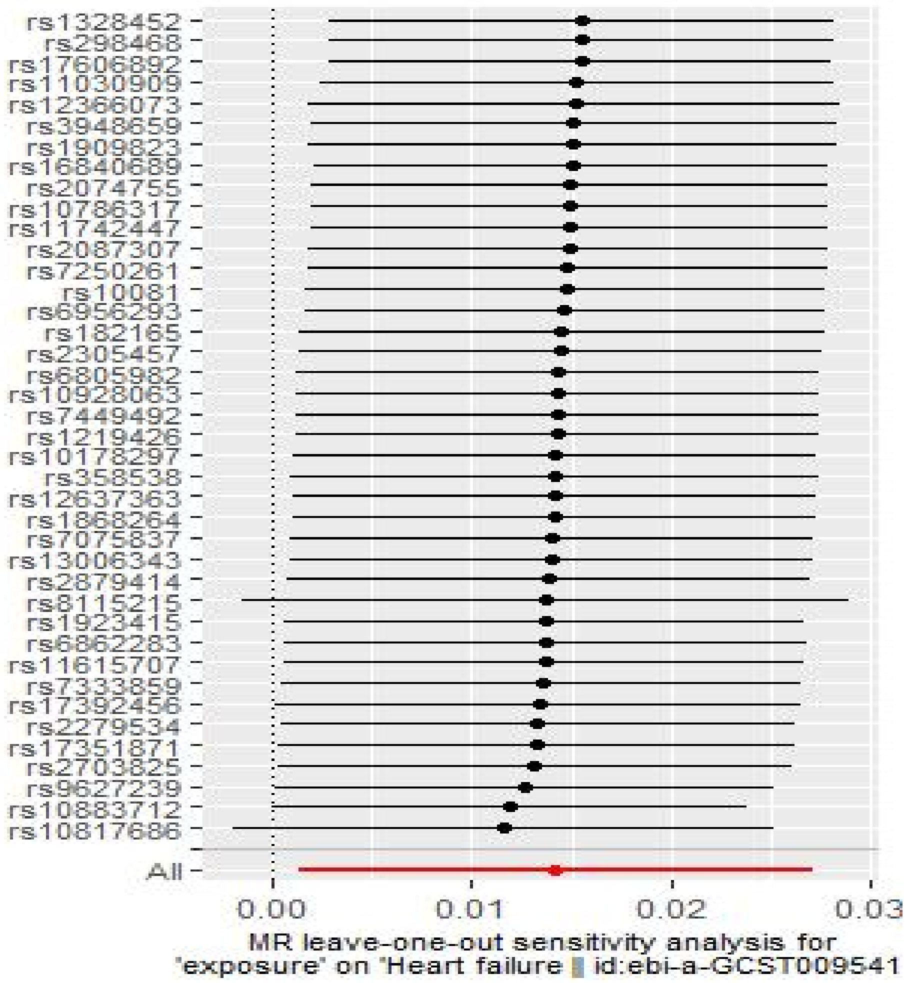

Figure 4: Funnel plots to visualize overall heterogeneity of Mendelian randomization (MR)

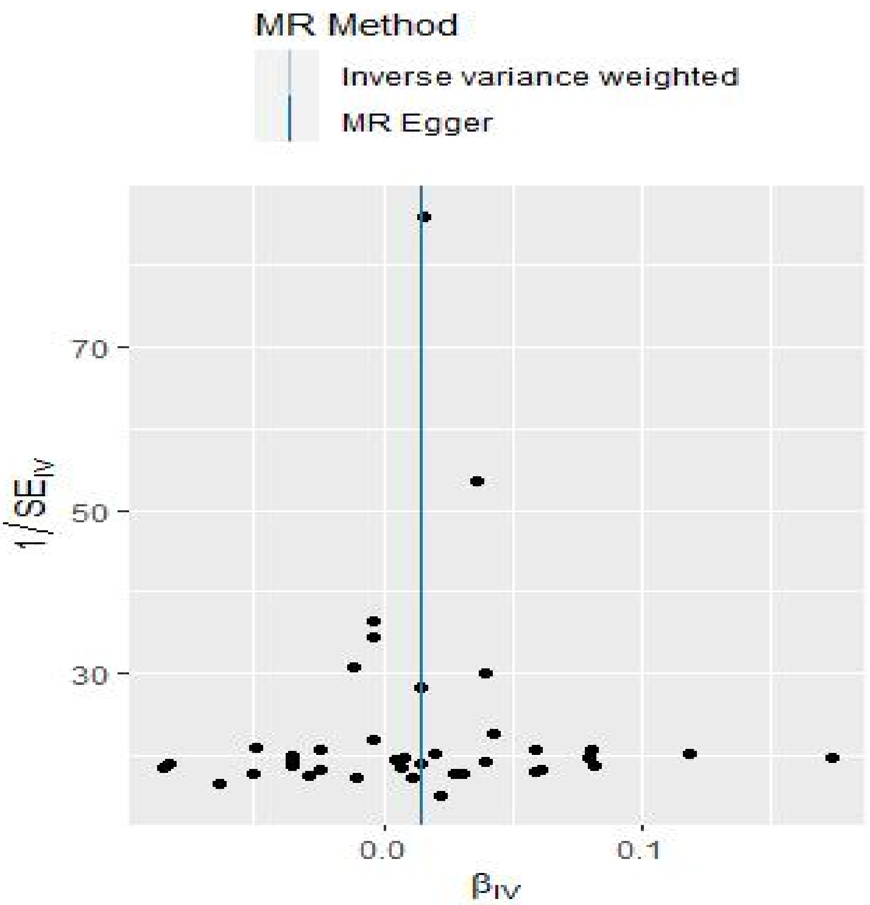

Figure 5: Leave-one-out plot to visualize causal effect of carnitine on the risk of heart failure when leaving one SNP out.

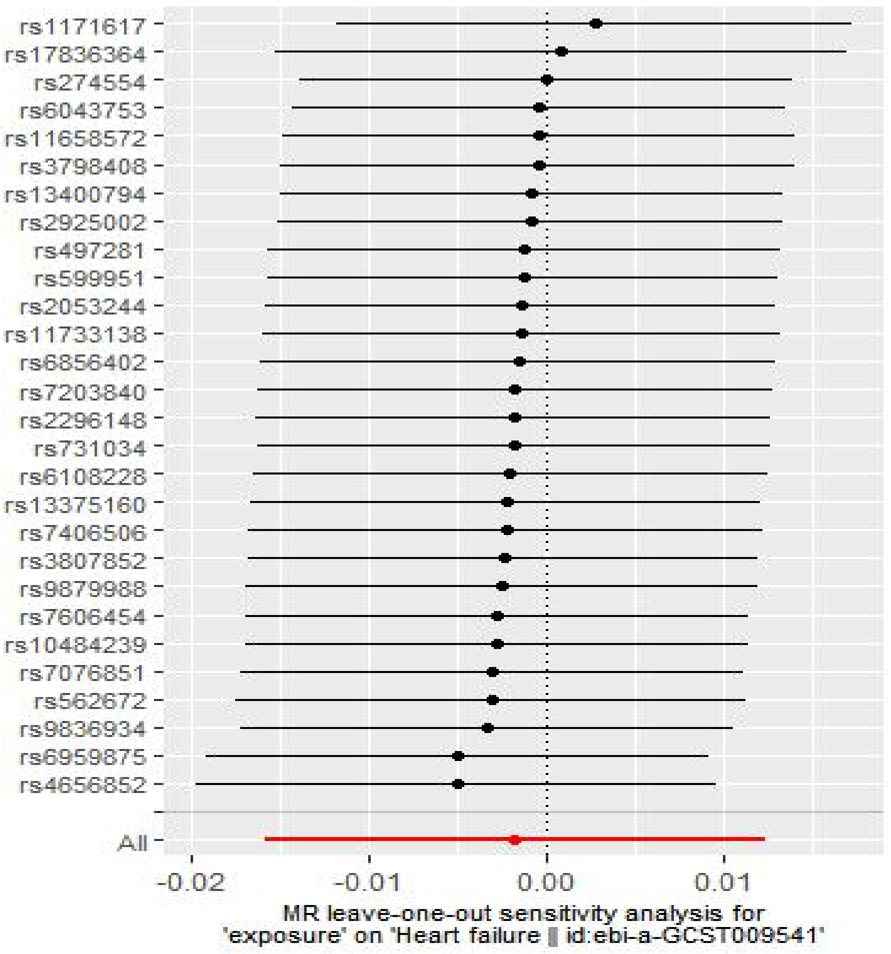

Figure 6: Funnel plots to visualize overall heterogeneity of Mendelian randomization (MR)

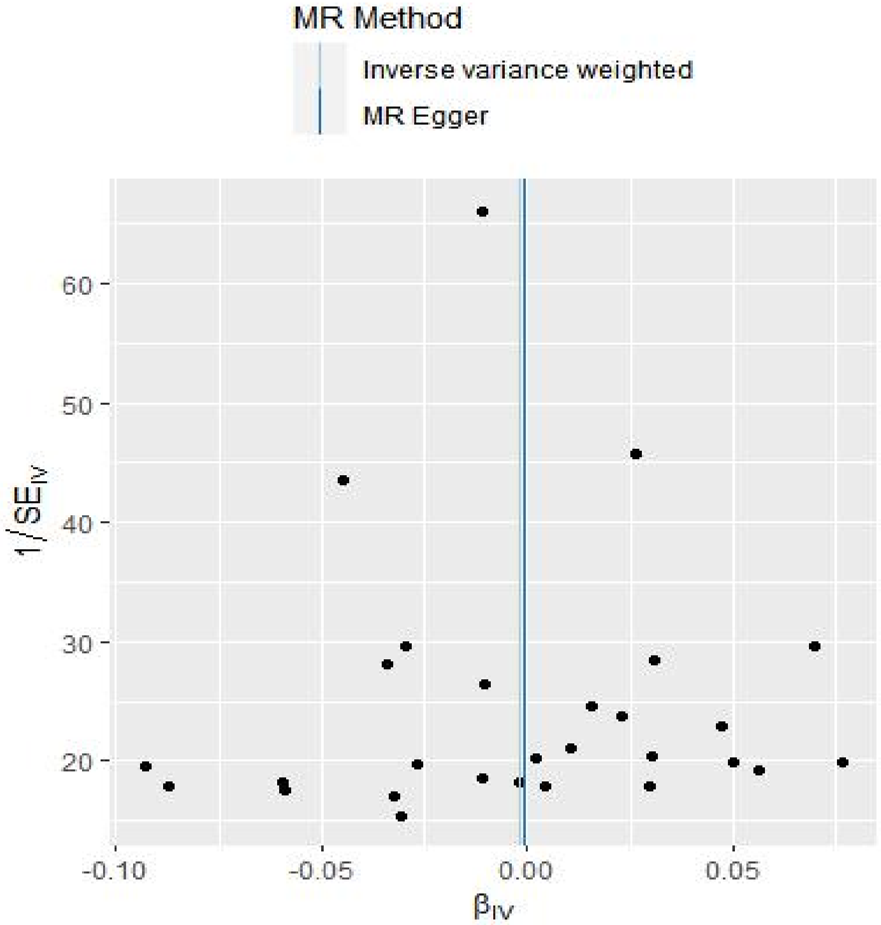

Figure 7: Leave-one-out plot to visualize causal effect of choline on the risk of heart failure when leaving one SNP out.

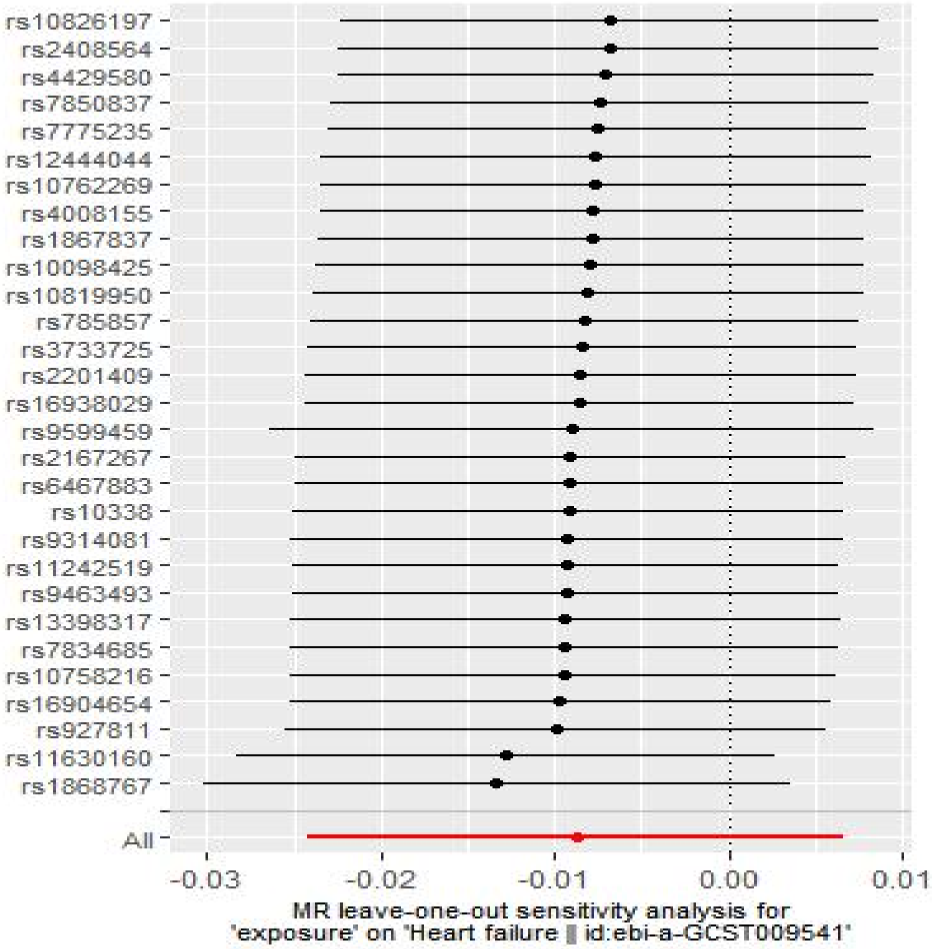

Figure 8: Funnel plots to visualize overall heterogeneity of Mendelian randomization (MR)

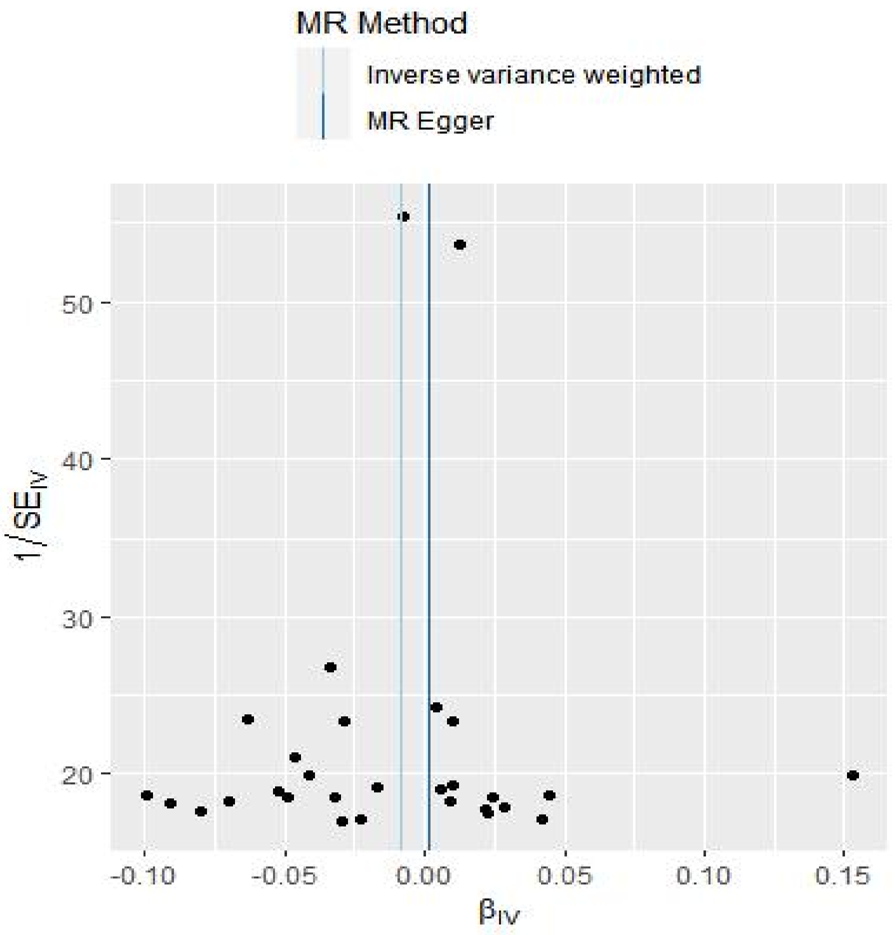

Figure 9: Leave-one-out plot to visualize causal effect of glutamate on the risk of heart failure when leaving one SNP out.

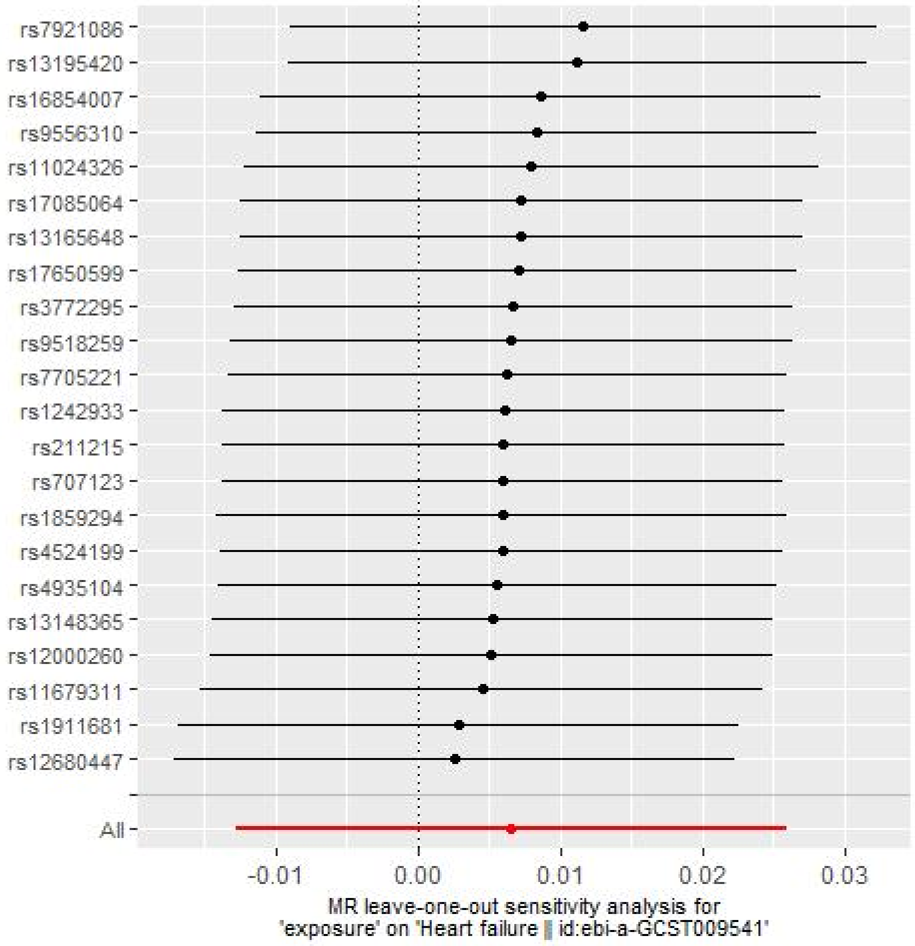

Figure 10: Funnel plots to visualize overall heterogeneity of Mendelian randomization (MR)

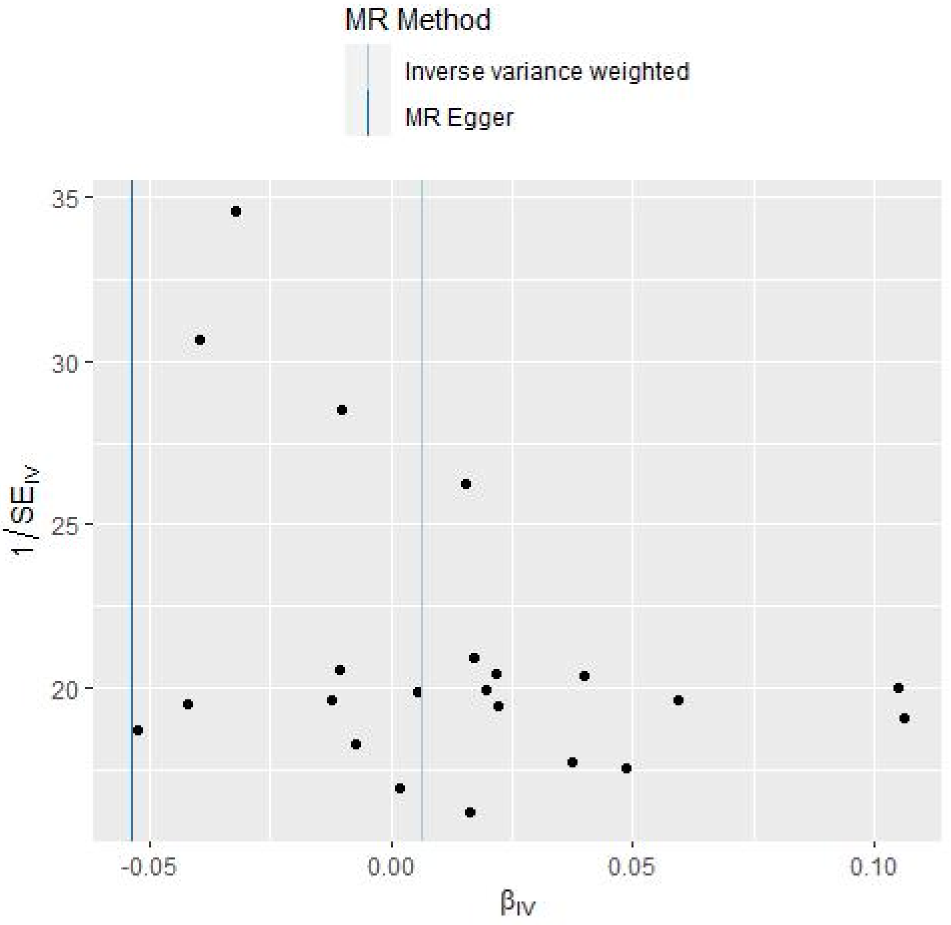

Figure 11: Leave-one-out plot to visualize causal effect of kynuremine on the risk of heart failure when leaving one SNP out.

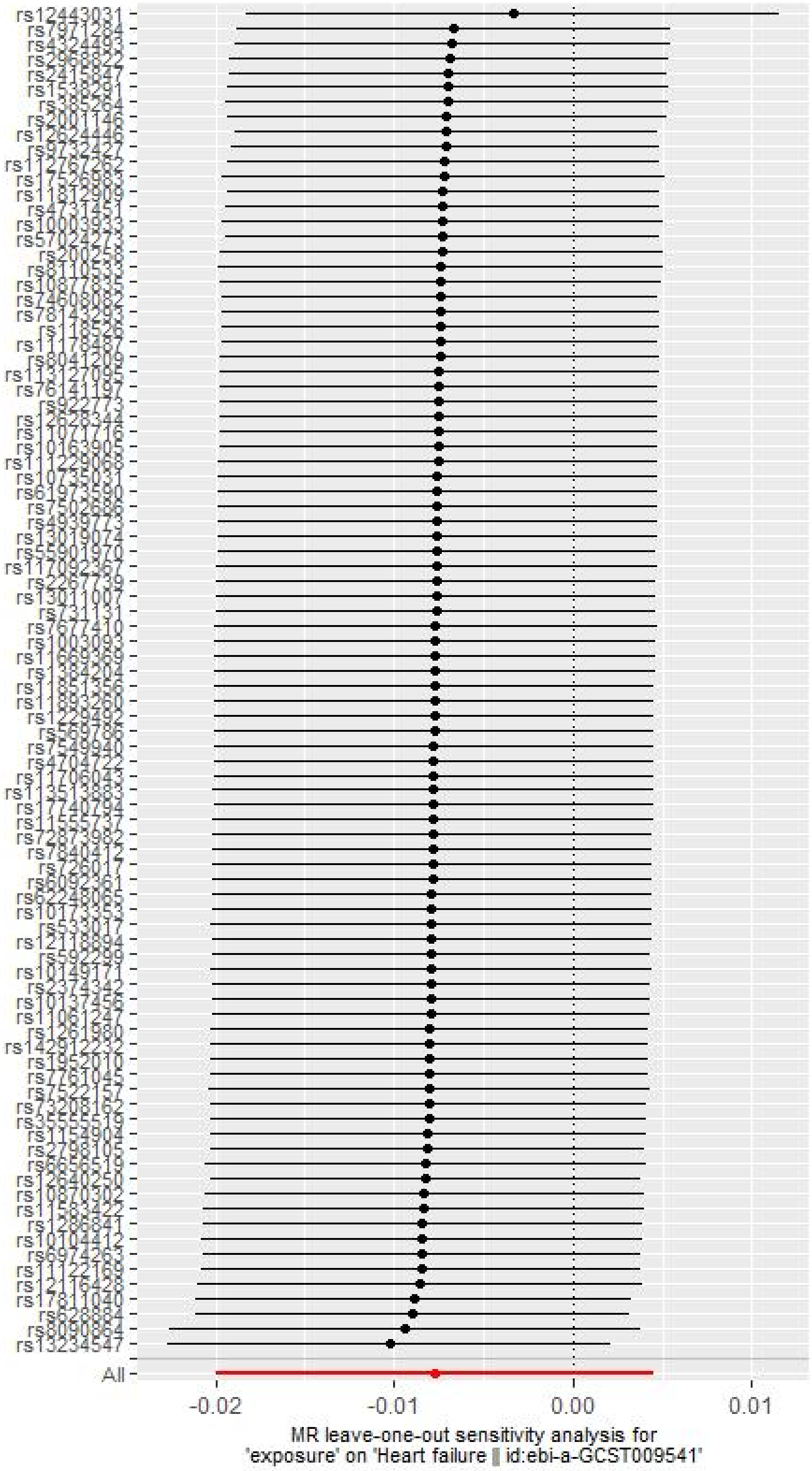

Figure 12: Funnel plots to visualize overall heterogeneity of Mendelian randomization (MR)

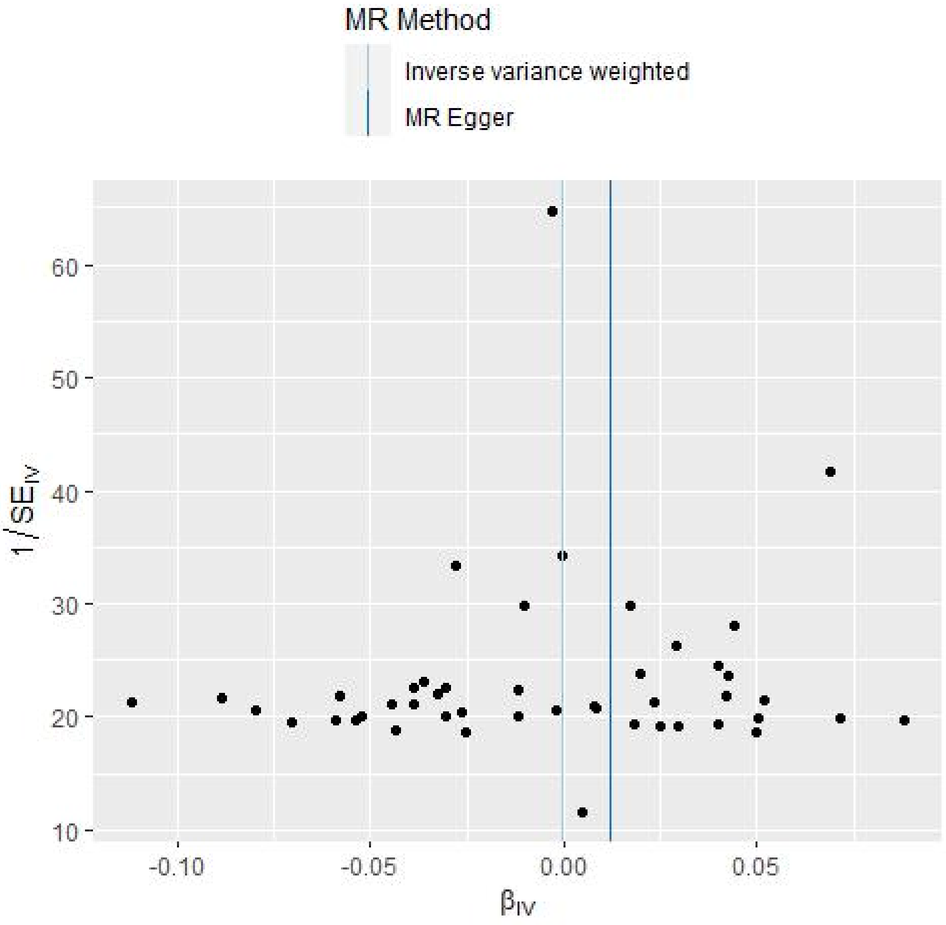

Figure 13: Leave-one-out plot to visualize causal effect of phenylalanine on the risk of heart failure when leaving one SNP out.

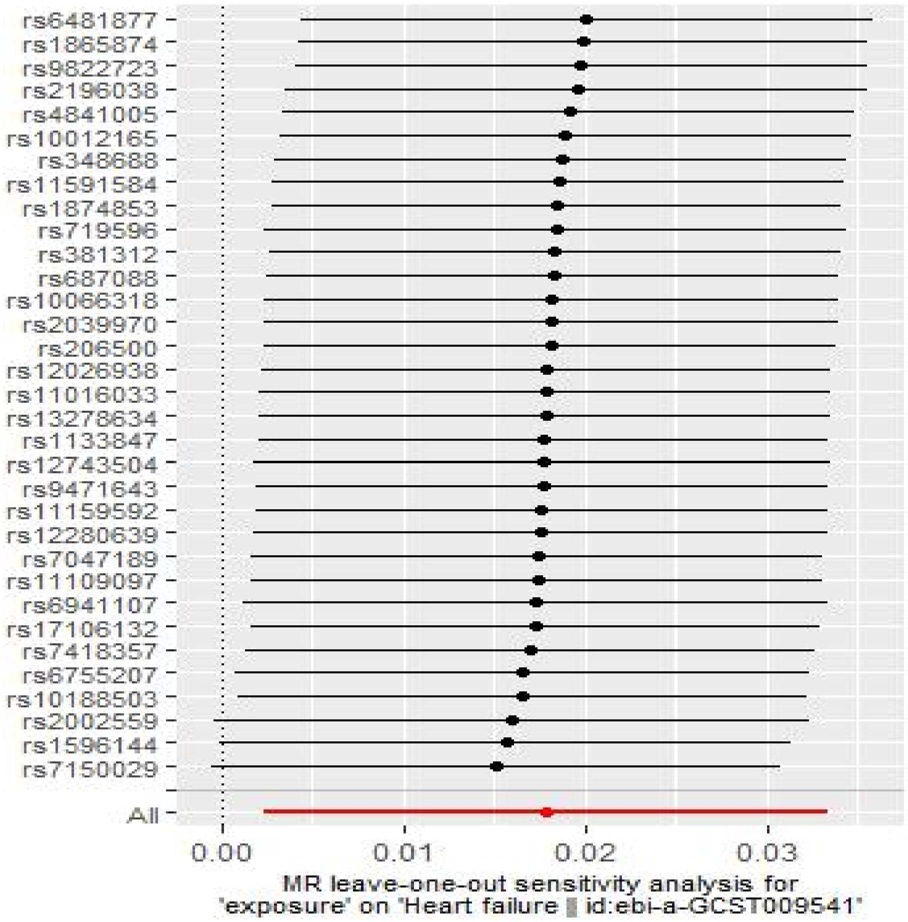

Figure 14: Funnel plots to visualize overall heterogeneity of Mendelian randomization (MR)

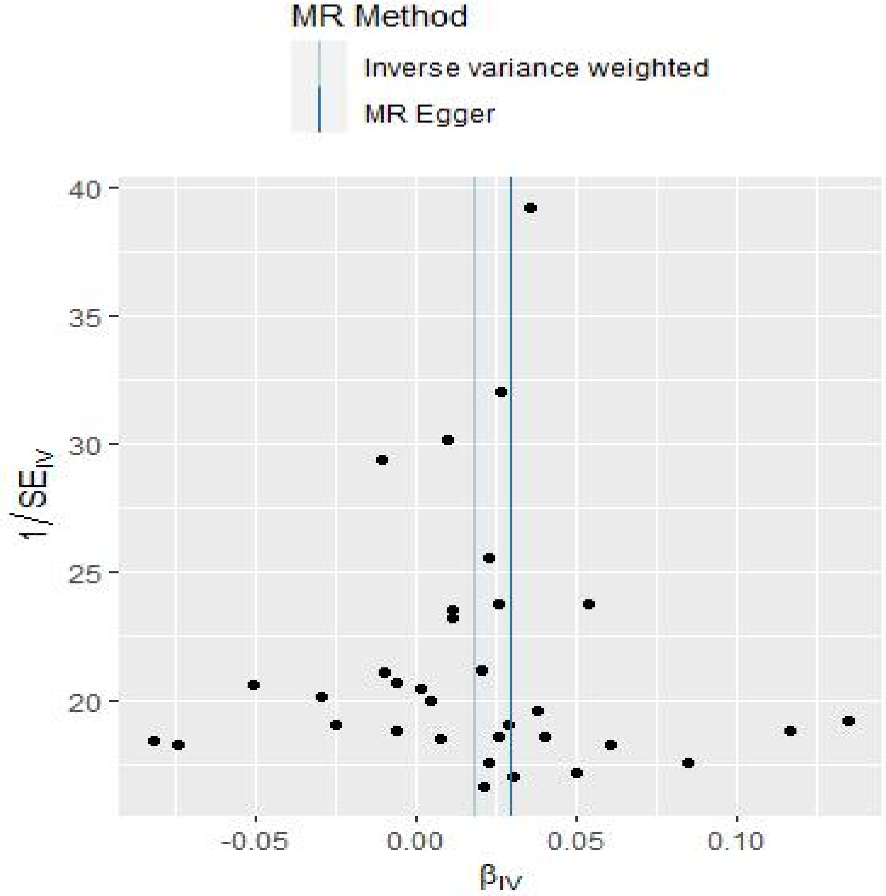

Figure 15: Leave-one-out plot to visualize causal effect of serotonin on the risk of heart failure when leaving one SNP out.

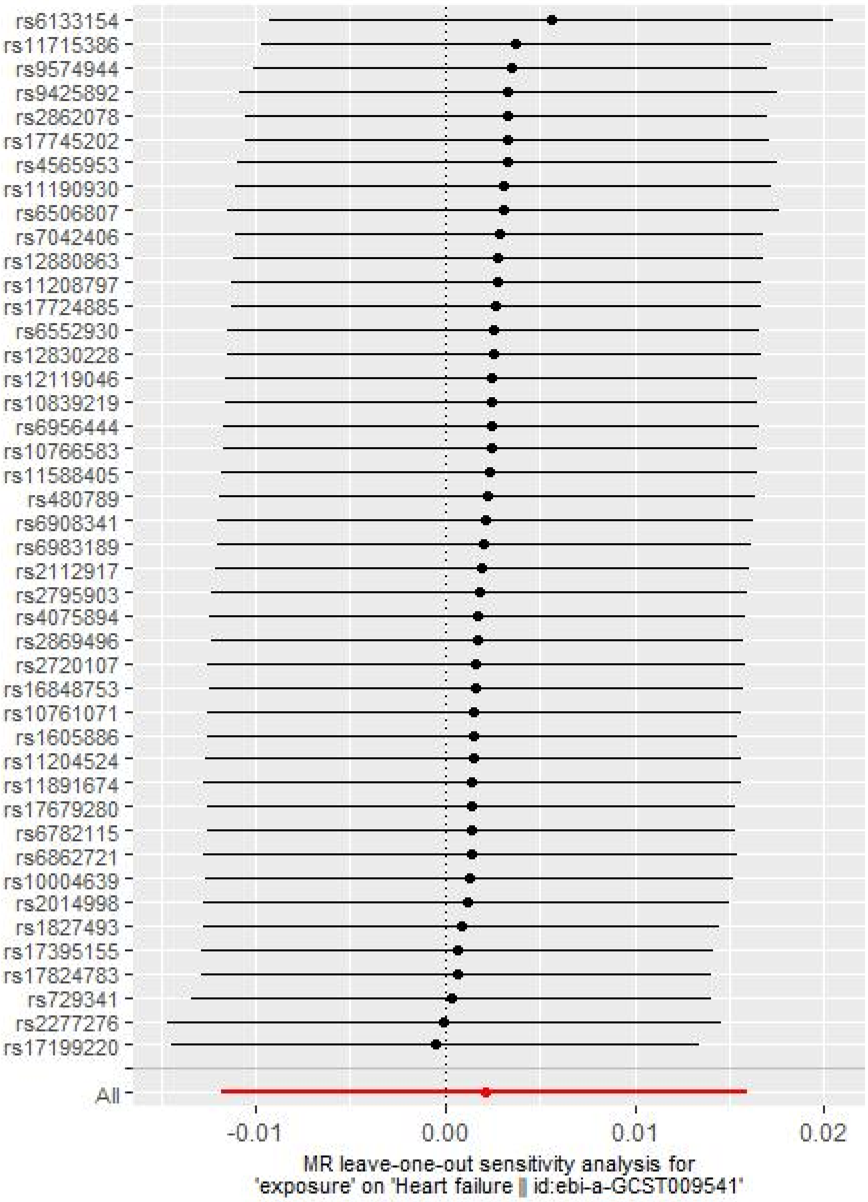

Figure 16: Funnel plots to visualize overall heterogeneity of Mendelian randomization (MR)

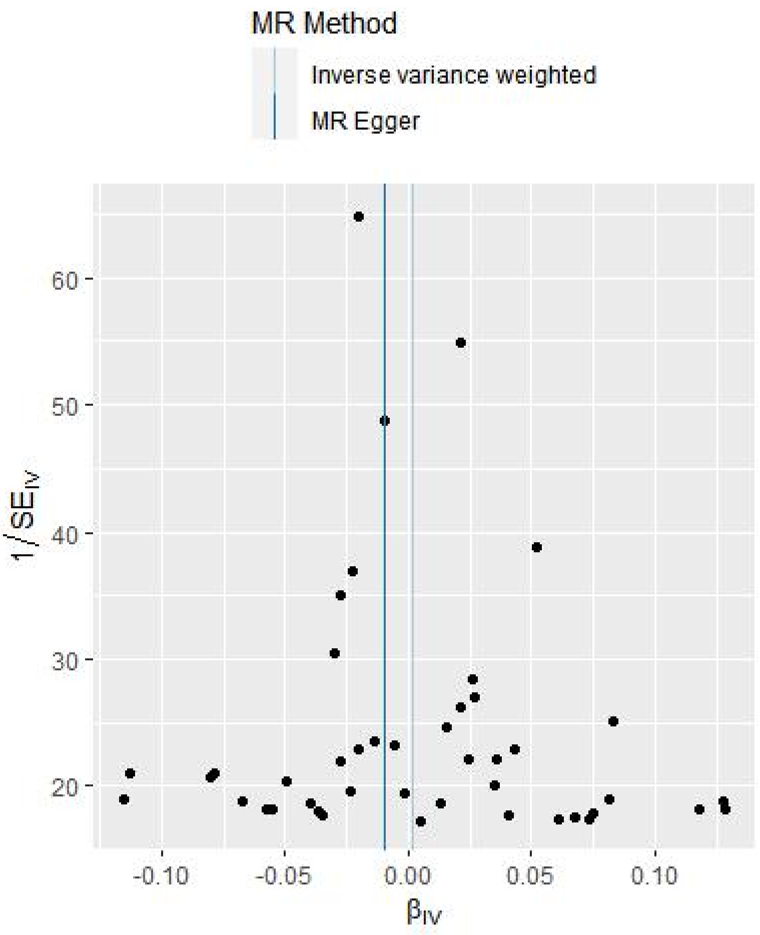

Figure 17: Leave-one-out plot to visualize causal effect of trimethylamine_N_oxide on the risk of heart failure when leaving one SNP out.

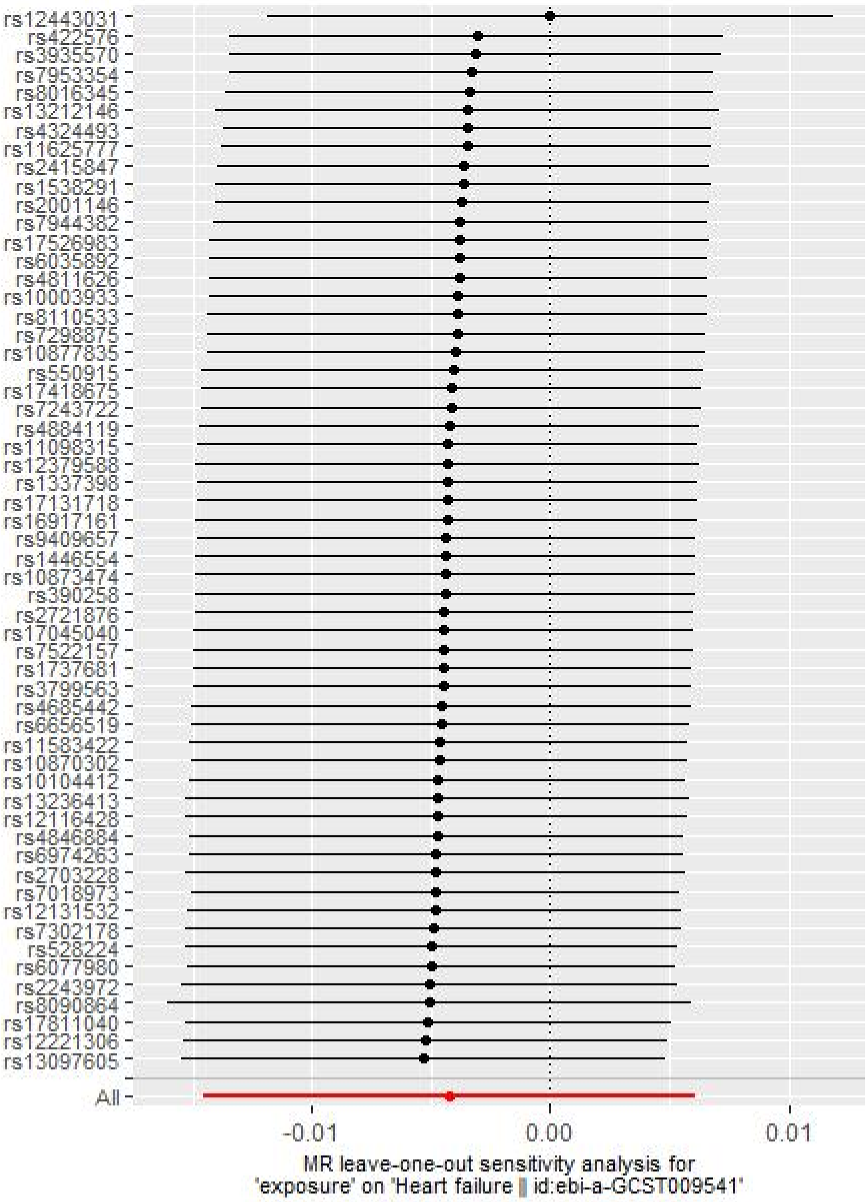

Figure 18: Funnel plots to visualize overall heterogeneity of Mendelian randomization (MR)

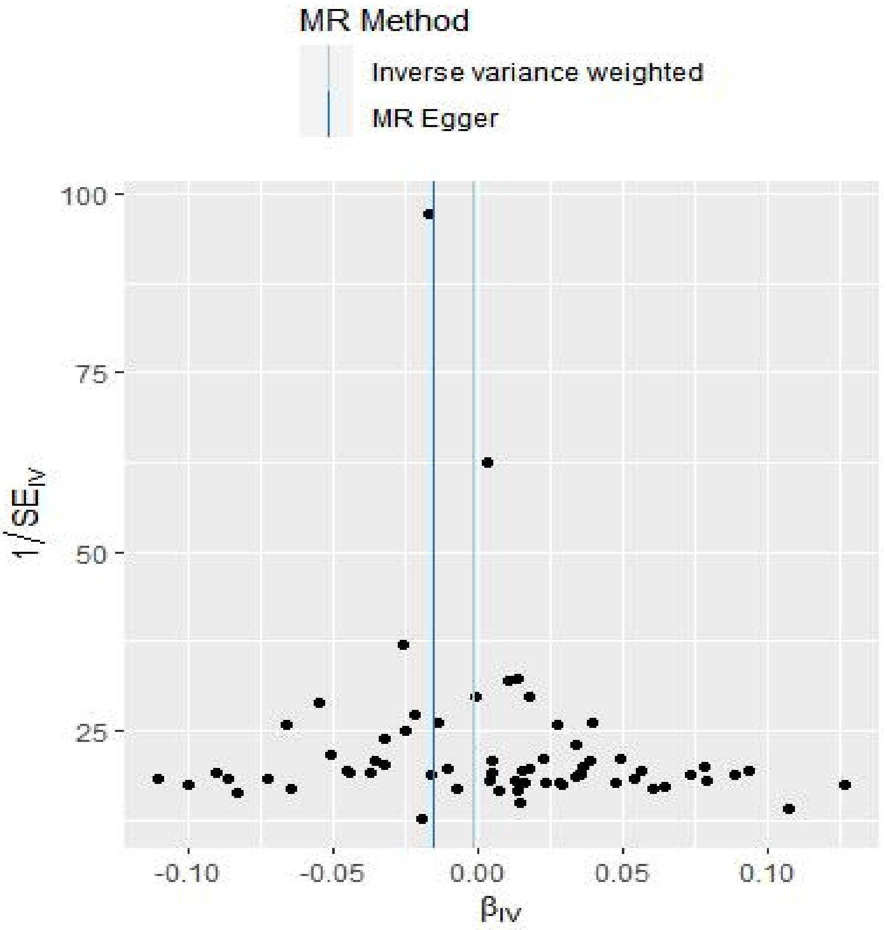

Figure 19: Leave-one-out plot to visualize causal effect of tryptophan on the risk of heart failure when leaving one SNP out.

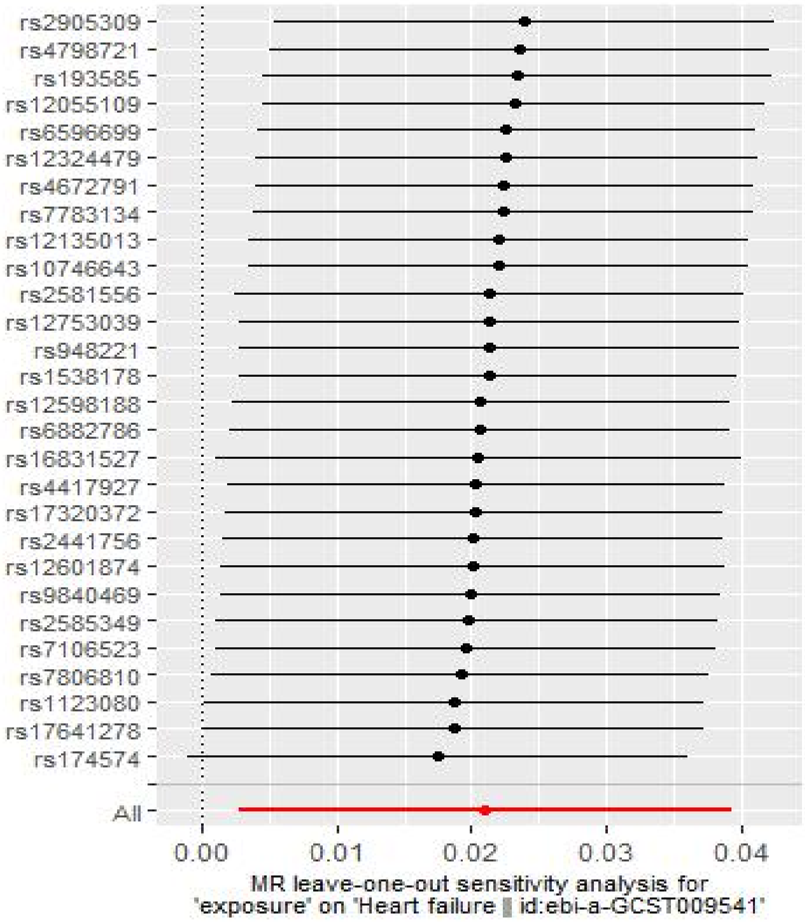

Figure 20: Funnel plots to visualize overall heterogeneity of Mendelian randomization (MR)

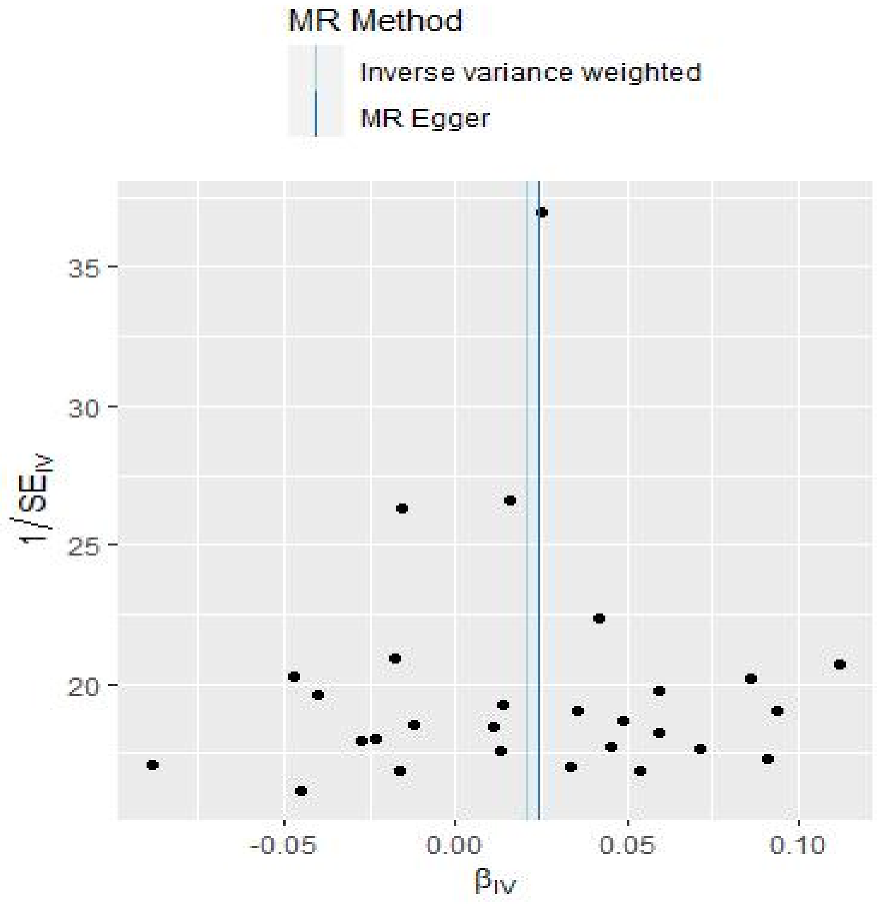

Figure 21: Leave-one-out plot to visualize causal effect of tyrosine on the risk of heart failure when leaving one SNP out.

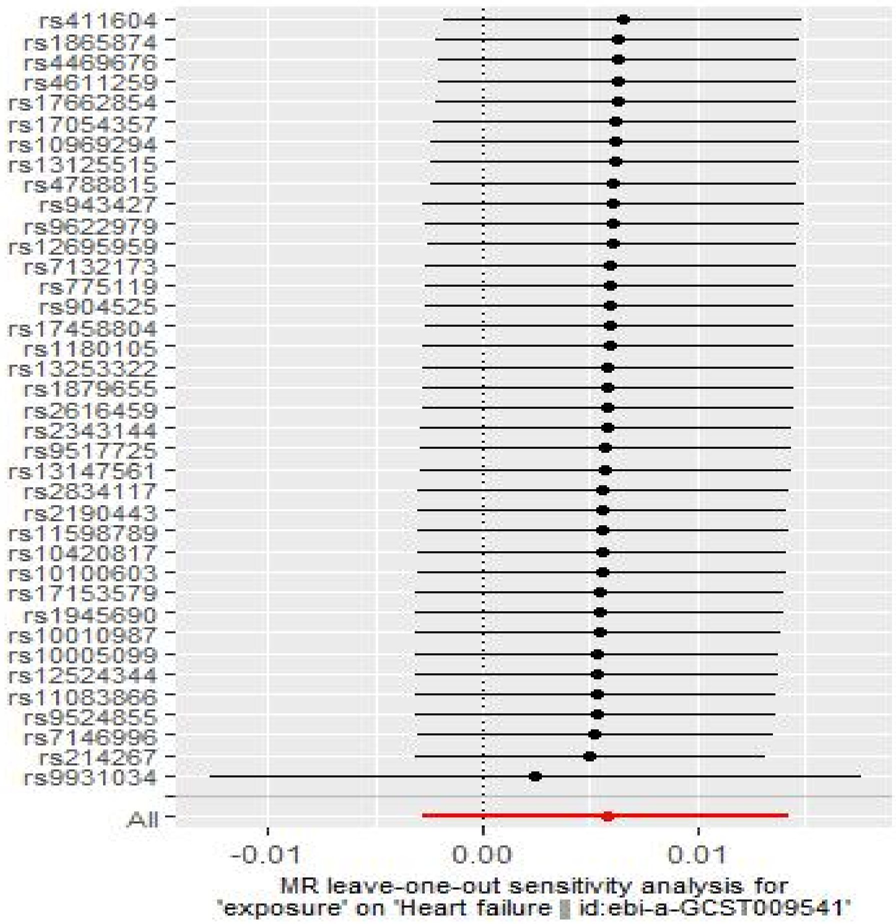

Figure 22: Funnel plots to visualize overall heterogeneity of Mendelian randomization (MR)

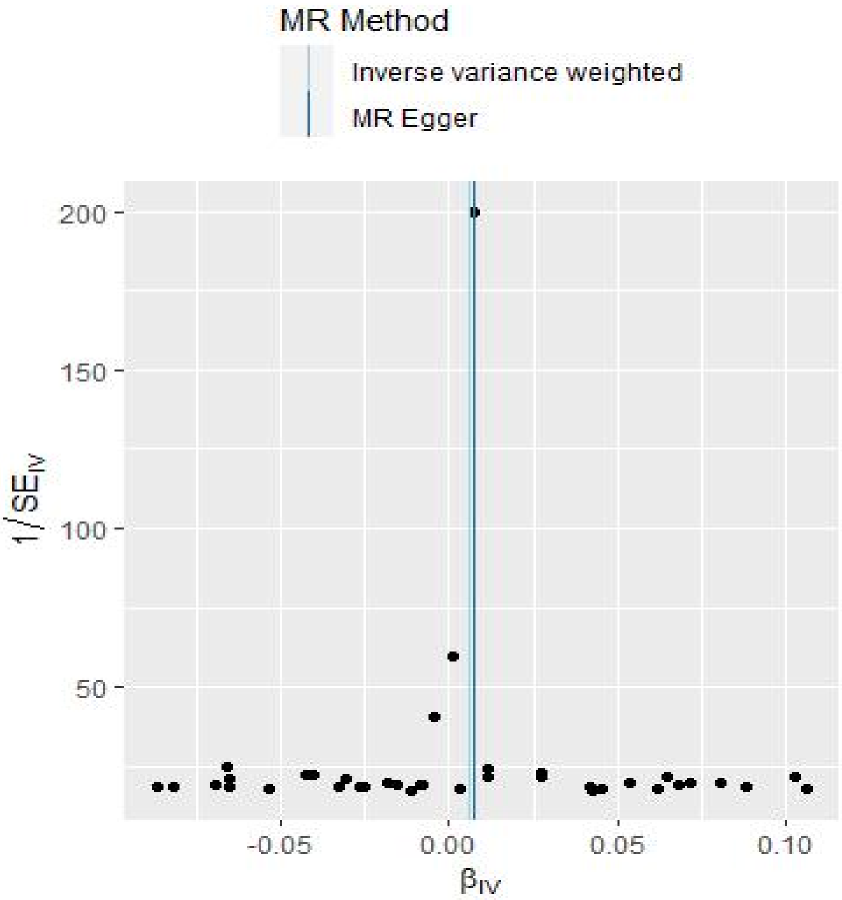

Figure 23: Leave-one-out plot to visualize causal effect of propionic acid on the risk of heart failure when leaving one SNP out.

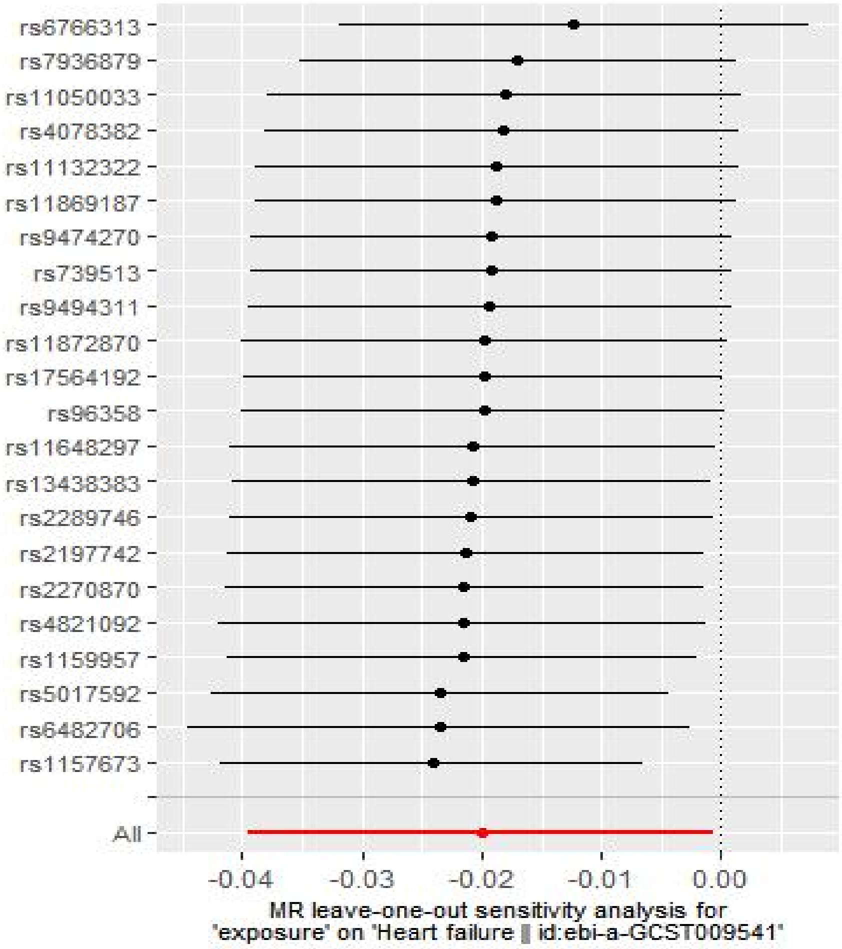

Figure 24: Funnel plots to visualize overall heterogeneity of Mendelian randomization (MR)

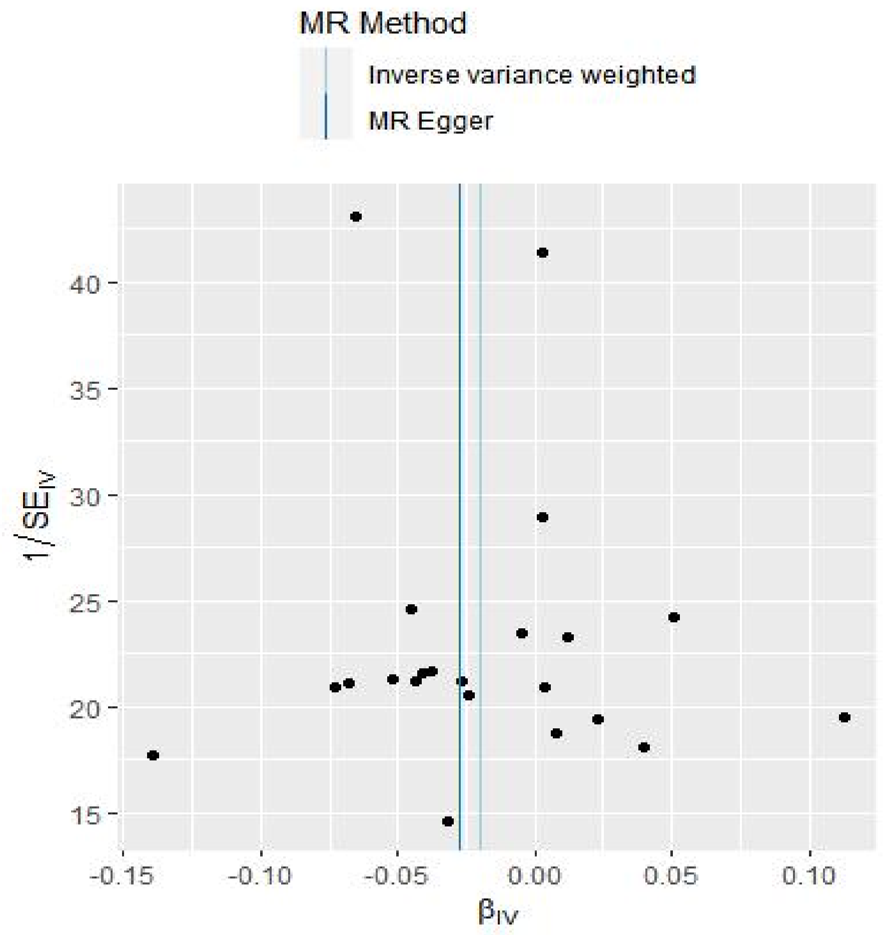

Figure 25: Leave-one-out plot to visualize causal effect of beta_hydroxybutyric acid on the risk of atrial fibrillation when leaving one SNP out.

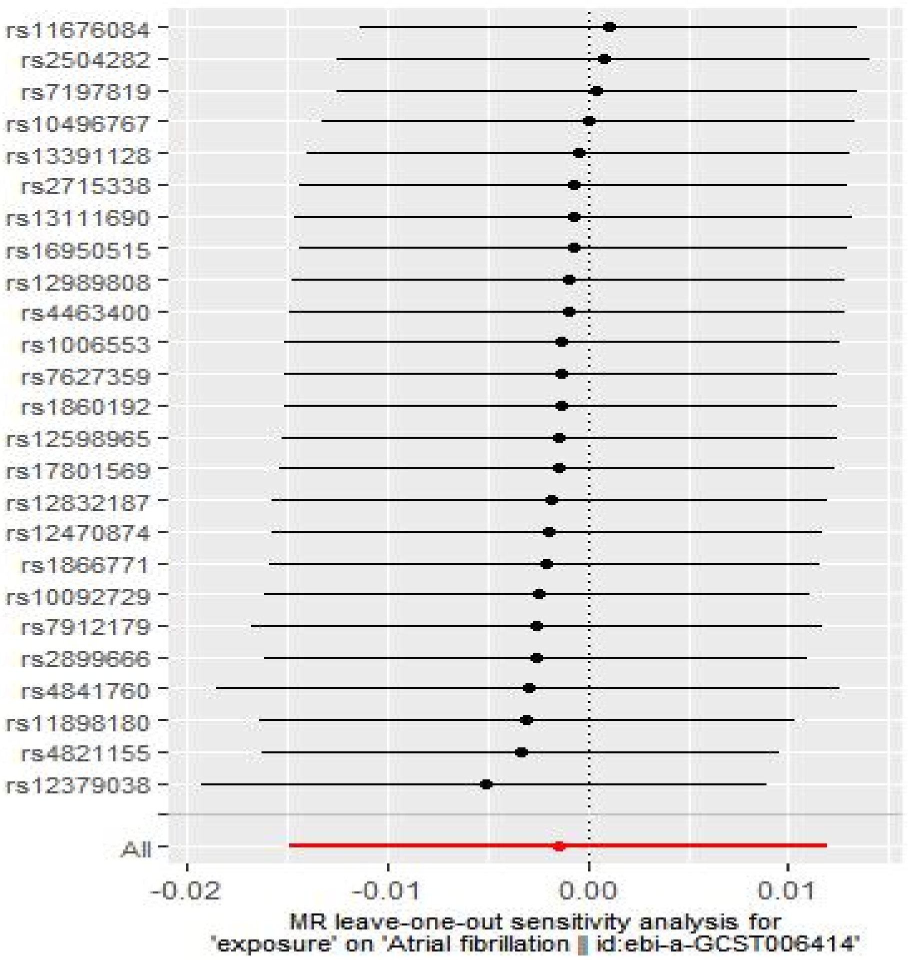

Figure 26: Funnel plots to visualize overall heterogeneity of Mendelian randomization (MR)

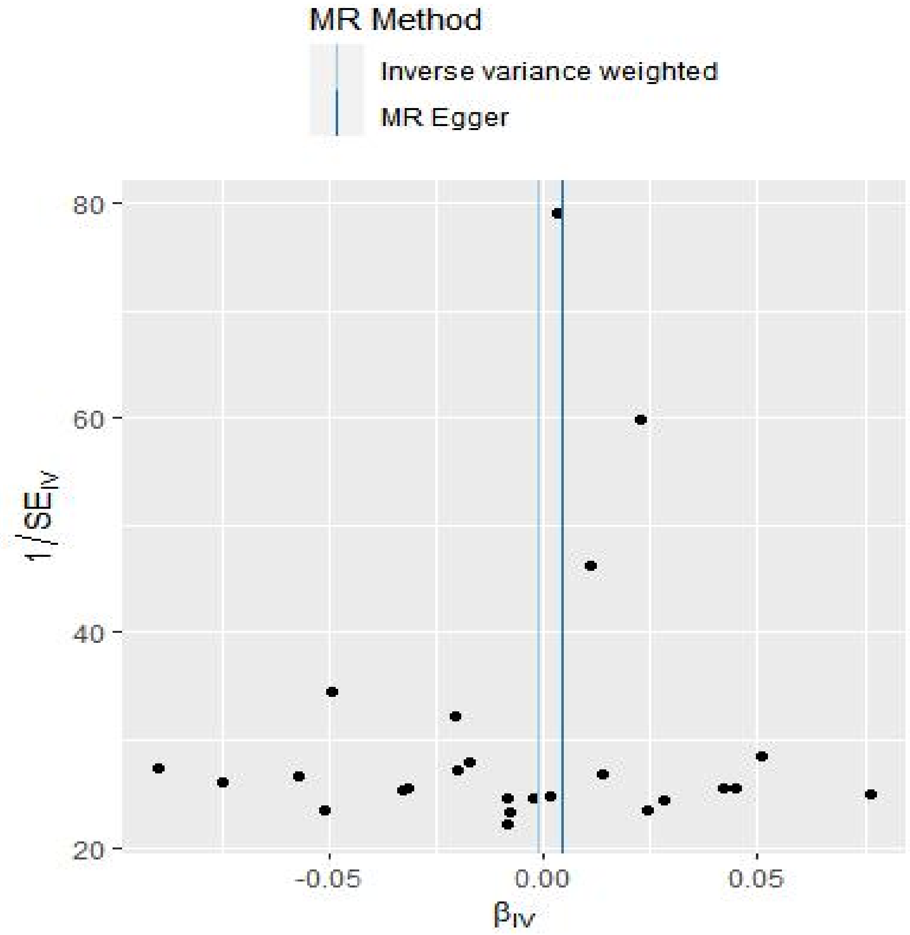

Figure 27: Leave-one-out plot to visualize causal effect of betaine on the risk of atrial fibrillation when leaving one SNP out.

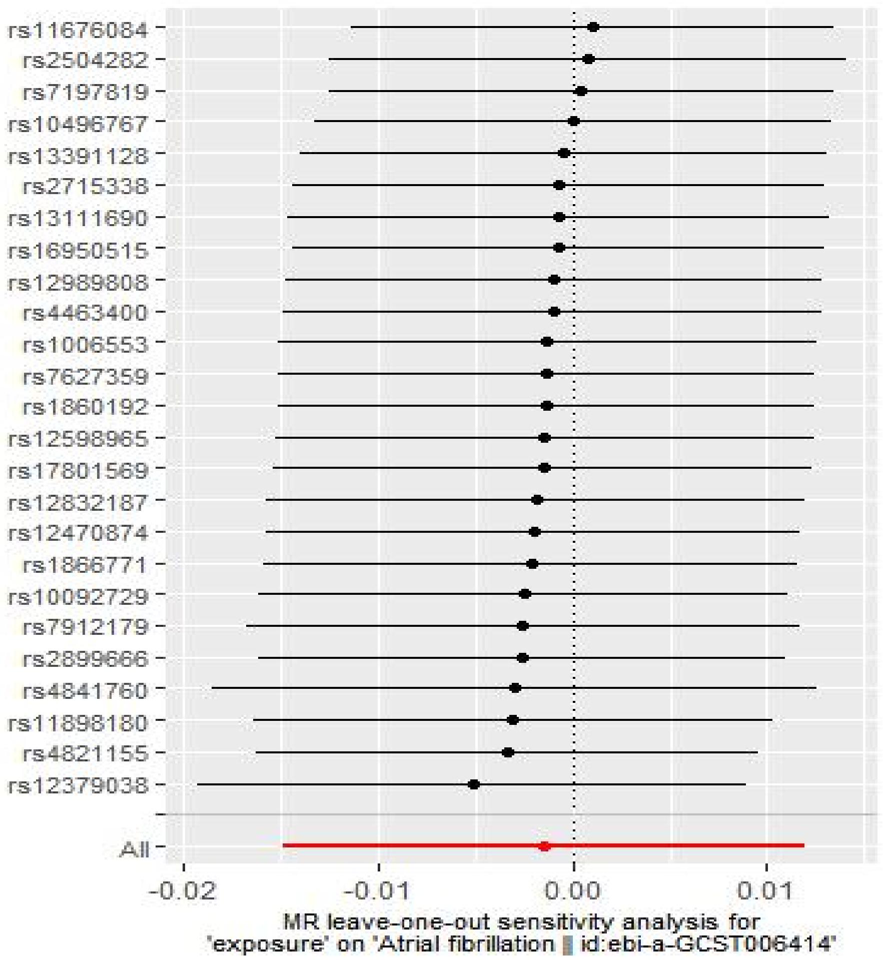

Figure 28: Funnel plots to visualize overall heterogeneity of Mendelian randomization (MR)

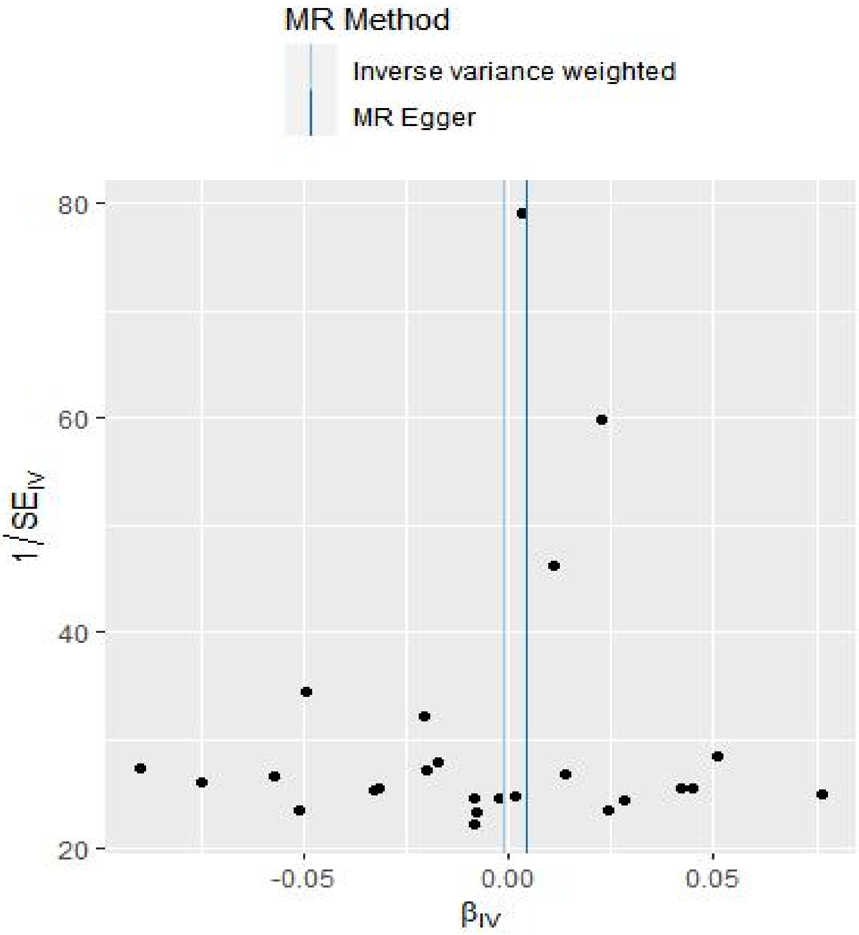

Figure 29: Leave-one-out plot to visualize causal effect of carnitine on the risk of atrial fibrillation when leaving one SNP out.

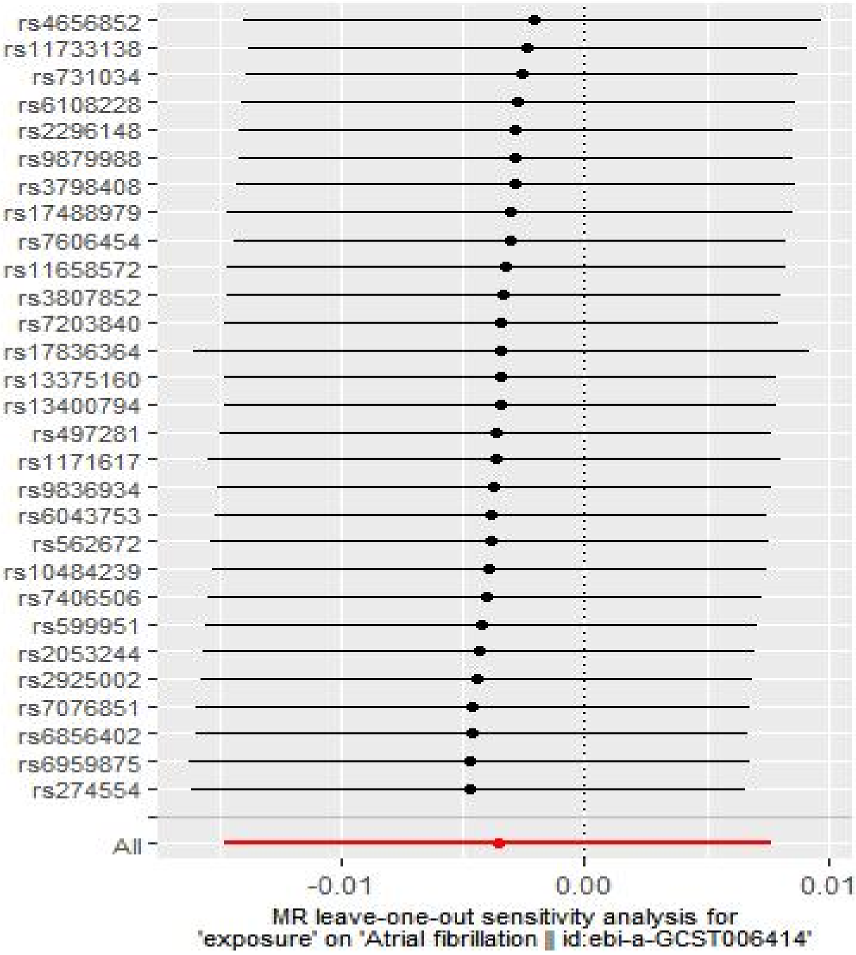

Figure 30: Funnel plots to visualize overall heterogeneity of Mendelian randomization (MR)

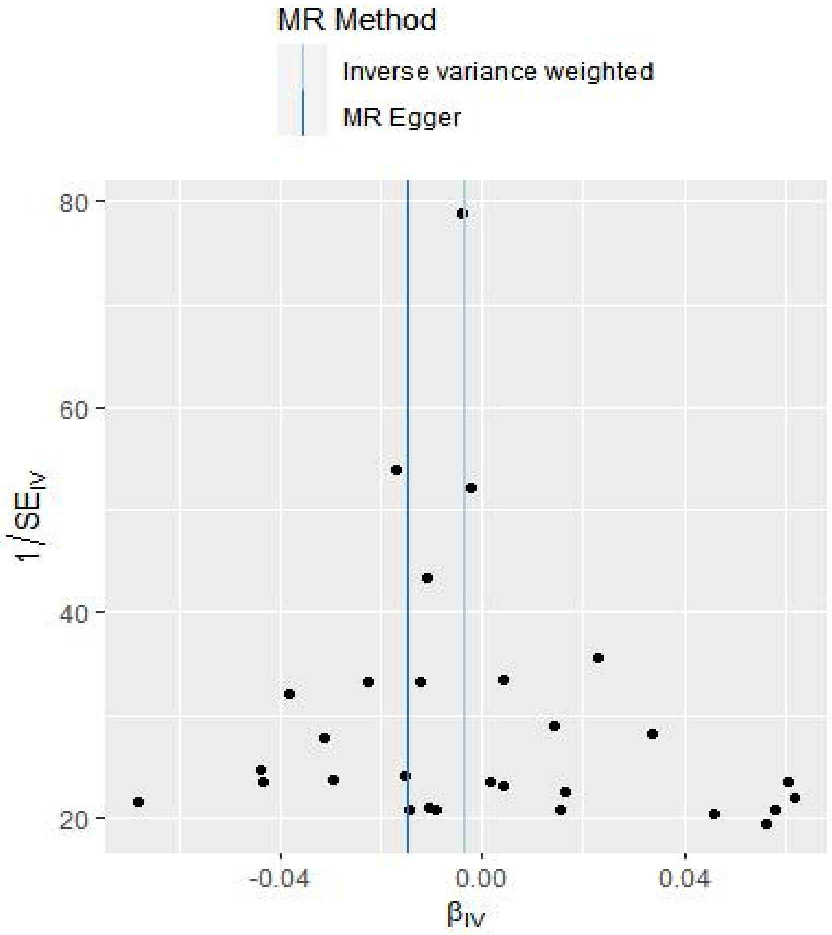

Figure 31: Leave-one-out plot to visualize causal effect of choline on the risk of atrial fibrillation when leaving one SNP out.

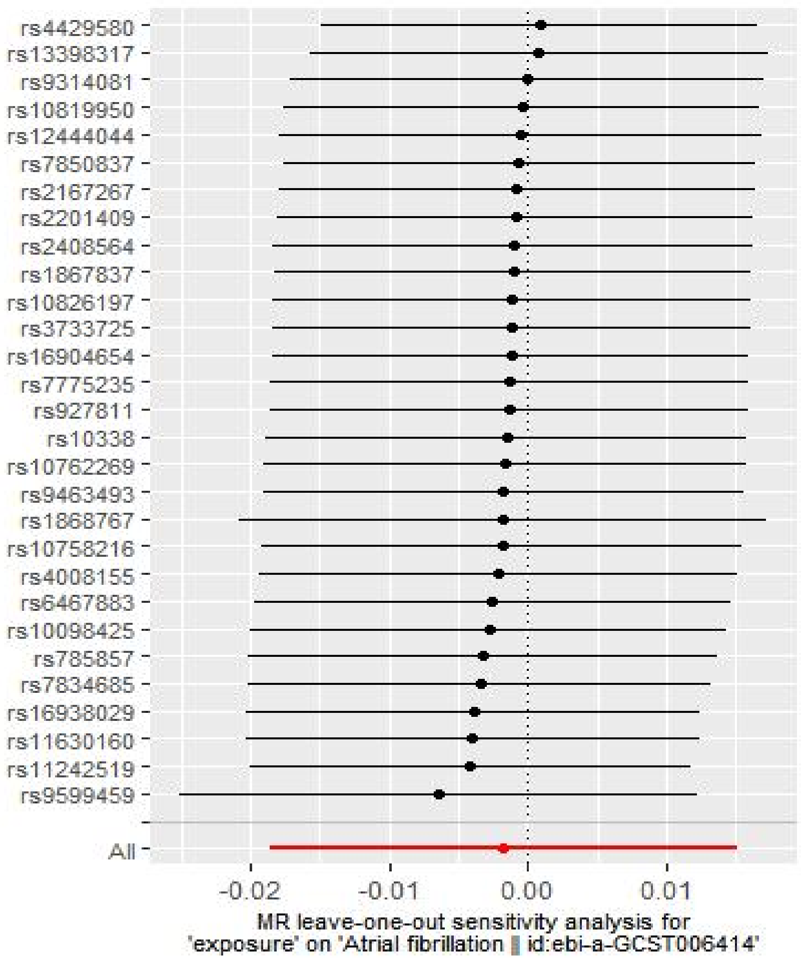

Figure 32: Funnel plots to visualize overall heterogeneity of Mendelian randomization (MR)

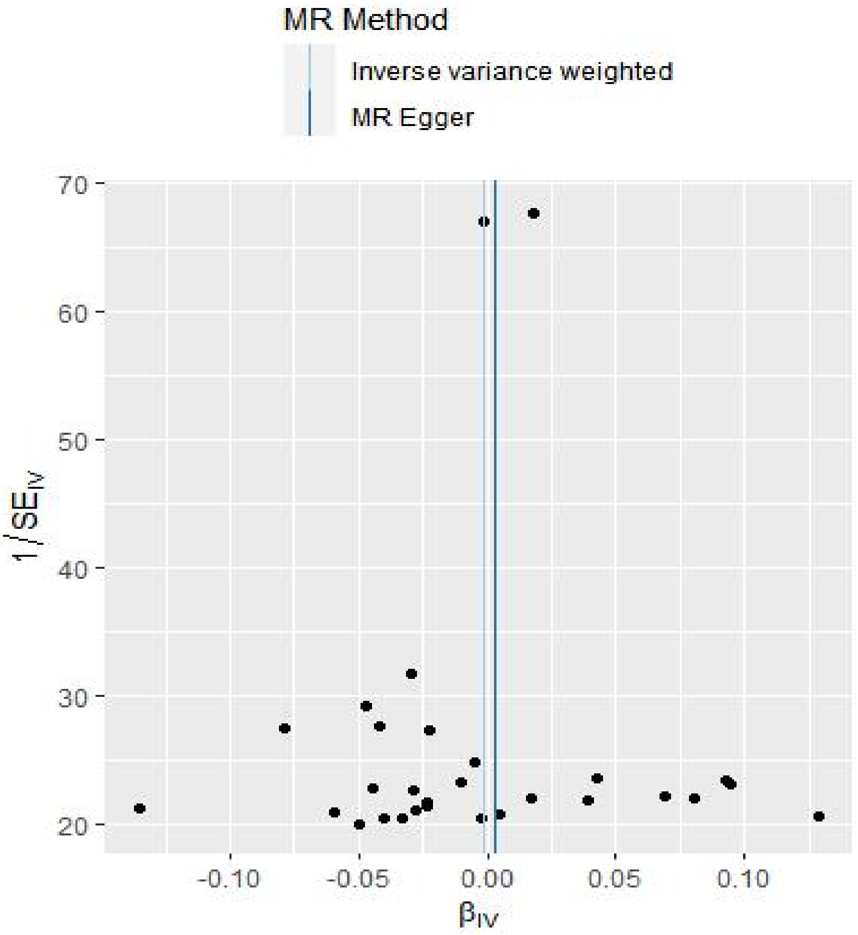

Figure 33: Leave-one-out plot to visualize causal effect of glutamate on the risk of atrial fibrillation when leaving one SNP out.

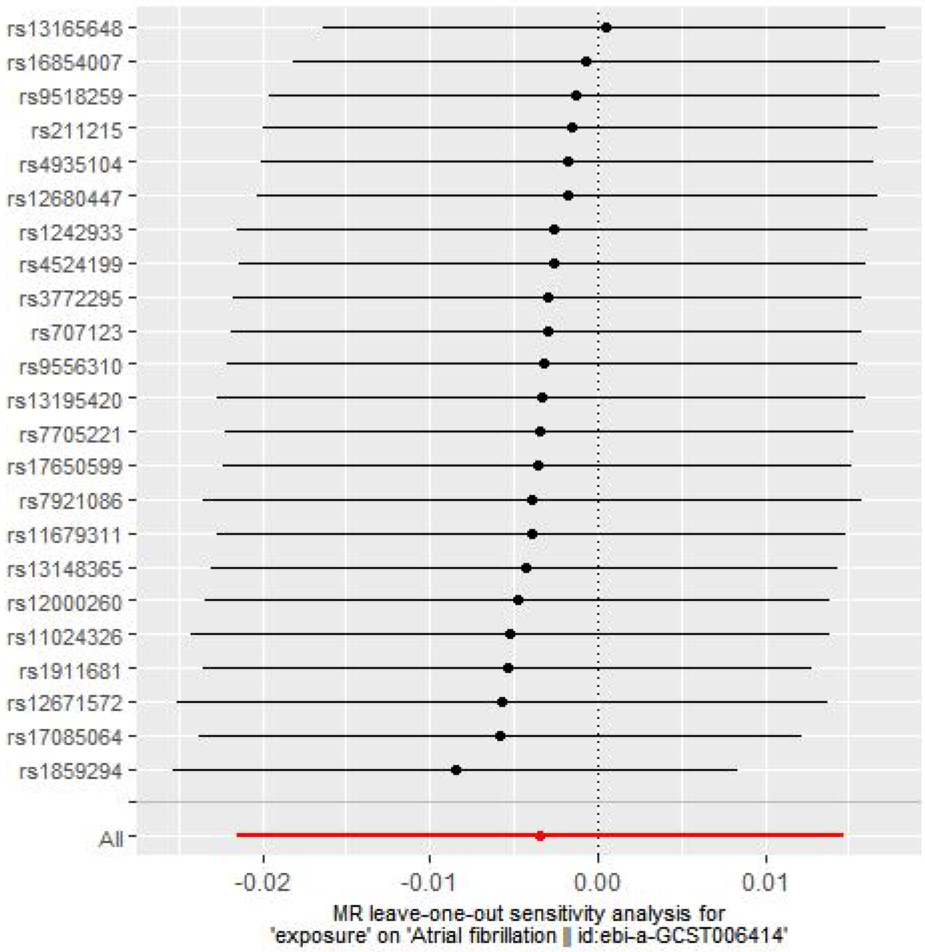

Figure 34: Funnel plots to visualize overall heterogeneity of Mendelian randomization (MR)

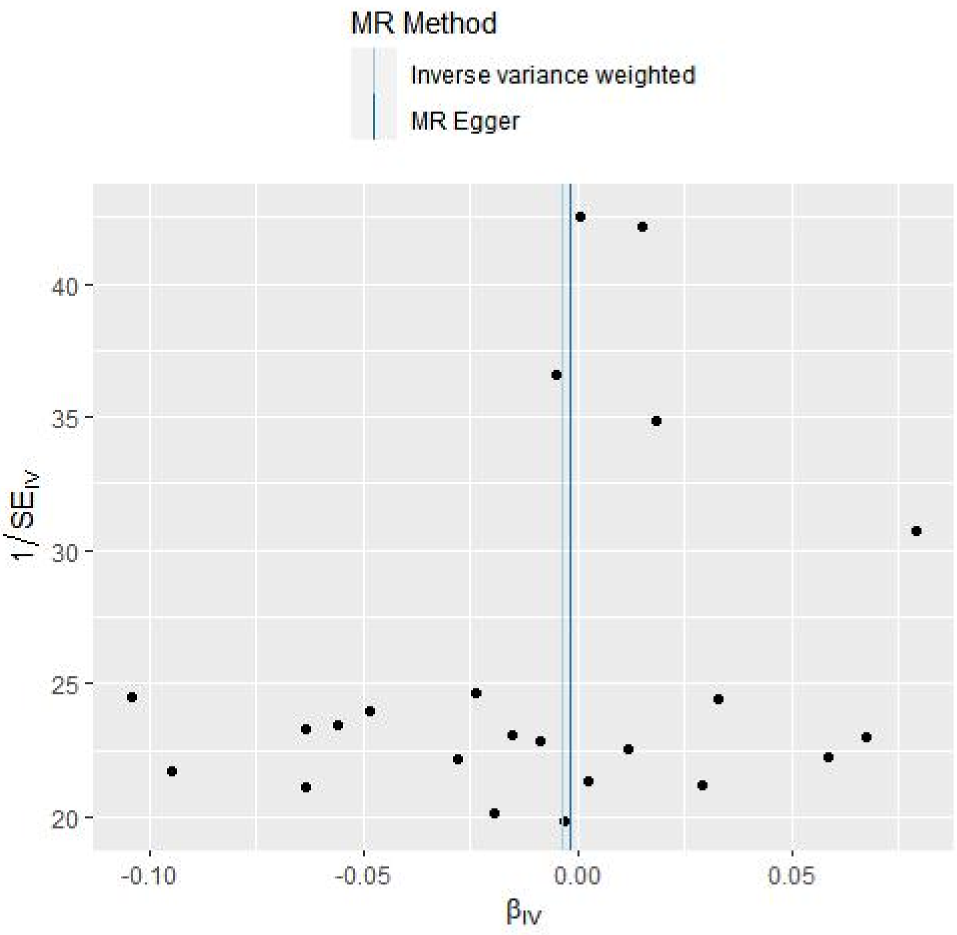

Figure 35: Leave-one-out plot to visualize causal effect of kynuremine on the risk of atrial fibrillation when leaving one SNP out.

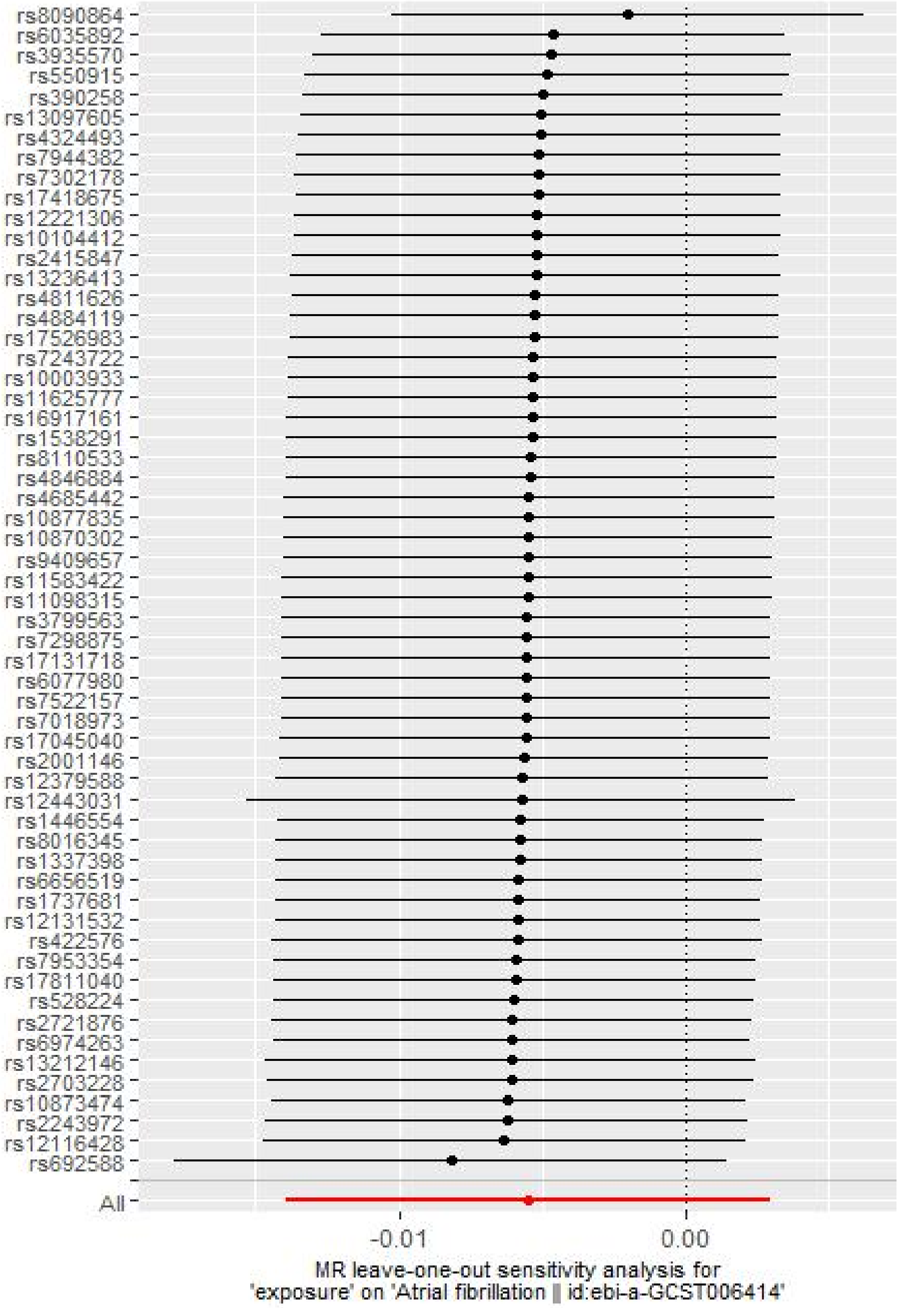

Figure 36: Funnel plots to visualize overall heterogeneity of Mendelian randomization (MR)

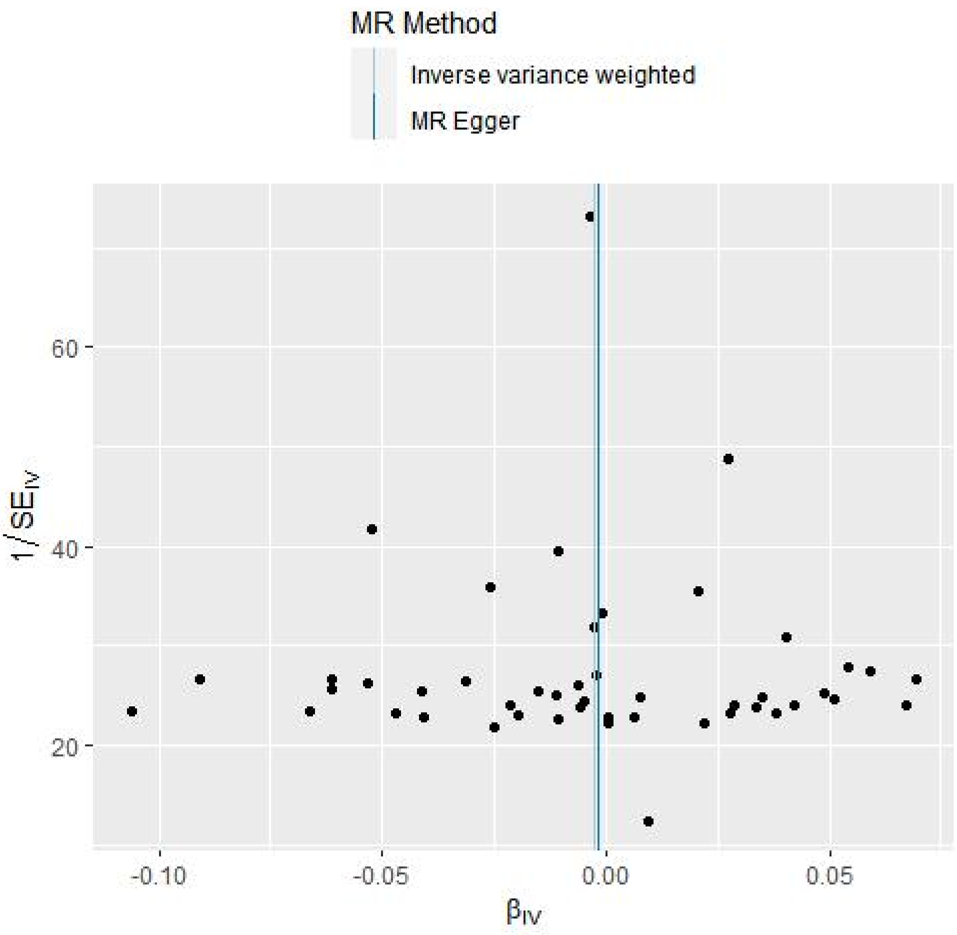

Figure 37: Leave-one-out plot to visualize causal effect of phenylalanine on the risk of atrial fibrillation when leaving one SNP out.

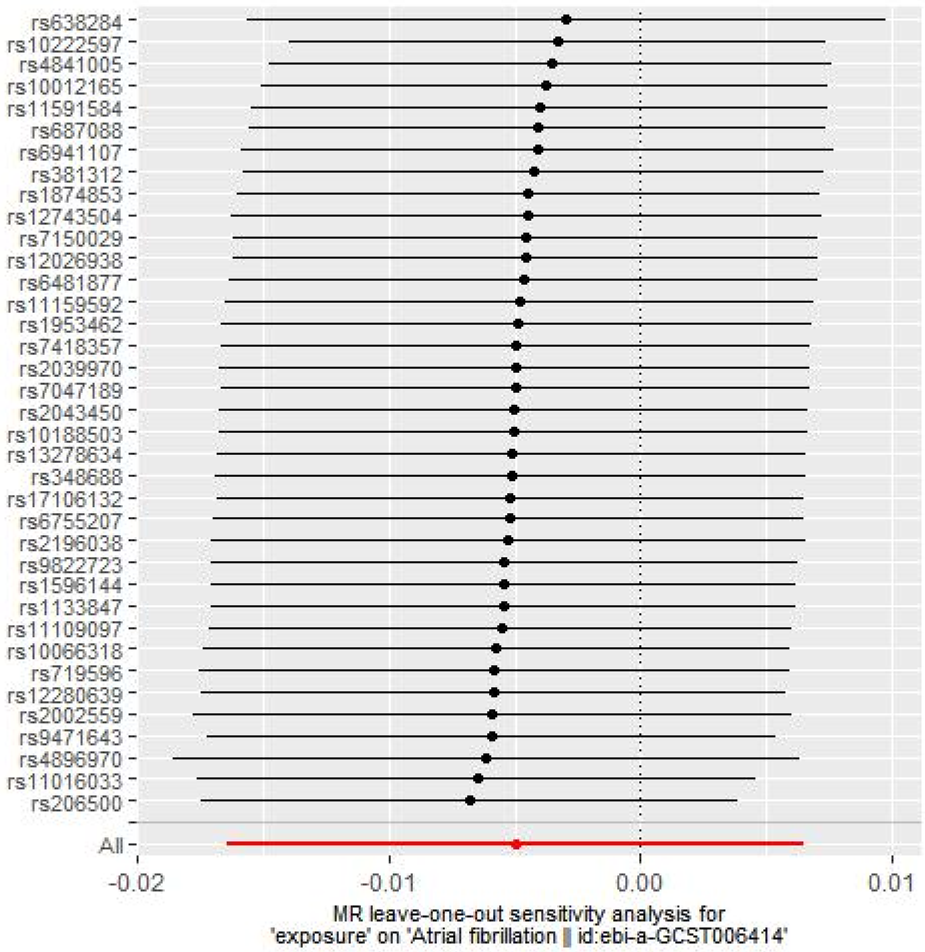

Figure 38: Funnel plots to visualize overall heterogeneity of Mendelian randomization (MR)

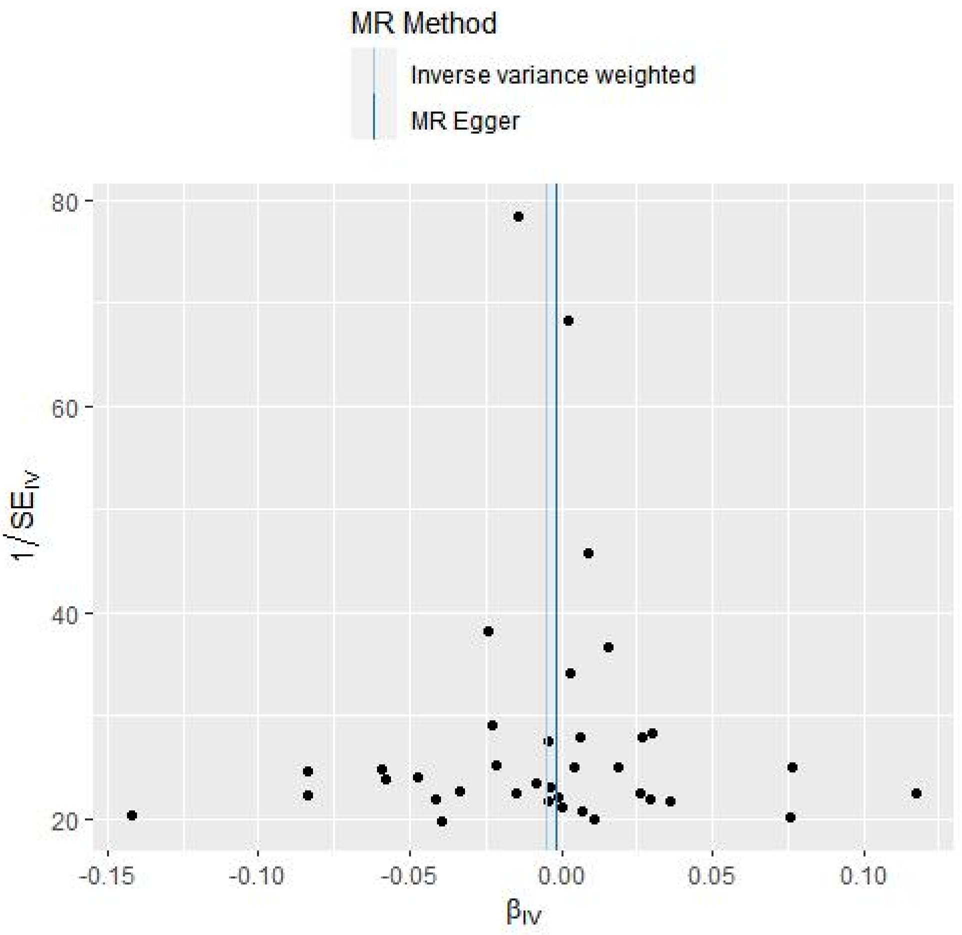

Figure 39: Leave-one-out plot to visualize causal effect of serotonin on the risk of atrial fibrillation when leaving one SNP out.

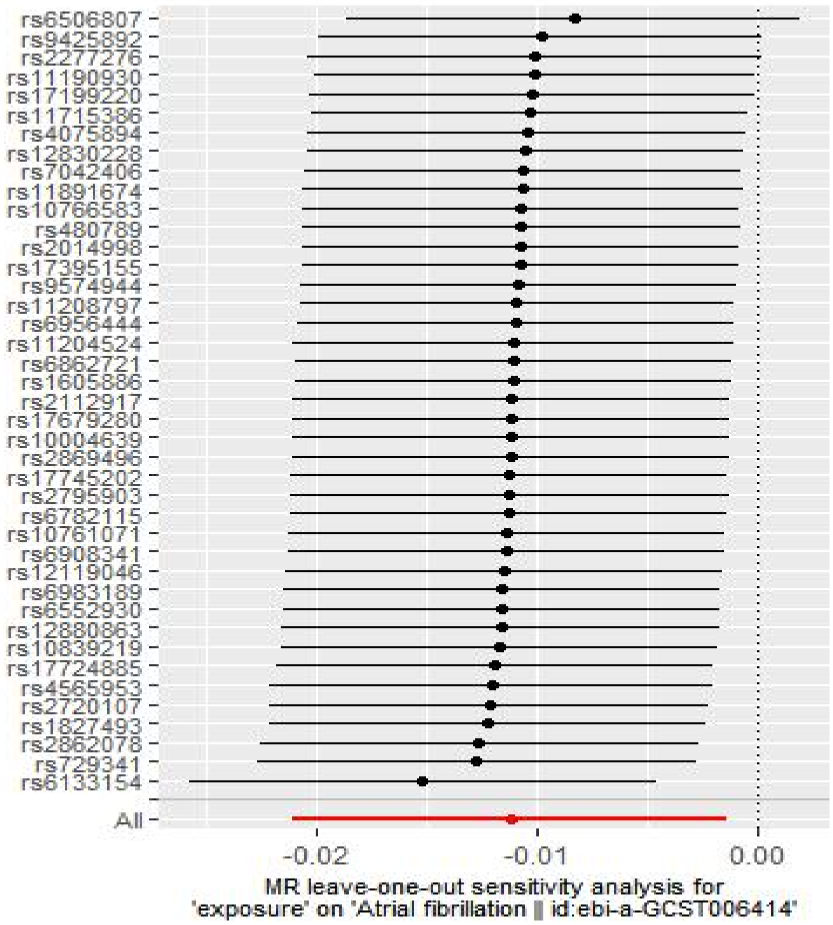

Figure 40: Funnel plots to visualize overall heterogeneity of Mendelian randomization (MR)

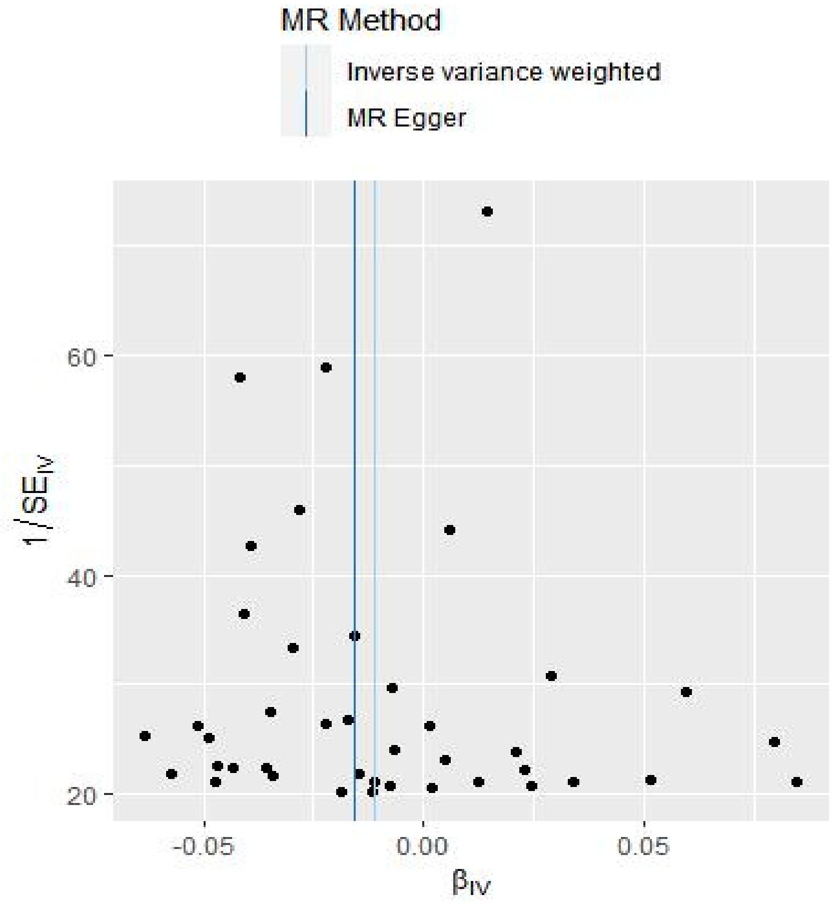

Figure 41: Leave-one-out plot to visualize causal effect of trimethylamine_N_oxide on the risk of atrial fibrillation when leaving one SNP out.

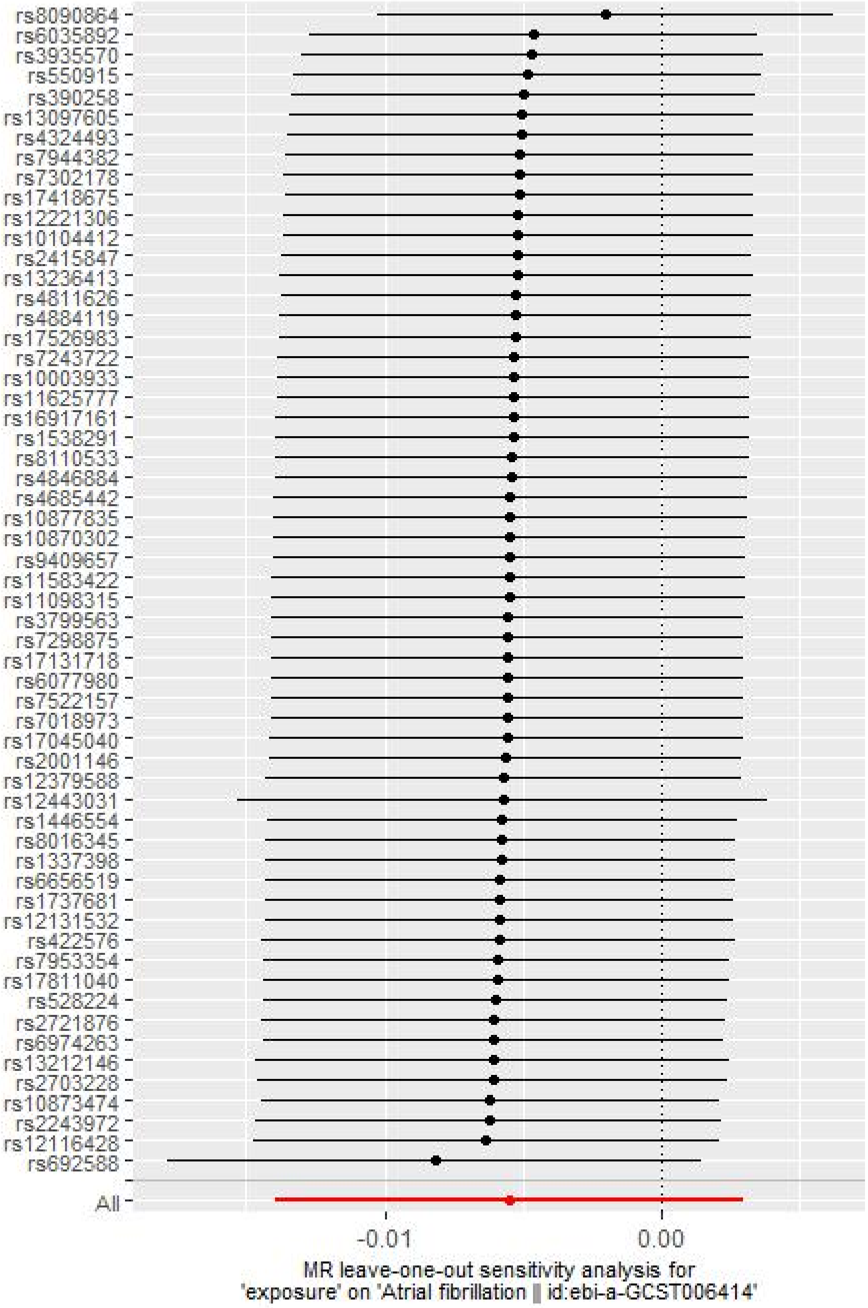

Figure 42: Funnel plots to visualize overall heterogeneity of Mendelian randomization (MR)

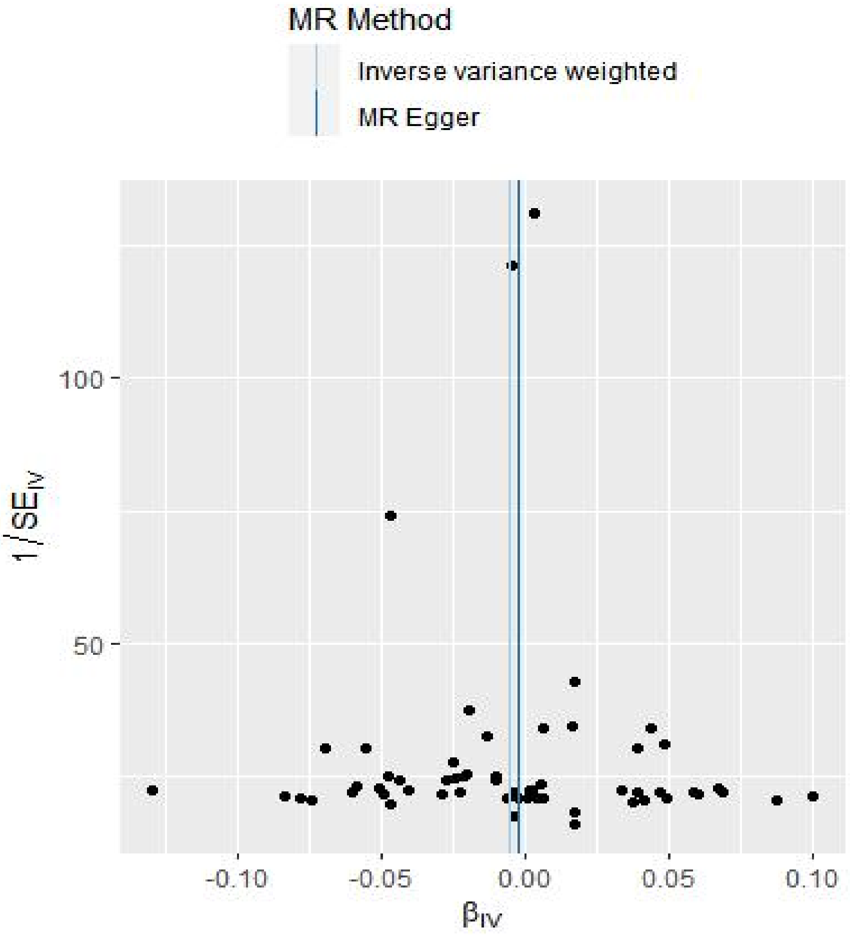

Figure 43: Leave-one-out plot to visualize causal effect of tryptophan on the risk of atrial fibrillation when leaving one SNP out.

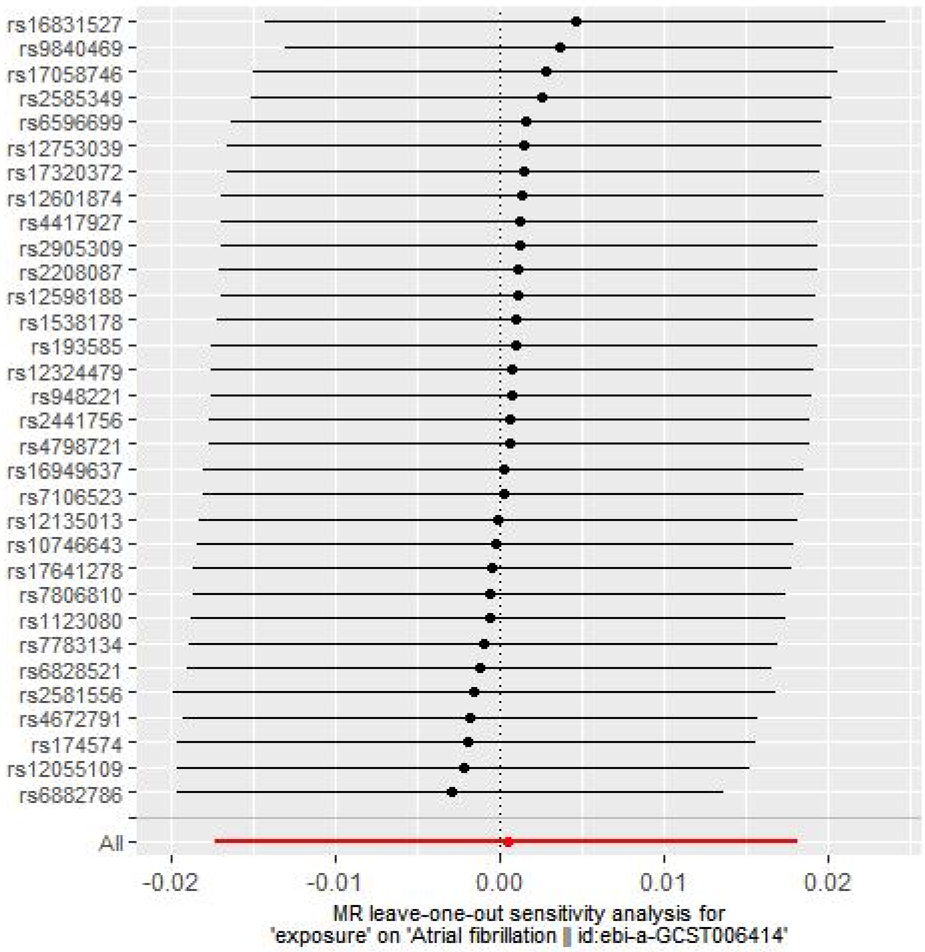

Figure 44: Funnel plots to visualize overall heterogeneity of Mendelian randomization (MR)

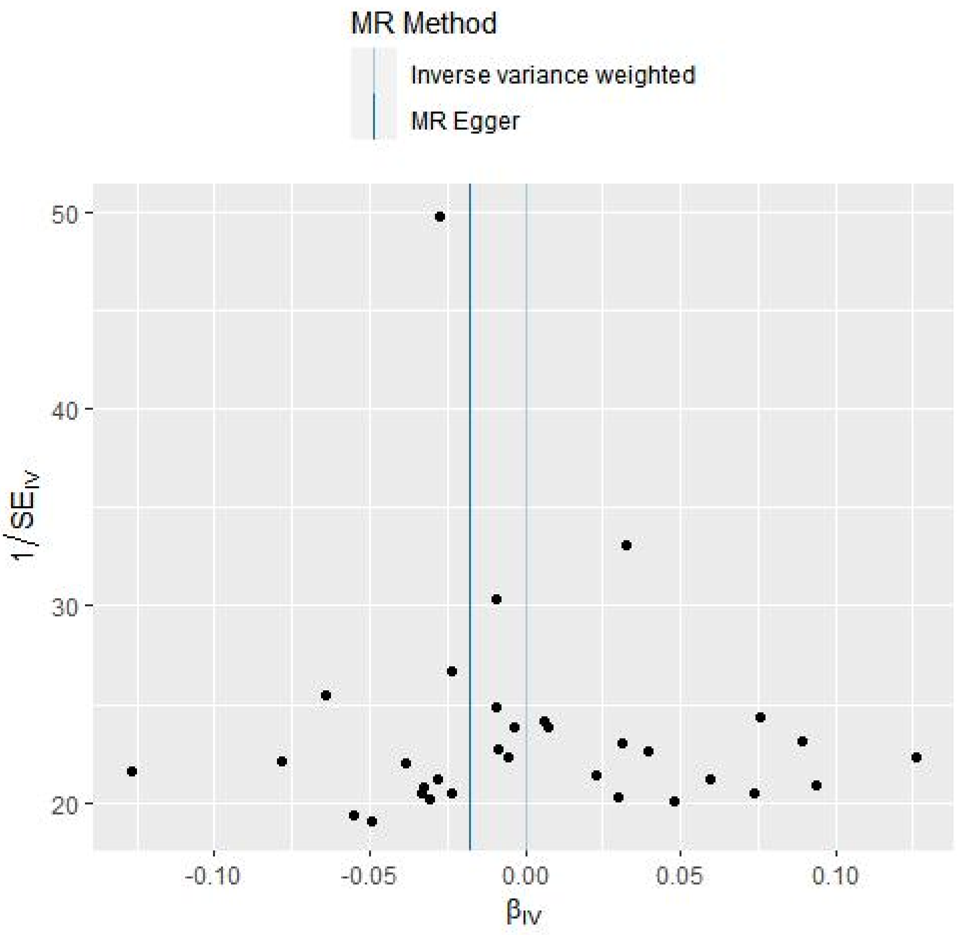

Figure 45: Leave-one-out plot to visualize causal effect of tyrosine on the risk of atrial fibrillation when leaving one SNP out.

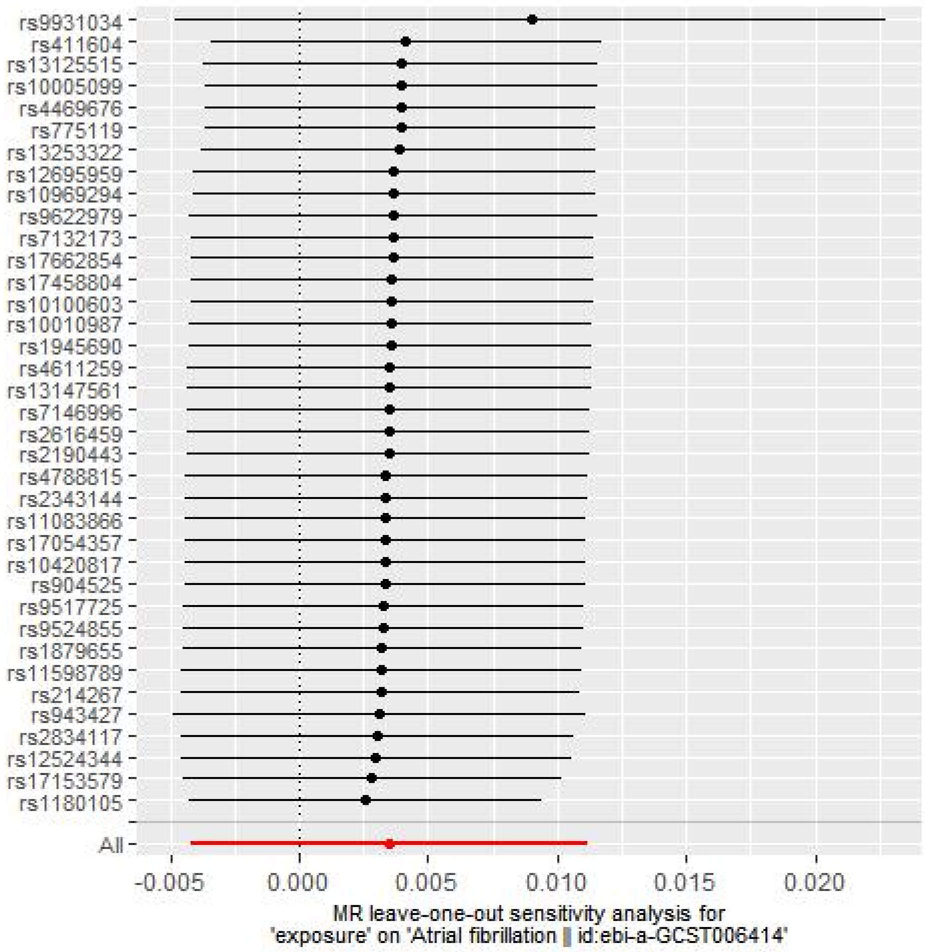

Figure 46: Funnel plots to visualize overall heterogeneity of Mendelian randomization (MR)

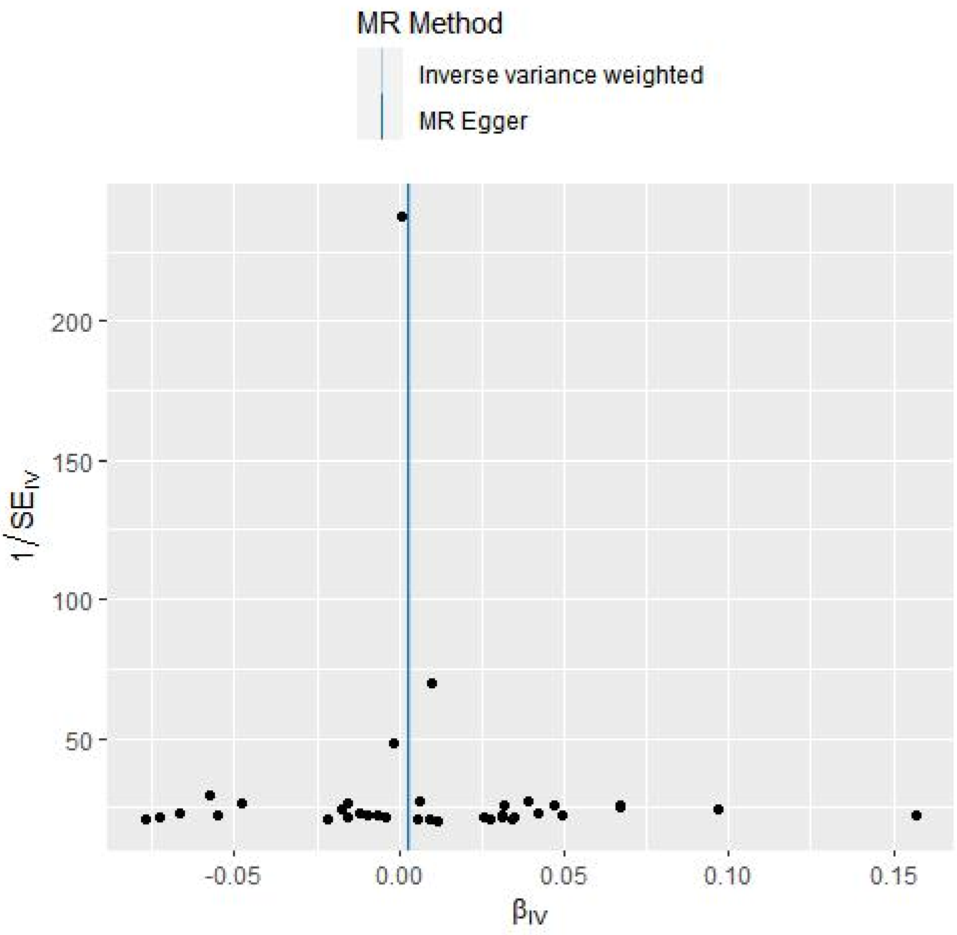

Figure 47: Leave-one-out plot to visualize causal effect of propionic acid on the risk of atrial fibrillation when leaving one SNP out.

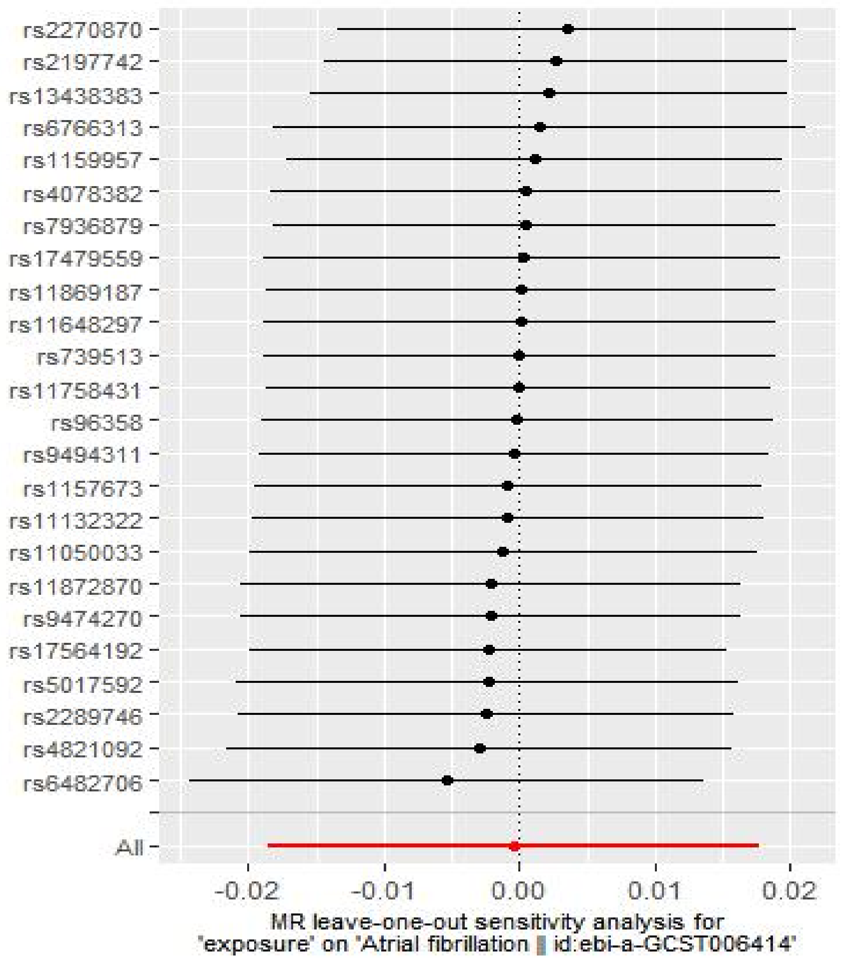

Figure 48: Funnel plots to visualize overall heterogeneity of Mendelian randomization (MR)

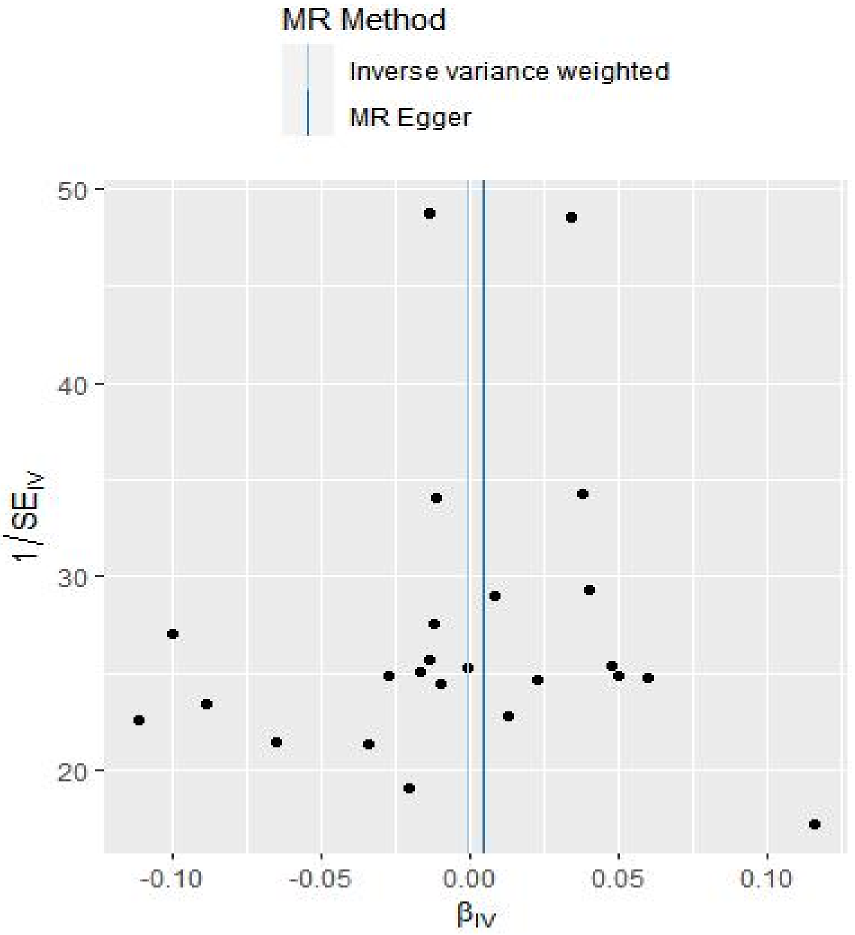

Figure 49: Leave-one-out plot to visualize causal effect of beta_hydroxybutyric acid on the risk of hypertrophic cardiomyopathy when leaving one SNP out.

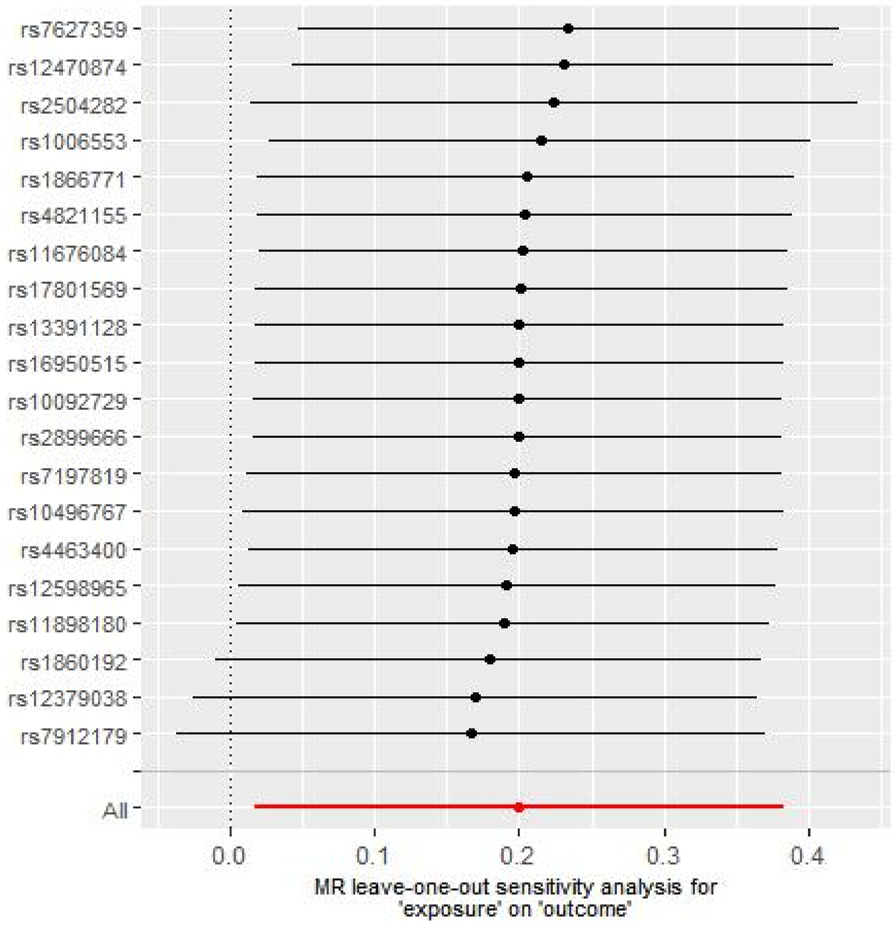

Figure 50: Funnel plots to visualize overall heterogeneity of Mendelian randomization (MR)

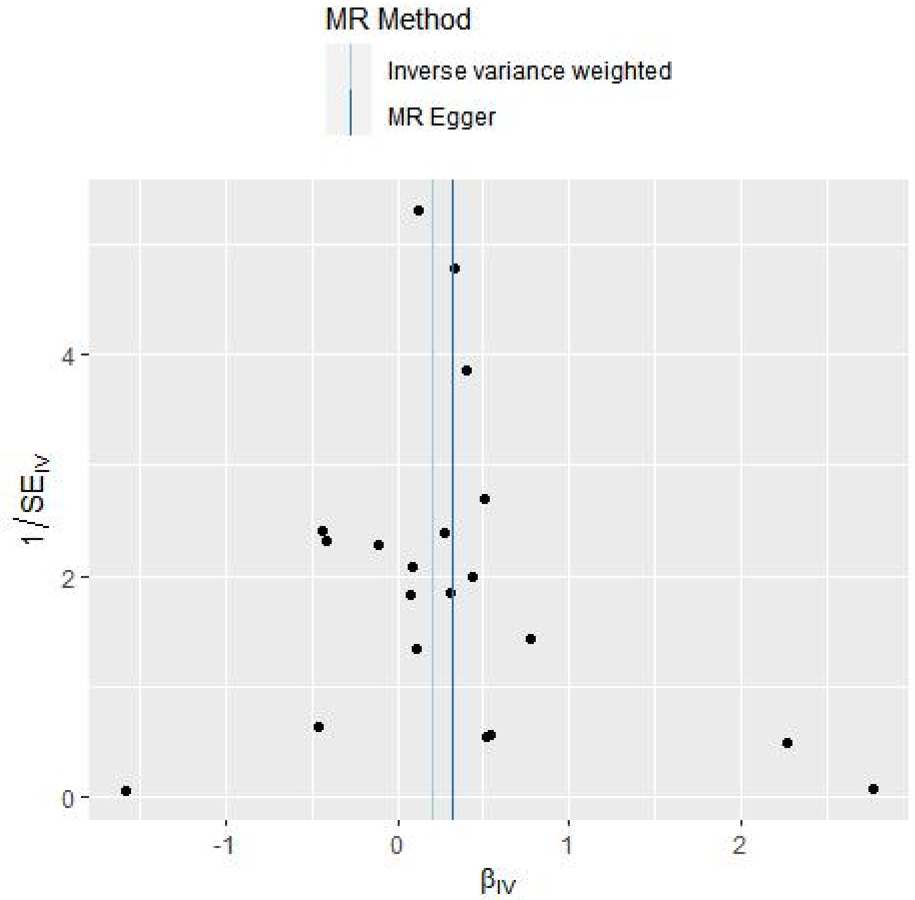

Figure 51: Leave-one-out plot to visualize causal effect of betaine on the risk of hypertrophic cardiomyopathy when leaving one SNP out.

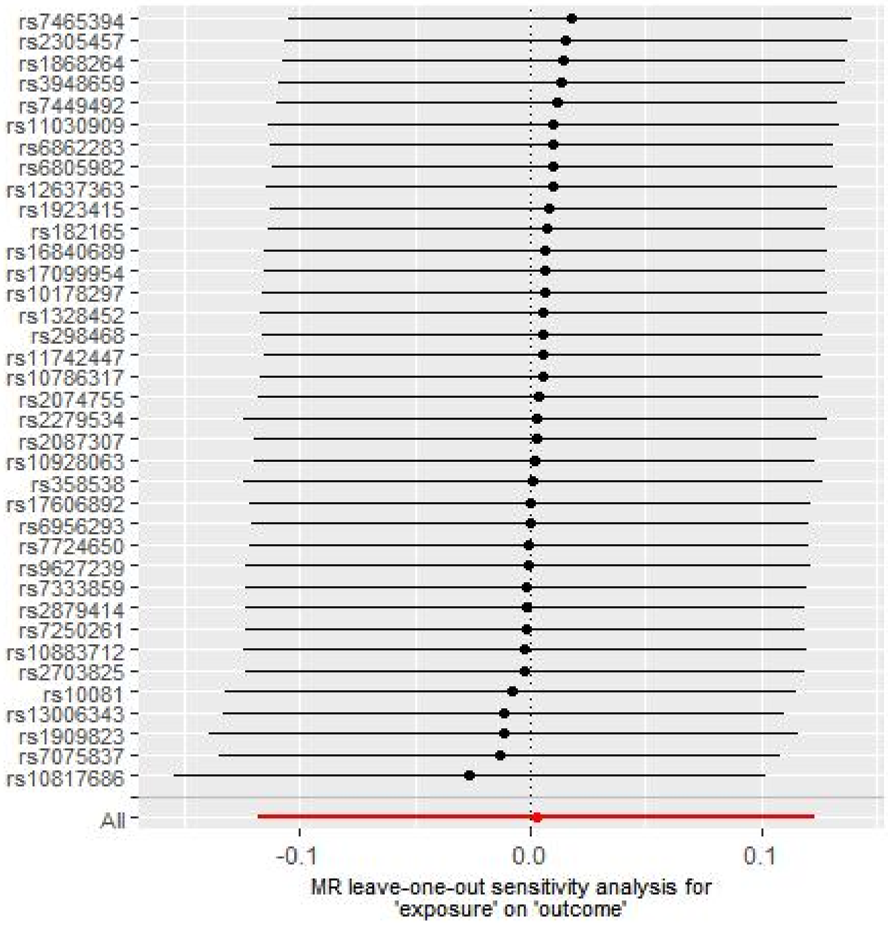

Figure 52: Funnel plots to visualize overall heterogeneity of Mendelian randomization (MR)

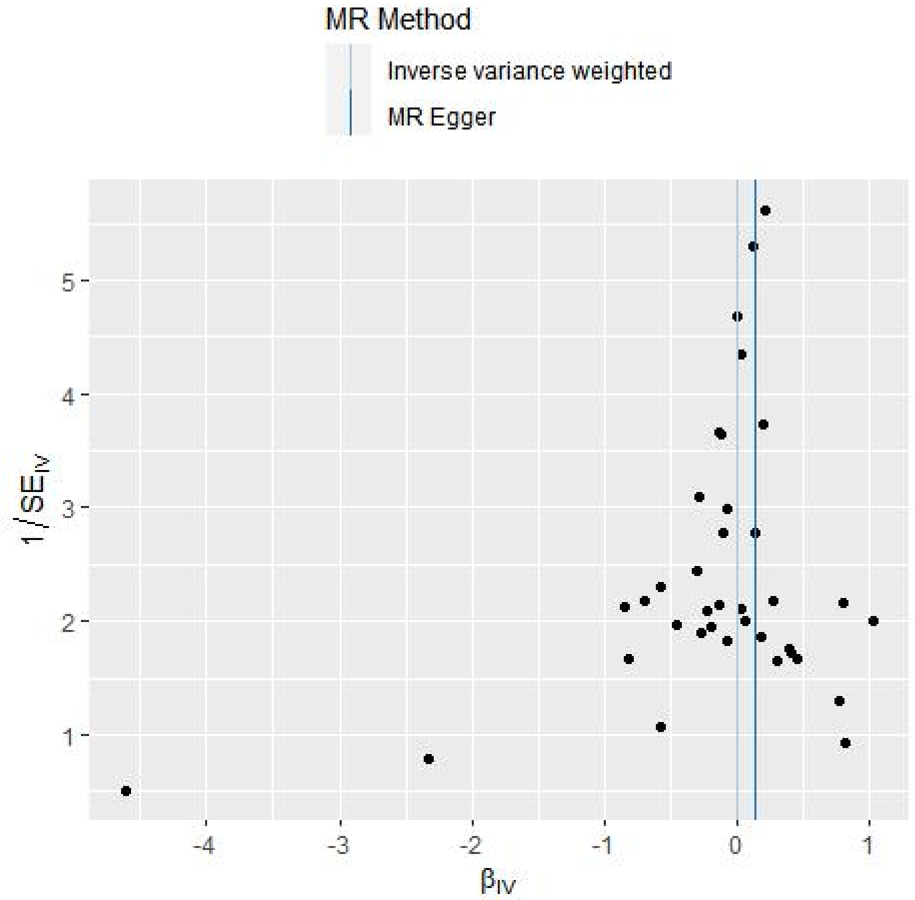

Figure 53: Leave-one-out plot to visualize causal effect of carnitine on the risk of hypertrophic cardiomyopathy when leaving one SNP out.

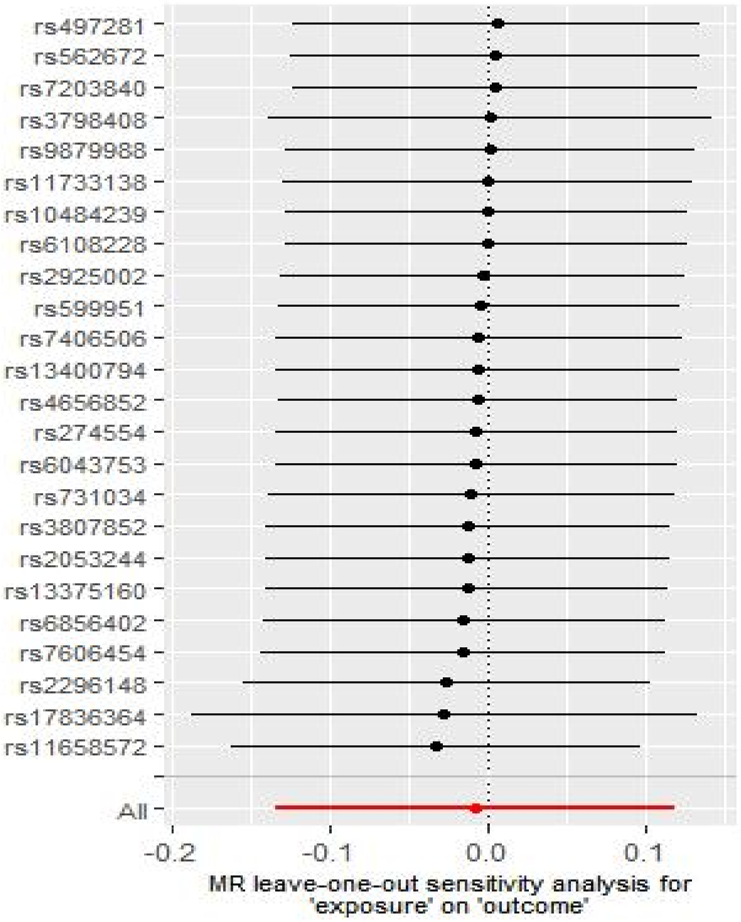

Figure 54: Funnel plots to visualize overall heterogeneity of Mendelian randomization (MR)

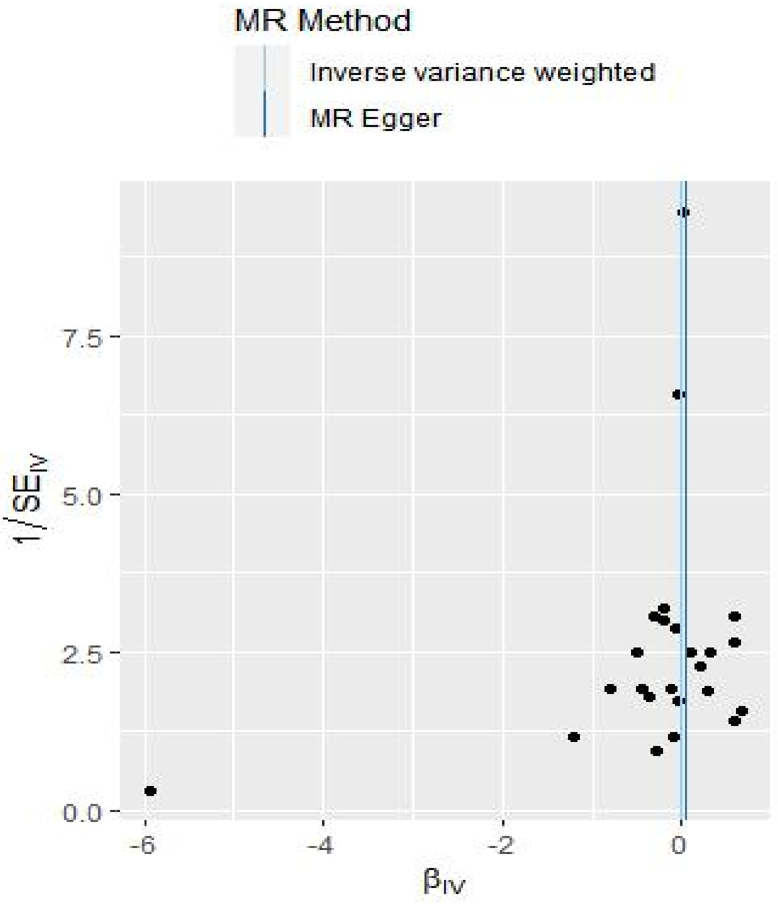

Figure 55: Leave-one-out plot to visualize causal effect of choline on the risk of hypertrophic cardiomyopathy when leaving one SNP out.

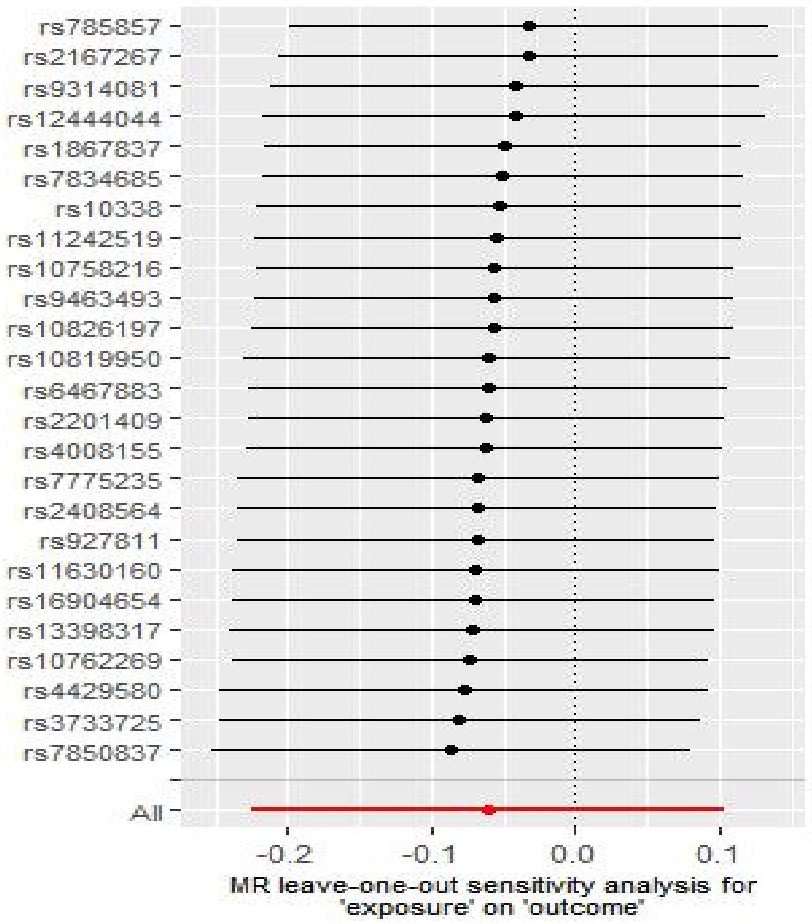

Figure 56: Funnel plots to visualize overall heterogeneity of Mendelian randomization (MR)

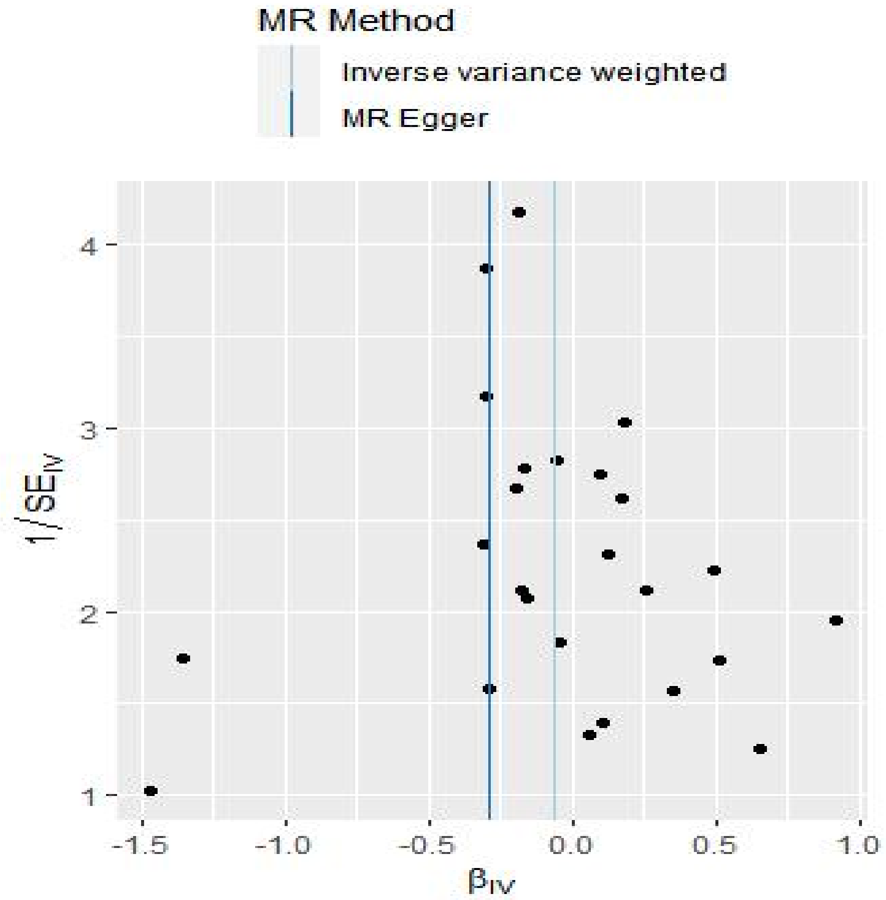

Figure 57: Leave-one-out plot to visualize causal effect of glutamate on the risk of hypertrophic cardiomyopathy when leaving one SNP out.

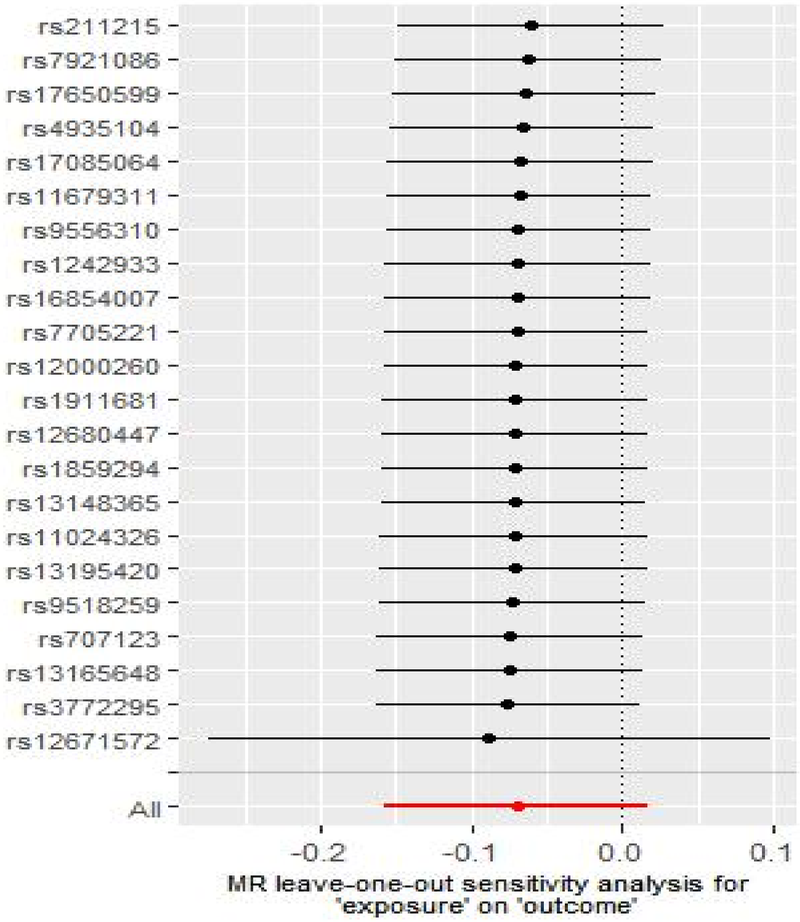

Figure 58: Funnel plots to visualize overall heterogeneity of Mendelian randomization (MR)

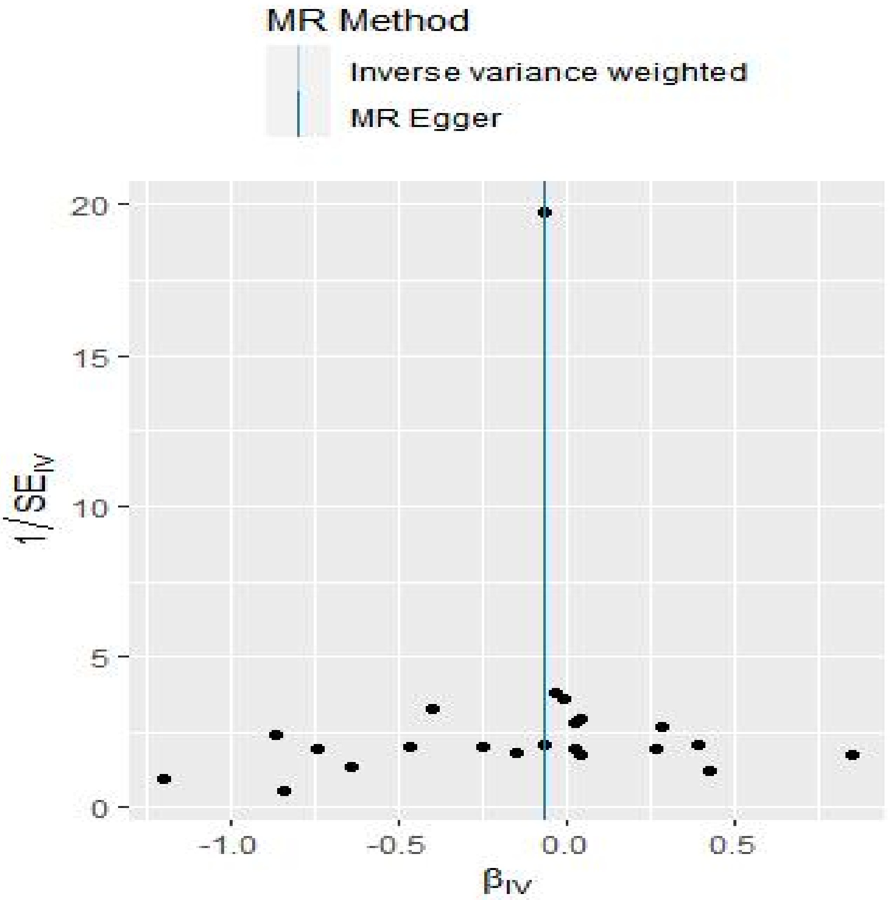

Figure 59: Leave-one-out plot to visualize causal effect of kynuremine on the risk of hypertrophic cardiomyopathy when leaving one SNP out.

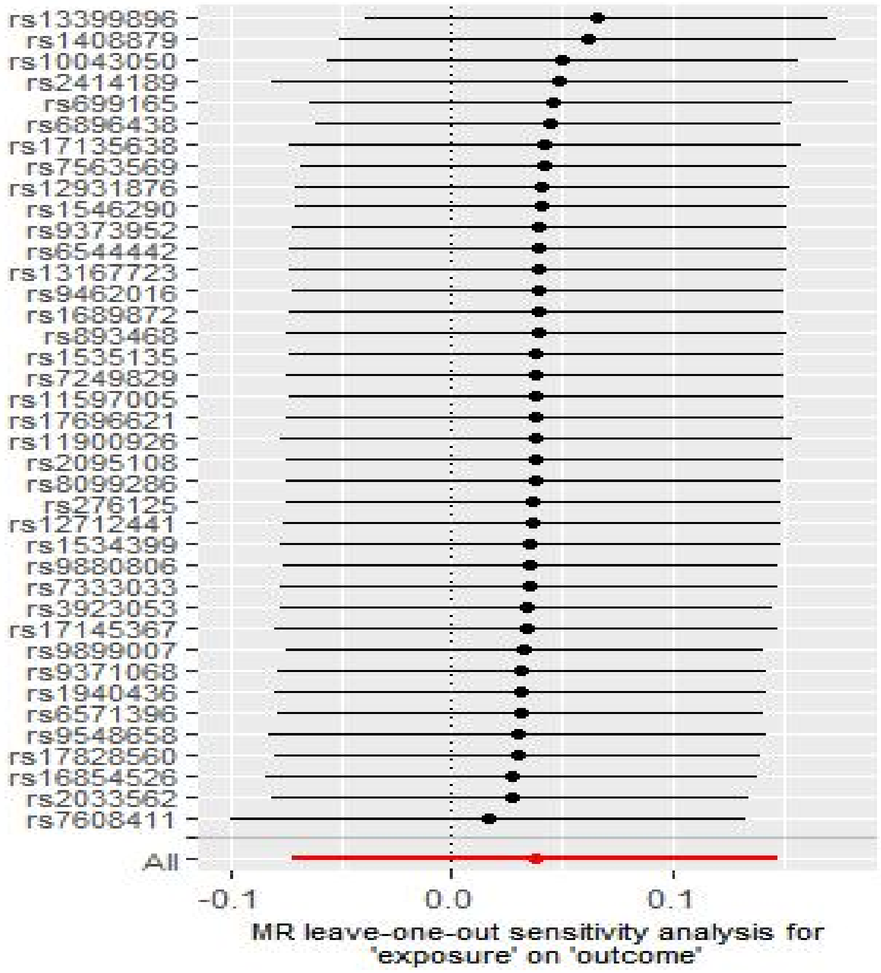

Figure 60: Funnel plots to visualize overall heterogeneity of Mendelian randomization (MR)

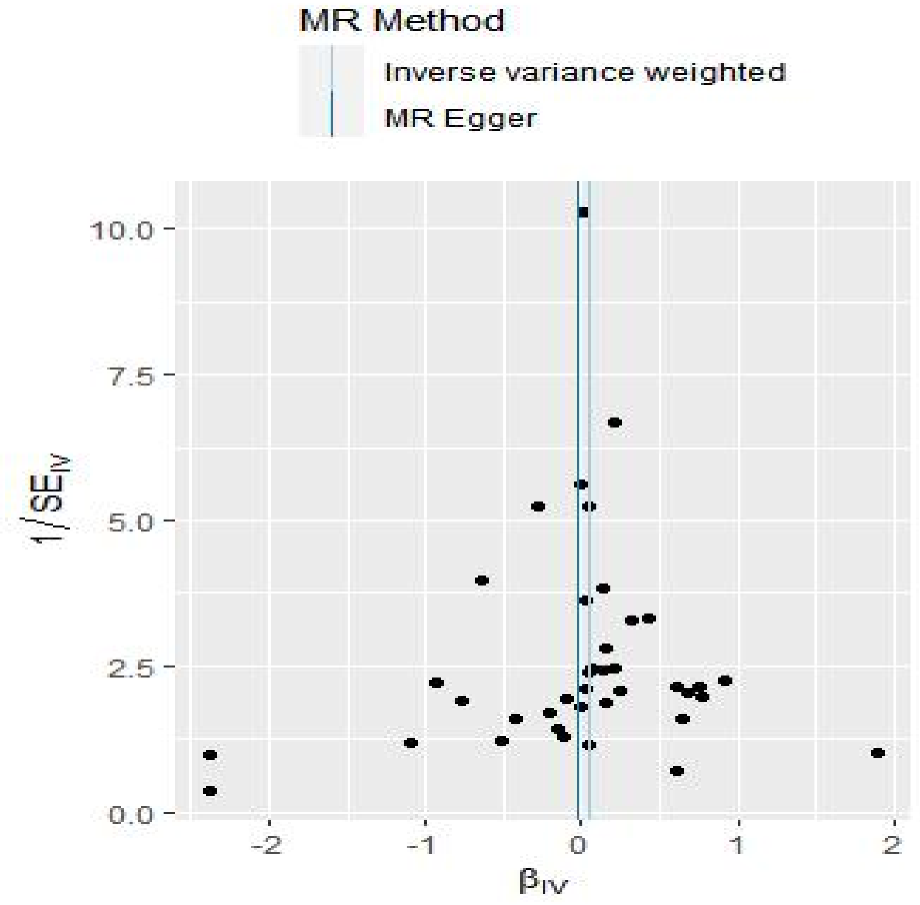

Figure 61: Leave-one-out plot to visualize causal effect of phenylalanine on the risk of hypertrophic cardiomyopathy when leaving one SNP out.

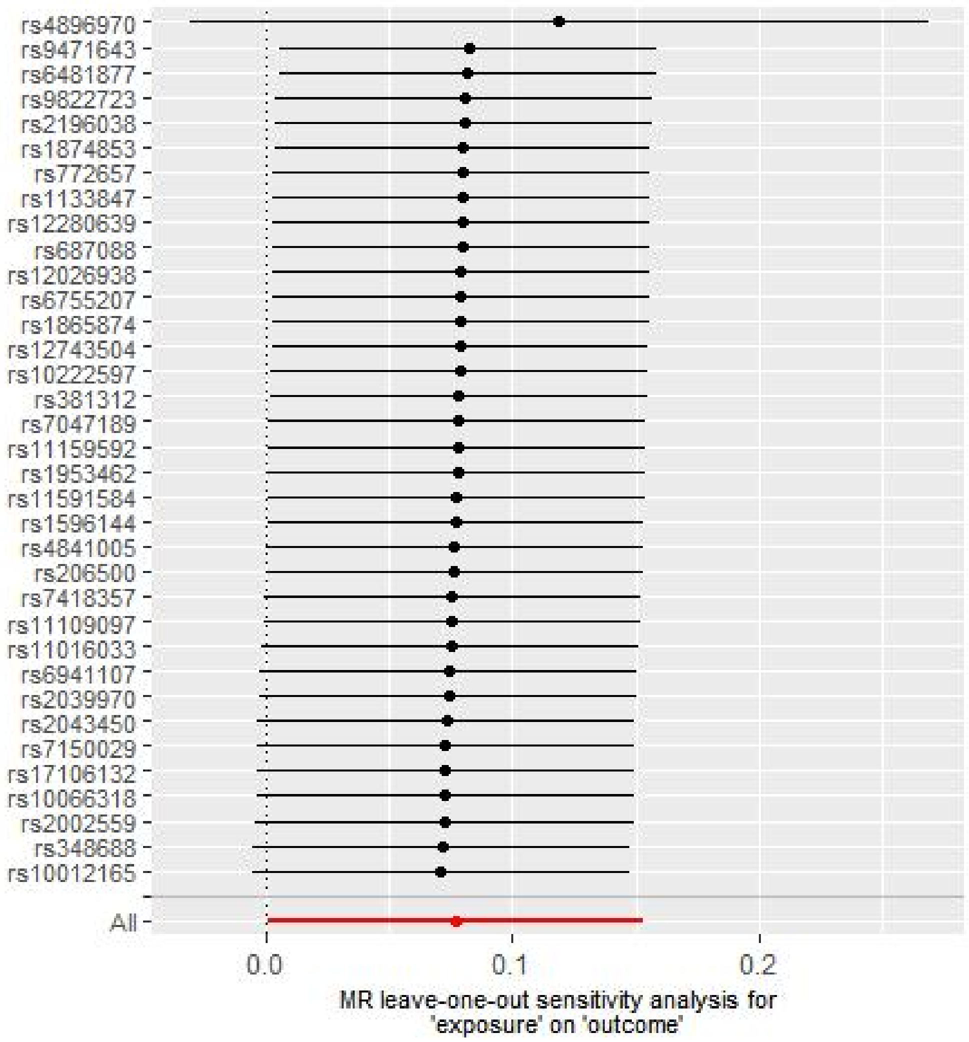

Figure 62: Funnel plots to visualize overall heterogeneity of Mendelian randomization (MR)

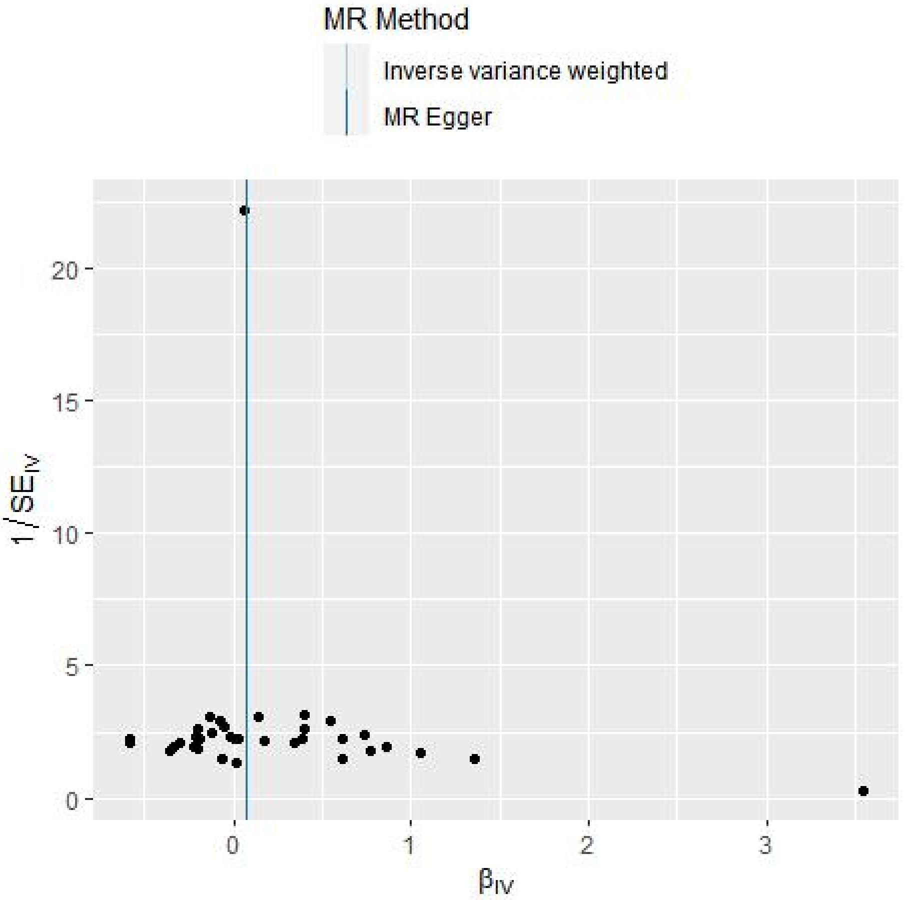

Figure 63: Leave-one-out plot to visualize causal effect of serotonin on the risk of hypertrophic cardiomyopathy when leaving one SNP out.

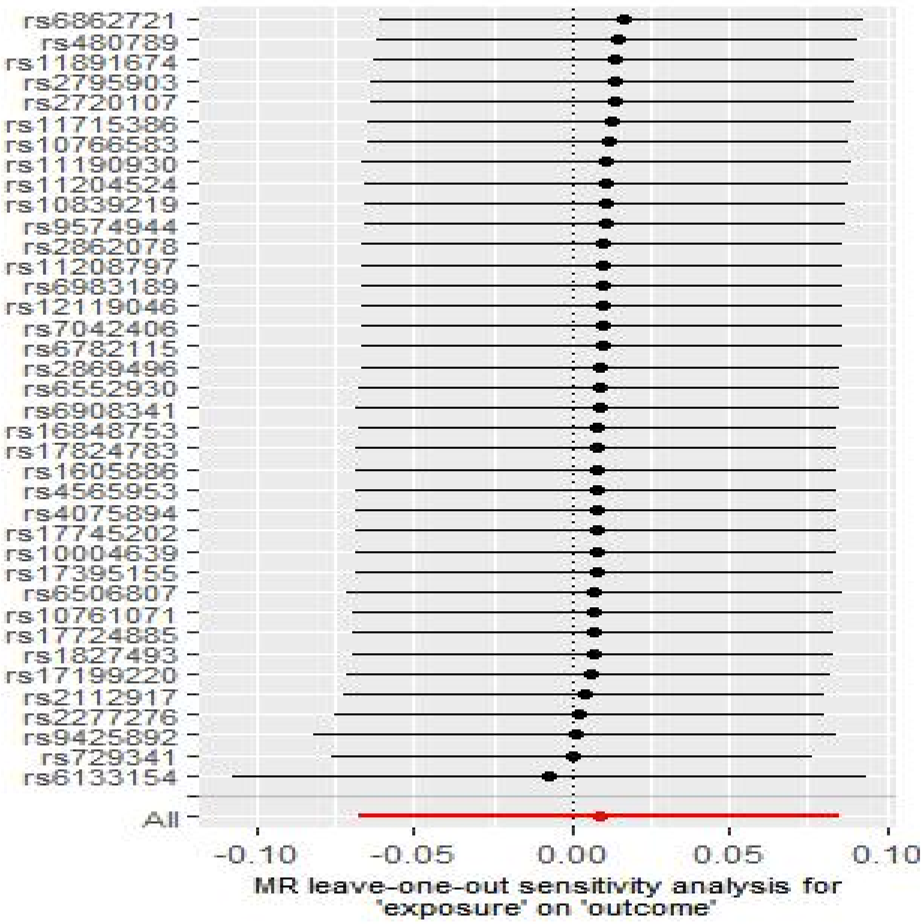

Figure 64: Funnel plots to visualize overall heterogeneity of Mendelian randomization (MR)

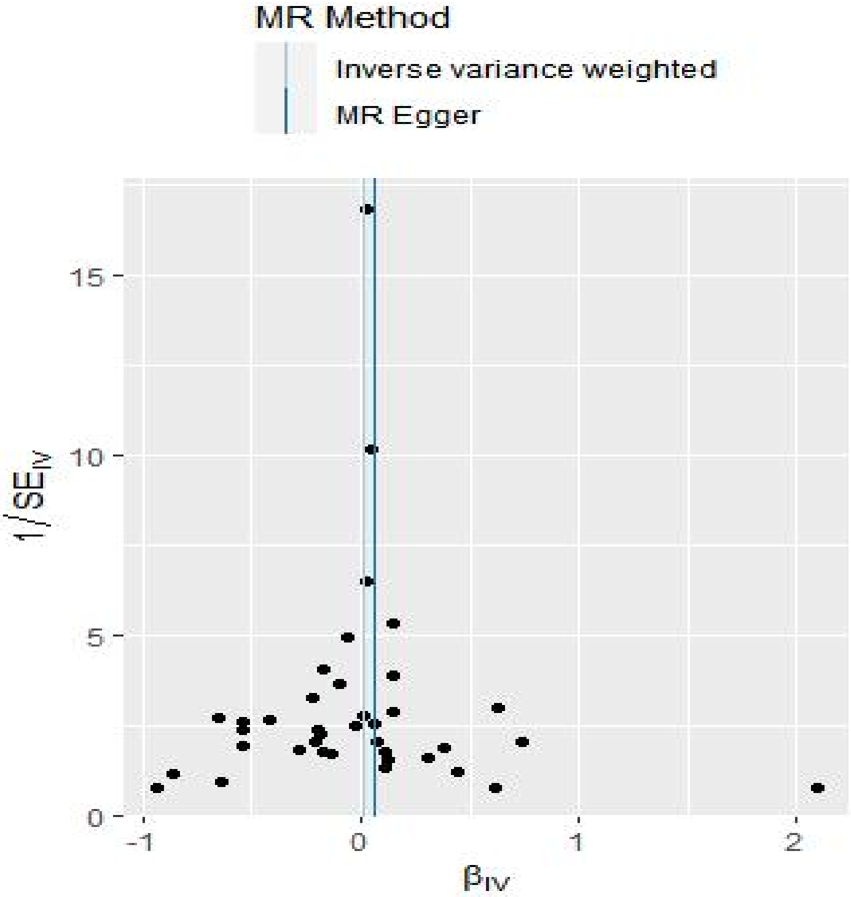

Figure 65: Leave-one-out plot to visualize causal effect of trimethylamine_N_oxide on the risk of hypertrophic cardiomyopathy when leaving one SNP out.

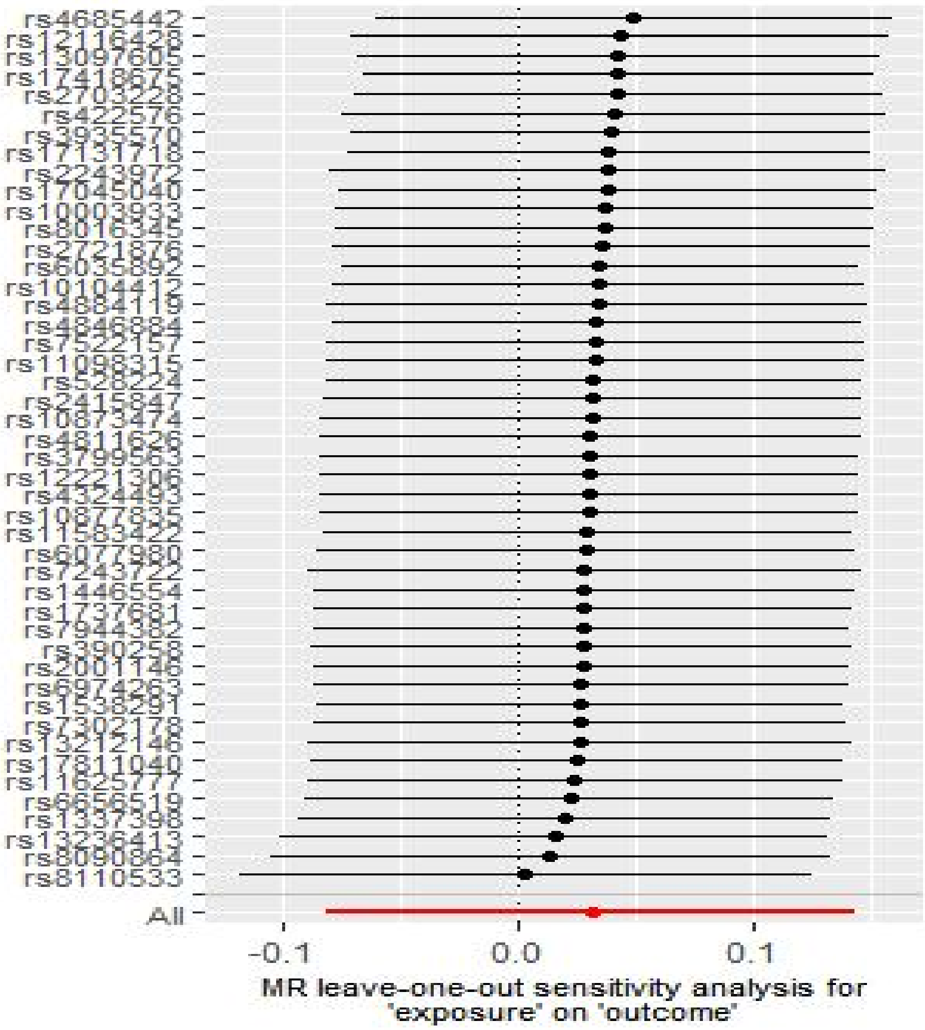

Figure 66: Funnel plots to visualize overall heterogeneity of Mendelian randomization (MR)

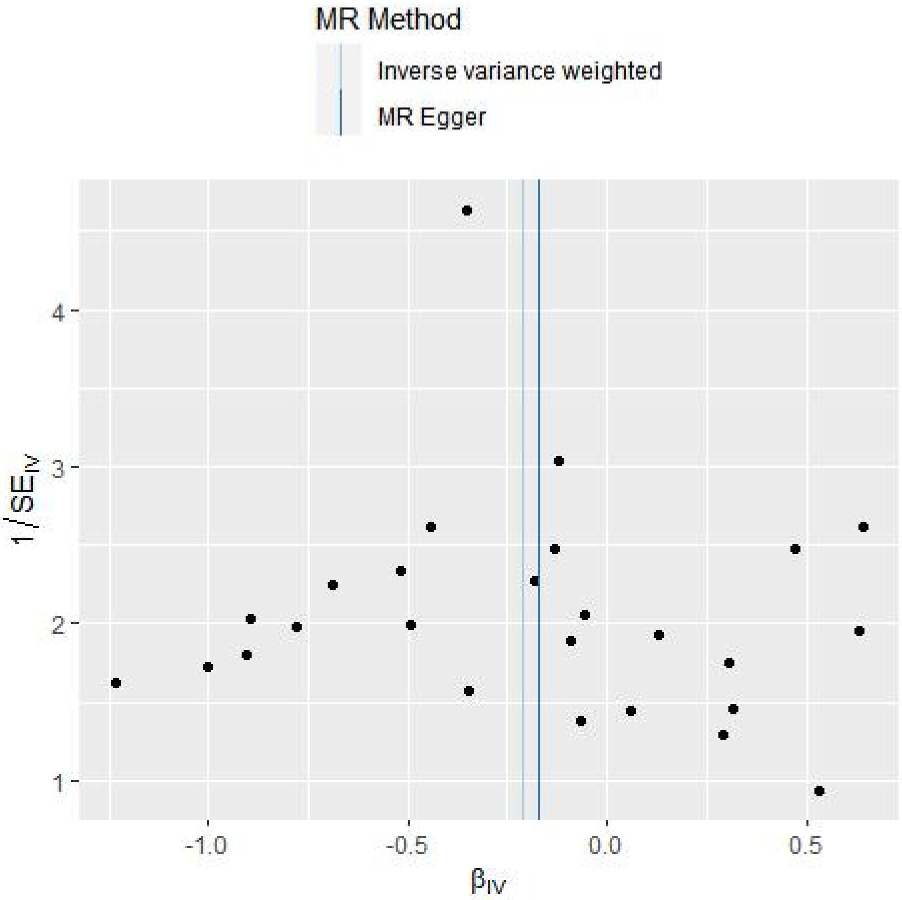

Figure 67: Leave-one-out plot to visualize causal effect of tryptophan on the risk of hypertrophic cardiomyopathy when leaving one SNP out.

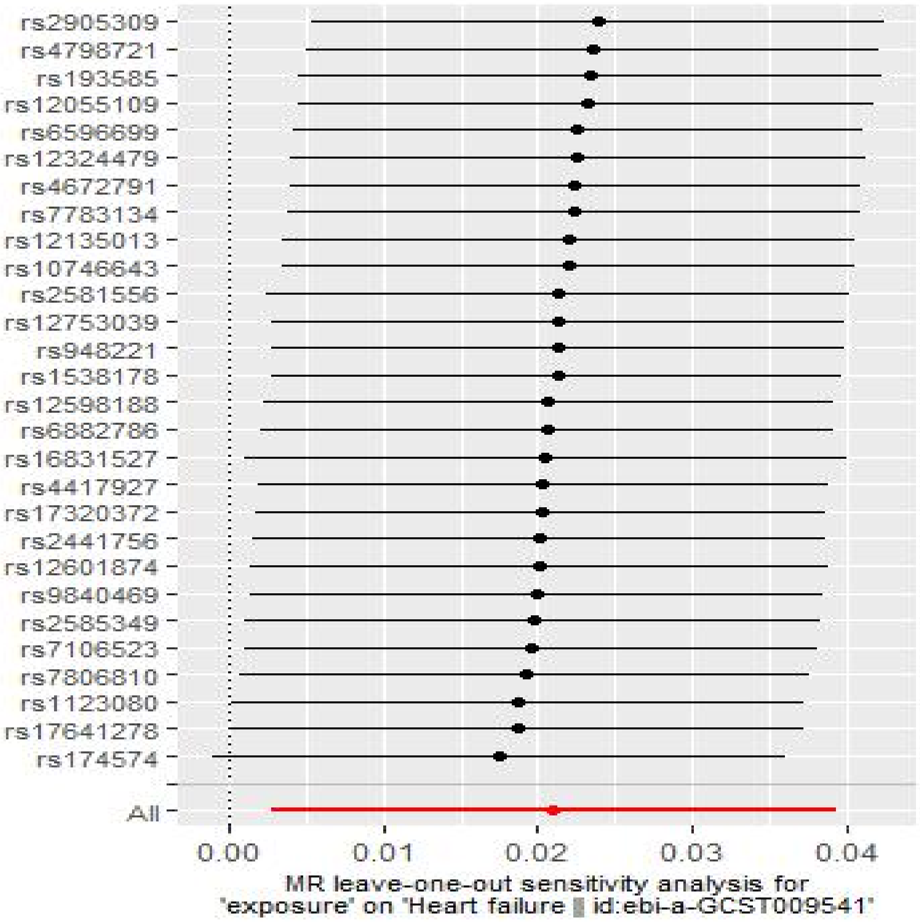

Figure 68: Funnel plots to visualize overall heterogeneity of Mendelian randomization (MR)

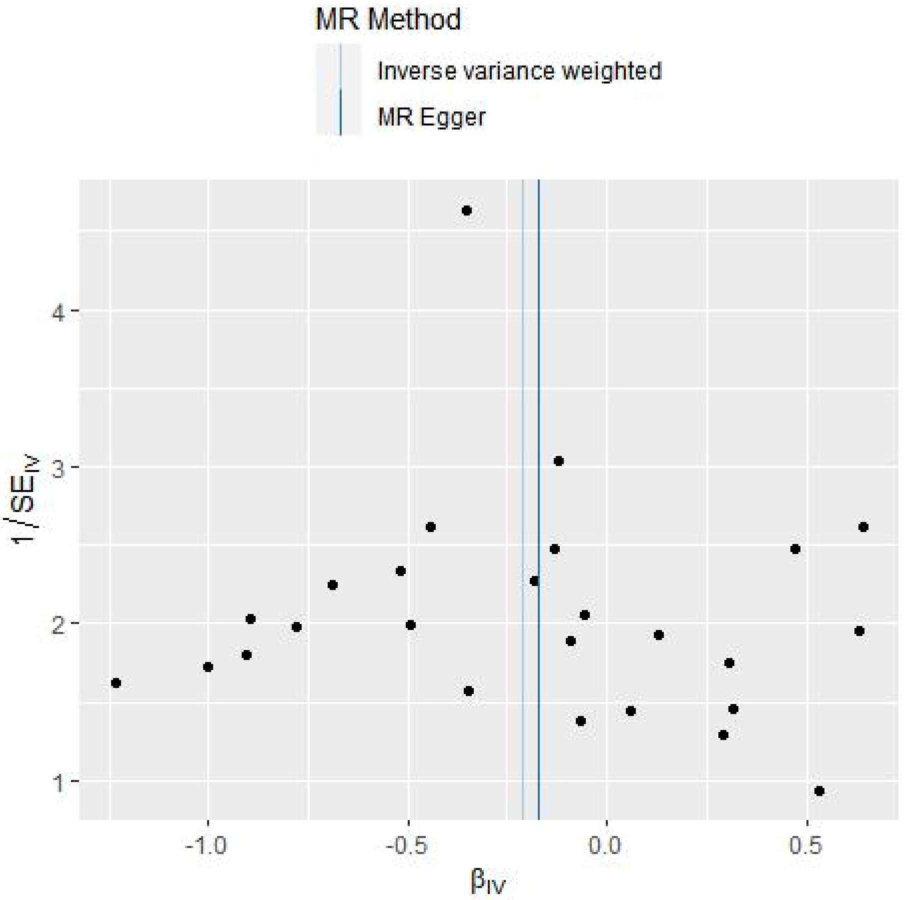

Figure 69: Leave-one-out plot to visualize causal effect of tyrosine on the risk of hypertrophic cardiomyopathy when leaving one SNP out.

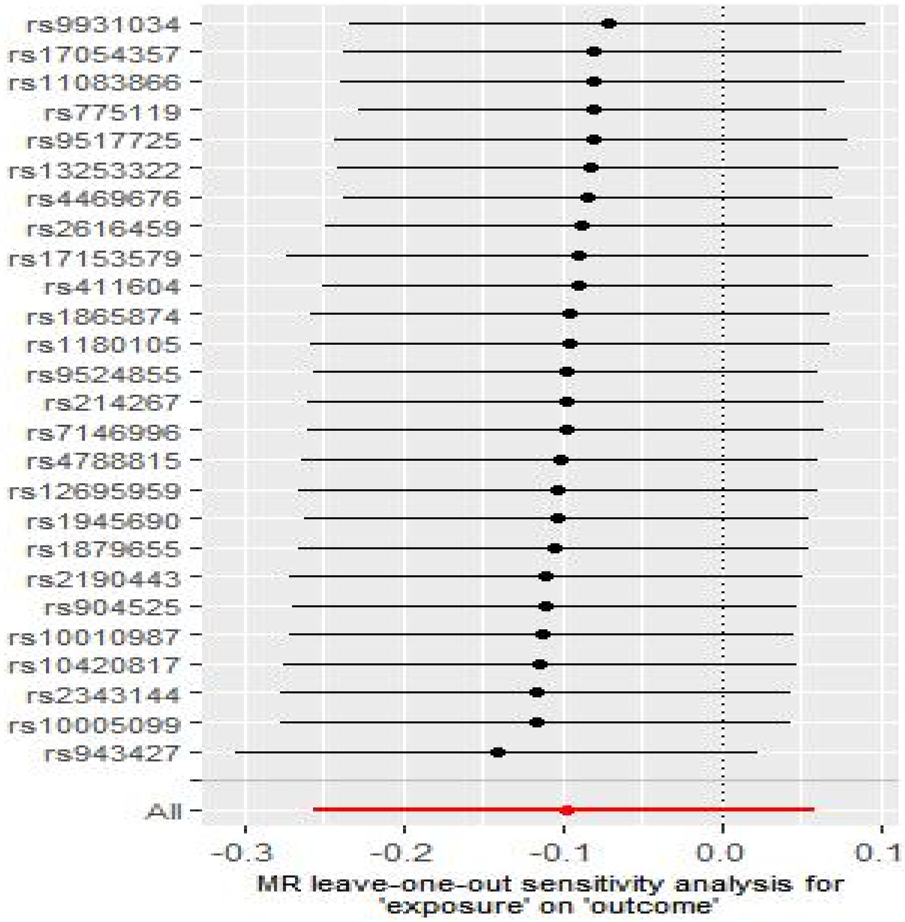

Figure 70: Funnel plots to visualize overall heterogeneity of Mendelian randomization (MR)

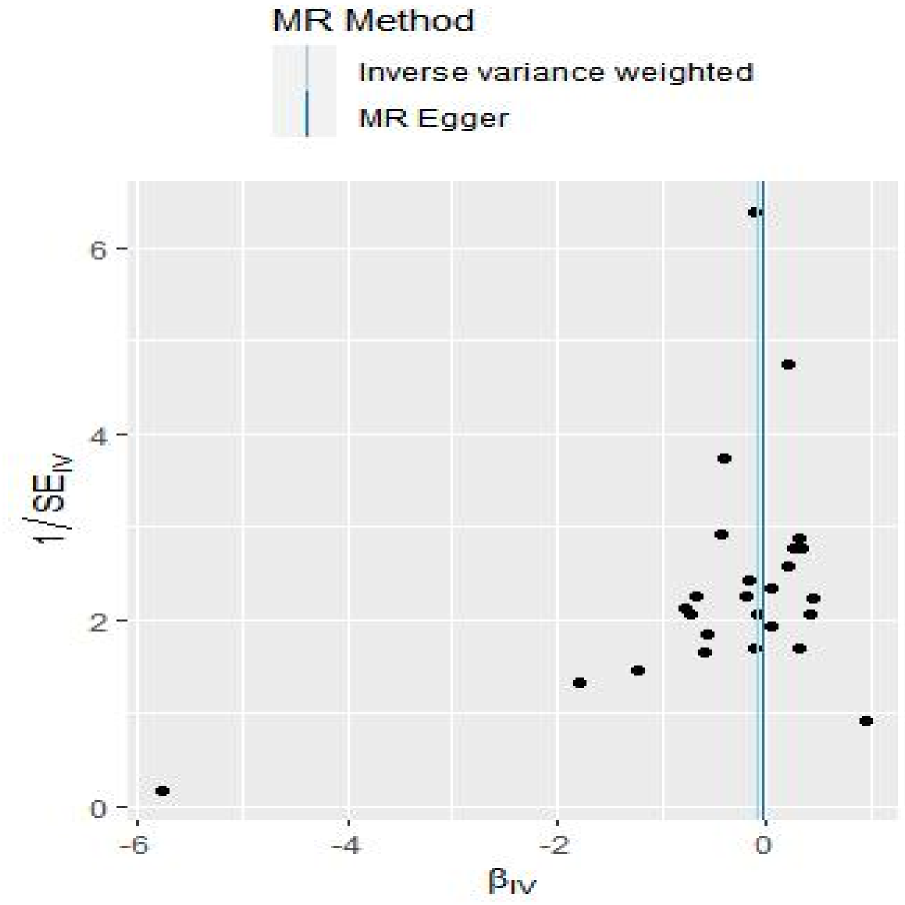

Figure 71: Leave-one-out plot to visualize causal effect of propionic acid on the risk of hypertrophic cardiomyopathy when leaving one SNP out.

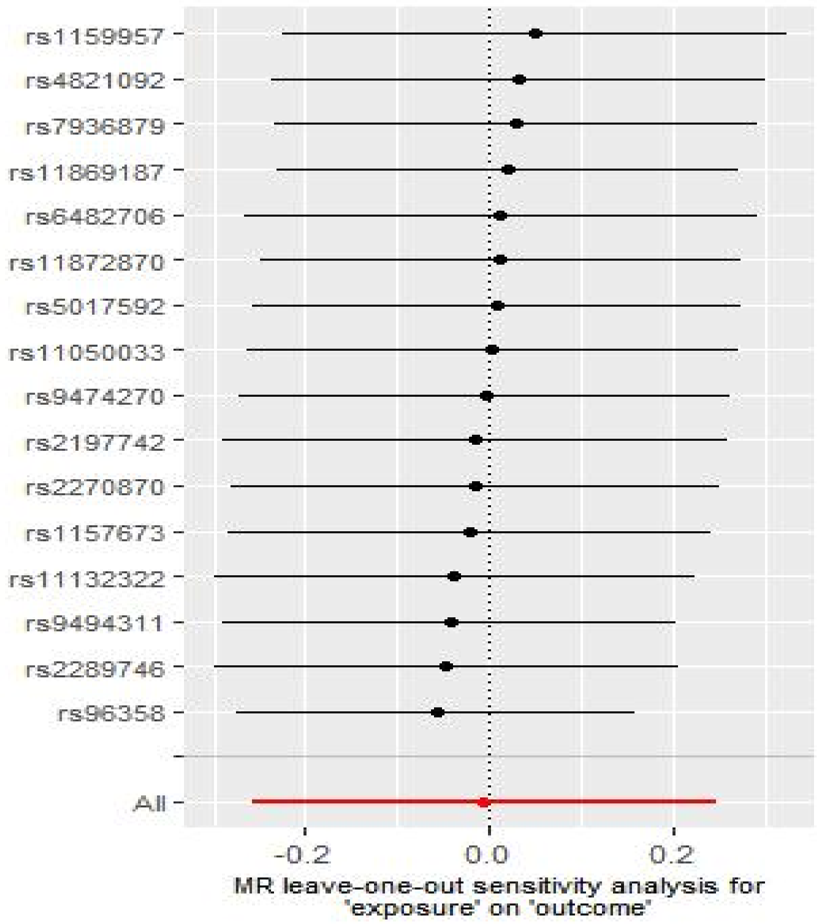

Figure 72: Funnel plots to visualize overall heterogeneity of Mendelian randomization (MR)

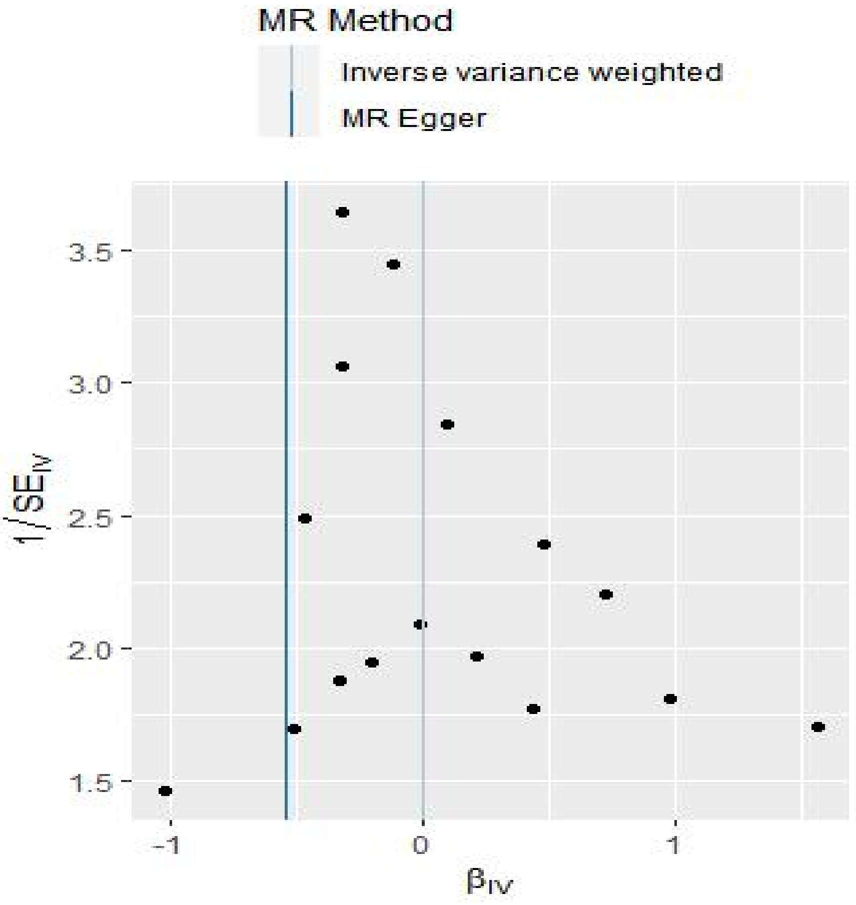

Figure 73: Leave-one-out plot to visualize causal effect of beta_hydroxybutyric acid on the risk of coronary heart disease when leaving one SNP out.

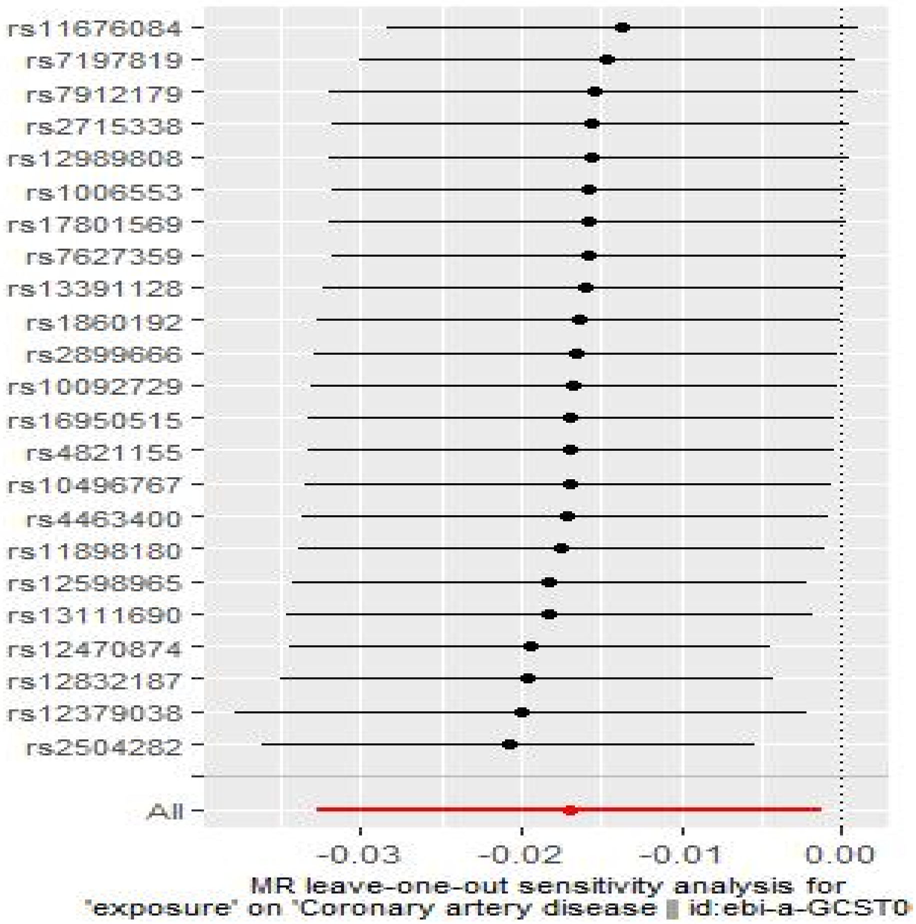

Figure 74: Funnel plots to visualize overall heterogeneity of Mendelian randomization (MR)

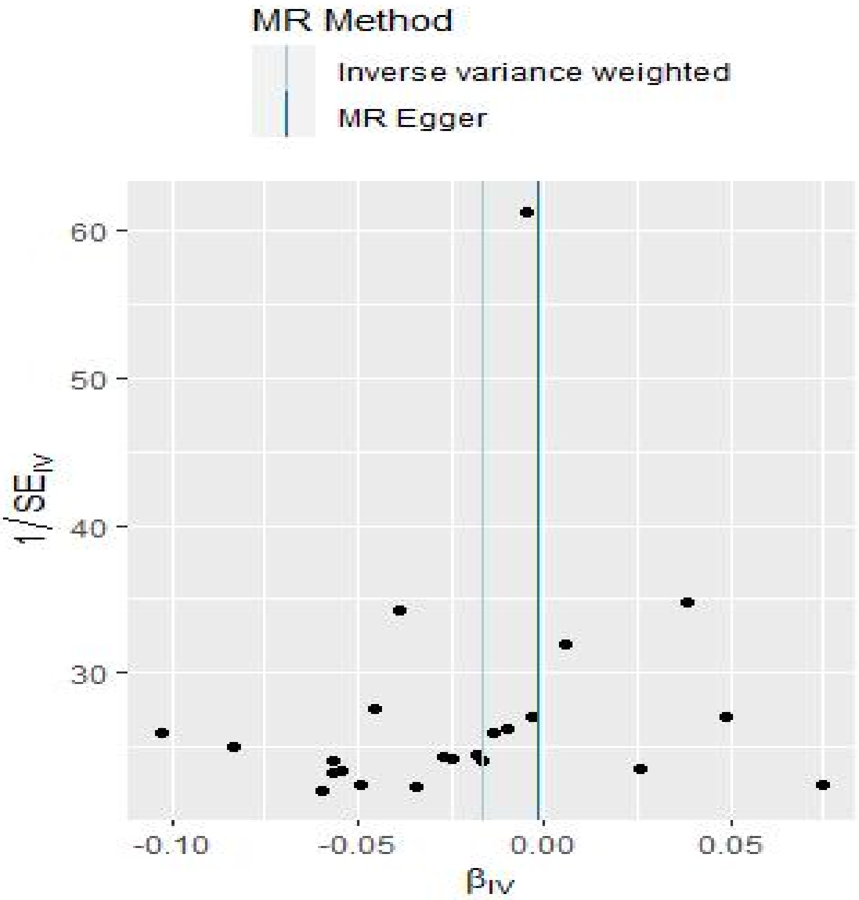

Figure 75: Leave-one-out plot to visualize causal effect of betaine on the risk of coronary heart disease when leaving one SNP out.

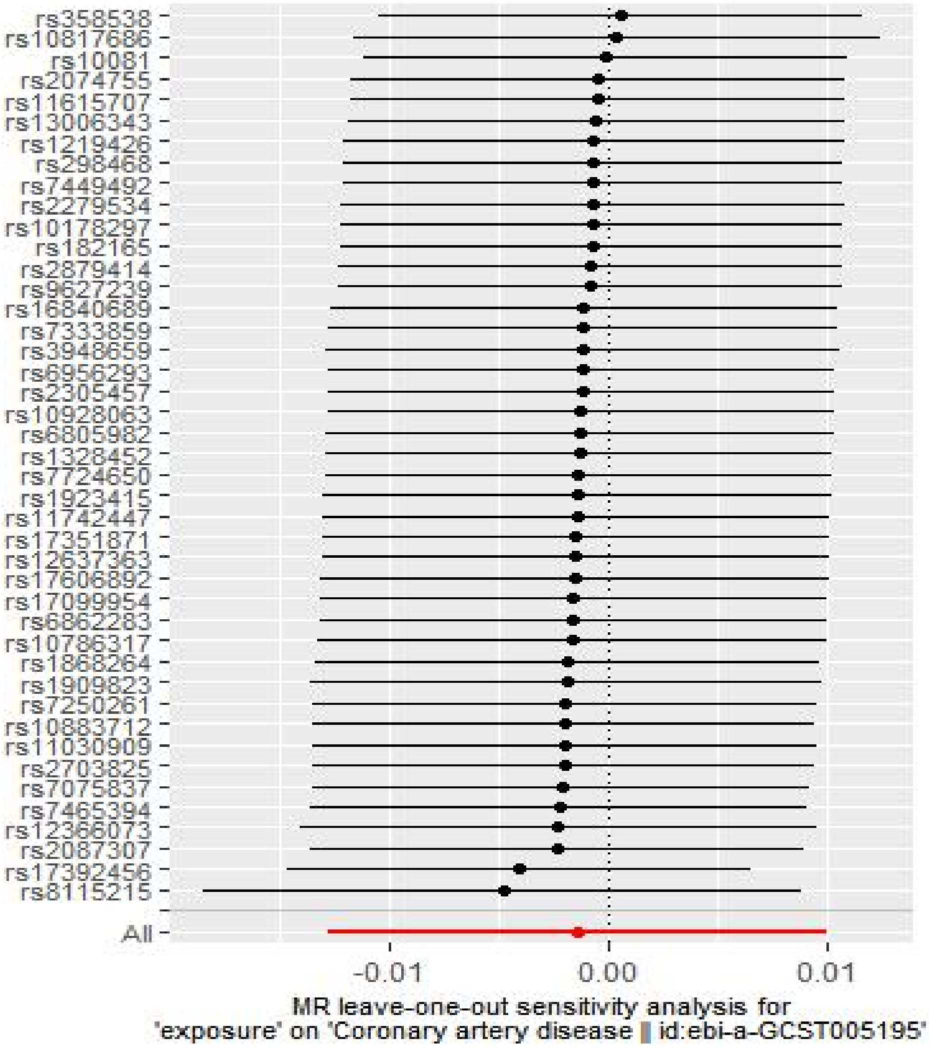

Figure 76: Funnel plots to visualize overall heterogeneity of Mendelian randomization (MR)

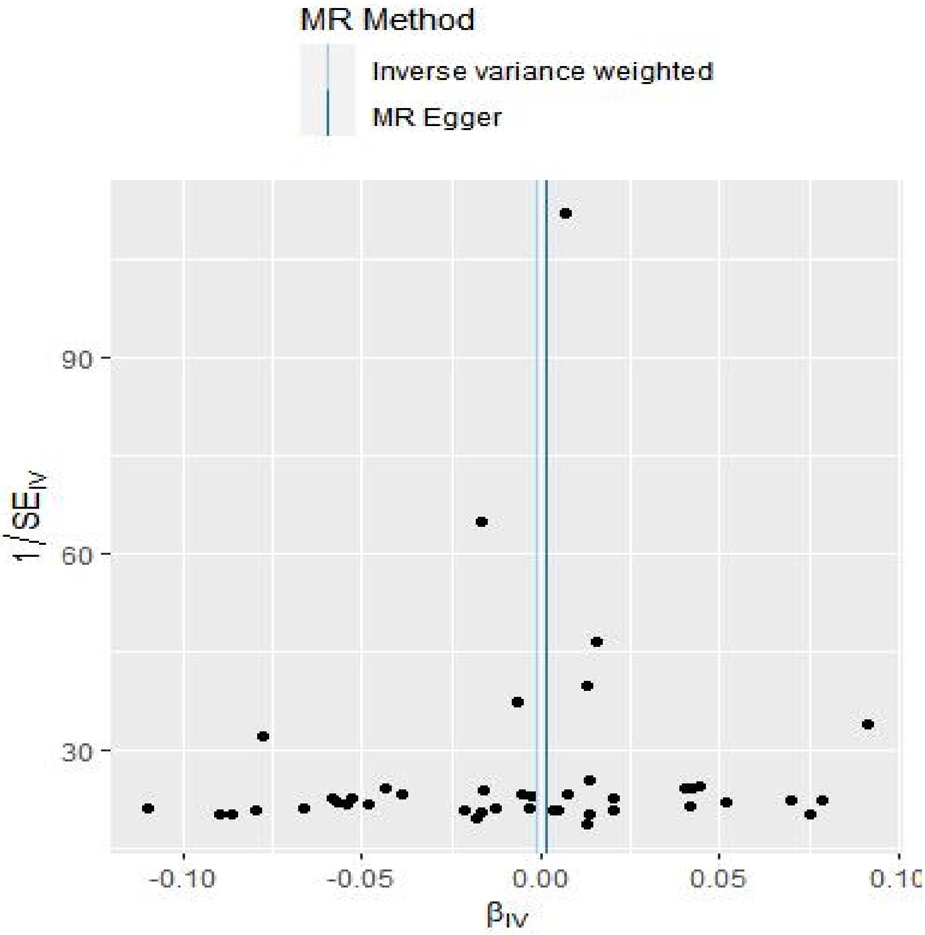

Figure 77: Leave-one-out plot to visualize causal effect of carnitine on the risk of coronary heart disease when leaving one SNP out.

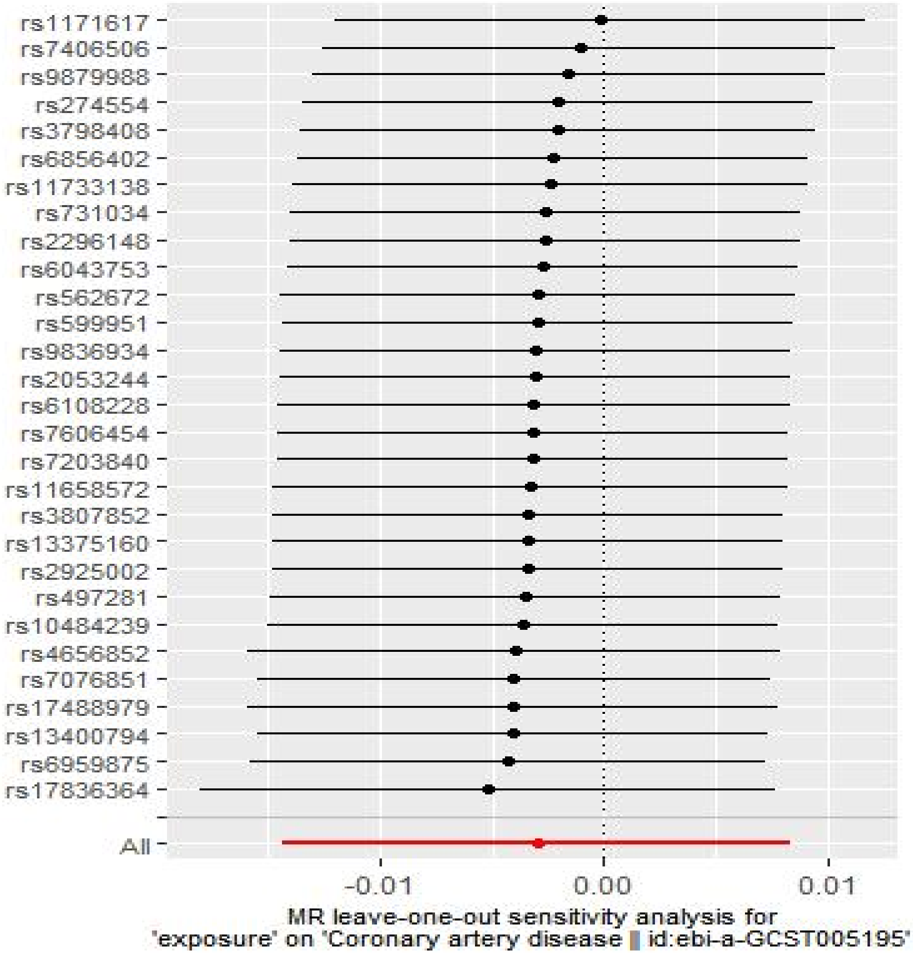

Figure 78: Funnel plots to visualize overall heterogeneity of Mendelian randomization (MR)

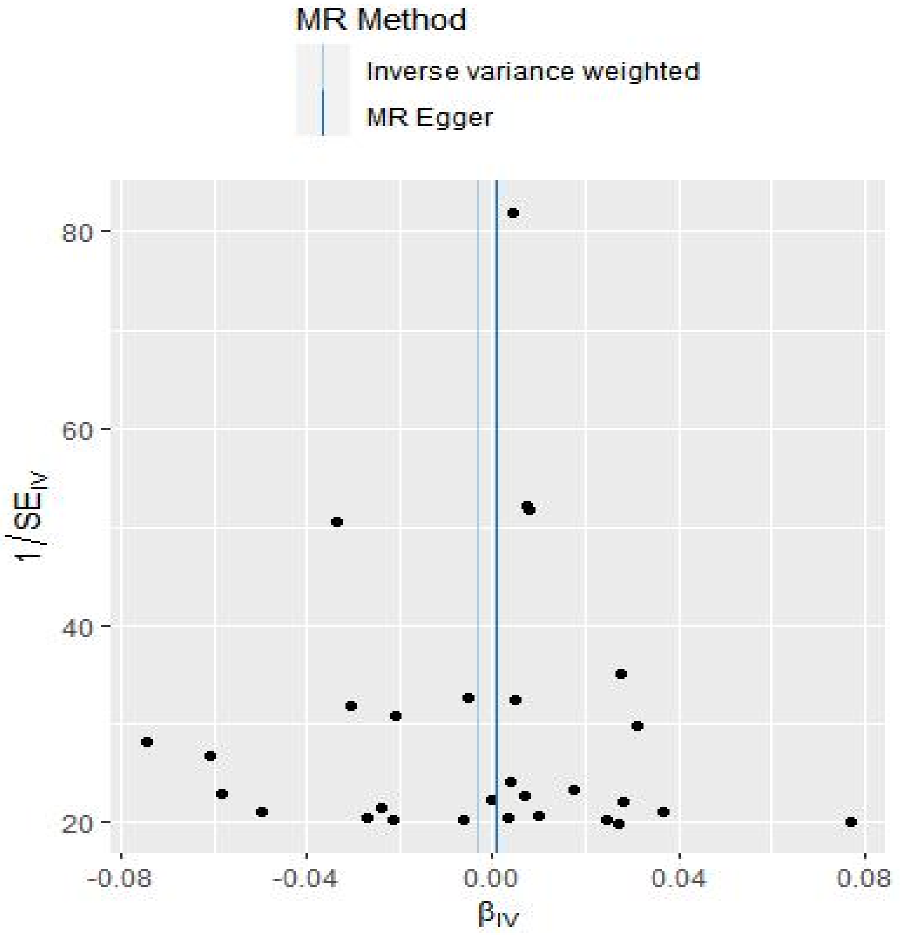

Figure 79: Leave-one-out plot to visualize causal effect of choline on the risk of coronary heart disease when leaving one SNP out.

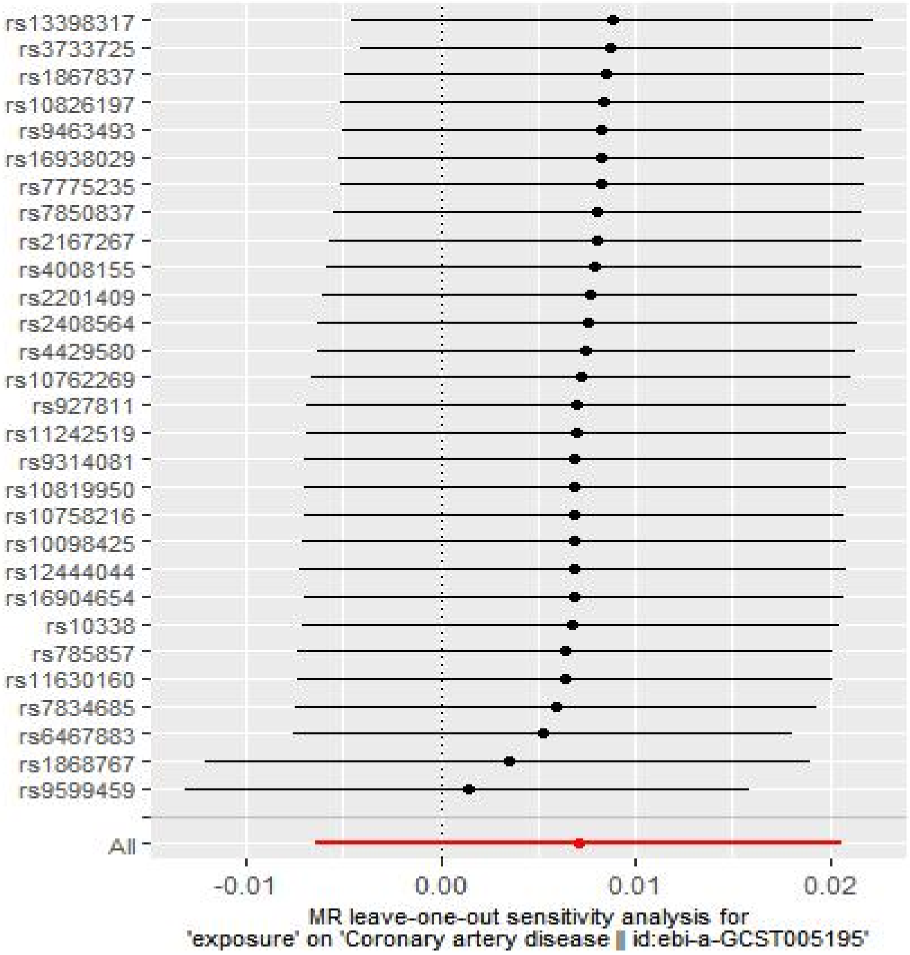

Figure 80: Funnel plots to visualize overall heterogeneity of Mendelian randomization (MR)

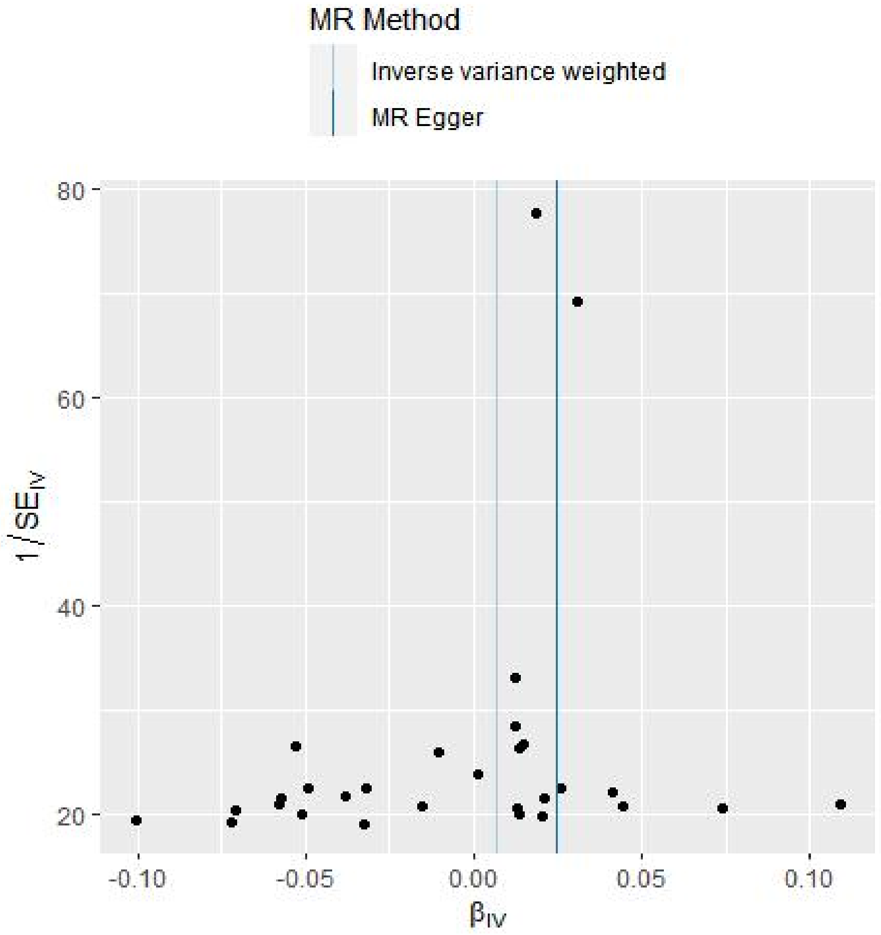

Figure 81: Leave-one-out plot to visualize causal effect of glutamate on the risk of coronary heart disease when leaving one SNP out.

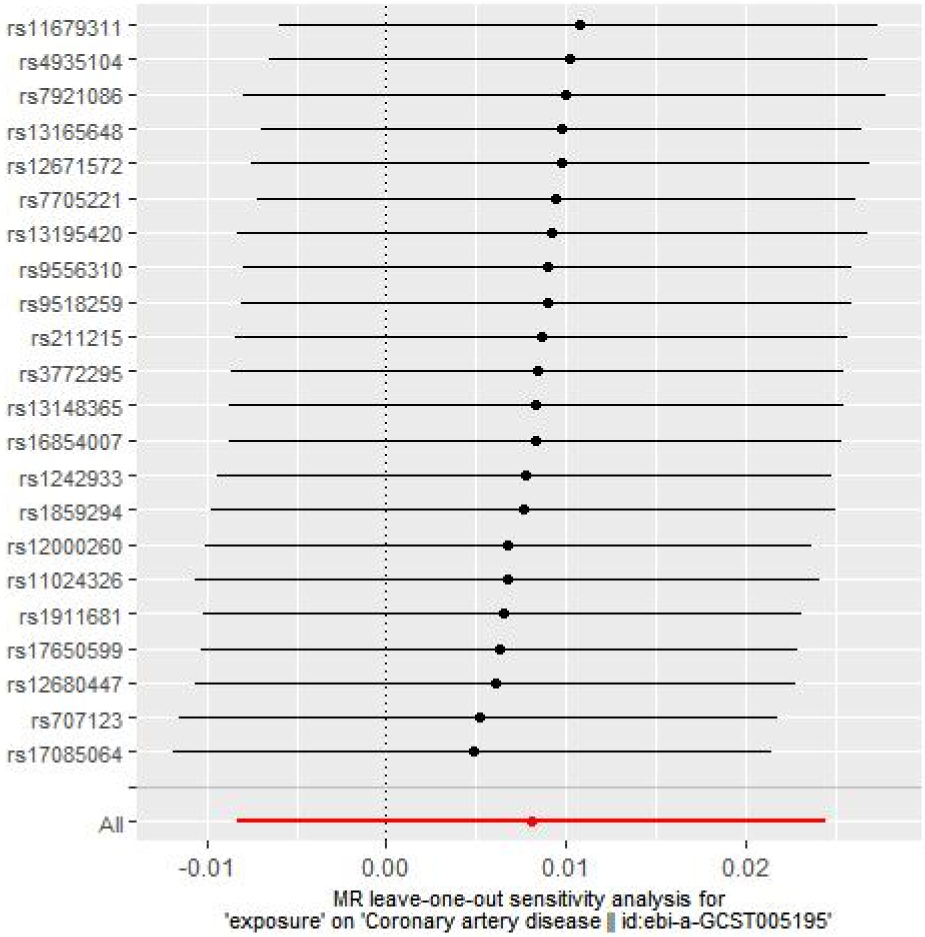

Figure 82: Funnel plots to visualize overall heterogeneity of Mendelian randomization (MR)

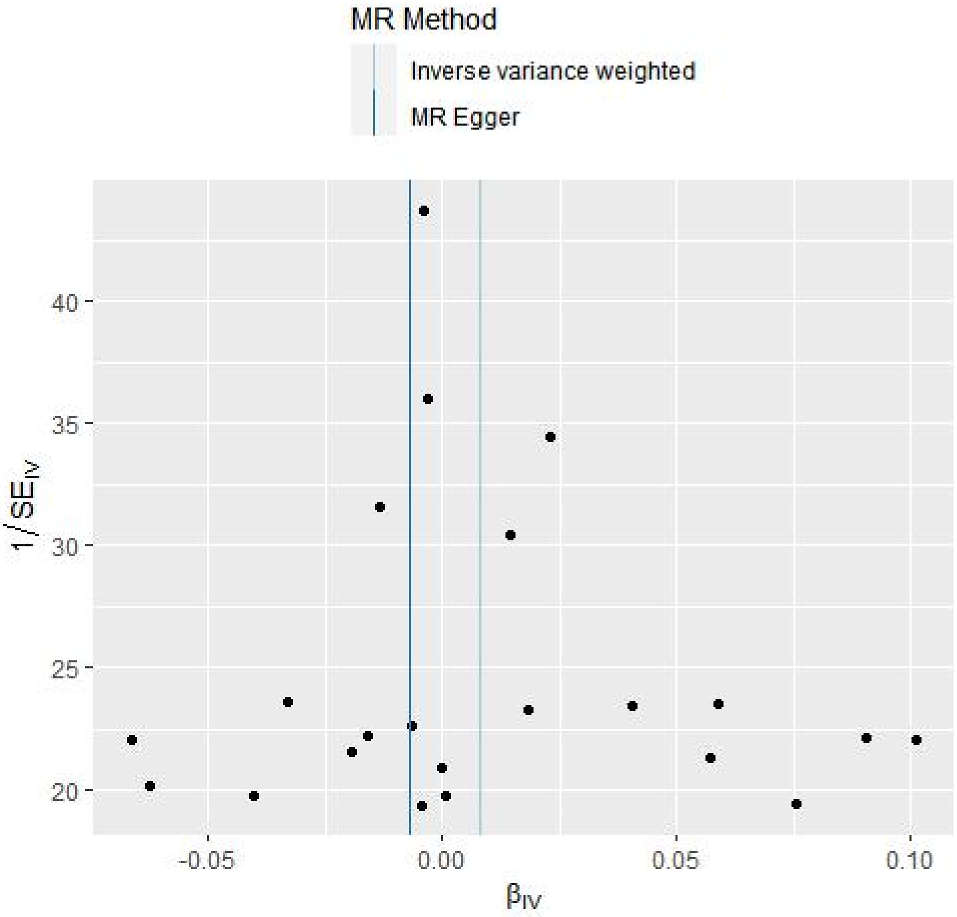

Figure 83: Leave-one-out plot to visualize causal effect of kynuremine on the risk of coronary heart disease when leaving one SNP out.

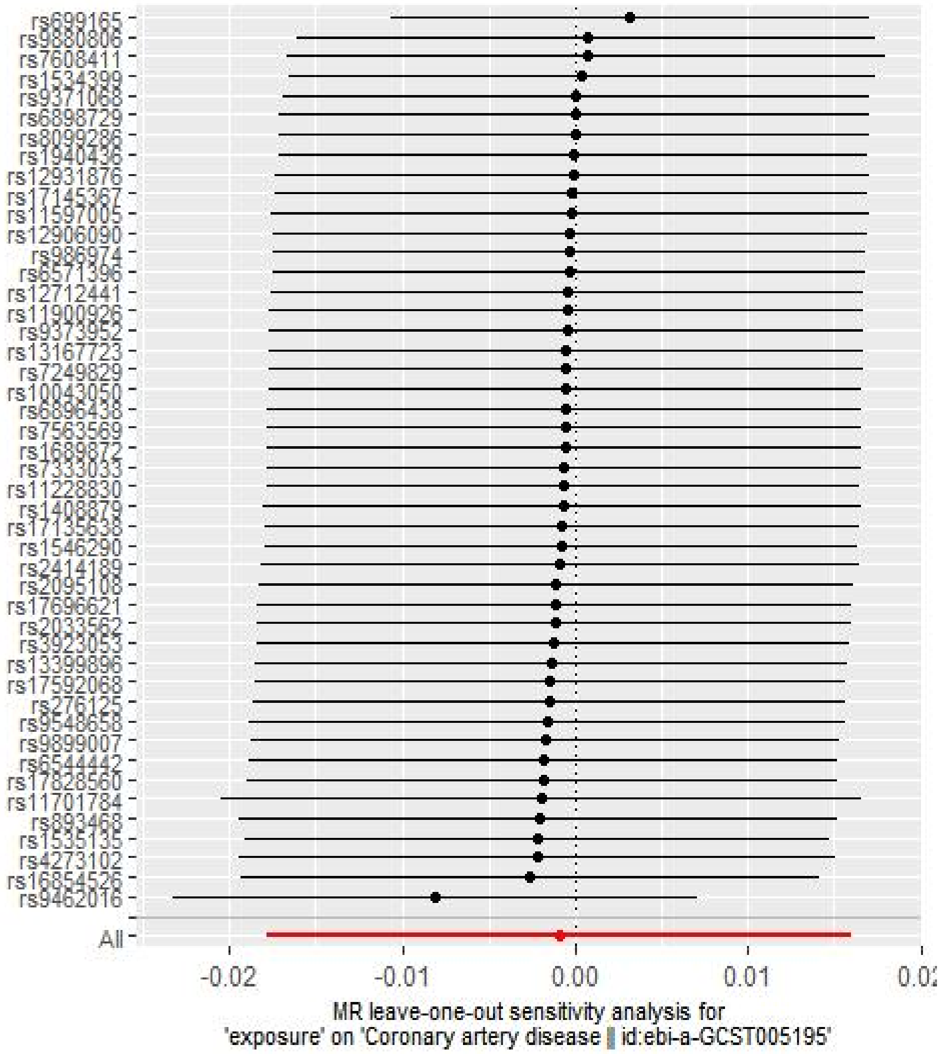

Figure 84: Funnel plots to visualize overall heterogeneity of Mendelian randomization (MR)

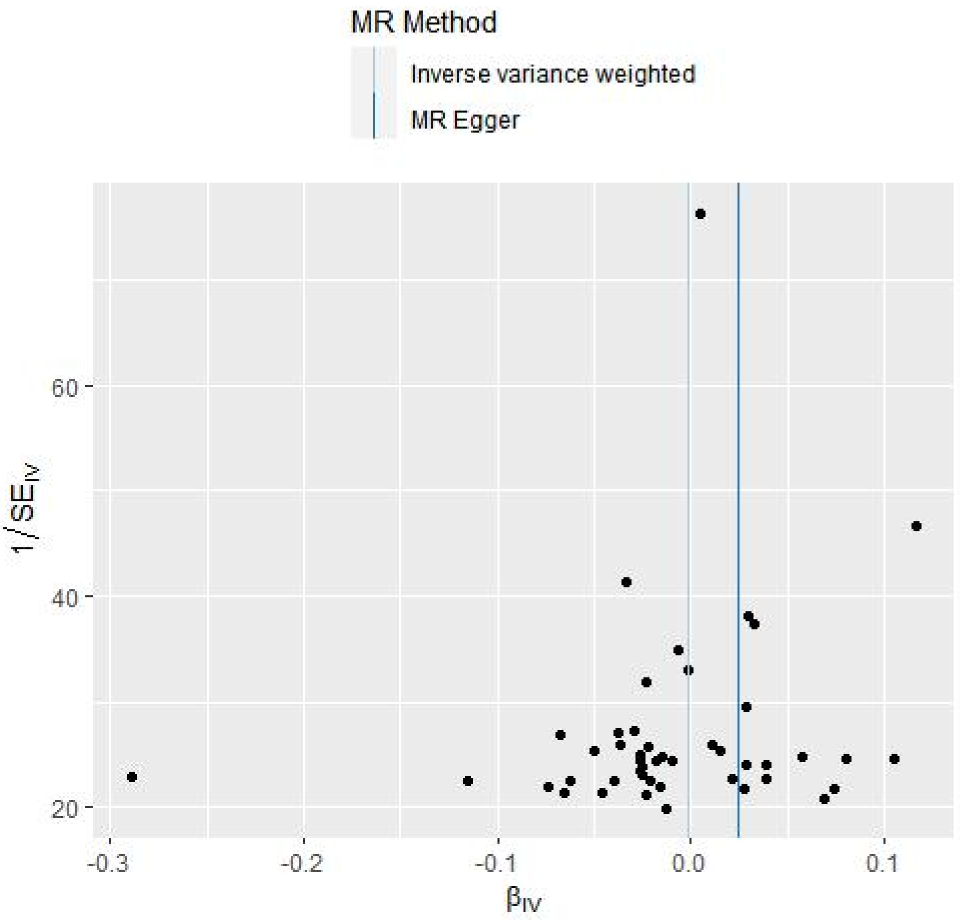

Figure 85: Leave-one-out plot to visualize causal effect of phenylalanine on the risk of coronary heart disease when leaving one SNP out.

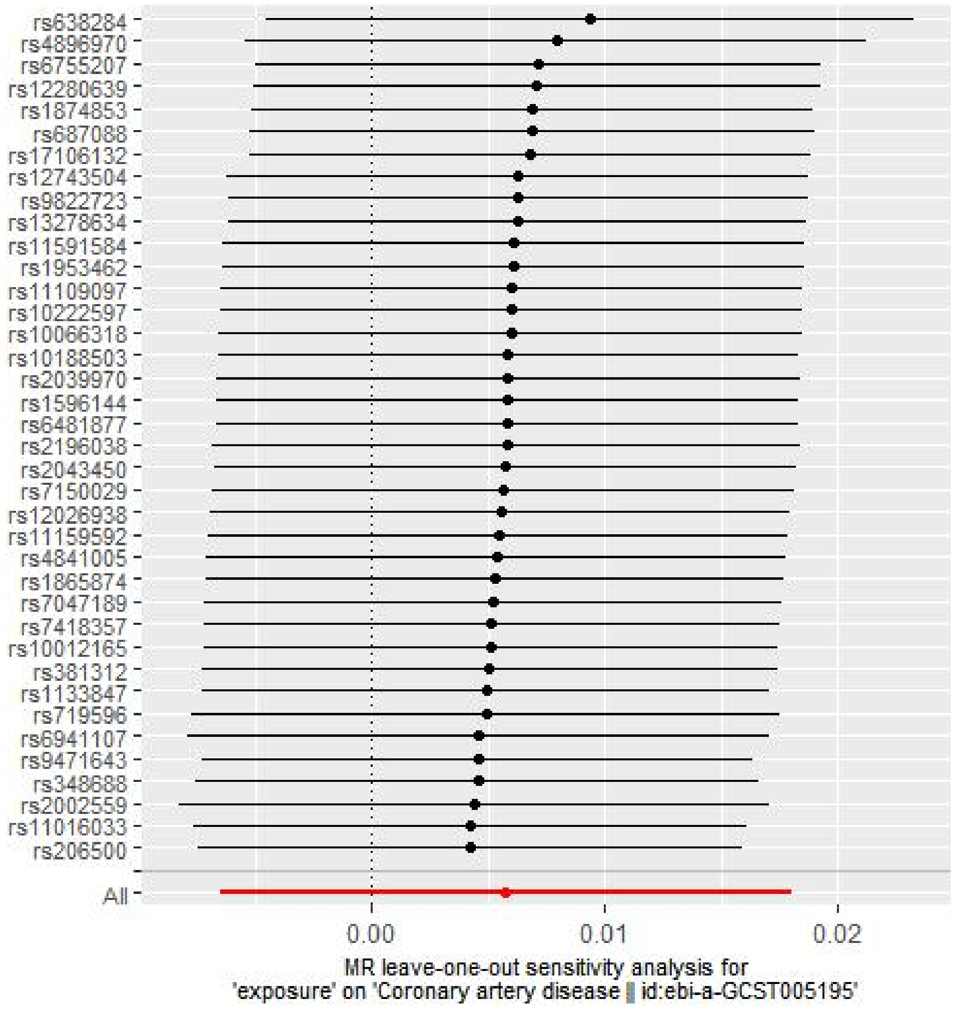

Figure 86: Funnel plots to visualize overall heterogeneity of Mendelian randomization (MR)

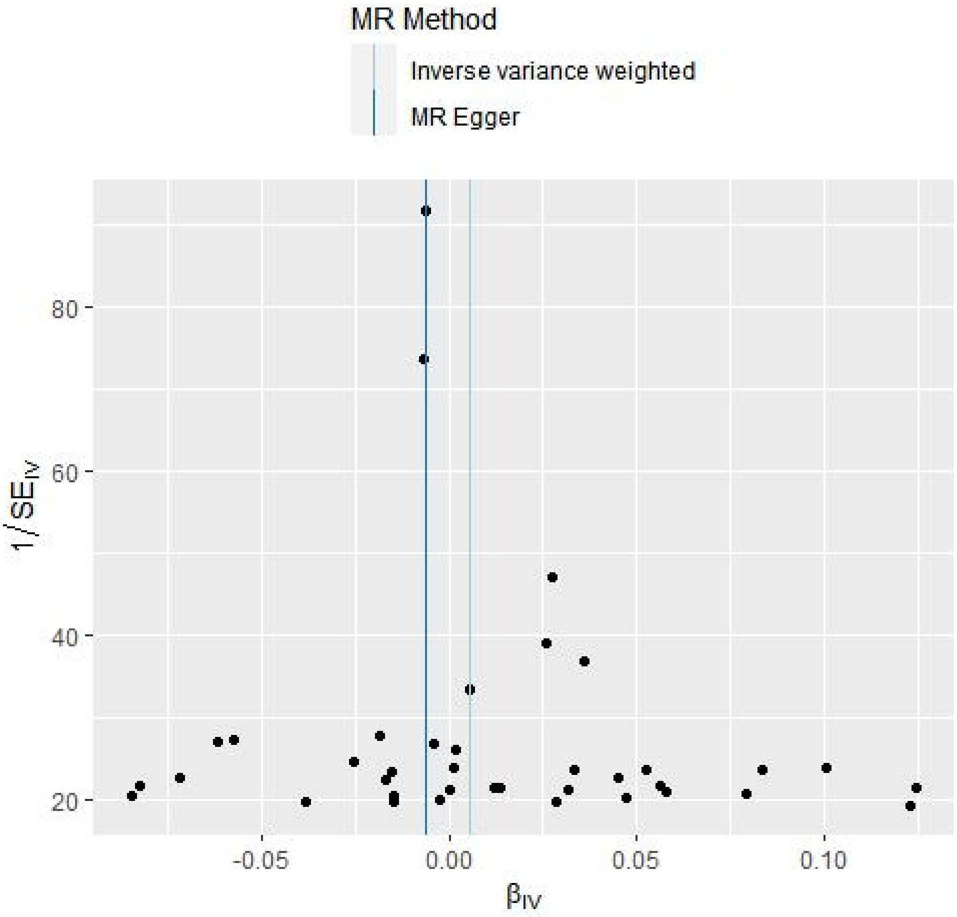

Figure 87: Leave-one-out plot to visualize causal effect of serotonin on the risk of coronary heart disease when leaving one SNP out.

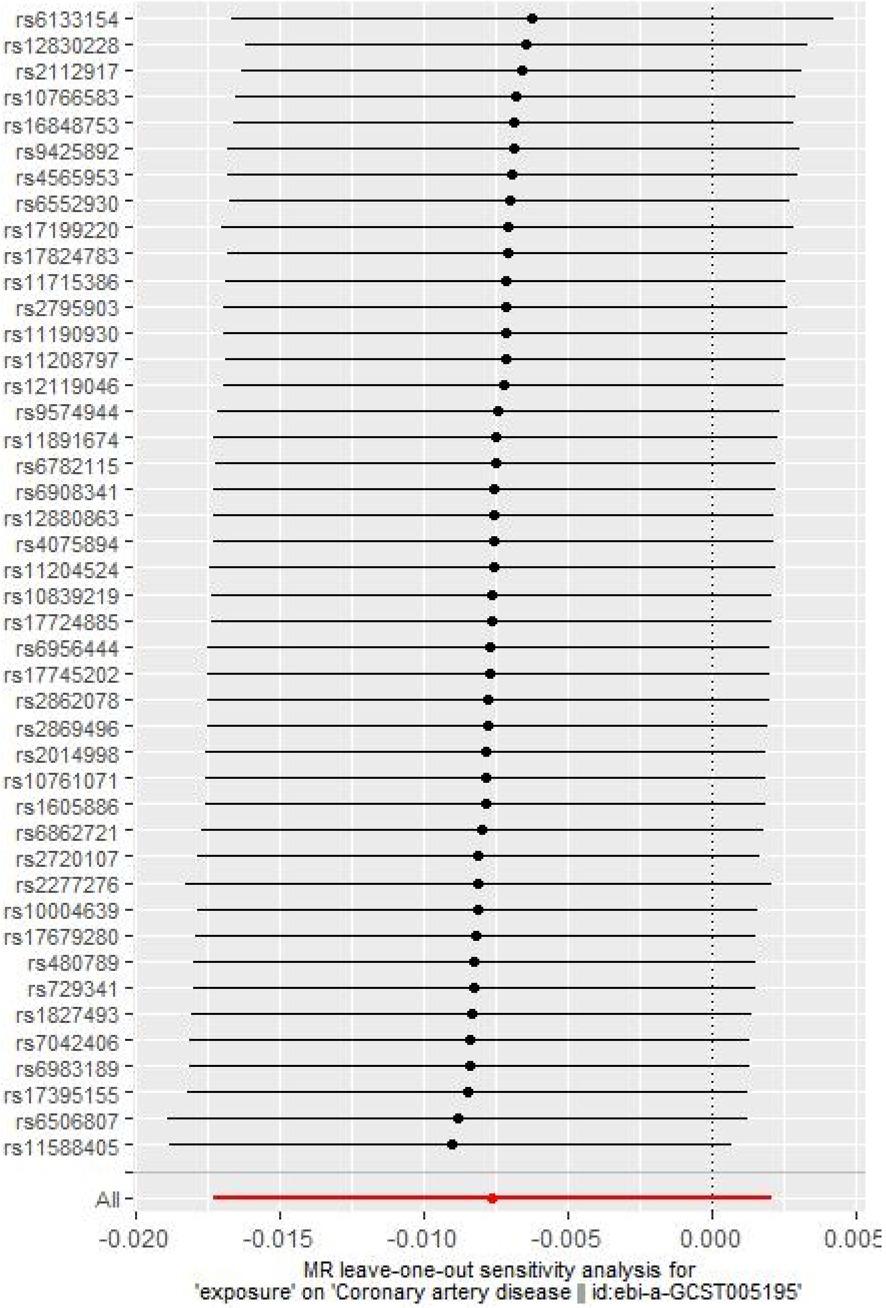

Figure 88: Funnel plots to visualize overall heterogeneity of Mendelian randomization (MR)

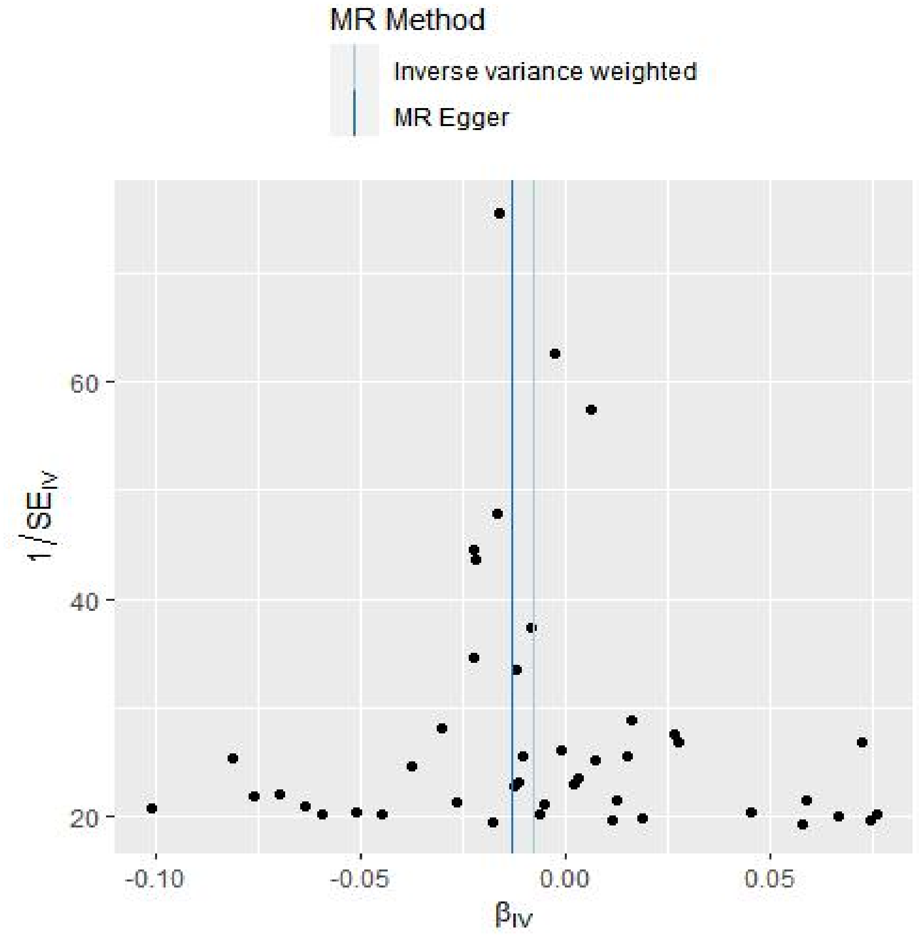

Figure 89: Leave-one-out plot to visualize causal effect of trimethylamine_N_oxide on the risk of coronary heart disease when leaving one SNP out.

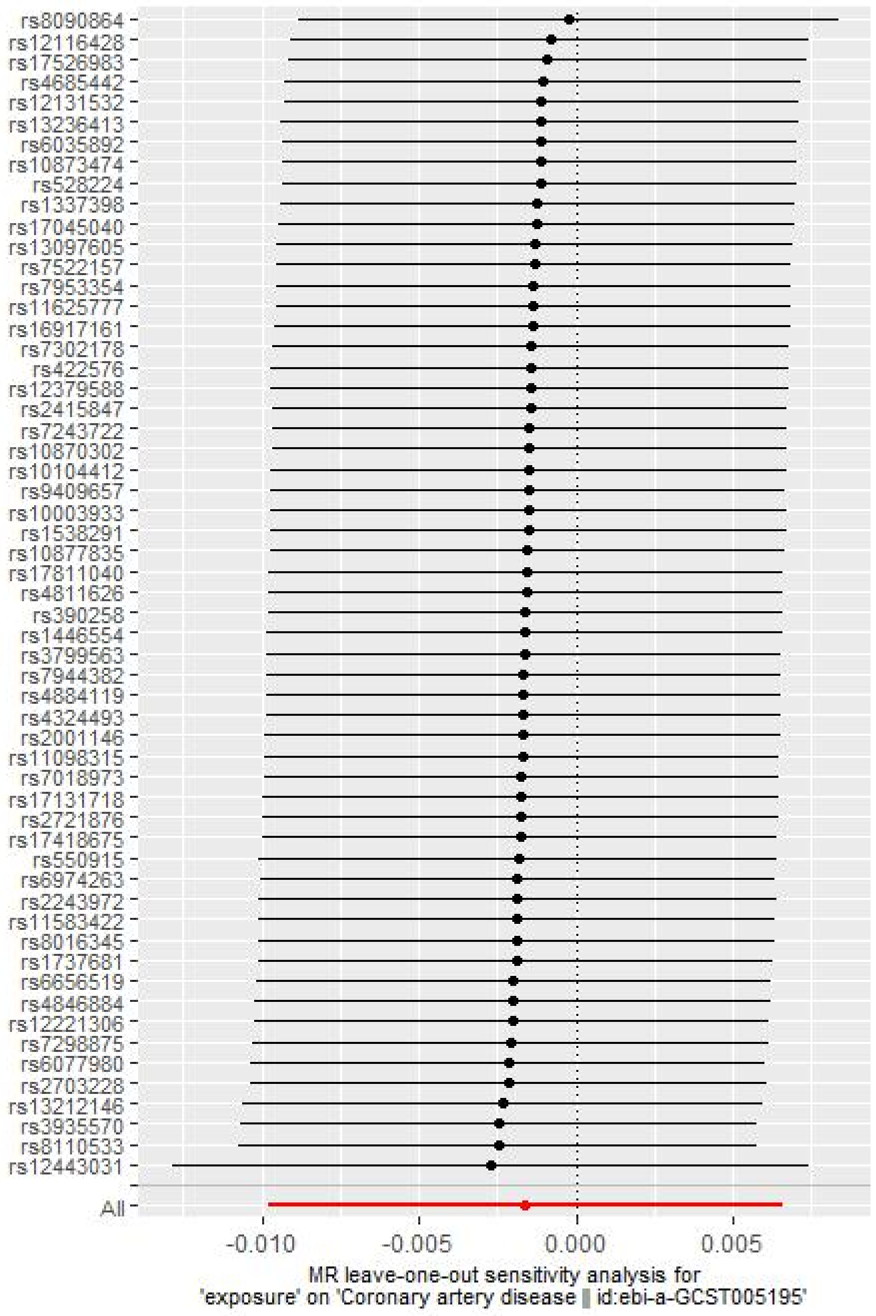

Figure 90: Funnel plots to visualize overall heterogeneity of Mendelian randomization (MR)

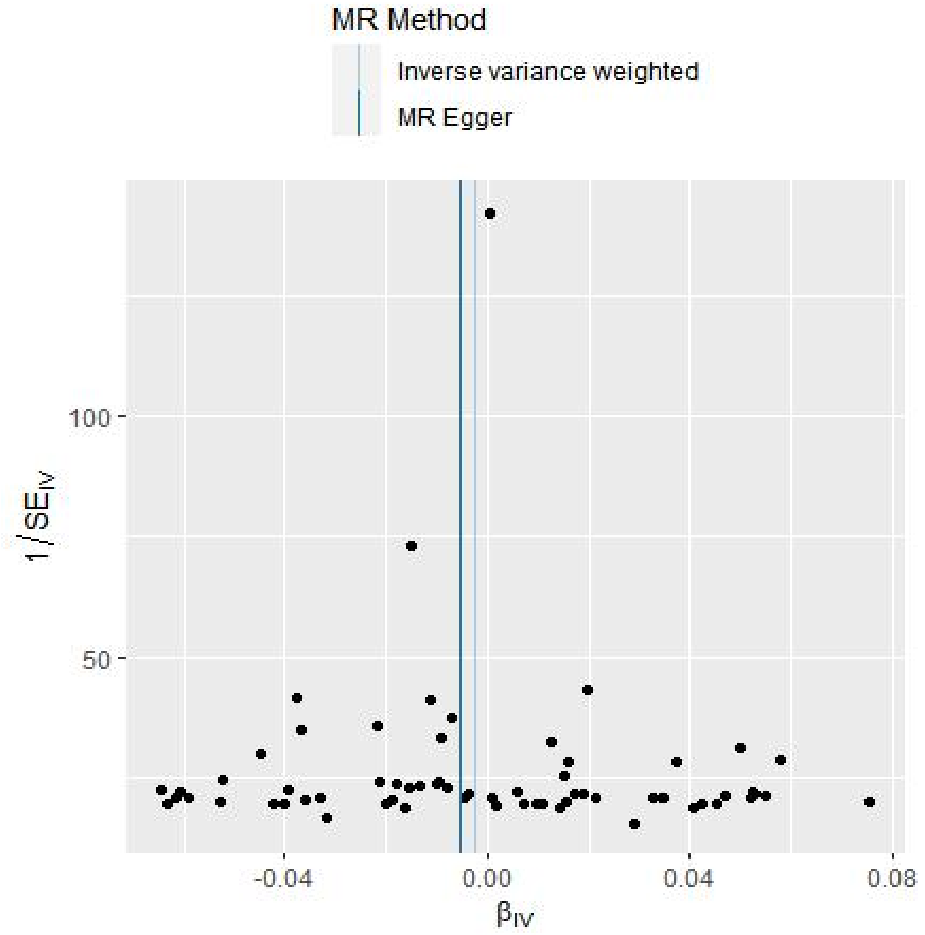

Figure 91: Leave-one-out plot to visualize causal effect of tryptophan on the risk of coronary heart disease when leaving one SNP out.

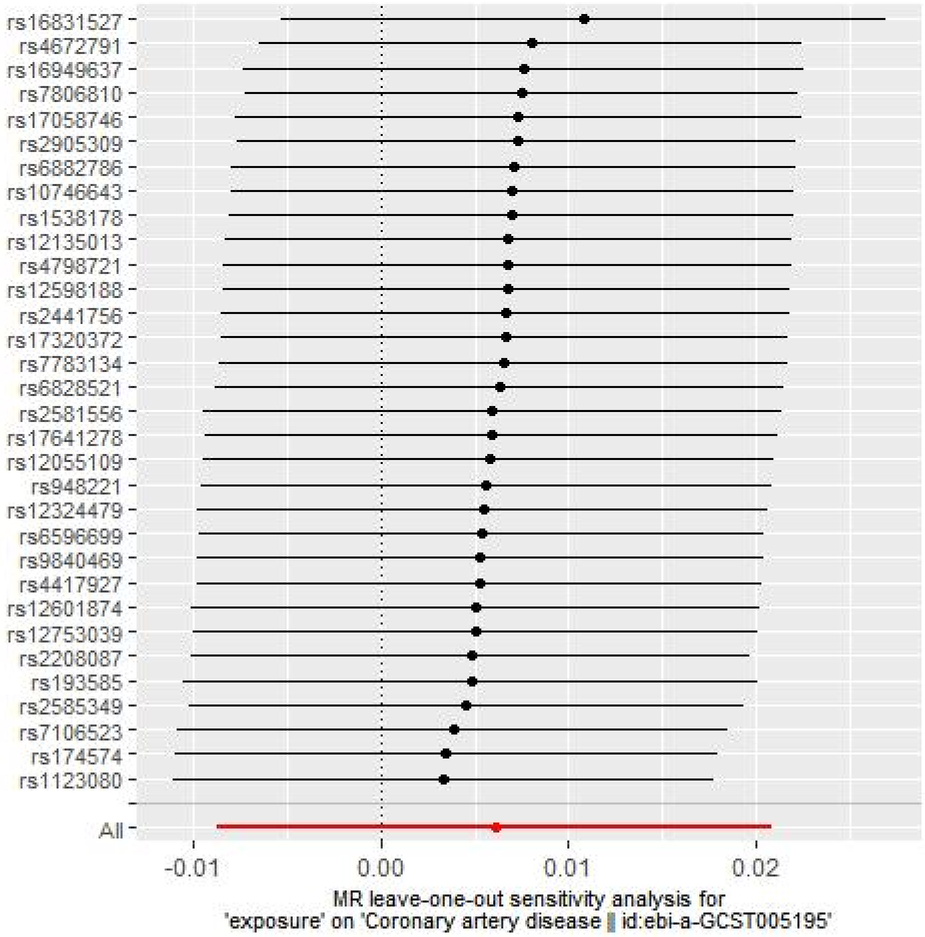

Figure 92: Funnel plots to visualize overall heterogeneity of Mendelian randomization (MR)

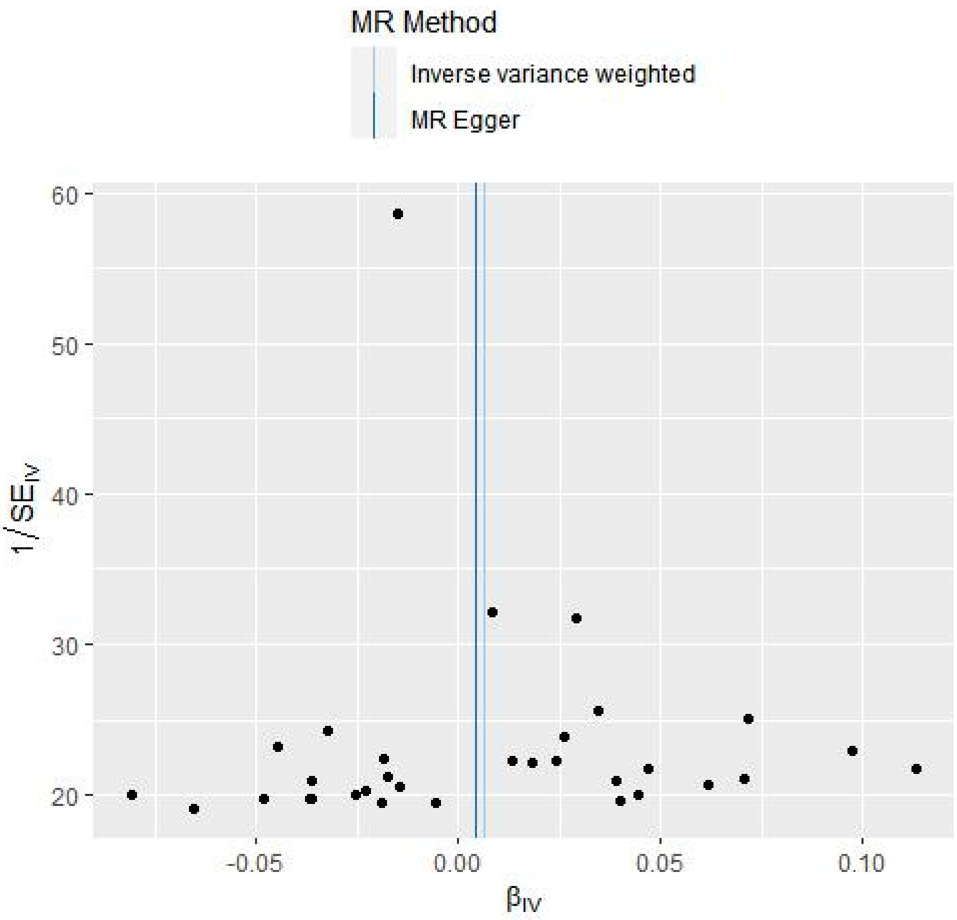

Figure 93: Leave-one-out plot to visualize causal effect of tyrosine on the risk of coronary heart disease when leaving one SNP out.

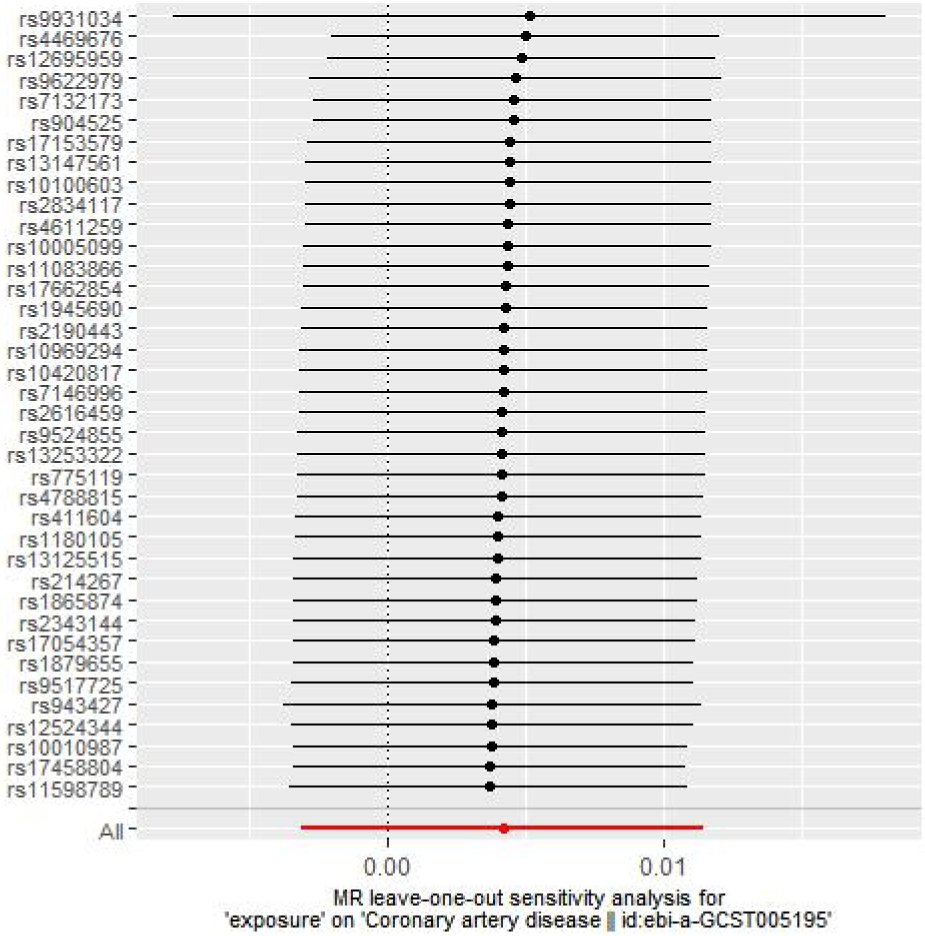

Figure 94: Funnel plots to visualize overall heterogeneity of Mendelian randomization (MR)

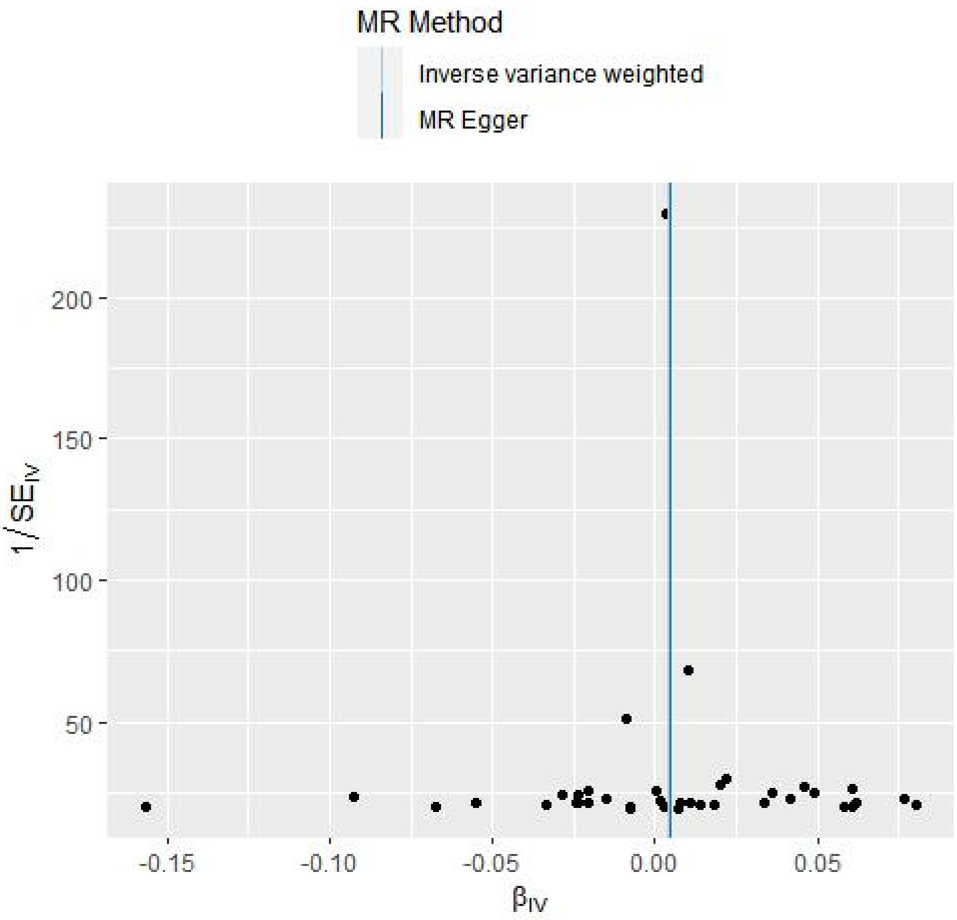

Figure 95: Leave-one-out plot to visualize causal effect of propionic acid on the risk of coronary heart disease when leaving one SNP out.

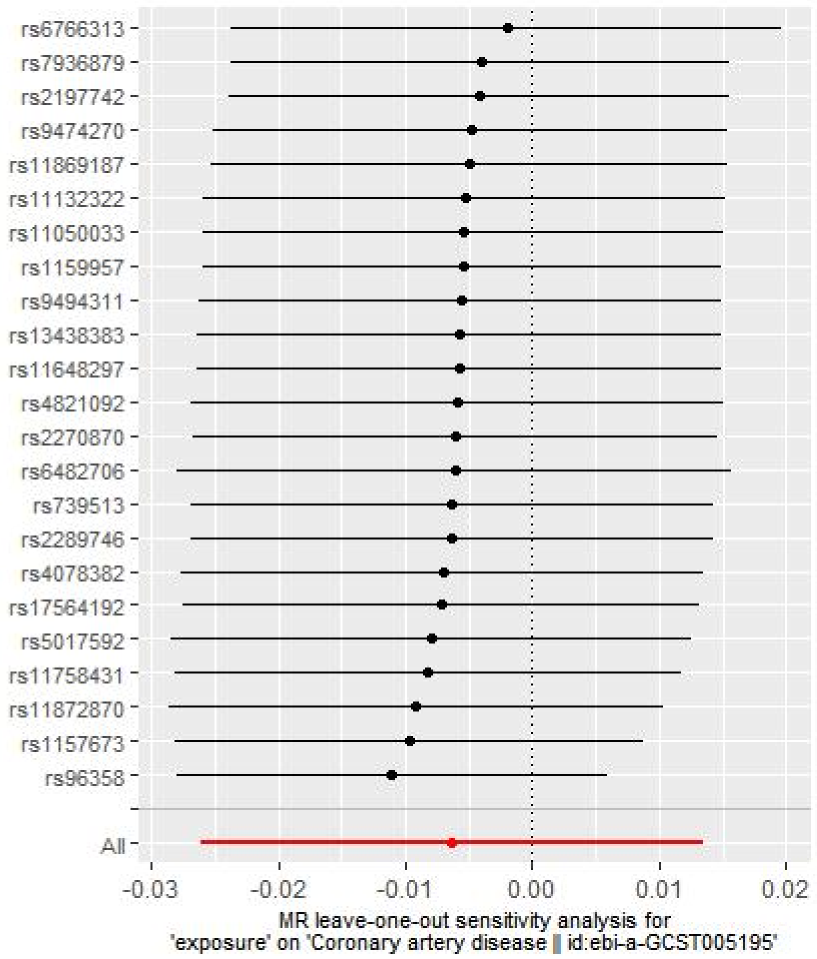

Figure 96: Funnel plots to visualize overall heterogeneity of Mendelian randomization (MR)

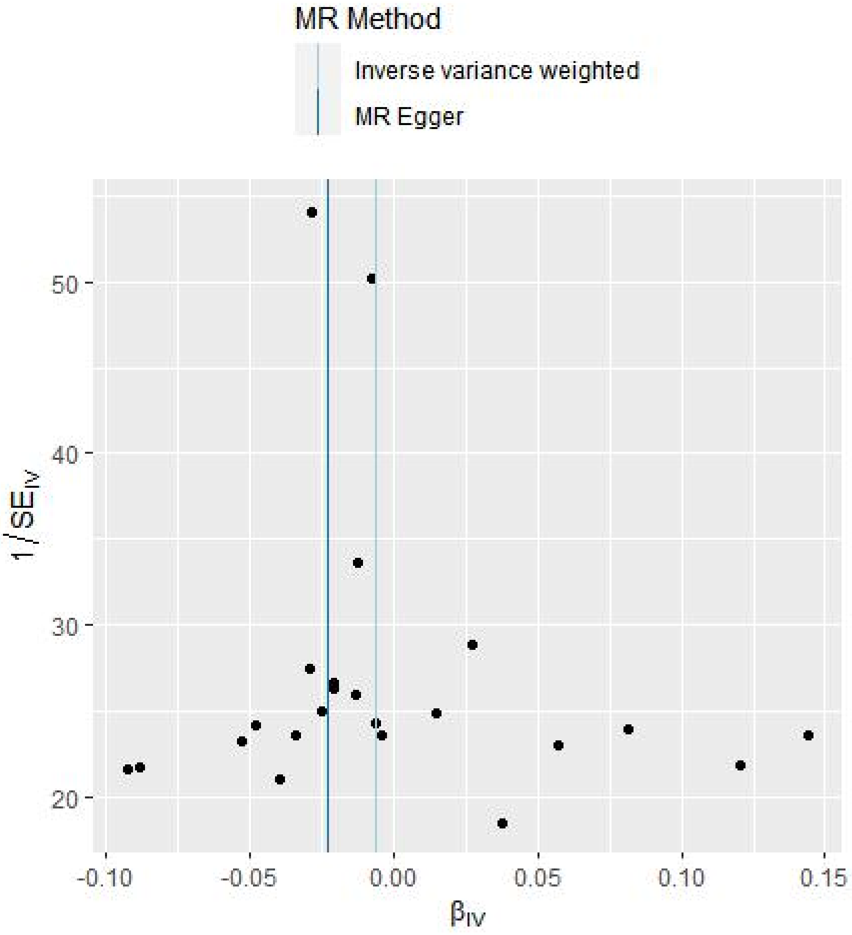

Figure 97: Leave-one-out plot to visualize causal effect of beta_hydroxybutyric acid on the risk of dilated cardiomyopathy when leaving one SNP out.

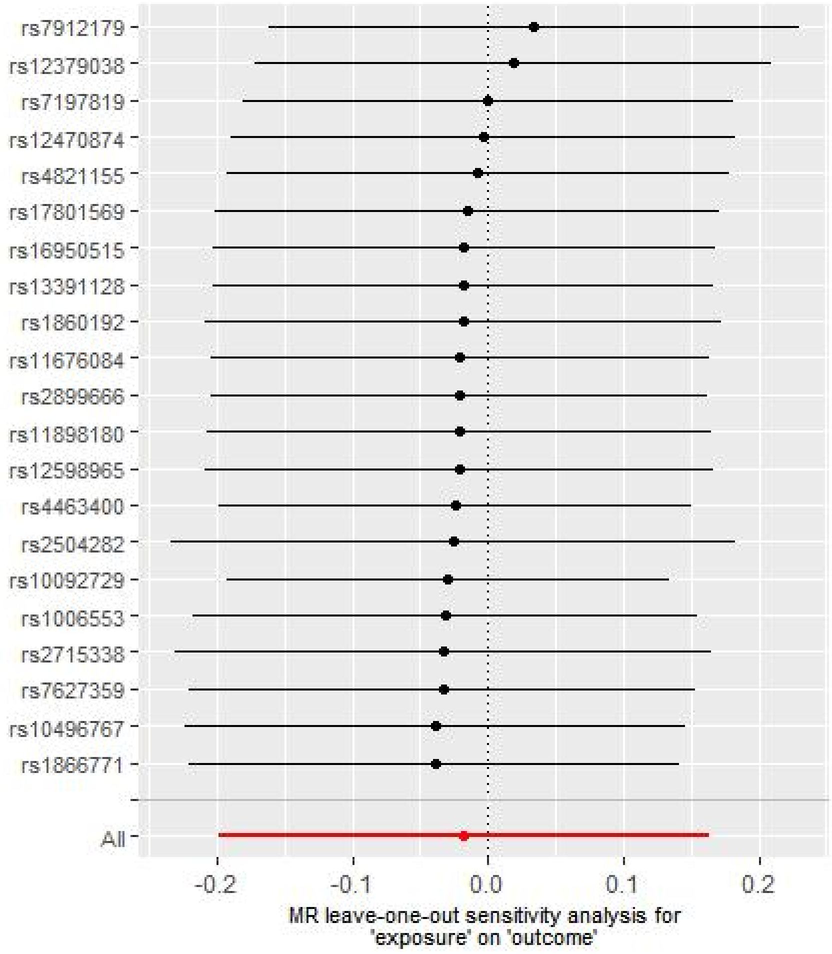

Figure 98: Funnel plots to visualize overall heterogeneity of Mendelian randomization (MR)

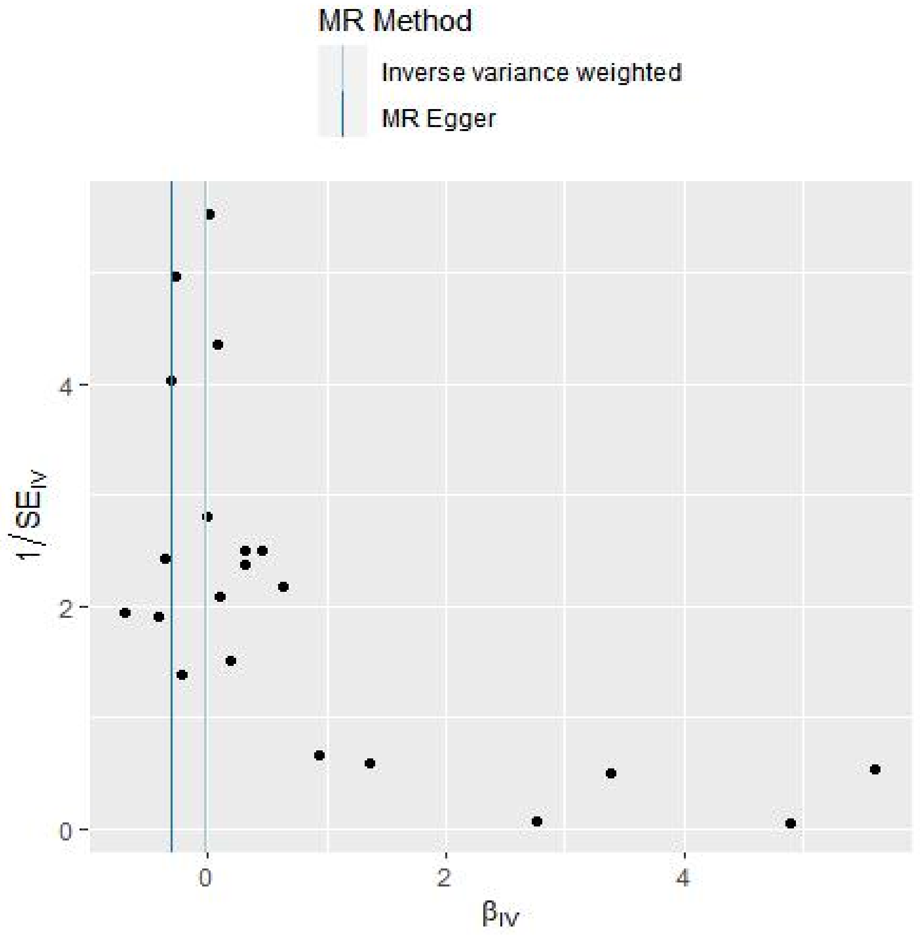

Figure 99: Leave-one-out plot to visualize causal effect of betaine on the risk of dilated cardiomyopathy when leaving one SNP out.

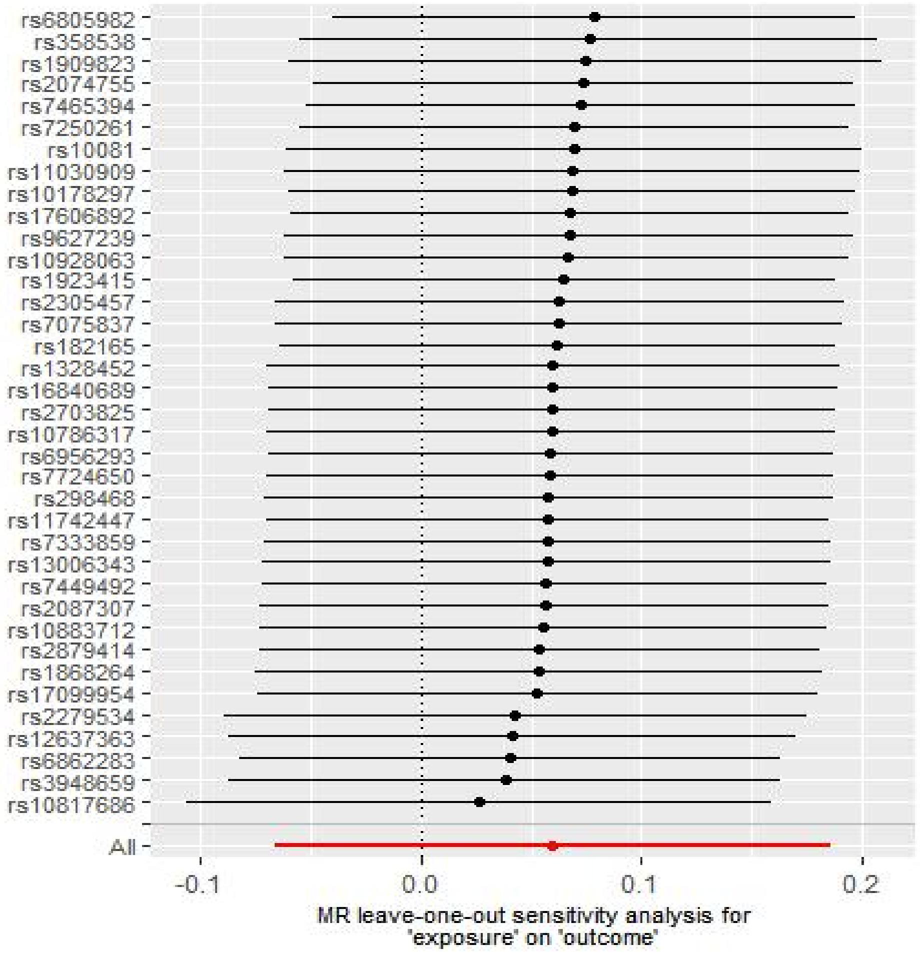

Figure 100: Funnel plots to visualize overall heterogeneity of Mendelian randomization (MR)

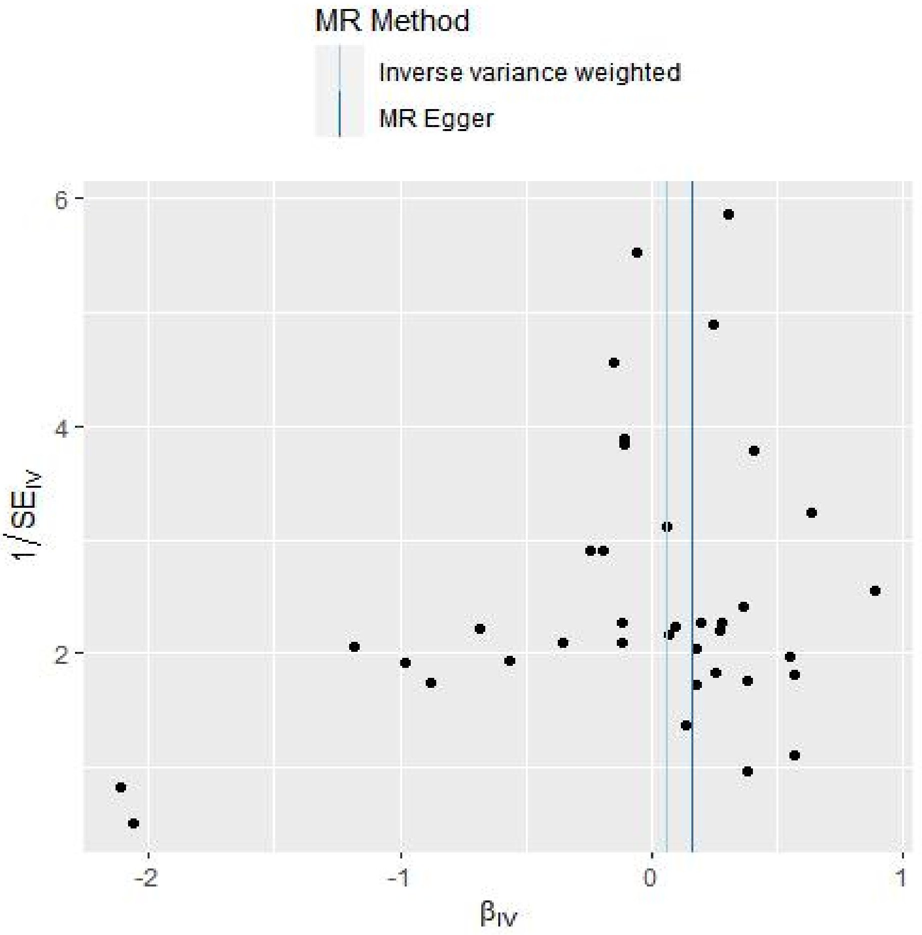

Supplement: Supplementary file 1 [file Data_Sheet_1.zip › Supplementary Material/Supplementary File 2.pdf]
