# Supplementary material for: Effects of Gut Microbiota and Metabolites on Heart Failure and Its Risk Factors: A Two-Sample Mendelian Randomization Study
Source: Front Nutr. 2022 Jun 20;9:899746. doi: 10.3389/fnut.2022.899746 (PMC9253861; doi:10.3389/fnut.2022.899746)
Supplement: Supplementary file 1 [file Data_Sheet_1.zip › Supplementary Material/Supplementary File 3.pdf]

Supplementary Information

Effects of Gut Microbiota and Metabolites on Heart Failure and Its Risk Factors: A Two-sample Mendelian Randomization Study

Qiang Luo[a], Yilan Hu[a], Xin Chen[a], Yong Luo[a], Jie Chen[a], Han Wang[a, *]

Figure 1-24: Effects of gut metabolites and heart failure.

Figure 25-48: Effects of gut metabolites and atrial fibrillation

Figure 49-72: Effects of gut metabolites and hypertrophic cardiomyopathy

Figure 73-96: Effects of gut metabolites and coronary heart disease

Figure 97-120: Effects of gut metabolites and dilated cardiomyopathy

Figure 121-144: Effects of gut metabolites and chronic kidney disease

Figure 145-168: Effects of gut metabolites and systolic blood pressure

Figure 169-192: Effects of gut metabolites and diastolic blood pressure

Figure 193-216: Effects of gut metabolites and diabetes

Figure 217-240: Effects of gut metabolites and myocardial infarction

Figure 241-254: Effects of gut metabolites and myocarditis

Figure 255-278: Effects of gut metabolites and valvular heart disease

Figure 279-284: Effects of gut microbiota and atrial fibrillation

Figure 285-290: Effects of gut microbiota and hypertrophic cardiomyopathy

Figure 291-296: Effects of gut microbiota and coronary heart disease

Figure 297-302: Effects of gut microbiota and dilated cardiomyopathy

Figure 303-308: Effects of gut microbiota and chronic kidney disease

Figure 309-314: Effects of gut microbiota and systolic blood pressure

Figure 315-320: Effects of gut microbiota and diastolic blood pressure

Figure 321-326: Effects of gut microbiota and diabetes

Figure 327-322: Effects of gut microbiota and valvular heart disease

Figure 333-338: Effects of gut microbiota and myocardial infarction

Figure 339-342: Effects of gut microbiota and myocarditis

Figure 343-348: Effects of gut microbiota and heart failure

Figure 101: Leave-one-out plot to visualize causal effect of carnitine on the risk of dilated cardiomyopathy when leaving one SNP out.

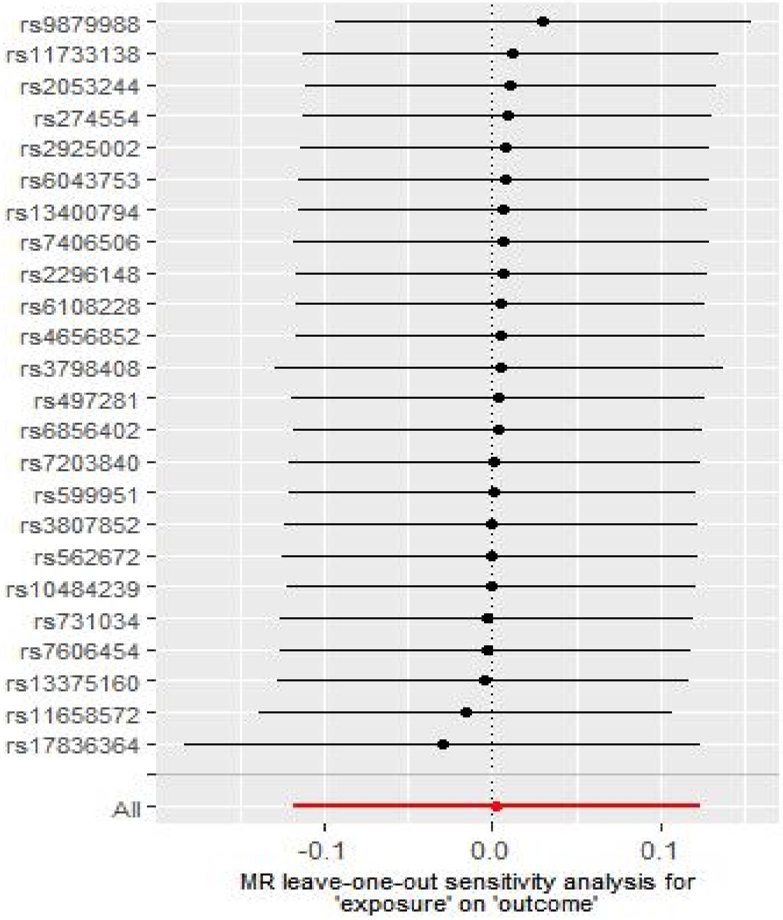

Figure 102: Funnel plots to visualize overall heterogeneity of Mendelian randomization (MR)

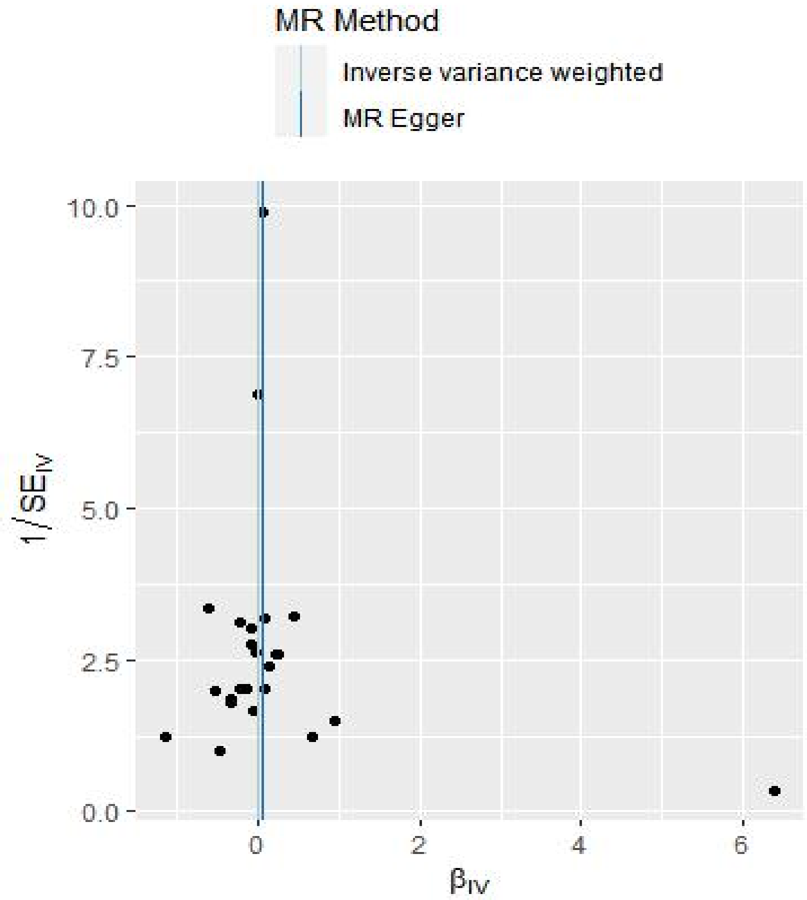

Figure 103: Leave-one-out plot to visualize causal effect of choline on the risk of dilated cardiomyopathy when leaving one SNP out.

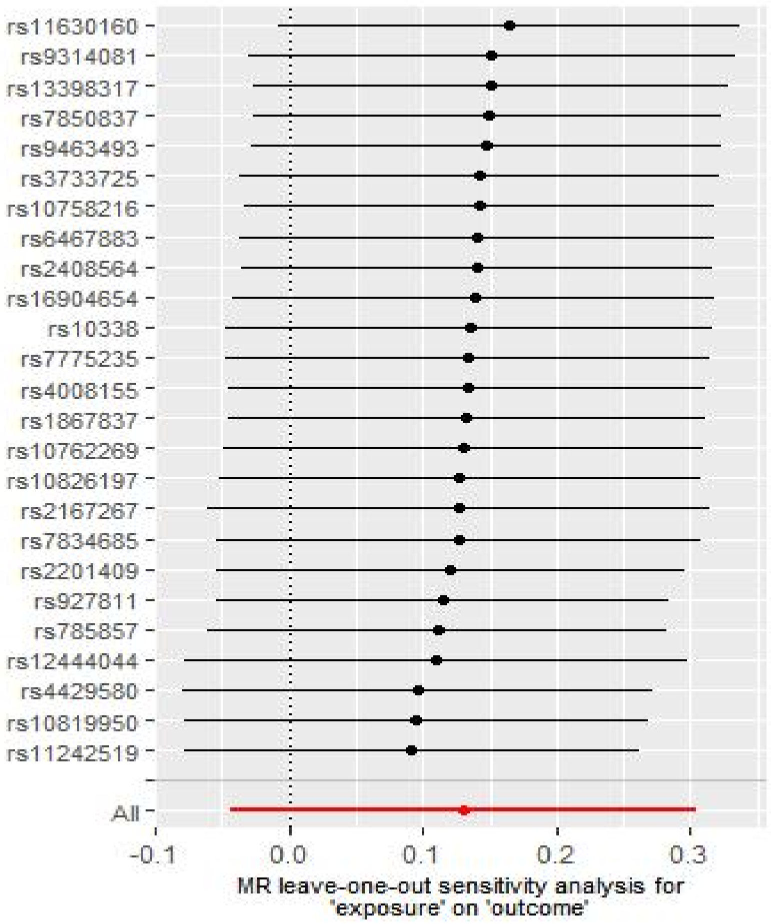

Figure 104: Funnel plots to visualize overall heterogeneity of Mendelian randomization (MR)

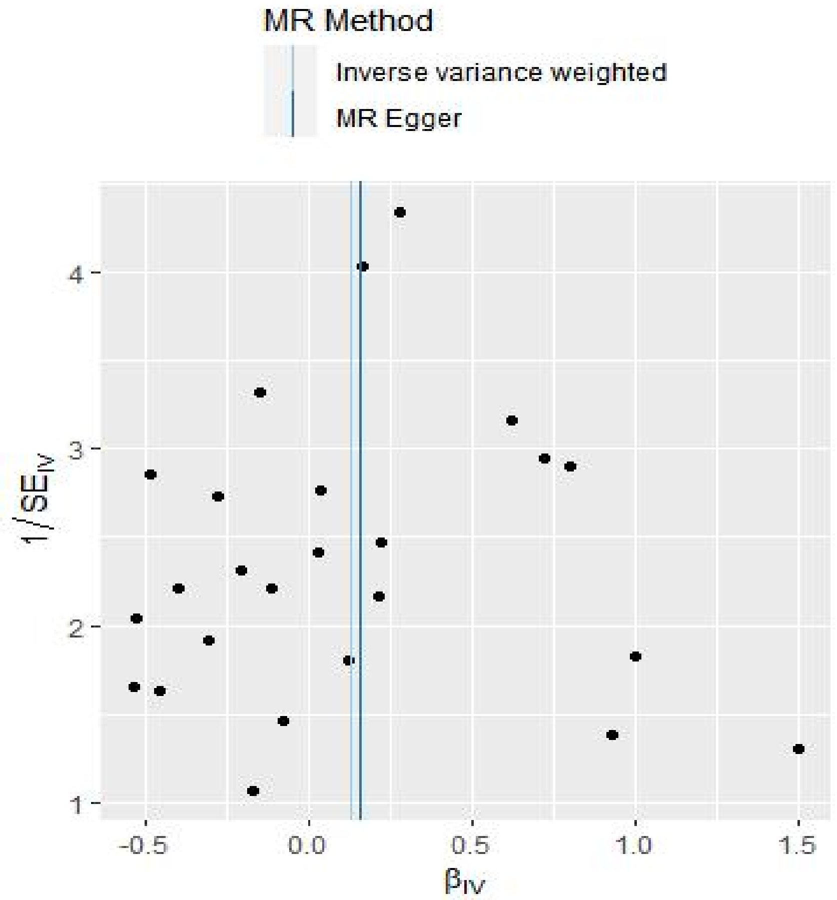

Figure 105: Leave-one-out plot to visualize causal effect of glutamate on the risk of dilated cardiomyopathy when leaving one SNP out.

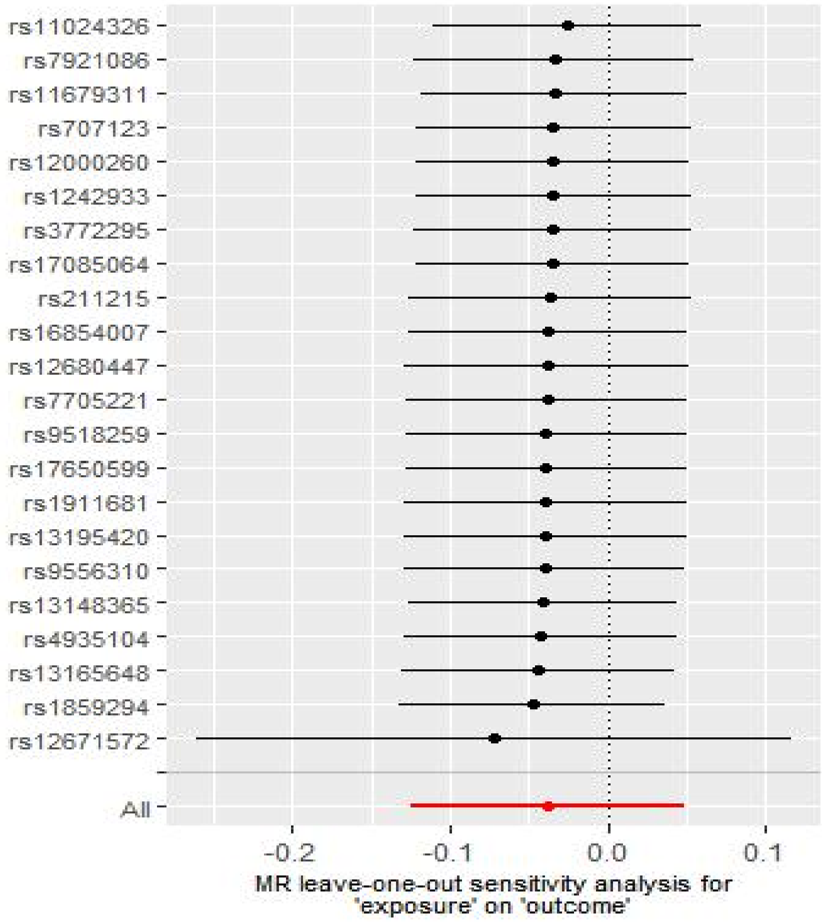

Figure 106: Funnel plots to visualize overall heterogeneity of Mendelian randomization (MR)

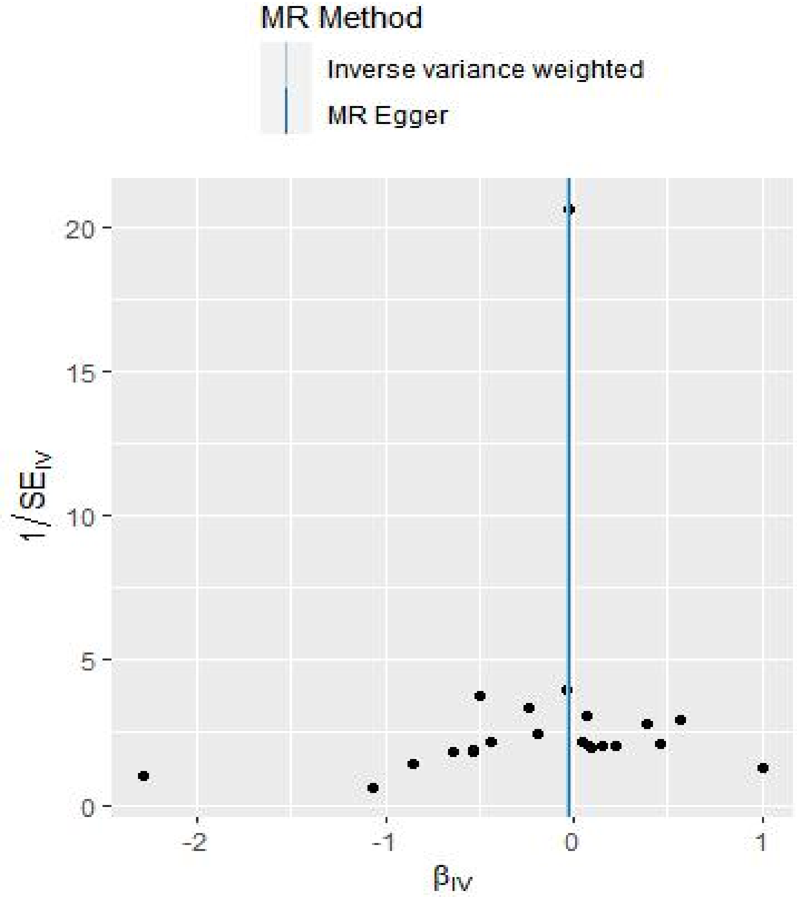

Figure 107: Leave-one-out plot to visualize causal effect of kynuremine on the risk of dilated cardiomyopathy when leaving one SNP out.

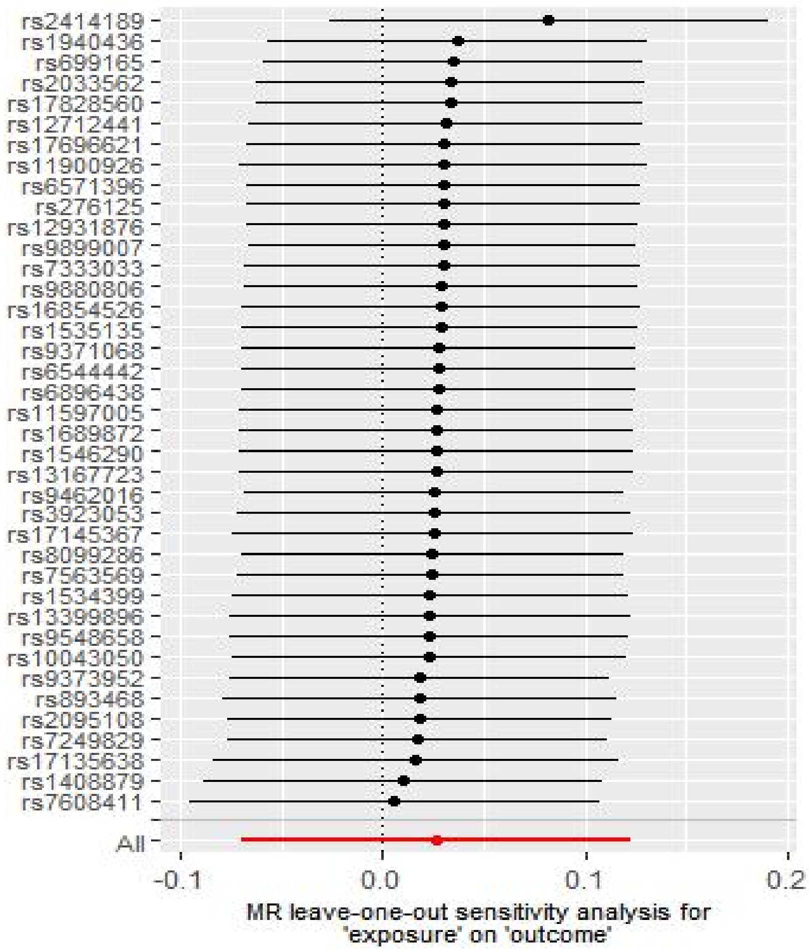

Figure 108: Funnel plots to visualize overall heterogeneity of Mendelian randomization (MR)

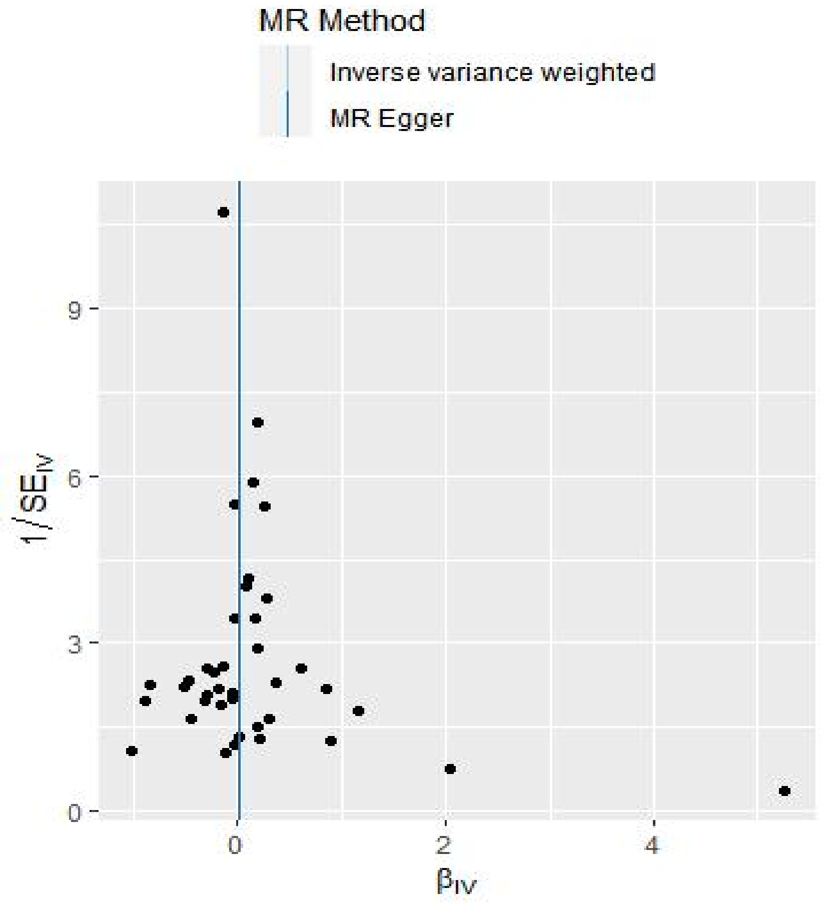

Figure 109: Leave-one-out plot to visualize causal effect of phenylalanine on the risk of dilated cardiomyopathy when leaving one SNP out.

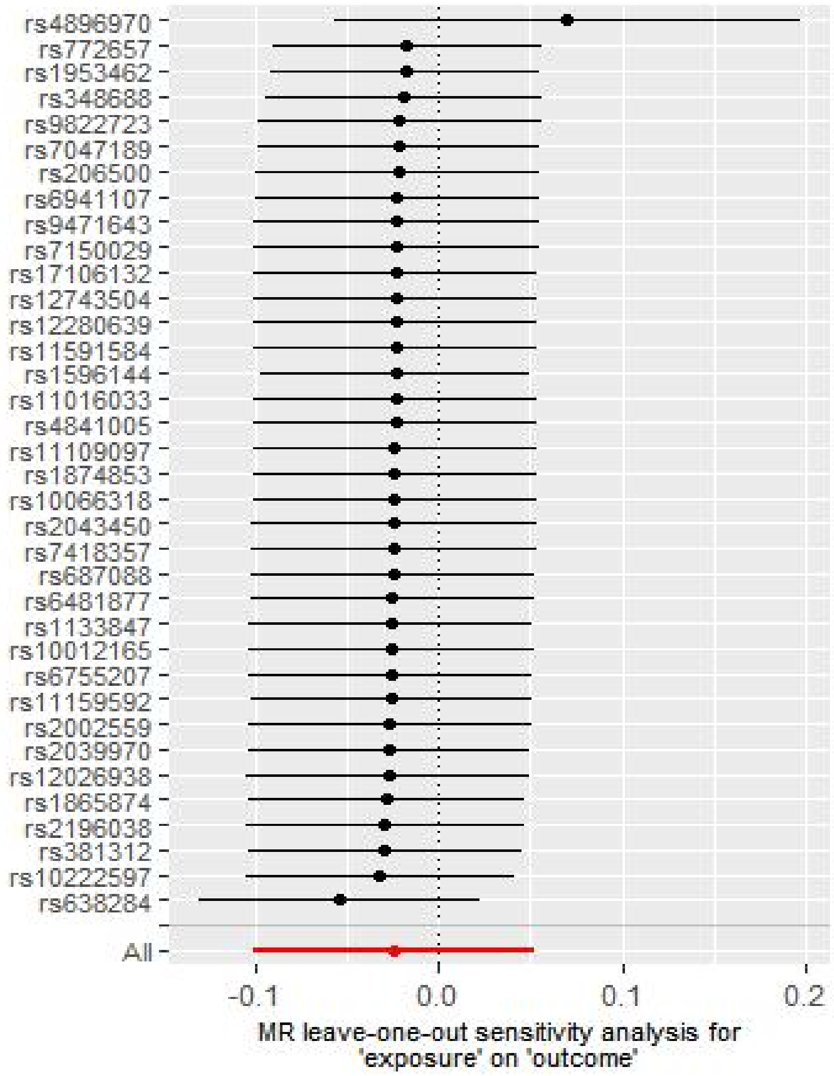

Figure 110: Funnel plots to visualize overall heterogeneity of Mendelian randomization (MR)

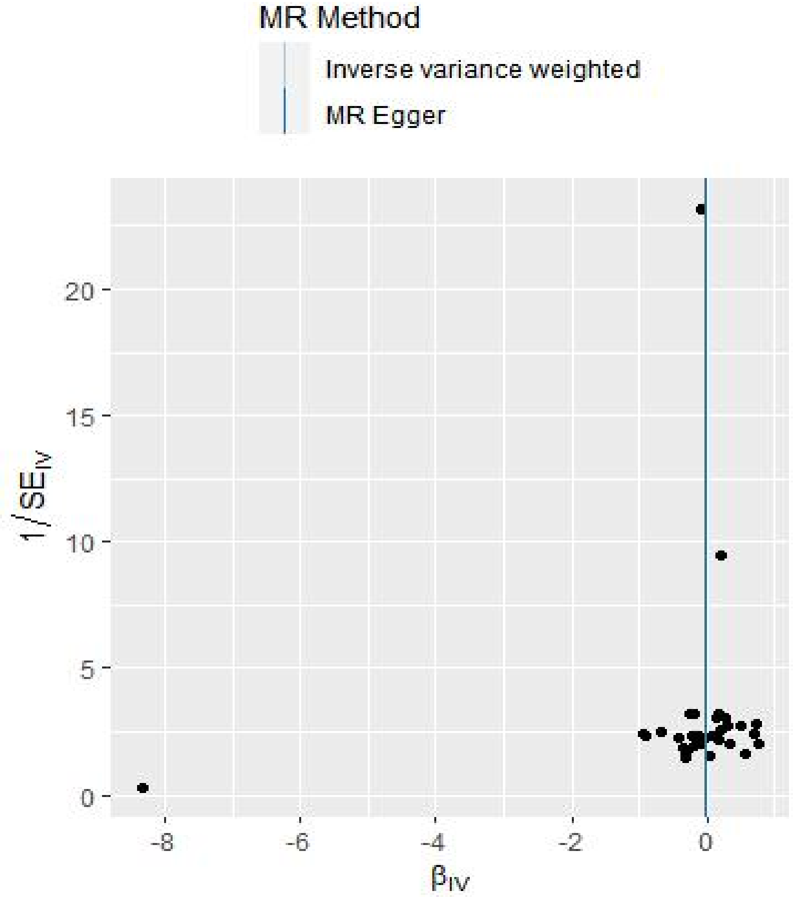

Figure 111: Leave-one-out plot to visualize causal effect of serotonin on the risk of dilated cardiomyopathy when leaving one SNP out.

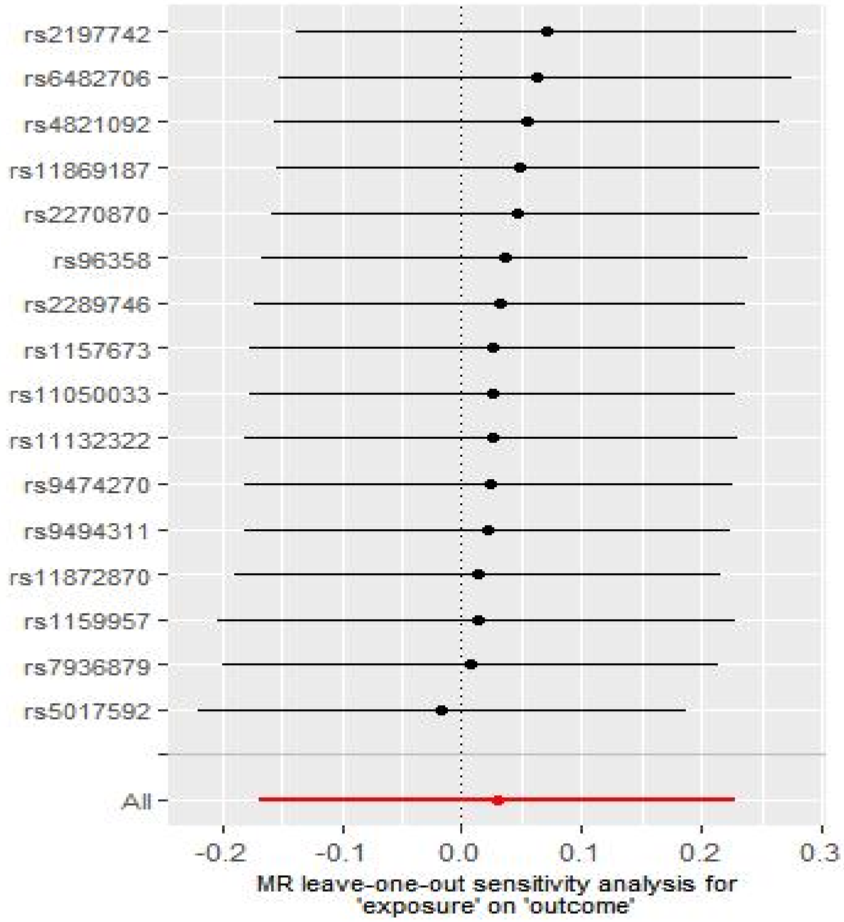

Figure 112: Funnel plots to visualize overall heterogeneity of Mendelian randomization (MR)

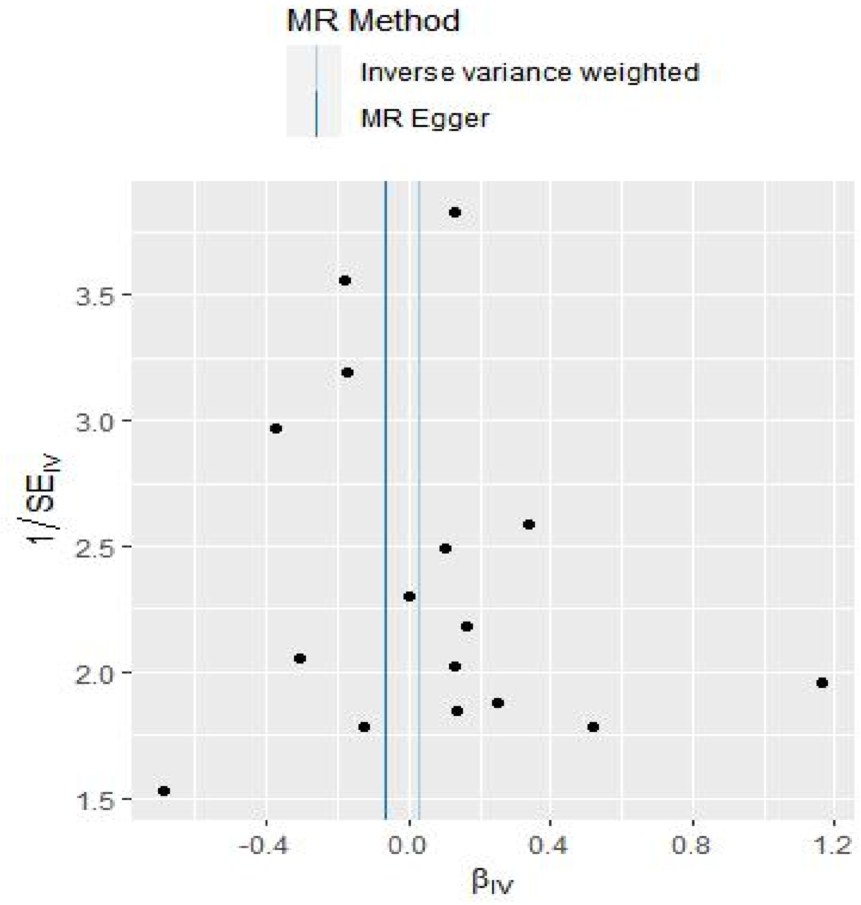

Figure 113: Leave-one-out plot to visualize causal effect of trimethylamine_N_oxide on the risk of dilated cardiomyopathy when leaving one SNP out.

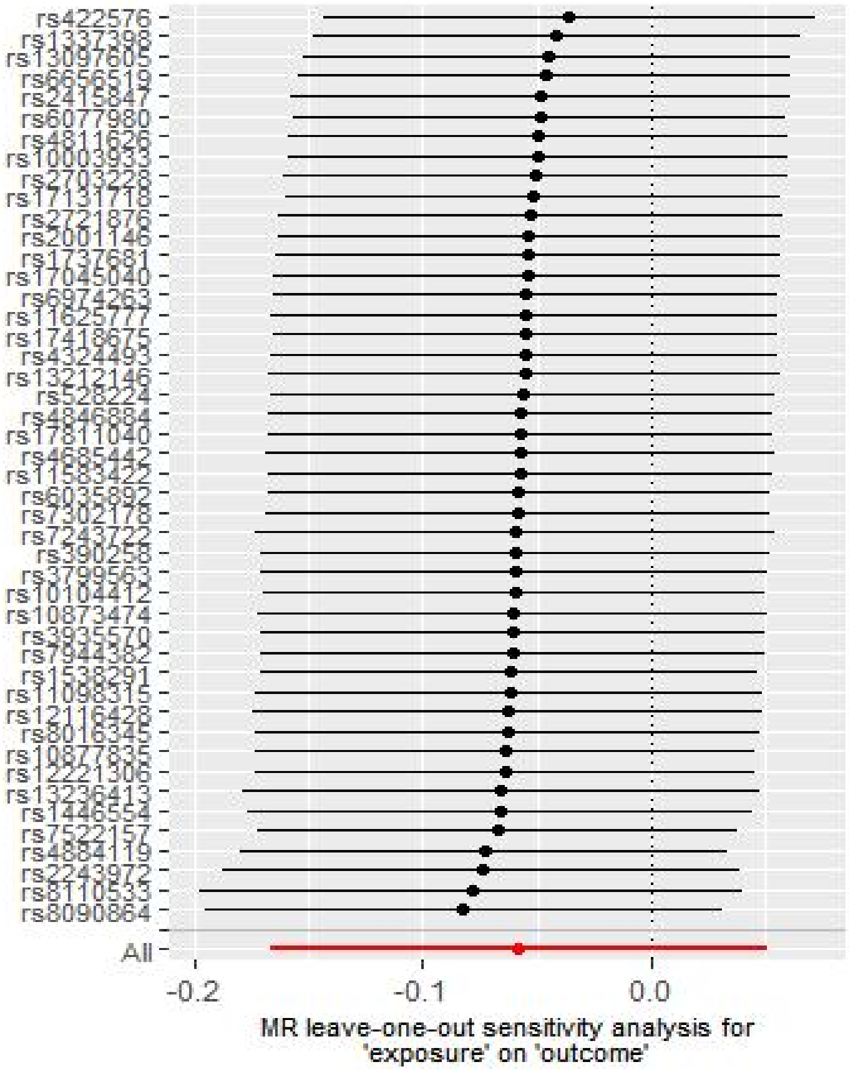

Figure 114: Funnel plots to visualize overall heterogeneity of Mendelian randomization (MR)

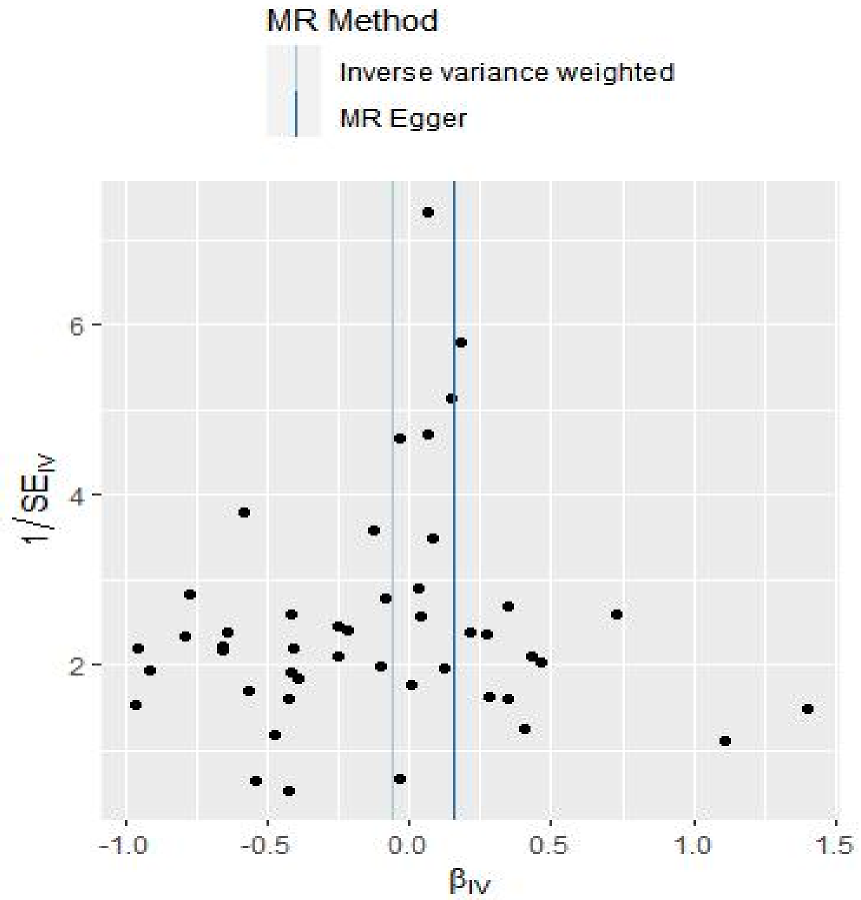

Figure 115: Leave-one-out plot to visualize causal effect of tryptophan on the risk of dilated cardiomyopathy when leaving one SNP out.

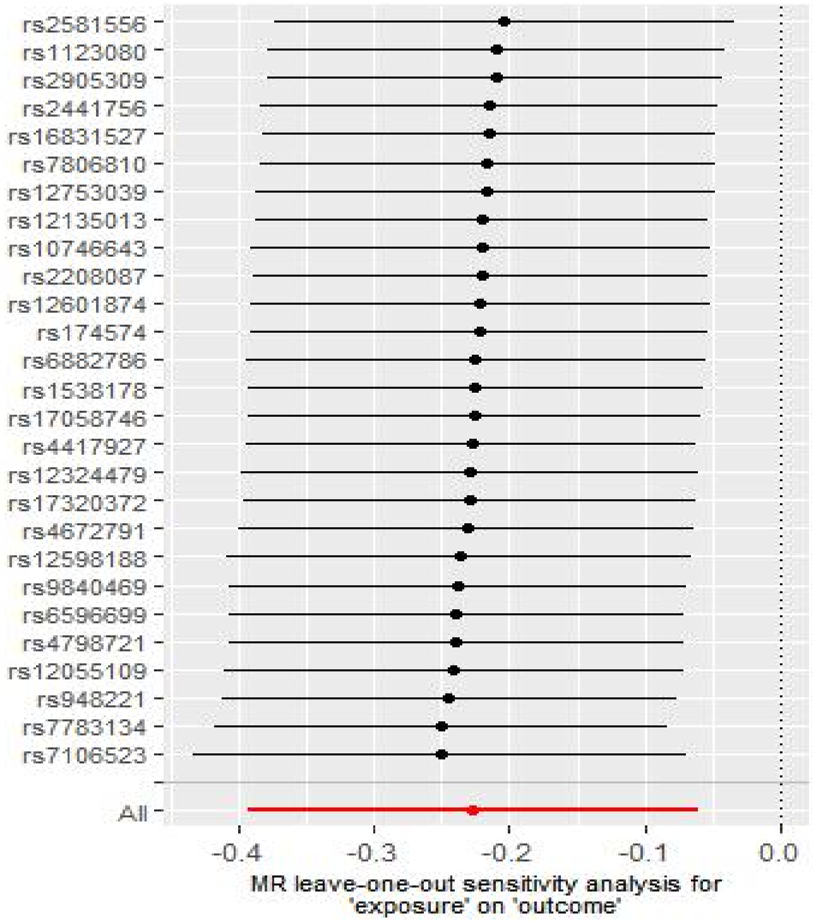

Figure 116: Funnel plots to visualize overall heterogeneity of Mendelian randomization (MR)

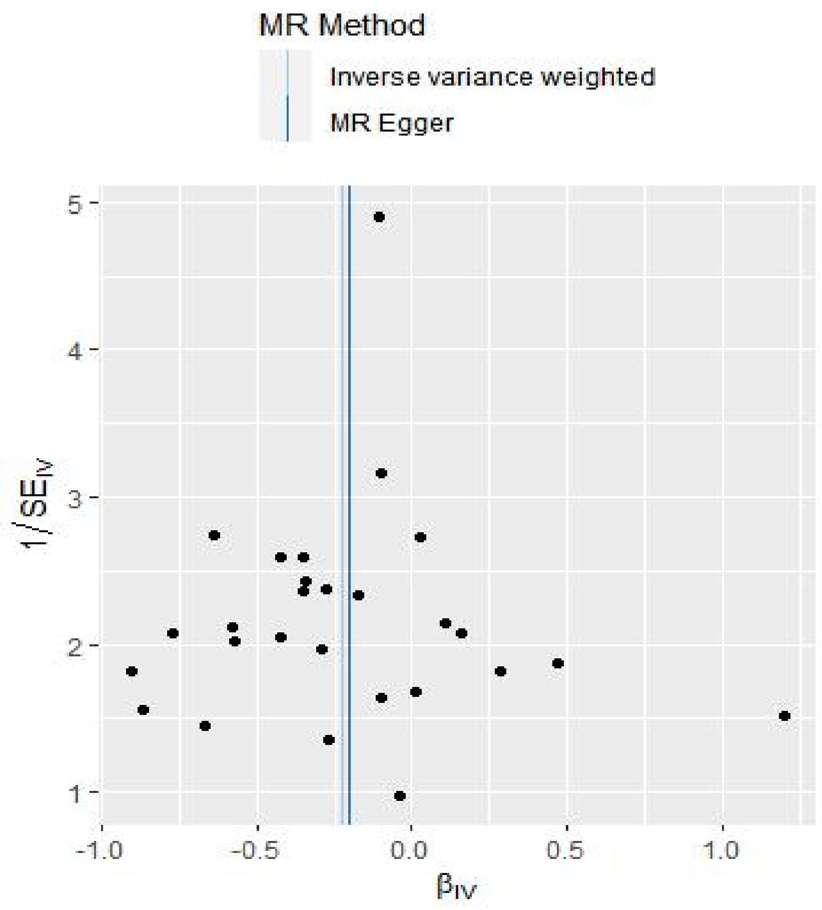

Figure 117: Leave-one-out plot to visualize causal effect of tyrosine on the risk of dilated cardiomyopathy when leaving one SNP out.

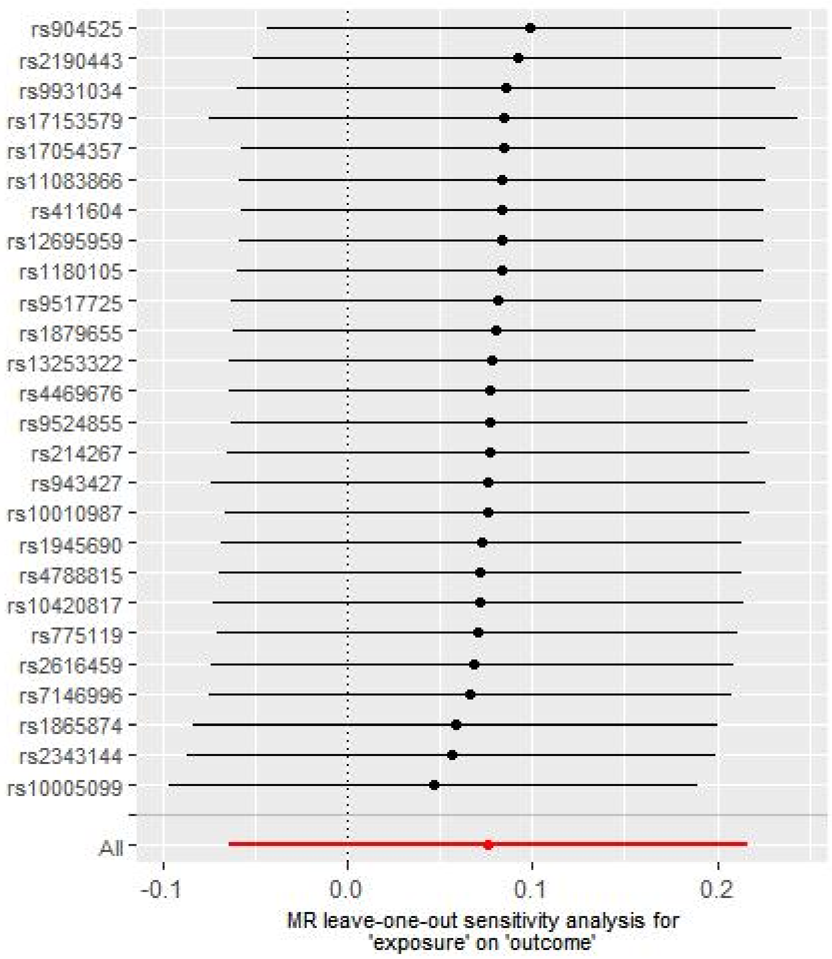

Figure 118: Funnel plots to visualize overall heterogeneity of Mendelian randomization (MR)

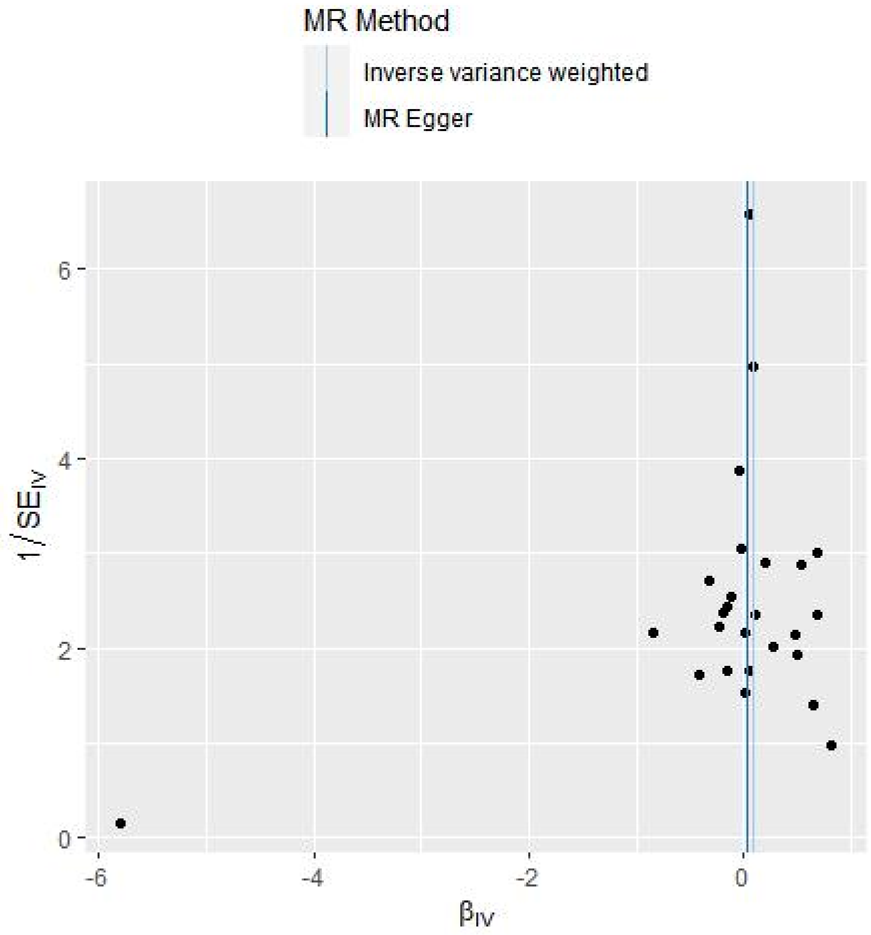

Figure 119: Leave-one-out plot to visualize causal effect of propionic acid on the risk of dilated cardiomyopathy when leaving one SNP out.

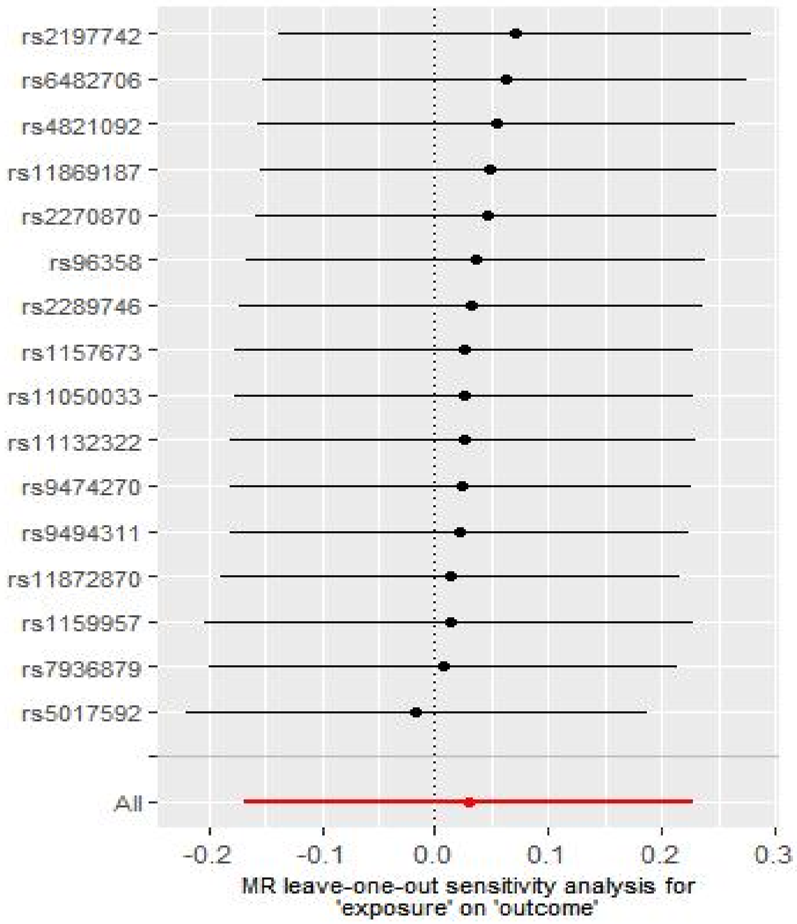

Figure 120: Funnel plots to visualize overall heterogeneity of Mendelian randomization (MR)

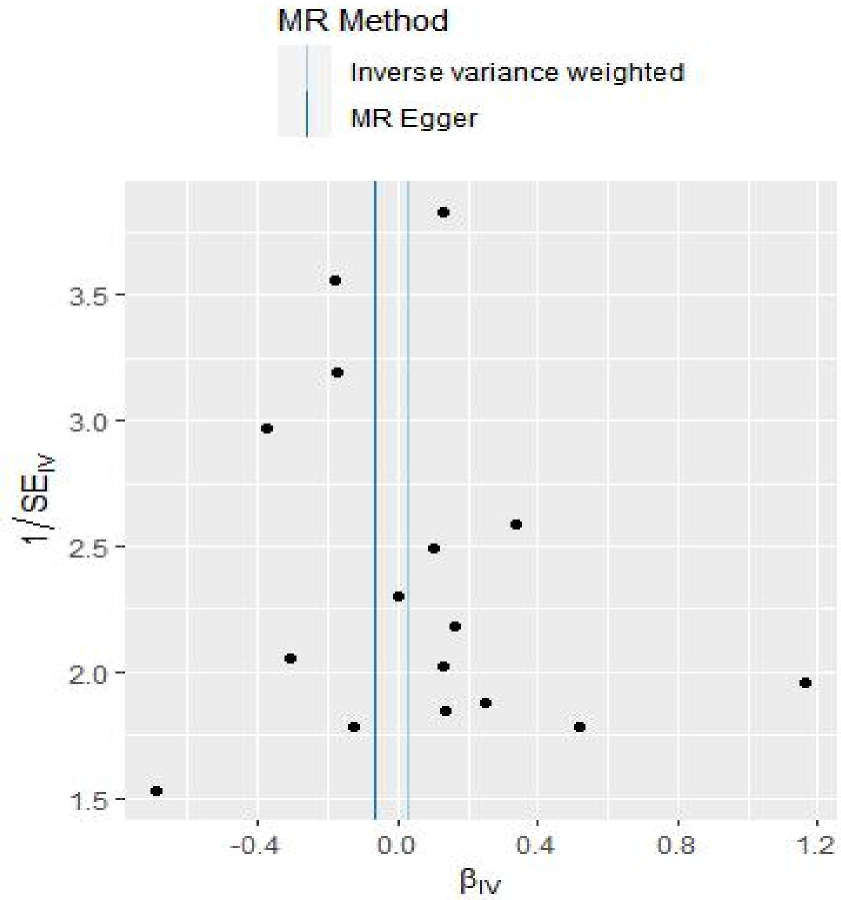

Figure 121: Leave-one-out plot to visualize causal effect of beta_hydroxybutyric acid on the risk of chronic kidney disease when leaving one SNP out.

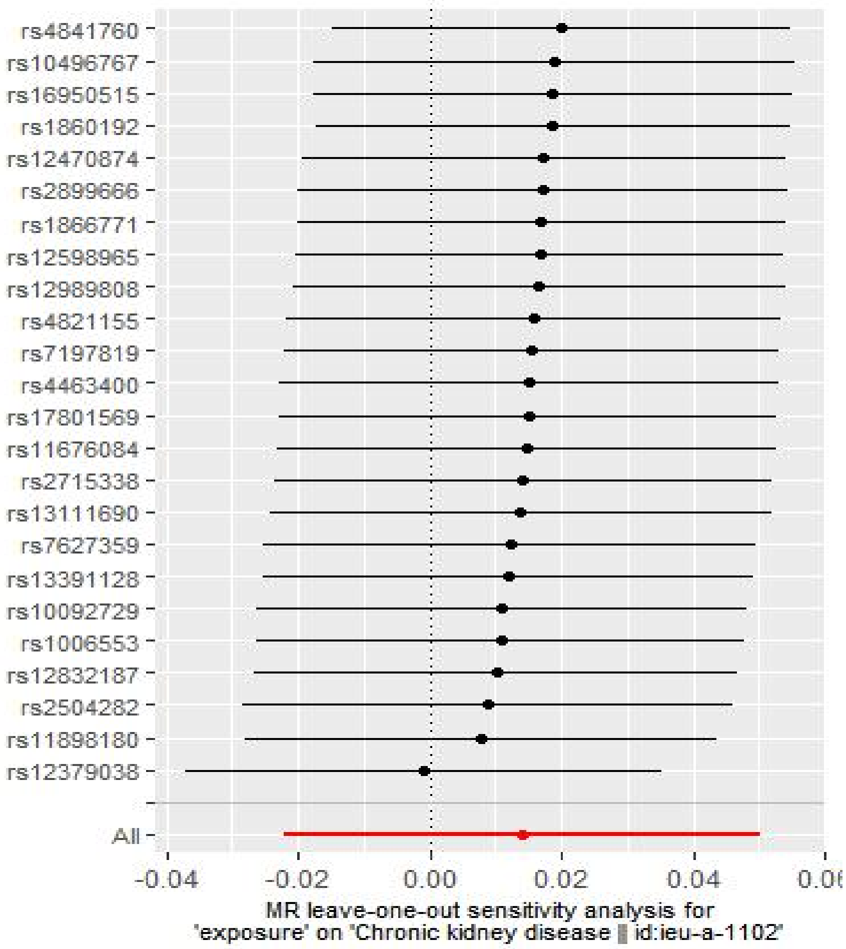

Figure 122: Funnel plots to visualize overall heterogeneity of Mendelian randomization (MR)

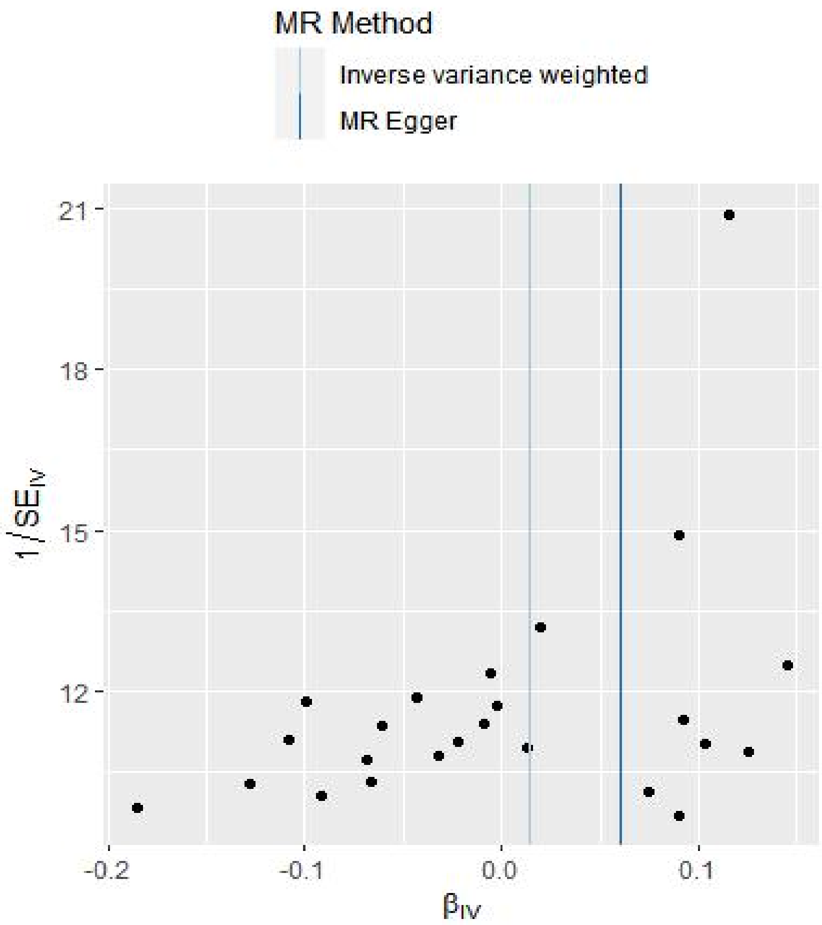

Figure 123: Leave-one-out plot to visualize causal effect of betaine on the risk of chronic kidney disease when leaving one SNP out.

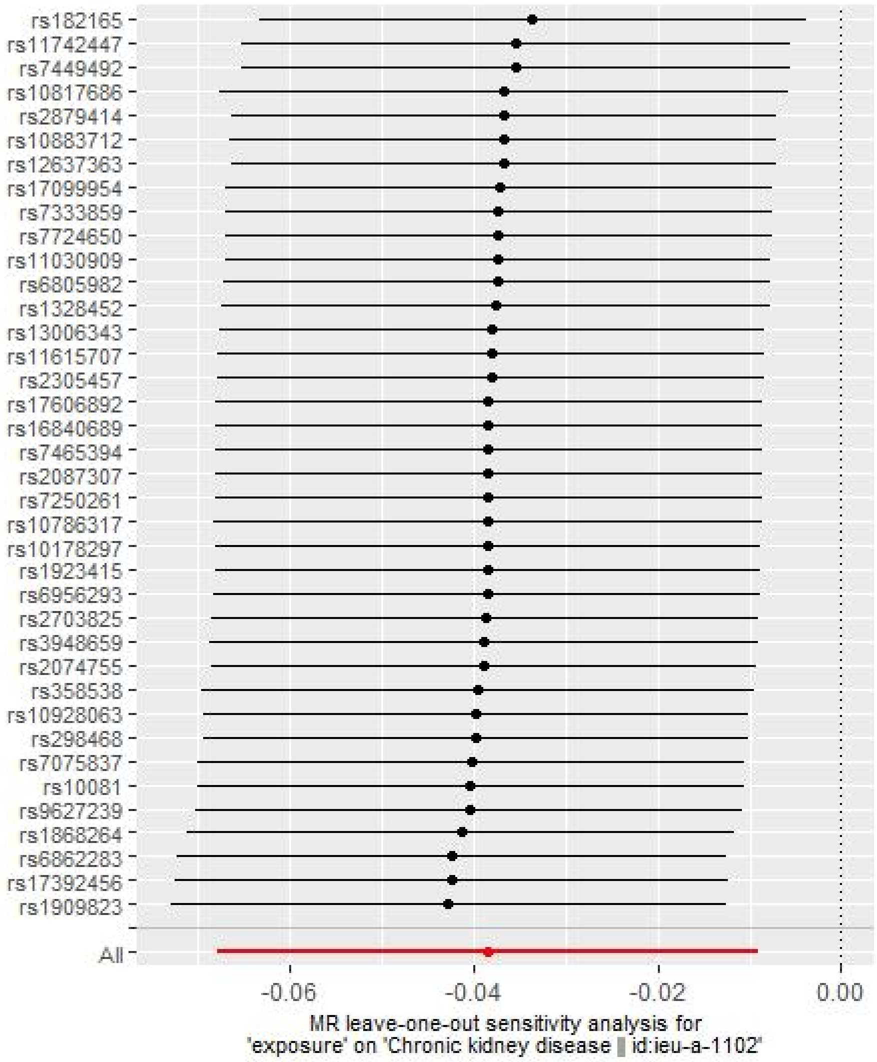

Figure 124: Funnel plots to visualize overall heterogeneity of Mendelian randomization (MR)

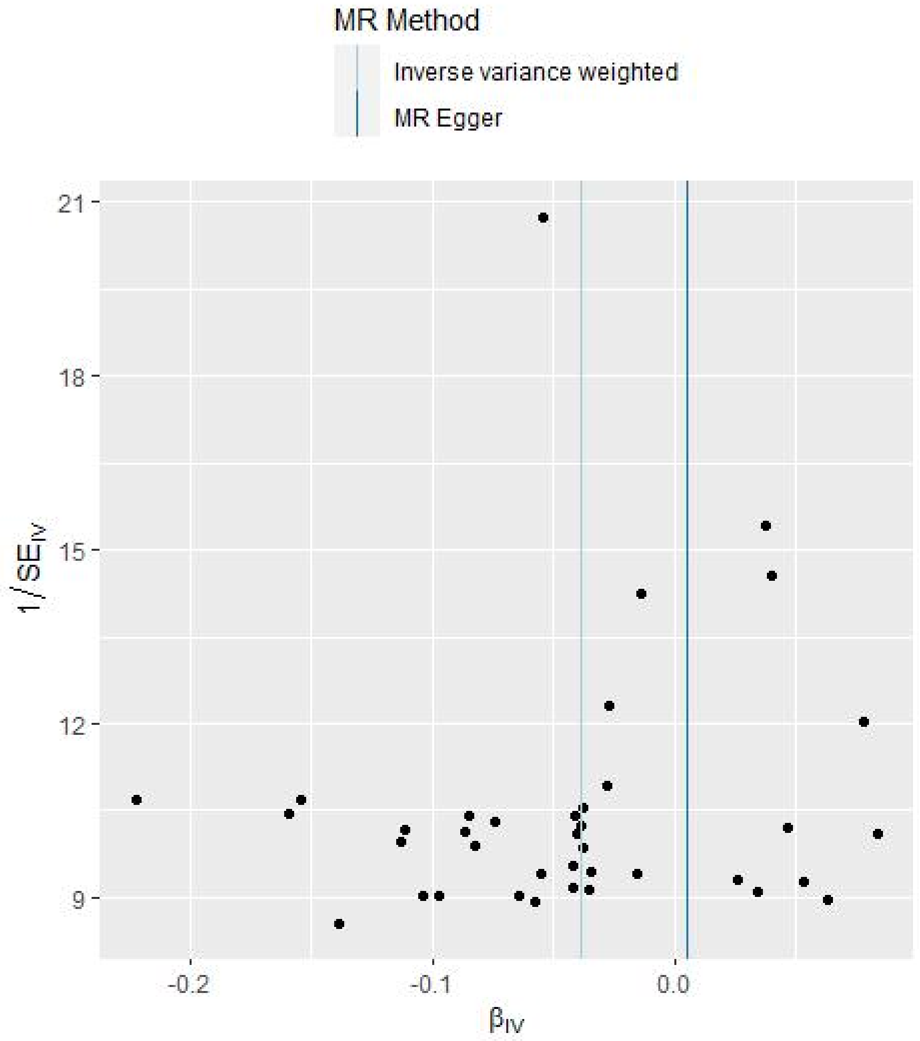

Figure 125: Leave-one-out plot to visualize causal effect of carnitine on the risk of chronic kidney disease when leaving one SNP out.

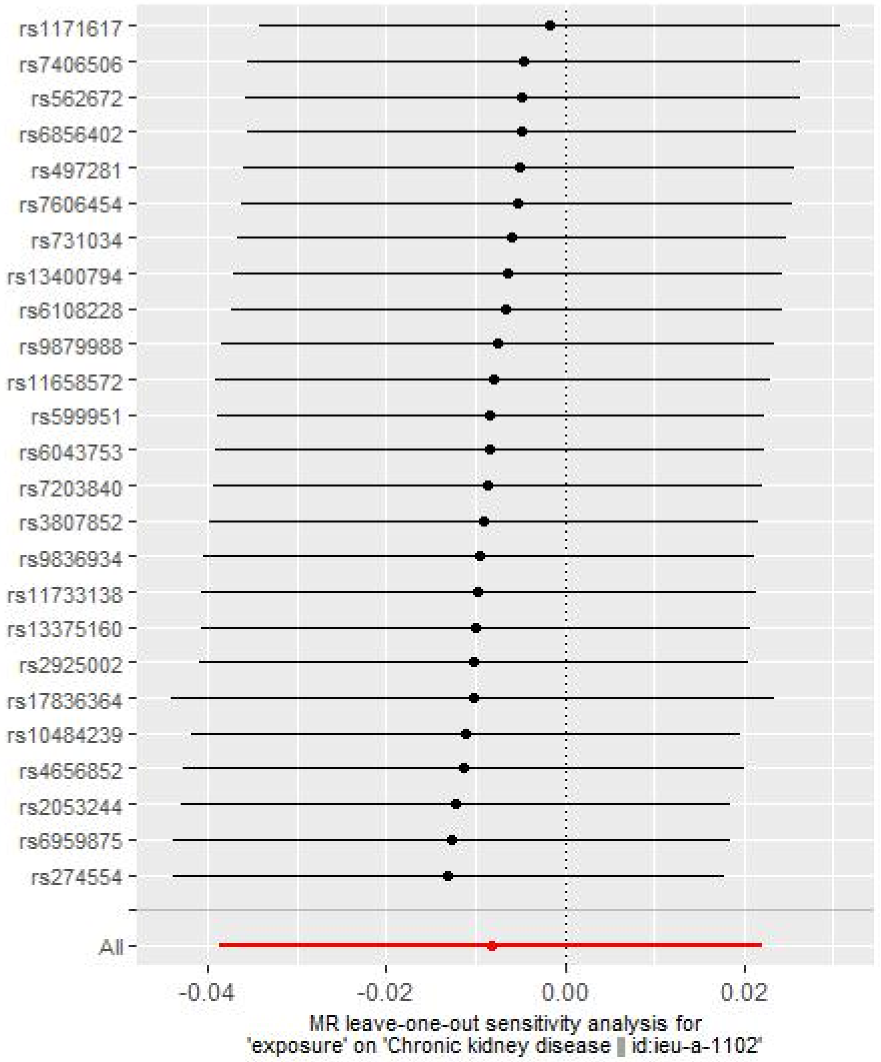

Figure 126: Funnel plots to visualize overall heterogeneity of Mendelian randomization (MR)

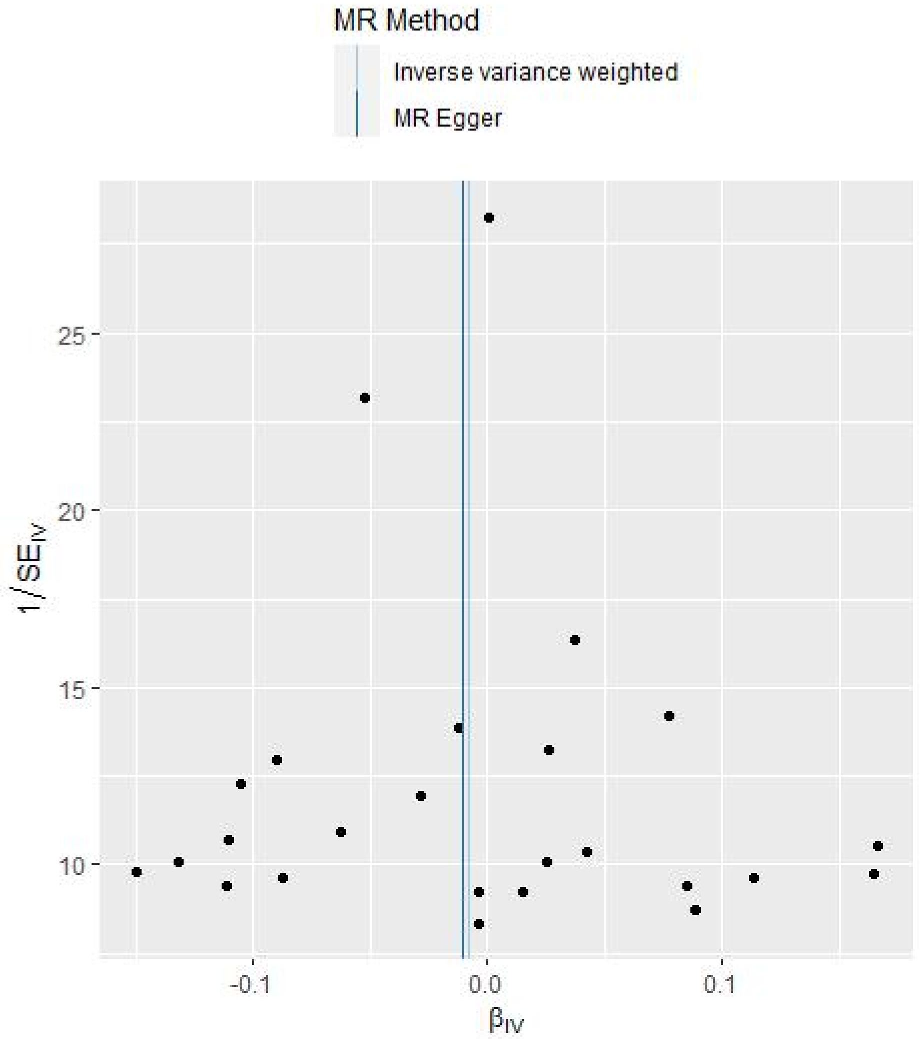

Figure 127: Leave-one-out plot to visualize causal effect of choline on the risk of chronic kidney disease when leaving one SNP out.

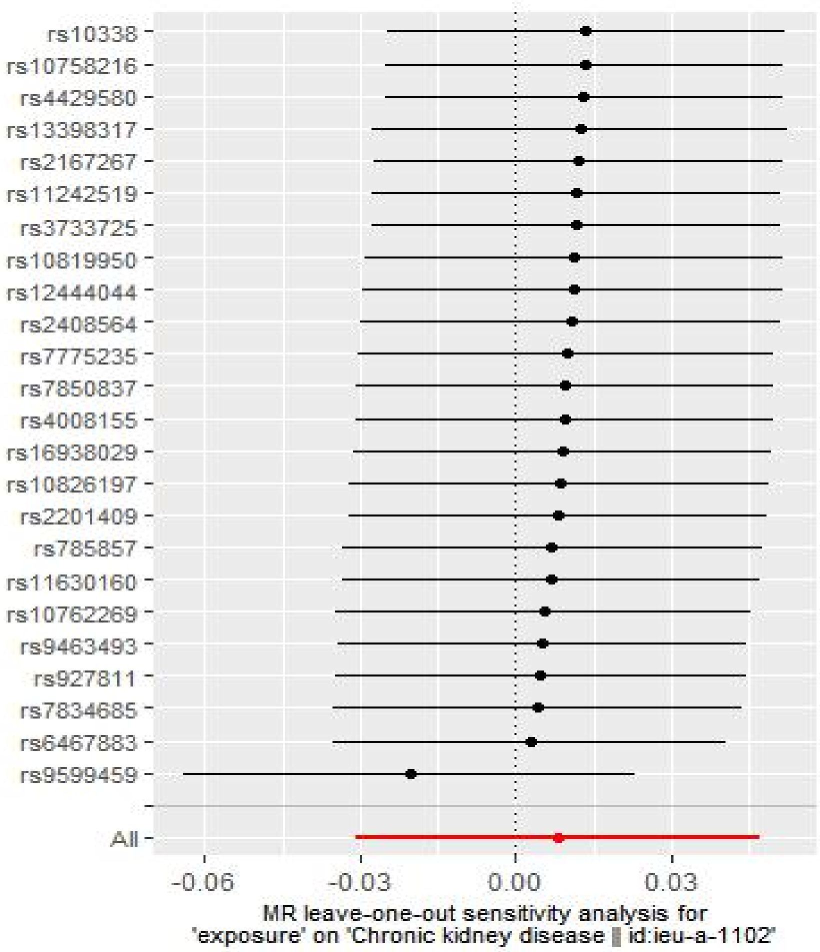

Figure 128: Funnel plots to visualize overall heterogeneity of Mendelian randomization (MR)

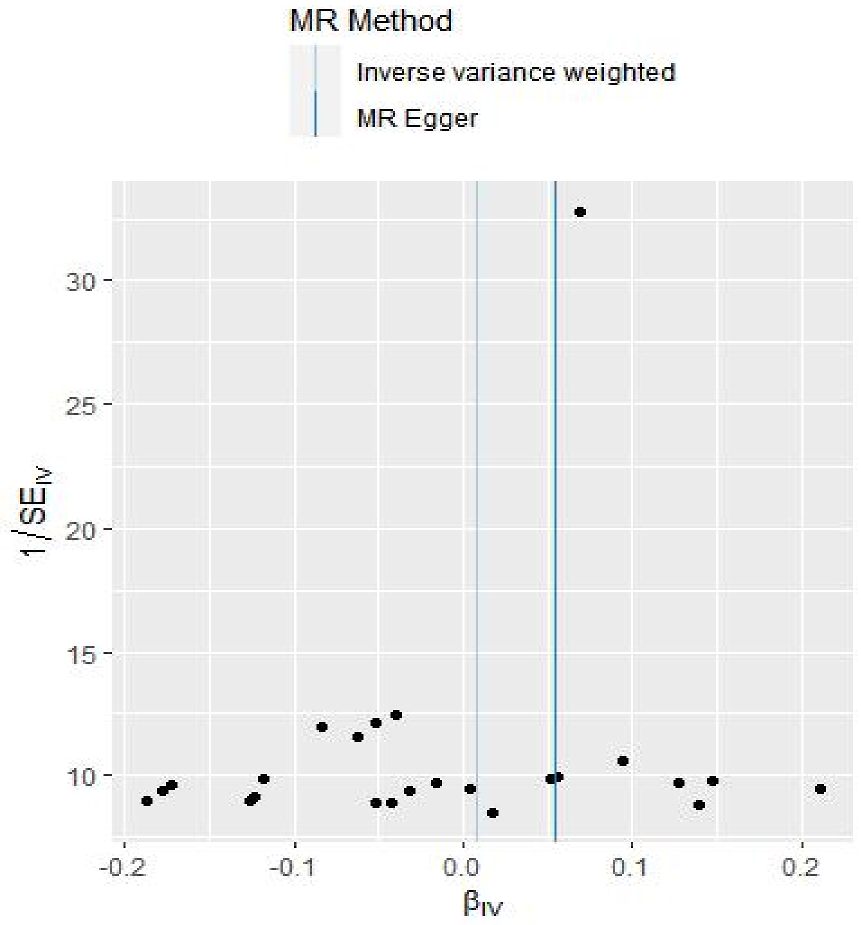

Figure 129: Leave-one-out plot to visualize causal effect of glutamate on the risk of chronic kidney disease when leaving one SNP out.

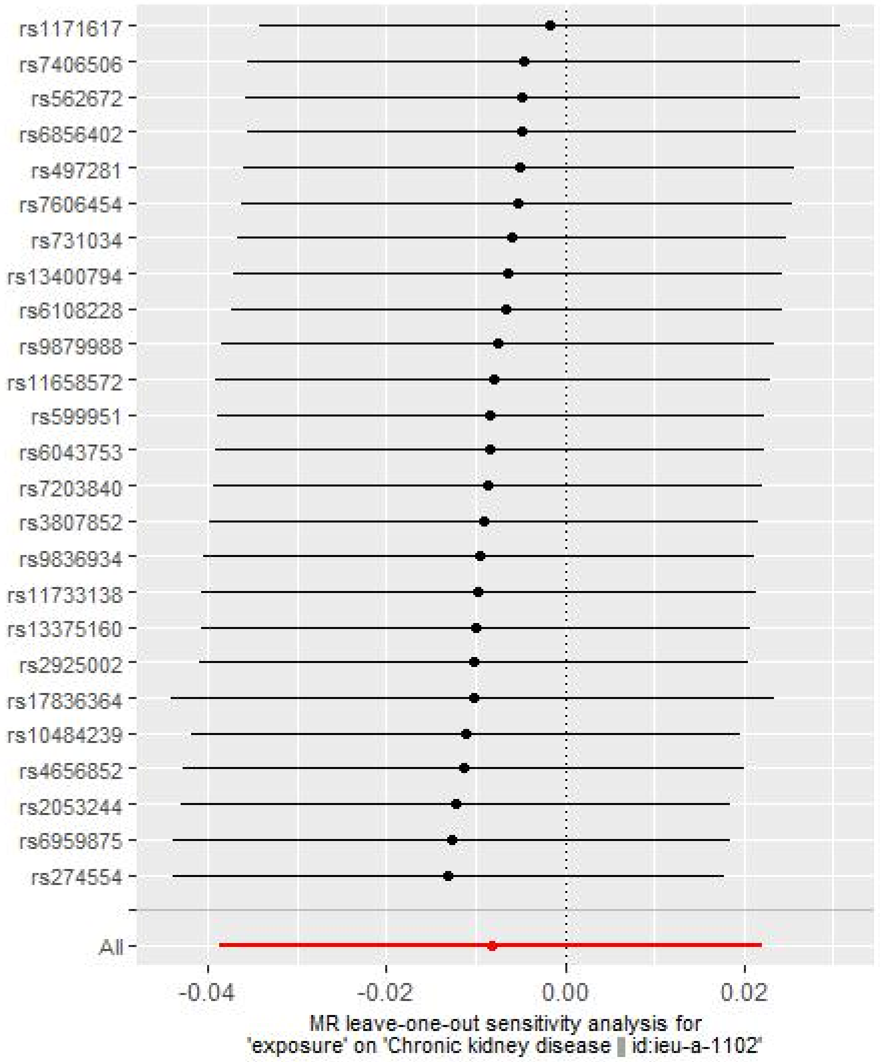

Figure 130: Funnel plots to visualize overall heterogeneity of Mendelian randomization (MR)

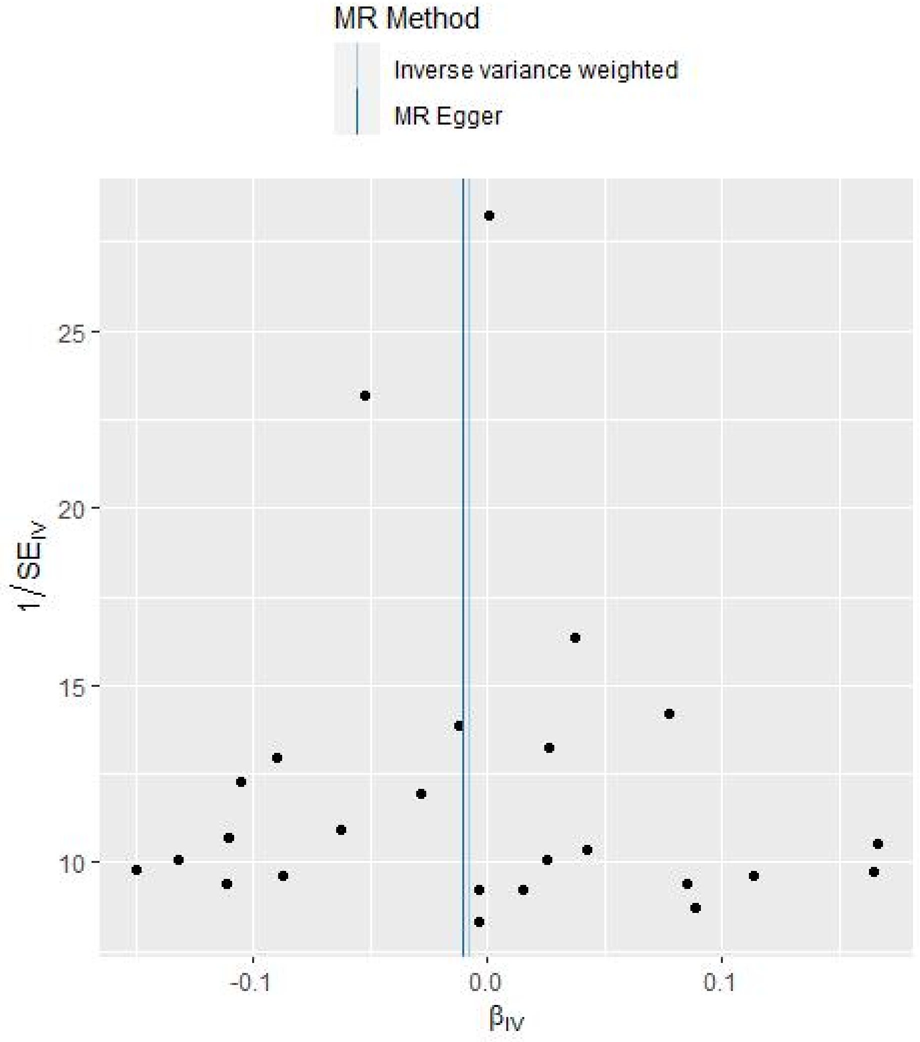

Figure 131: Leave-one-out plot to visualize causal effect of kynuremine on the risk of chronic kidney disease when leaving one SNP out.

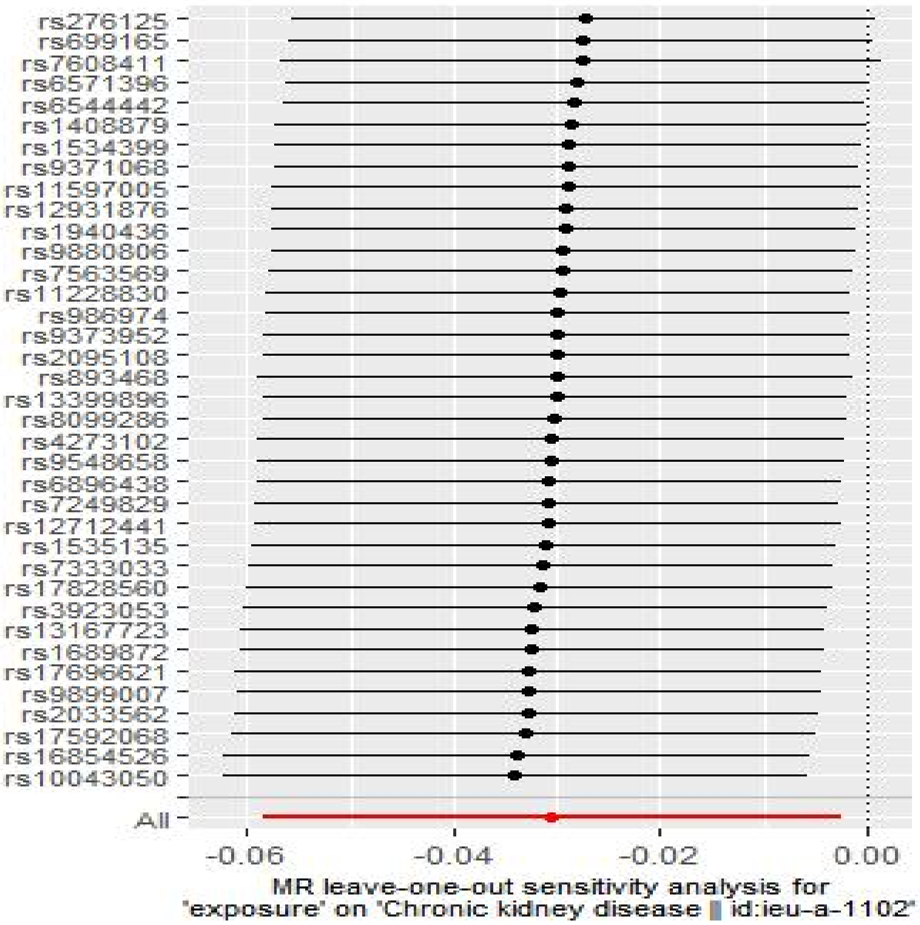

Figure 132: Funnel plots to visualize overall heterogeneity of Mendelian randomization (MR)

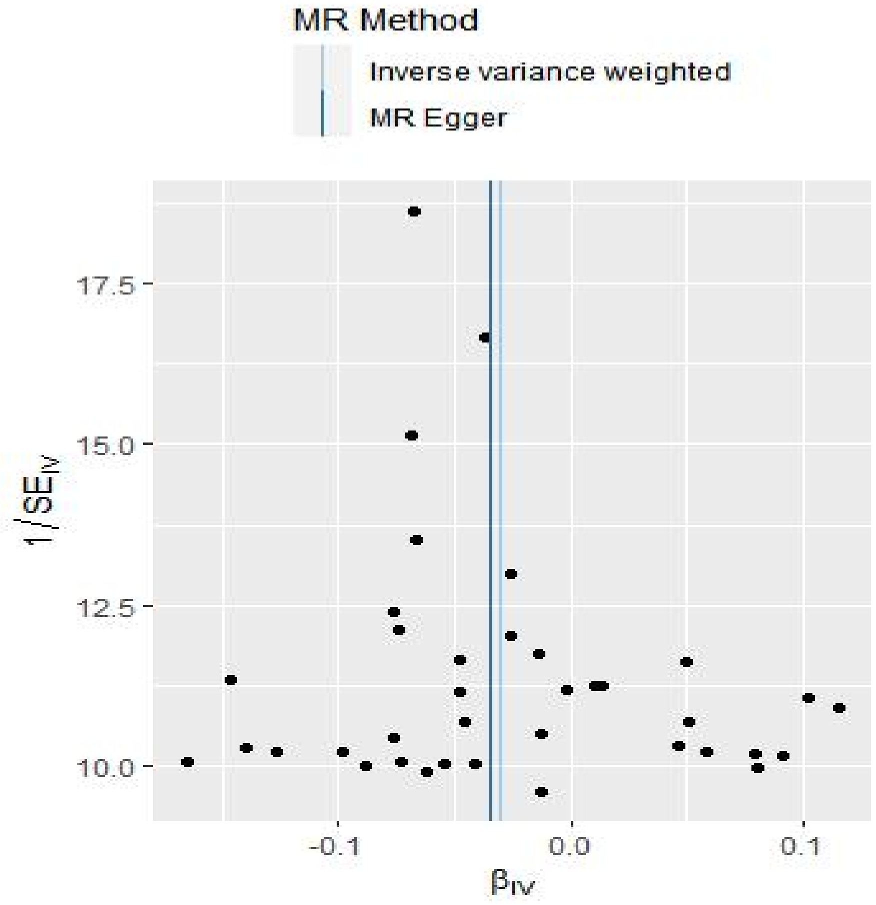

Figure 133: Leave-one-out plot to visualize causal effect of phenylalanine on the risk of chronic kidney disease when leaving one SNP out.

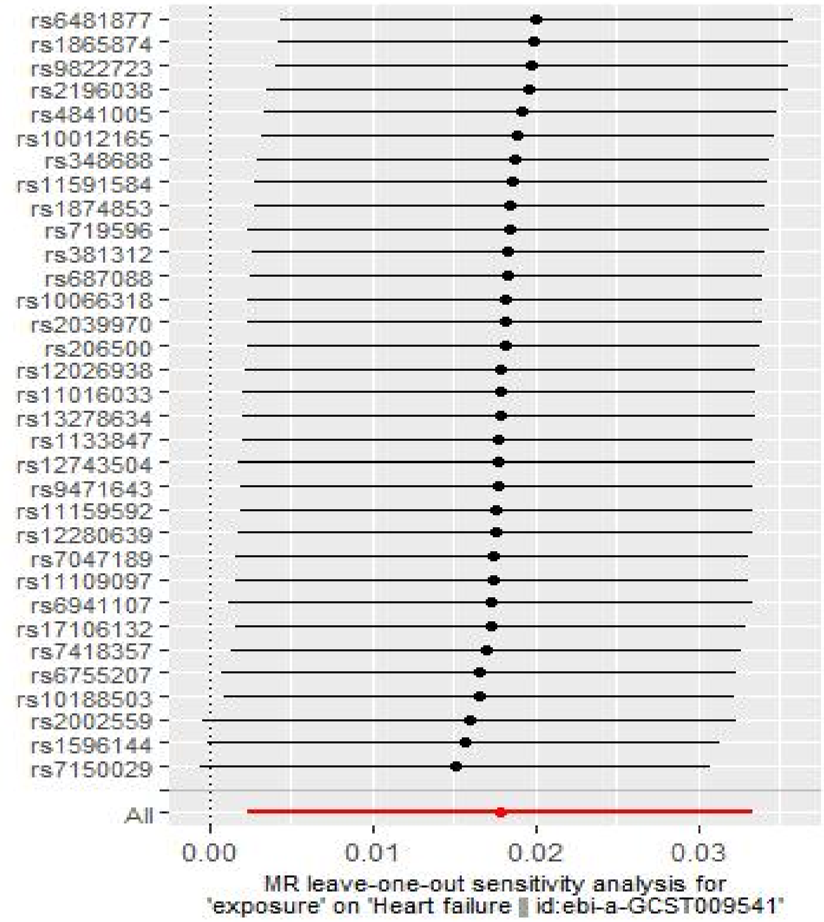

Figure 134: Funnel plots to visualize overall heterogeneity of Mendelian randomization (MR)

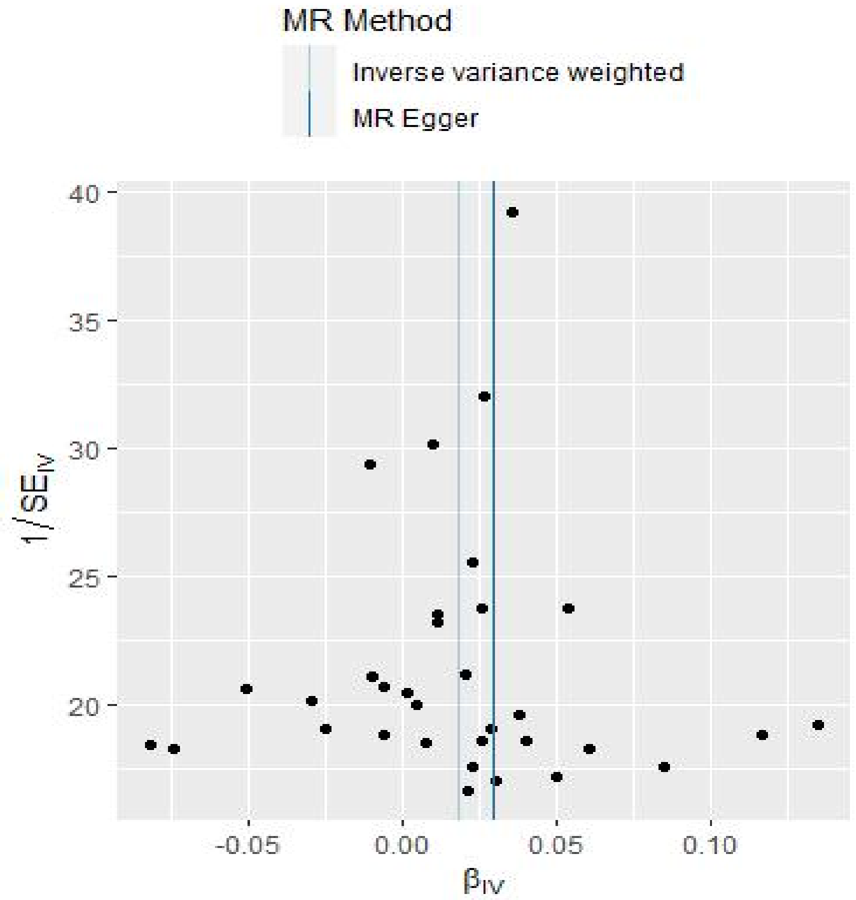

Figure 135: Leave-one-out plot to visualize causal effect of serotonin on the risk of chronic kidney disease when leaving one SNP out.

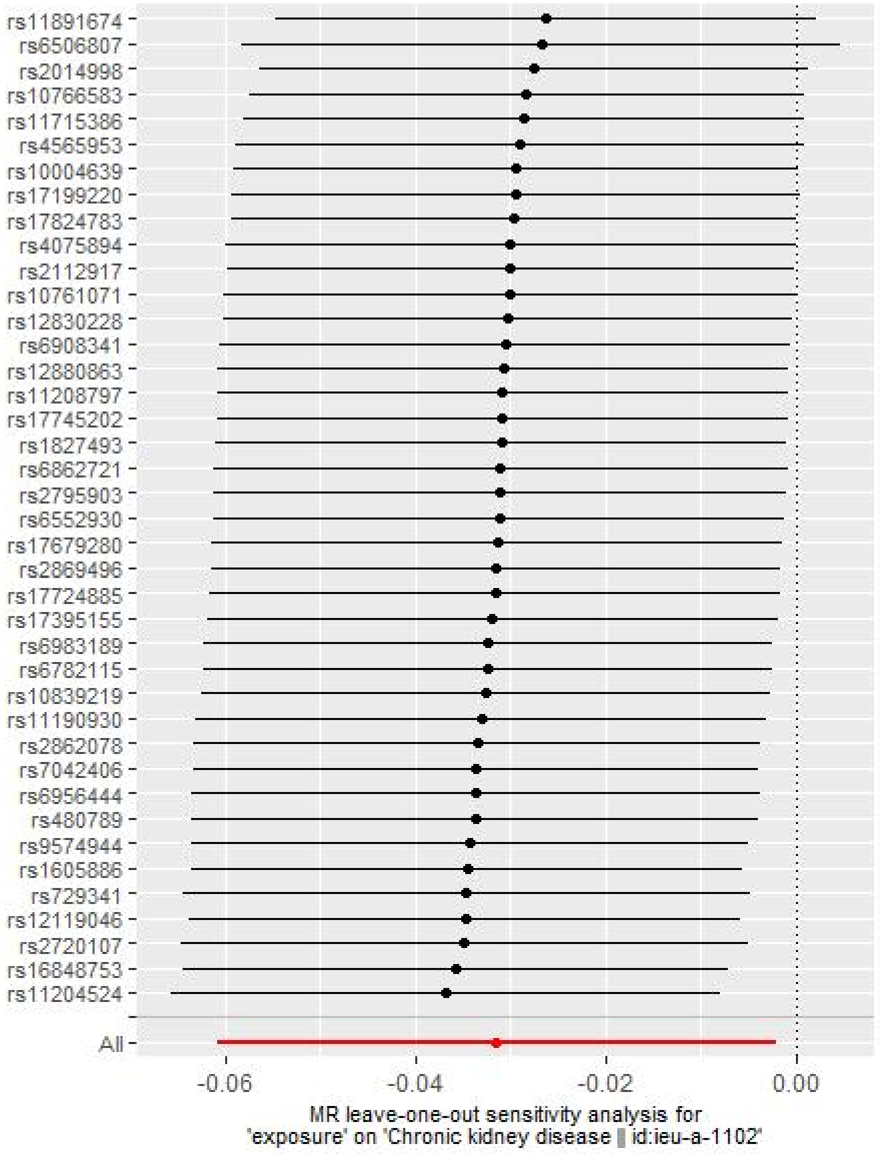

Figure 136: Funnel plots to visualize overall heterogeneity of Mendelian randomization (MR)

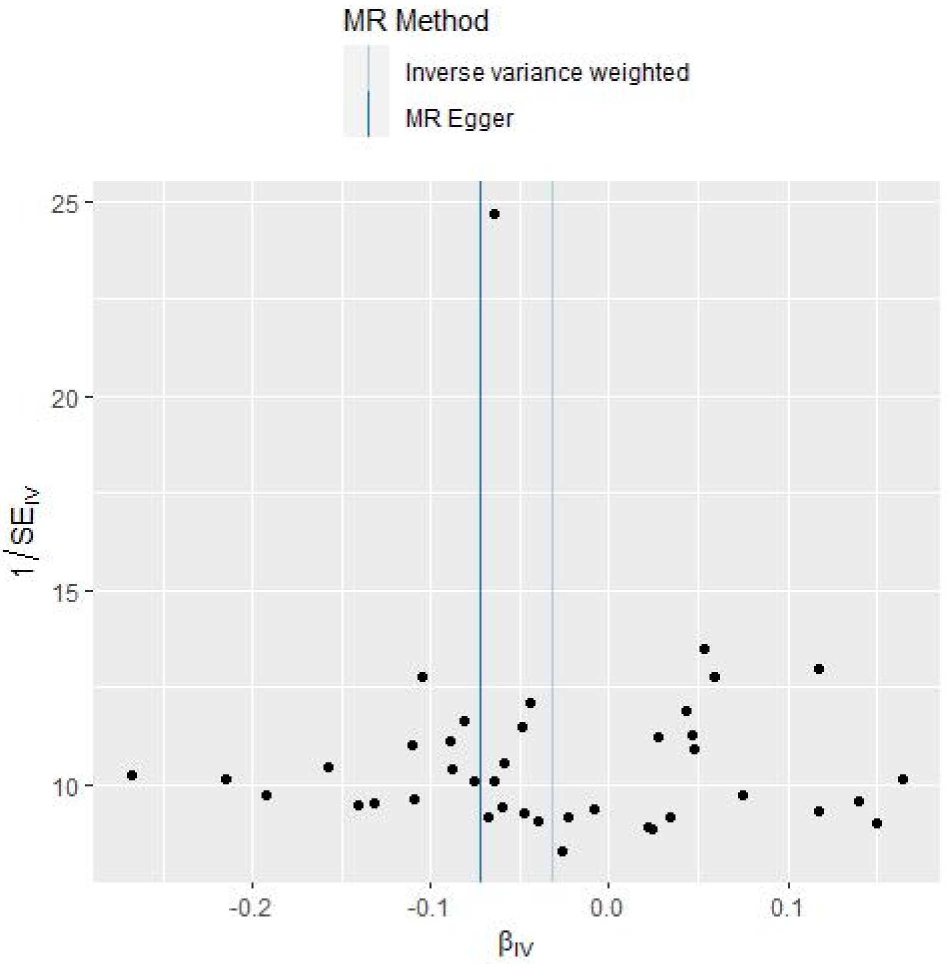

Figure 137: Leave-one-out plot to visualize causal effect of trimethylamine_N_oxide on the risk of chronic kidney disease when leaving one SNP out.

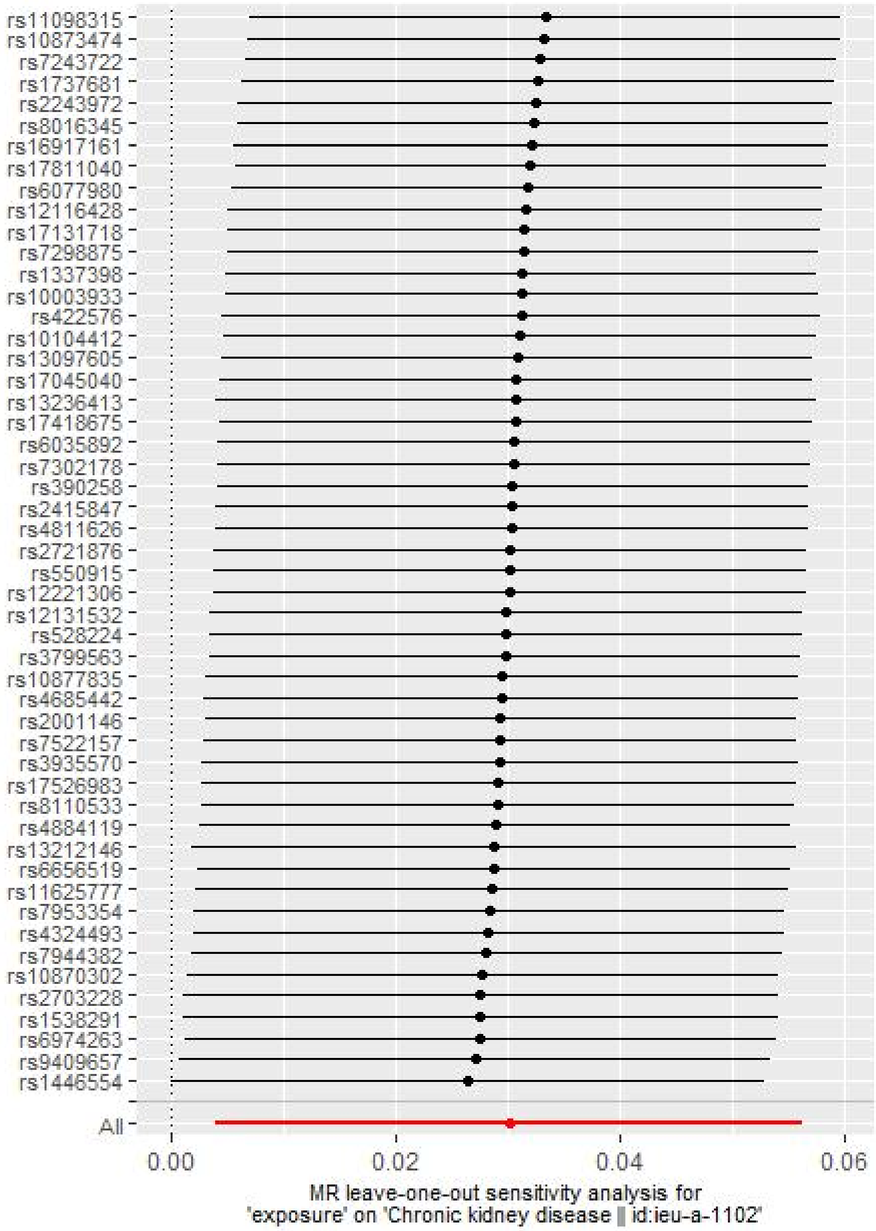

Figure 138: Funnel plots to visualize overall heterogeneity of Mendelian randomization (MR)

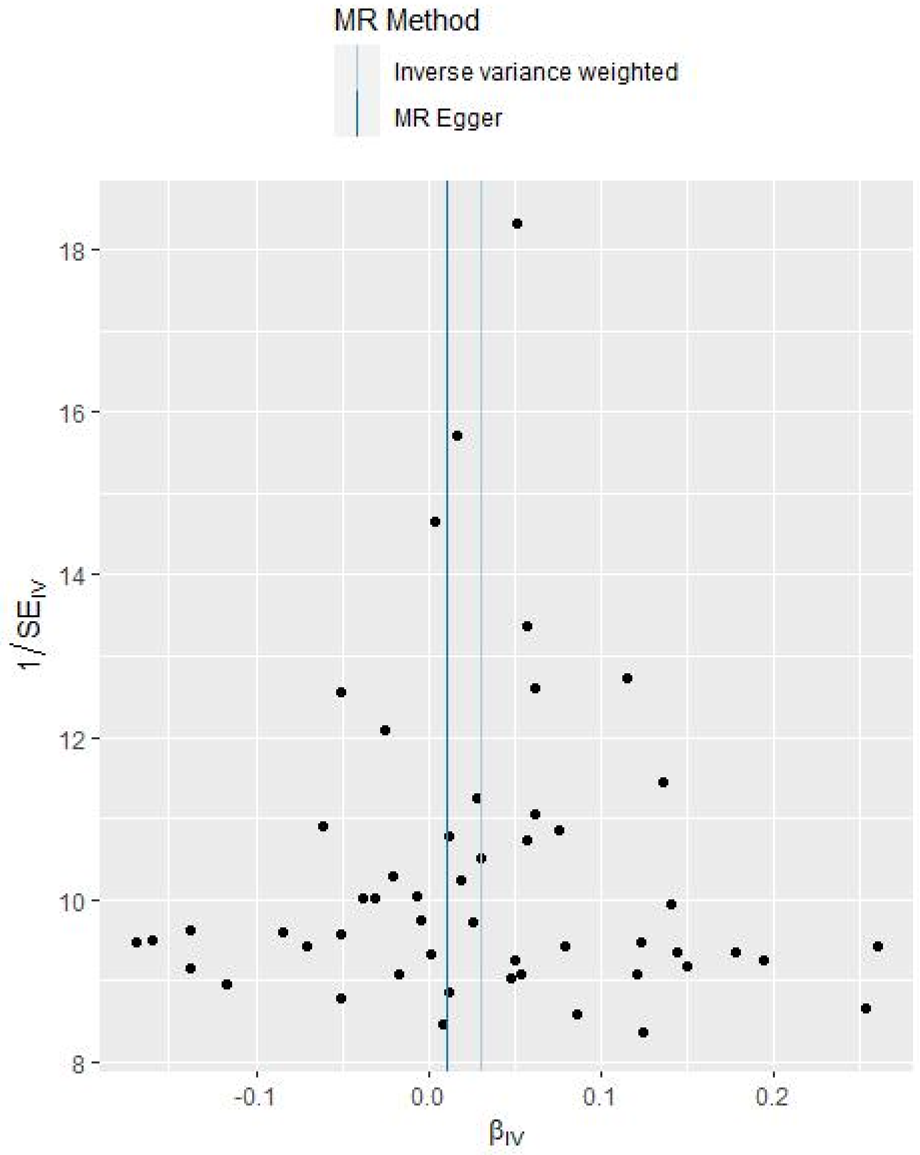

Figure 139: Leave-one-out plot to visualize causal effect of tryptophan on the risk of chronic kidney disease when leaving one SNP out.

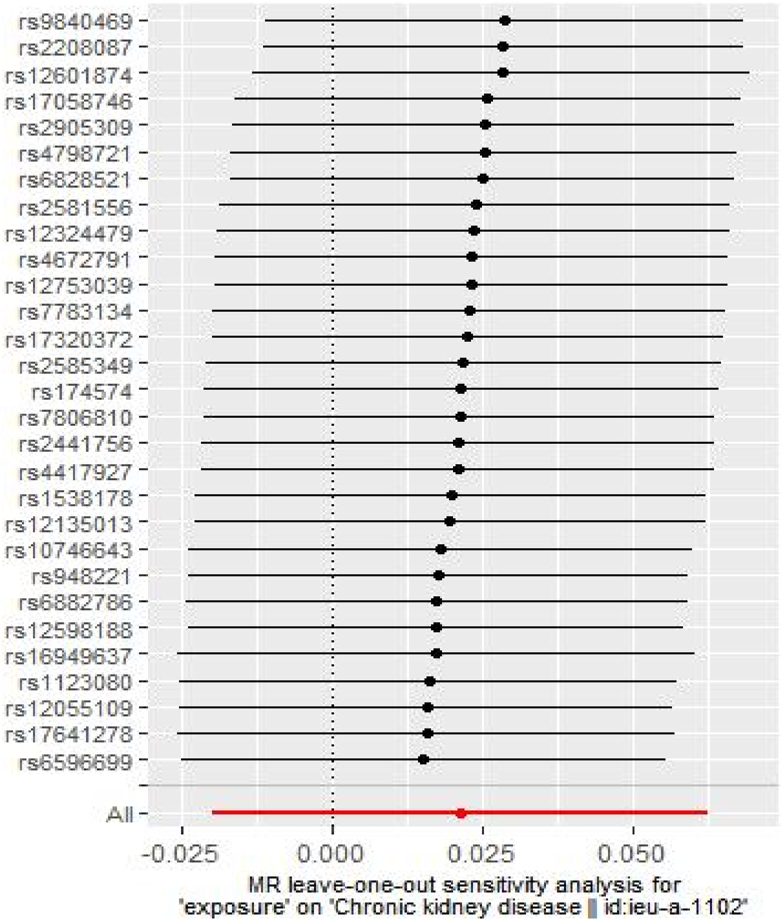

Figure 140: Funnel plots to visualize overall heterogeneity of Mendelian randomization (MR)

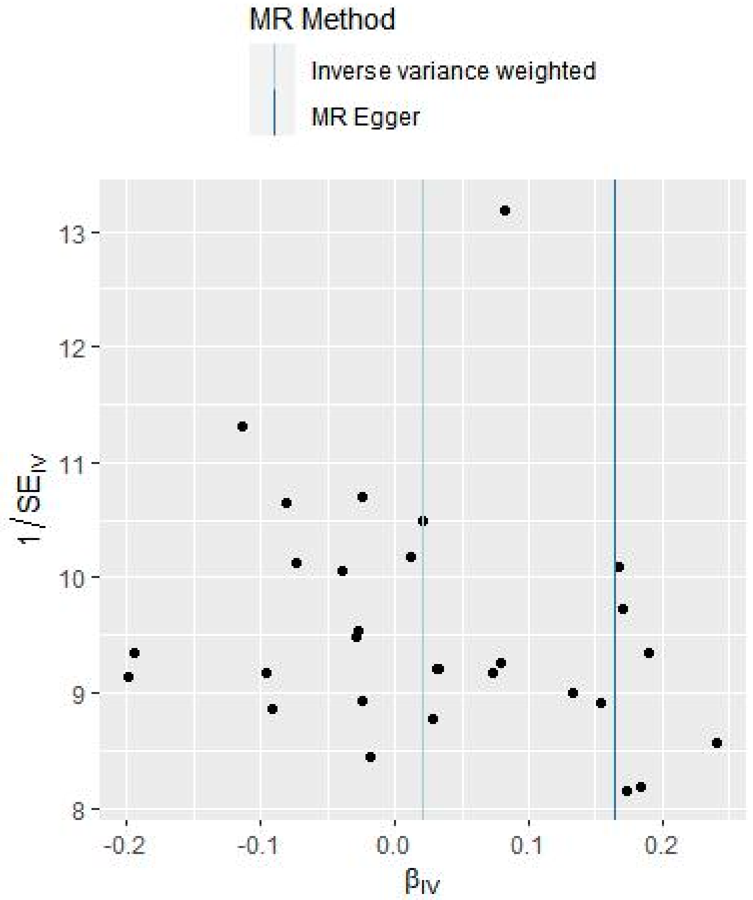

Figure 141: Leave-one-out plot to visualize causal effect of tyrosine on the risk of chronic kidney disease when leaving one SNP out.

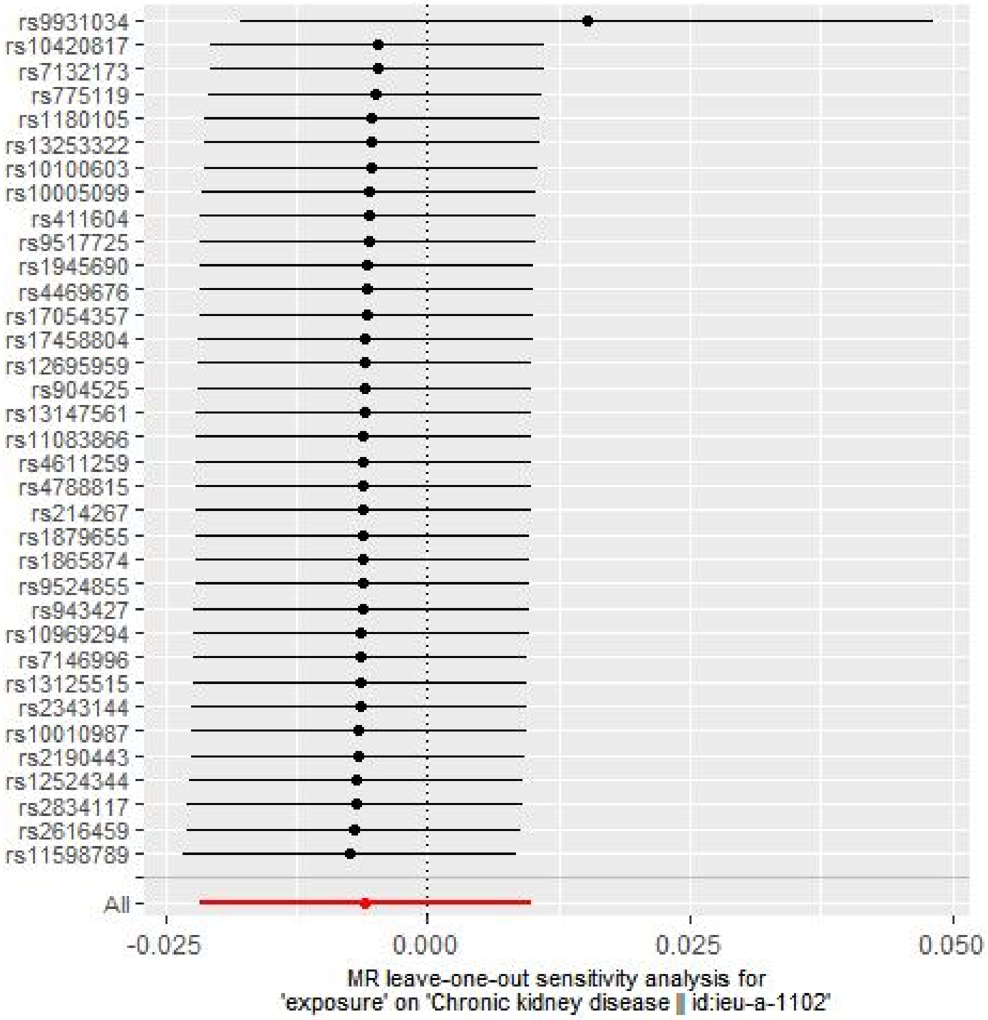

Figure 142: Funnel plots to visualize overall heterogeneity of Mendelian randomization (MR)

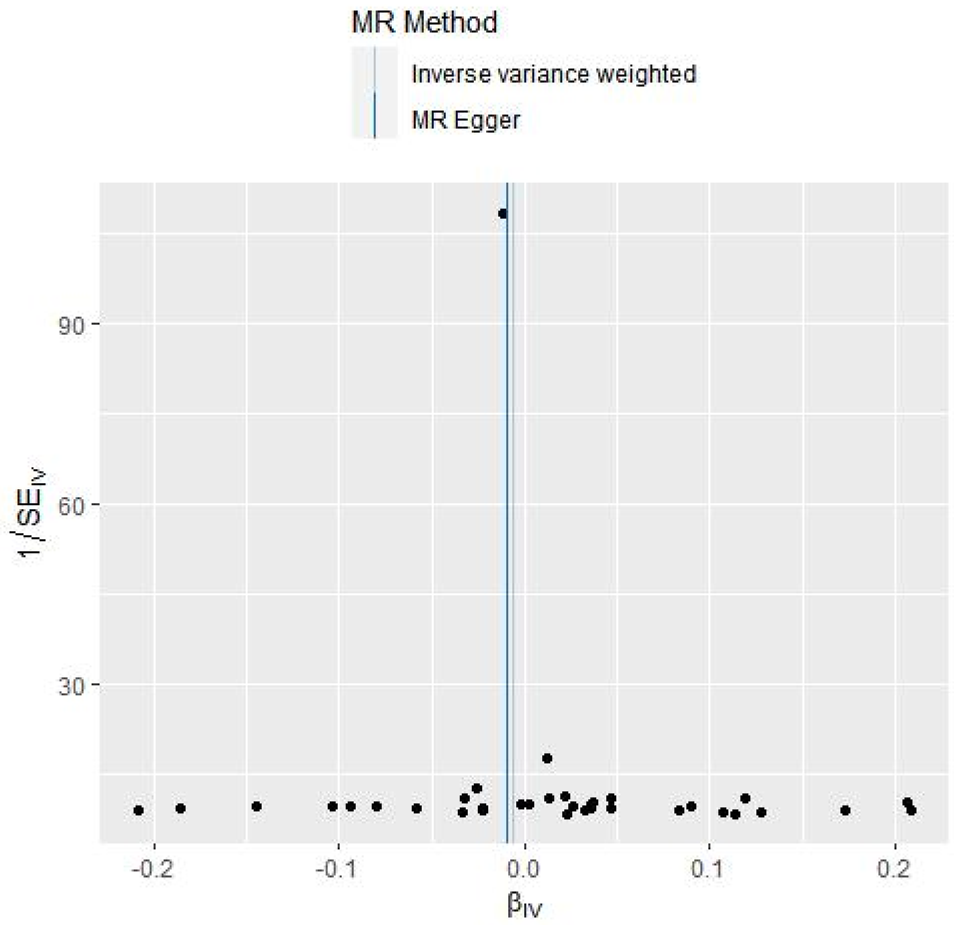

Figure 143: Leave-one-out plot to visualize causal effect of propionic acid on the risk of chronic kidney

disease when leaving one SNP out.

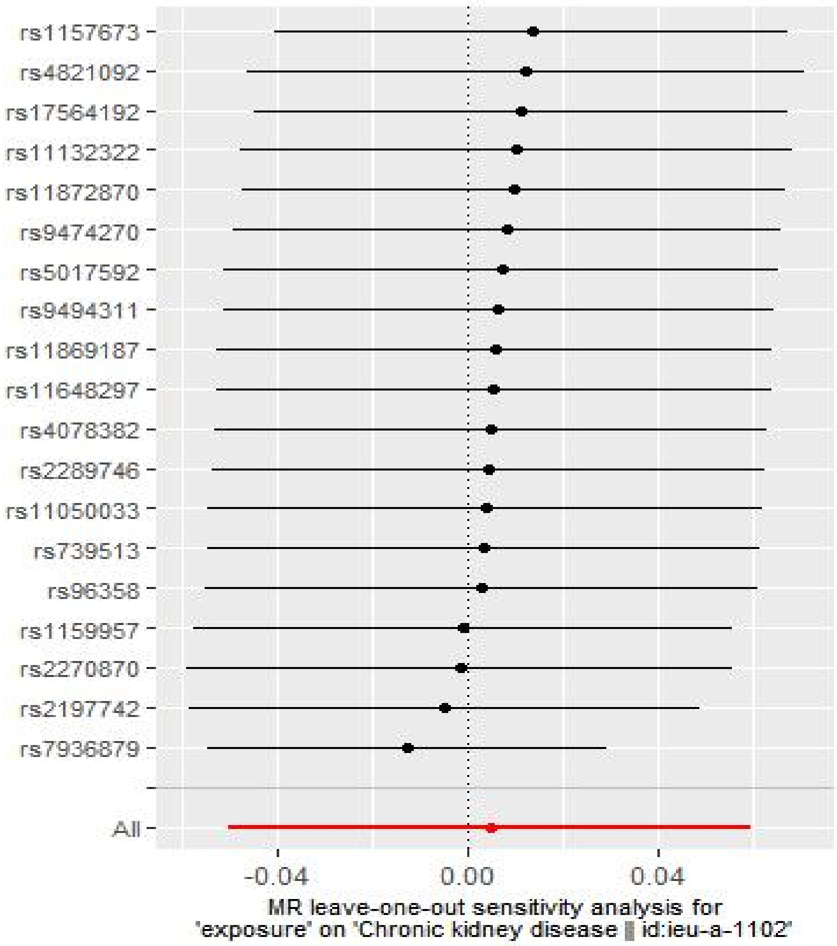

Figure 144: Funnel plots to visualize overall heterogeneity of Mendelian randomization (MR)

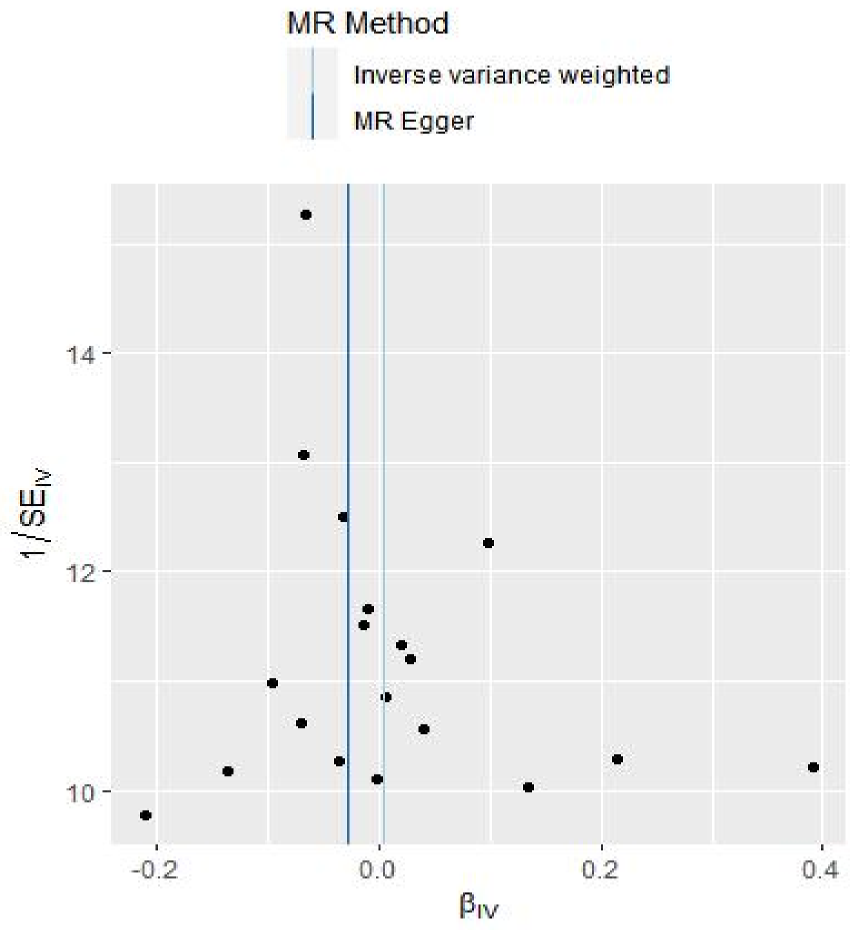

Figure 145: Leave-one-out plot to visualize causal effect of beta_hydroxybutyric acid on the risk of

systolic blood pressure when leaving one SNP out.

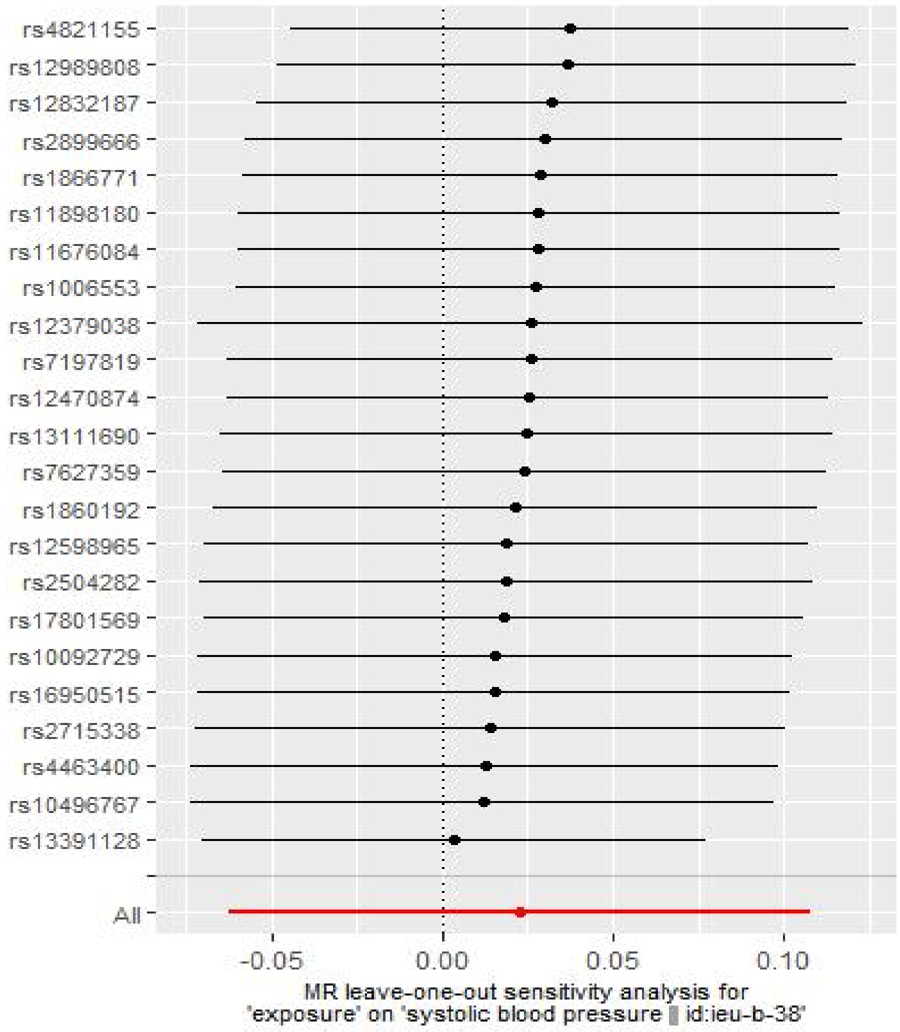

Figure 146: Funnel plots to visualize overall heterogeneity of Mendelian randomization (MR)

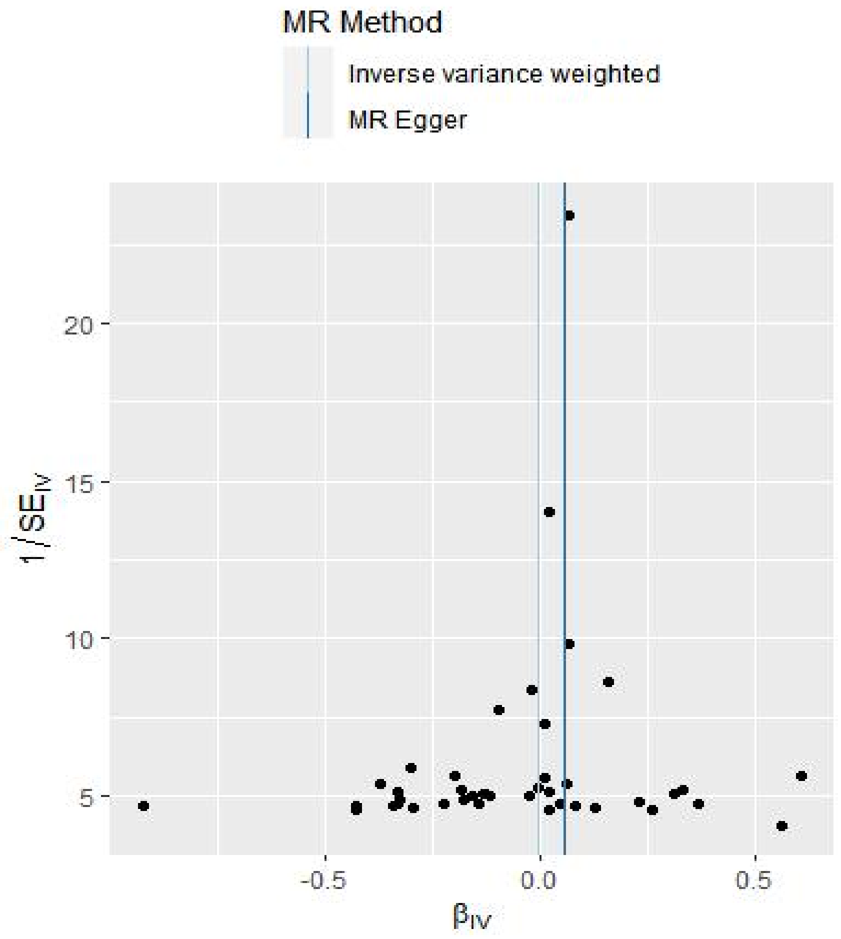

Figure 147: Leave-one-out plot to visualize causal effect of betaine on the risk of systolic blood

pressure when leaving one SNP out.

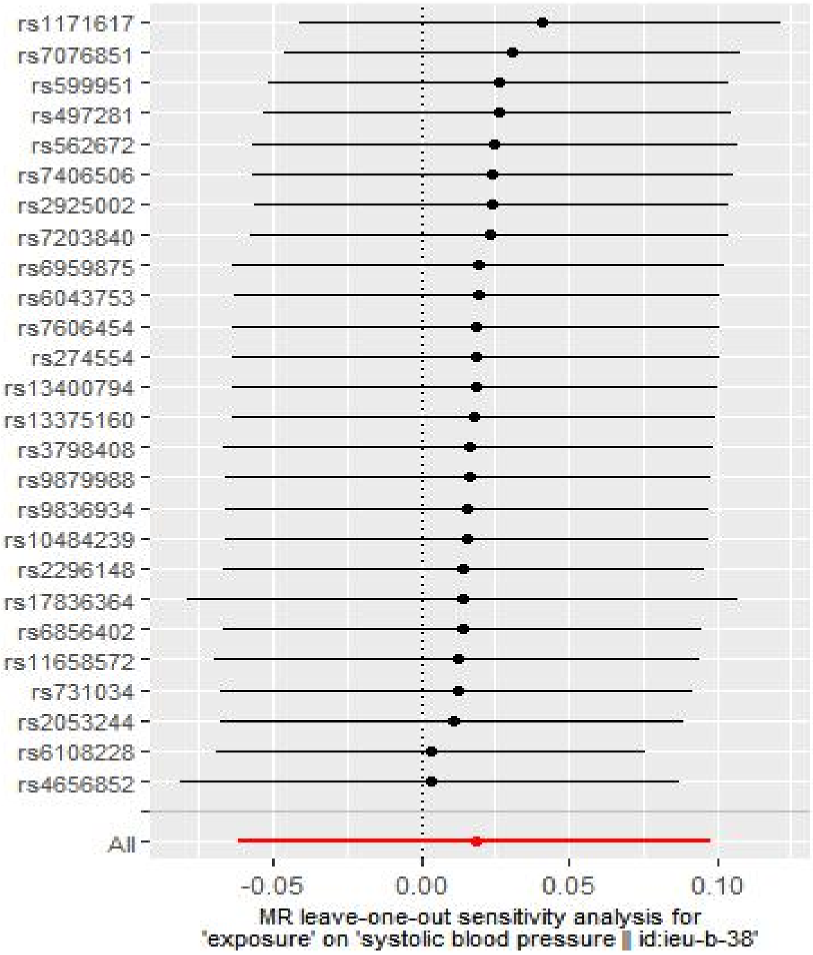

Figure 148: Funnel plots to visualize overall heterogeneity of Mendelian randomization (MR)

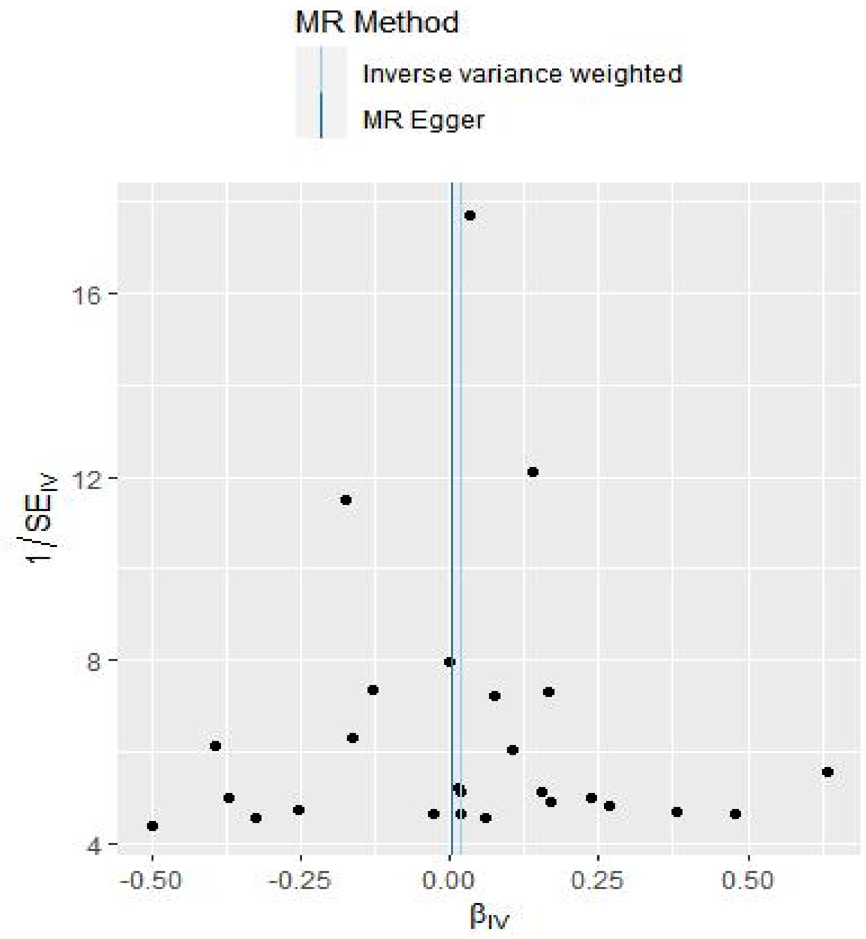

Figure 149: Leave-one-out plot to visualize causal effect of carnitine on the risk of systolic blood

pressure when leaving one SNP out.

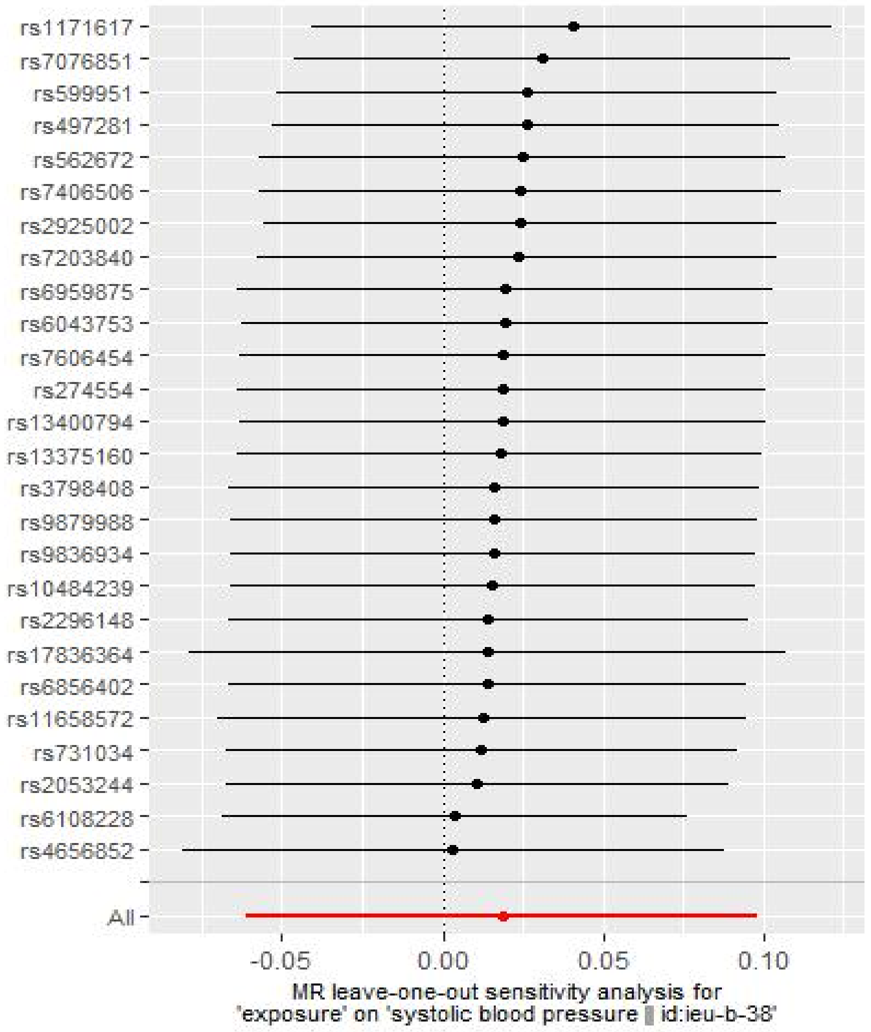

Figure 150: Funnel plots to visualize overall heterogeneity of Mendelian randomization (MR)

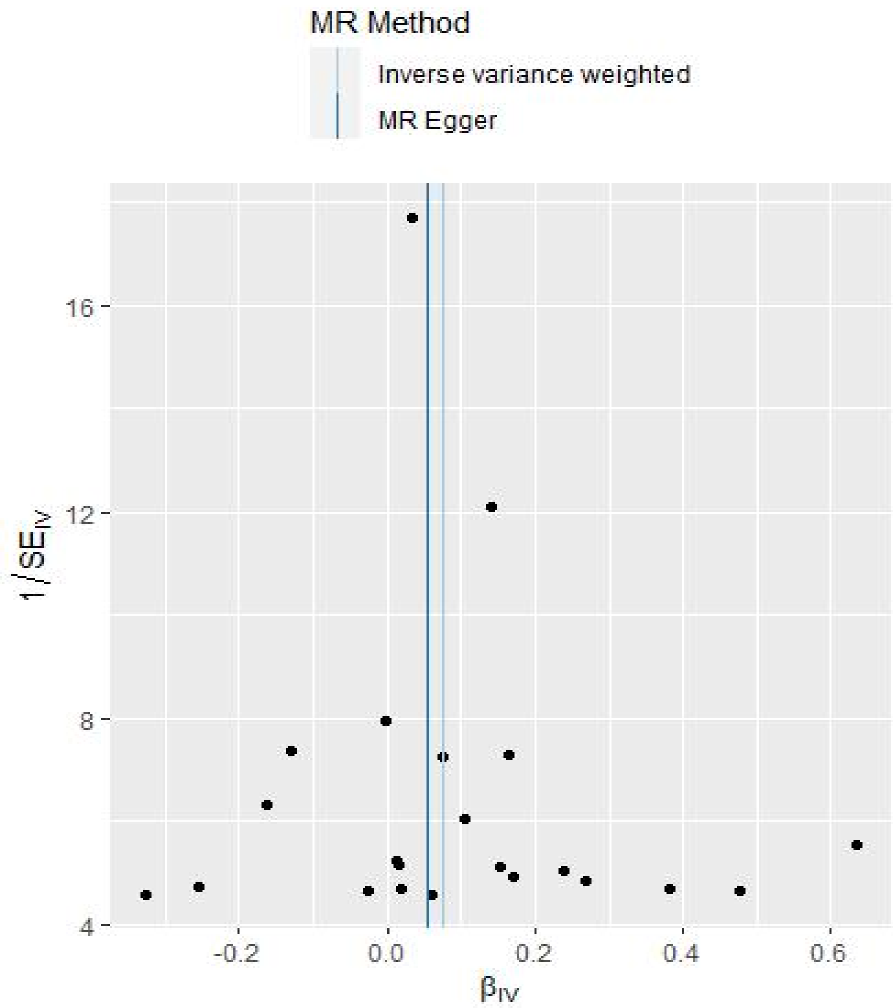

Figure 151: Leave-one-out plot to visualize causal effect of choline on the risk of systolic blood

pressure when leaving one SNP out.

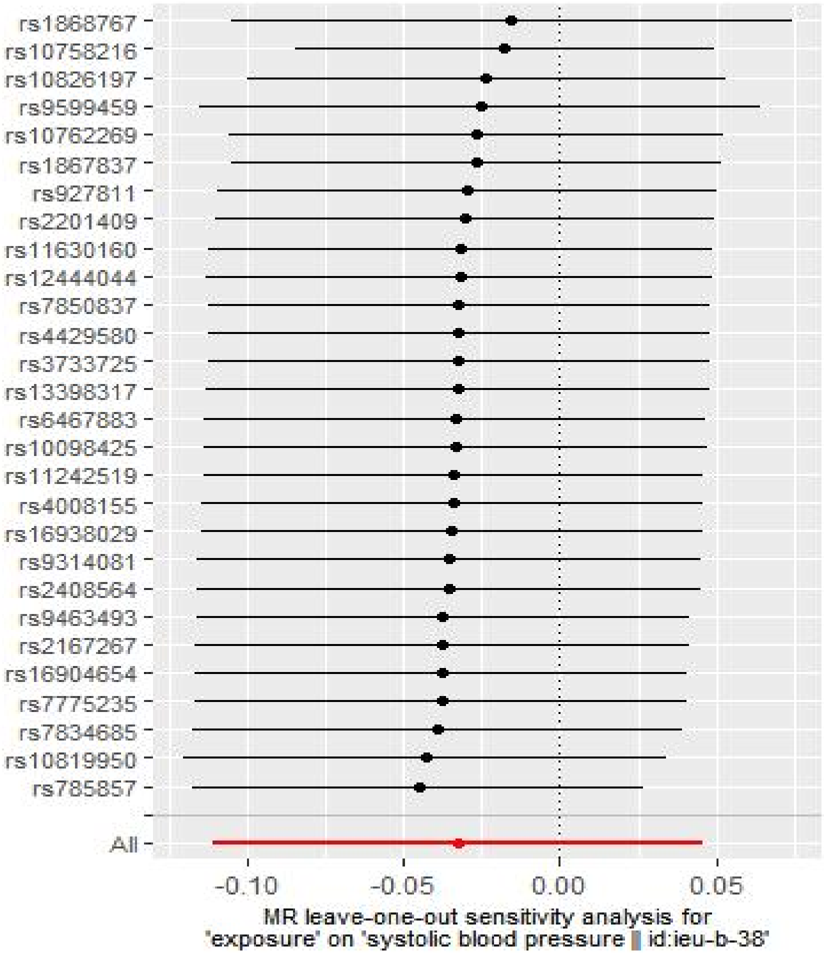

Figure 152: Funnel plots to visualize overall heterogeneity of Mendelian randomization (MR)

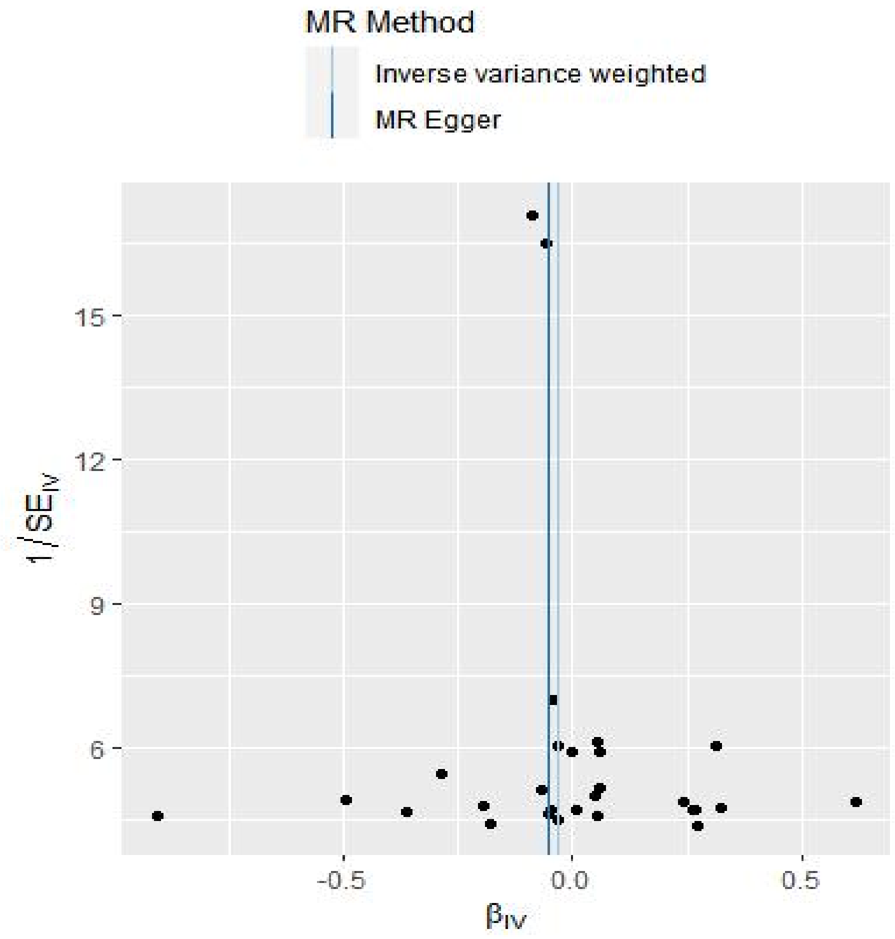

Figure 153: Leave-one-out plot to visualize causal effect of glutamate on the risk of systolic blood

pressure when leaving one SNP out.

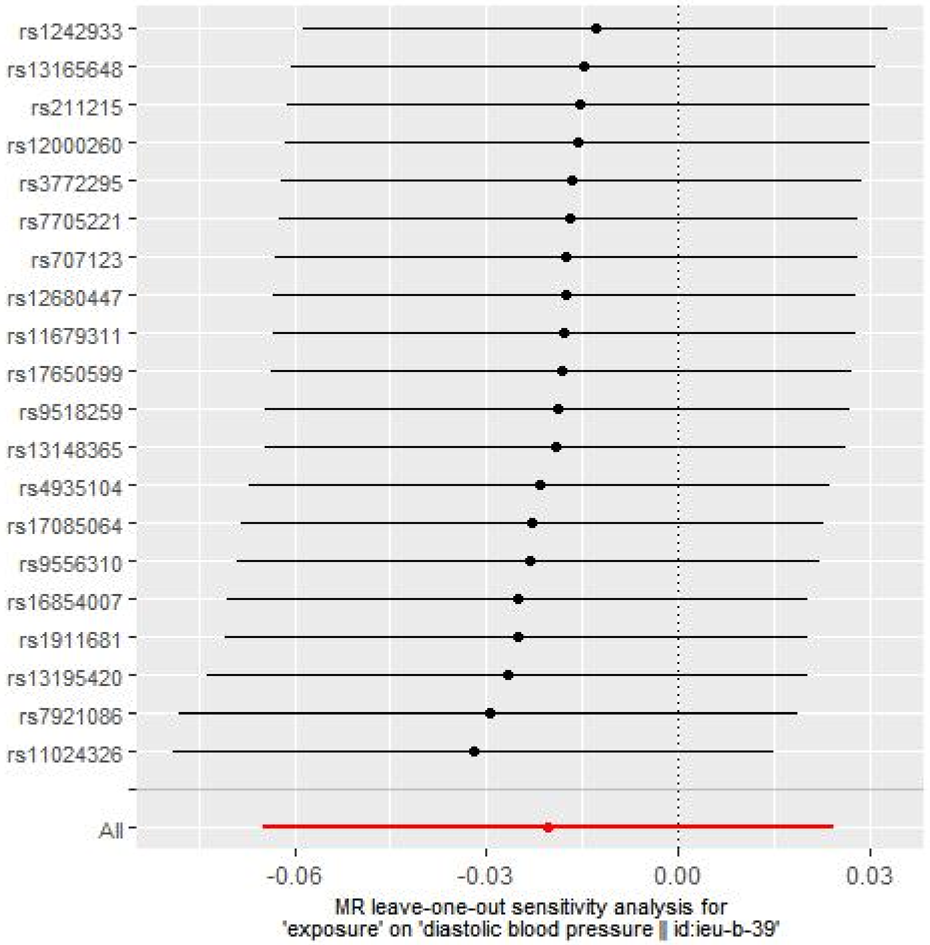

Figure 154: Funnel plots to visualize overall heterogeneity of Mendelian randomization (MR)

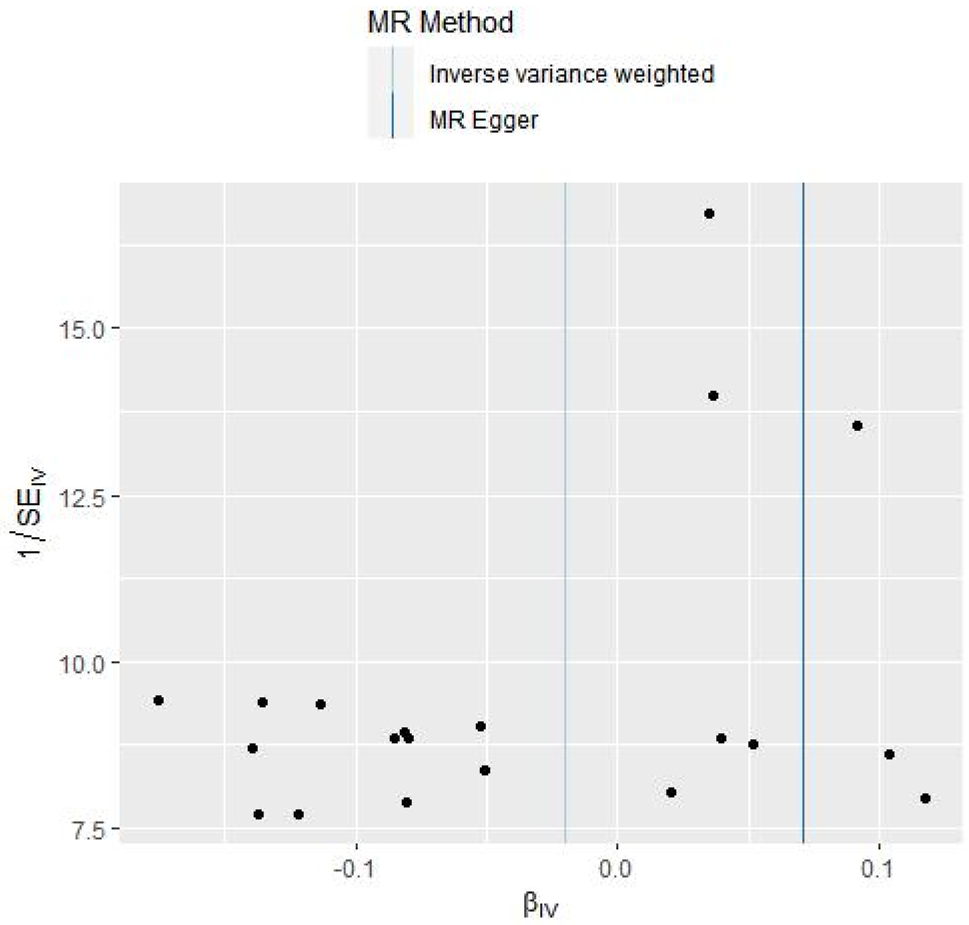

Figure 155: Leave-one-out plot to visualize causal effect of kynuremine on the risk of systolic blood

pressure when leaving one SNP out.

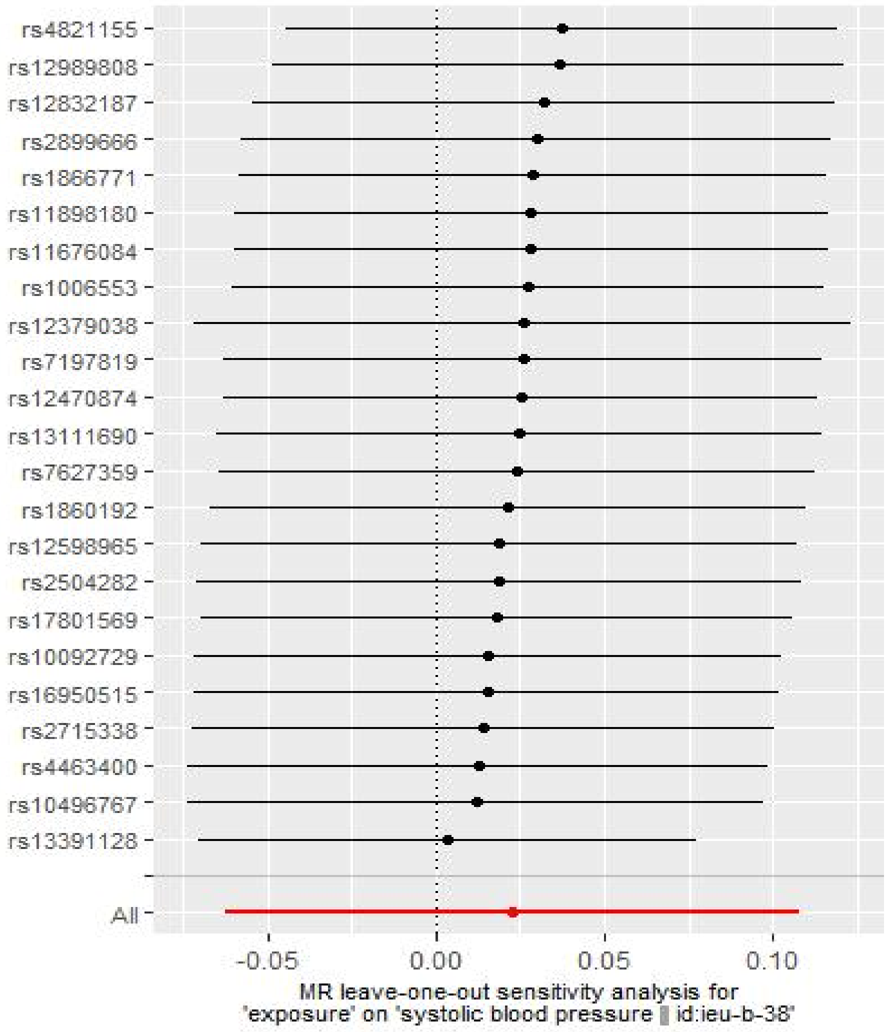

Figure 156: Funnel plots to visualize overall heterogeneity of Mendelian randomization (MR)

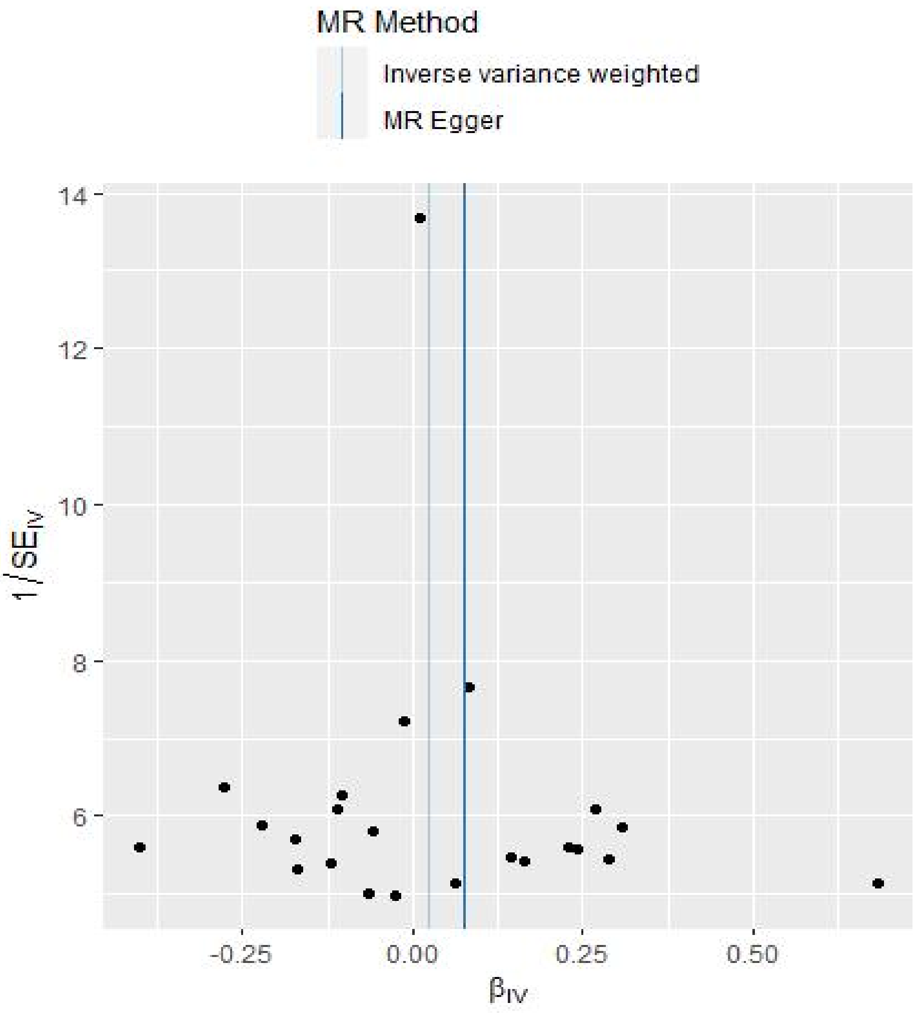

Figure 157: Leave-one-out plot to visualize causal effect of phenylalanine on the risk of systolic blood

pressure when leaving one SNP out.

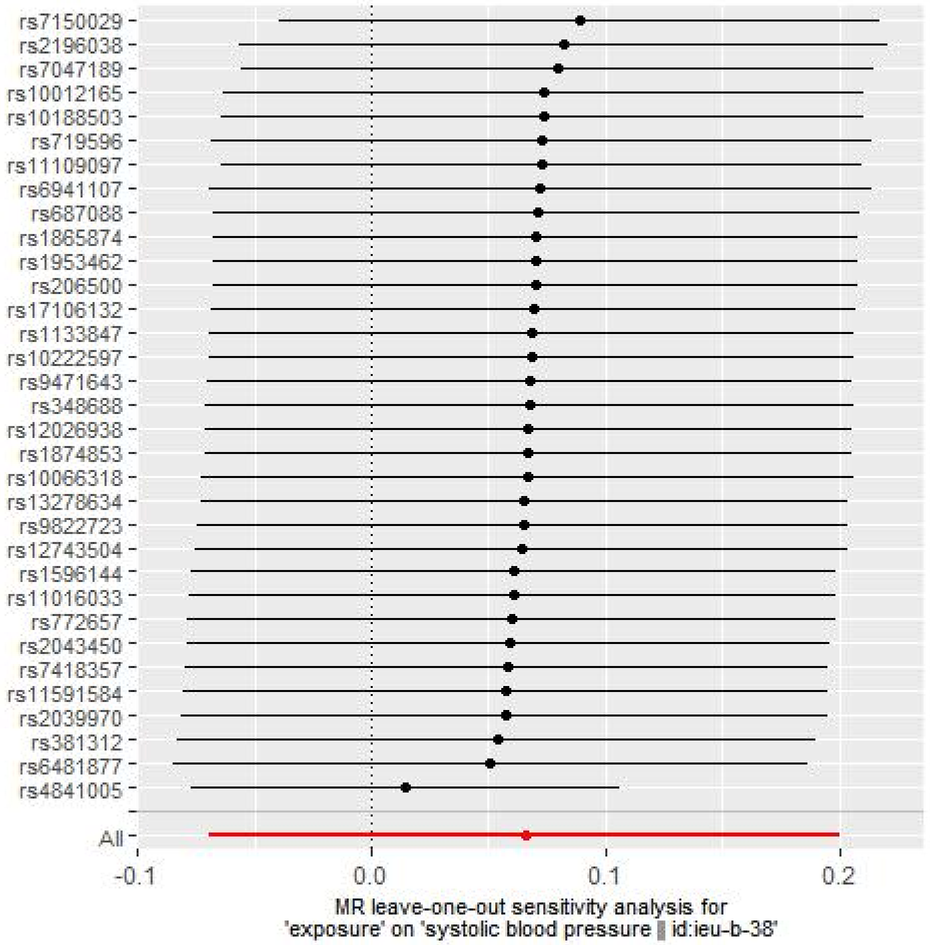

Figure 158: Funnel plots to visualize overall heterogeneity of Mendelian randomization (MR)

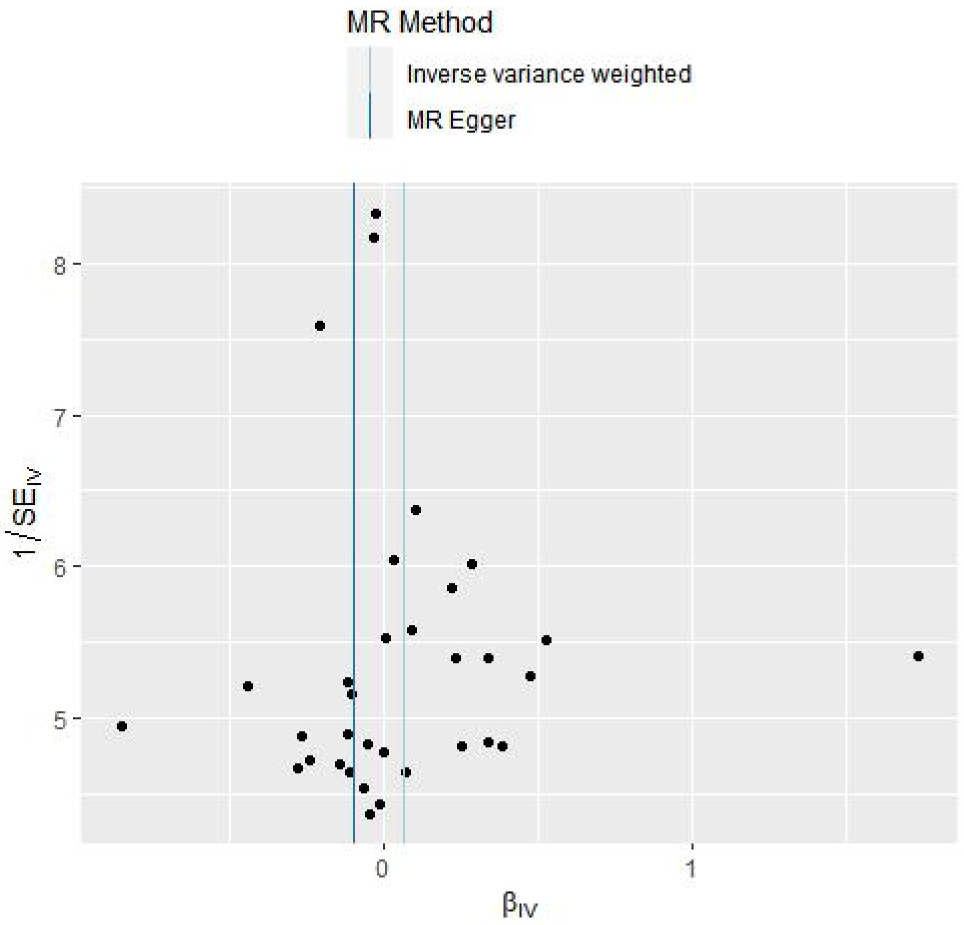

Figure 159: Leave-one-out plot to visualize causal effect of serotonin on the risk of systolic blood

pressure when leaving one SNP out.

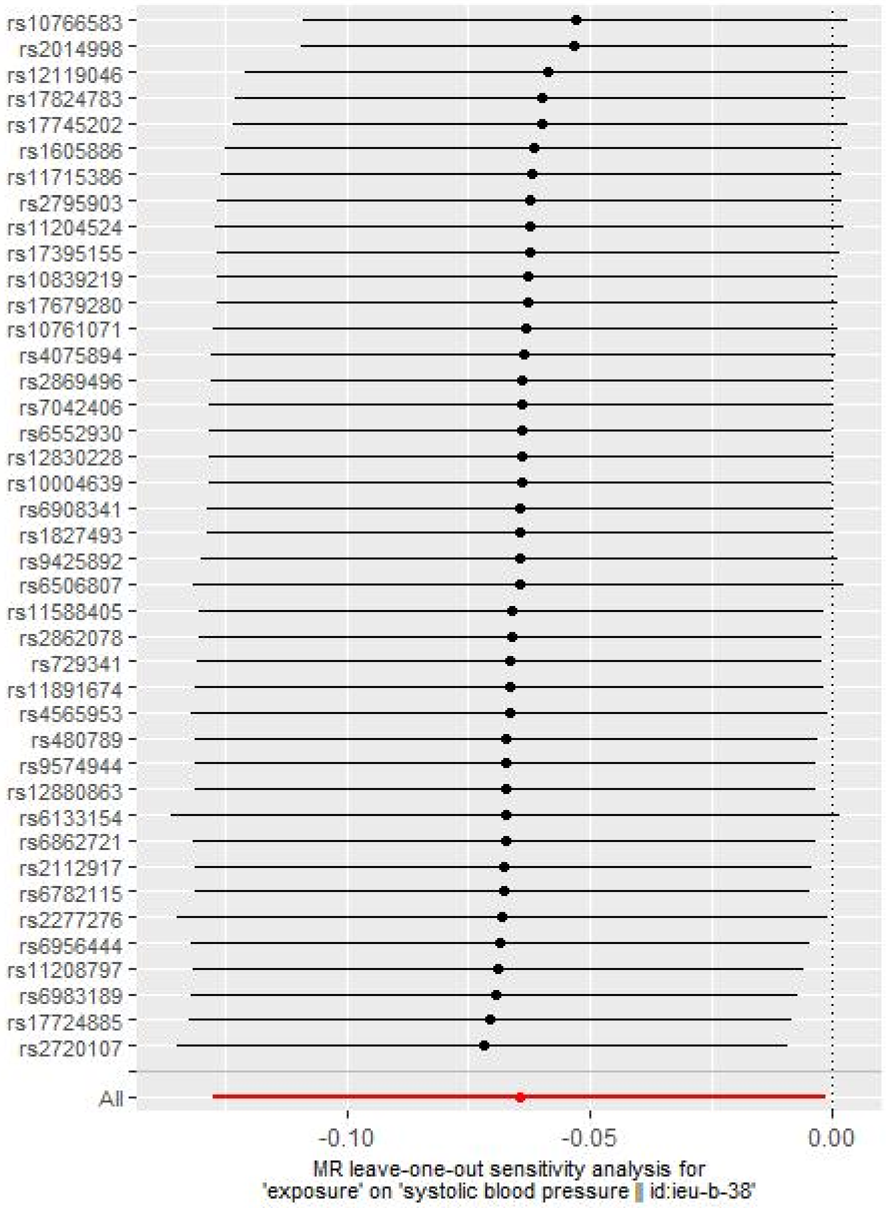

Figure 160: Funnel plots to visualize overall heterogeneity of Mendelian randomization (MR)

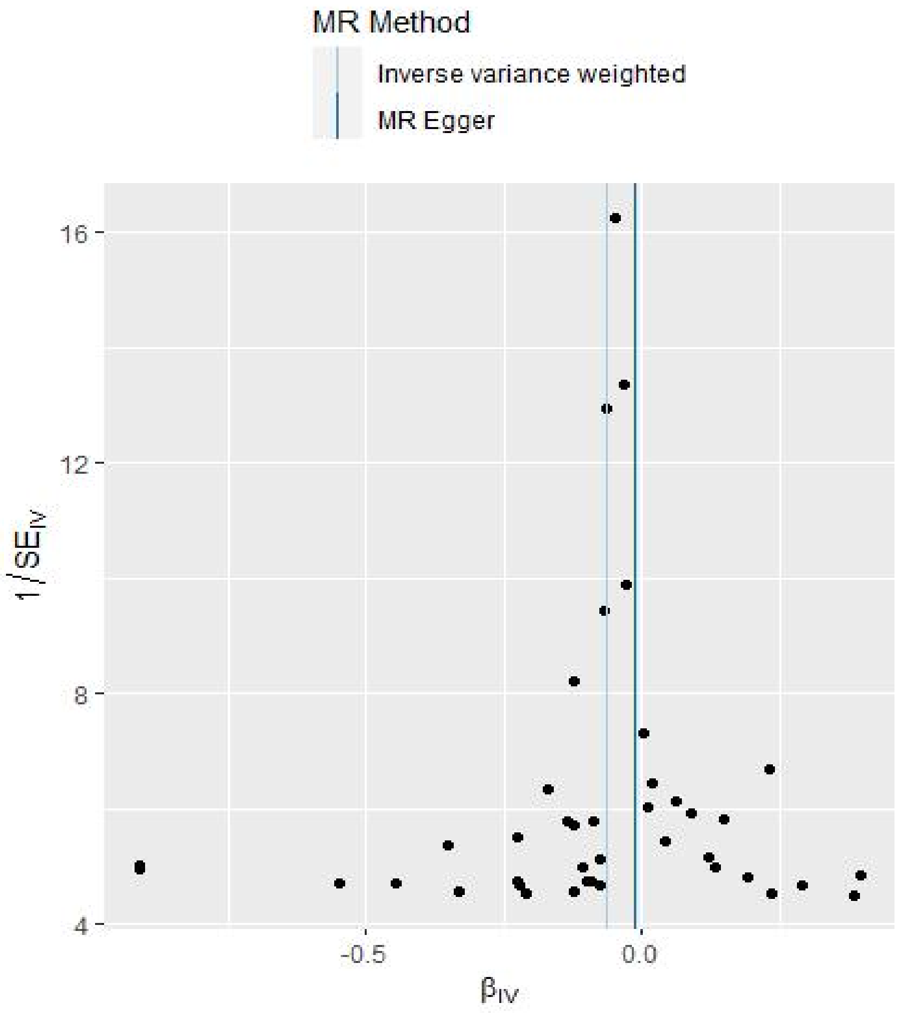

Figure 161: Leave-one-out plot to visualize causal effect of trimethylamine_N_oxide on the risk of

systolic blood pressure when leaving one SNP out.

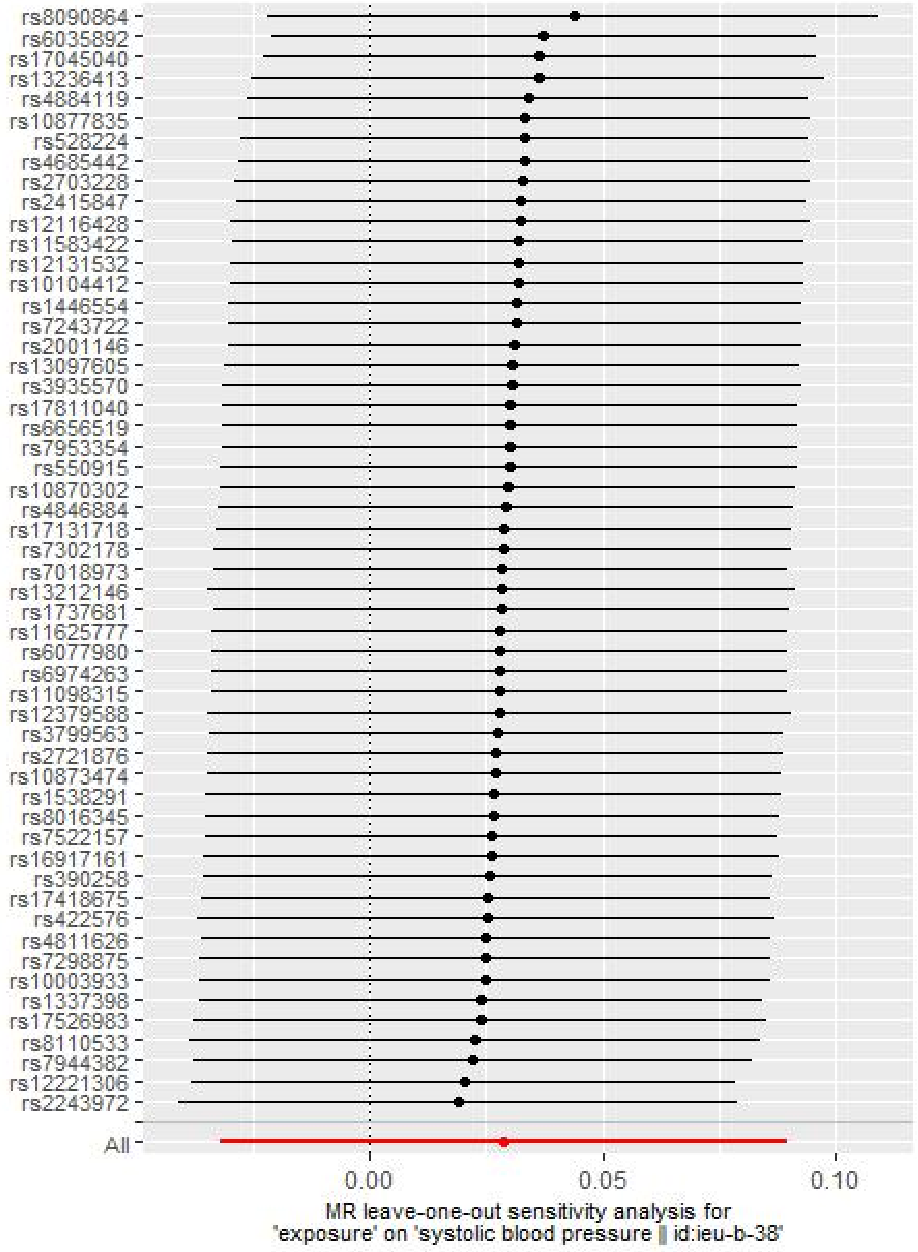

Figure 162: Funnel plots to visualize overall heterogeneity of Mendelian randomization (MR)

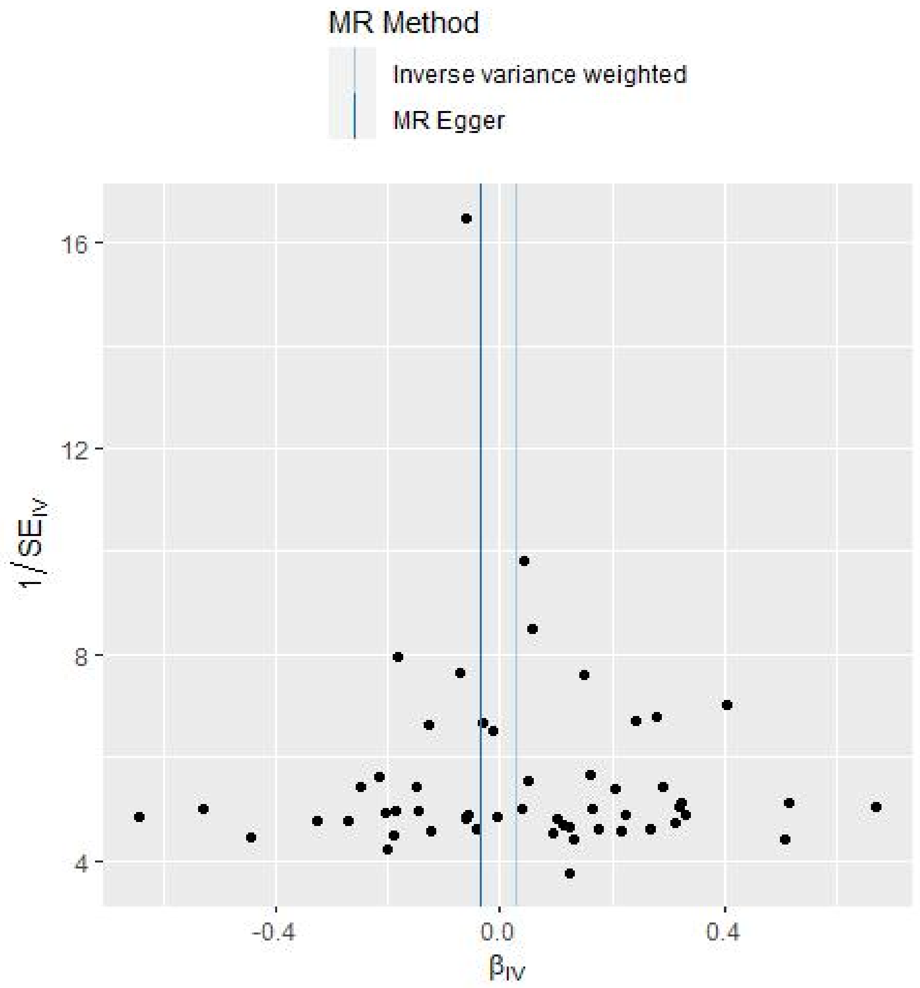

Figure 163: Leave-one-out plot to visualize causal effect of tryptophan on the risk of systolic blood

pressure when leaving one SNP out.

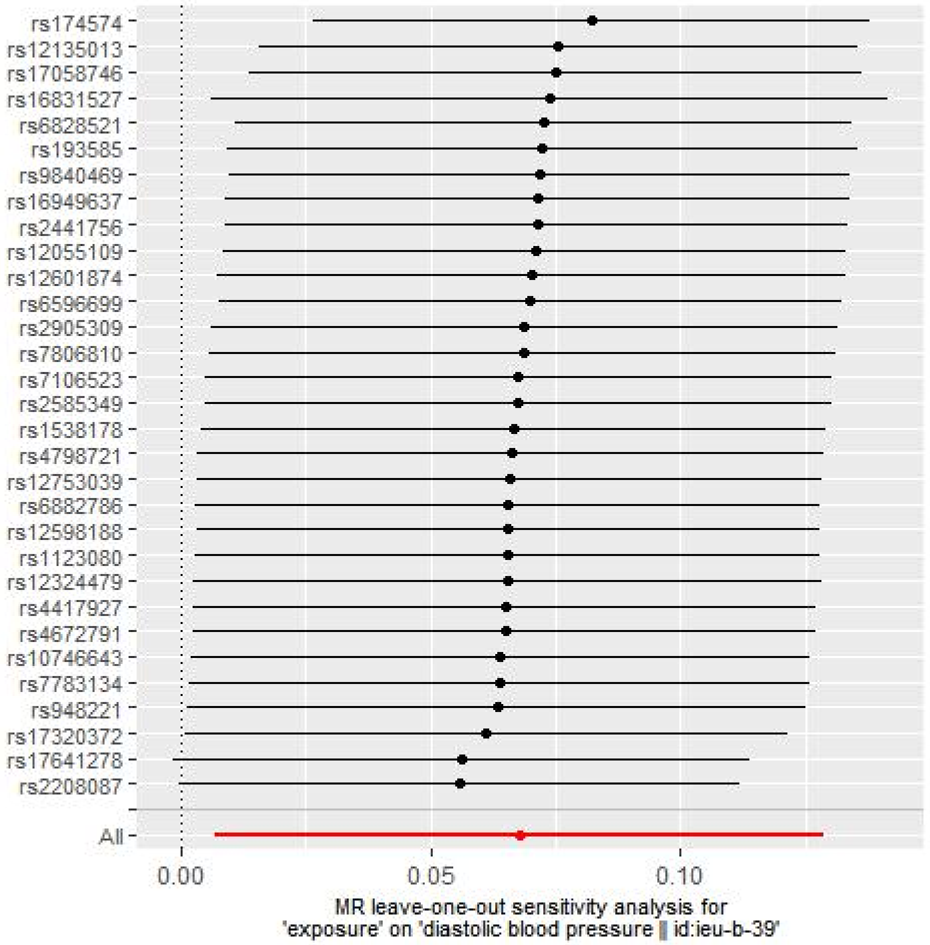

Figure 164: Funnel plots to visualize overall heterogeneity of Mendelian randomization (MR)

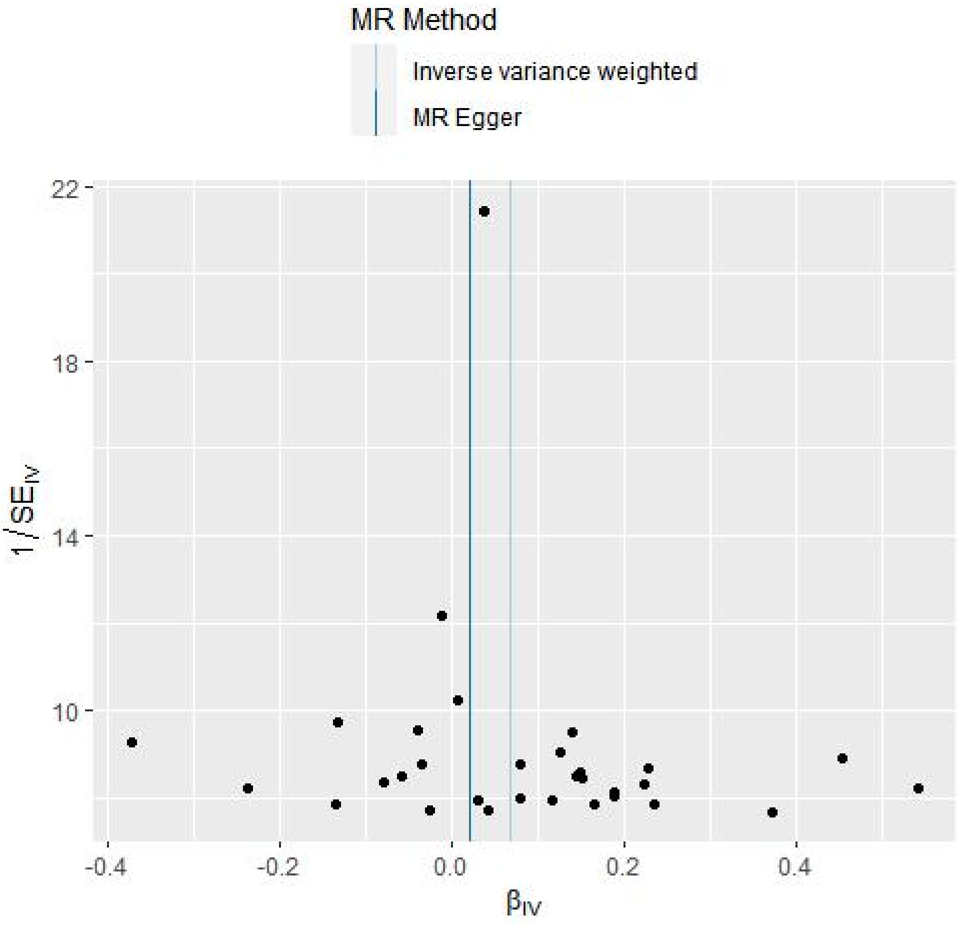

Figure 165: Leave-one-out plot to visualize causal effect of tyrosine on the risk of systolic blood

pressure when leaving one SNP out.

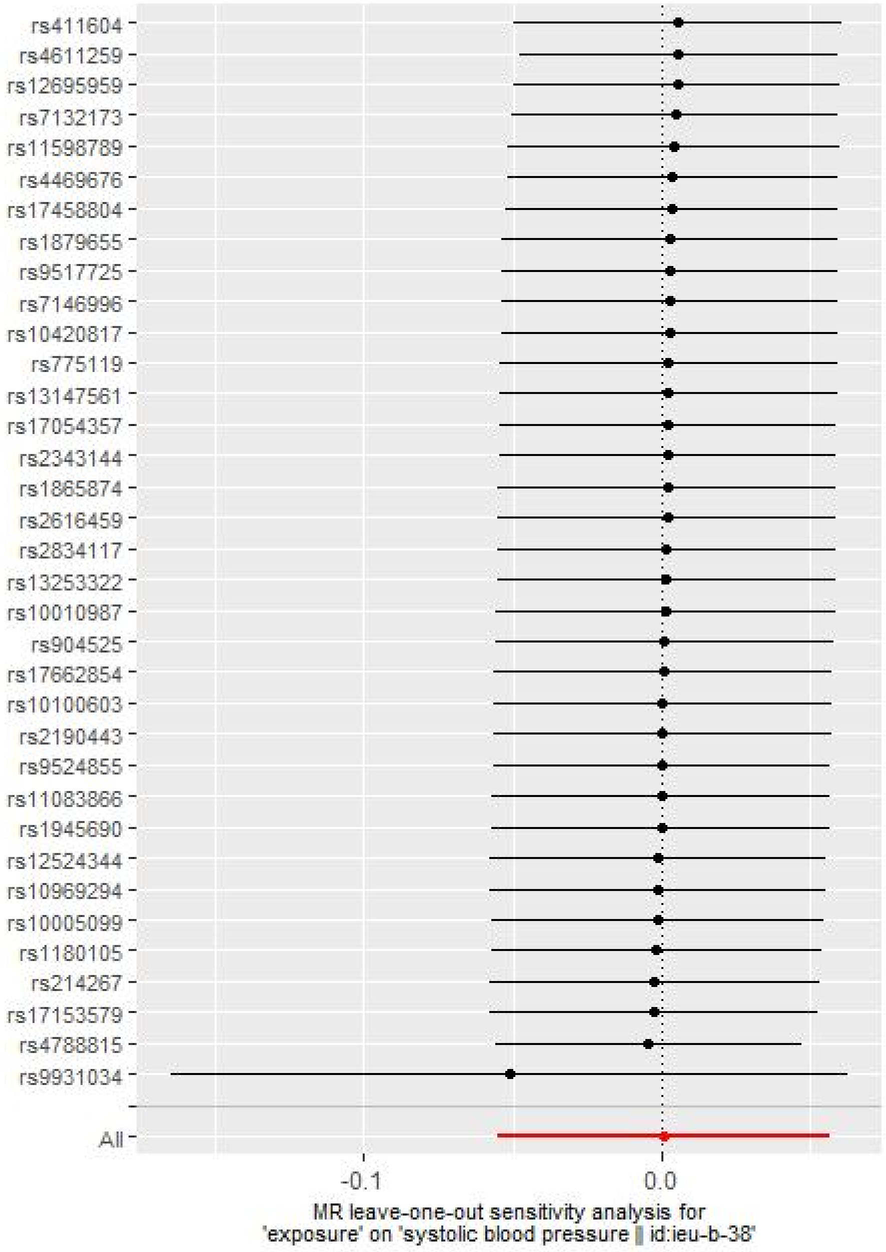

Figure 166: Funnel plots to visualize overall heterogeneity of Mendelian randomization (MR)

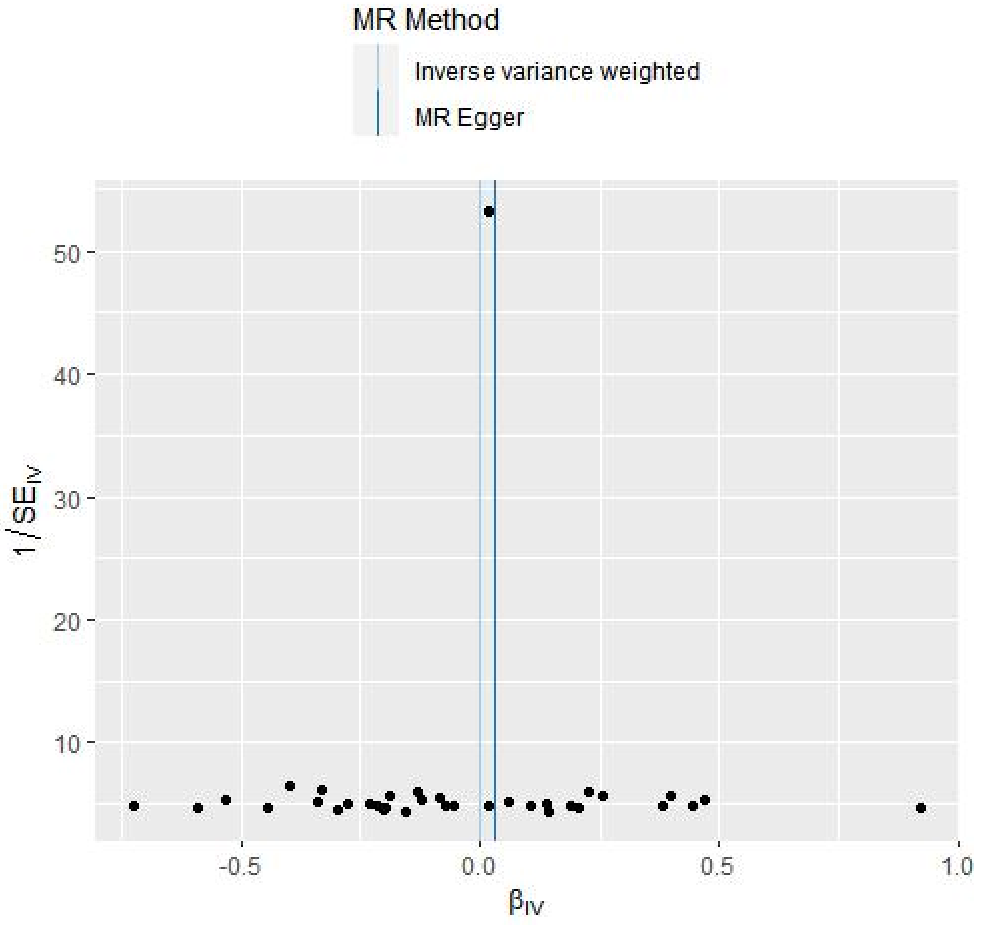

Figure 167: Leave-one-out plot to visualize causal effect of propionic acid on the risk of systolic blood

pressure when leaving one SNP out.

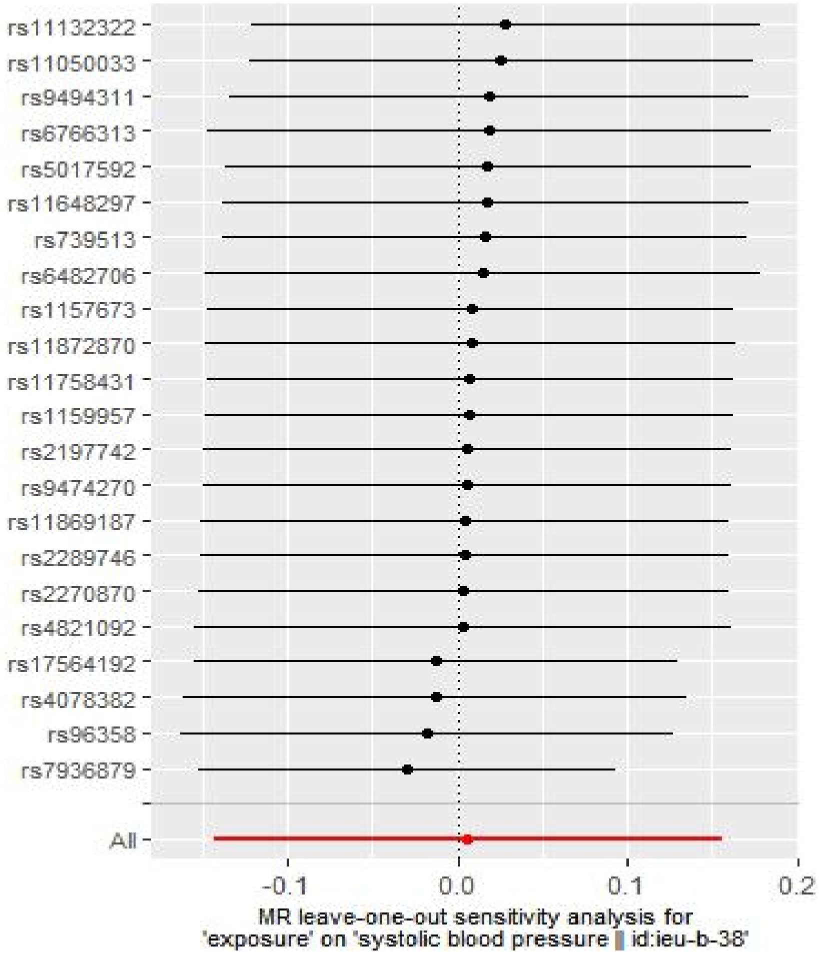

Figure 168: Funnel plots to visualize overall heterogeneity of Mendelian randomization (MR)

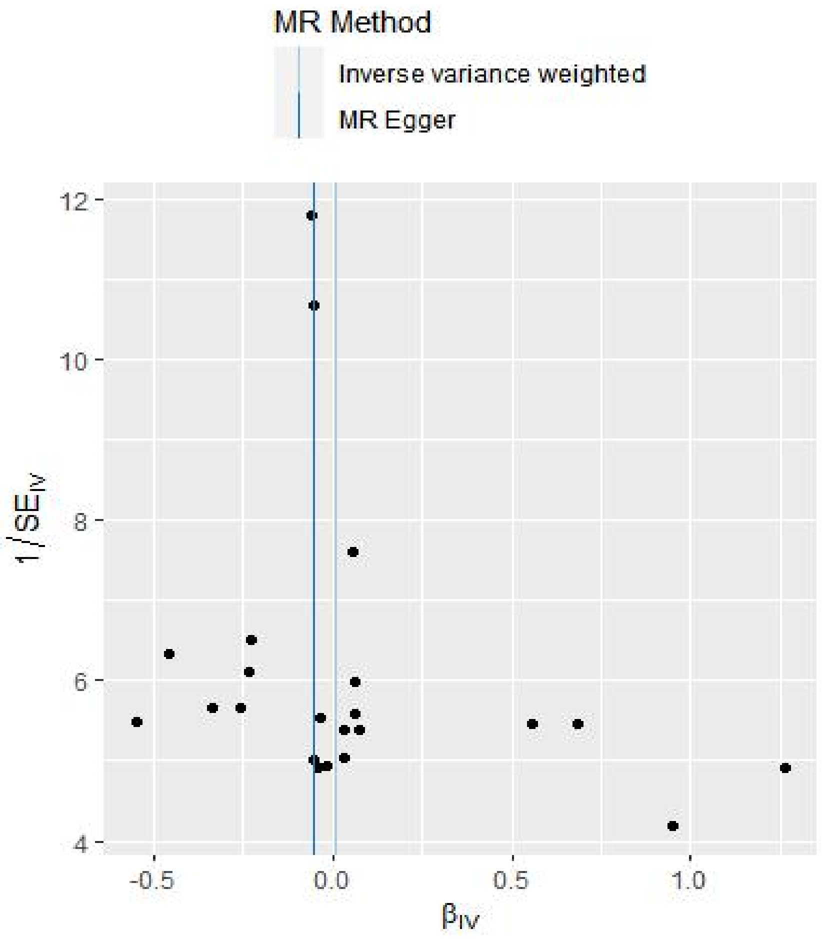

Figure 169: Leave-one-out plot to visualize causal effect of beta_hydroxybutyric acid on the risk of

diastolic blood pressure when leaving one SNP out.

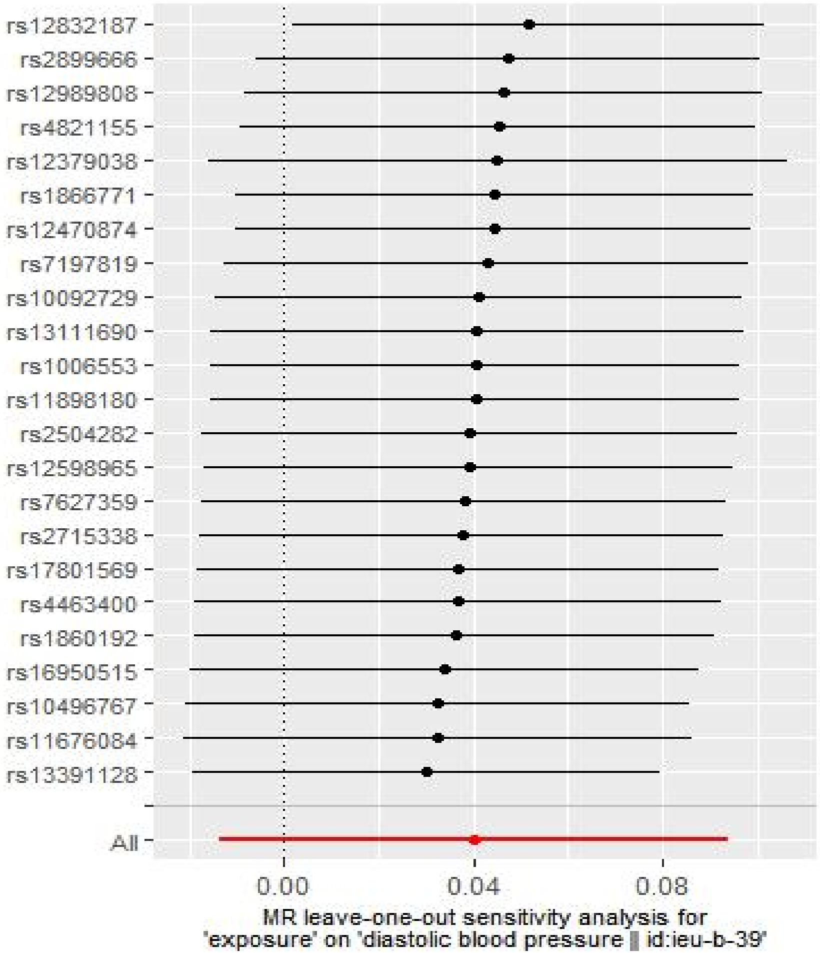

Figure 170: Funnel plots to visualize overall heterogeneity of Mendelian randomization (MR)

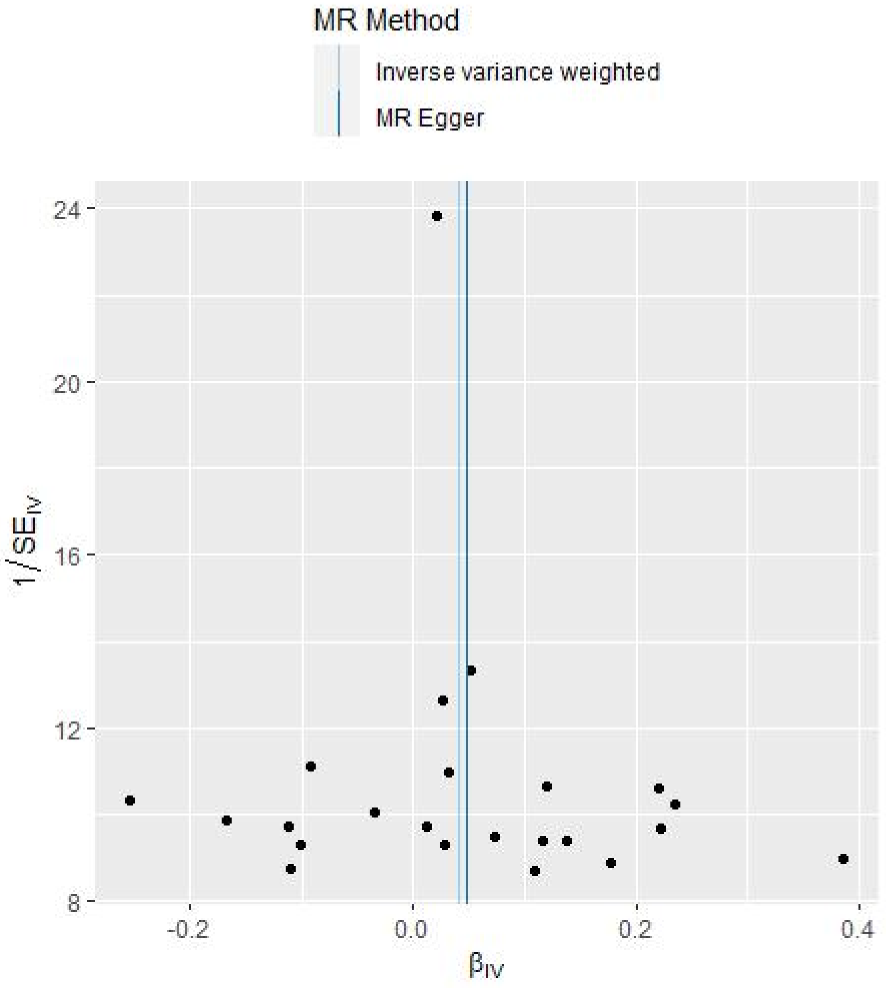

Figure 171: Leave-one-out plot to visualize causal effect of betaine on the risk of diastolic blood

pressure when leaving one SNP out.

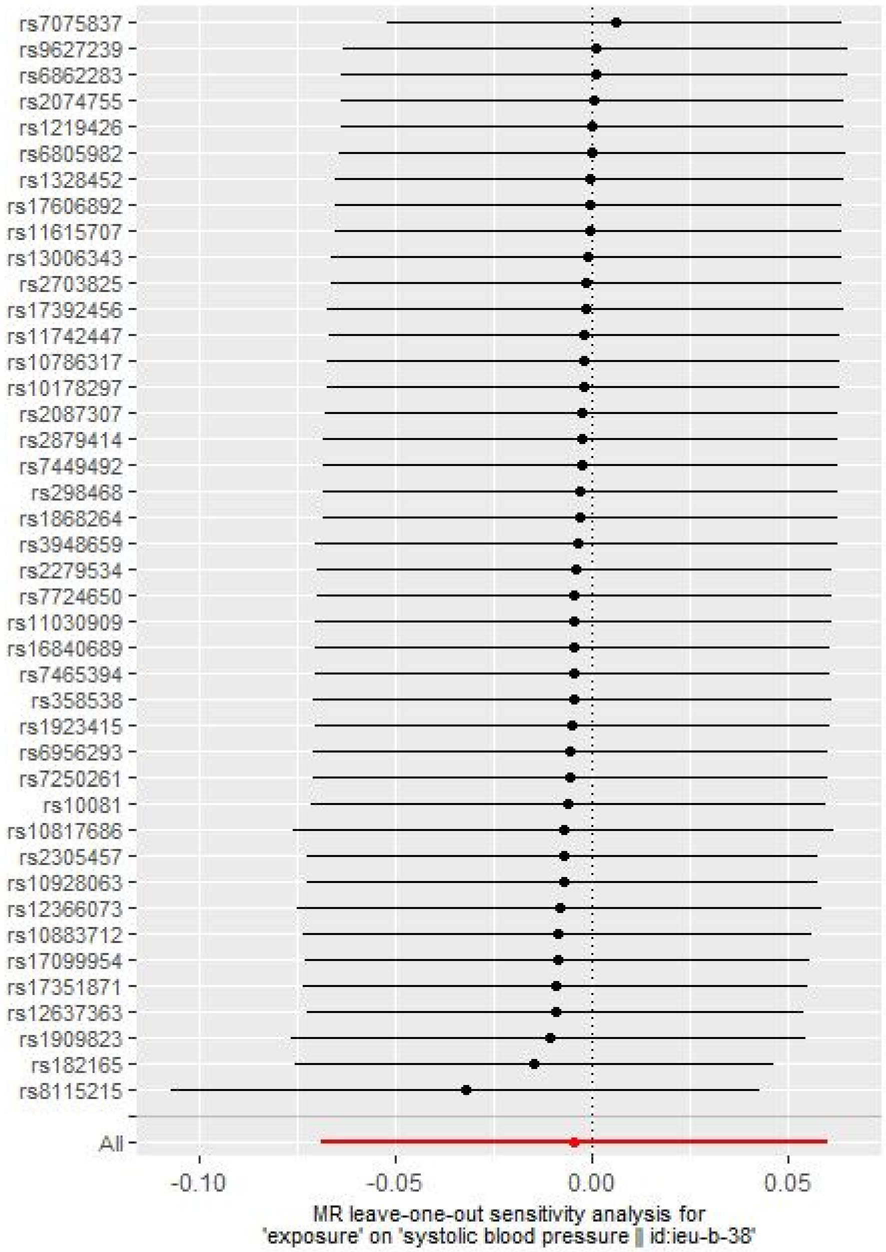

Figure 172: Funnel plots to visualize overall heterogeneity of Mendelian randomization (MR)

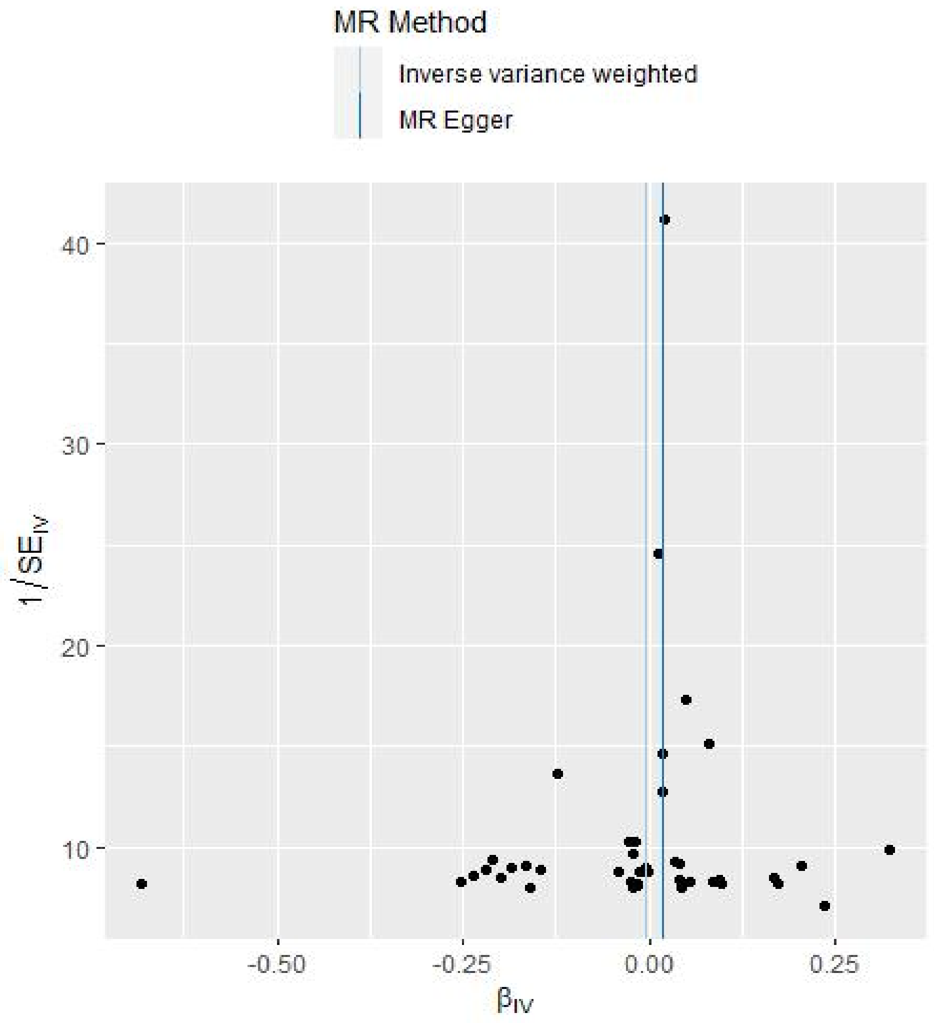

Figure 173: Leave-one-out plot to visualize causal effect of carnitine on the risk of diastolic blood

pressure when leaving one SNP out.

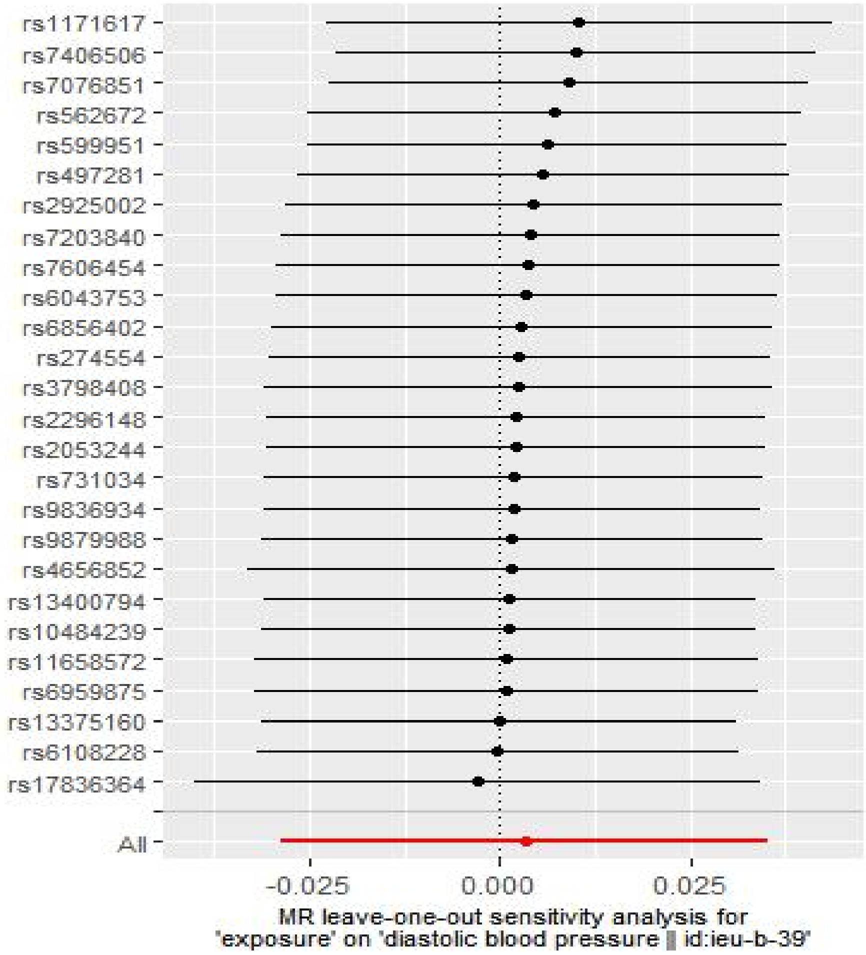

Figure 174: Funnel plots to visualize overall heterogeneity of Mendelian randomization (MR)

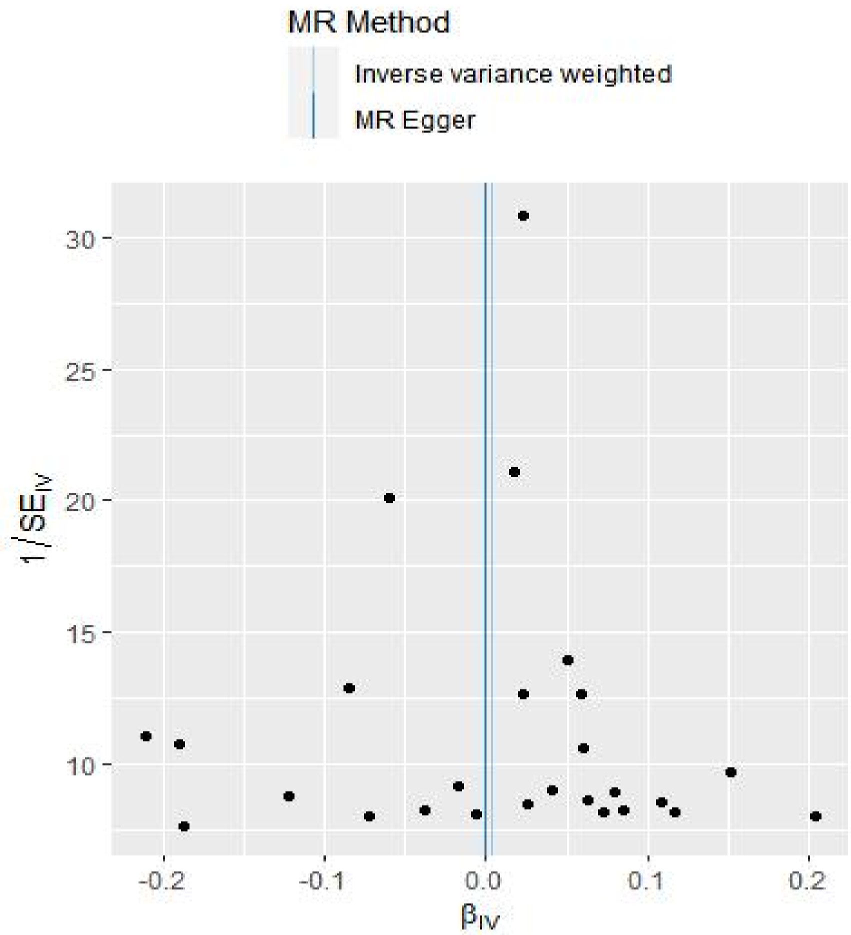

Figure 175: Leave-one-out plot to visualize causal effect of choline on the risk of diastolic blood

pressure when leaving one SNP out.

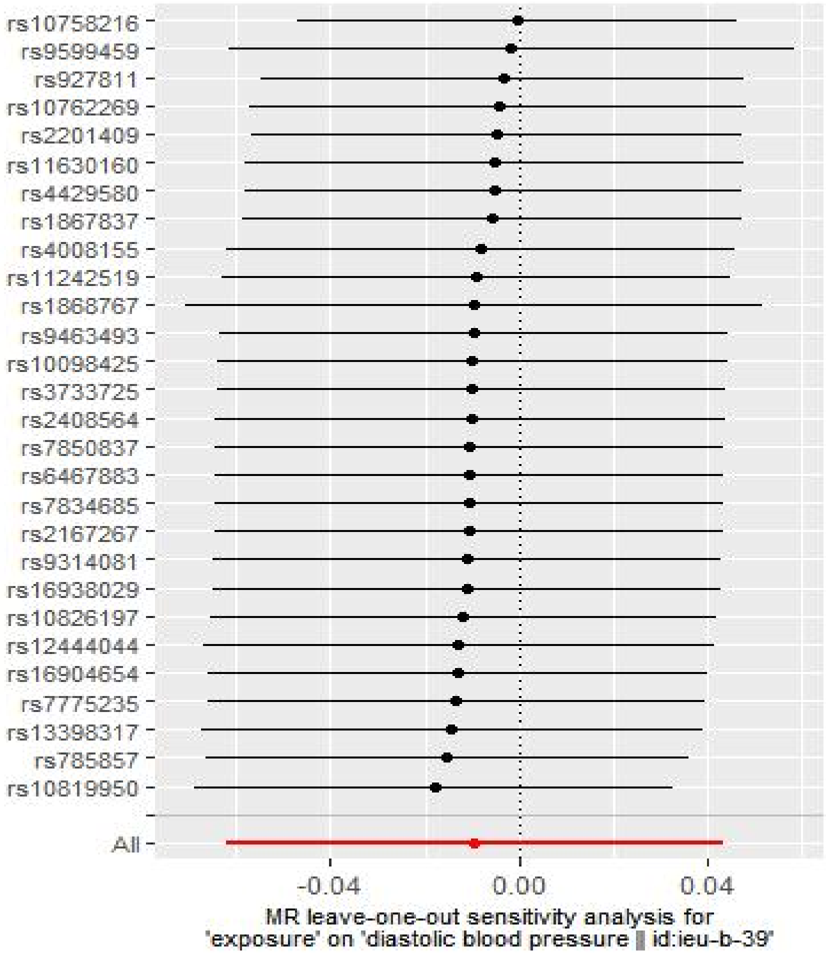

Figure 176: Funnel plots to visualize overall heterogeneity of Mendelian randomization (MR)

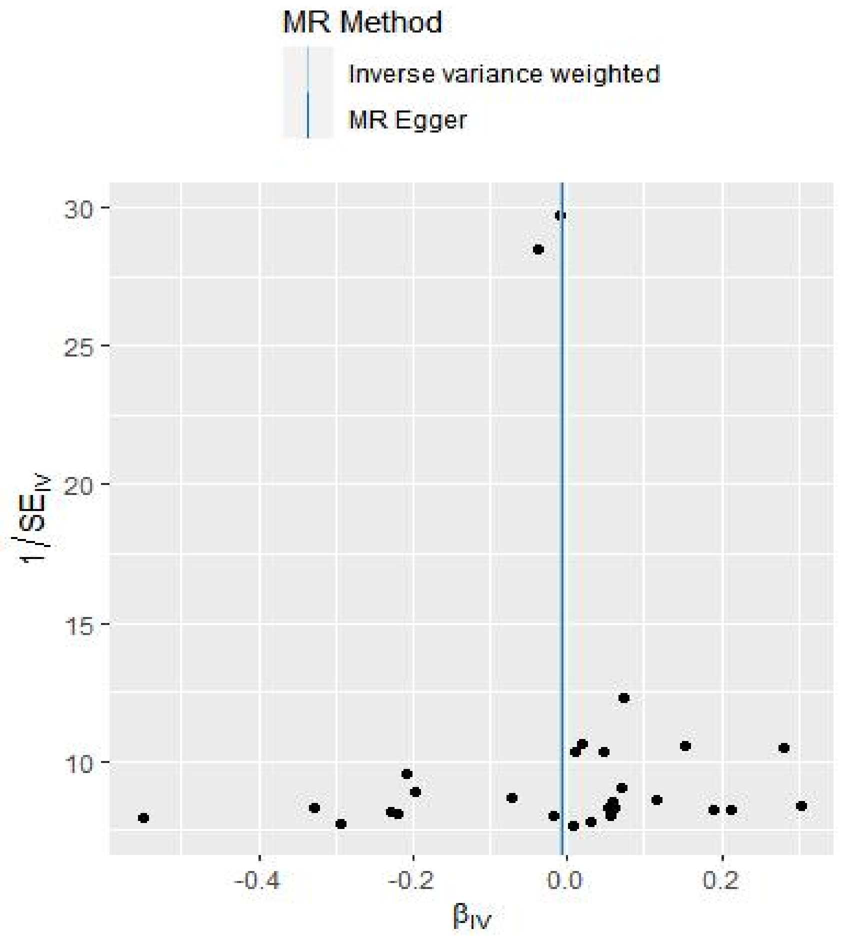

Figure 177: Leave-one-out plot to visualize causal effect of glutamate on the risk of diastolic blood

pressure when leaving one SNP out.

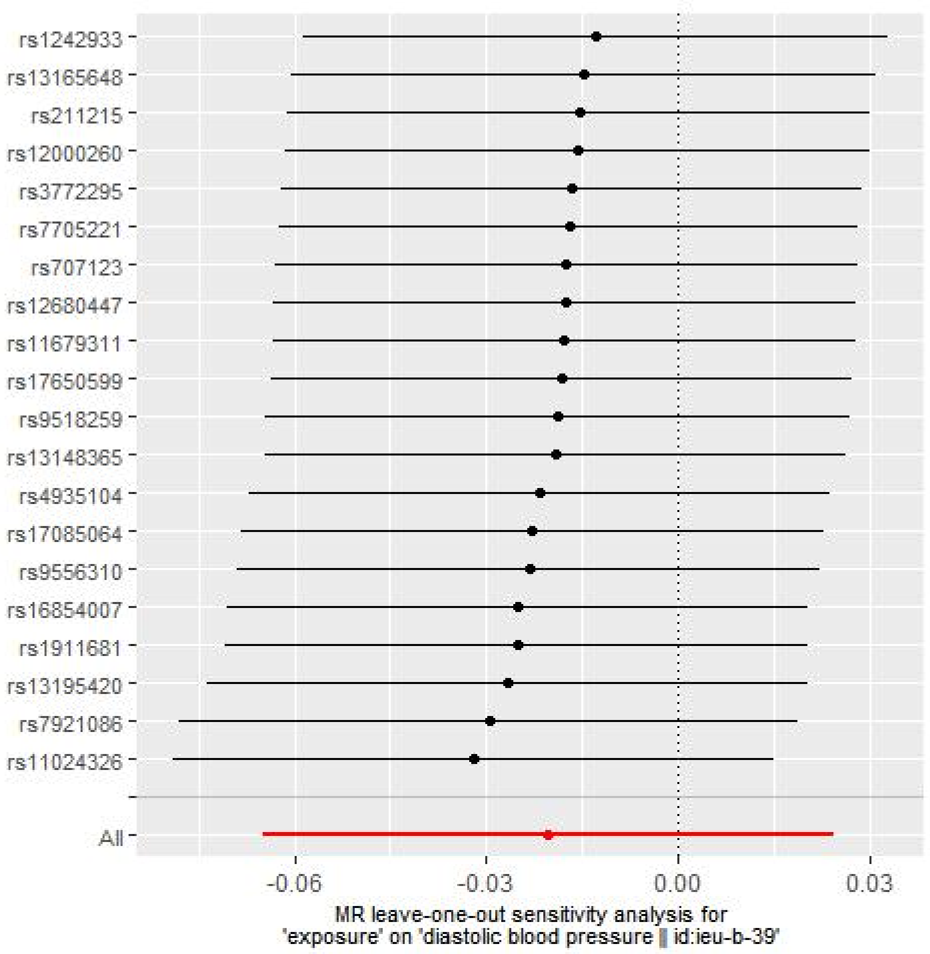

Figure 178: Funnel plots to visualize overall heterogeneity of Mendelian randomization (MR)

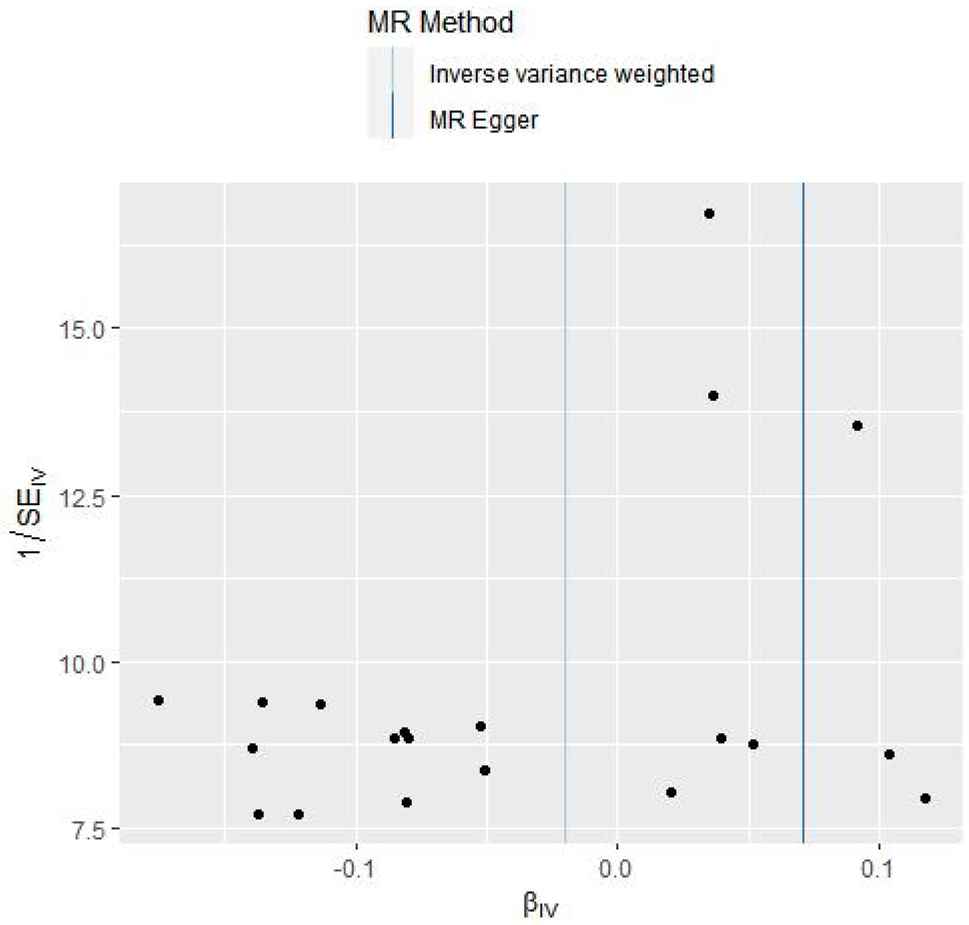

Figure 179: Leave-one-out plot to visualize causal effect of kynuremine on the risk of diastolic blood

pressure when leaving one SNP out.

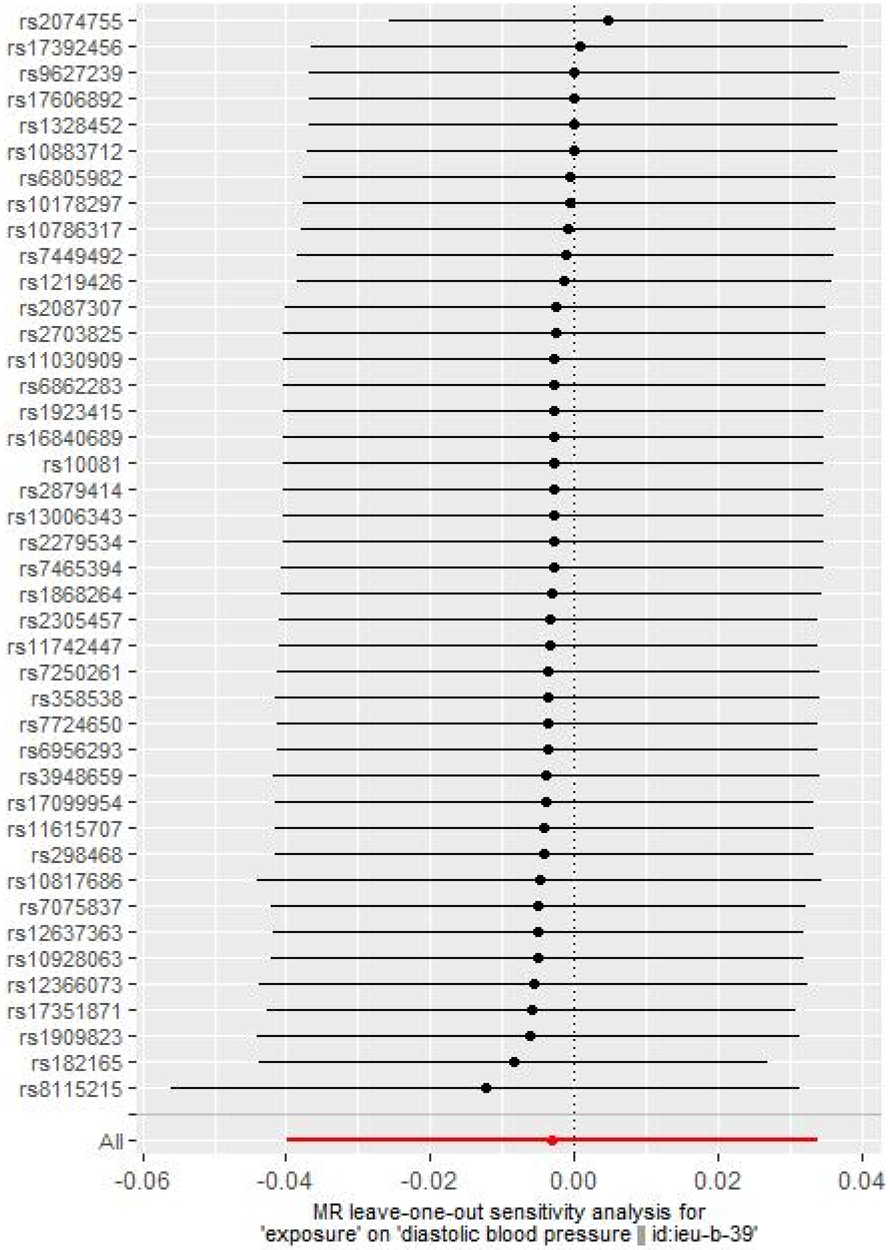

Figure 180: Funnel plots to visualize overall heterogeneity of Mendelian randomization (MR)

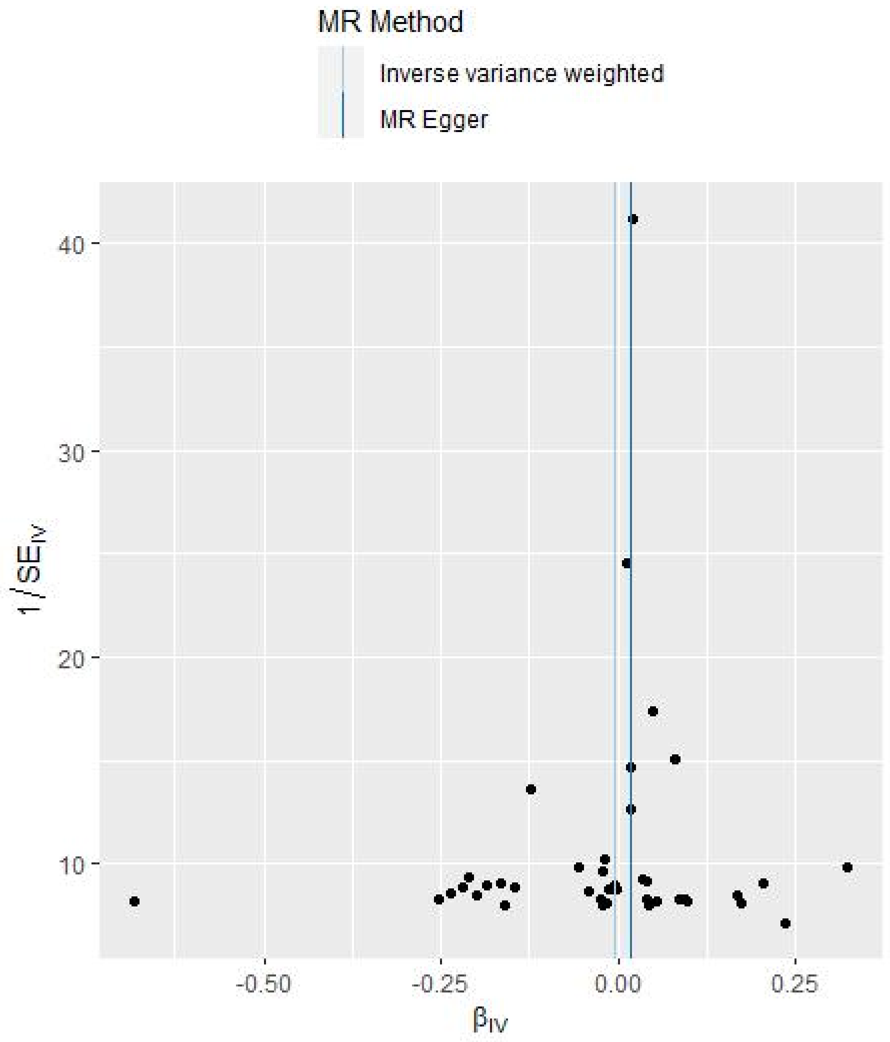

Figure 181: Leave-one-out plot to visualize causal effect of phenylalanine on the risk of diastolic blood

pressure when leaving one SNP out.

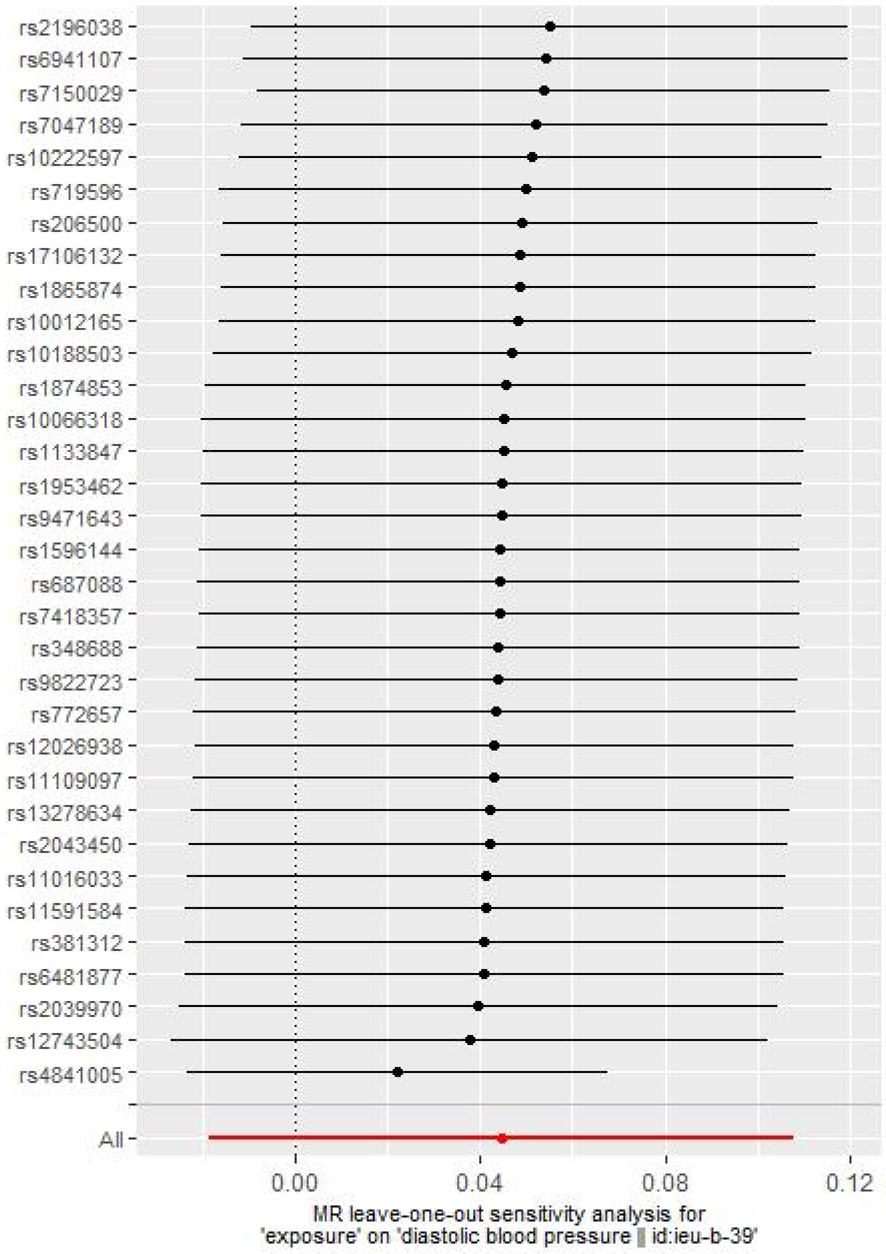

Figure 182: Funnel plots to visualize overall heterogeneity of Mendelian randomization (MR)

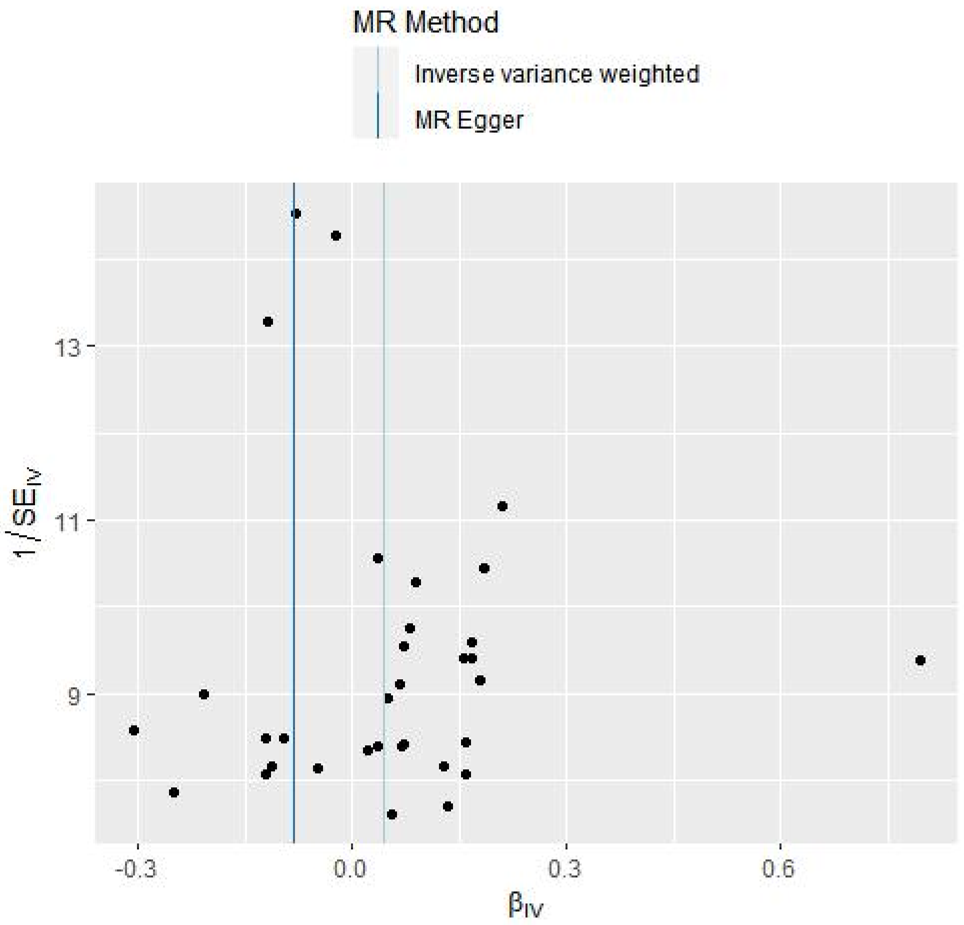

Figure 183: Leave-one-out plot to visualize causal effect of serotonin on the risk of diastolic blood

pressure when leaving one SNP out.

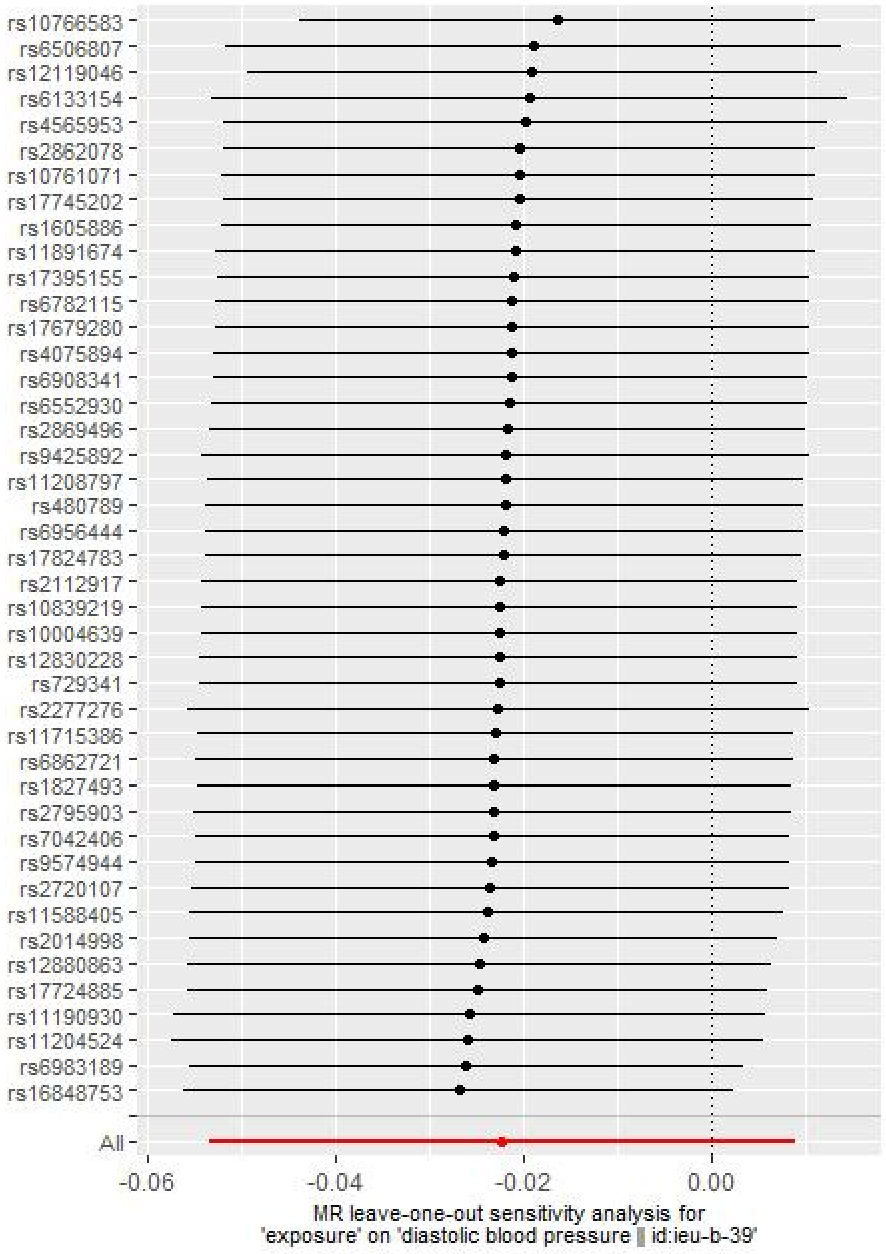

Figure 184: Funnel plots to visualize overall heterogeneity of Mendelian randomization (MR)

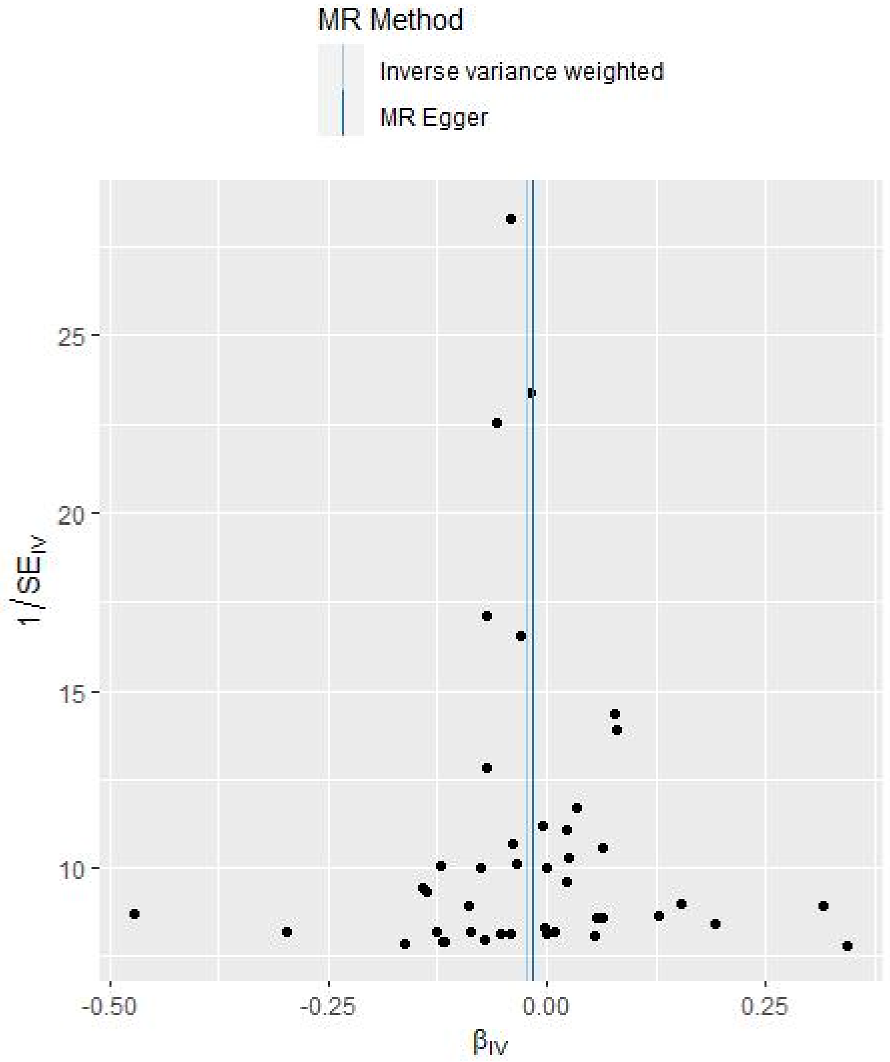

Figure 185: Leave-one-out plot to visualize causal effect of trimethylamine_N_oxide on the risk of

diastolic blood pressure when leaving one SNP out.

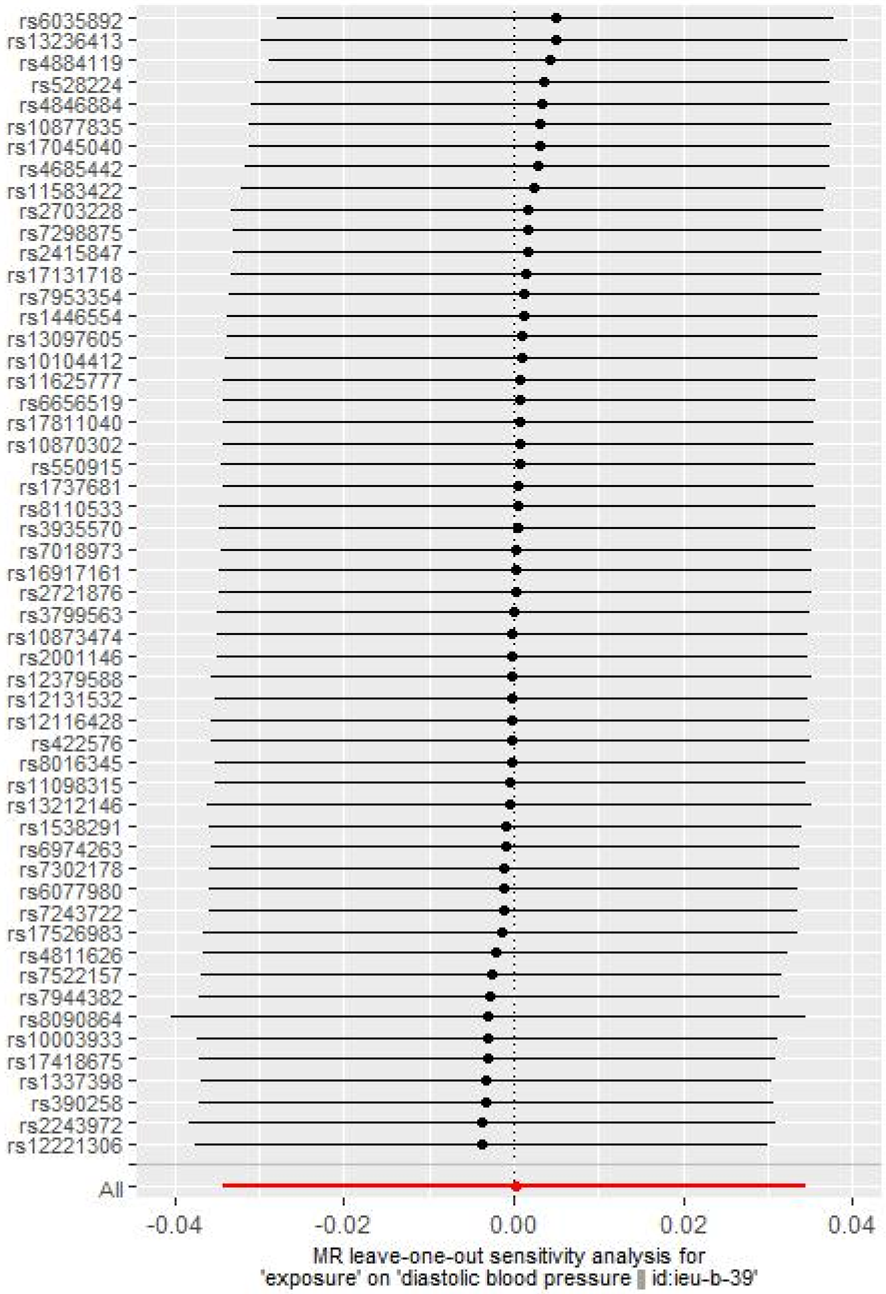

Figure 186: Funnel plots to visualize overall heterogeneity of Mendelian randomization (MR)

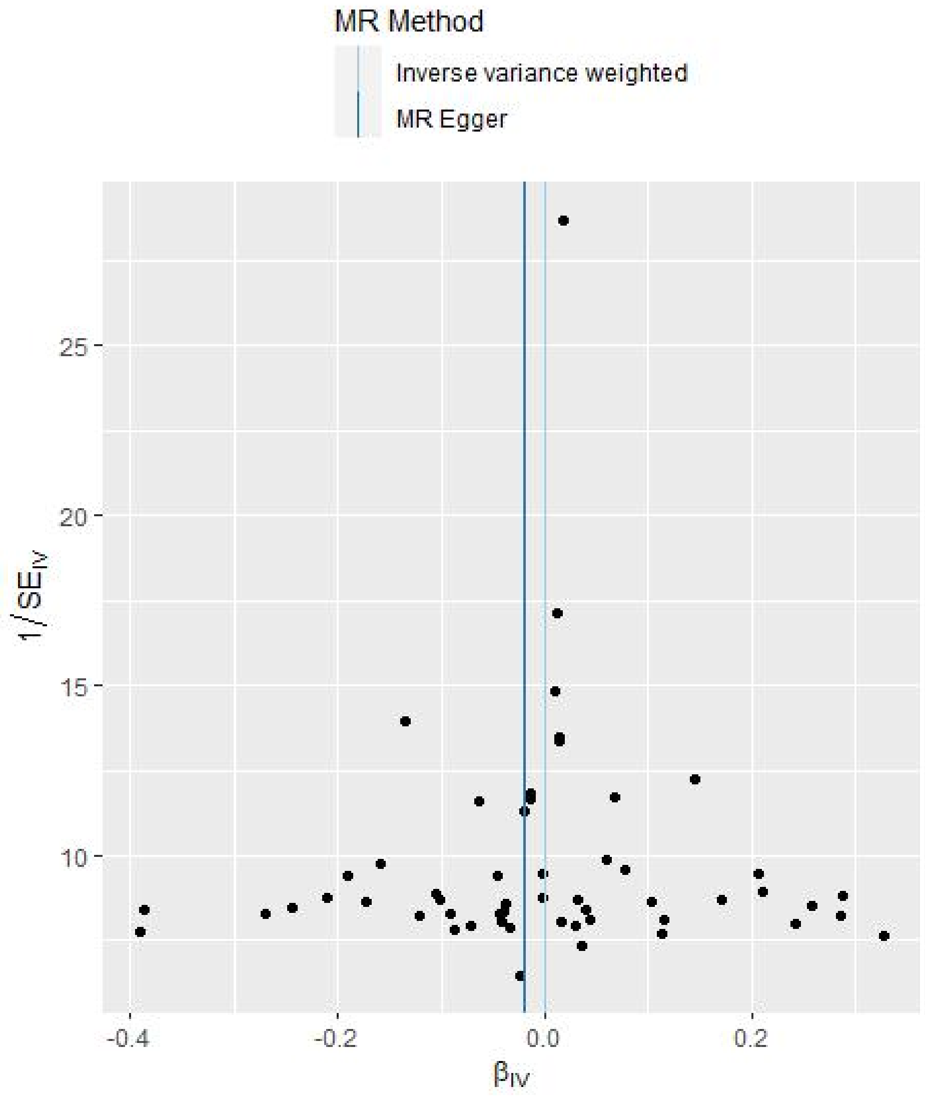

Figure 187: Leave-one-out plot to visualize causal effect of tyrosine on the risk of diastolic blood

pressure when leaving one SNP out.

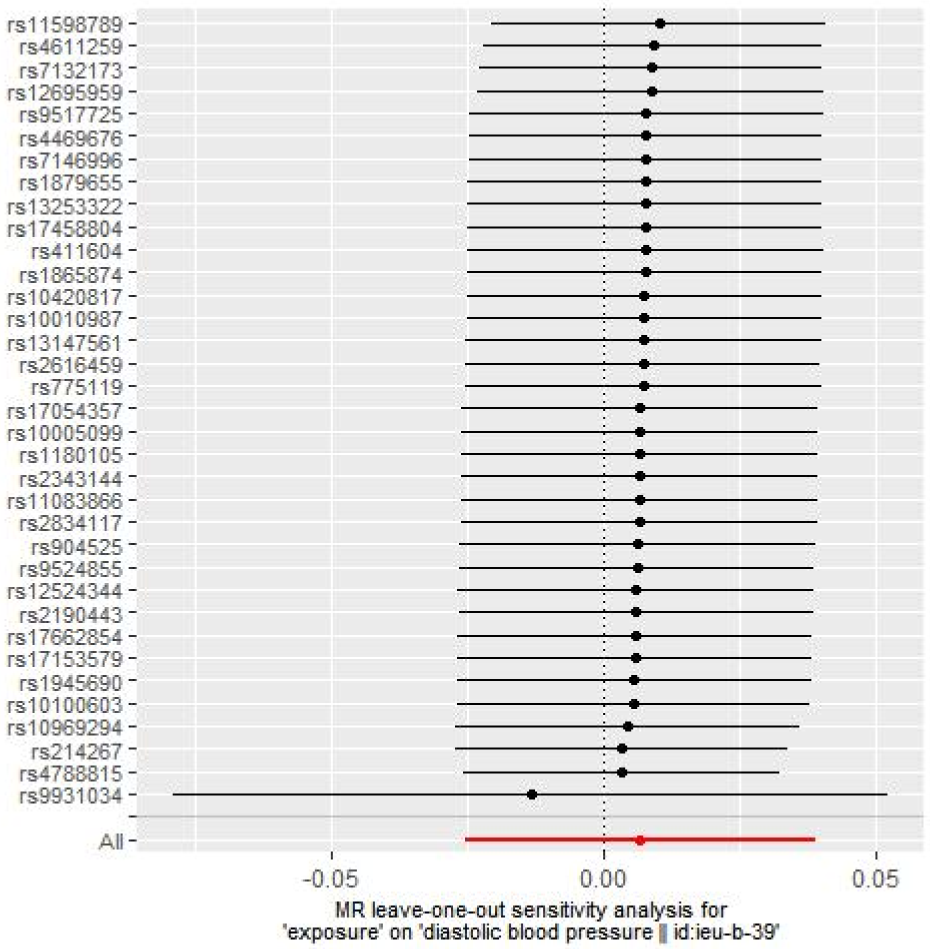

Figure 188: Funnel plots to visualize overall heterogeneity of Mendelian randomization (MR)

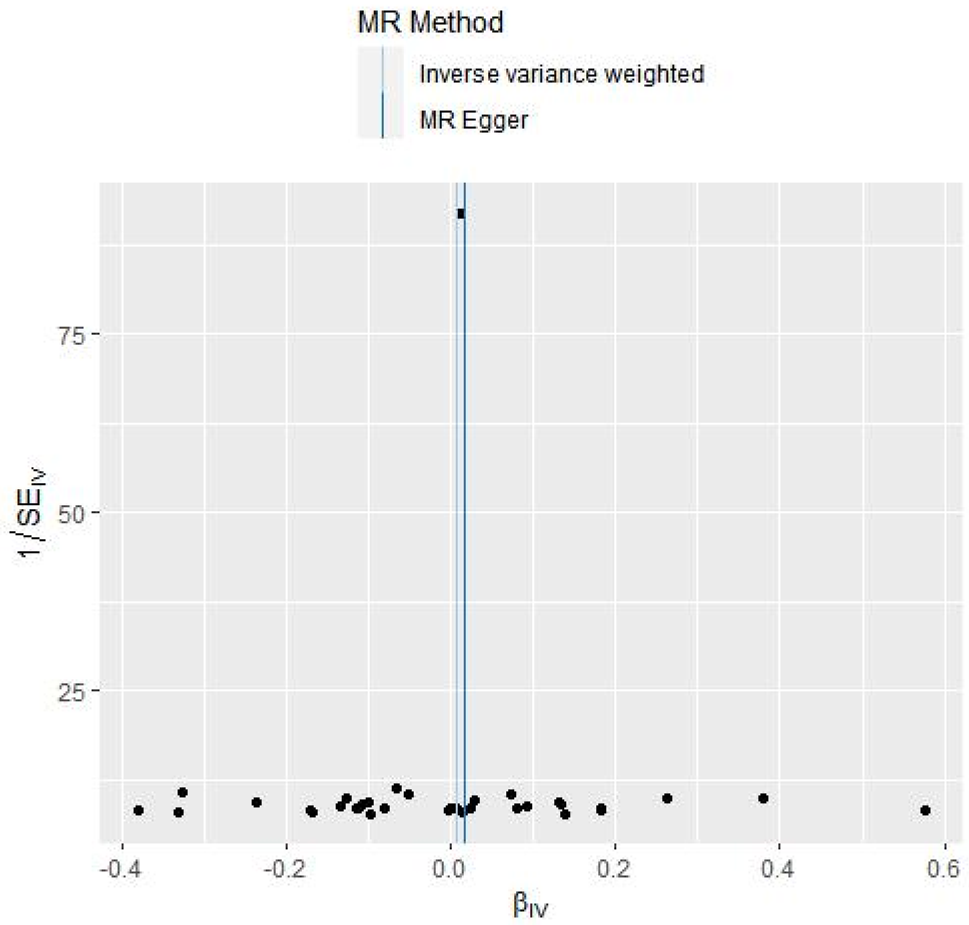

Figure 189: Leave-one-out plot to visualize causal effect of tryptophan on the risk of diastolic blood

pressure when leaving one SNP out.

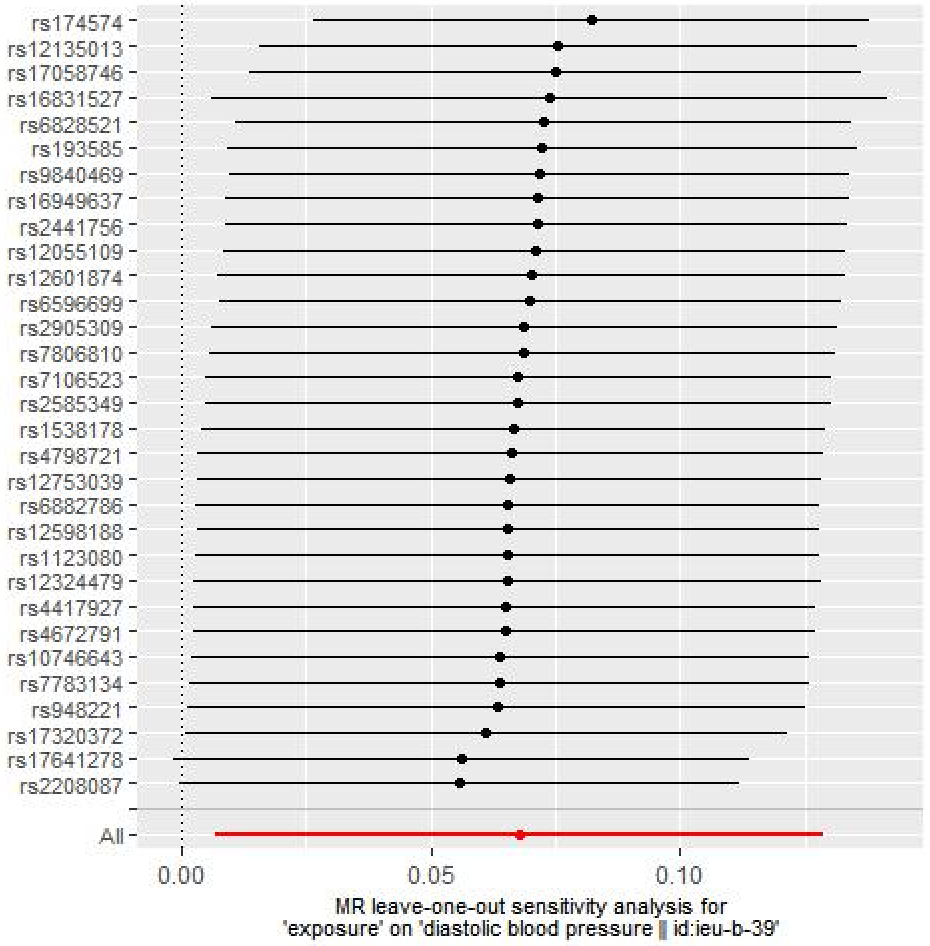

Figure 190: Funnel plots to visualize overall heterogeneity of Mendelian randomization (MR)

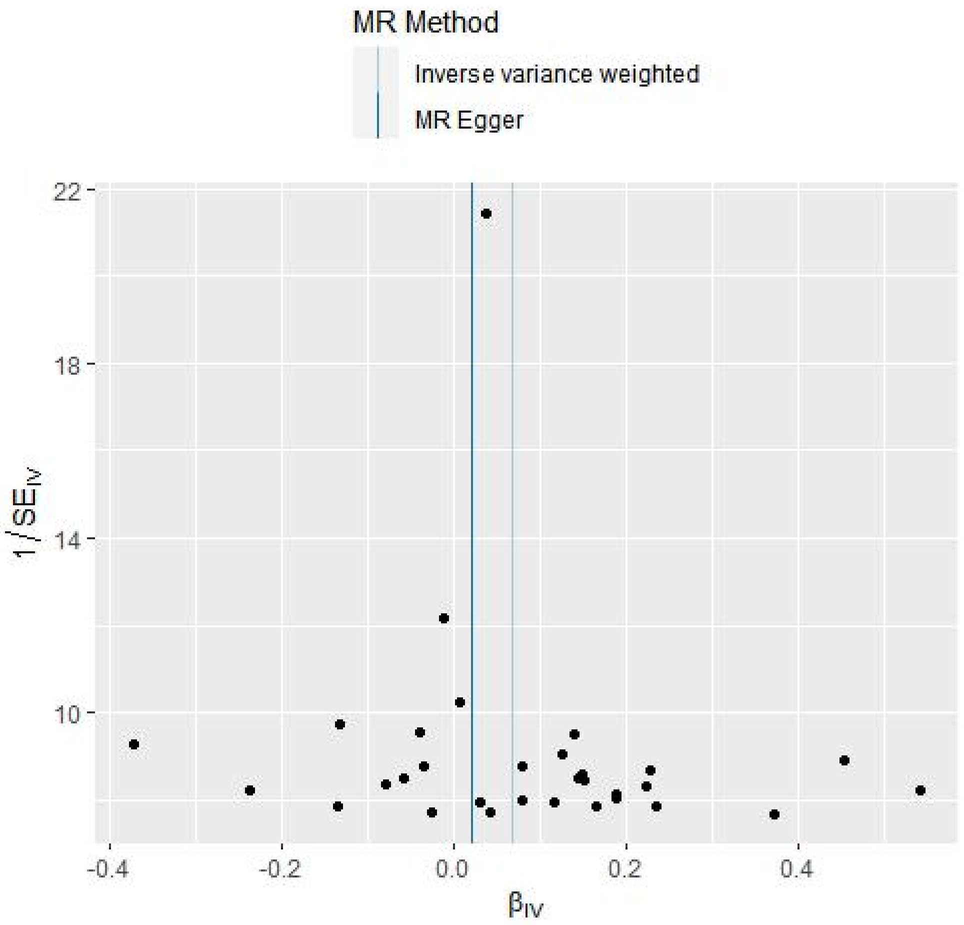

Figure 191: Leave-one-out plot to visualize causal effect of propionic acid on the risk of diastolic blood

pressure when leaving one SNP out.

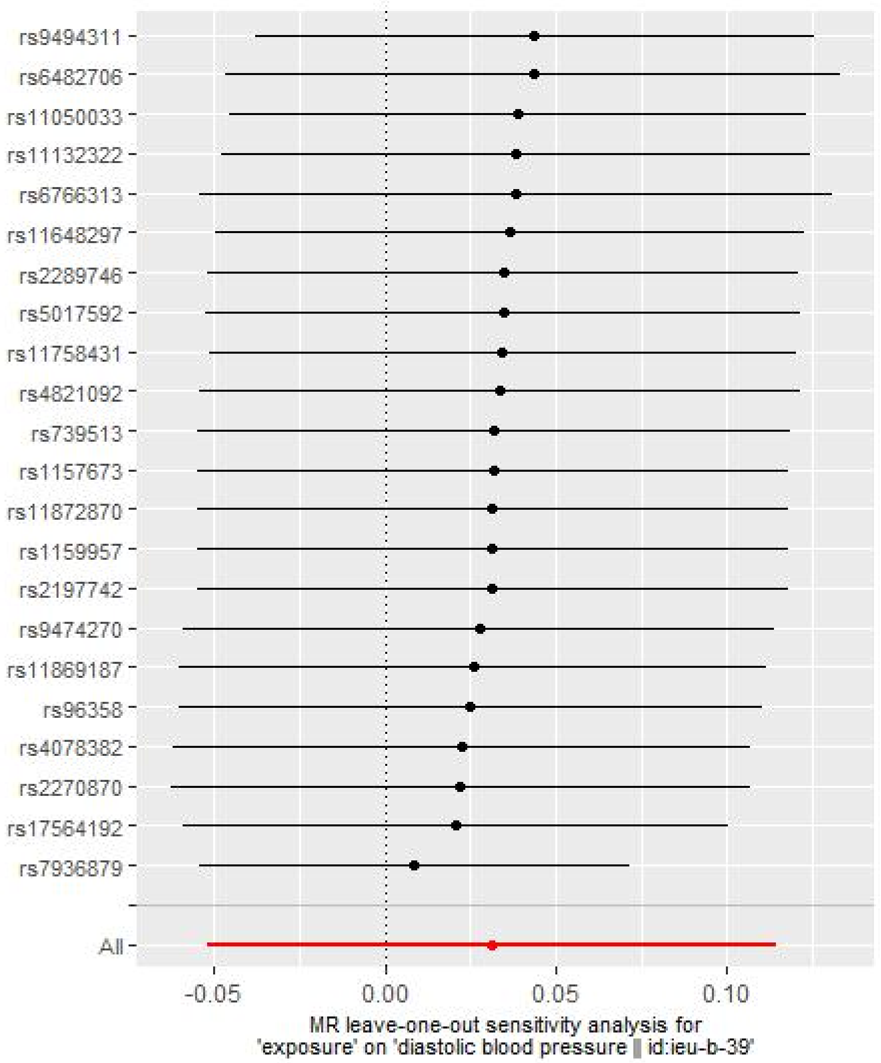

Figure 192: Funnel plots to visualize overall heterogeneity of Mendelian randomization (MR)

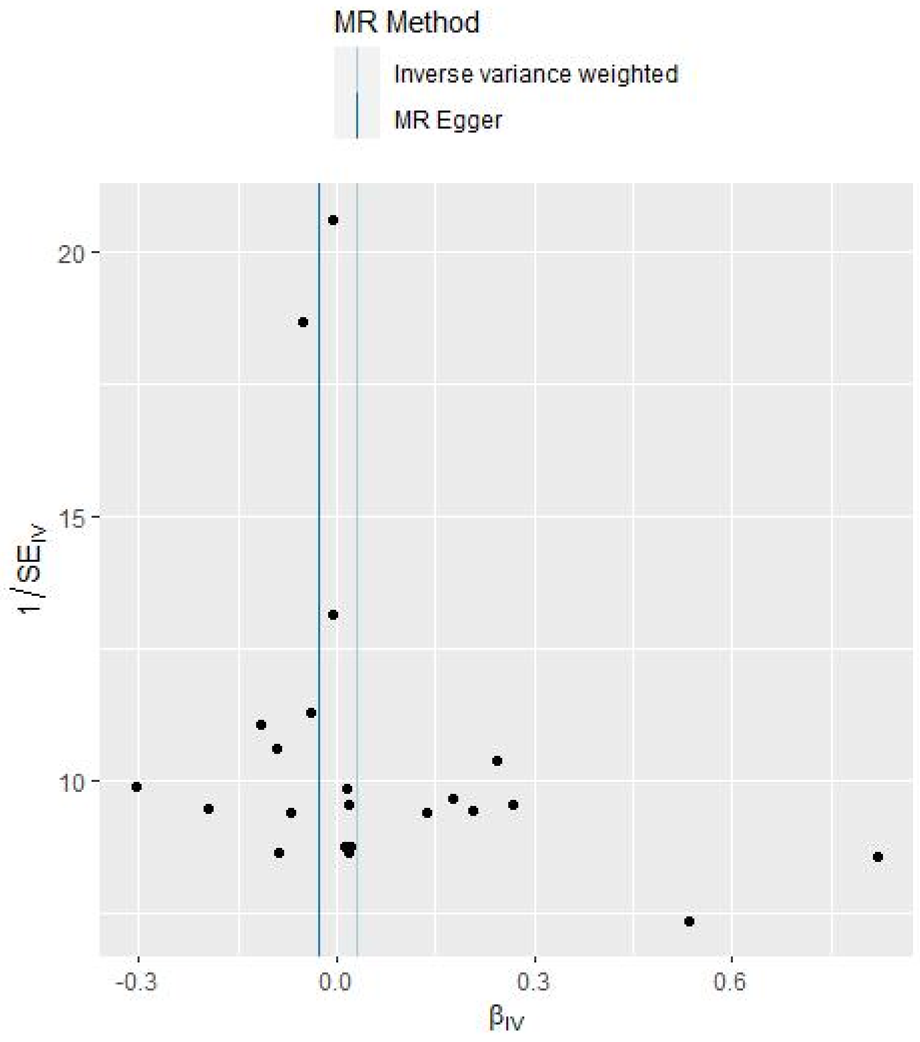

Figure 193: Leave-one-out plot to visualize causal effect of beta_hydroxybutyric acid on the risk of

diabetes when leaving one SNP out.

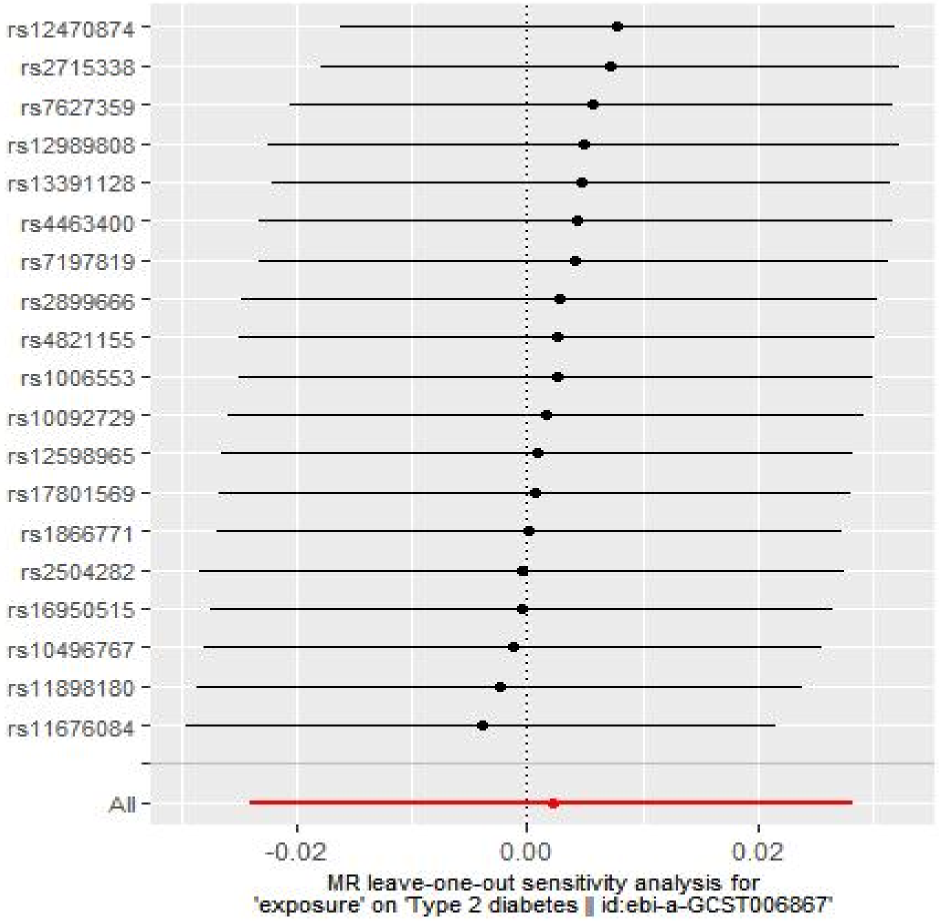

Figure 194: Funnel plots to visualize overall heterogeneity of Mendelian randomization (MR)

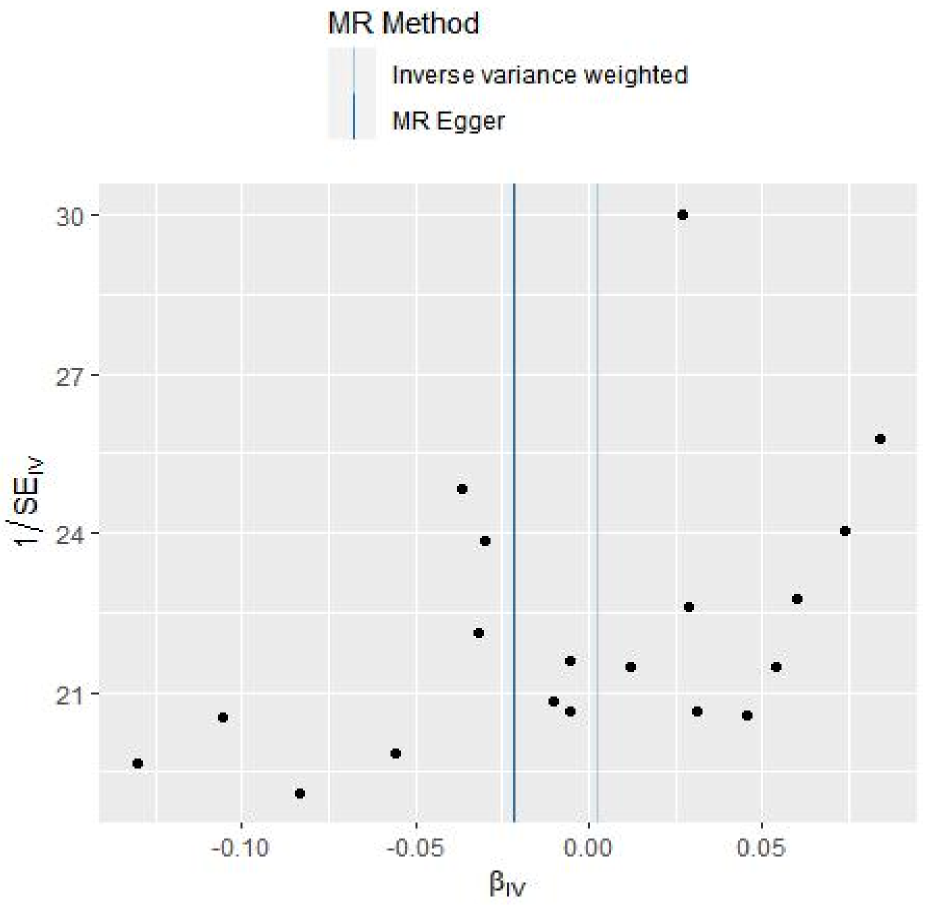

Figure 195: Leave-one-out plot to visualize causal effect of betaine on the risk of diabetes when leaving

one SNP out.

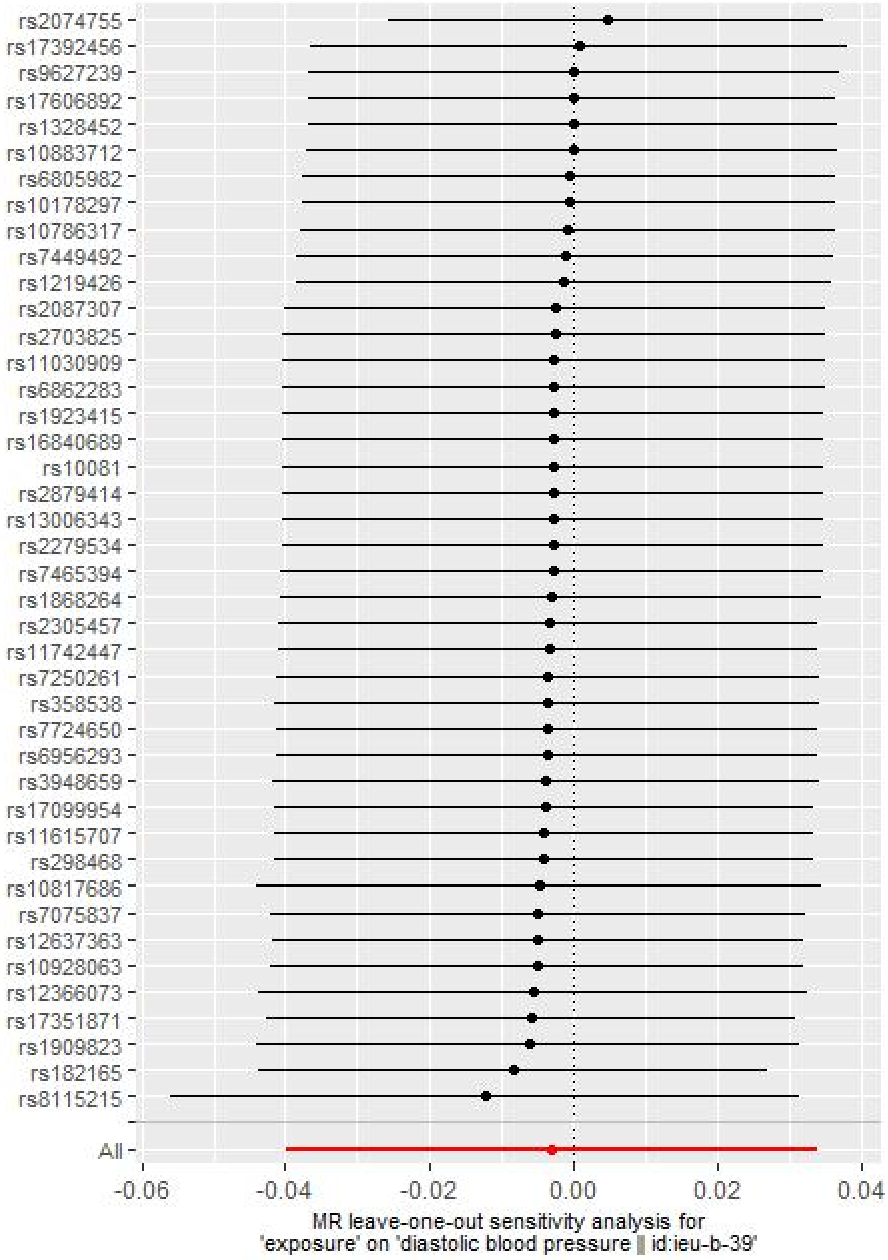

Figure 196: Funnel plots to visualize overall heterogeneity of Mendelian randomization (MR)

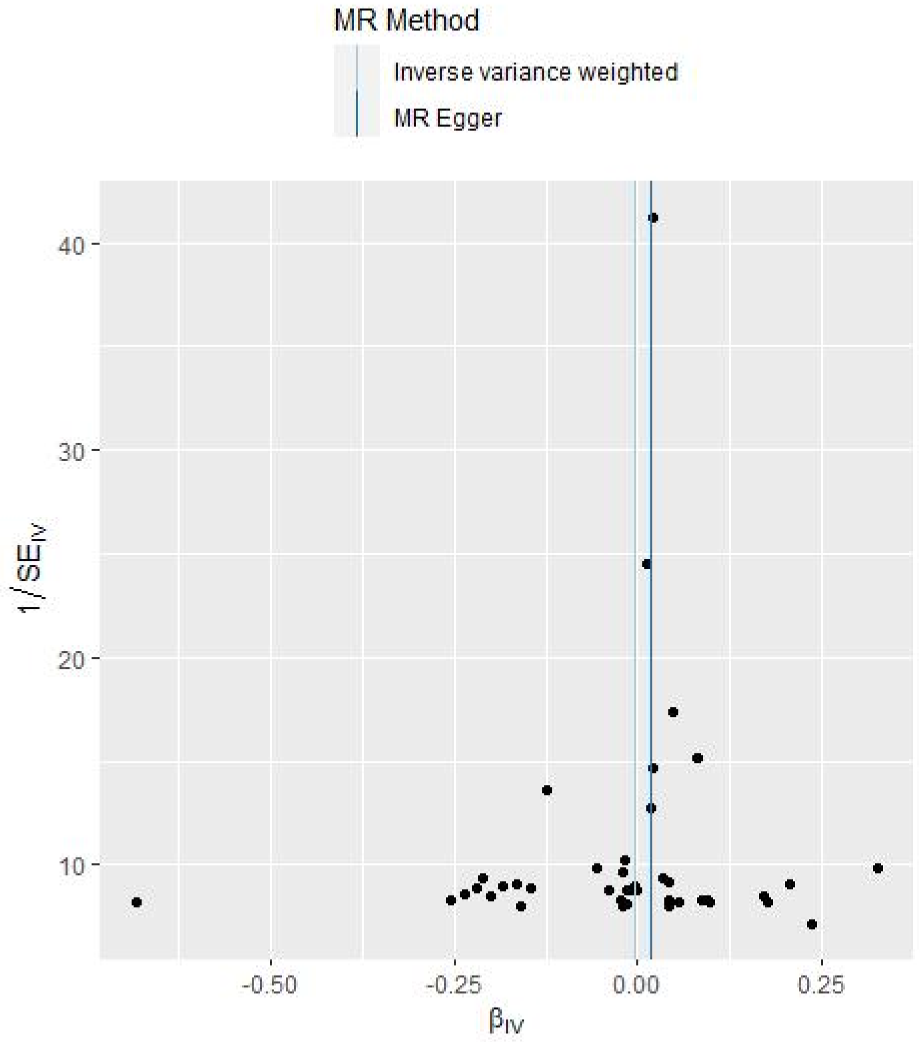

Figure 197: Leave-one-out plot to visualize causal effect of carnitine on the risk of diabetes when

leaving one SNP out.

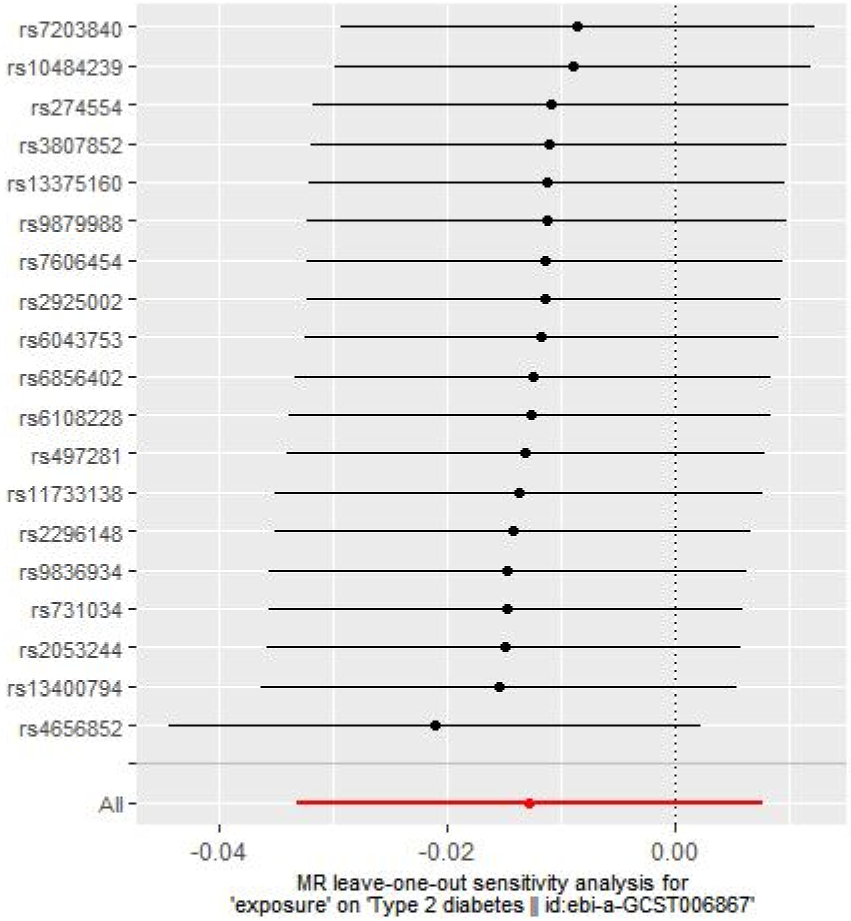

Figure 198: Funnel plots to visualize overall heterogeneity of Mendelian randomization (MR)

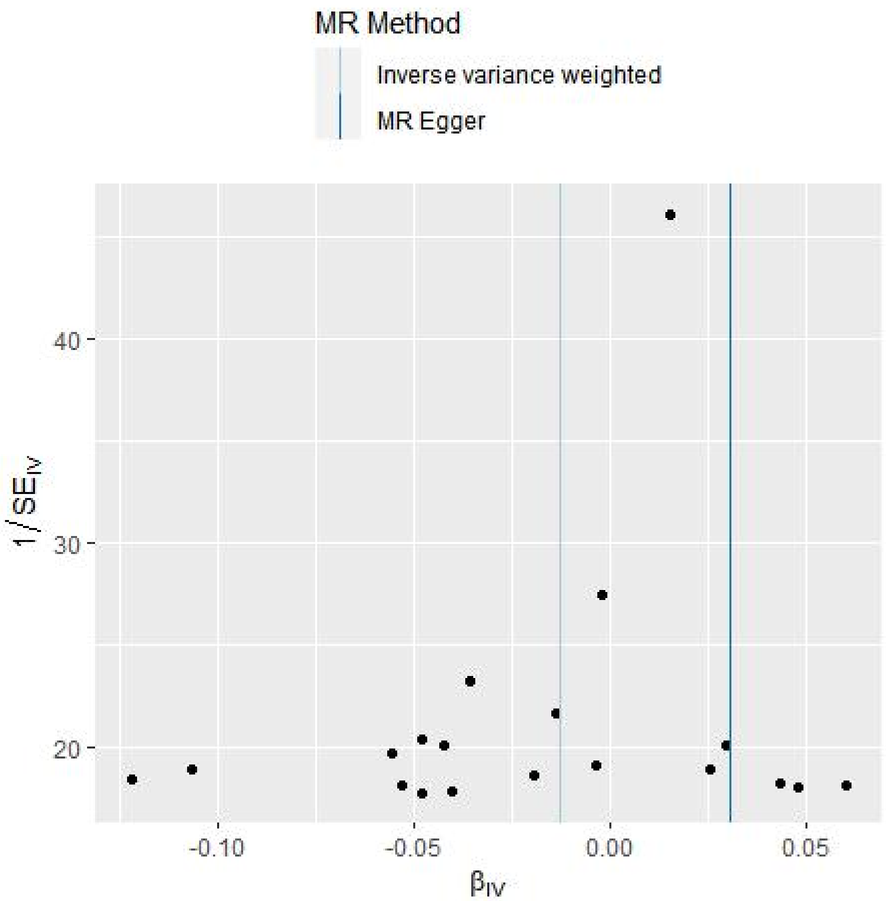

Figure 199: Leave-one-out plot to visualize causal effect of choline on the risk of diabetes when

leaving one SNP out.

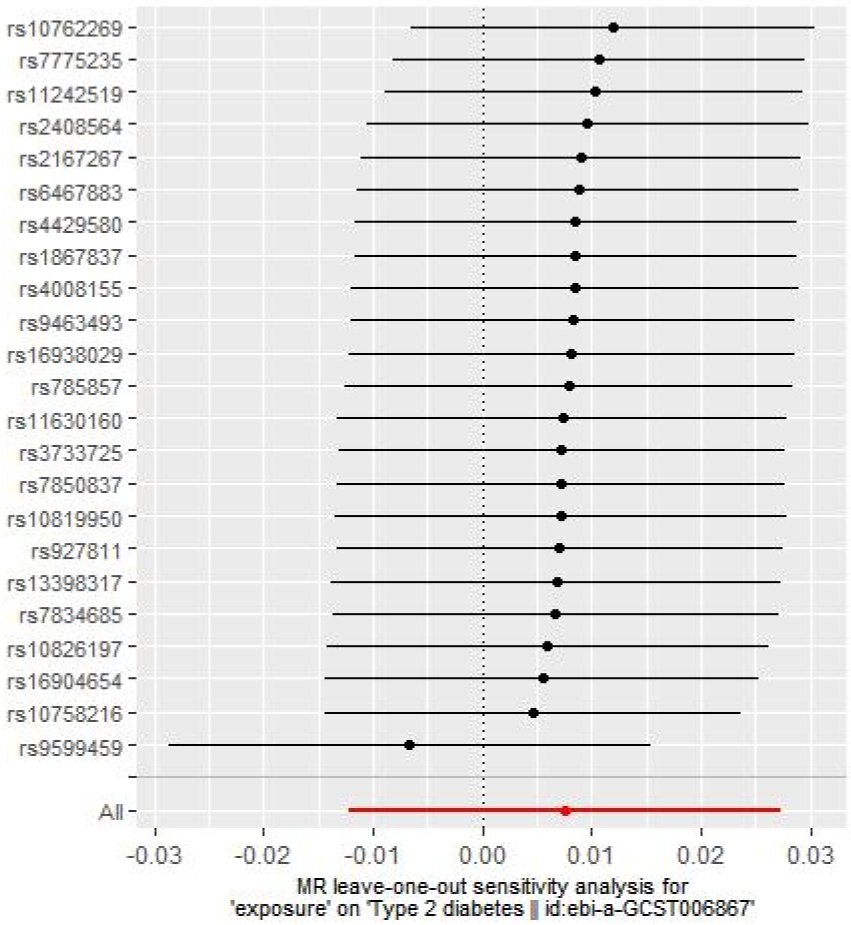

Figure 200: Funnel plots to visualize overall heterogeneity of Mendelian randomization (MR)

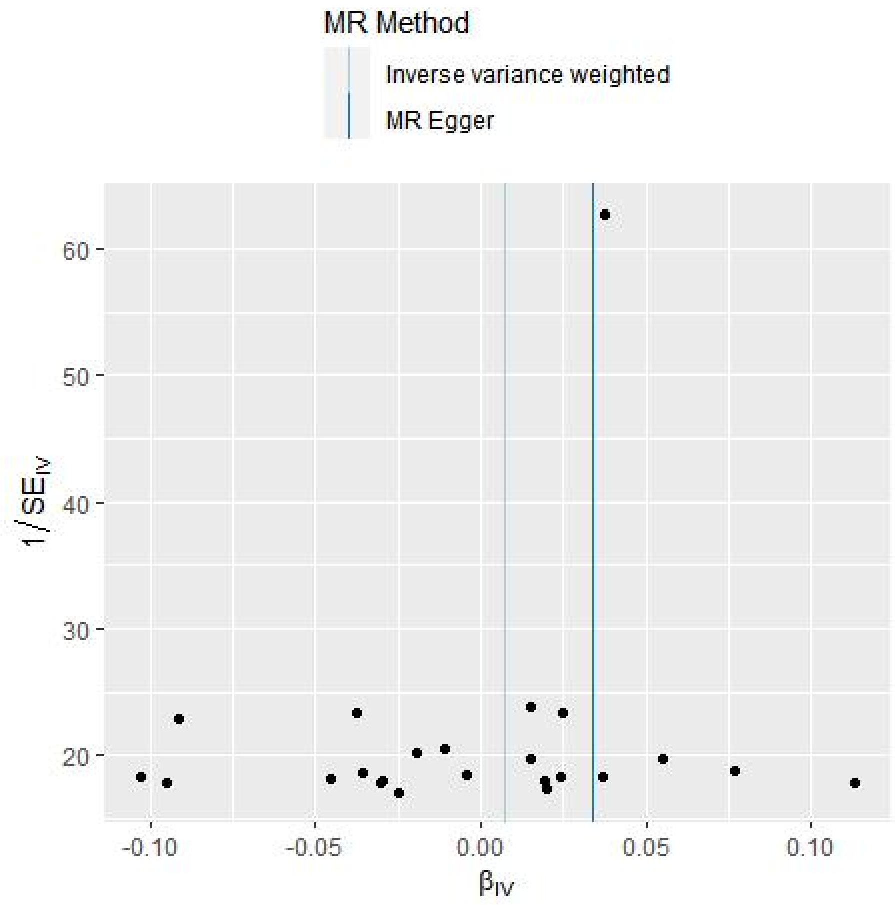
MR Method
Inverse variance weighted
MR Egger
60
50
40
30
20
-0.10
-0.05
0.00
0.05
0.10
$1/SE_{IV}$
β_{IV}
