# Supplementary material for: Effects of Gut Microbiota and Metabolites on Heart Failure and Its Risk Factors: A Two-Sample Mendelian Randomization Study
Source: Front Nutr. 2022 Jun 20;9:899746. doi: 10.3389/fnut.2022.899746 (PMC9253861; doi:10.3389/fnut.2022.899746)

Supplementary Information

Effects of Gut Microbiota and Metabolites on Heart Failure and Its Risk Factors: A Two-sample Mendelian Randomization Study

Qiang Luo[a], Yilan Hu[a], Xin Chen[a], Yong Luo[a], Jie Chen[a], Han Wang[a, *]

Figure 1-24: Effects of gut metabolites and heart failure.

Figure 25-48: Effects of gut metabolites and atrial fibrillation

Figure 49-72: Effects of gut metabolites and hypertrophic cardiomyopathy

Figure 73-96: Effects of gut metabolites and coronary heart disease

Figure 97-120: Effects of gut metabolites and dilated cardiomyopathy

Figure 121-144: Effects of gut metabolites and chronic kidney disease

Figure 145-168: Effects of gut metabolites and systolic blood pressure

Figure 169-192: Effects of gut metabolites and diastolic blood pressure

Figure 193-216: Effects of gut metabolites and diabetes

Figure 217-240: Effects of gut metabolites and myocardial infarction

Figure 241-254: Effects of gut metabolites and myocarditis

Figure 255-278: Effects of gut metabolites and valvular heart disease

Figure 279-284: Effects of gut microbiota and atrial fibrillation

Figure 285-290: Effects of gut microbiota and hypertrophic cardiomyopathy

Figure 291-296: Effects of gut microbiota and coronary heart disease

Figure 297-302: Effects of gut microbiota and dilated cardiomyopathy

Figure 303-308: Effects of gut microbiota and chronic kidney disease

Figure 309-314: Effects of gut microbiota and systolic blood pressure

Figure 315-320: Effects of gut microbiota and diastolic blood pressure

Figure 321-326: Effects of gut microbiota and diabetes

Figure 327-322: Effects of gut microbiota and valvular heart disease

Figure 333-338: Effects of gut microbiota and myocardial infarction

Figure 339-342: Effects of gut microbiota and myocarditis

Figure 343-348: Effects of gut microbiota and heart failure

Figure 201: Leave-one-out plot to visualize causal effect of glutamate on the risk of diabetes when leaving one SNP out.

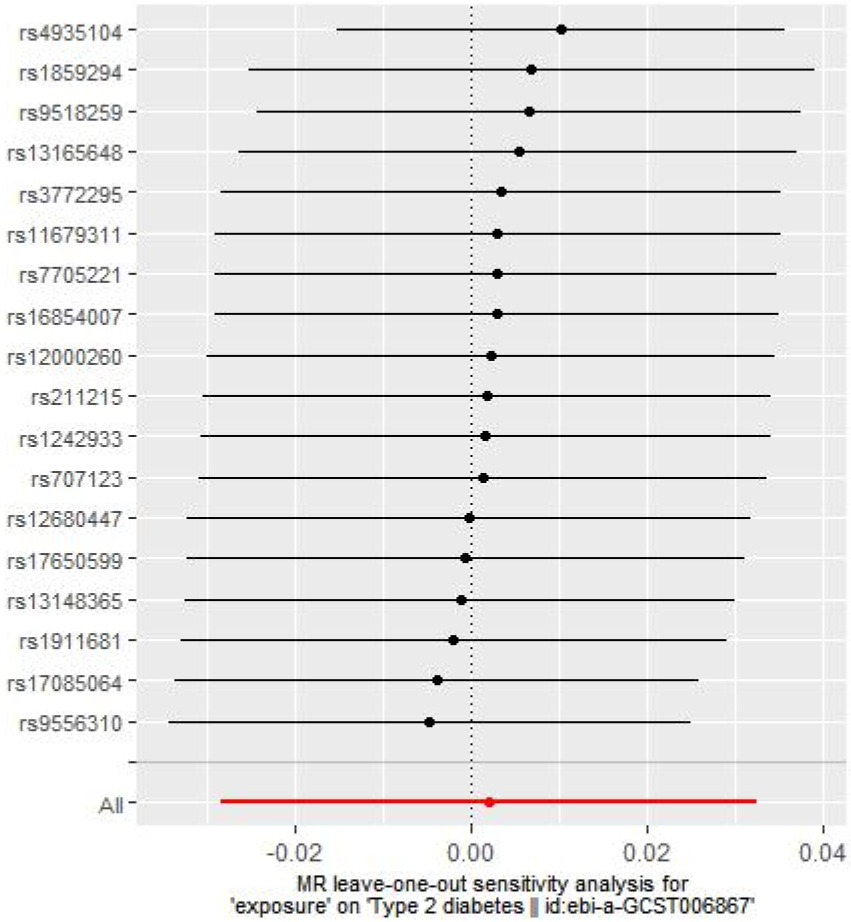

Figure 202: Funnel plots to visualize overall heterogeneity of Mendelian randomization (MR)

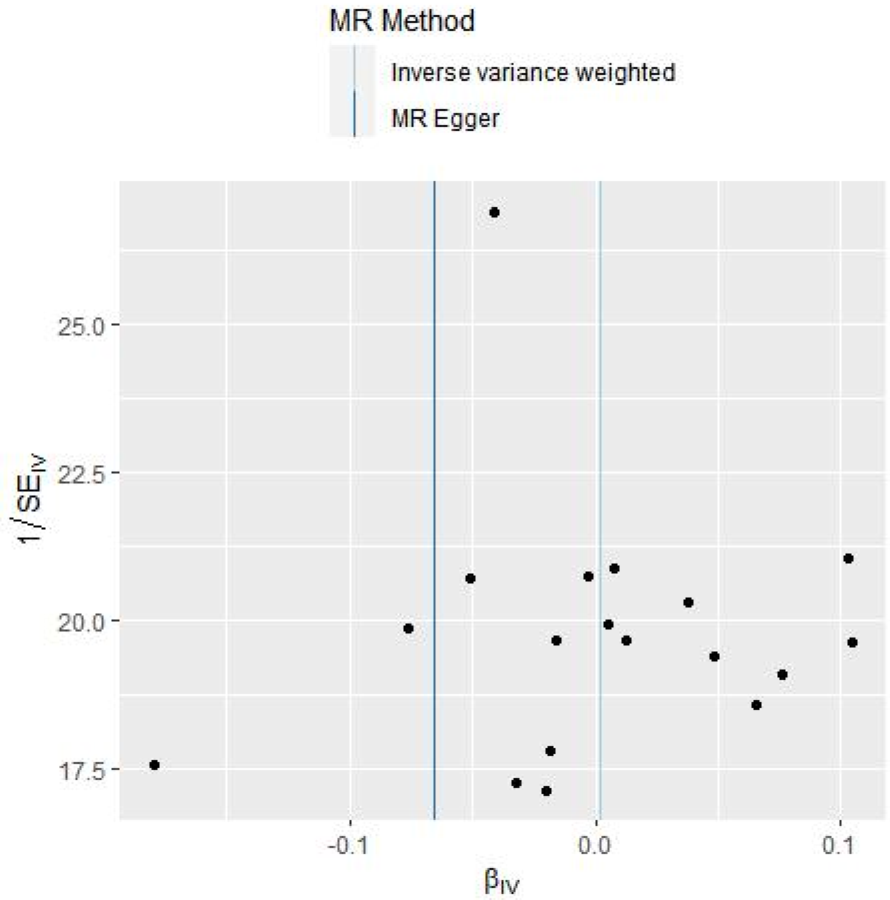

Figure 203: Leave-one-out plot to visualize causal effect of kynuremine on the risk of diabetes when leaving one SNP out.

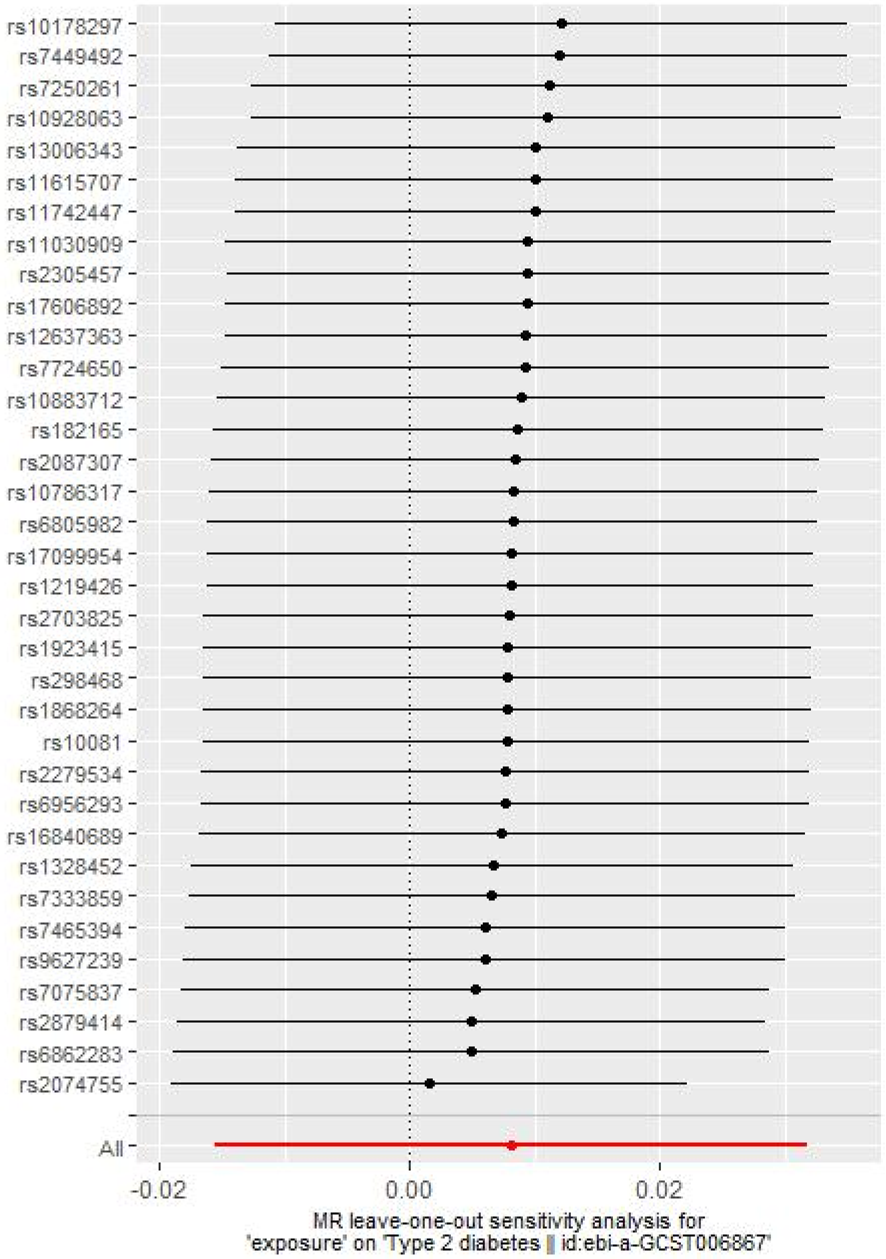

Figure 204: Funnel plots to visualize overall heterogeneity of Mendelian randomization (MR)

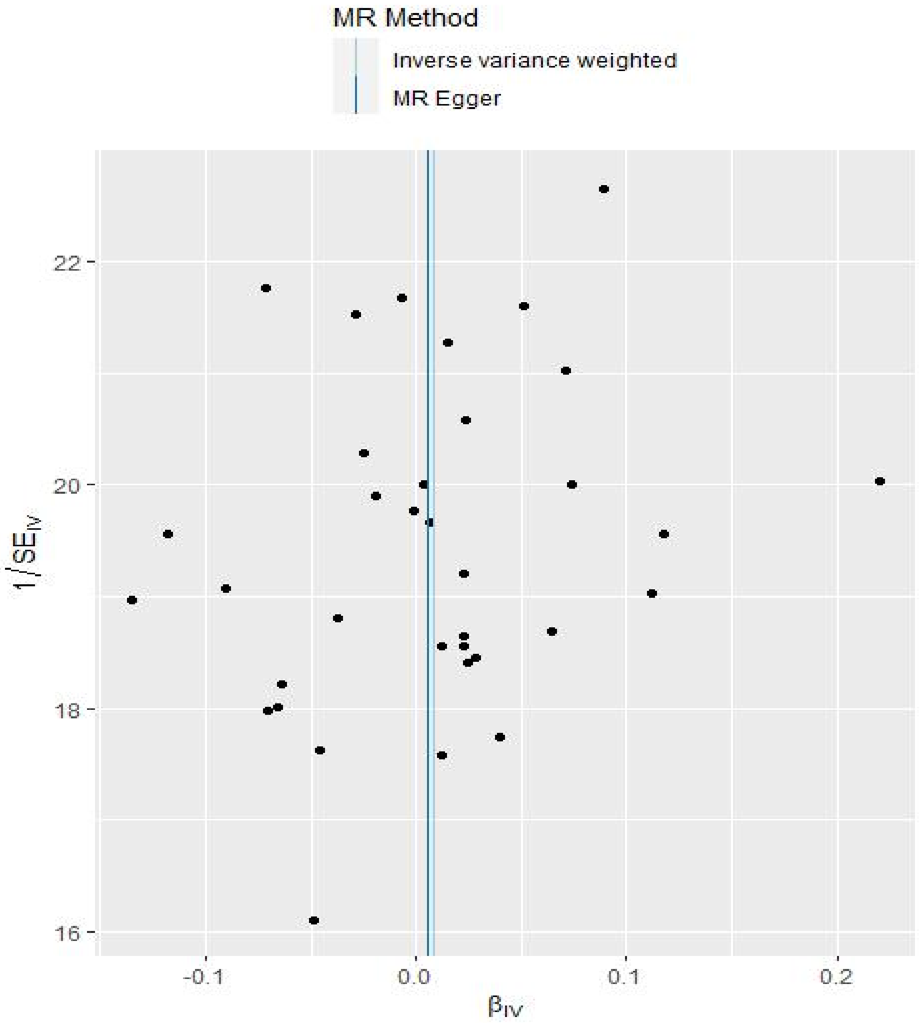

Figure 205: Leave-one-out plot to visualize causal effect of phenylalanine on the risk of diabetes when leaving one SNP out.

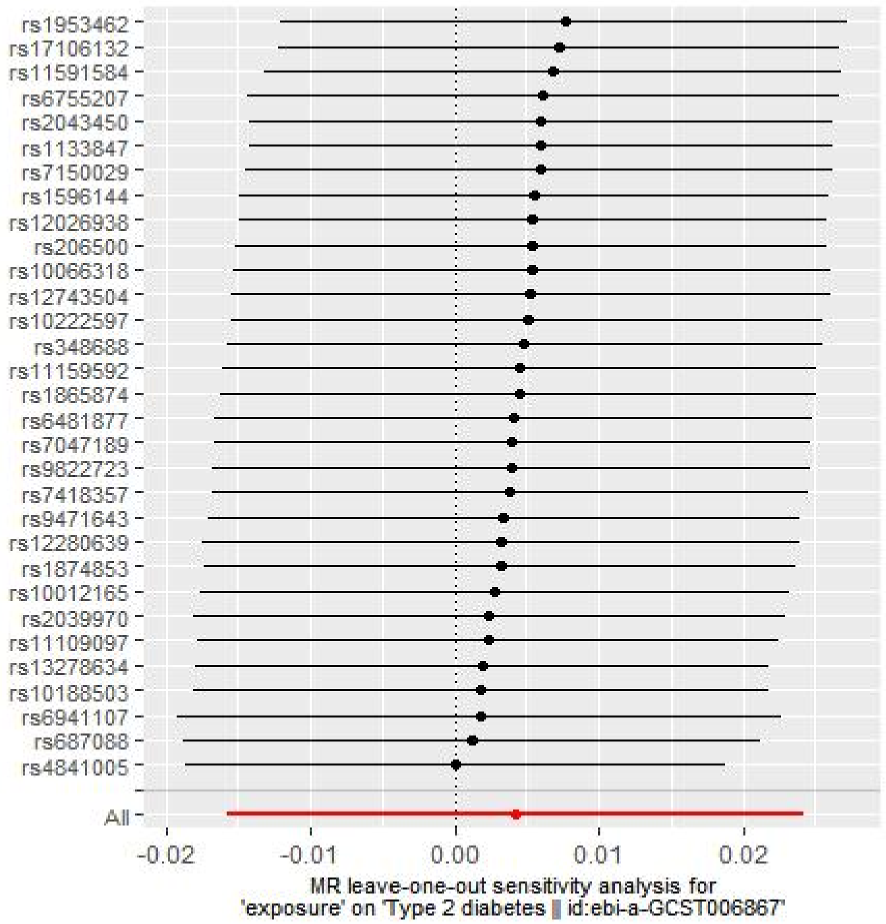

Figure 206: Funnel plots to visualize overall heterogeneity of Mendelian randomization (MR)

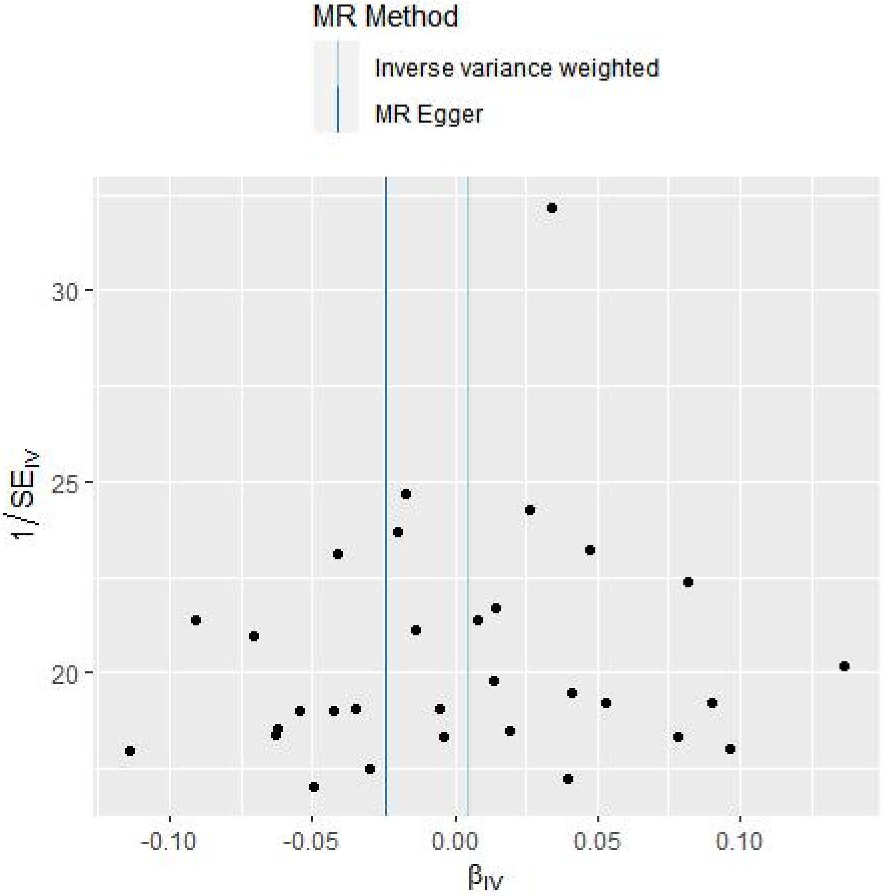

Figure 207: Leave-one-out plot to visualize causal effect of serotonin on the risk of diabetes when leaving one SNP out.

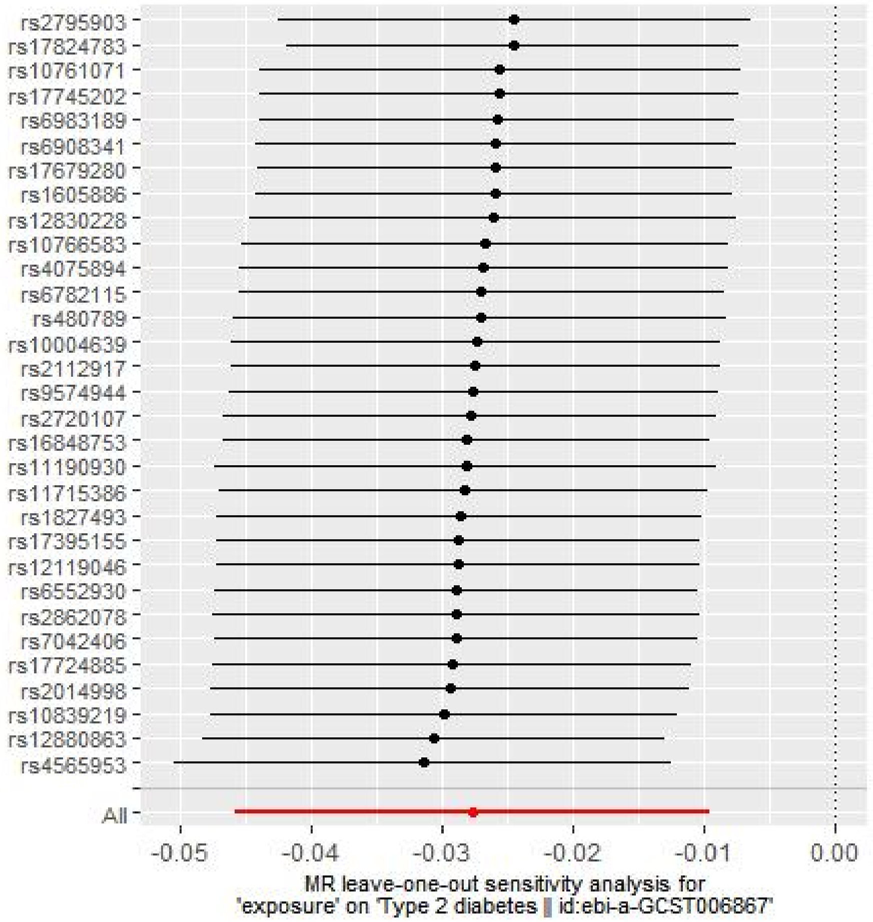

Figure 208: Funnel plots to visualize overall heterogeneity of Mendelian randomization (MR)

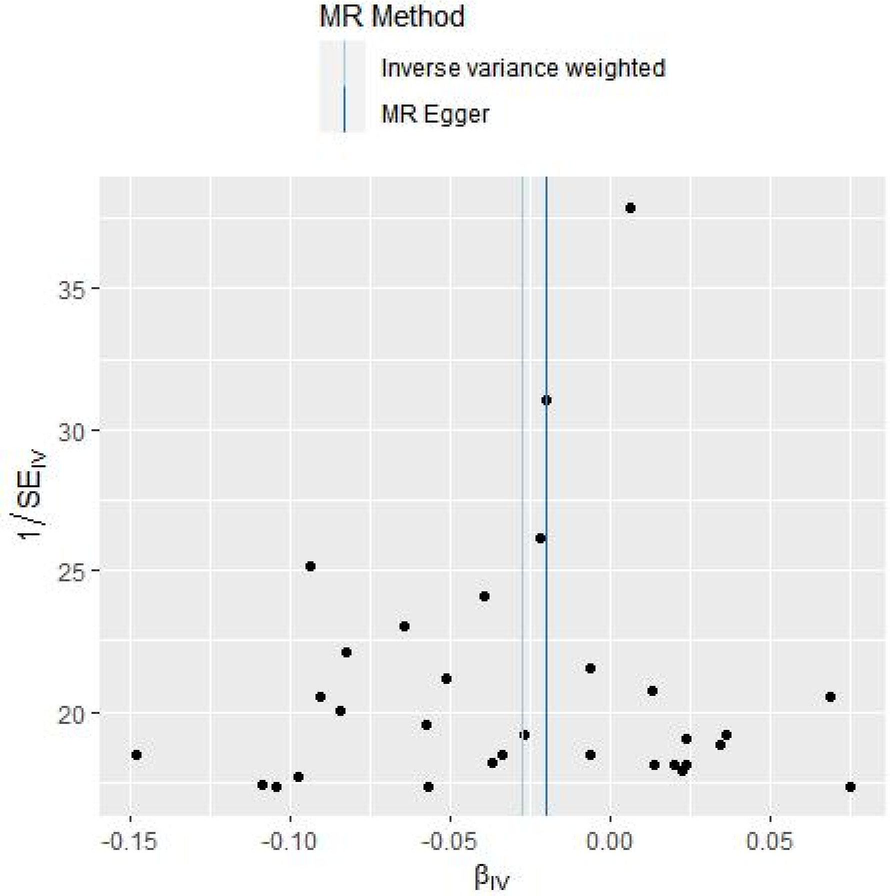

Figure 209: Leave-one-out plot to visualize causal effect of trimethylamine_N_oxide on the risk of diabetes disease when leaving one SNP out.

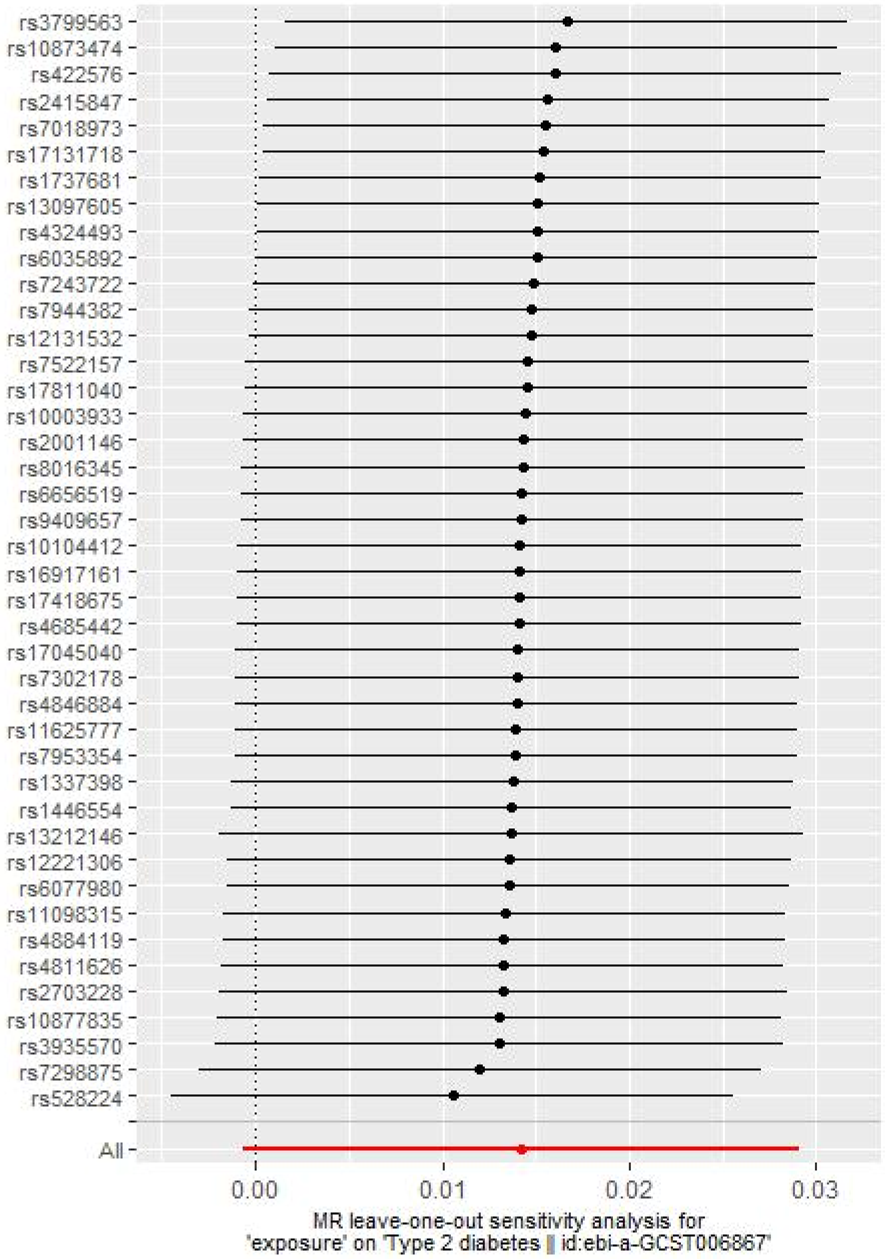

Figure 210: Funnel plots to visualize overall heterogeneity of Mendelian randomization (MR)

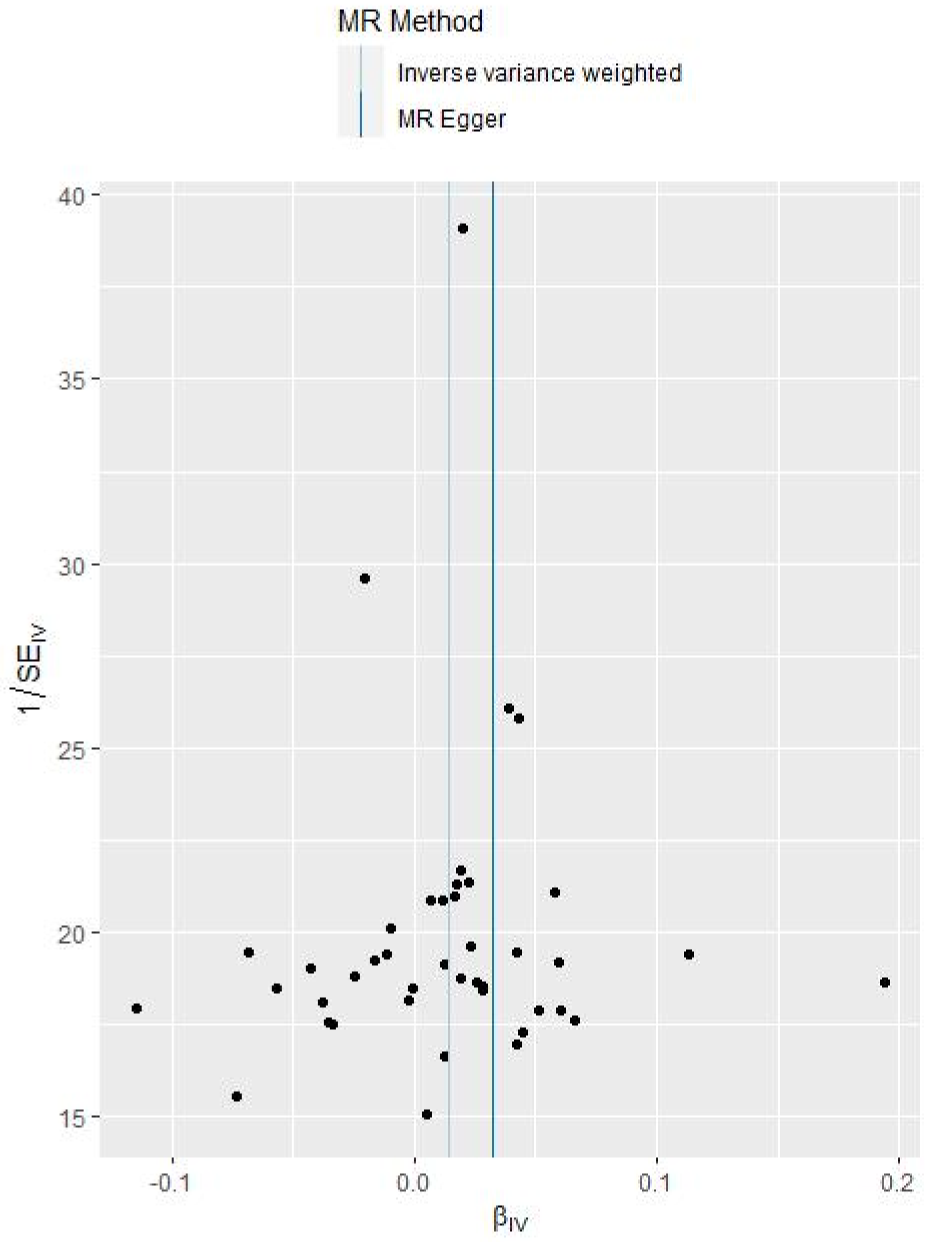

Figure 211: Leave-one-out plot to visualize causal effect of tryptophan on the risk of diabetes disease when leaving one SNP out.

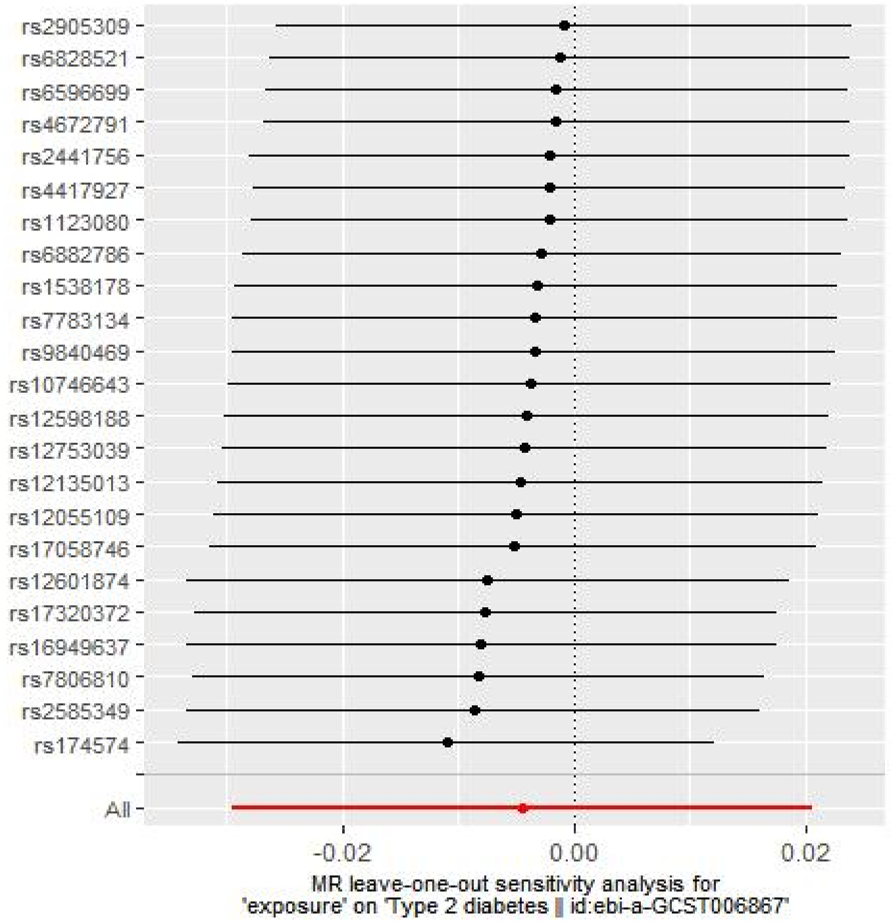

Figure 212: Funnel plots to visualize overall heterogeneity of Mendelian randomization (MR)

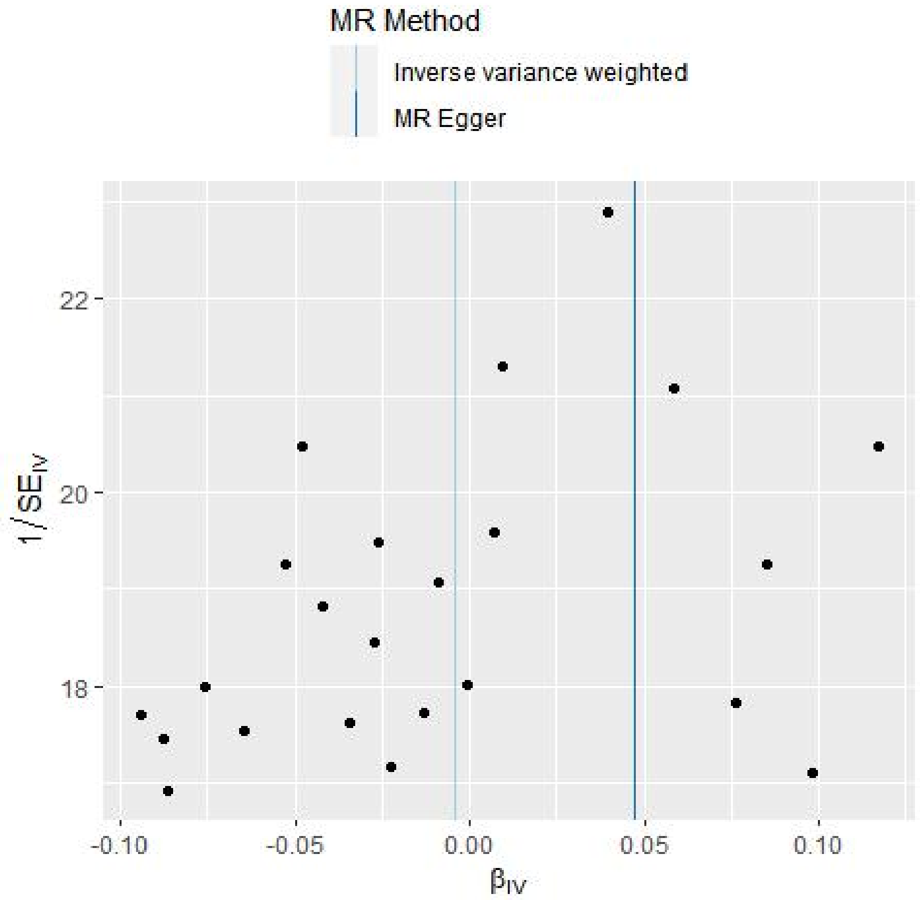

Figure 213: Leave-one-out plot to visualize causal effect of tyrosine on the risk of diabetes disease when leaving one SNP out.

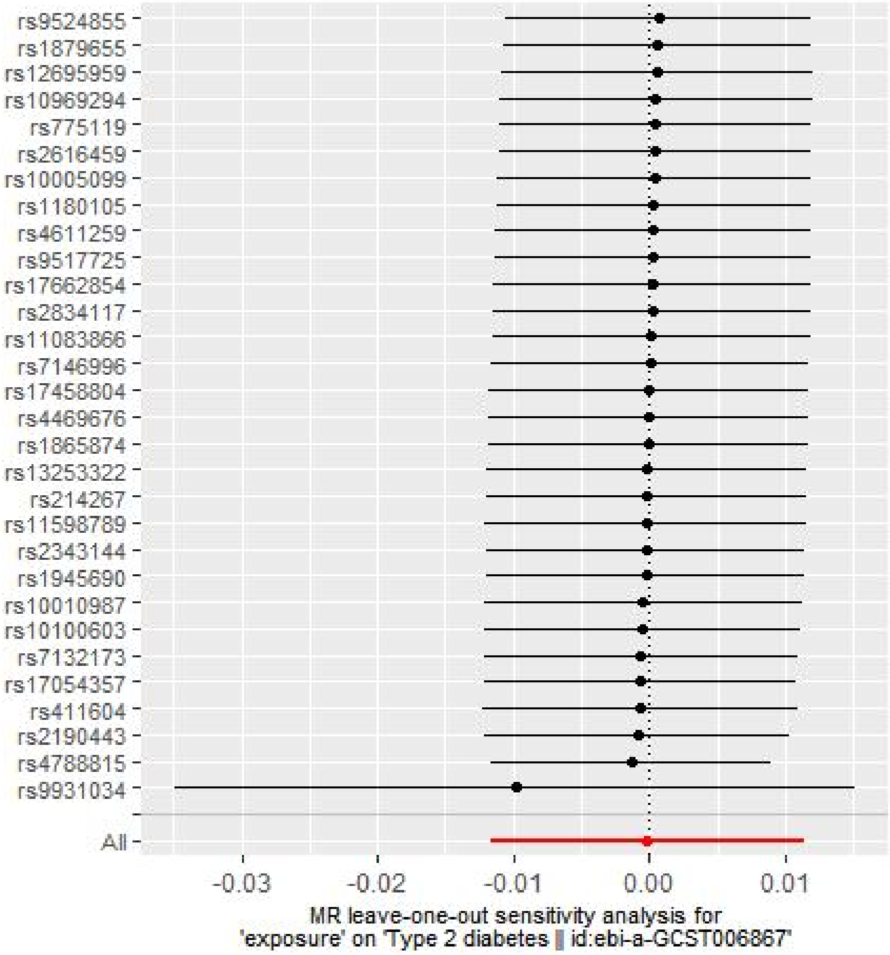

Figure 214: Funnel plots to visualize overall heterogeneity of Mendelian randomization (MR)

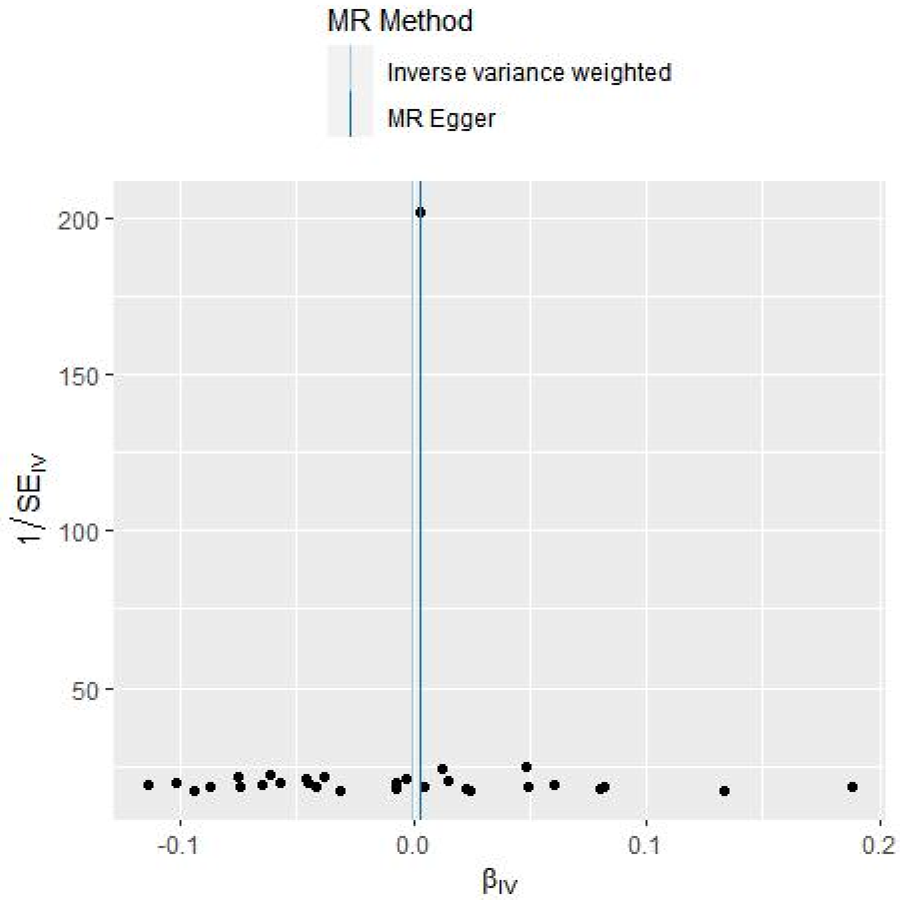

Figure 215: Leave-one-out plot to visualize causal effect of propionic acid on the risk of diabetes when leaving one SNP out.

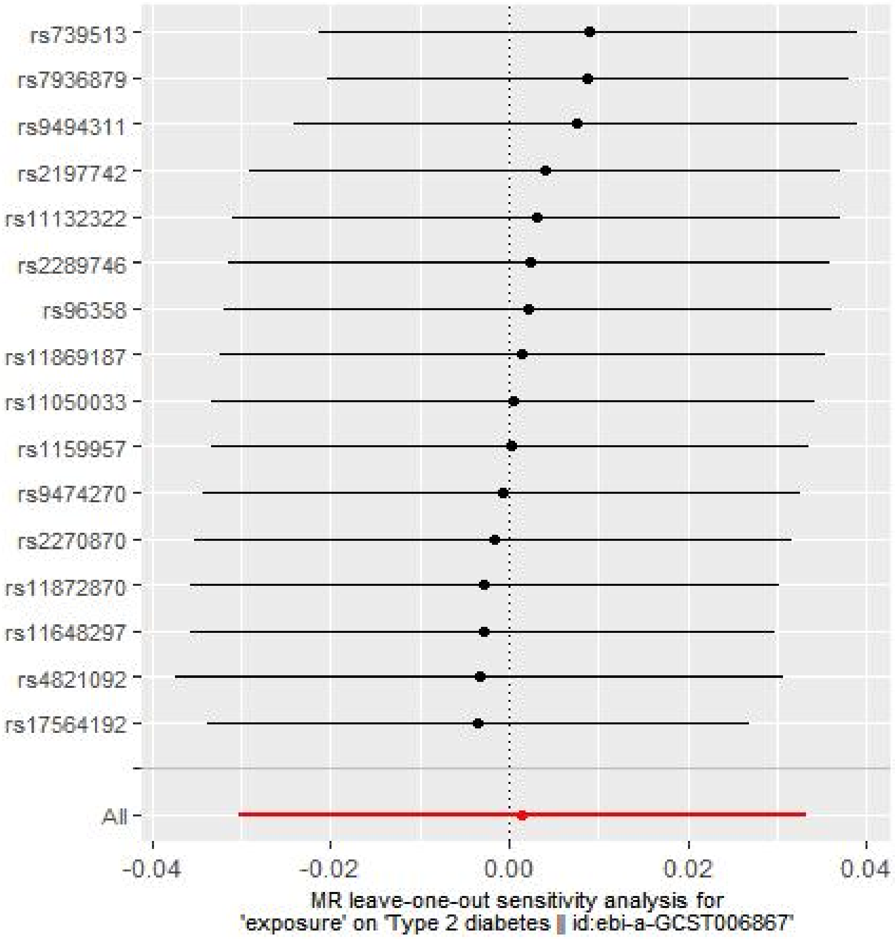

Figure 216: Funnel plots to visualize overall heterogeneity of Mendelian randomization (MR)

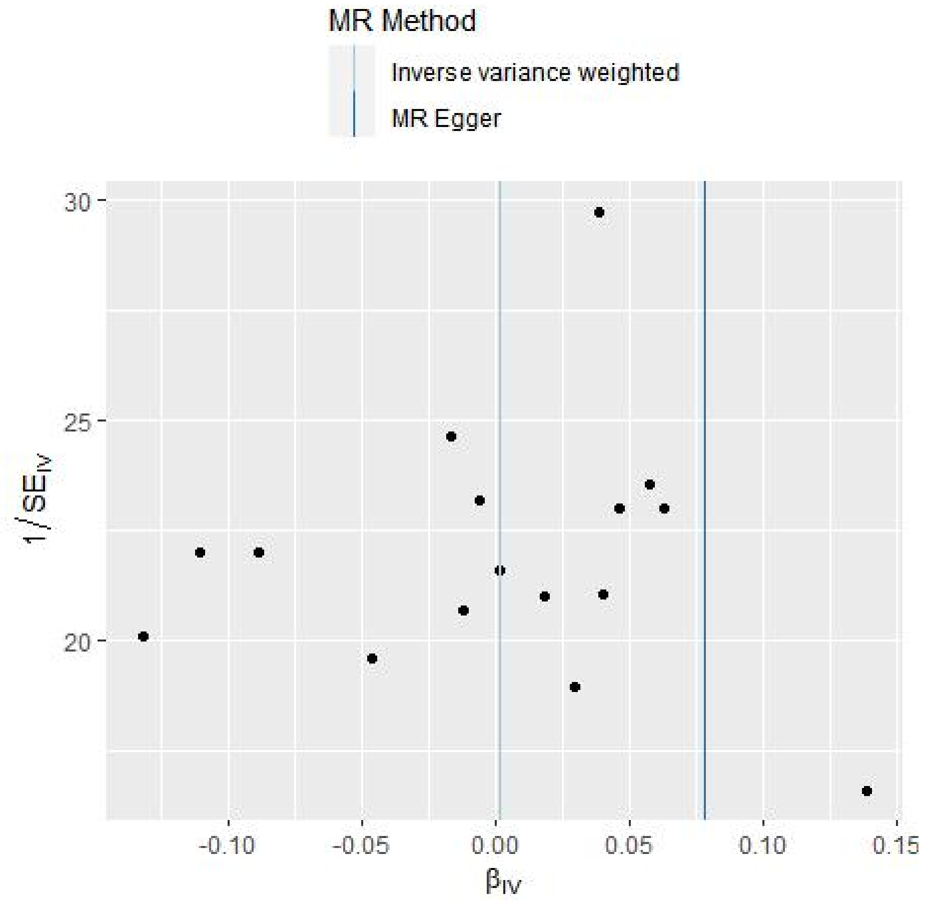

Figure 217: Leave-one-out plot to visualize causal effect of beta_hydroxybutyric acid on the risk of myocardial infarction when leaving one SNP out.

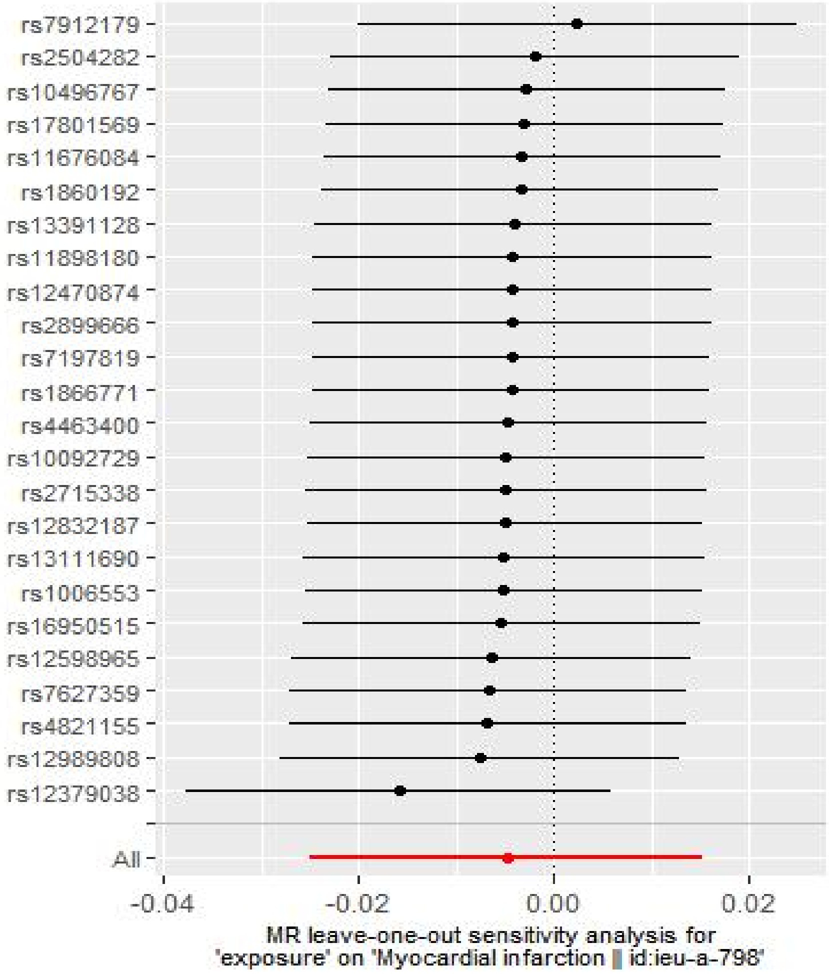

Figure 218: Funnel plots to visualize overall heterogeneity of Mendelian randomization (MR)

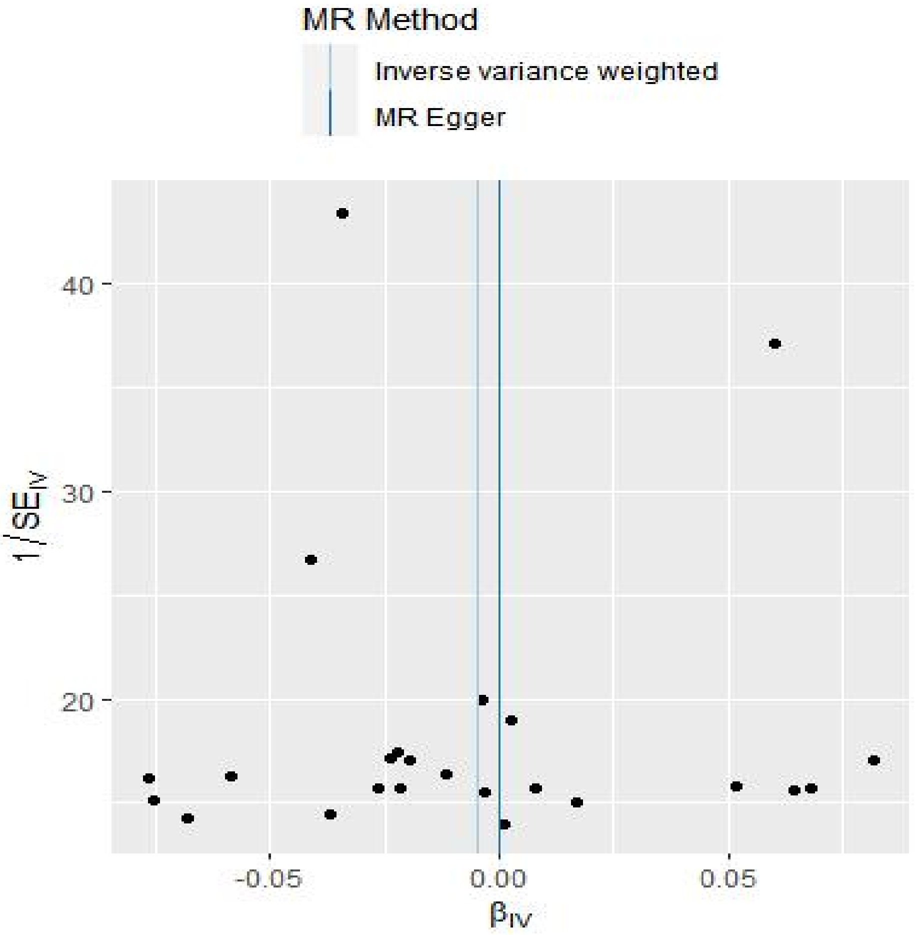

Figure 219: Leave-one-out plot to visualize causal effect of betaine on the risk of myocardial infarction when leaving one SNP out.

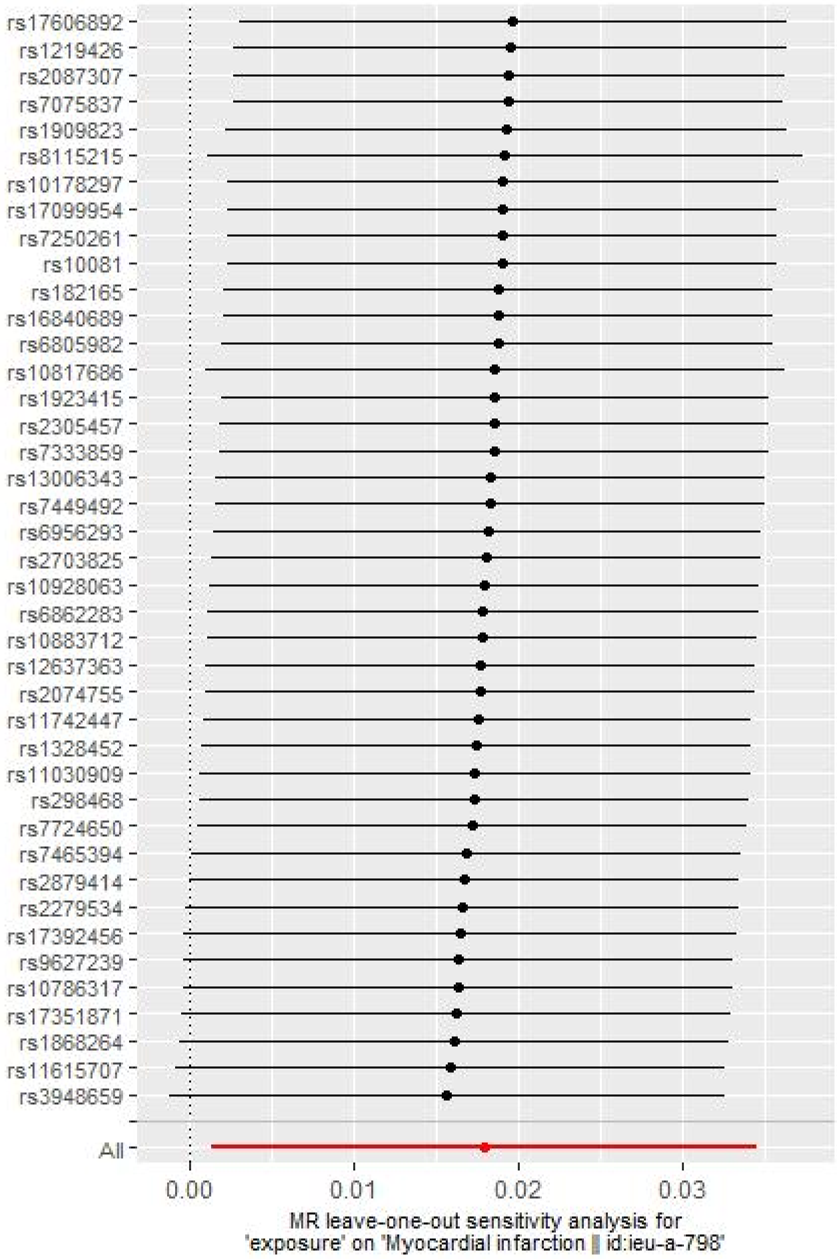

Figure 220: Funnel plots to visualize overall heterogeneity of Mendelian randomization (MR)

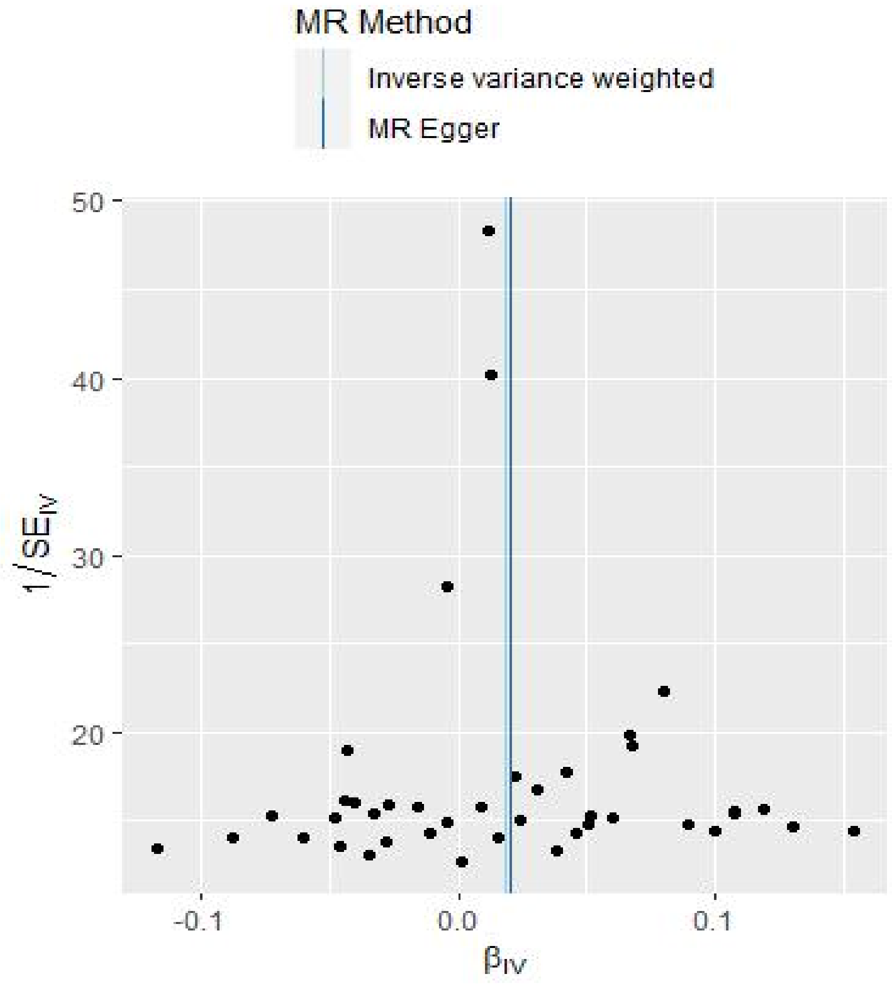

Figure 221: Leave-one-out plot to visualize causal effect of carnitine on the risk of myocardial infarction when leaving one SNP out.

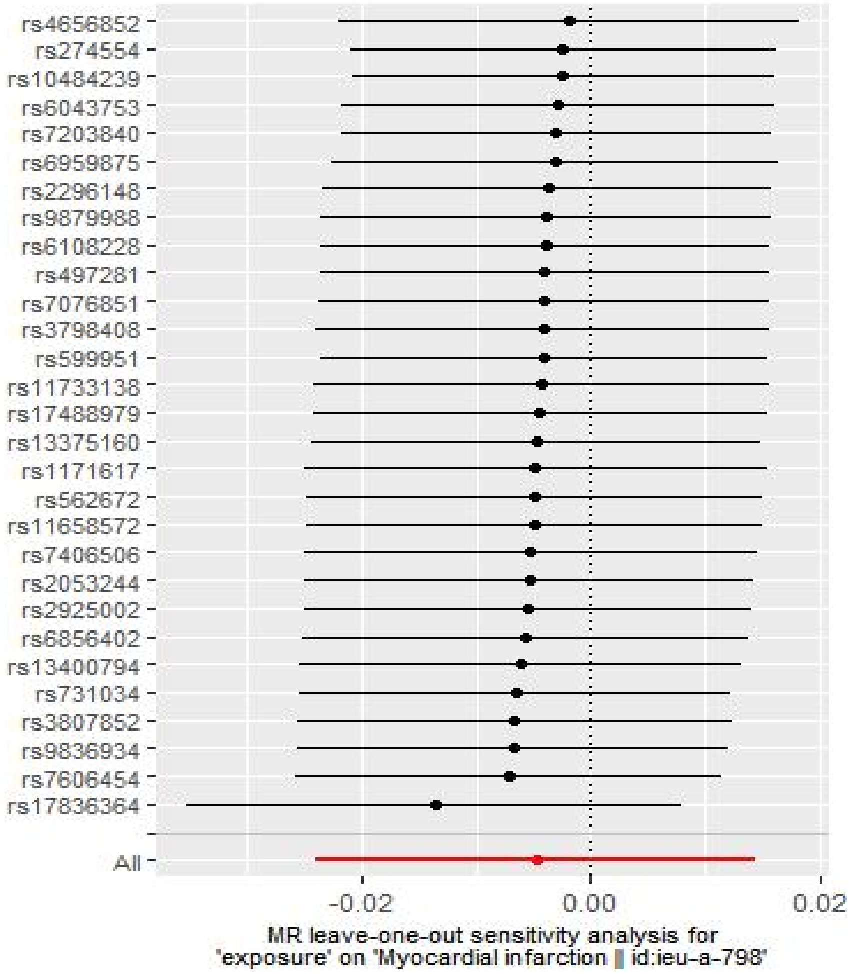

Figure 222: Funnel plots to visualize overall heterogeneity of Mendelian randomization (MR)

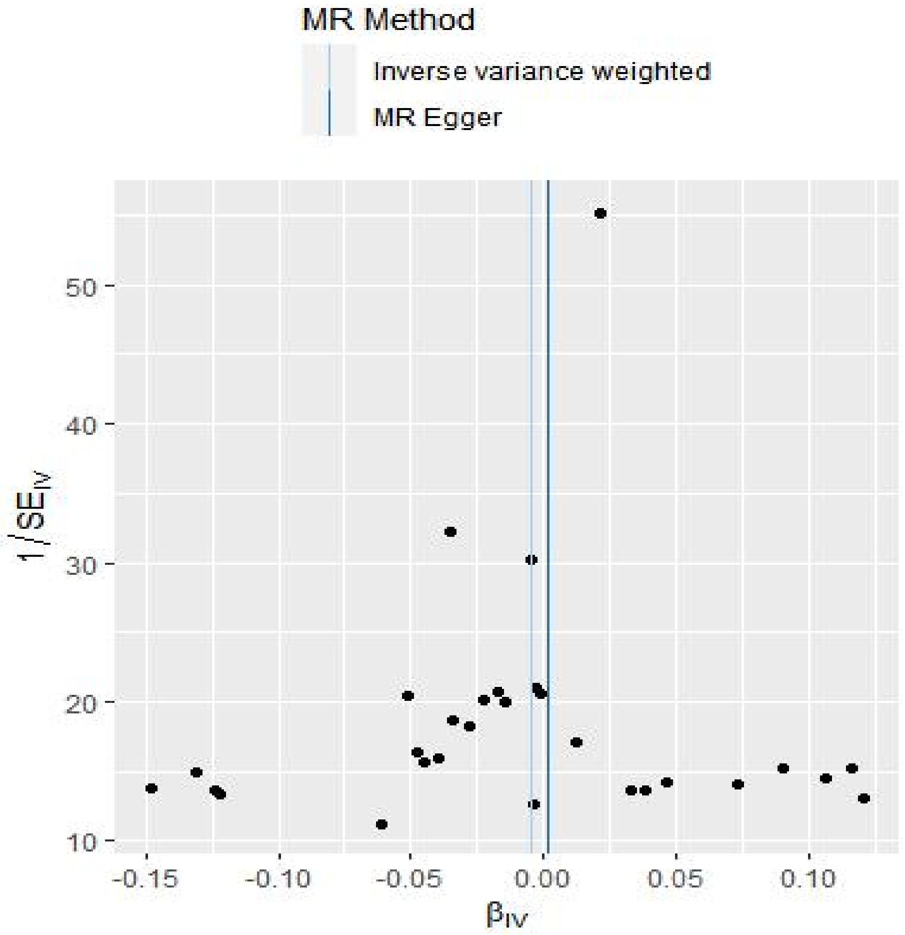

Figure 223: Leave-one-out plot to visualize causal effect of choline on the risk of myocardial infarction when leaving one SNP out.

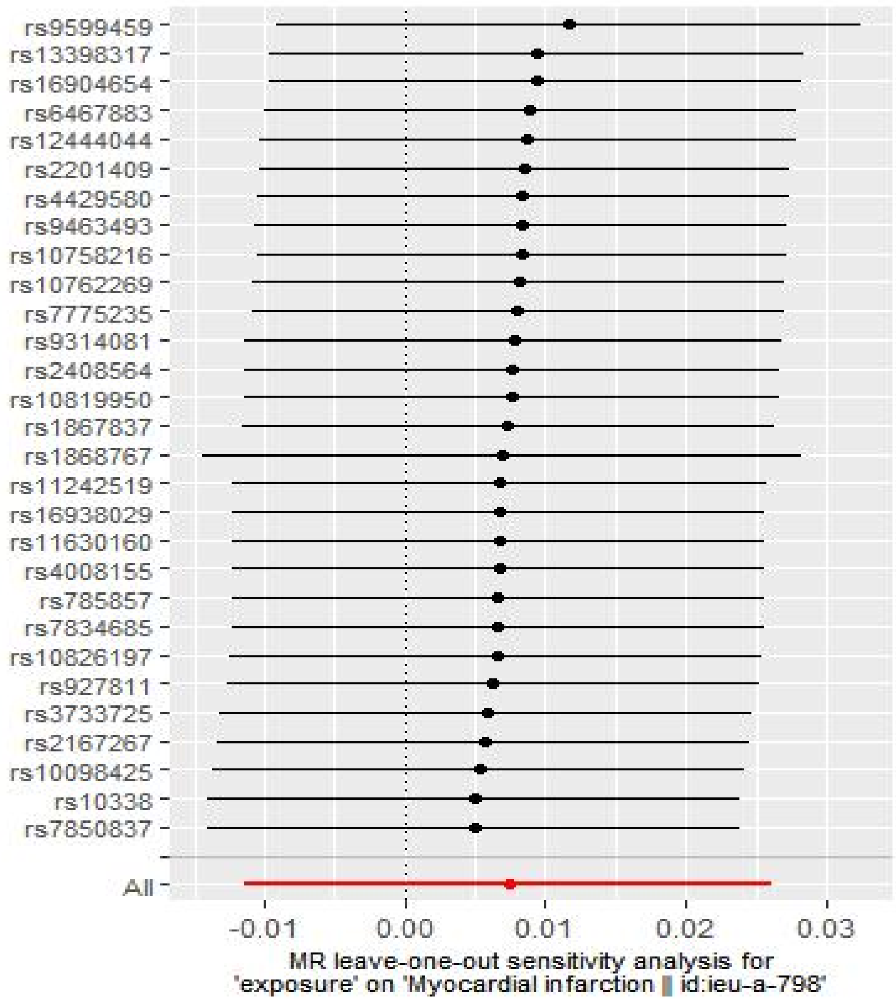

Figure 224: Funnel plots to visualize overall heterogeneity of Mendelian randomization (MR)

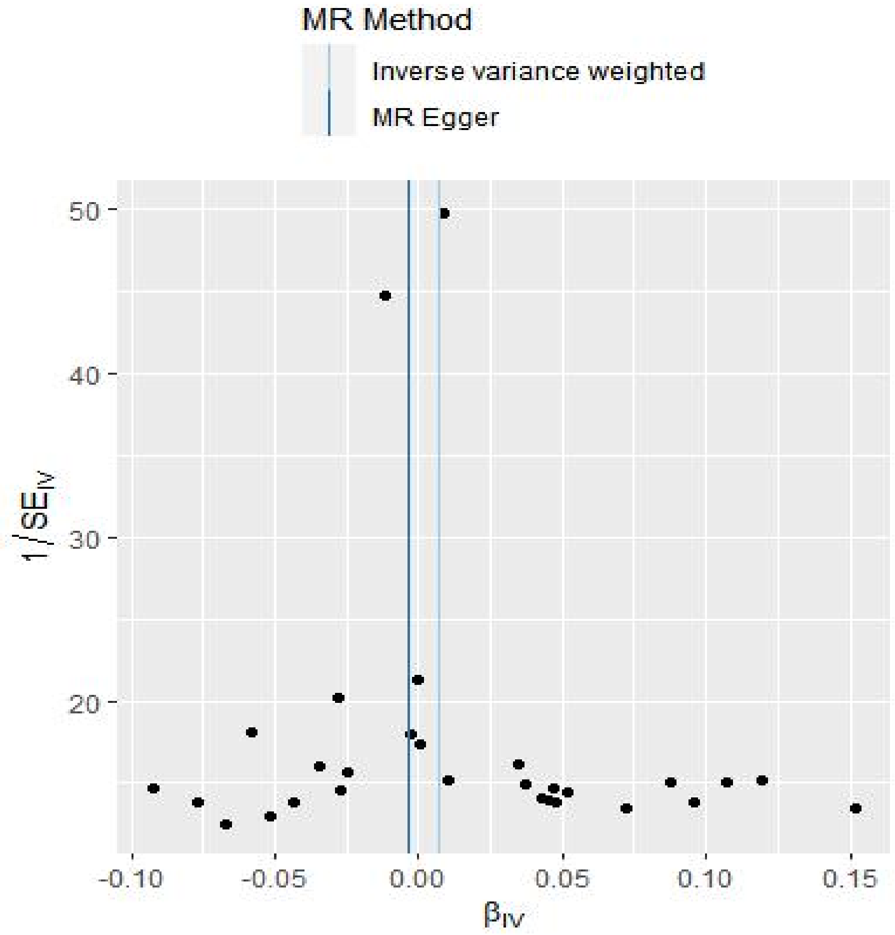

Figure 225: Leave-one-out plot to visualize causal effect of glutamate on the risk of myocardial infarction when leaving one SNP out.

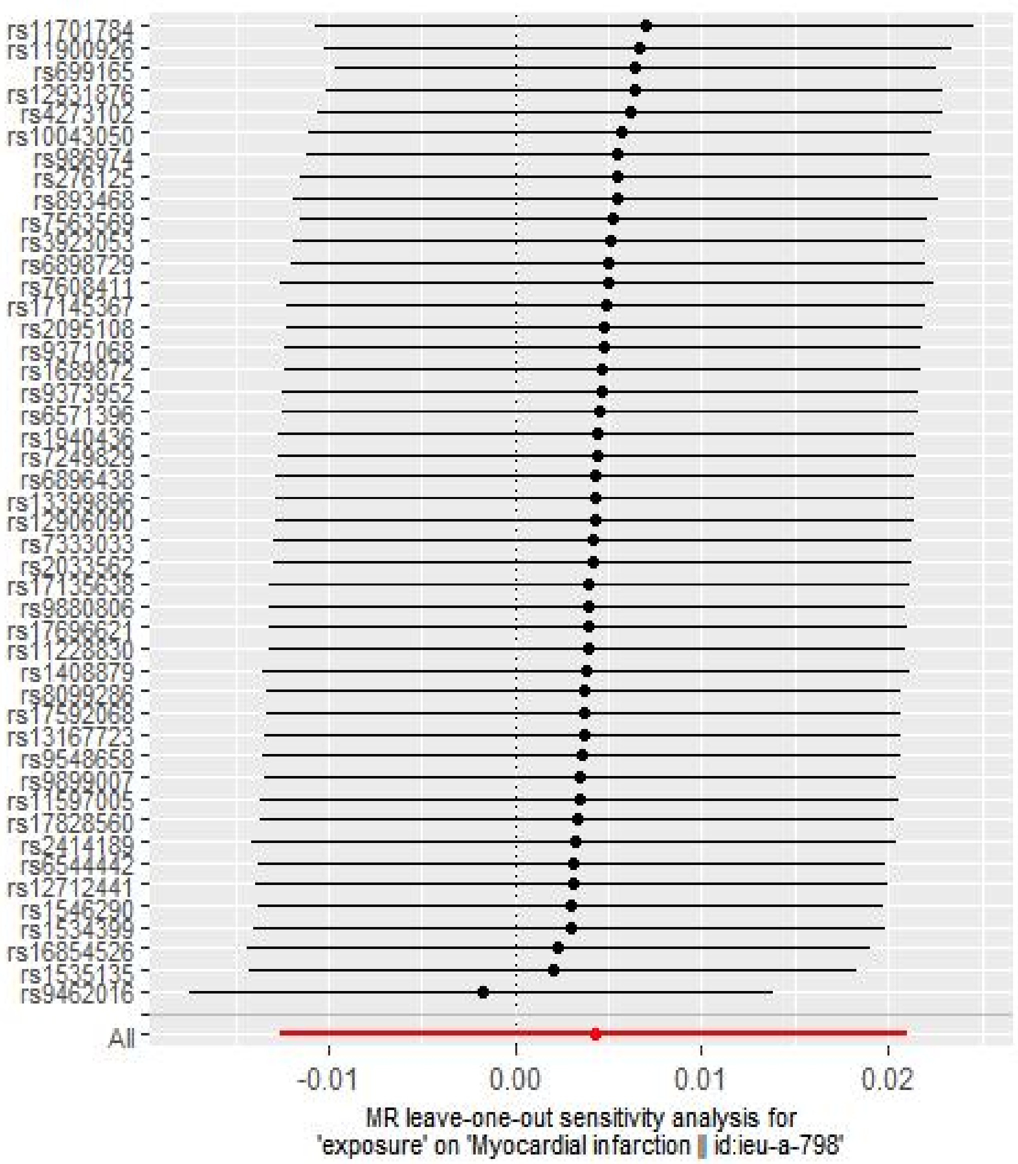

Figure 226: Funnel plots to visualize overall heterogeneity of Mendelian randomization (MR)

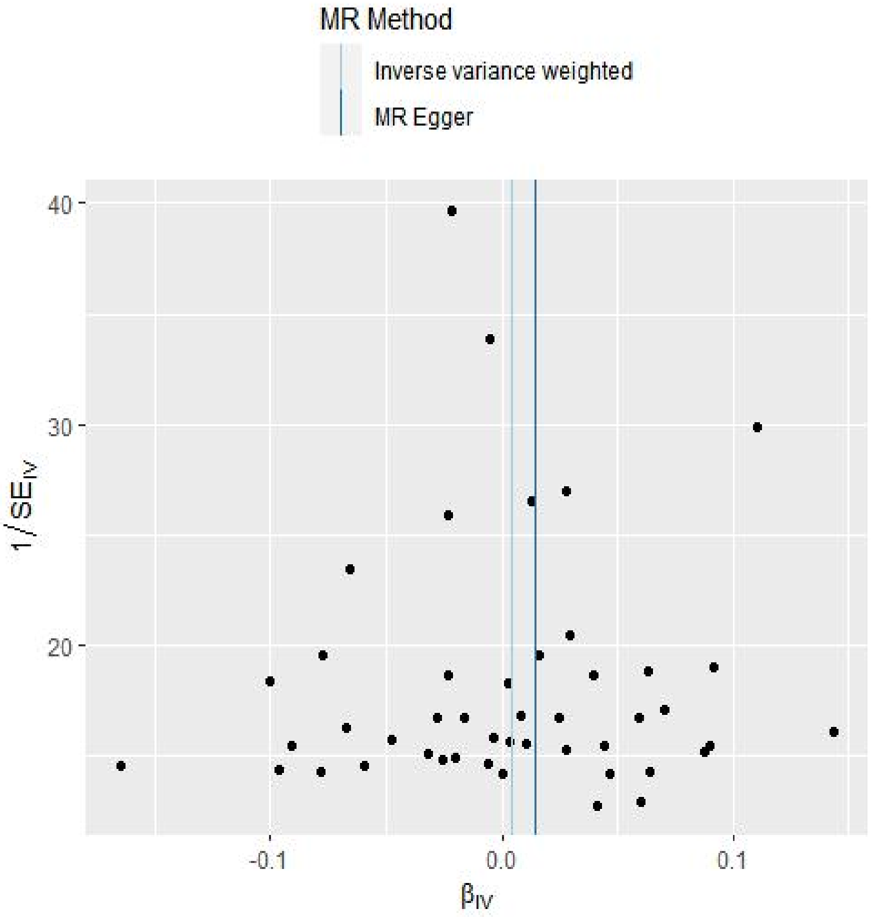

Figure 227: Leave-one-out plot to visualize causal effect of kynuremine on the risk of myocardial infarction when leaving one SNP out.

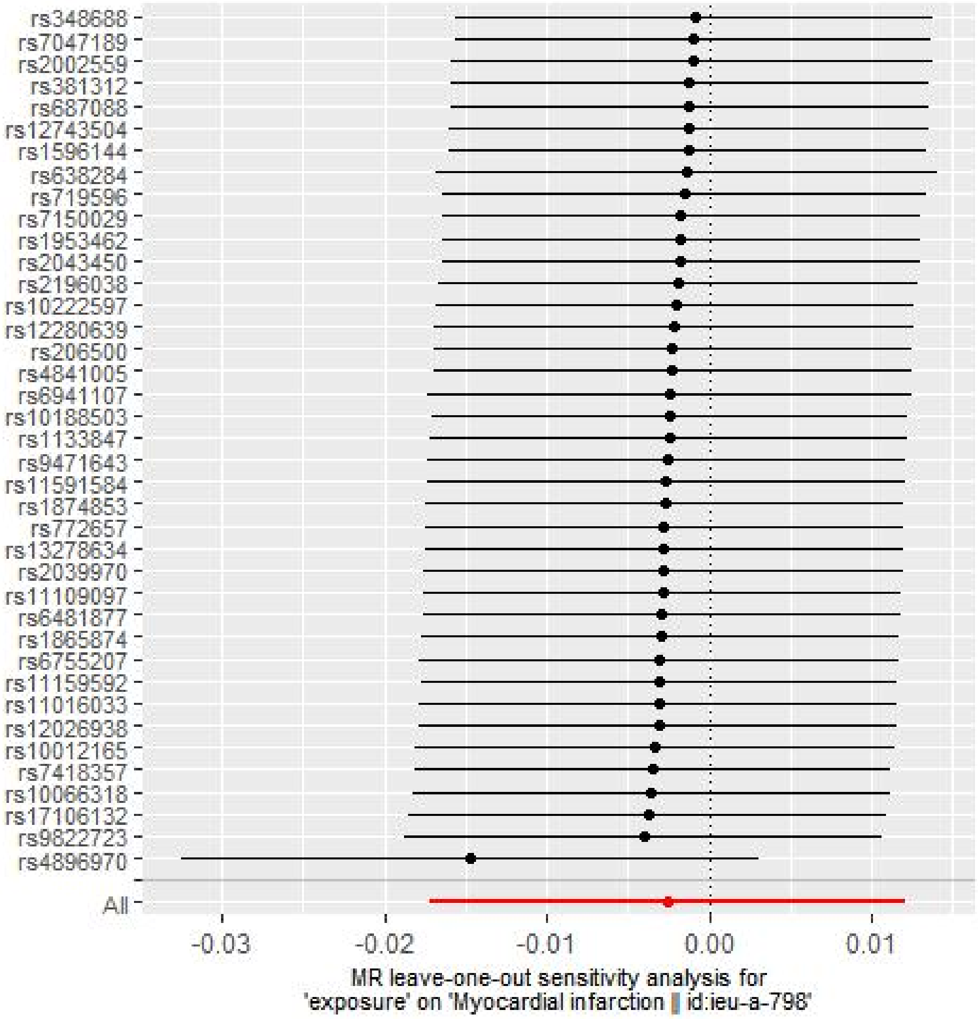

Figure 228: Funnel plots to visualize overall heterogeneity of Mendelian randomization (MR)

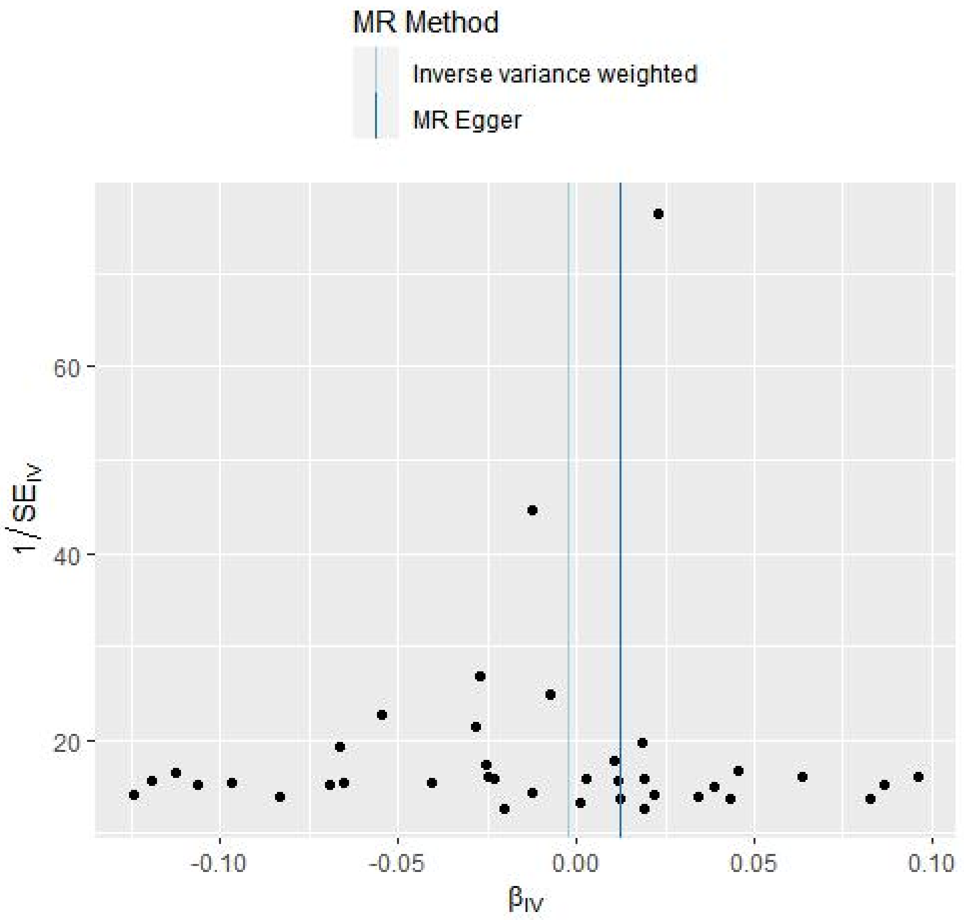

Figure 229: Leave-one-out plot to visualize causal effect of phenylalanine on the risk of myocardial infarction when leaving one SNP out.

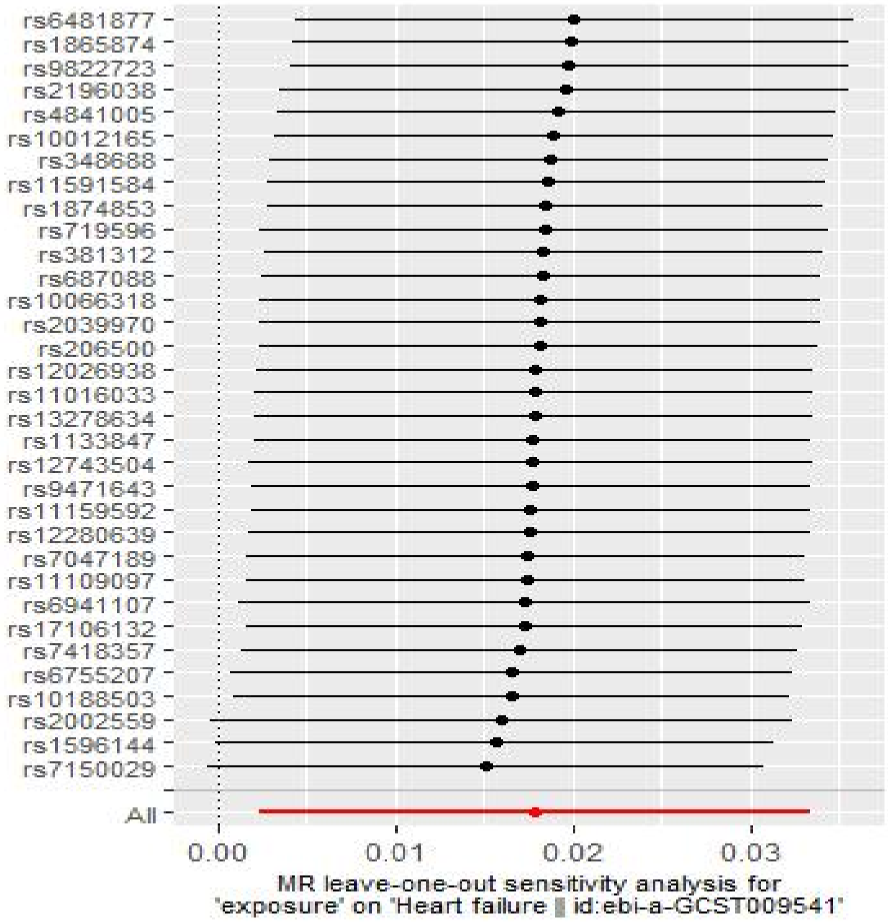

Figure 230: Funnel plots to visualize overall heterogeneity of Mendelian randomization (MR)

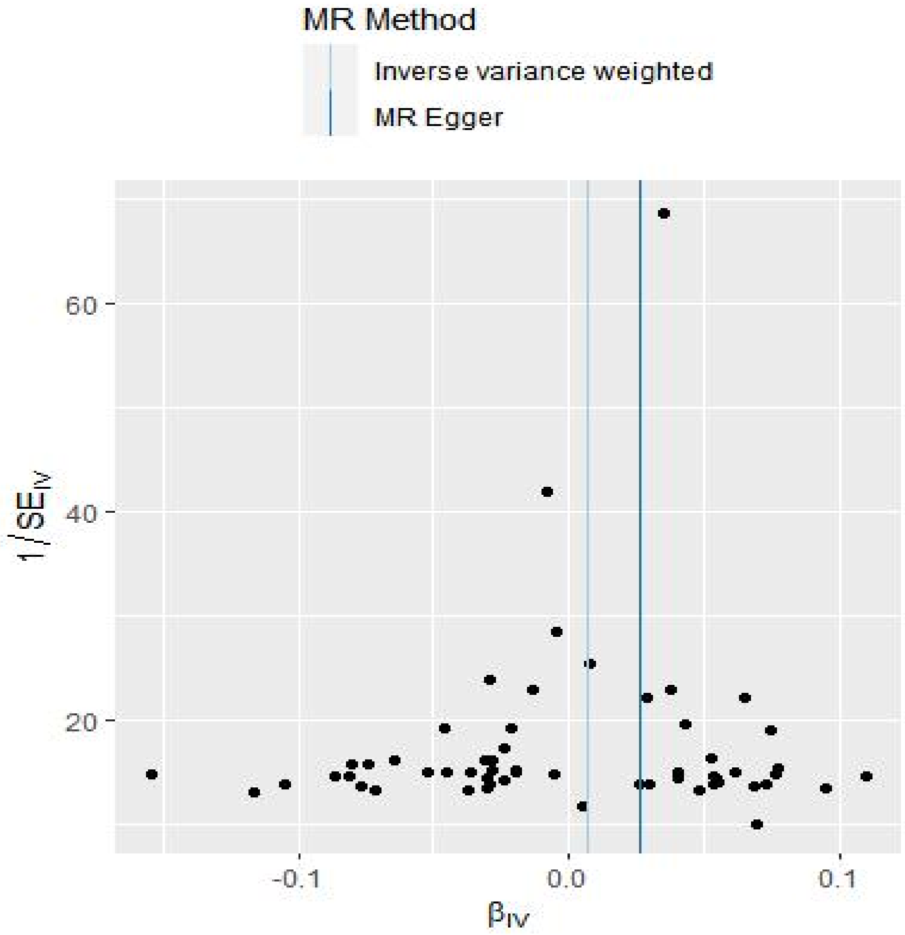

Figure 231: Leave-one-out plot to visualize causal effect of serotonin on the risk of myocardial infarction when leaving one SNP out.

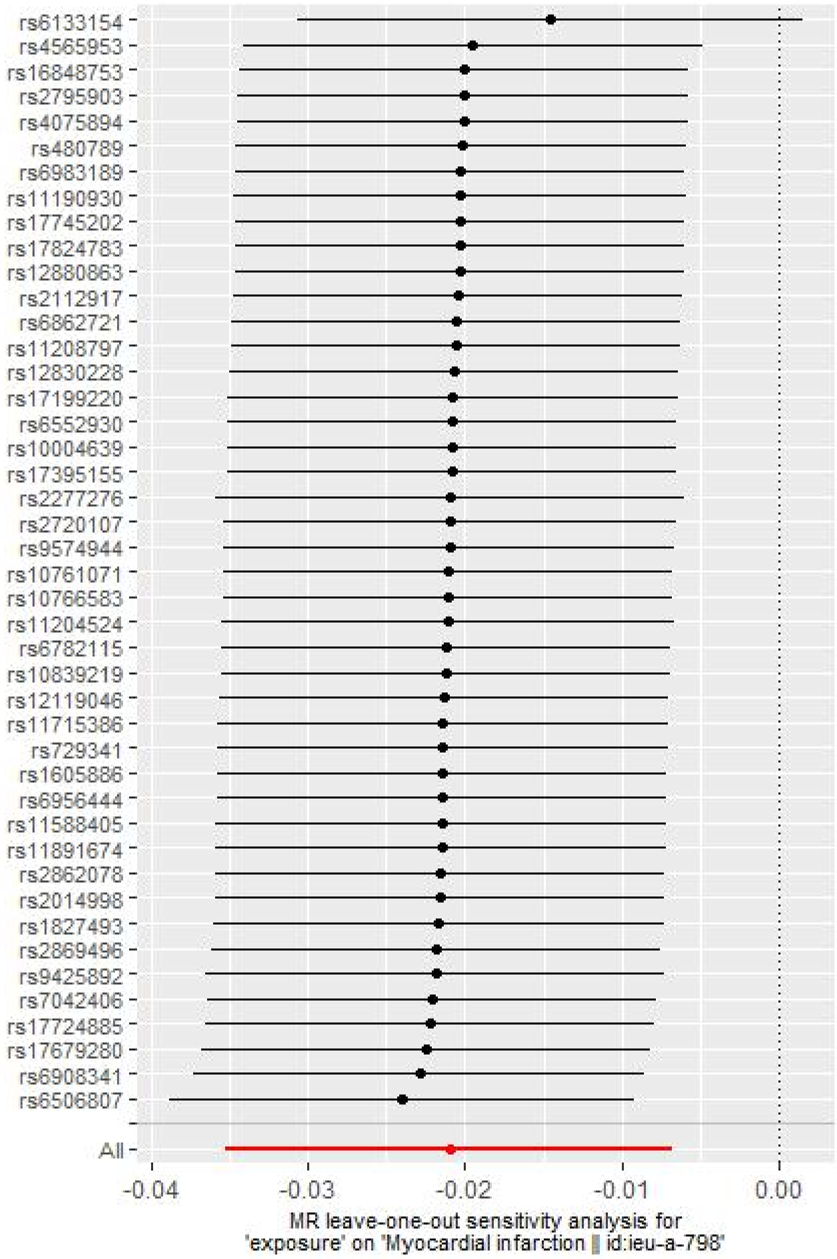

Figure 232: Funnel plots to visualize overall heterogeneity of Mendelian randomization (MR)

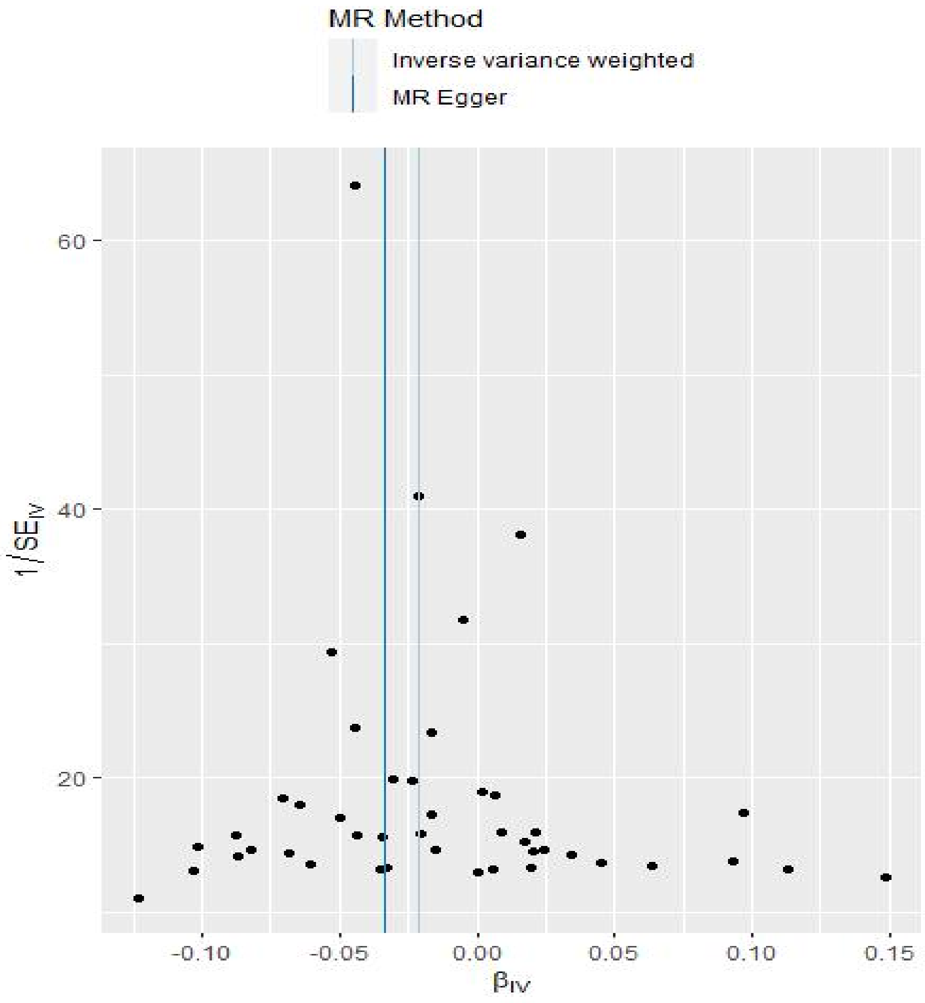

Figure 233: Leave-one-out plot to visualize causal effect of trimethylamine_N_oxide on the risk of myocardial infarction when leaving one SNP out.

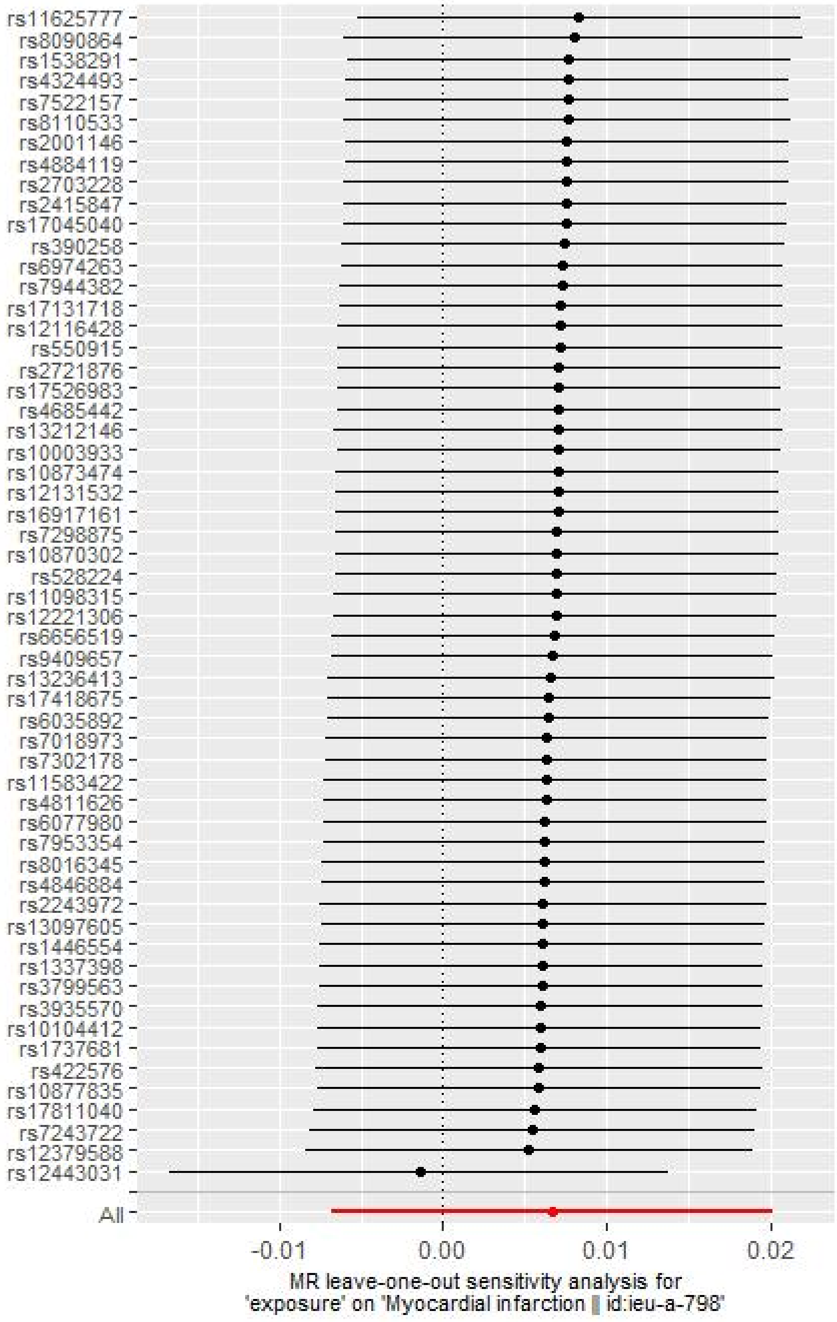

Figure 234: Funnel plots to visualize overall heterogeneity of Mendelian randomization (MR)

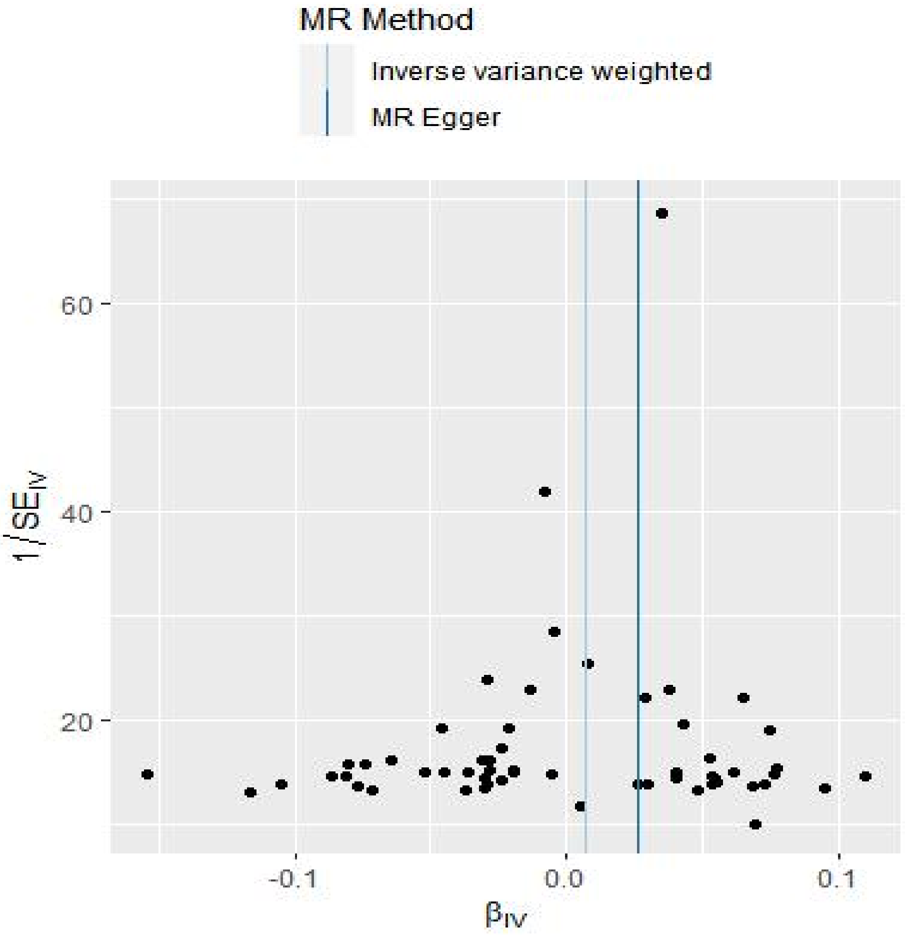

Figure 235: Leave-one-out plot to visualize causal effect of tryptophan on the risk of myocardial infarction when leaving one SNP out.

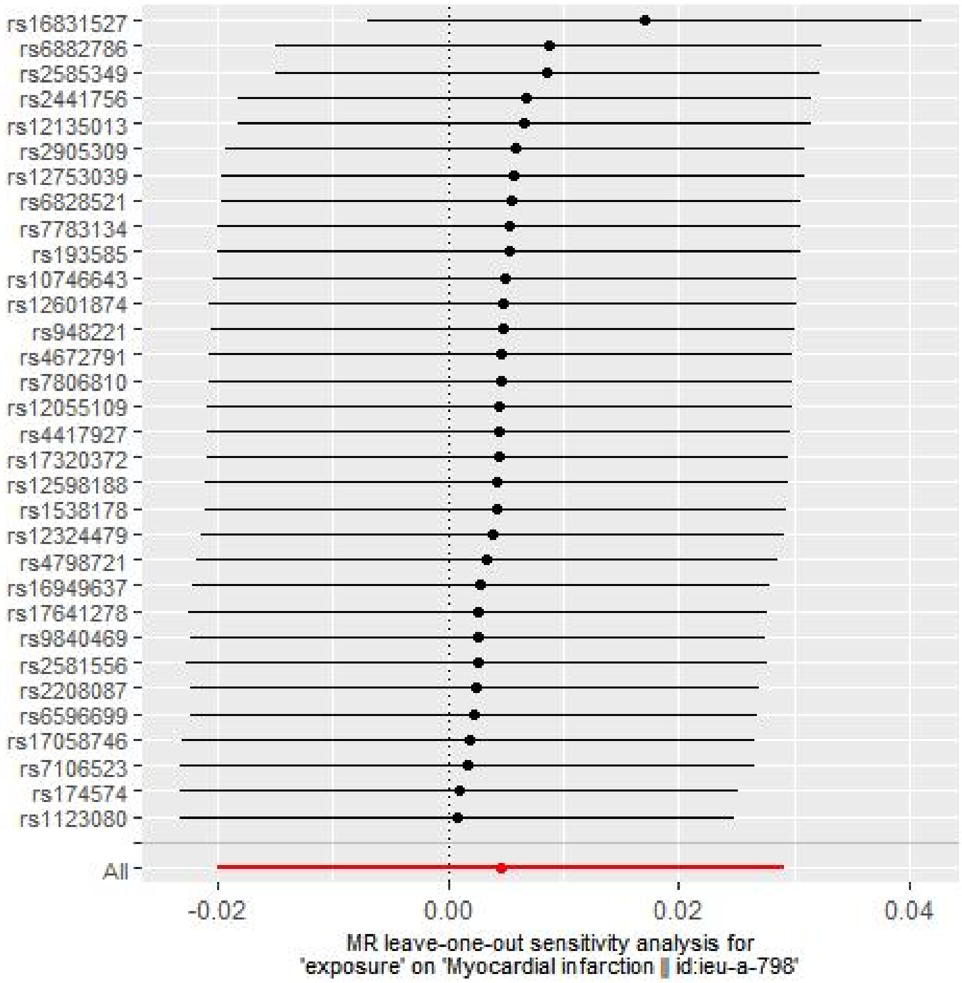

Figure 236: Funnel plots to visualize overall heterogeneity of Mendelian randomization (MR)

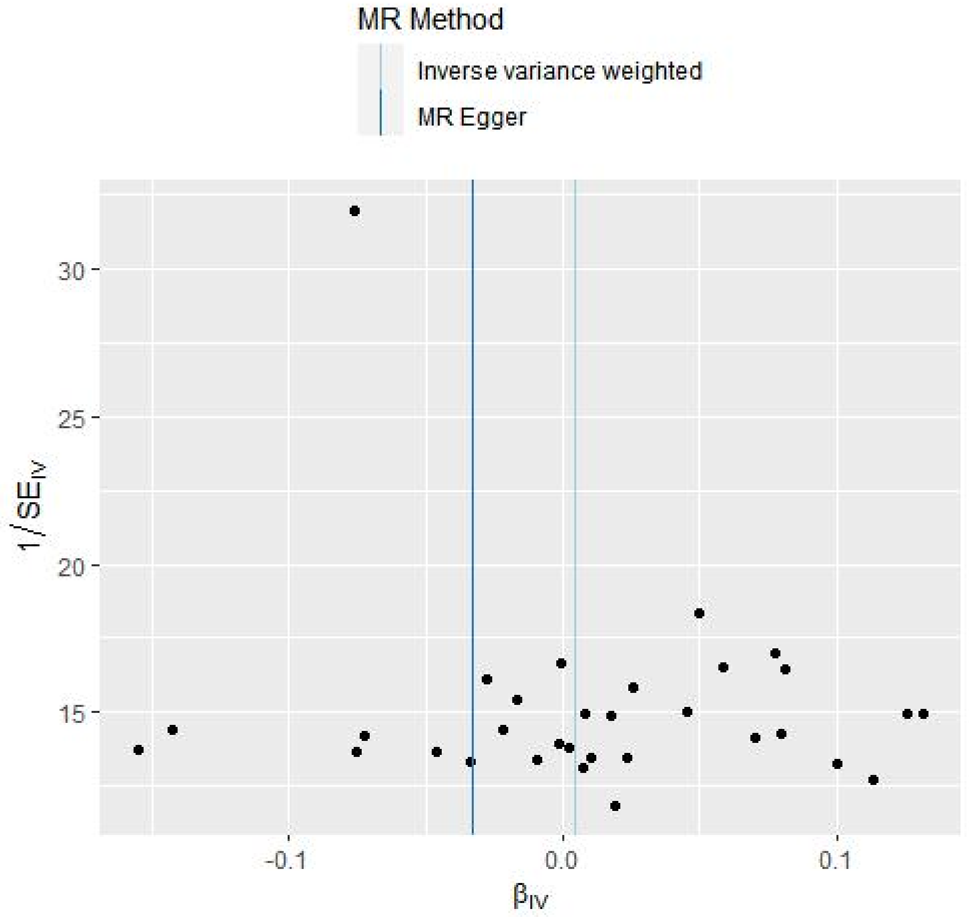

Figure 237: Leave-one-out plot to visualize causal effect of tyrosine on the risk of myocardial infarction when leaving one SNP out.

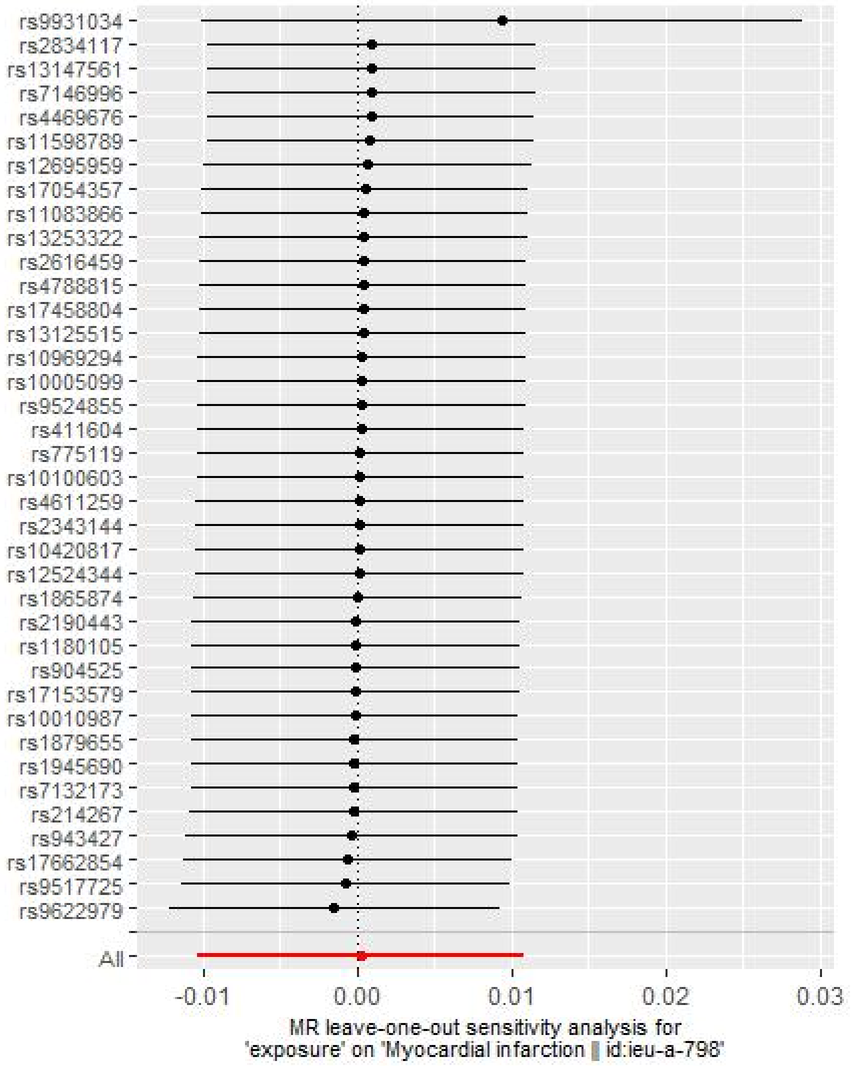

Figure 238: Funnel plots to visualize overall heterogeneity of Mendelian randomization (MR)

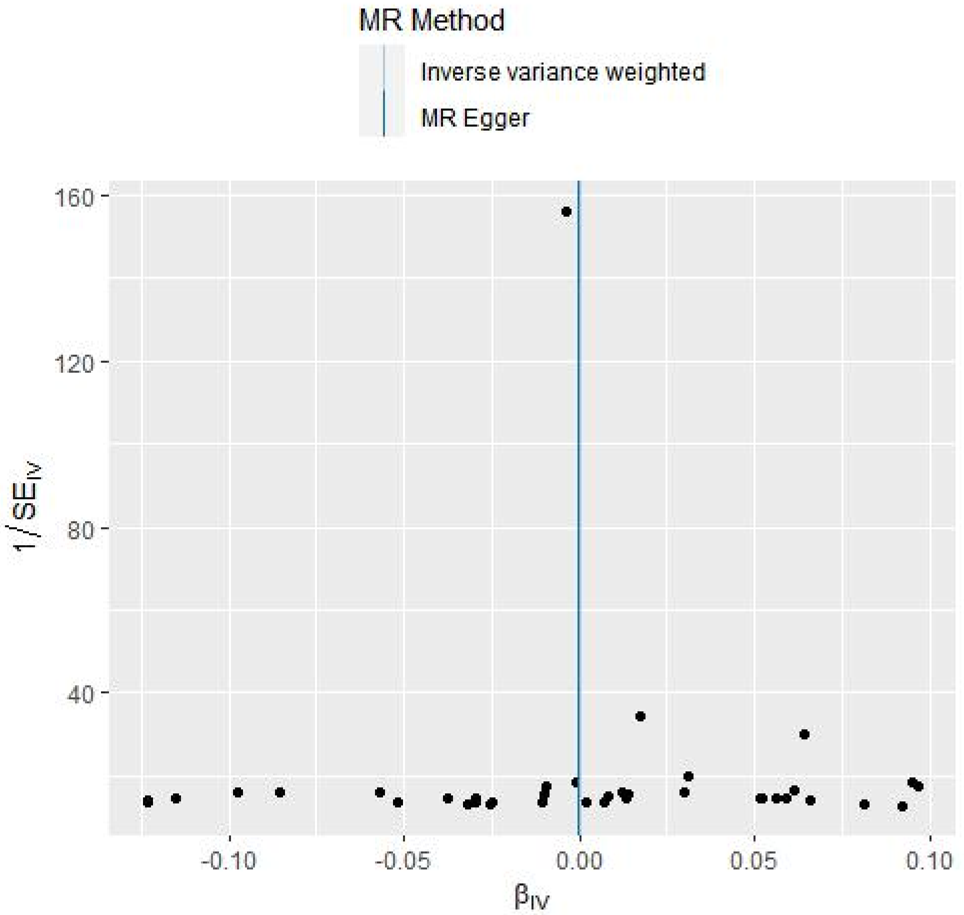

Figure 239: Leave-one-out plot to visualize causal effect of propionic acid on the risk of myocardial infarction when leaving one SNP out.

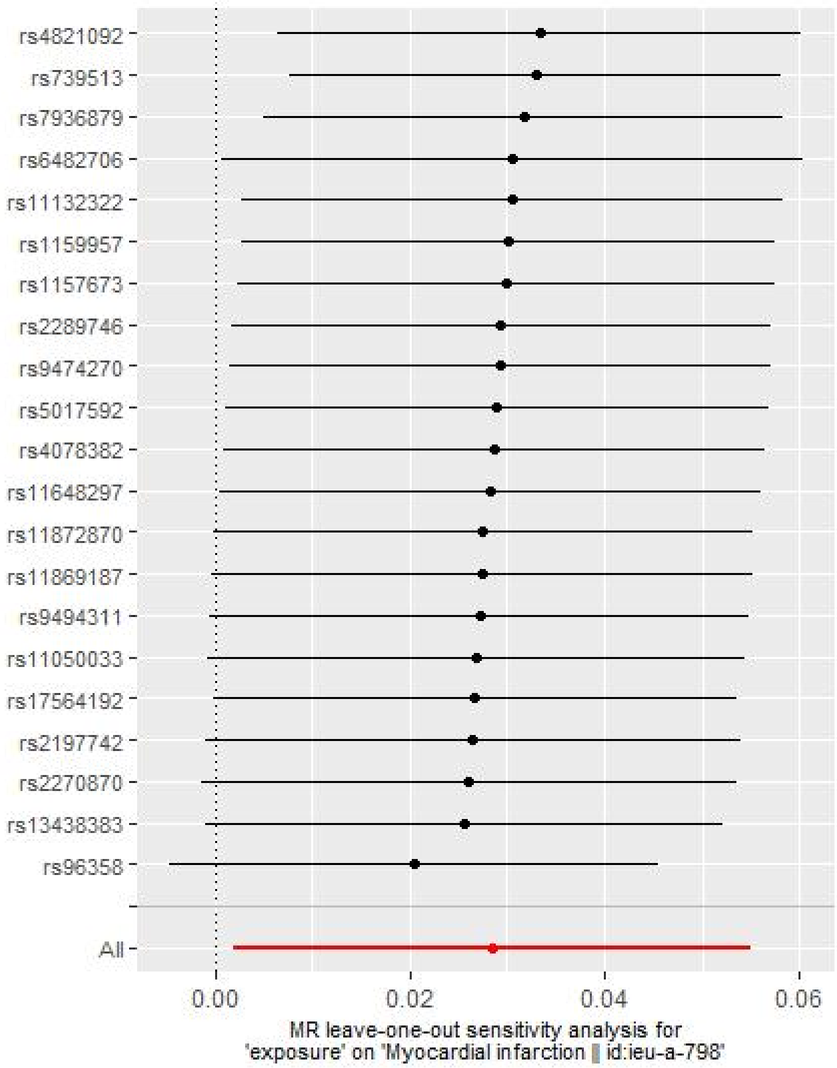

Figure 240: Funnel plots to visualize overall heterogeneity of Mendelian randomization (MR)

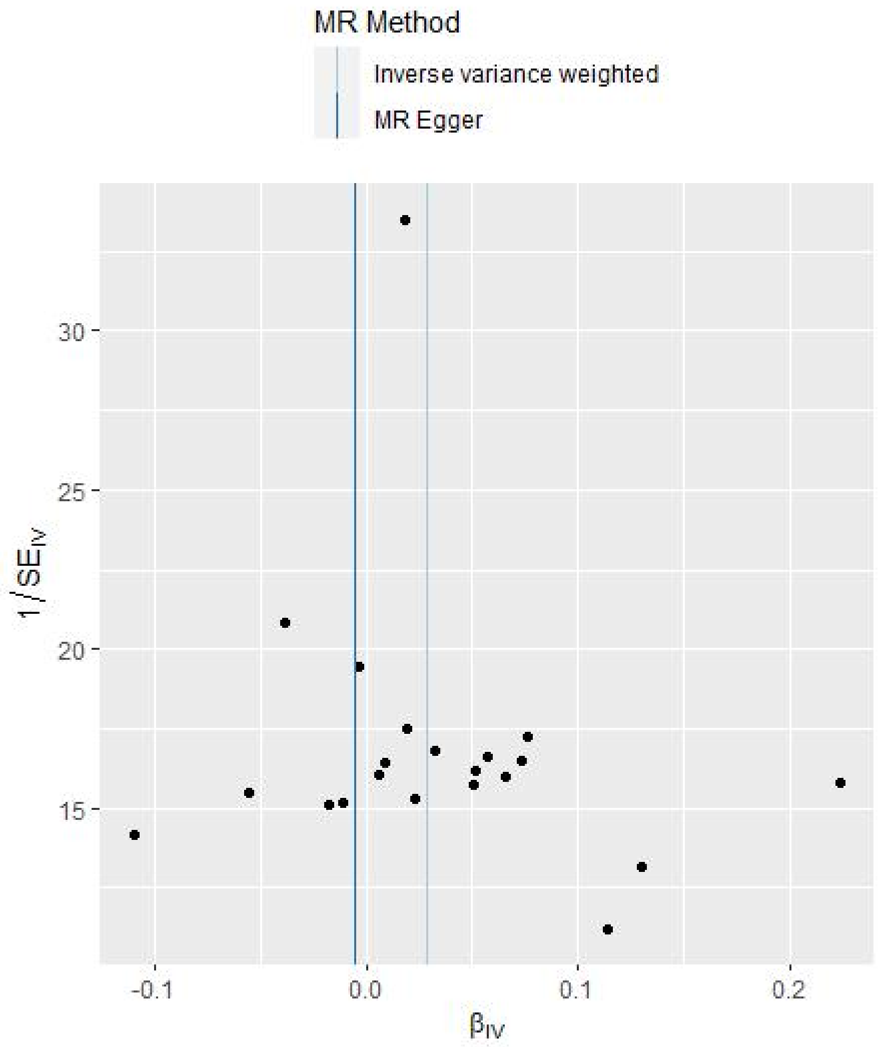

Figure 241: Leave-one-out plot to visualize causal effect of betaine on the risk of myocarditis when leaving one SNP out.

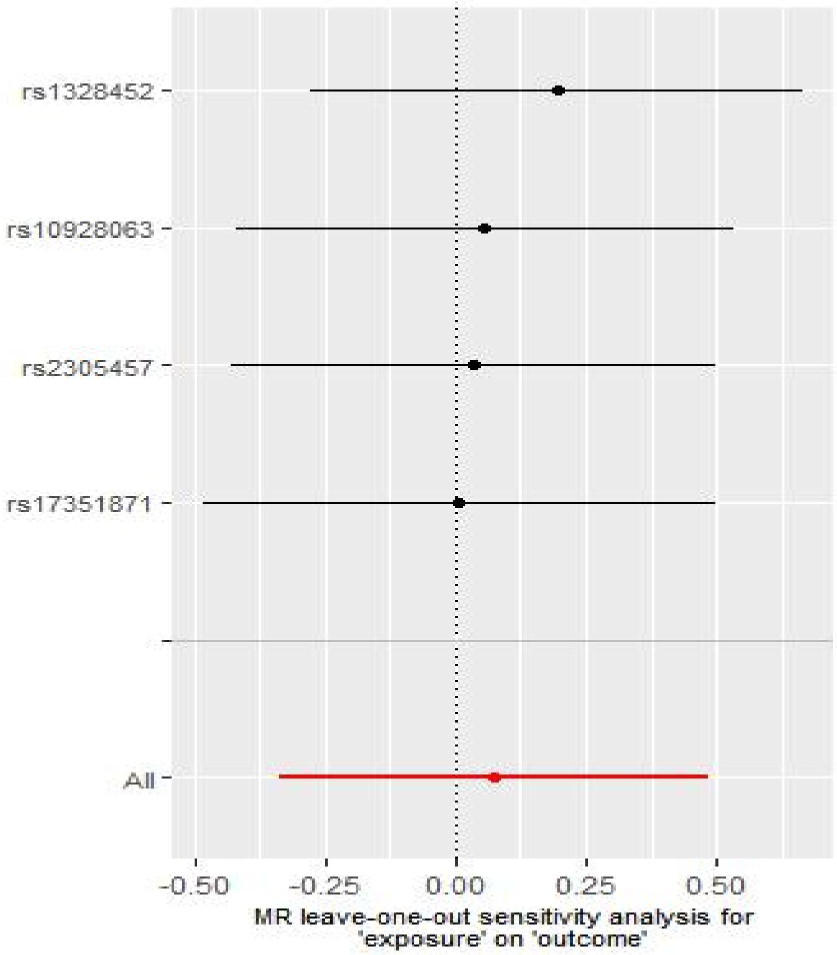

Figure 242: Funnel plots to visualize overall heterogeneity of Mendelian randomization (MR)

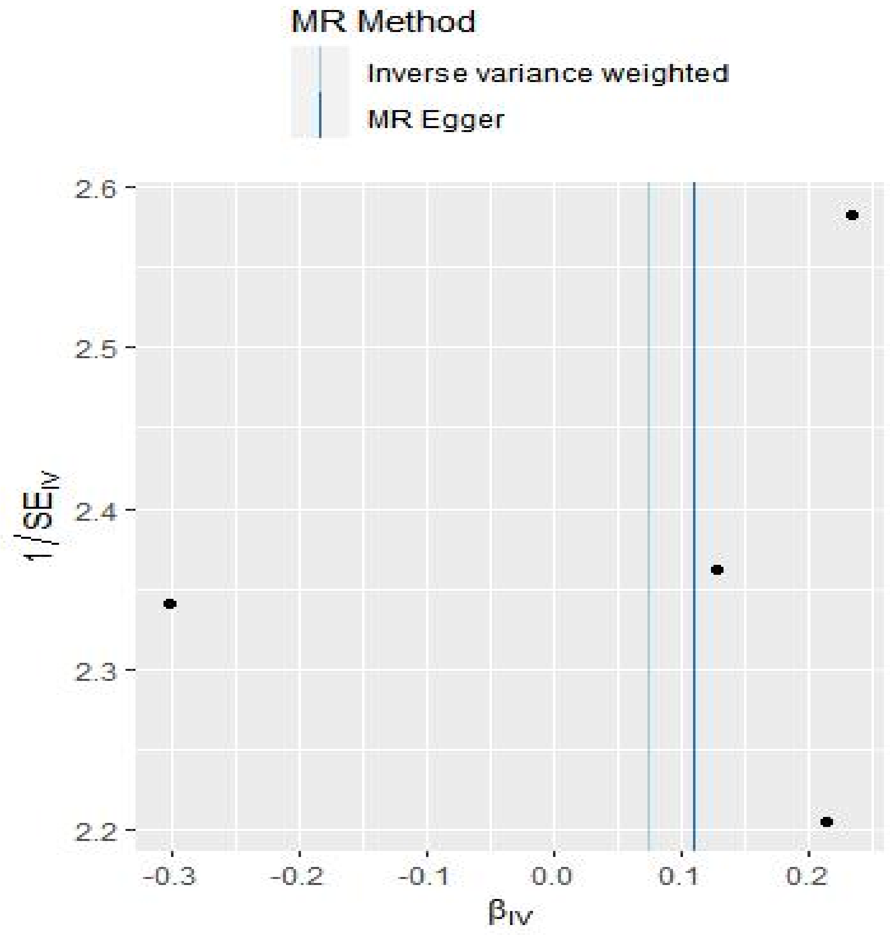

Figure 243: Leave-one-out plot to visualize causal effect of carnitine on the risk of myocarditis when leaving one SNP out.

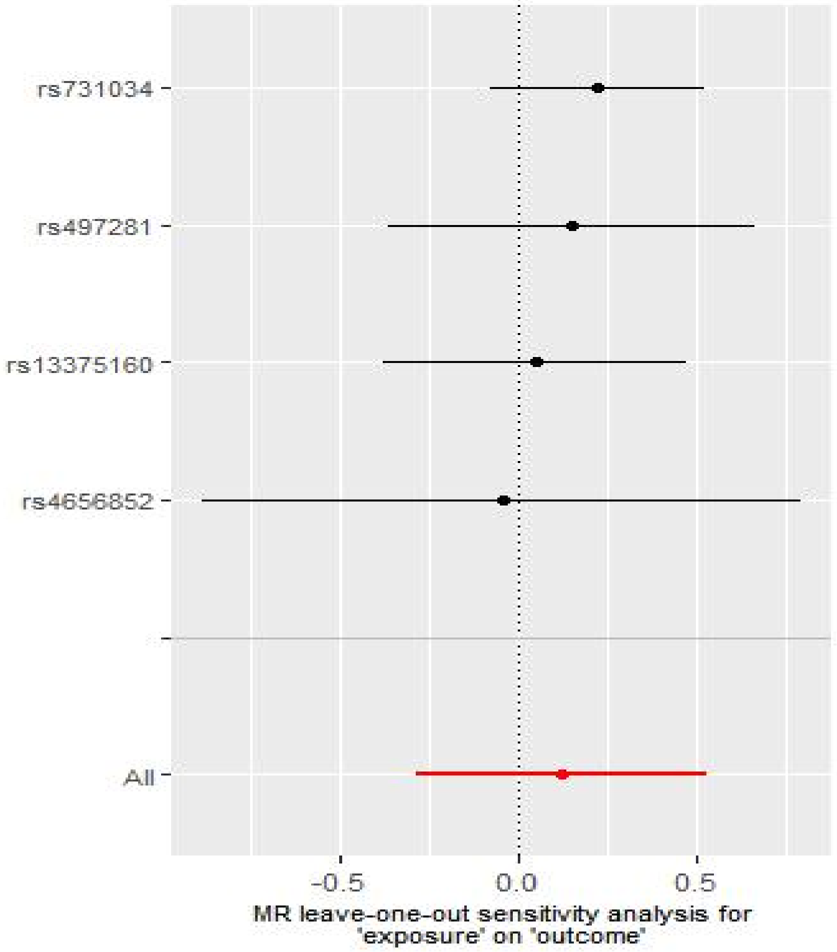

Figure 244: Funnel plots to visualize overall heterogeneity of Mendelian randomization (MR)

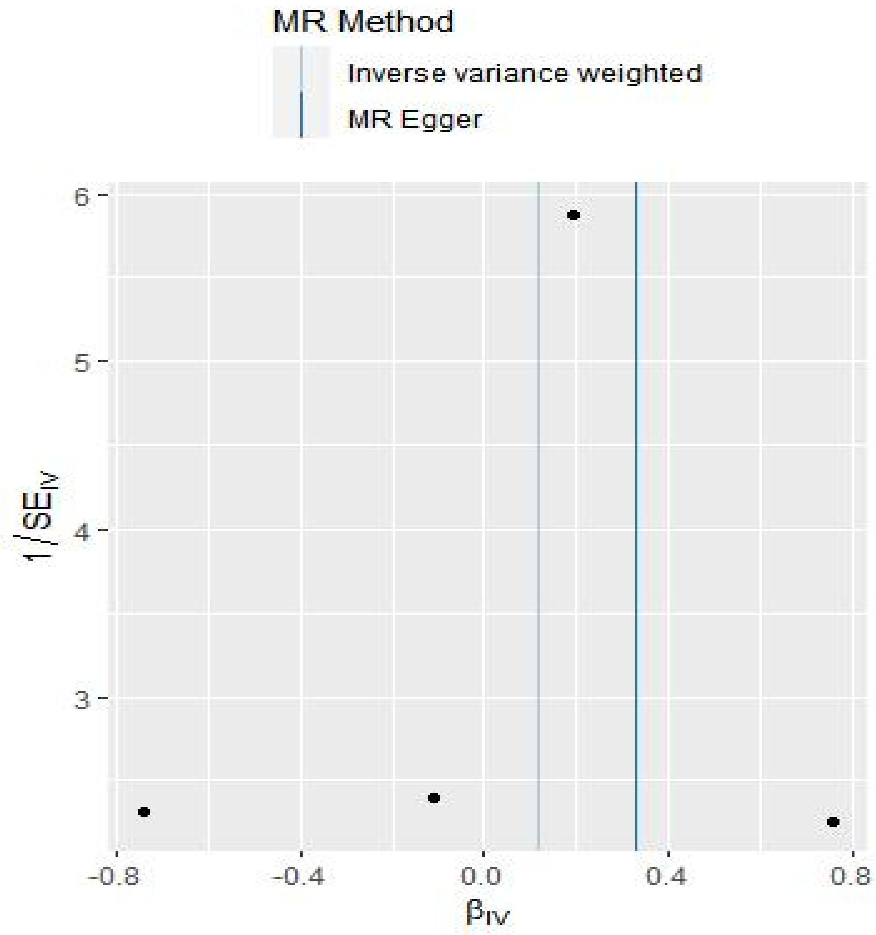

Figure 245: Leave-one-out plot to visualize causal effect of choline on the risk of myocarditis when leaving one SNP out.

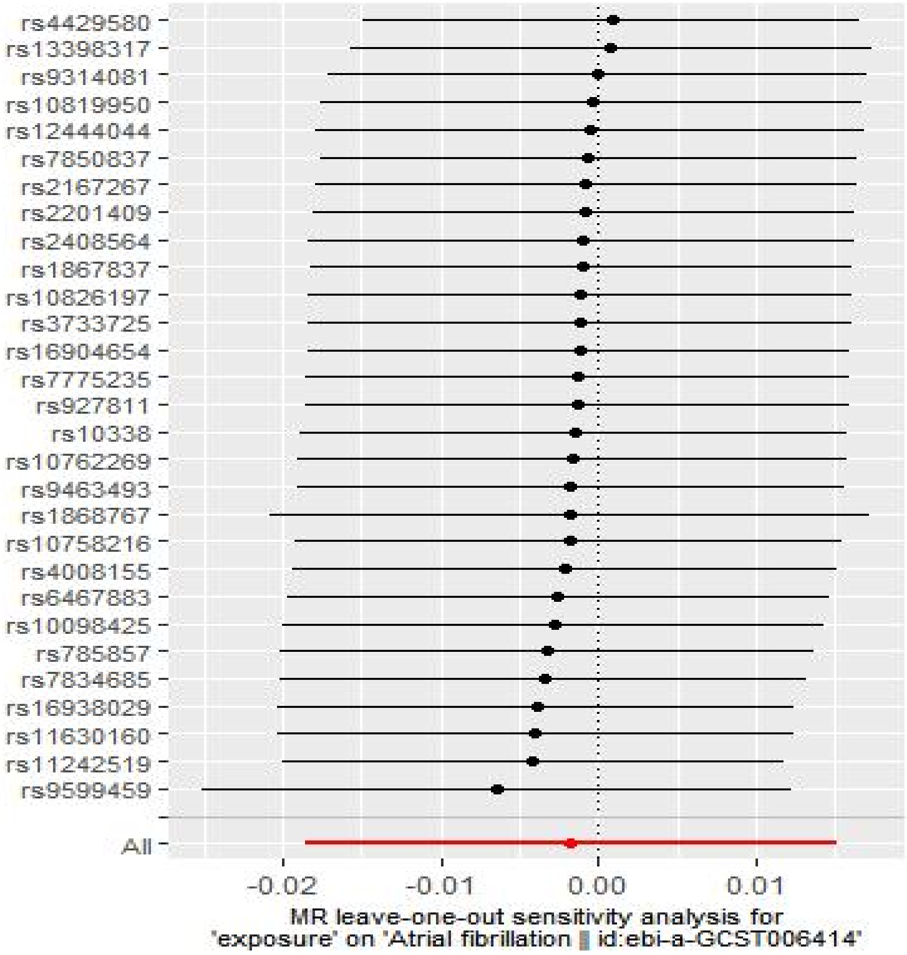

Figure 246: Funnel plots to visualize overall heterogeneity of Mendelian randomization (MR)

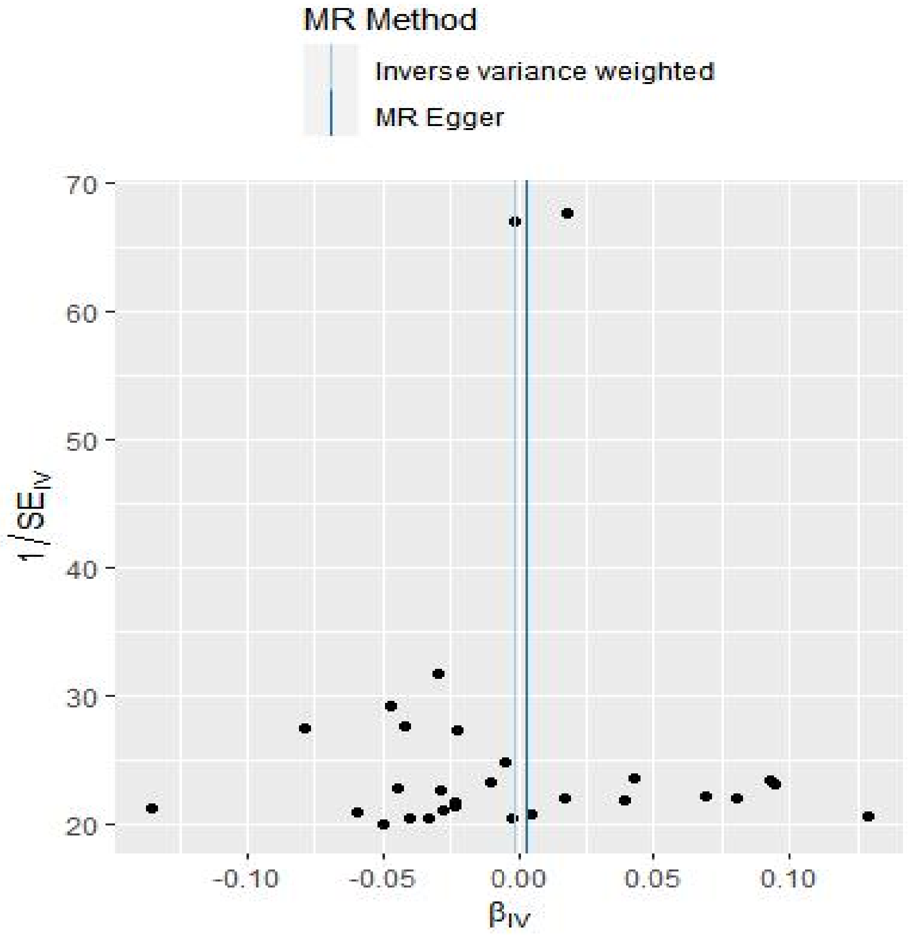

Figure 247: Leave-one-out plot to visualize causal effect of phenylalanine on the risk of myocarditis when leaving one SNP out.

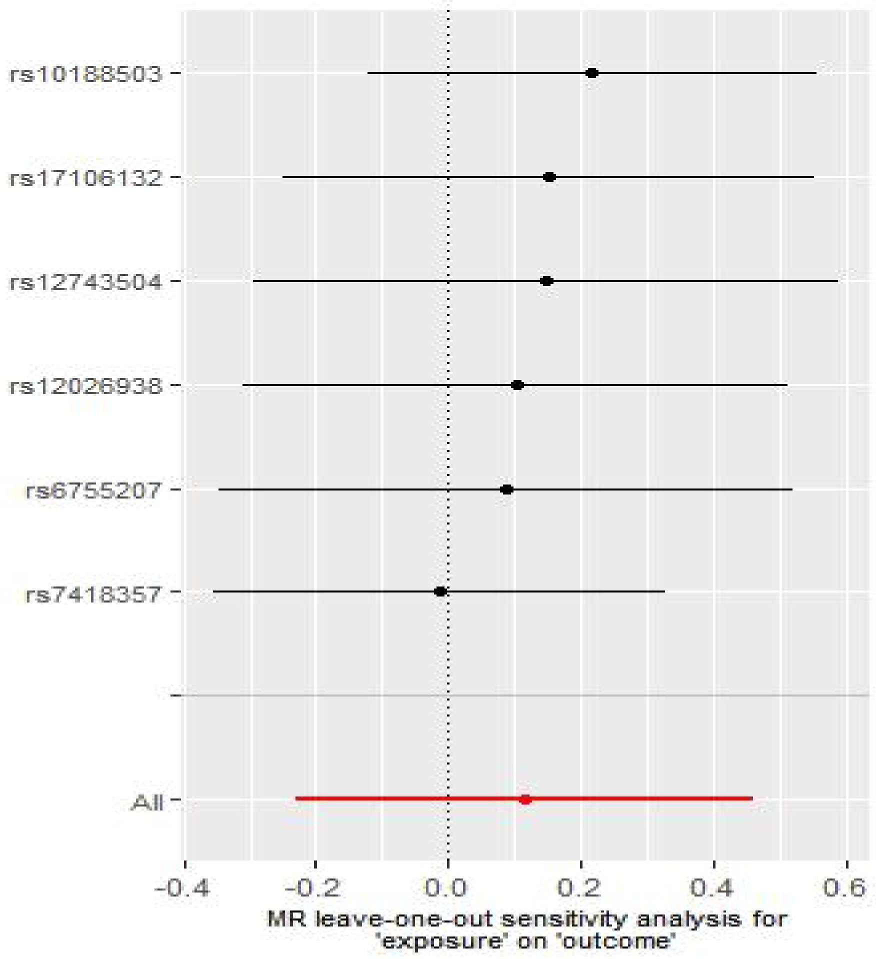

Figure 248: Funnel plots to visualize overall heterogeneity of Mendelian randomization (MR)

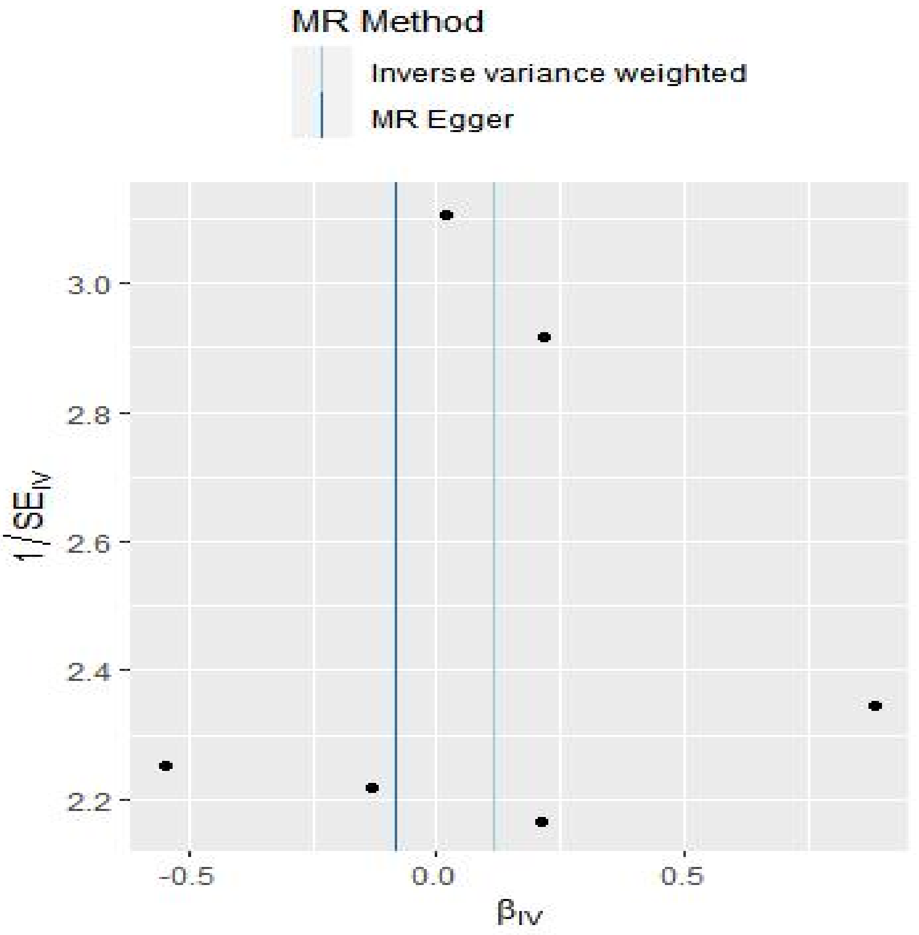

Figure 249: Leave-one-out plot to visualize causal effect of serotonin on the risk of myocarditis when leaving one SNP out.

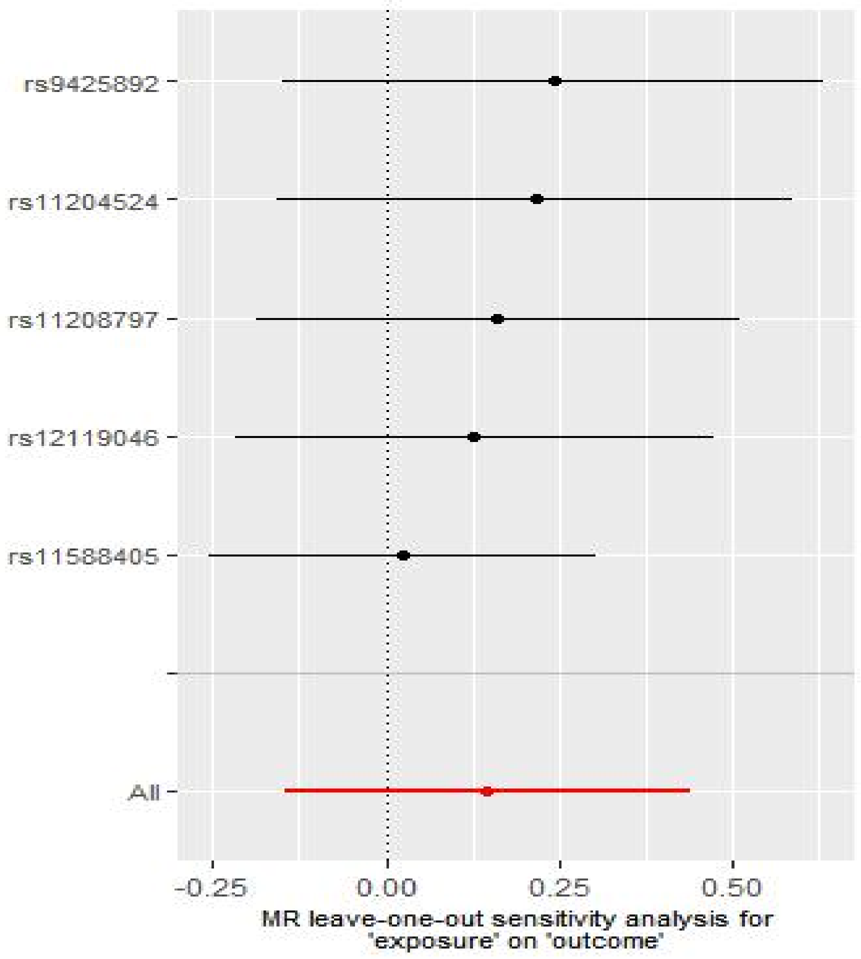

Figure 250: Funnel plots to visualize overall heterogeneity of Mendelian randomization (MR)

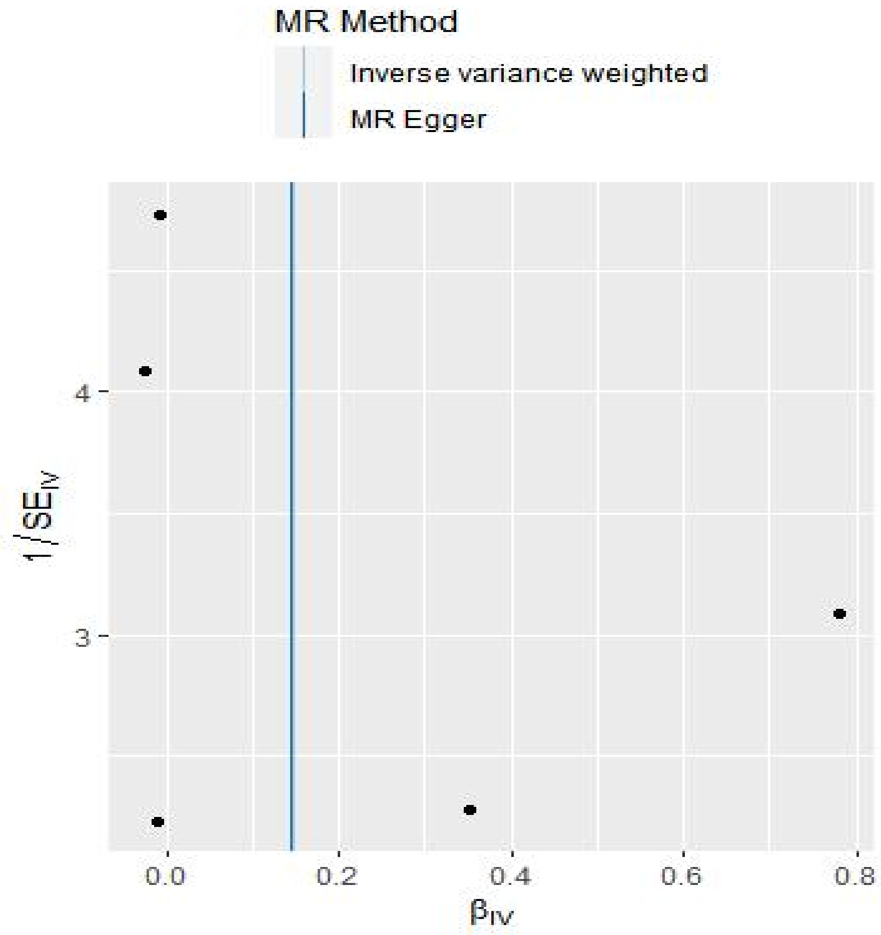

Figure 251: Leave-one-out plot to visualize causal effect of trimethylamine_N_oxide on the risk of myocarditis when leaving one SNP out.

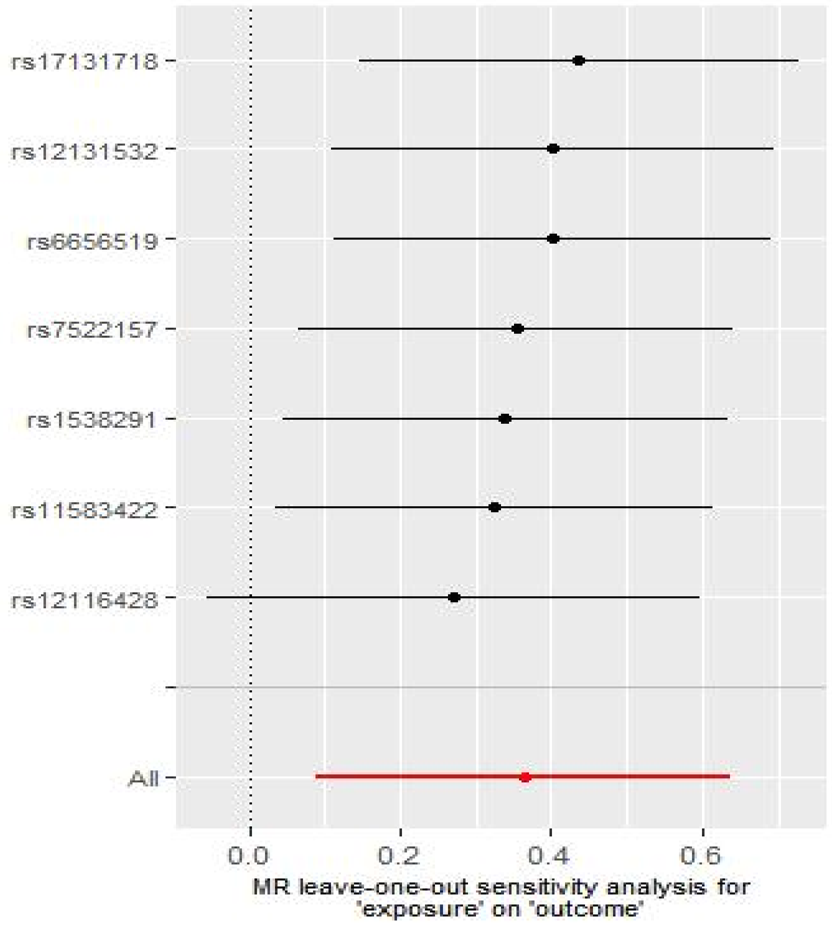

Figure 252: Funnel plots to visualize overall heterogeneity of Mendelian randomization (MR)

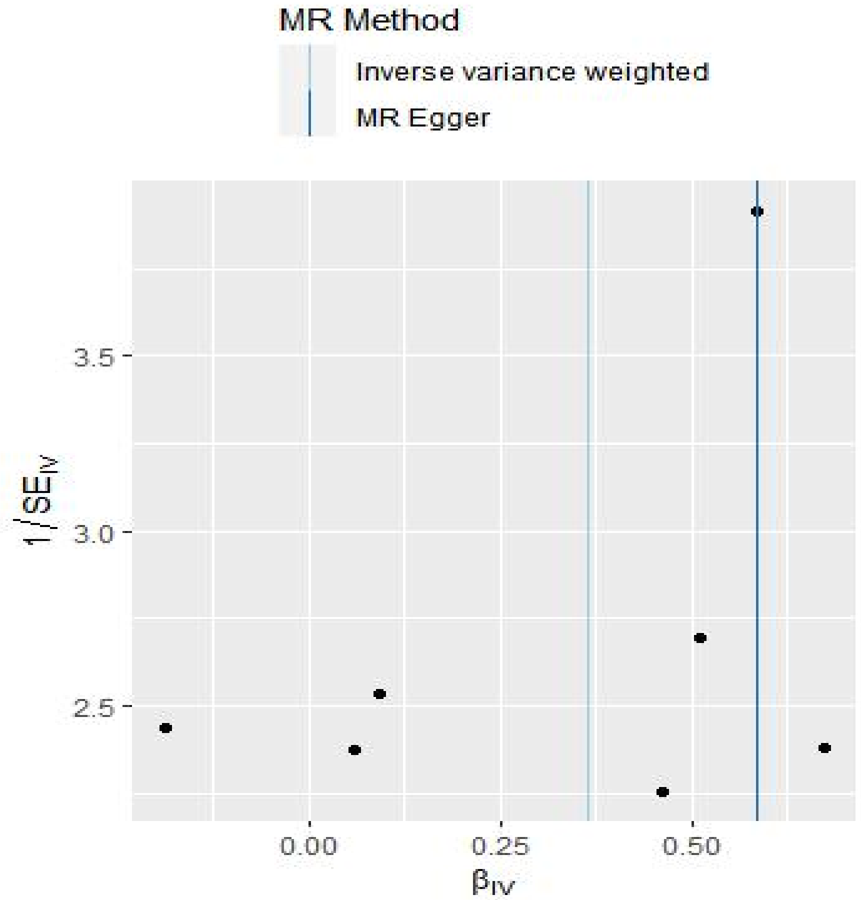

Figure 253: Leave-one-out plot to visualize causal effect of tryptophan on the risk of myocarditis when leaving one SNP out.

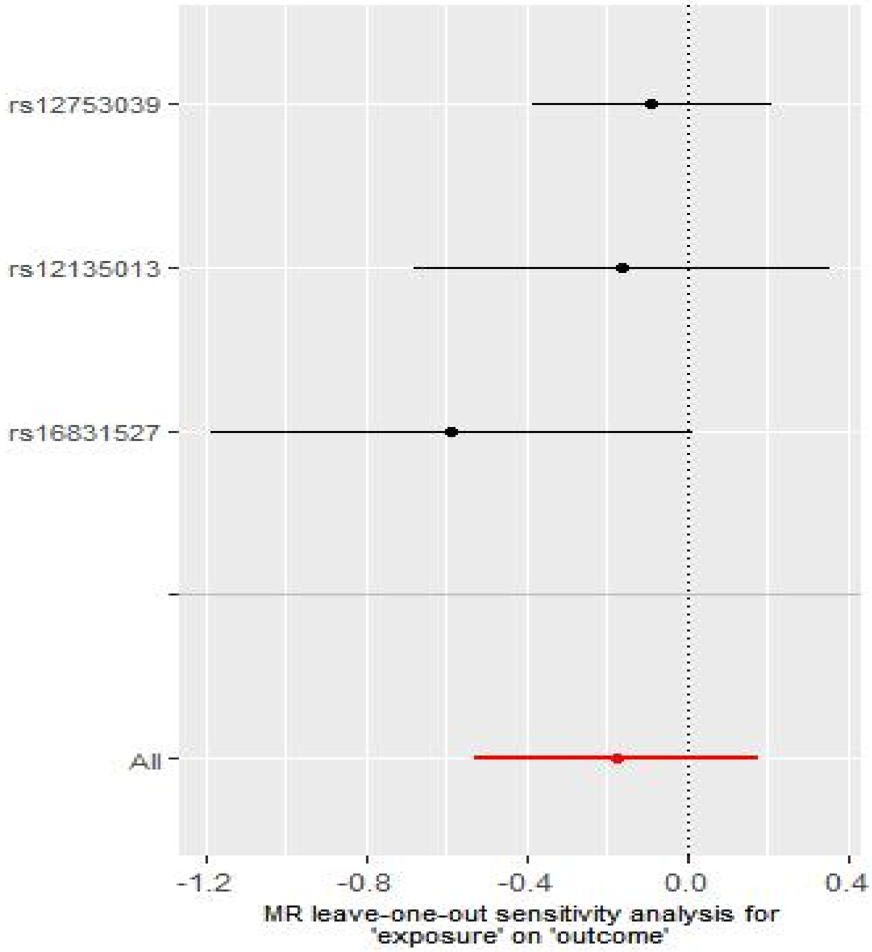

Figure 254: Funnel plots to visualize overall heterogeneity of Mendelian randomization (MR)

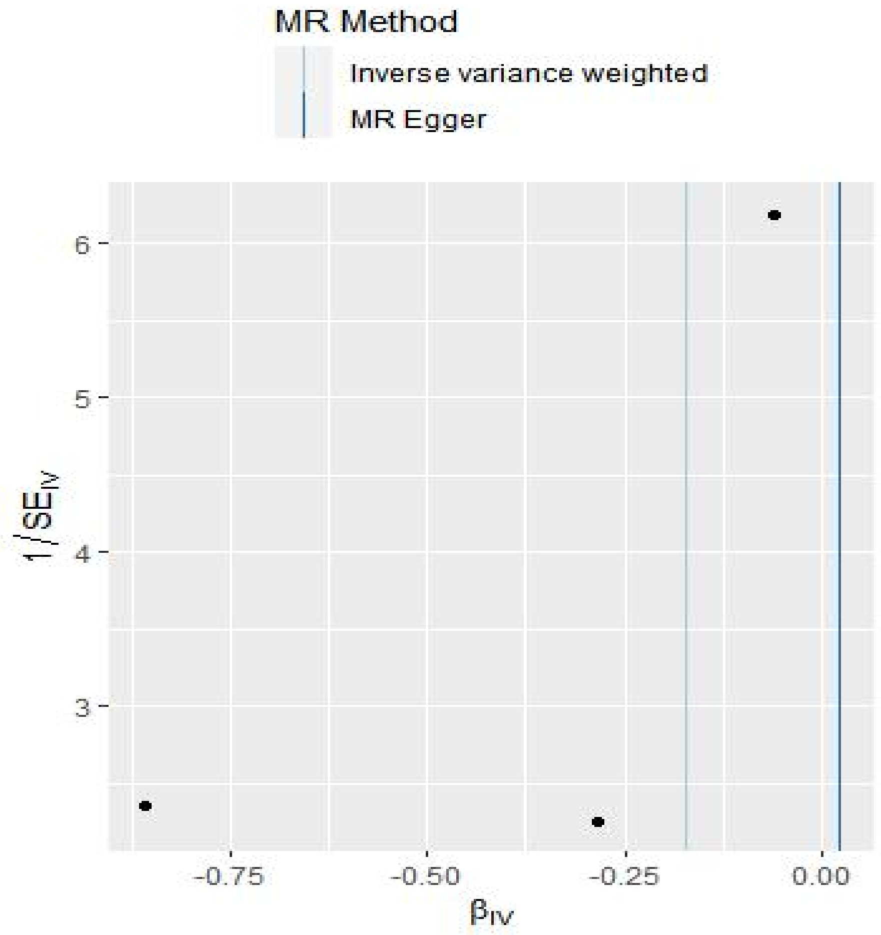

Figure 255: Leave-one-out plot to visualize causal effect of beta_hydroxybutyric acid on the risk of heart valve disease when leaving one SNP out.

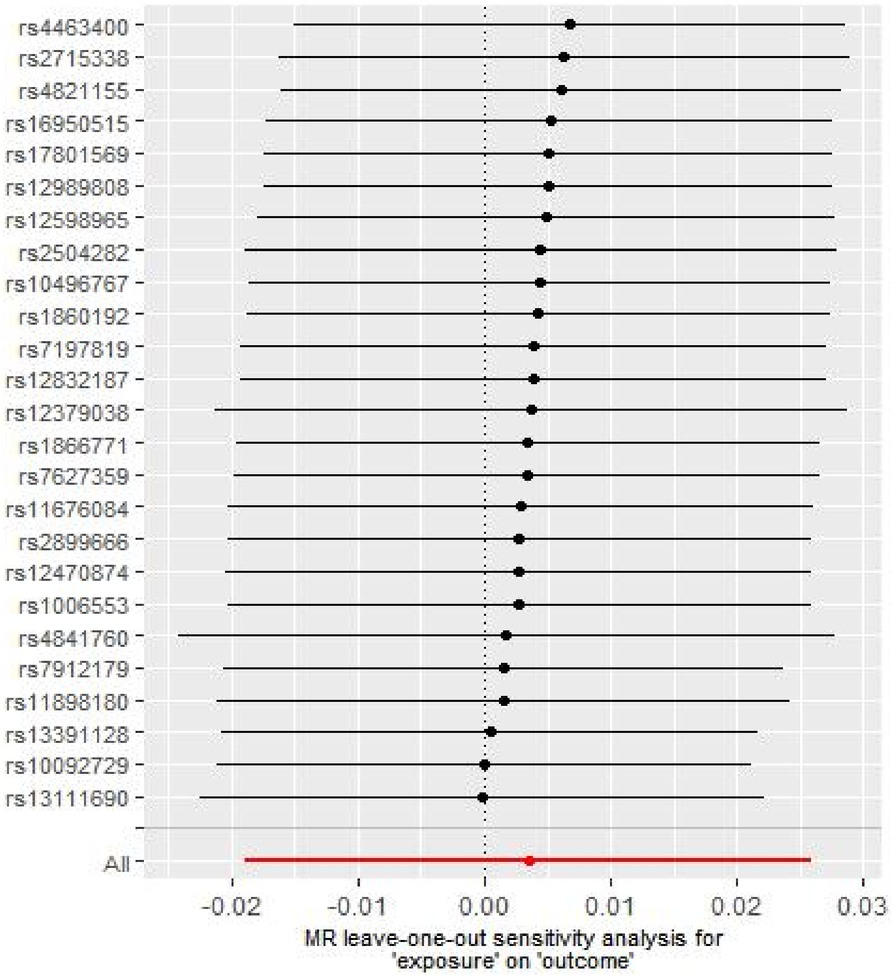

Figure 256: Funnel plots to visualize overall heterogeneity of Mendelian randomization (MR)

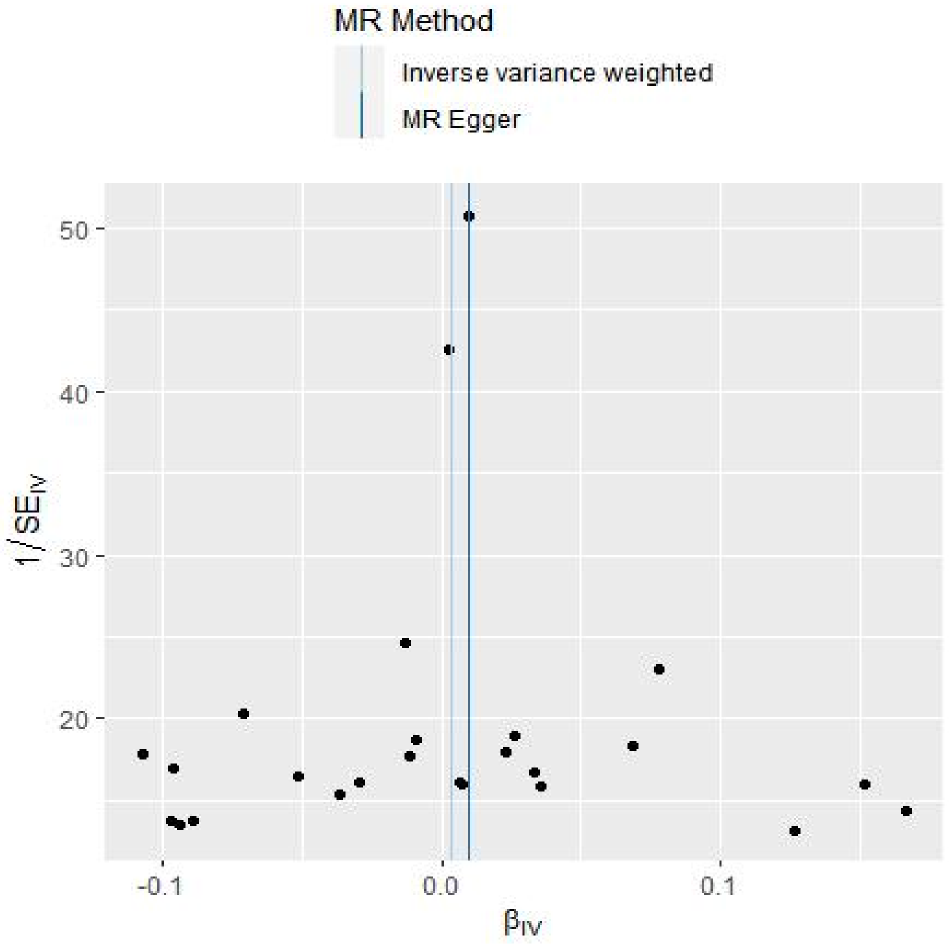

Figure 257: Leave-one-out plot to visualize causal effect of betaine on the risk of heart valve disease when leaving one SNP out.

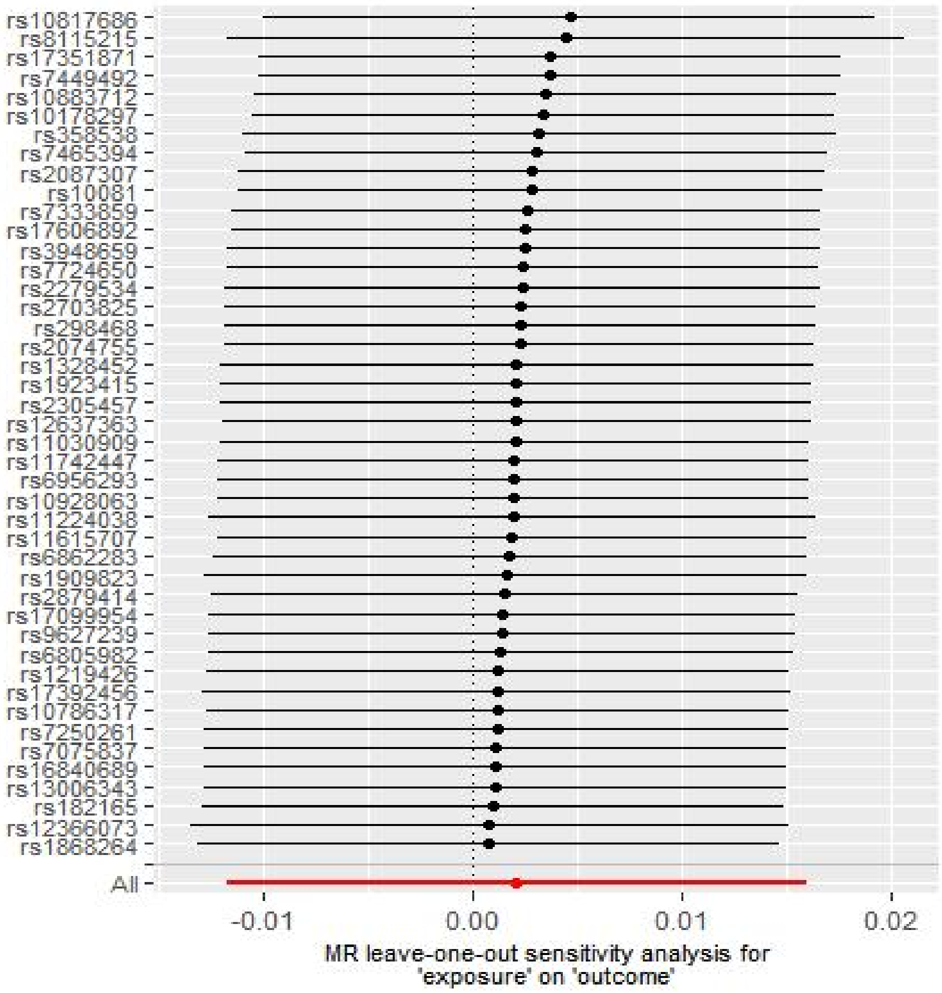

Figure 258: Funnel plots to visualize overall heterogeneity of Mendelian randomization (MR)

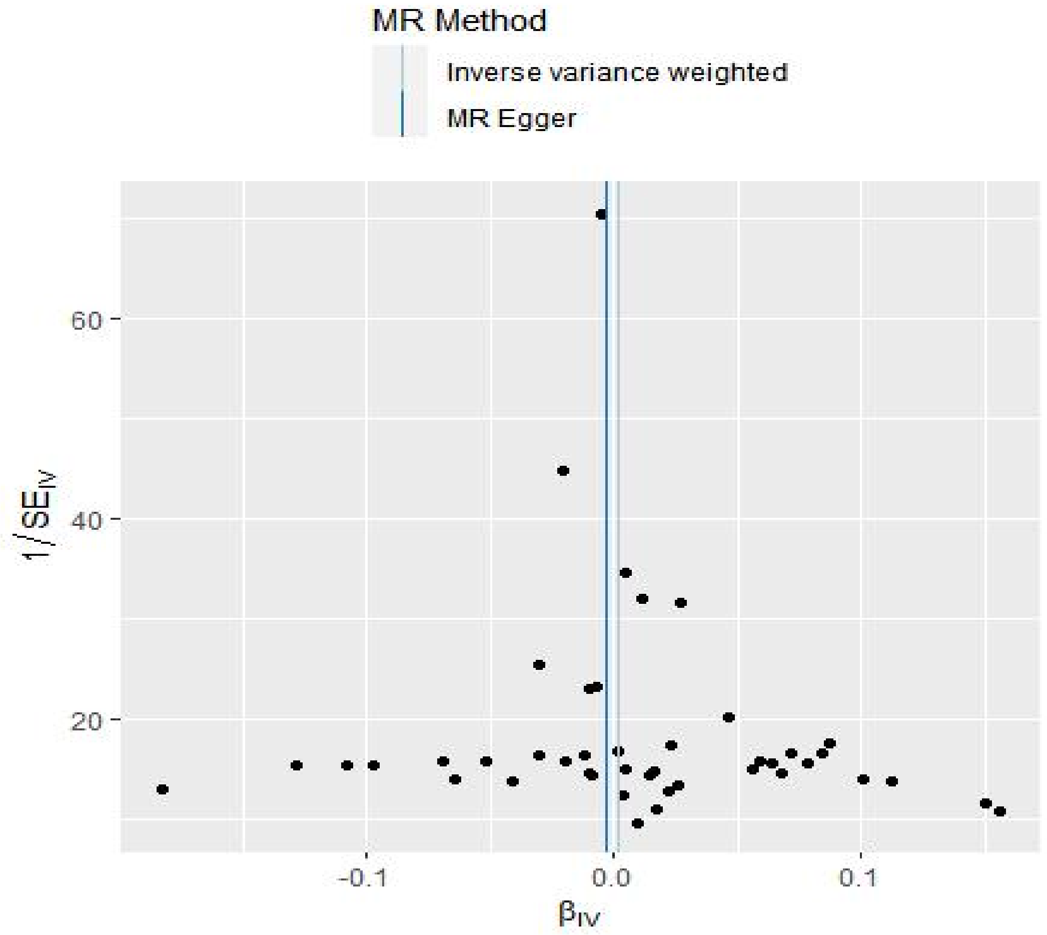

Figure 259: Leave-one-out plot to visualize causal effect of carnitine on the risk of heart valve disease when leaving one SNP out.

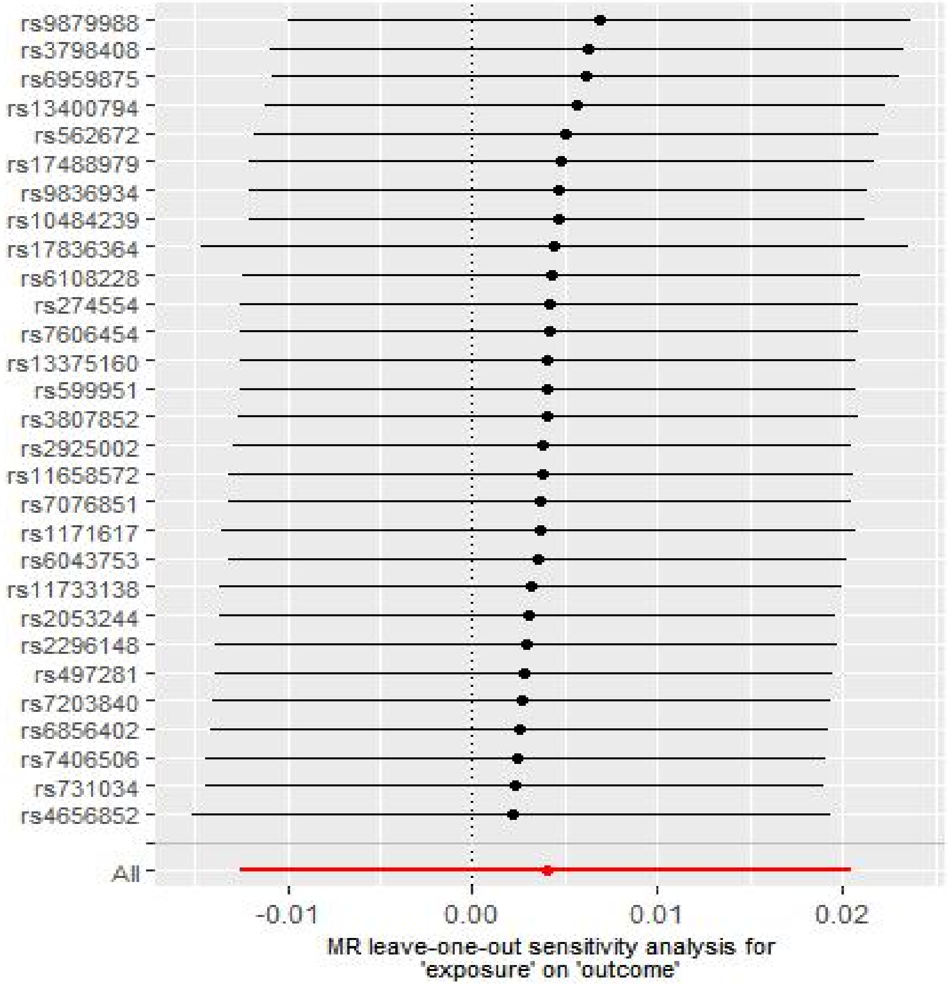

Figure 260: Funnel plots to visualize overall heterogeneity of Mendelian randomization (MR)

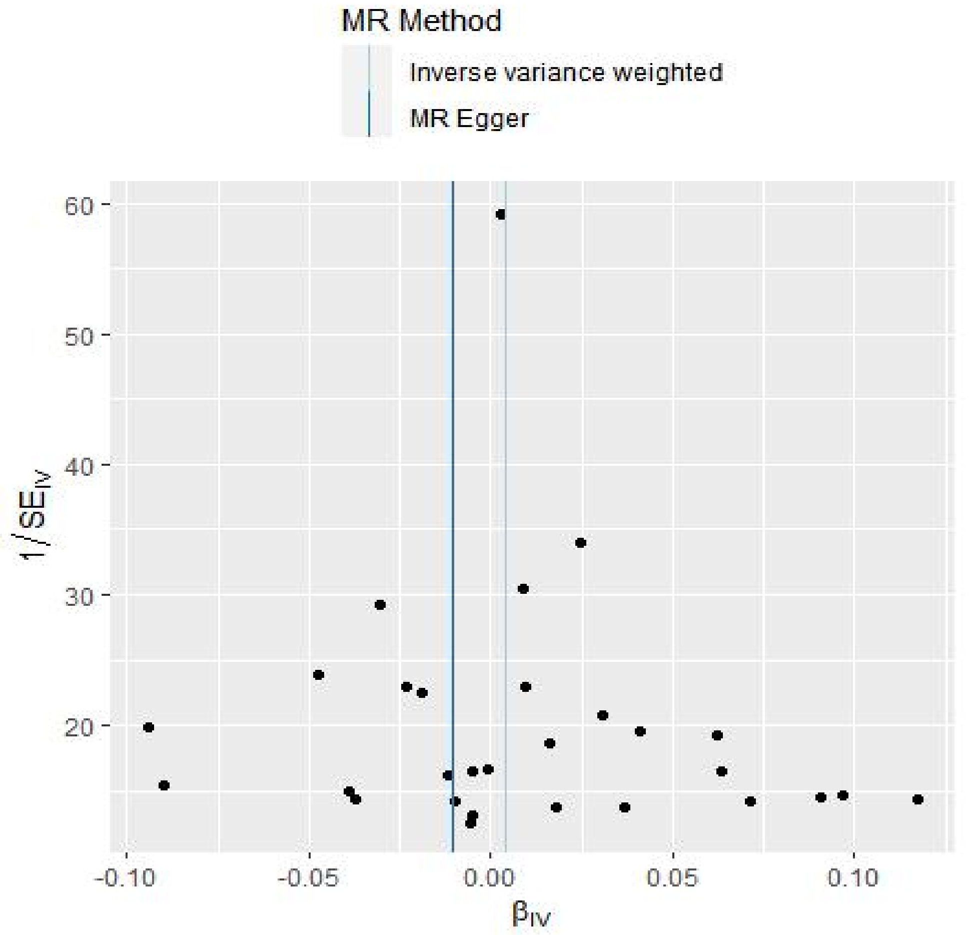

Figure 261: Leave-one-out plot to visualize causal effect of choline on the risk of heart valve disease when leaving one SNP out.

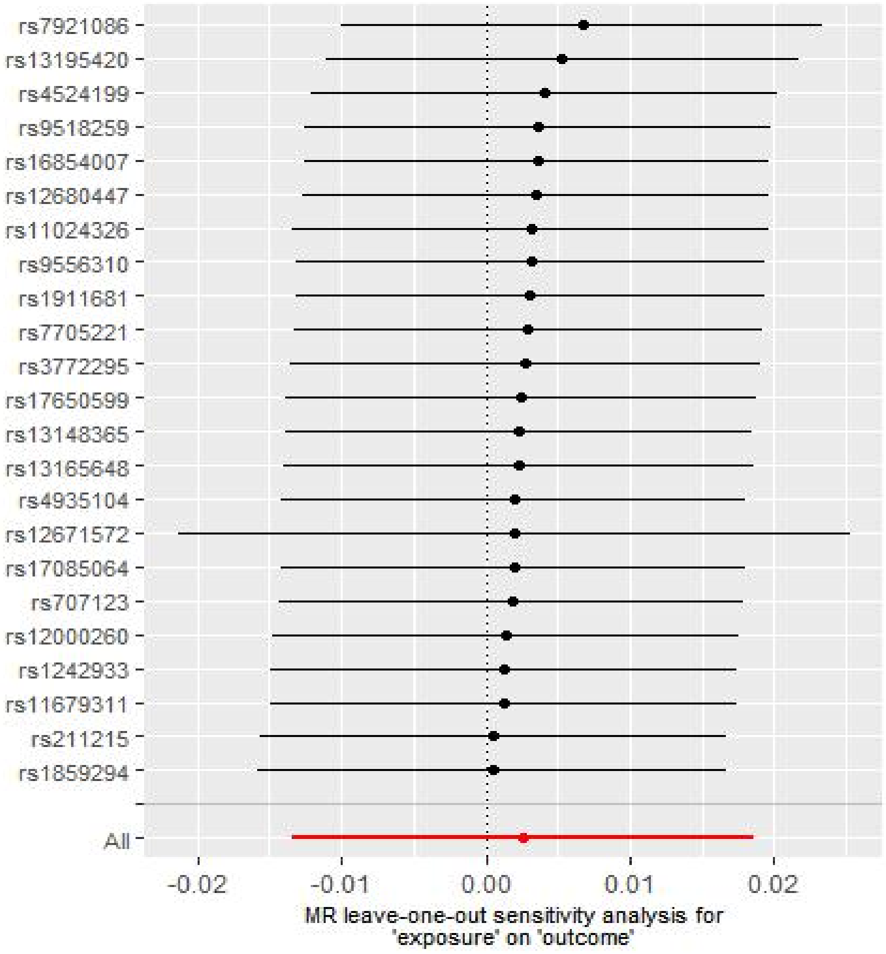

Figure 262: Funnel plots to visualize overall heterogeneity of Mendelian randomization (MR)

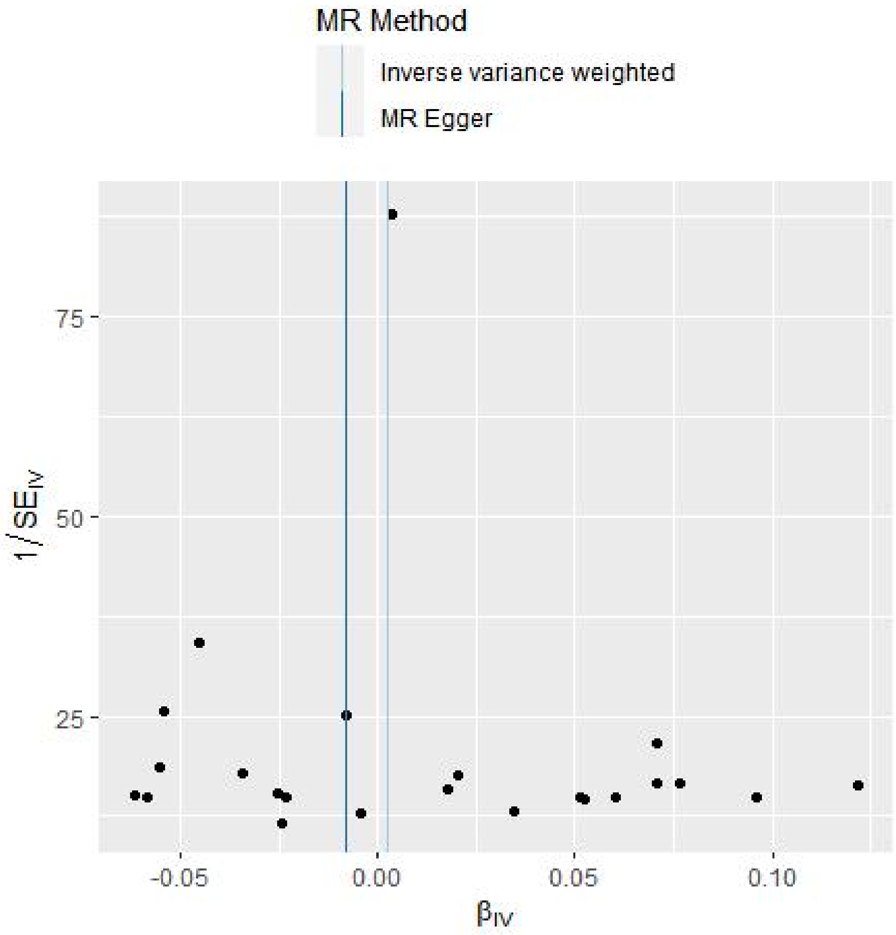

Figure 263: Leave-one-out plot to visualize causal effect of glutamate on the risk of heart valve disease when leaving one SNP out.

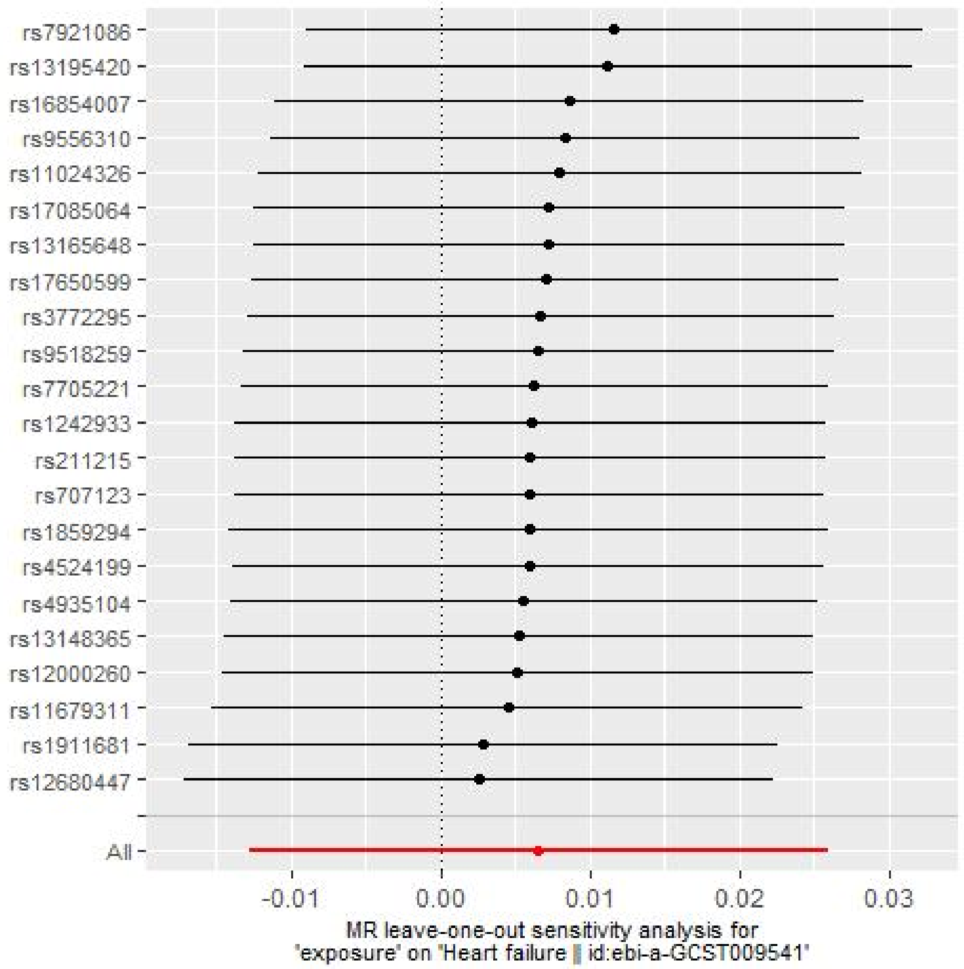

Figure 264: Funnel plots to visualize overall heterogeneity of Mendelian randomization (MR)

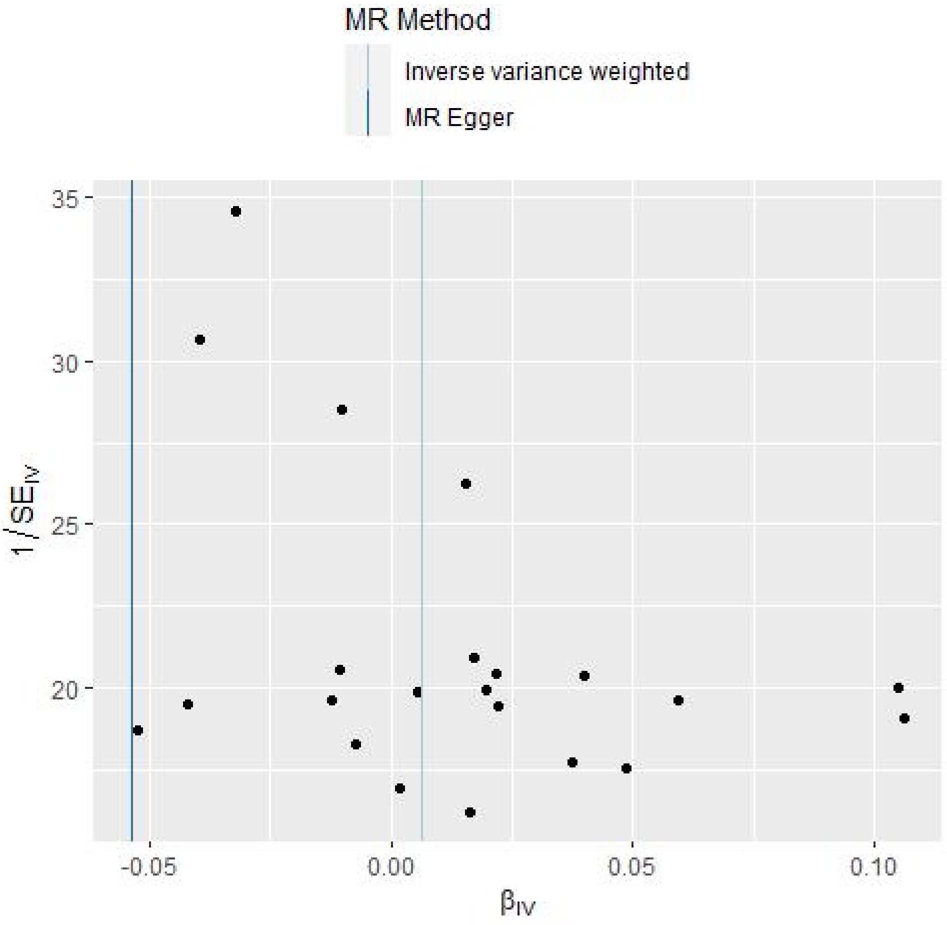

Figure 265: Leave-one-out plot to visualize causal effect of kynuremine on the risk of heart valve disease when leaving one SNP out.

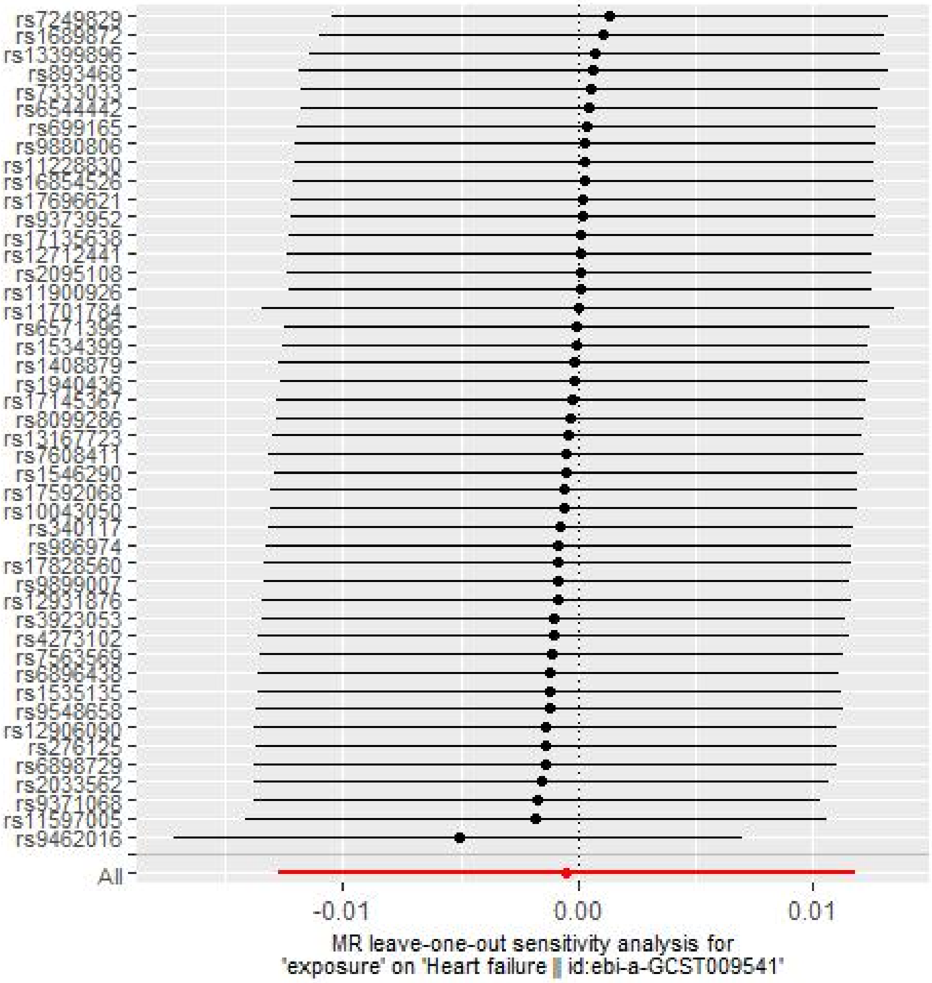

Figure 266: Funnel plots to visualize overall heterogeneity of Mendelian randomization (MR)

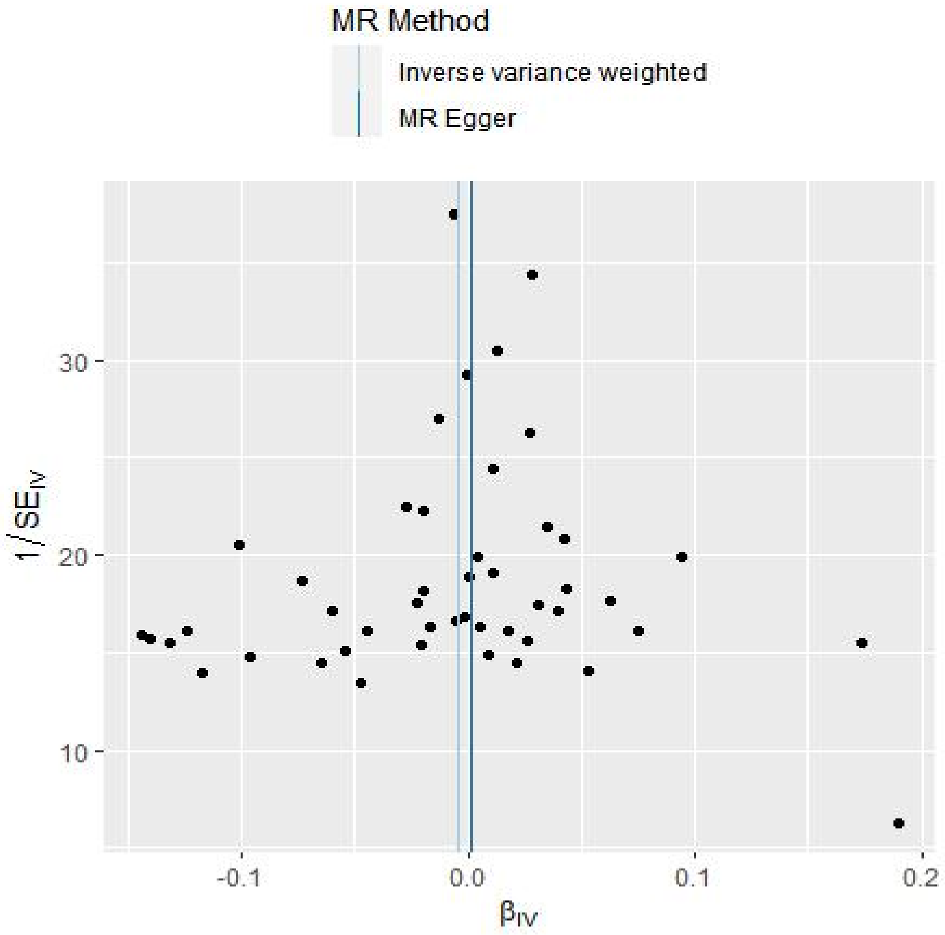

Figure 267: Leave-one-out plot to visualize causal effect of phenylalanine on the risk of heart valve disease when leaving one SNP out.

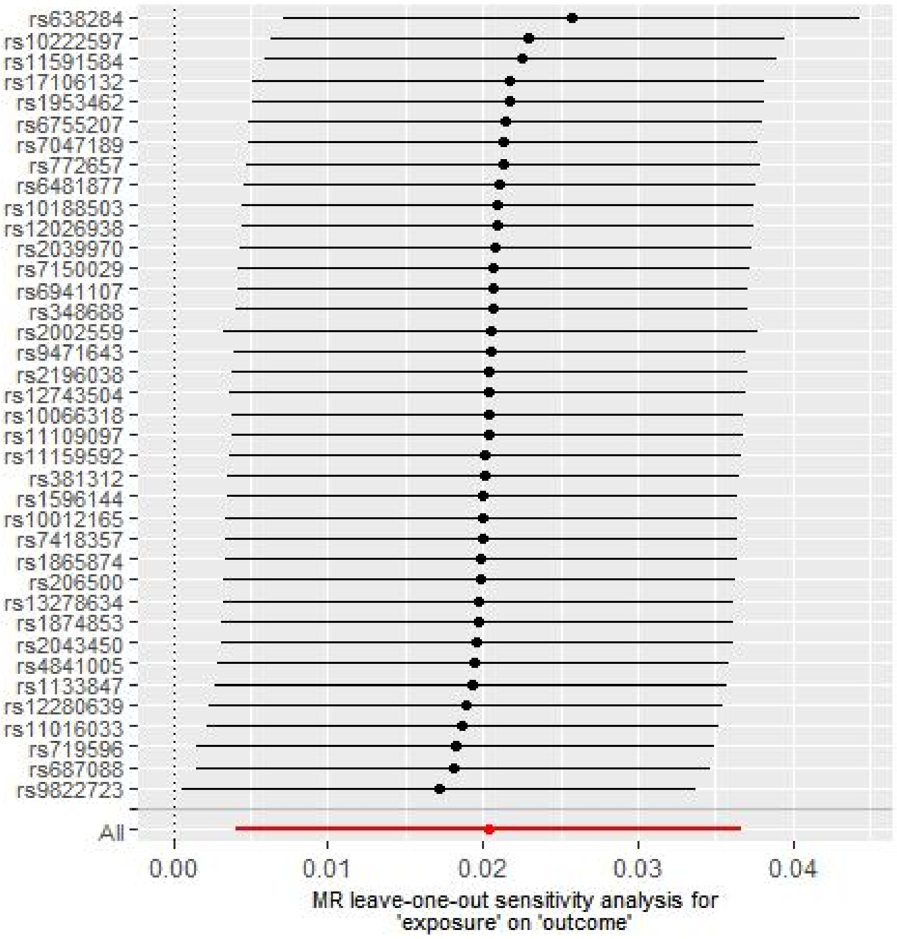

Figure 268: Funnel plots to visualize overall heterogeneity of Mendelian randomization (MR)

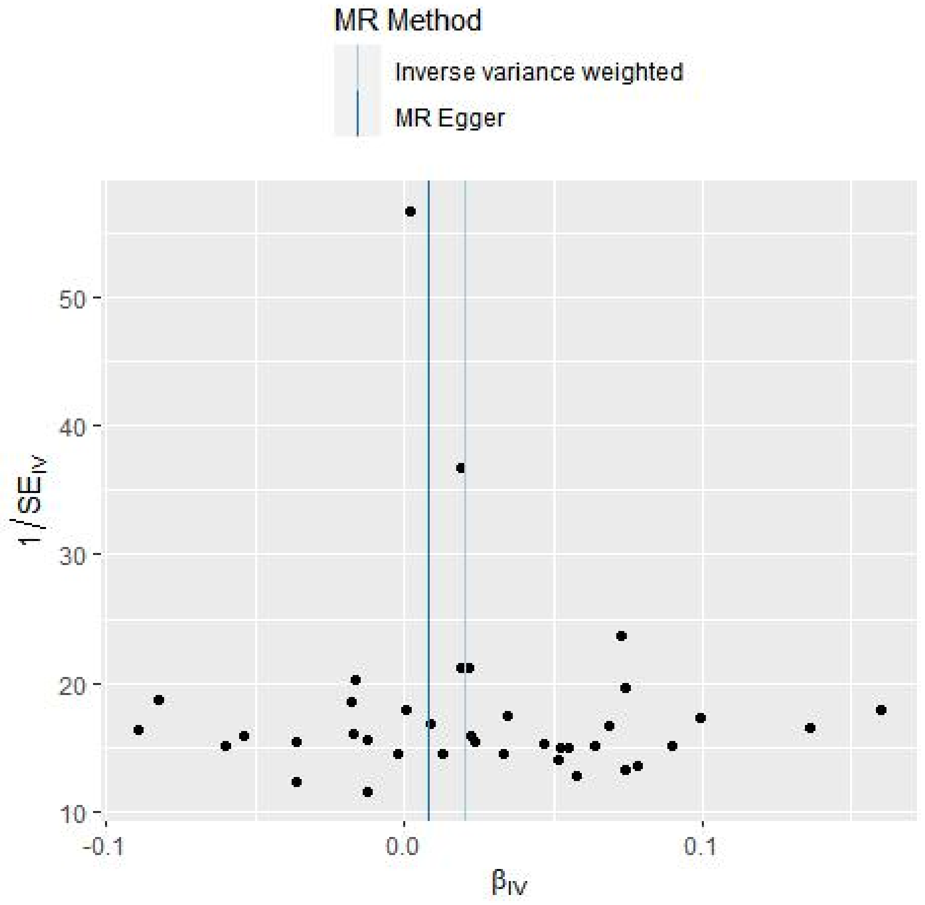

Figure 269: Leave-one-out plot to visualize causal effect of serotonin on the risk of heart valve disease when leaving one SNP out.

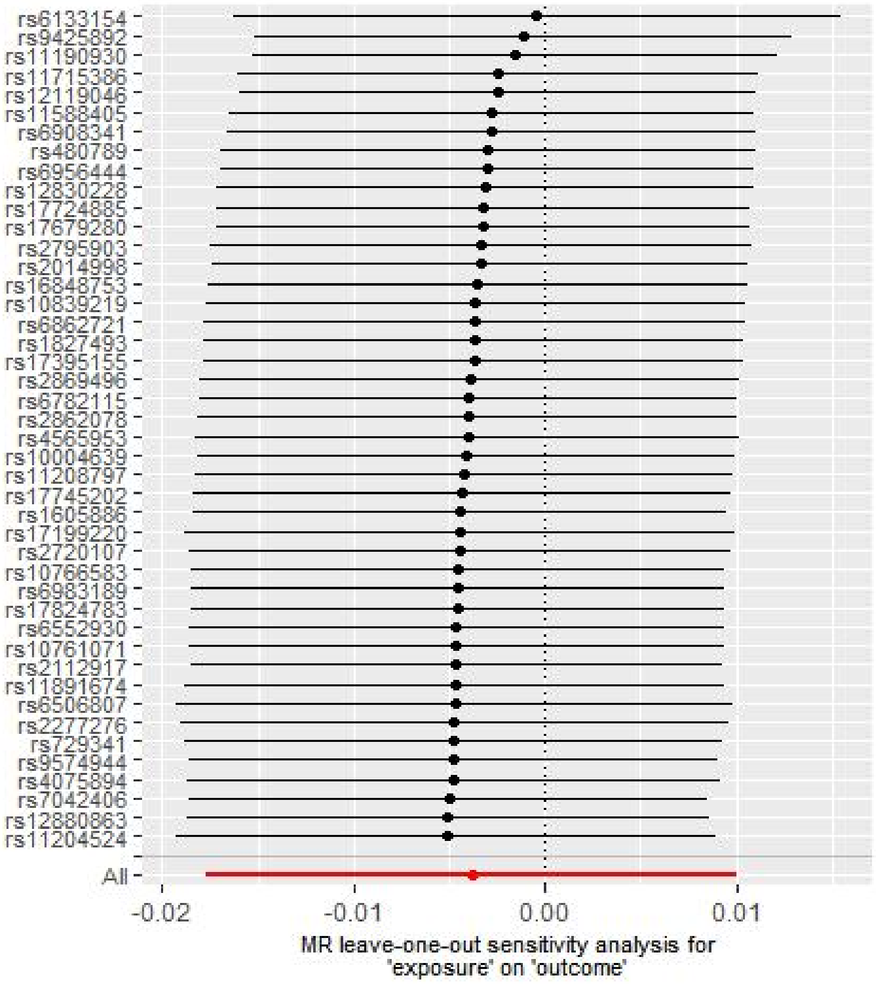

Figure 270: Funnel plots to visualize overall heterogeneity of Mendelian randomization (MR)

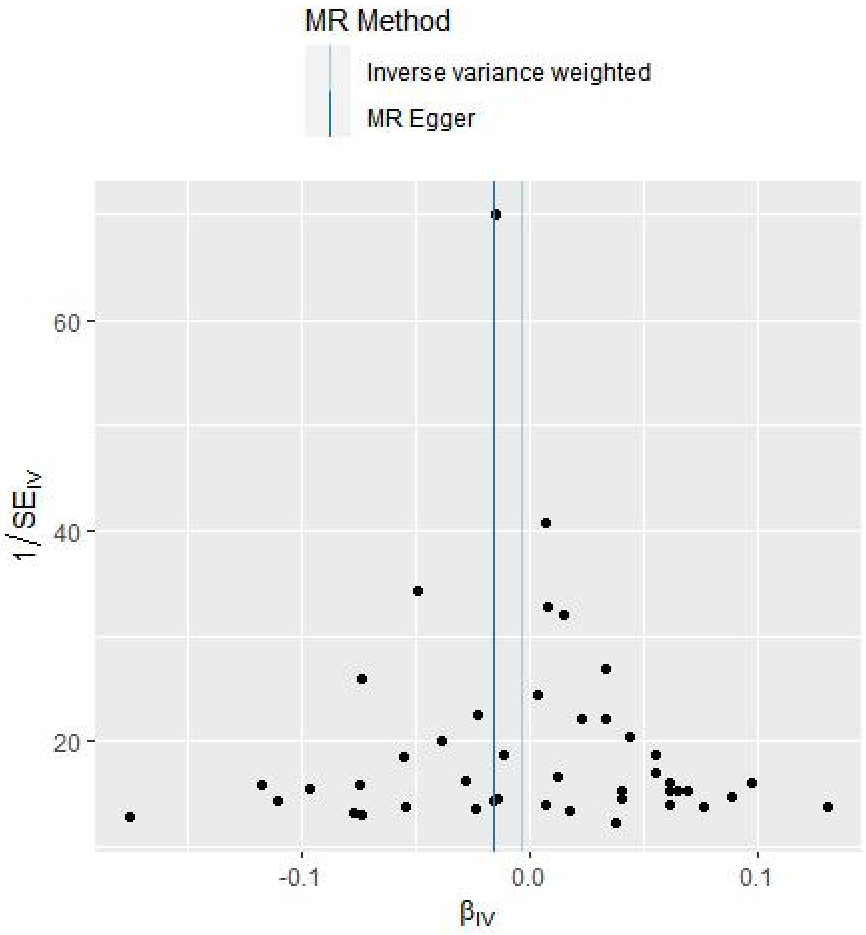

Figure 271: Leave-one-out plot to visualize causal effect of trimethylamine_N_oxide on the risk of heart valve disease when leaving one SNP out.

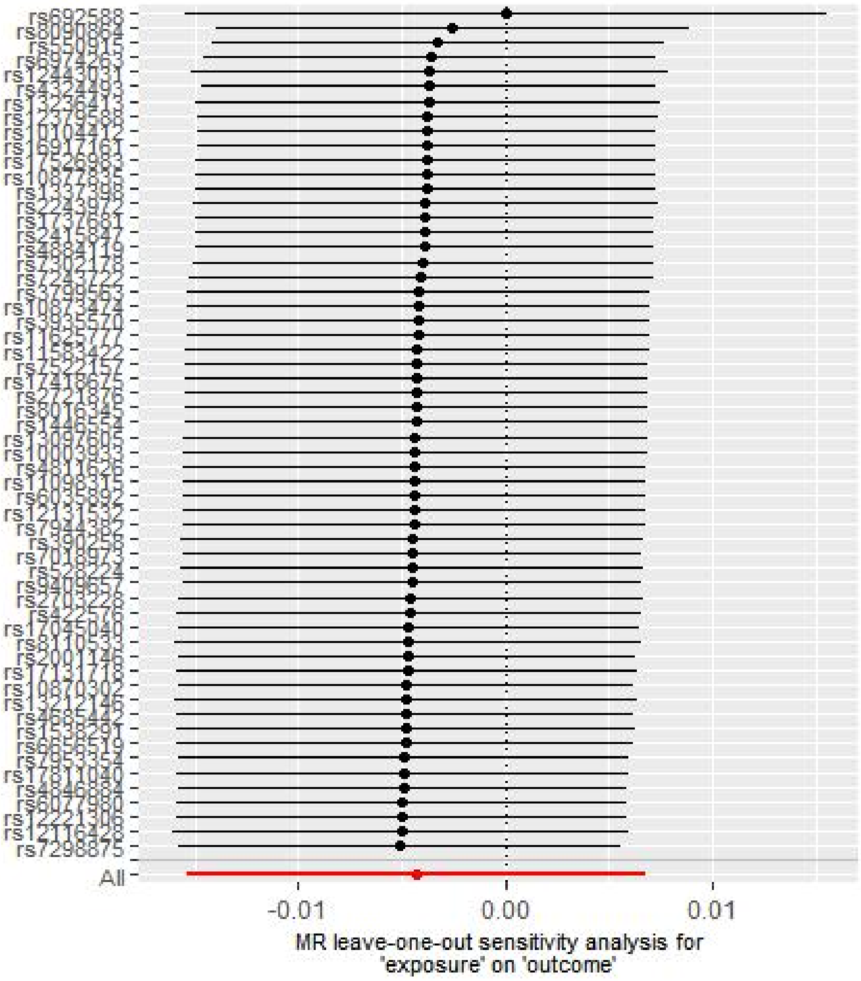

Figure 272: Funnel plots to visualize overall heterogeneity of Mendelian randomization (MR)

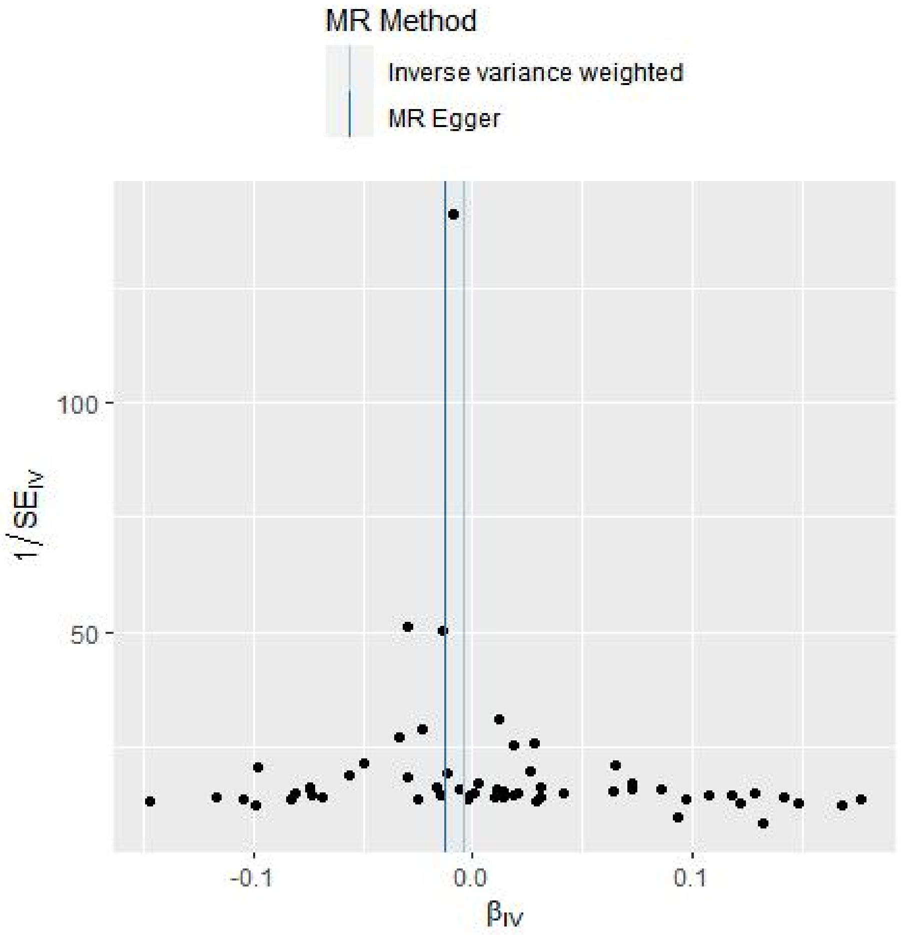

Figure 273: Leave-one-out plot to visualize causal effect of tryptophan on the risk of heart valve disease when leaving one SNP out.

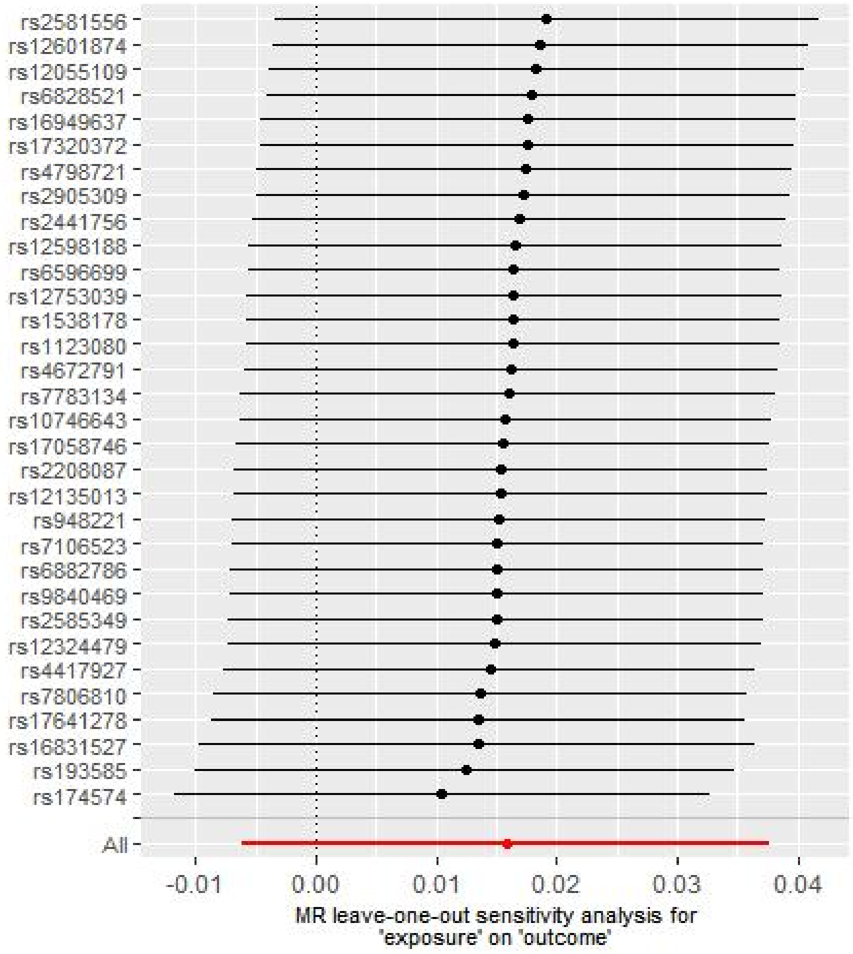

Figure 274: Funnel plots to visualize overall heterogeneity of Mendelian randomization (MR) estimates for the effect of psoriasis on the risk of heart valve disease

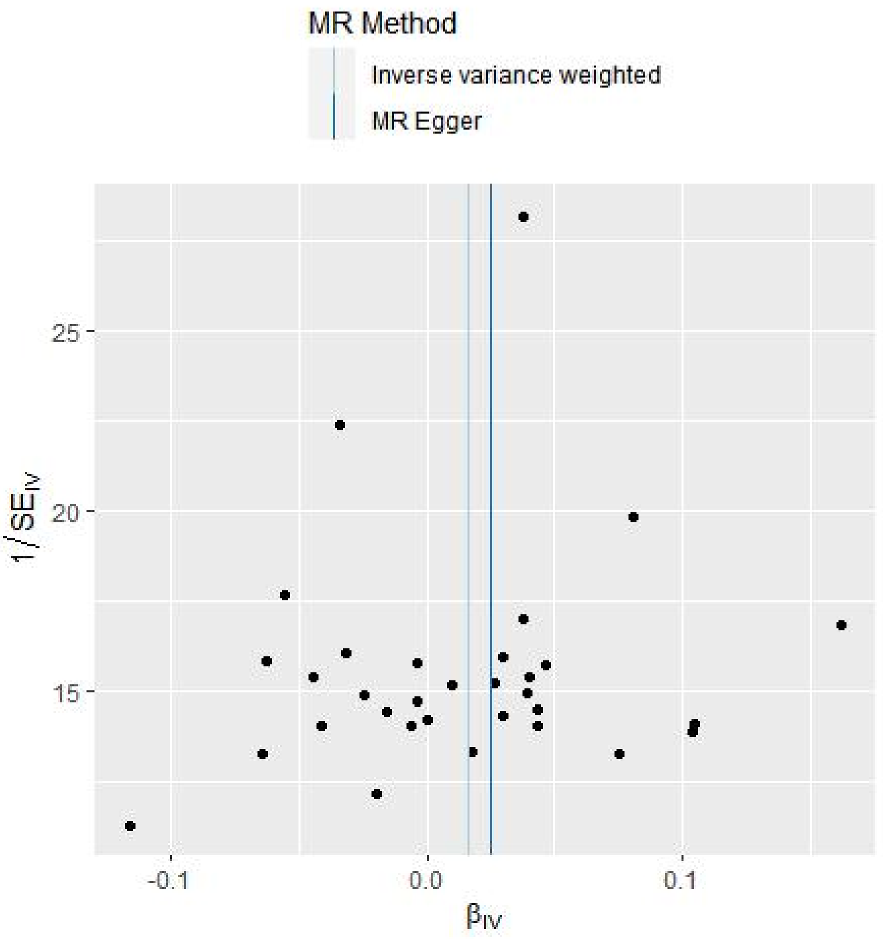

Figure 275: Leave-one-out plot to visualize causal effect of tyrosine on the risk of heart valve disease when leaving one SNP out.

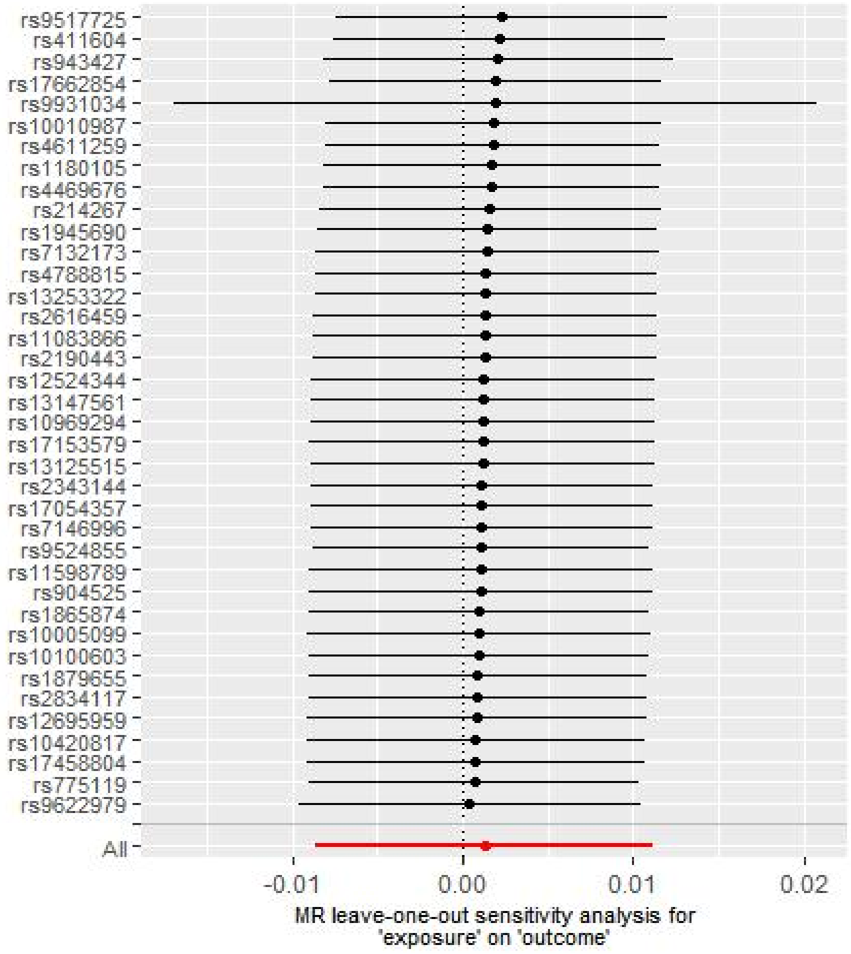

Figure 276: Funnel plots to visualize overall heterogeneity of Mendelian randomization (MR)

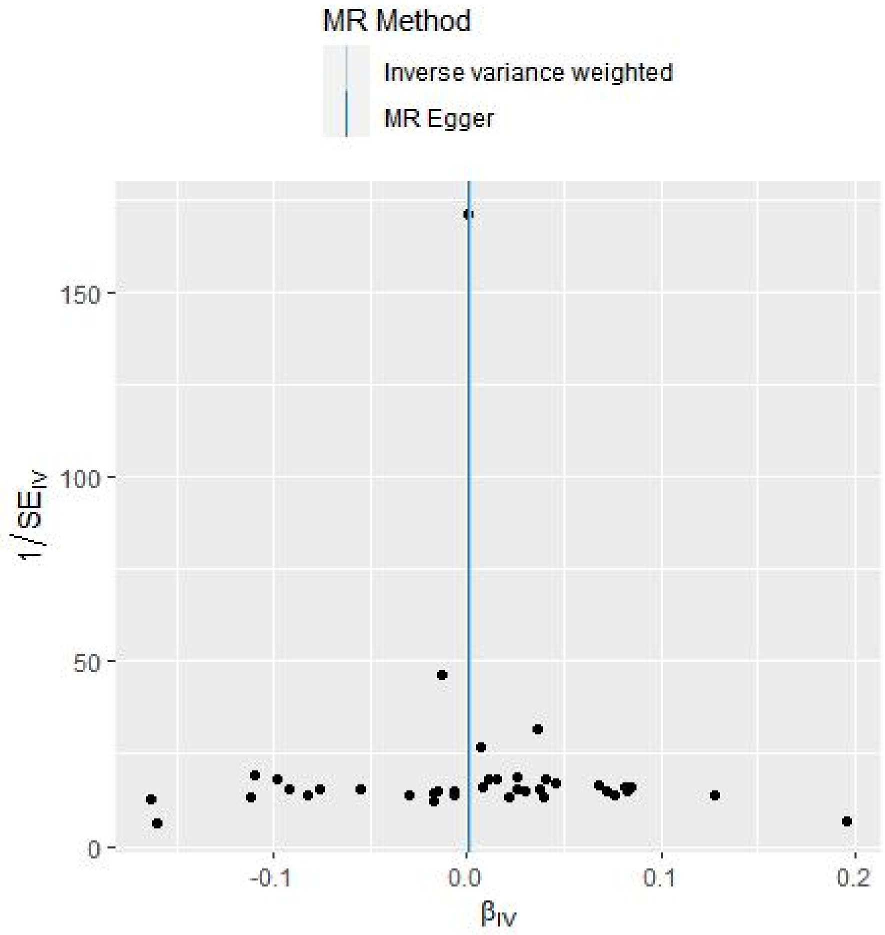

Figure 277: Leave-one-out plot to visualize causal effect of propionic acid on the risk of heart valve disease when leaving one SNP out.

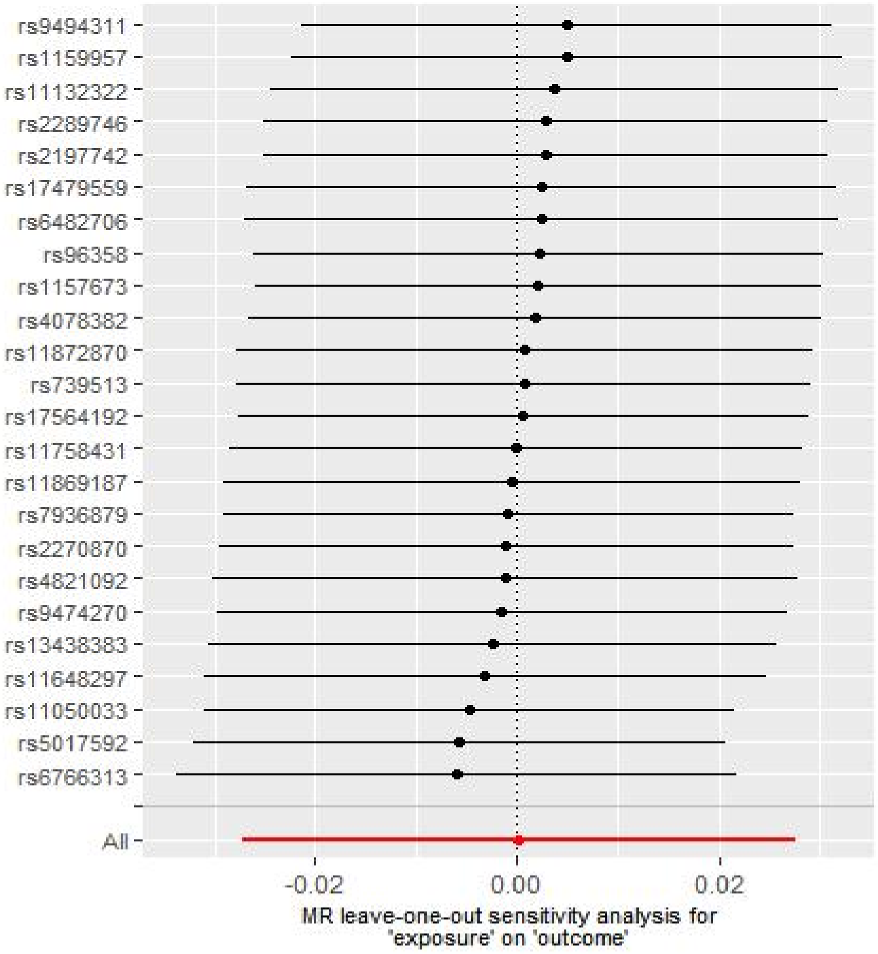

Figure 278: Funnel plots to visualize overall heterogeneity of Mendelian randomization (MR)

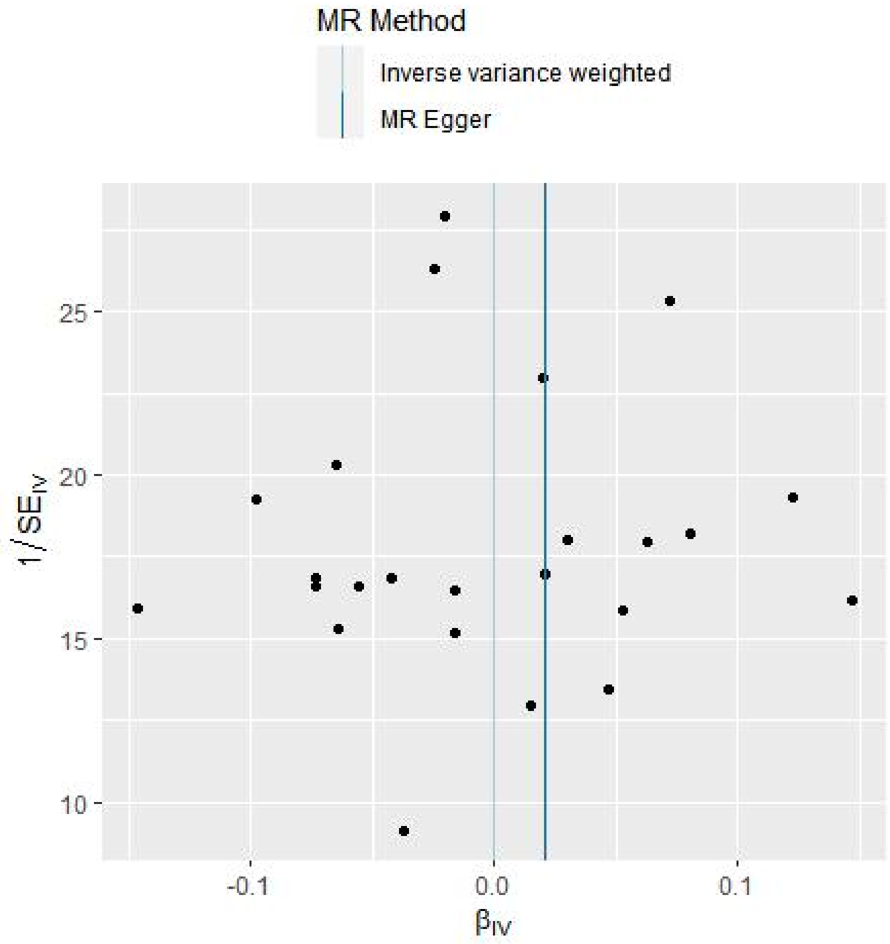

Figure 279: Leave-one-out plot to visualize causal effect of candida on the risk of atrial fibrillation when leaving one SNP out.

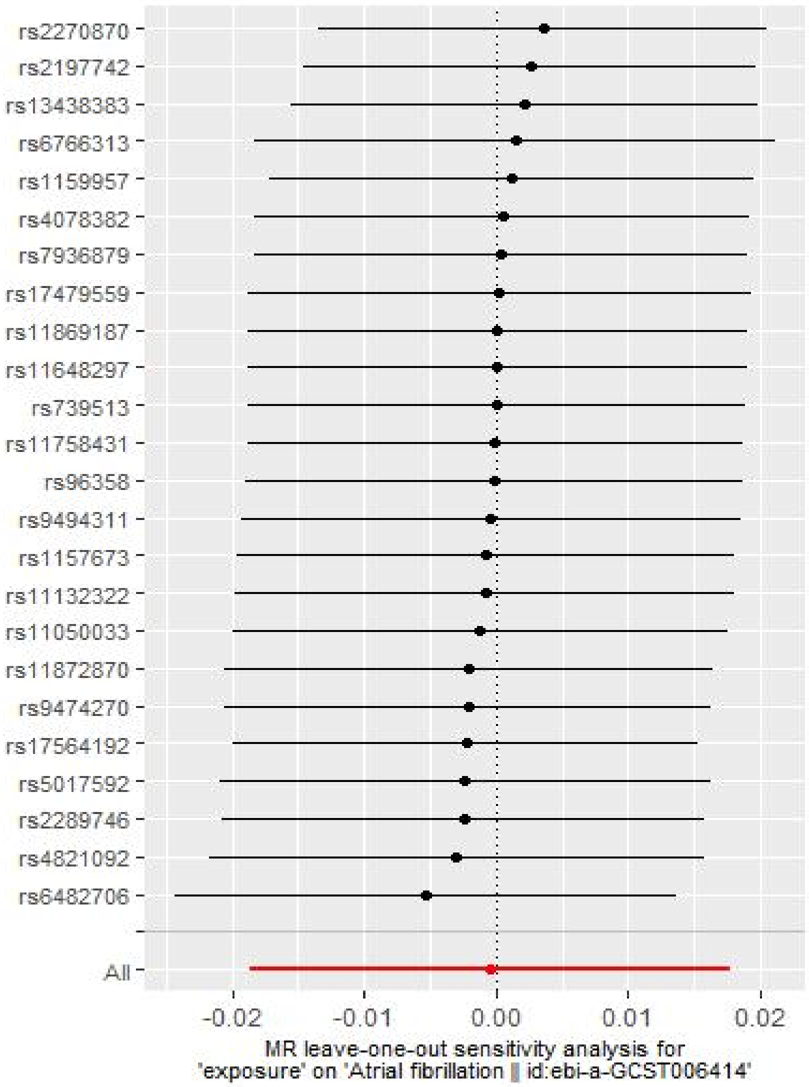

Figure 280: Funnel plots to visualize overall heterogeneity of Mendelian randomization (MR)

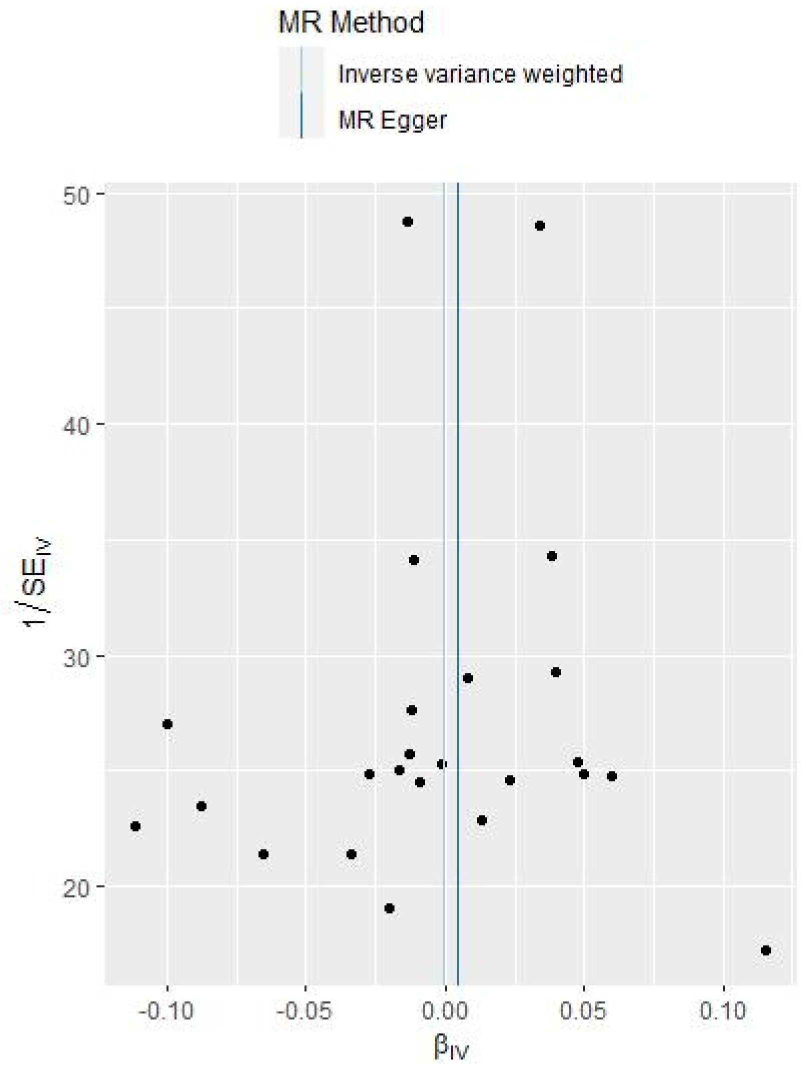

Figure 281: Leave-one-out plot to visualize causal effect of campylobacter on the risk of atrial fibrillation when leaving one SNP out.

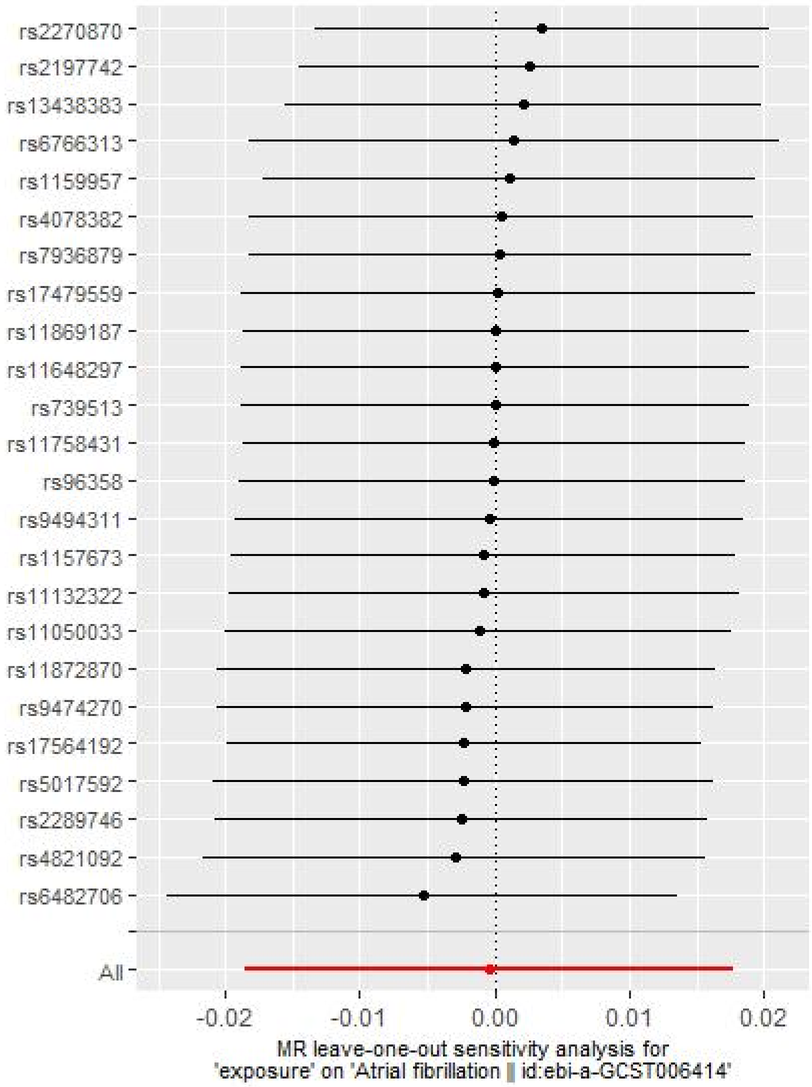

Figure 282: Funnel plots to visualize overall heterogeneity of Mendelian randomization (MR)

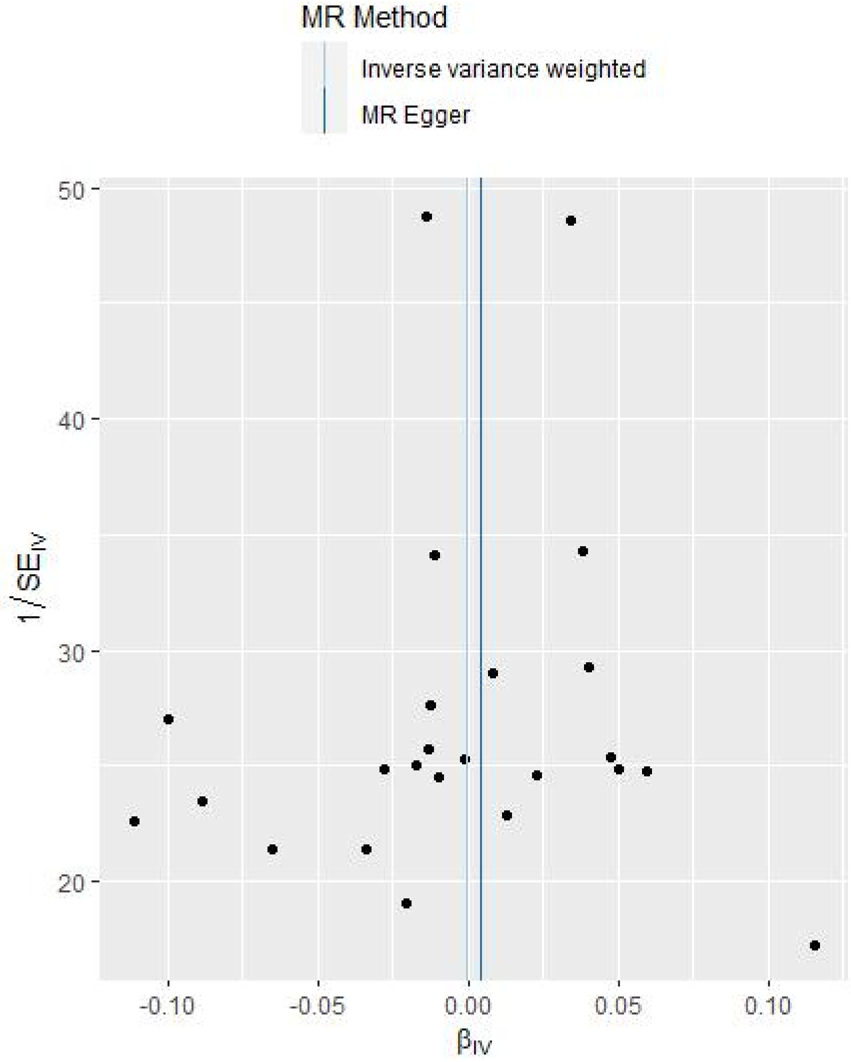

Figure 283: Leave-one-out plot to visualize causal effect of shigella on the risk of atrial fibrillation when leaving one SNP out.

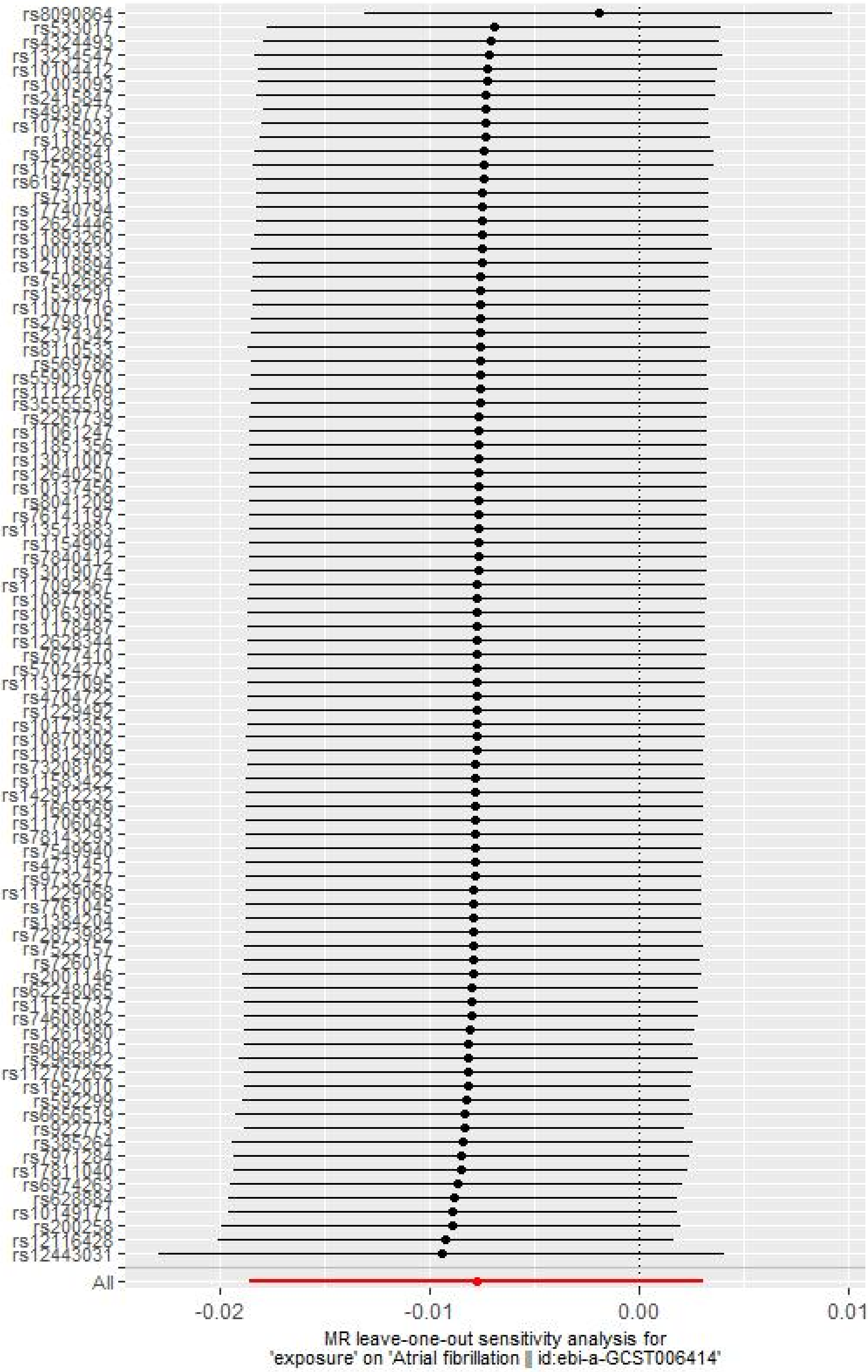

Figure 284: Funnel plots to visualize overall heterogeneity of Mendelian randomization (MR)

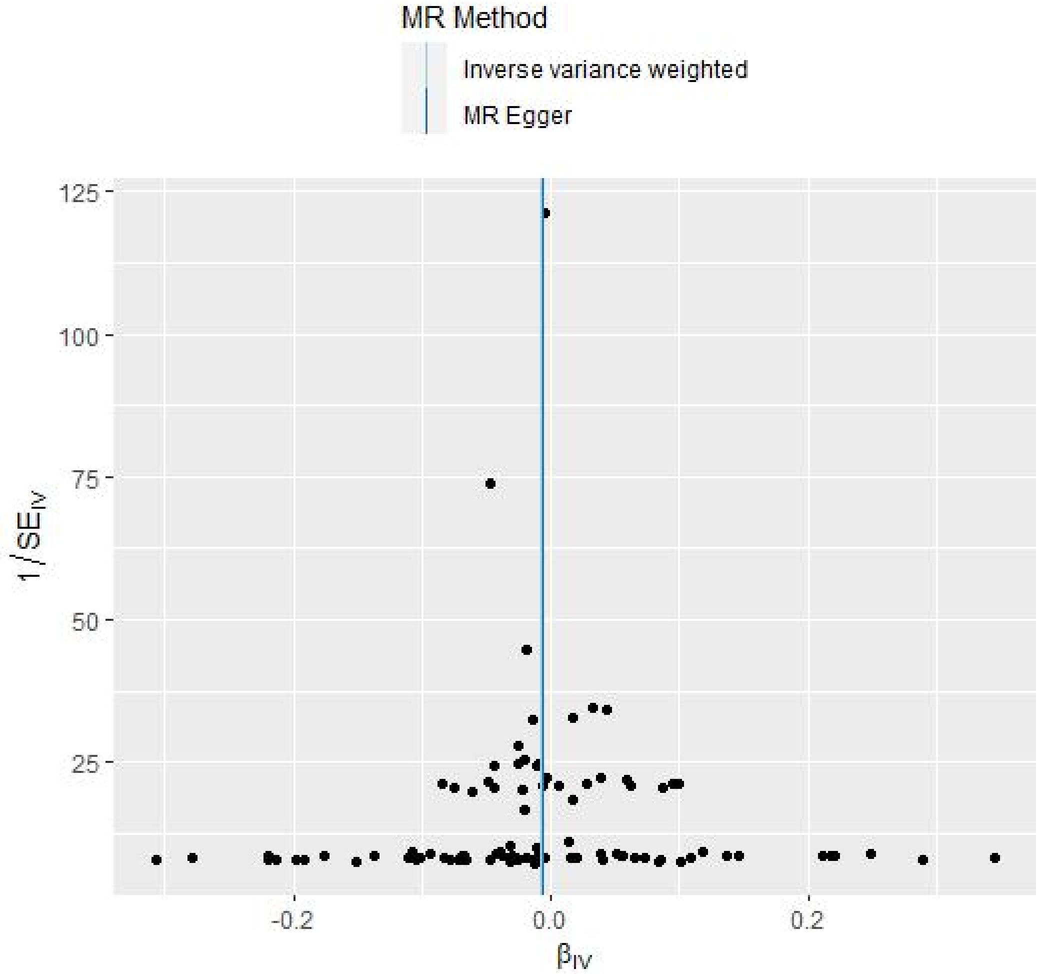

Figure 285: Leave-one-out plot to visualize causal effect of campylobacter on the risk of hypertrophic cardiomyopathy when leaving one SNP out.

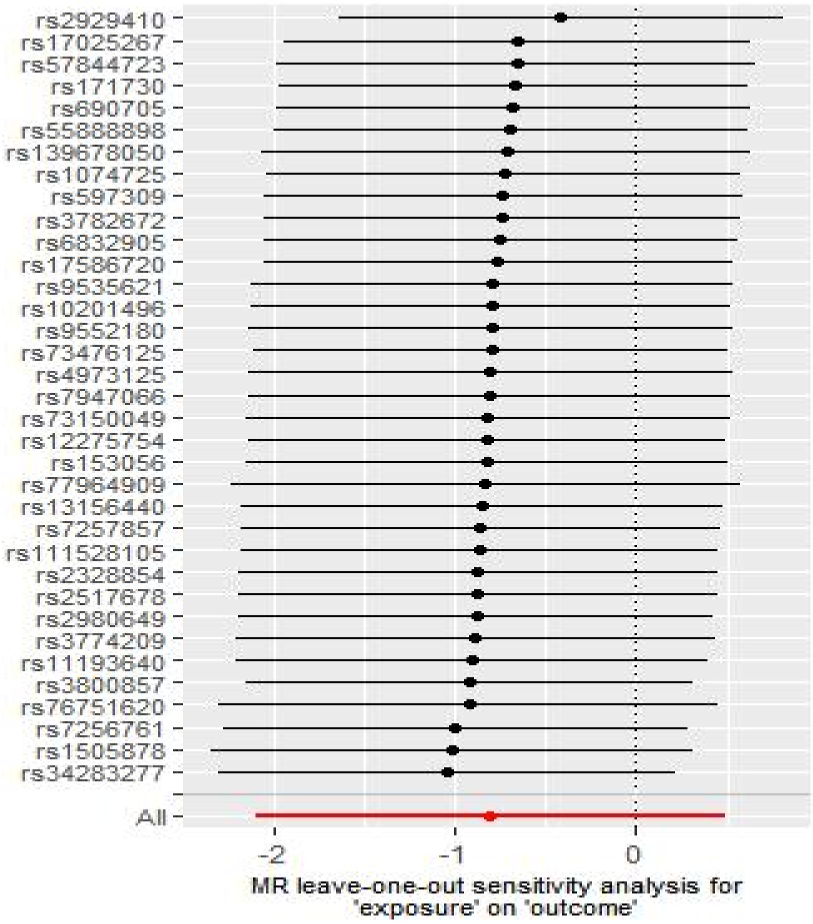

Figure 286: Funnel plots to visualize overall heterogeneity of Mendelian randomization (MR)

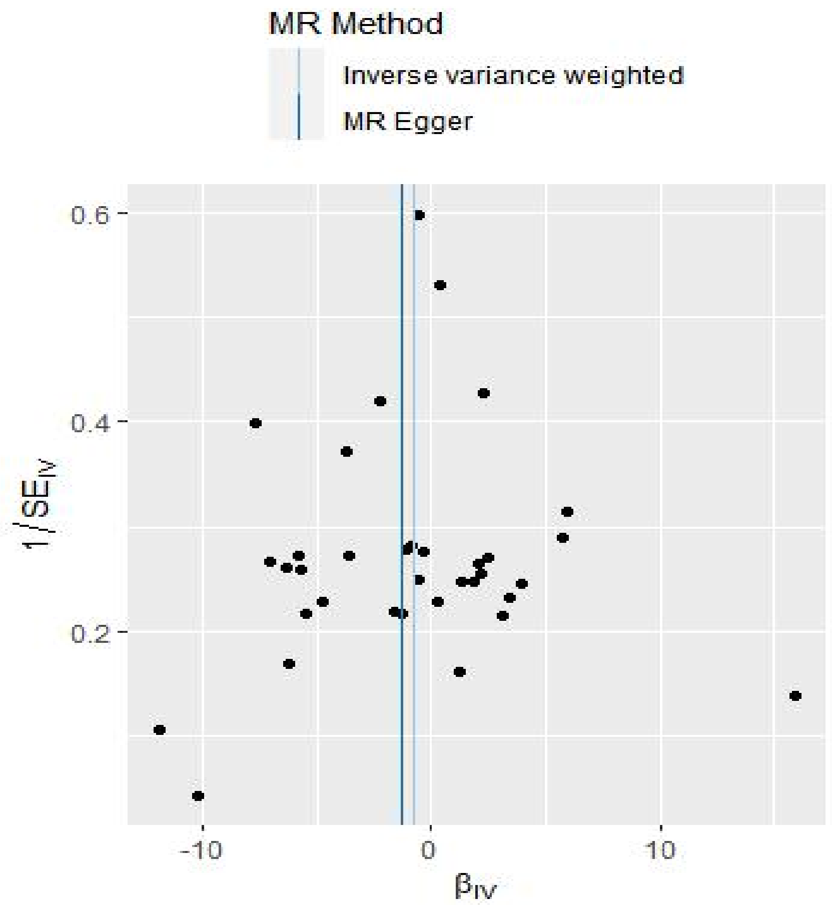

Figure 287: Leave-one-out plot to visualize causal effect of shigella on the risk of hypertrophic cardiomyopathy when leaving one SNP out.

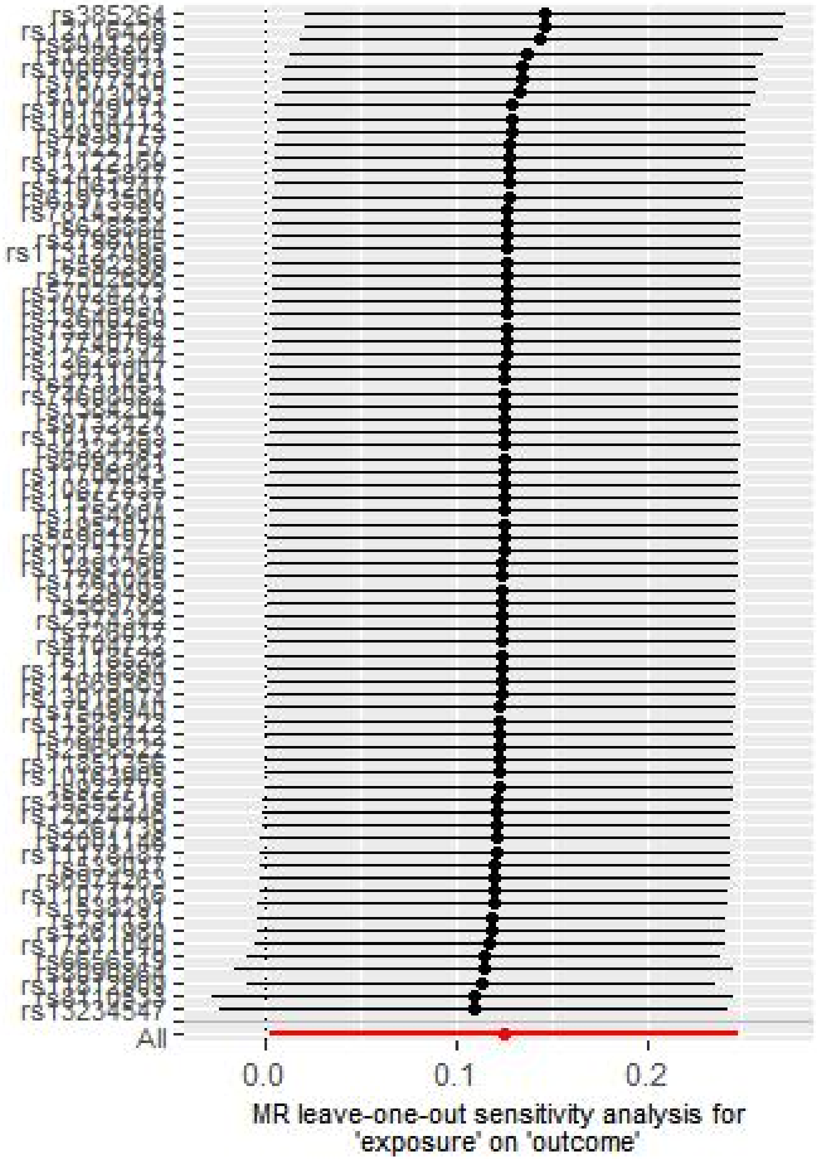

Figure 288: Funnel plots to visualize overall heterogeneity of Mendelian randomization (MR)

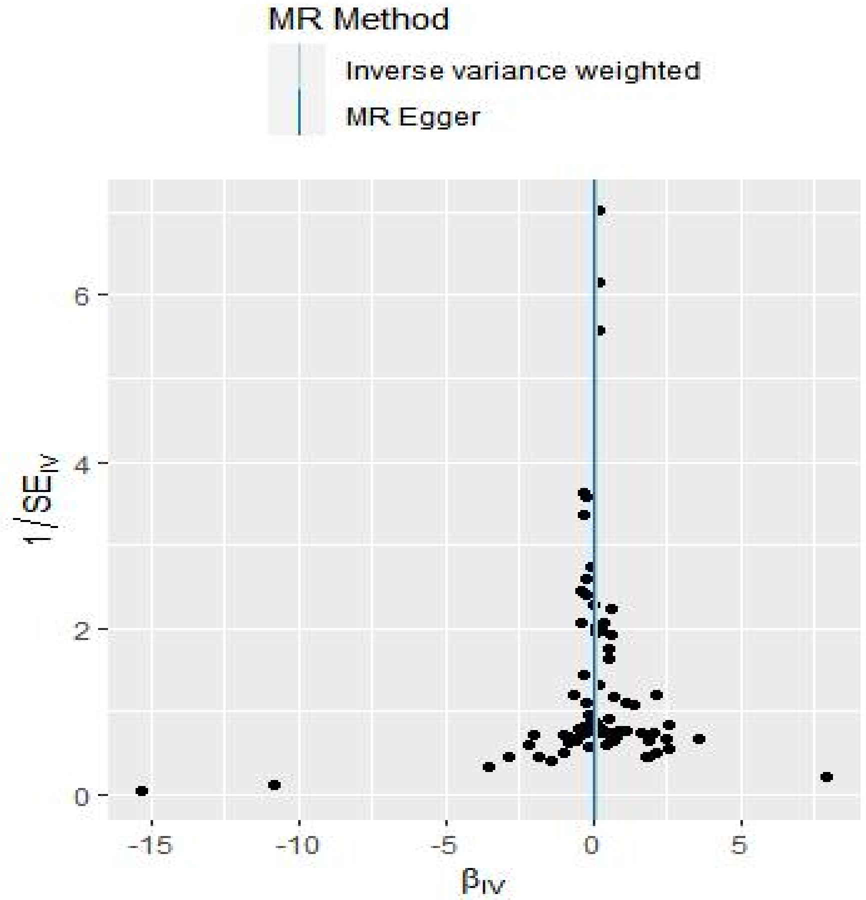

Figure 289: Leave-one-out plot to visualize causal effect of candida on the risk of hypertrophic cardiomyopathy when leaving one SNP out.

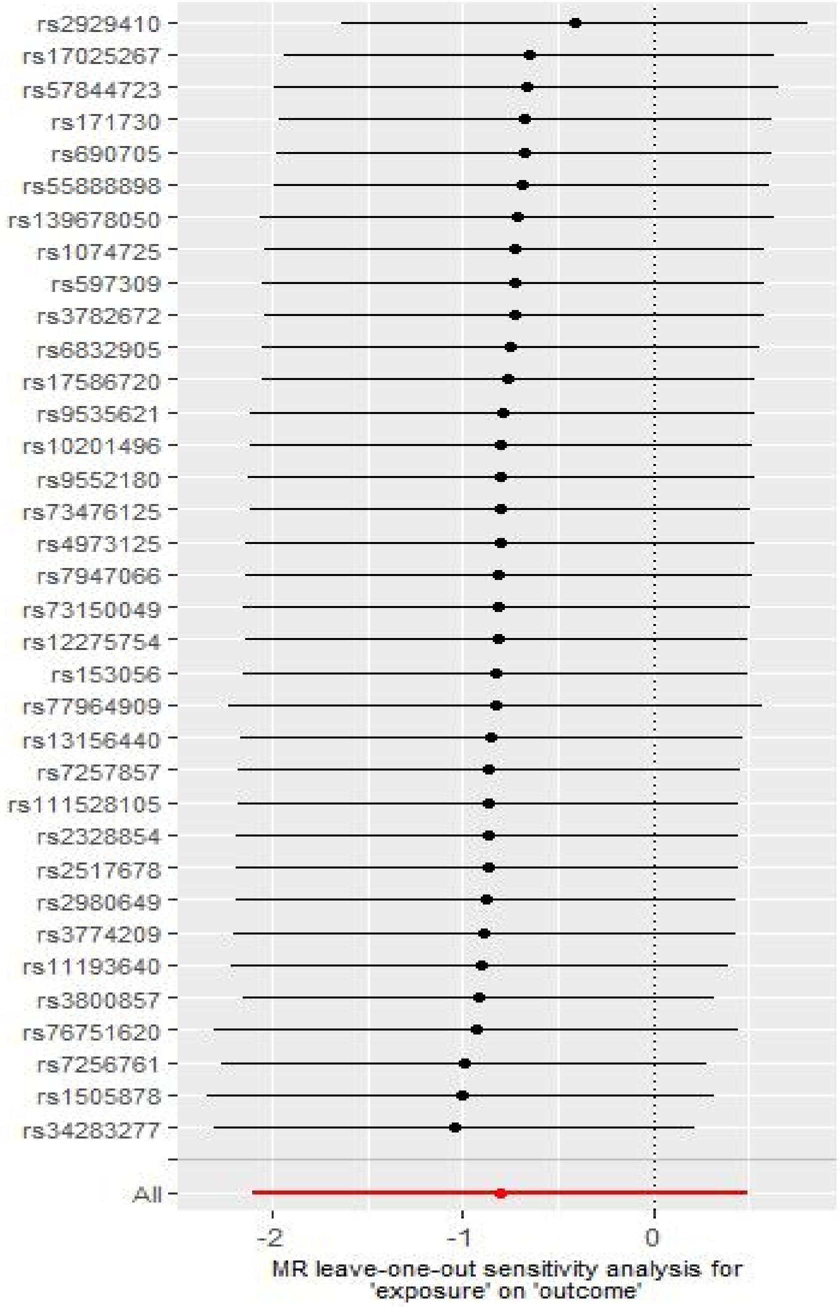

Figure 290: Funnel plots to visualize overall heterogeneity of Mendelian randomization (MR)

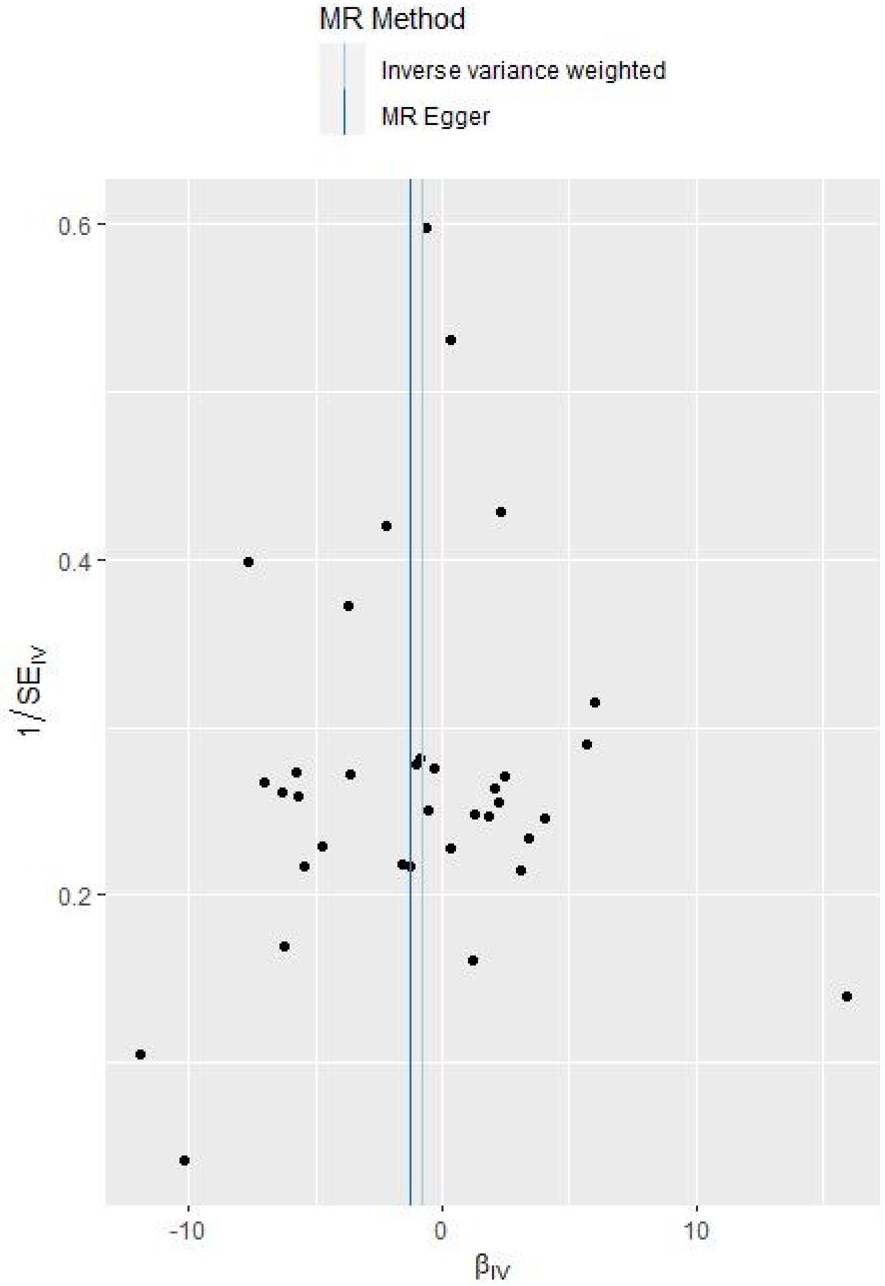

Figure 291: Leave-one-out plot to visualize causal effect of candida on the risk of coronary heart disease when leaving one SNP out.

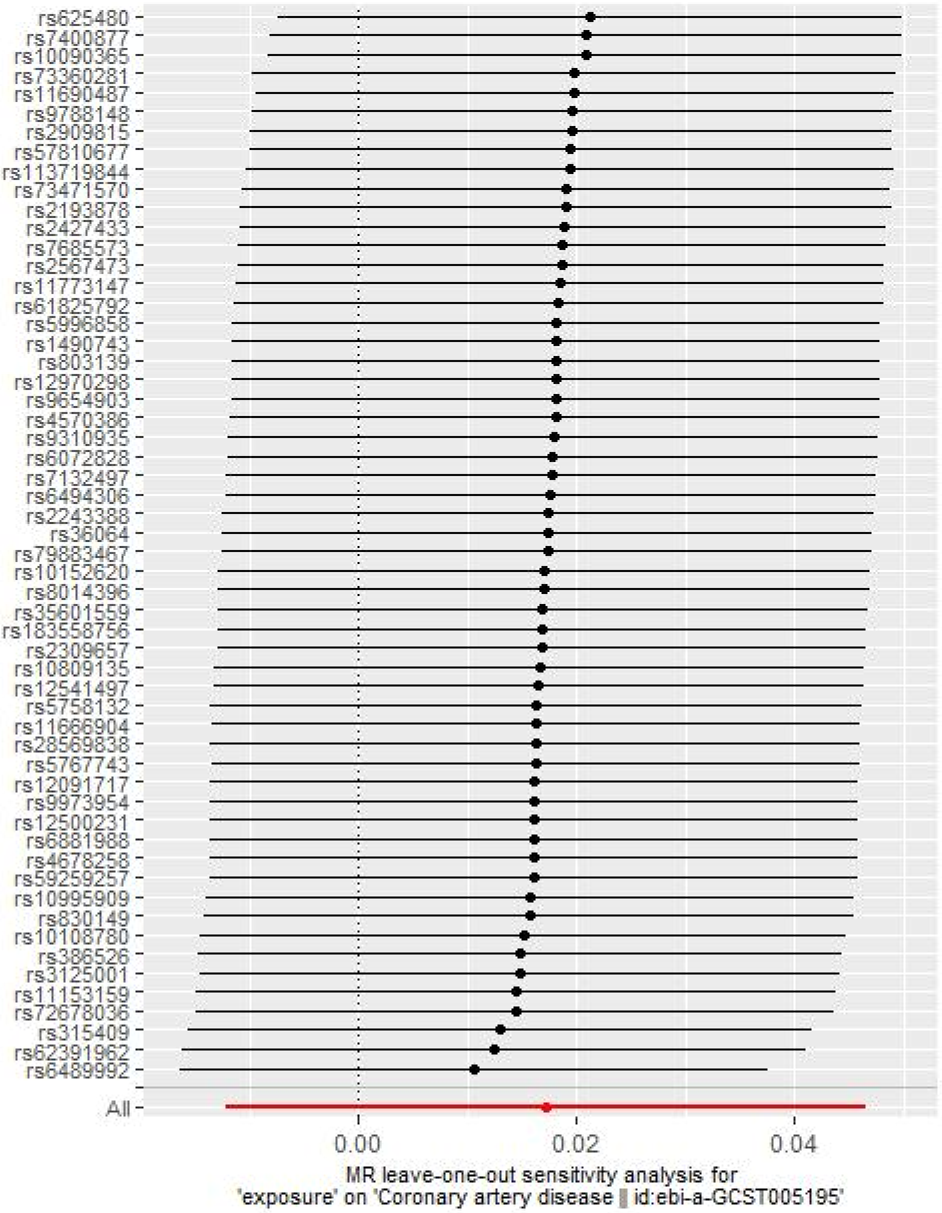

Figure 292: Funnel plots to visualize overall heterogeneity of Mendelian randomization (MR)

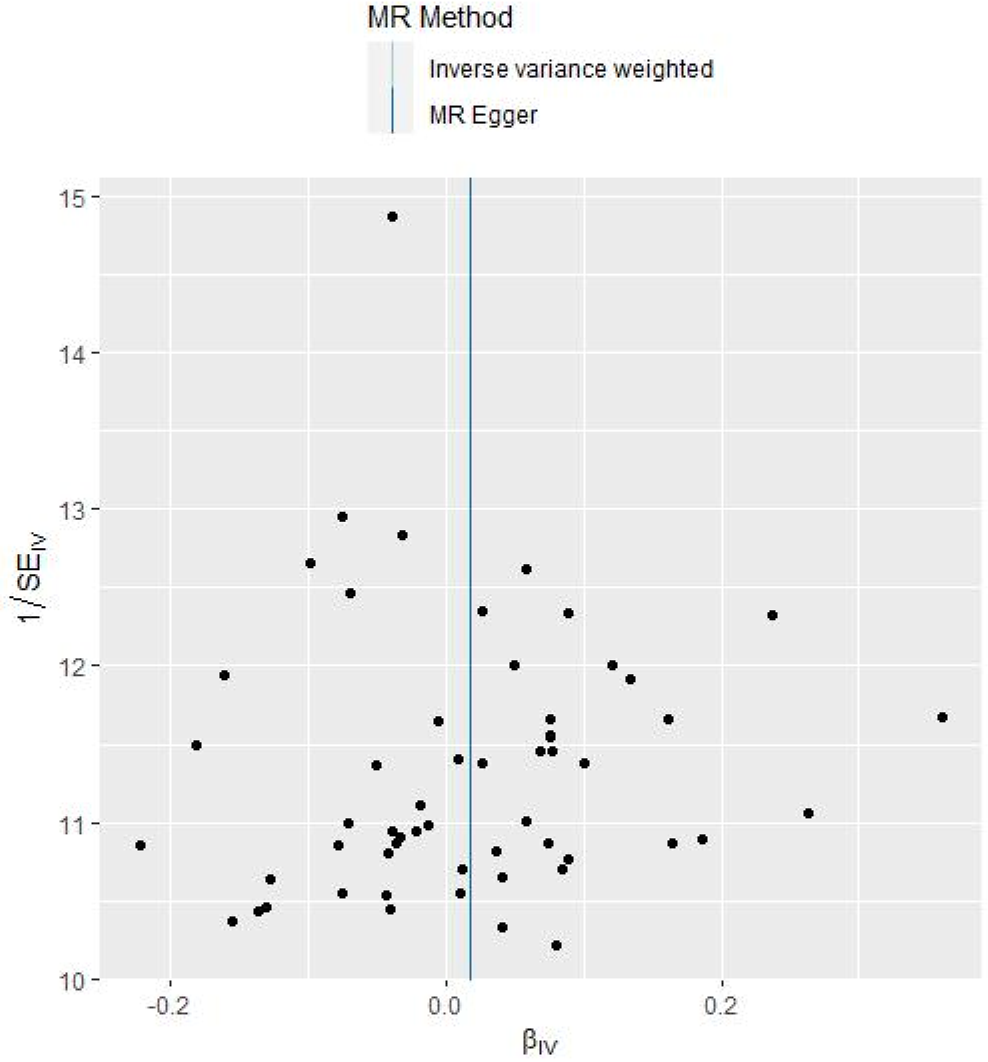

Figure 293: Leave-one-out plot to visualize causal effect of campylobacter on the risk of coronary heart disease when leaving one SNP out.

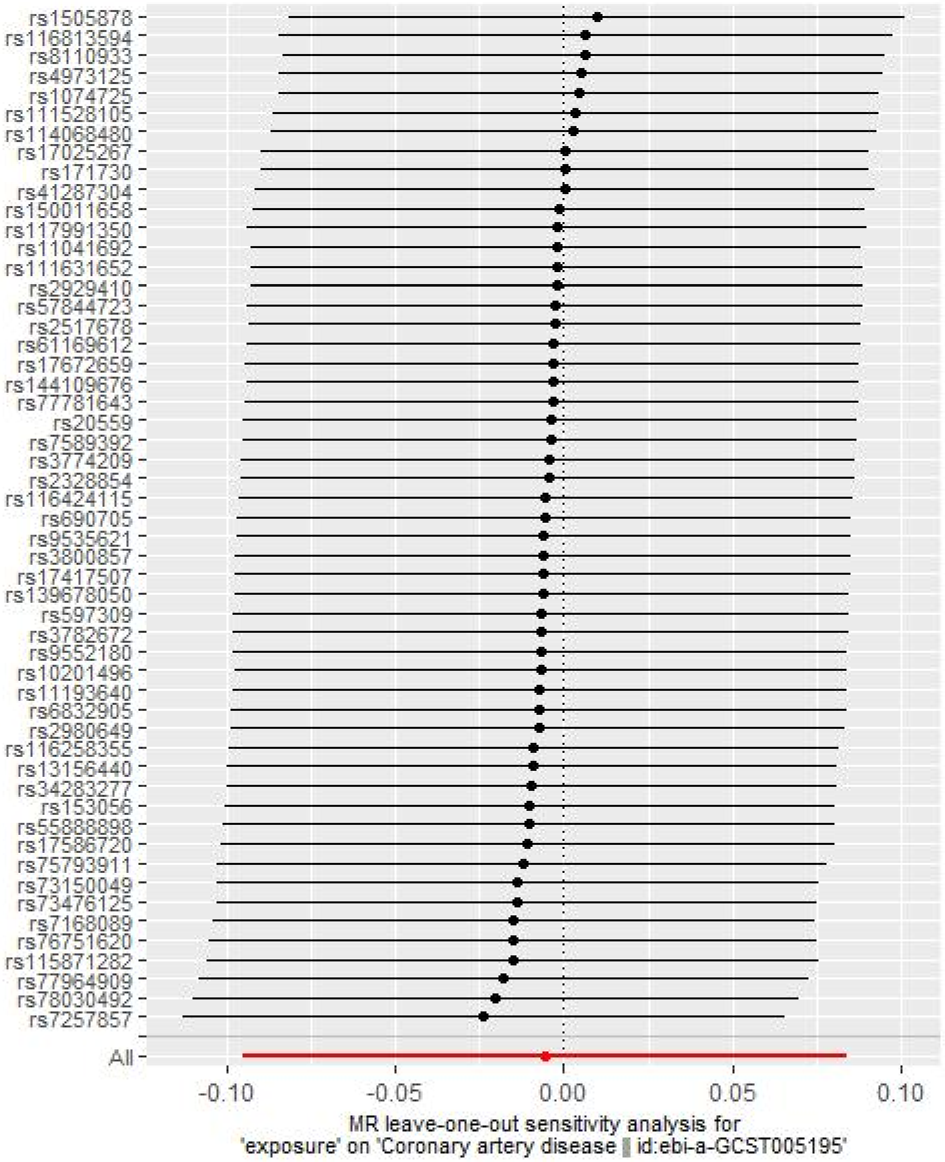

Figure 294: Funnel plots to visualize overall heterogeneity of Mendelian randomization (MR)

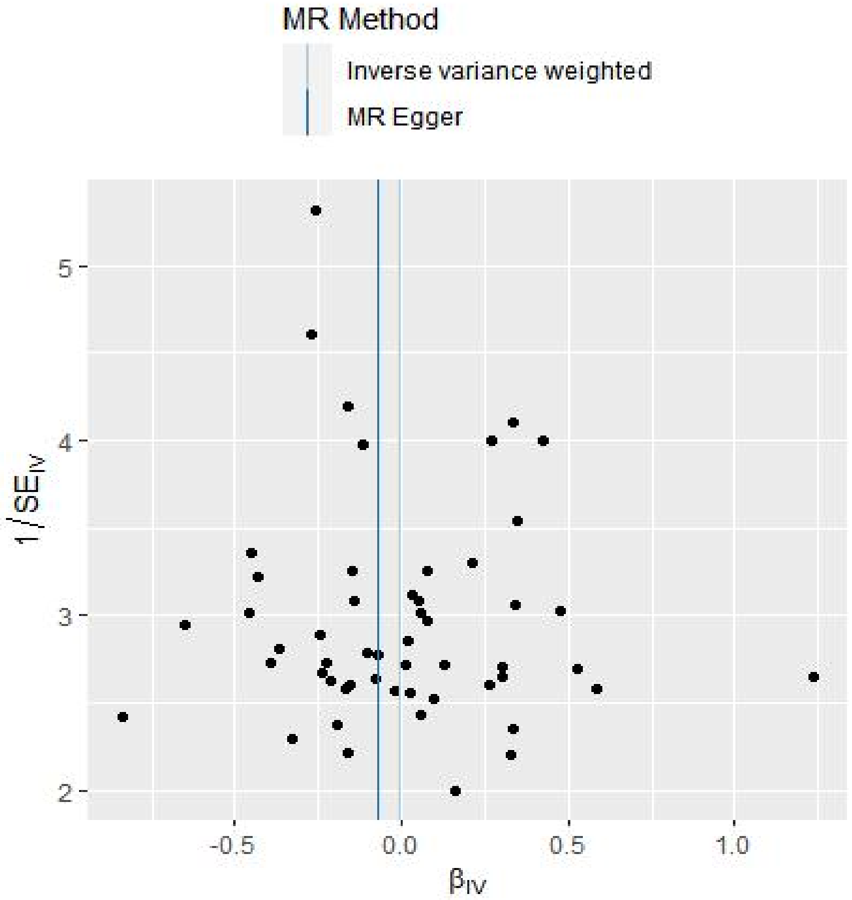

Figure 295: Leave-one-out plot to visualize causal effect of shigella on the risk of coronary heart disease when leaving one SNP out.

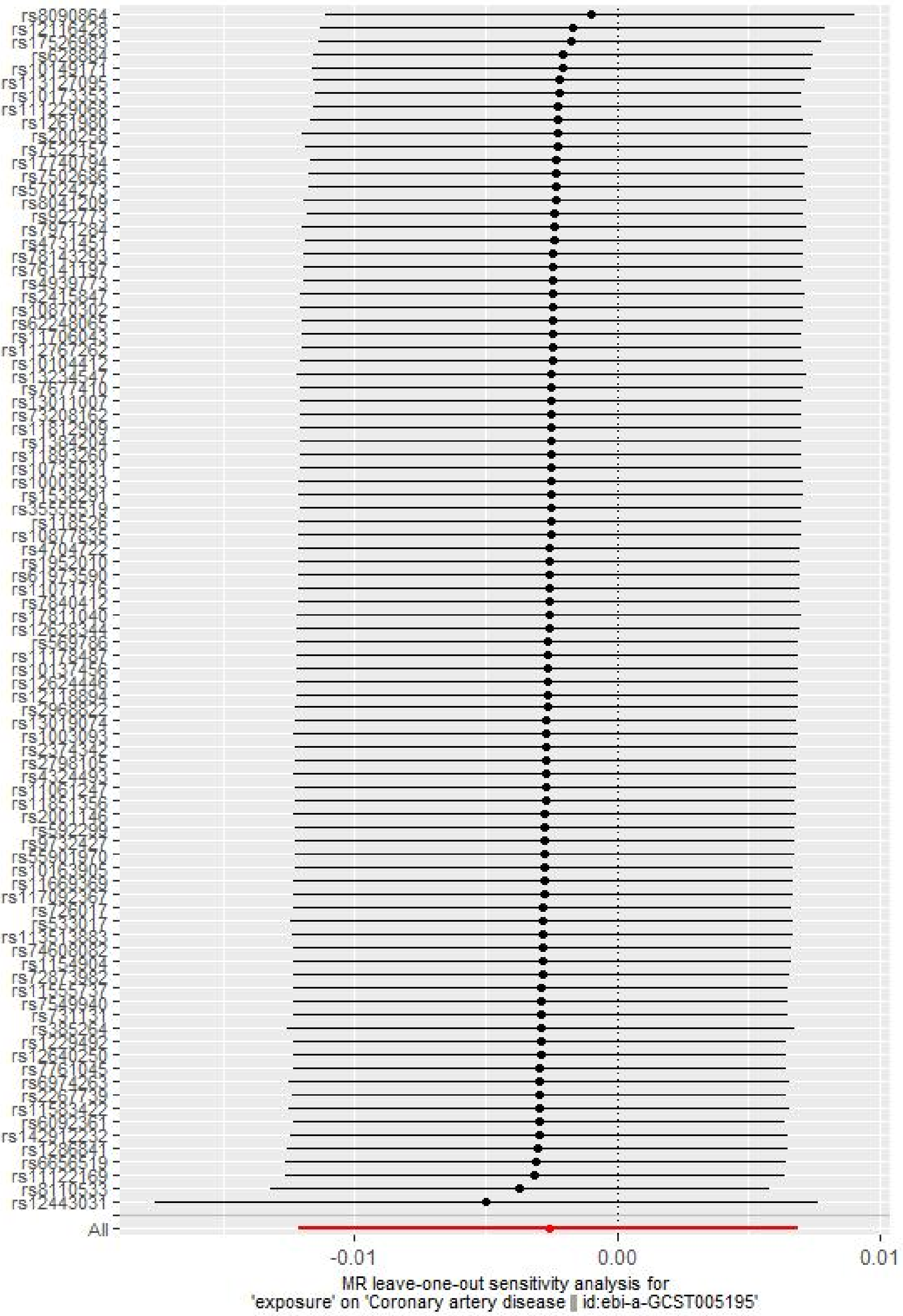

Figure 296: Funnel plots to visualize overall heterogeneity of Mendelian randomization (MR)

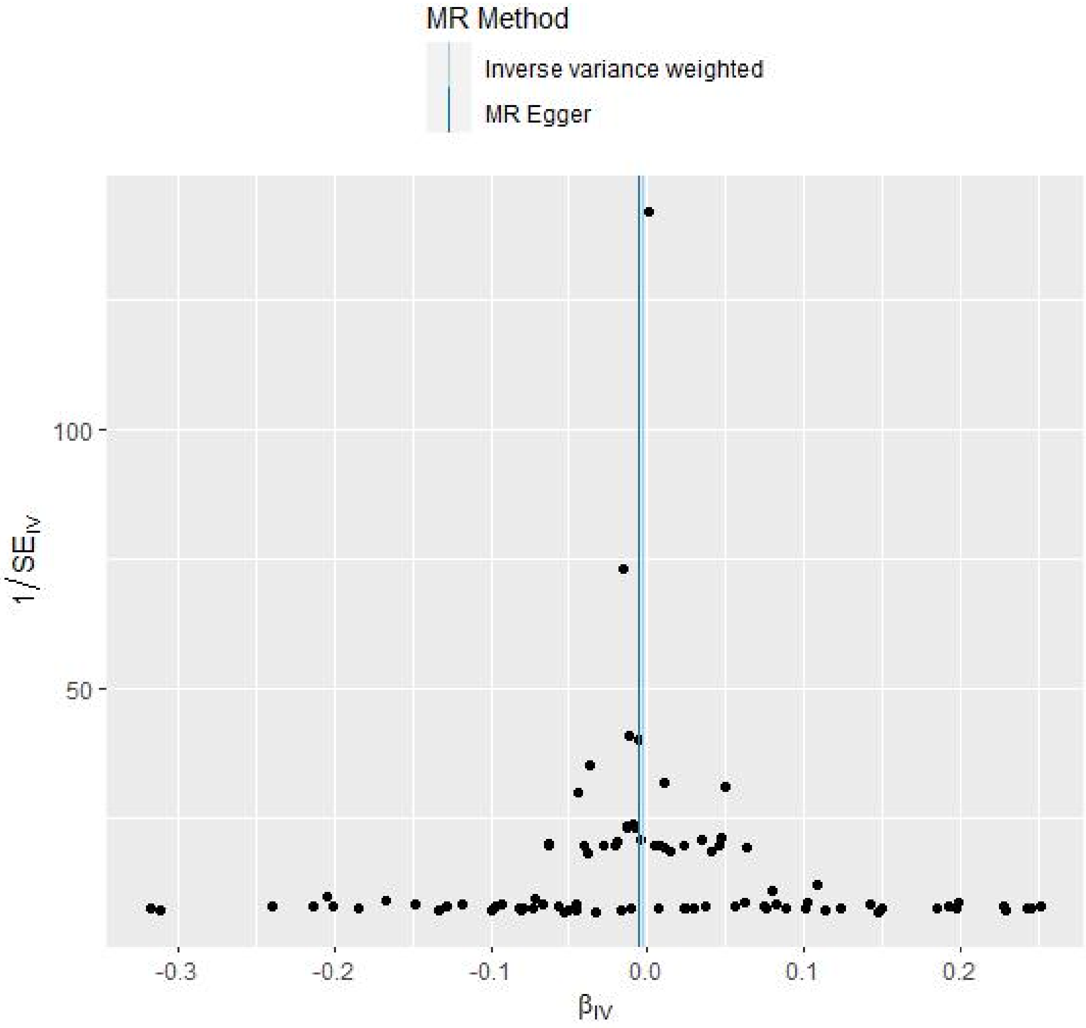

Figure 297: Leave-one-out plot to visualize causal effect of candida on the risk of dilated cardiomyopathy when leaving one SNP out.

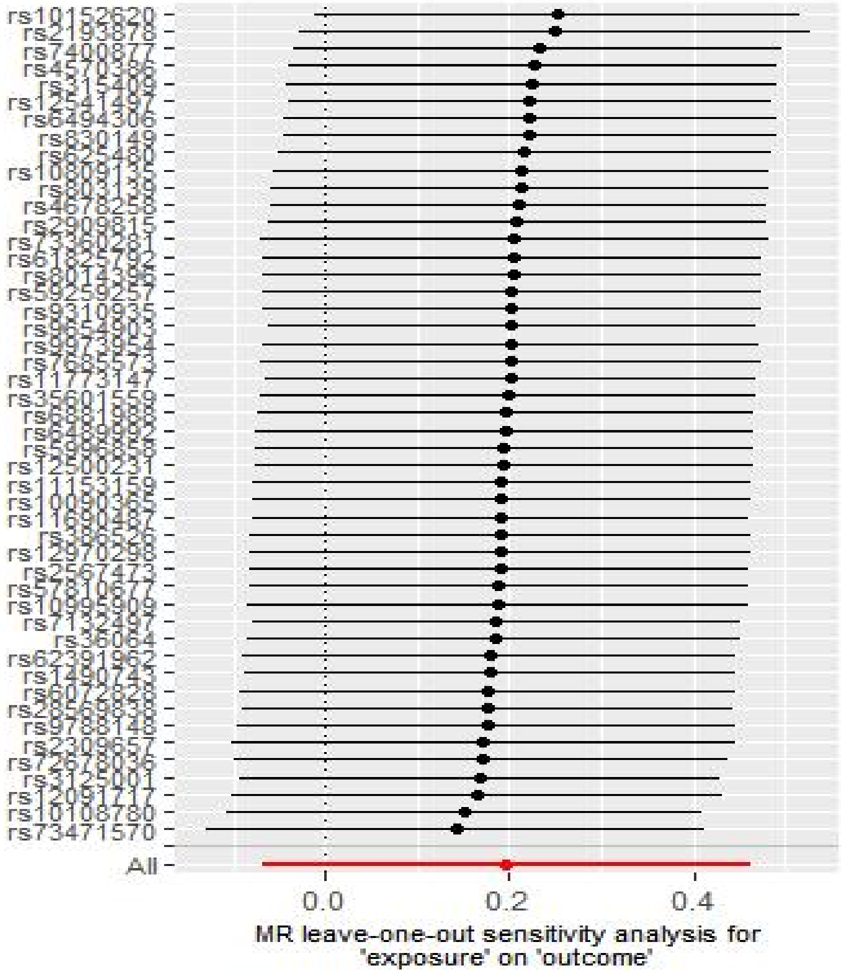

Figure 298: Funnel plots to visualize overall heterogeneity of Mendelian randomization (MR)

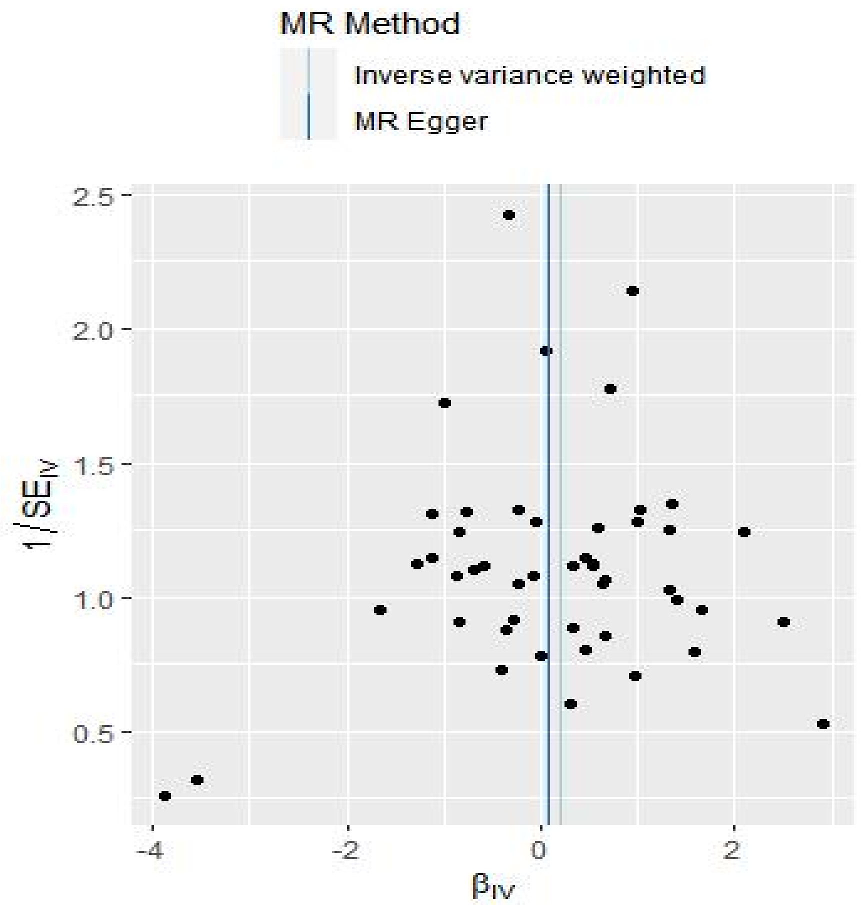

Figure 299: Leave-one-out plot to visualize causal effect of campylobacter on the risk of dilated cardiomyopathy when leaving one SNP out.

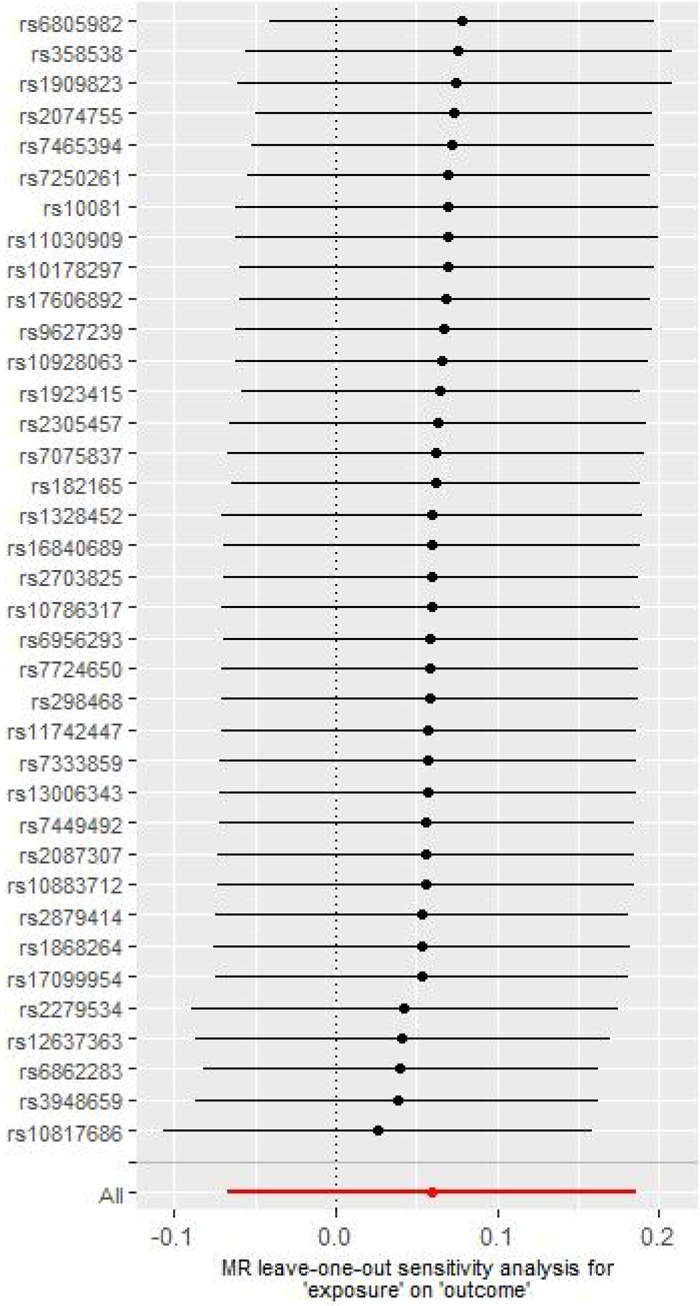

Figure 300: Funnel plots to visualize overall heterogeneity of Mendelian randomization (MR)

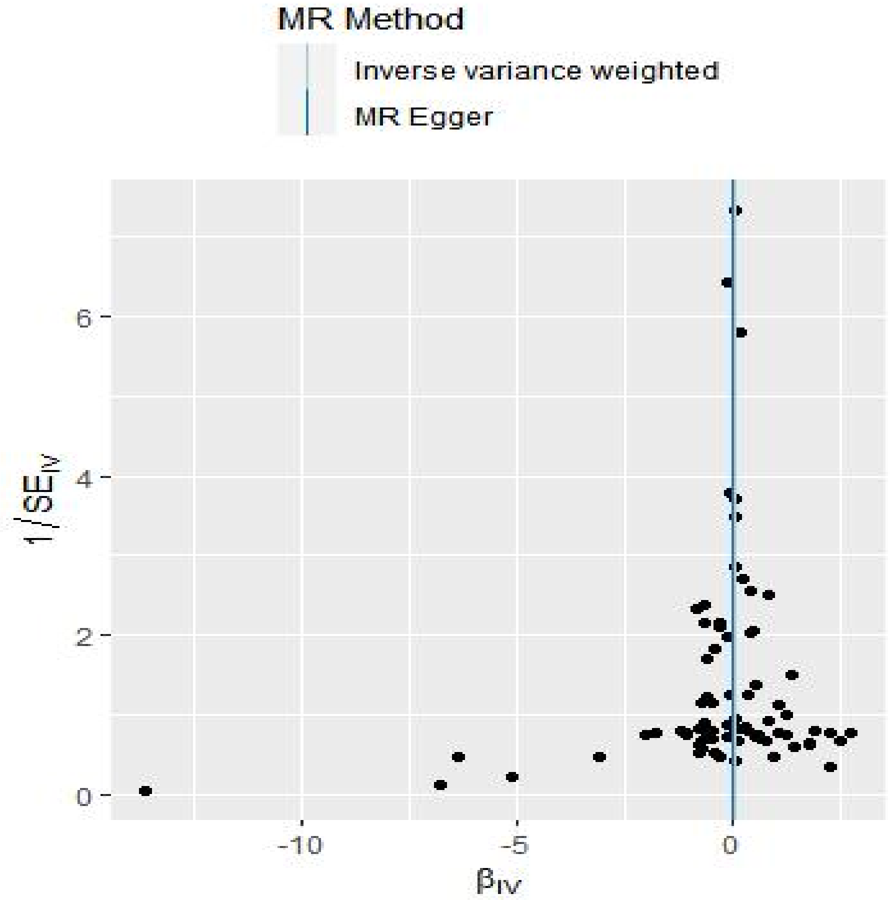

Supplement: Supplementary file 1 [file Data_Sheet_1.zip › Supplementary Material/Supplementary File 4.pdf]
