# Supplementary material for: Effects of Gut Microbiota and Metabolites on Heart Failure and Its Risk Factors: A Two-Sample Mendelian Randomization Study
Source: Front Nutr. 2022 Jun 20;9:899746. doi: 10.3389/fnut.2022.899746 (PMC9253861; doi:10.3389/fnut.2022.899746)

Supplementary Information

Effects of Gut Microbiota and Metabolites on Heart Failure and Its Risk Factors: A Two-sample Mendelian Randomization Study

Qiang Luo[a], Yilan Hu[a], Xin Chen[a], Yong Luo[a], Jie Chen[a], Han Wang[a, *]

Figure 1-24: Effects of gut metabolites and heart failure.

Figure 25-48: Effects of gut metabolites and atrial fibrillation

Figure 49-72: Effects of gut metabolites and hypertrophic cardiomyopathy

Figure 73-96: Effects of gut metabolites and coronary heart disease

Figure 97-120: Effects of gut metabolites and dilated cardiomyopathy

Figure 121-144: Effects of gut metabolites and chronic kidney disease

Figure 145-168: Effects of gut metabolites and systolic blood pressure

Figure 169-192: Effects of gut metabolites and diastolic blood pressure

Figure 193-216: Effects of gut metabolites and diabetes

Figure 217-240: Effects of gut metabolites and myocardial infarction

Figure 241-254: Effects of gut metabolites and myocarditis

Figure 255-278: Effects of gut metabolites and valvular heart disease

Figure 279-284: Effects of gut microbiota and atrial fibrillation

Figure 285-290: Effects of gut microbiota and hypertrophic cardiomyopathy

Figure 291-296: Effects of gut microbiota and coronary heart disease

Figure 297-302: Effects of gut microbiota and dilated cardiomyopathy

Figure 303-308: Effects of gut microbiota and chronic kidney disease

Figure 309-314: Effects of gut microbiota and systolic blood pressure

Figure 315-320: Effects of gut microbiota and diastolic blood pressure

Figure 321-326: Effects of gut microbiota and diabetes

Figure 327-322: Effects of gut microbiota and valvular heart disease

Figure 333-338: Effects of gut microbiota and myocardial infarction

Figure 339-342: Effects of gut microbiota and myocarditis

Figure 343-348: Effects of gut microbiota and heart failure

Figure 301: Leave-one-out plot to visualize causal effect of shigella on the risk of dilated cardiomyopathy when leaving one SNP out.

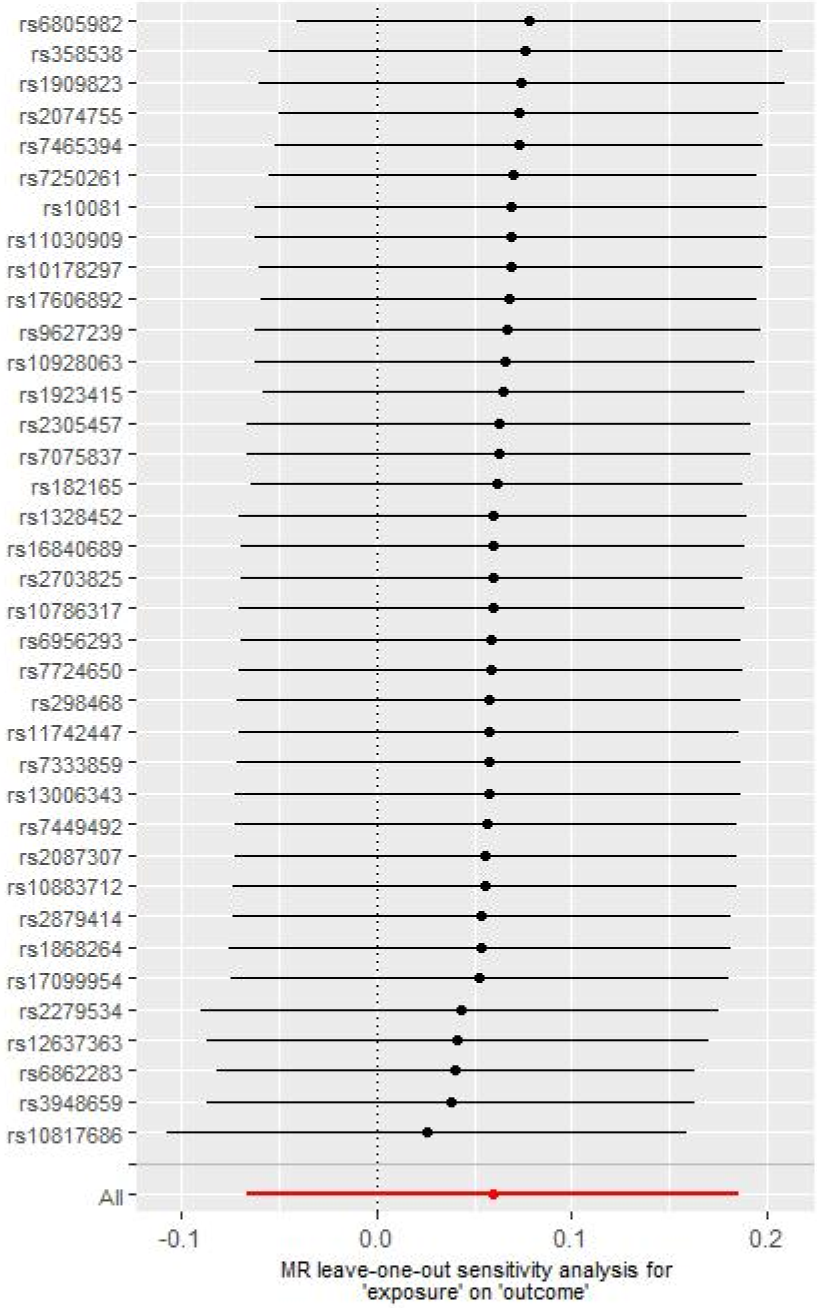

Figure 302: Funnel plots to visualize overall heterogeneity of Mendelian randomization (MR)

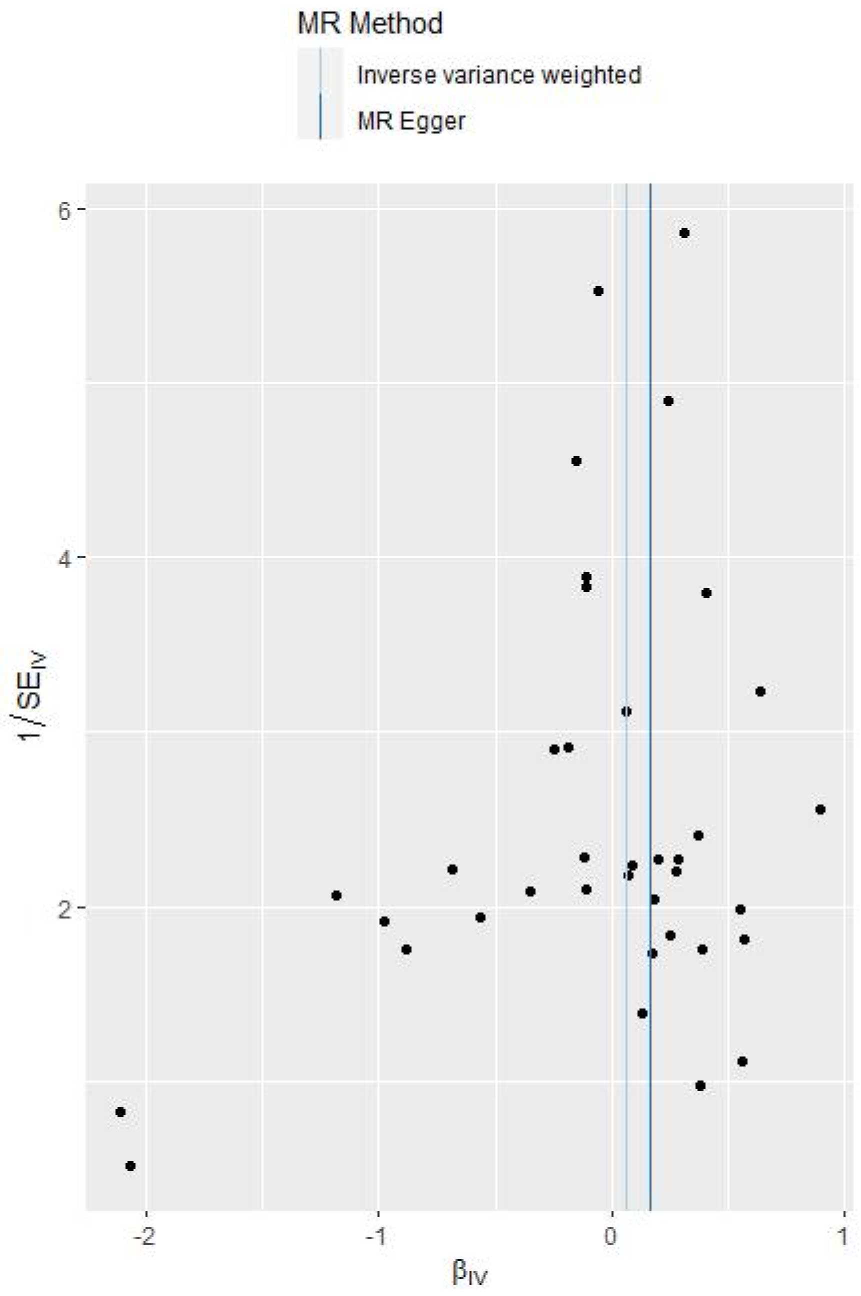

Figure 303: Leave-one-out plot to visualize causal effect of candida on the risk of chronic kidney disease when leaving one SNP out.

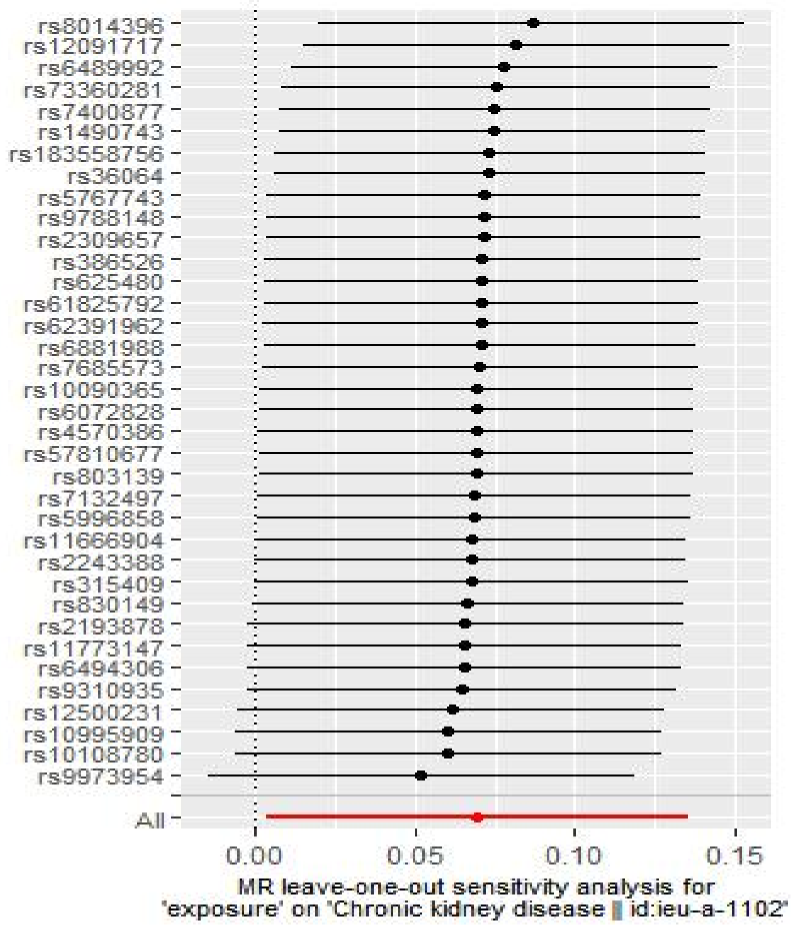

Figure 304: Funnel plots to visualize overall heterogeneity of Mendelian randomization (MR)

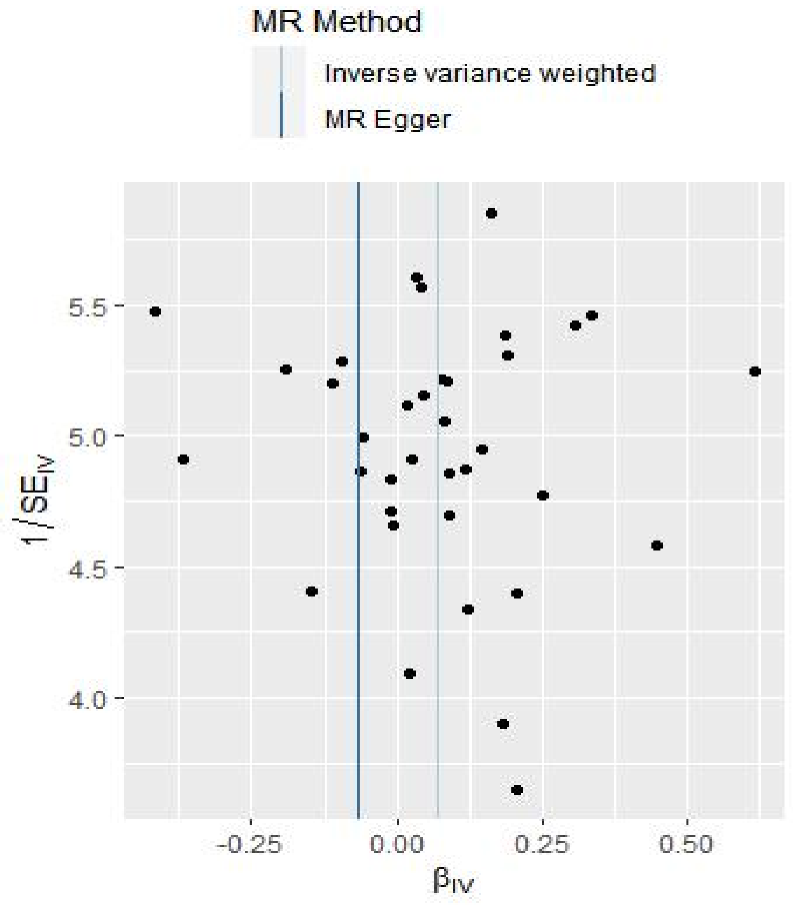

Figure 305: Leave-one-out plot to visualize causal effect of campylobacter on the risk of chronic kidney disease when leaving one SNP out.

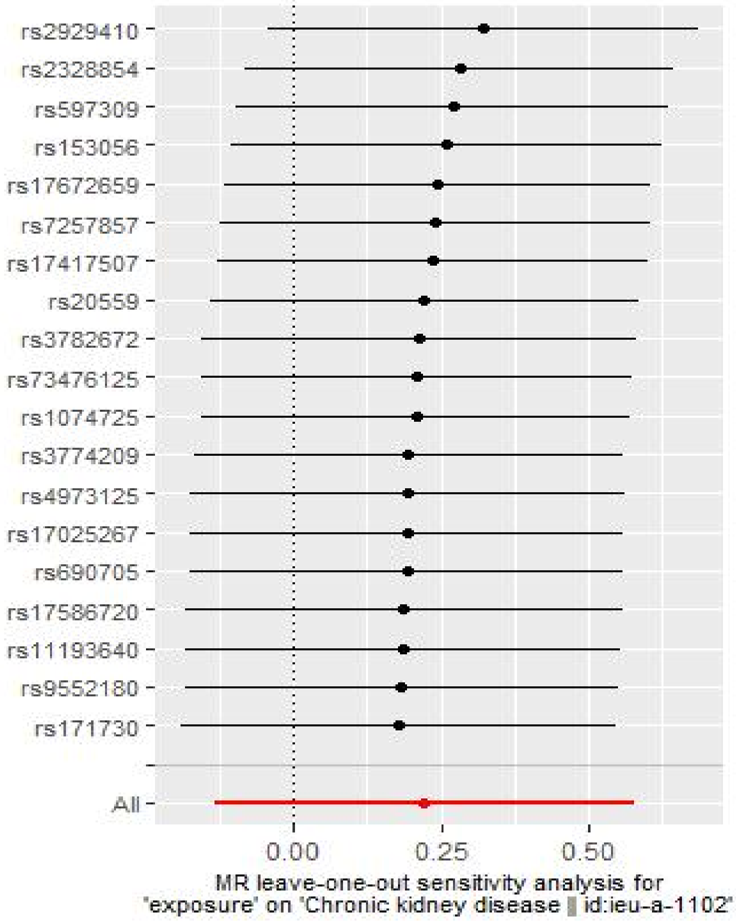

Figure 306: Funnel plots to visualize overall heterogeneity of Mendelian randomization (MR)

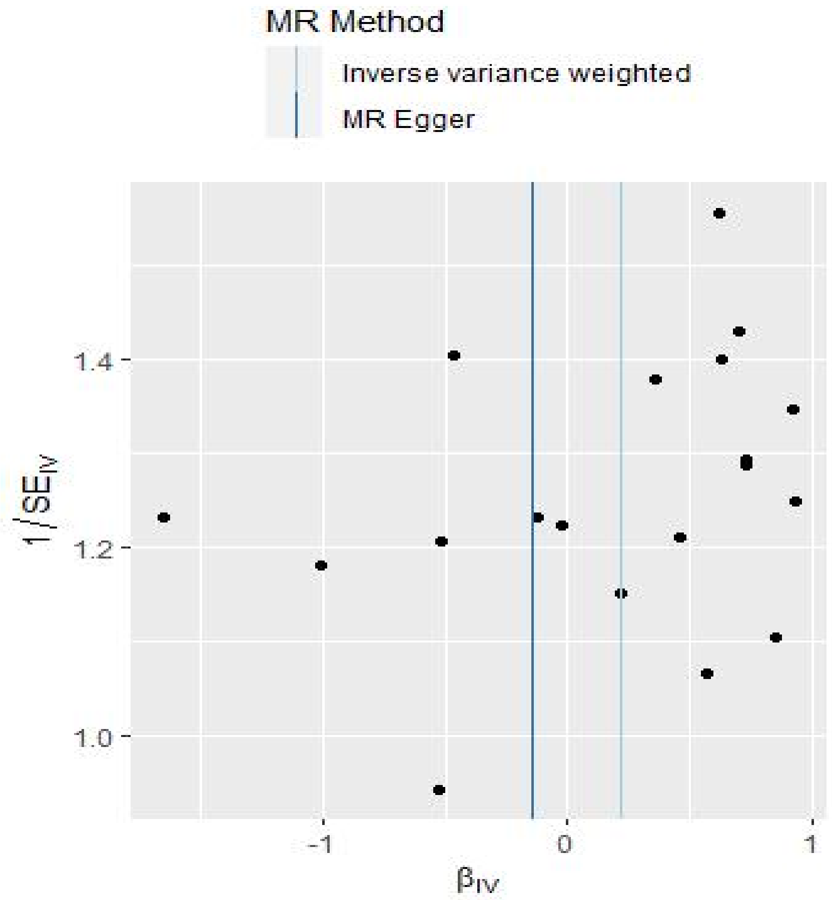

Figure 307: Leave-one-out plot to visualize causal effect of shigella on the risk of chronic kidney disease when leaving one SNP out.

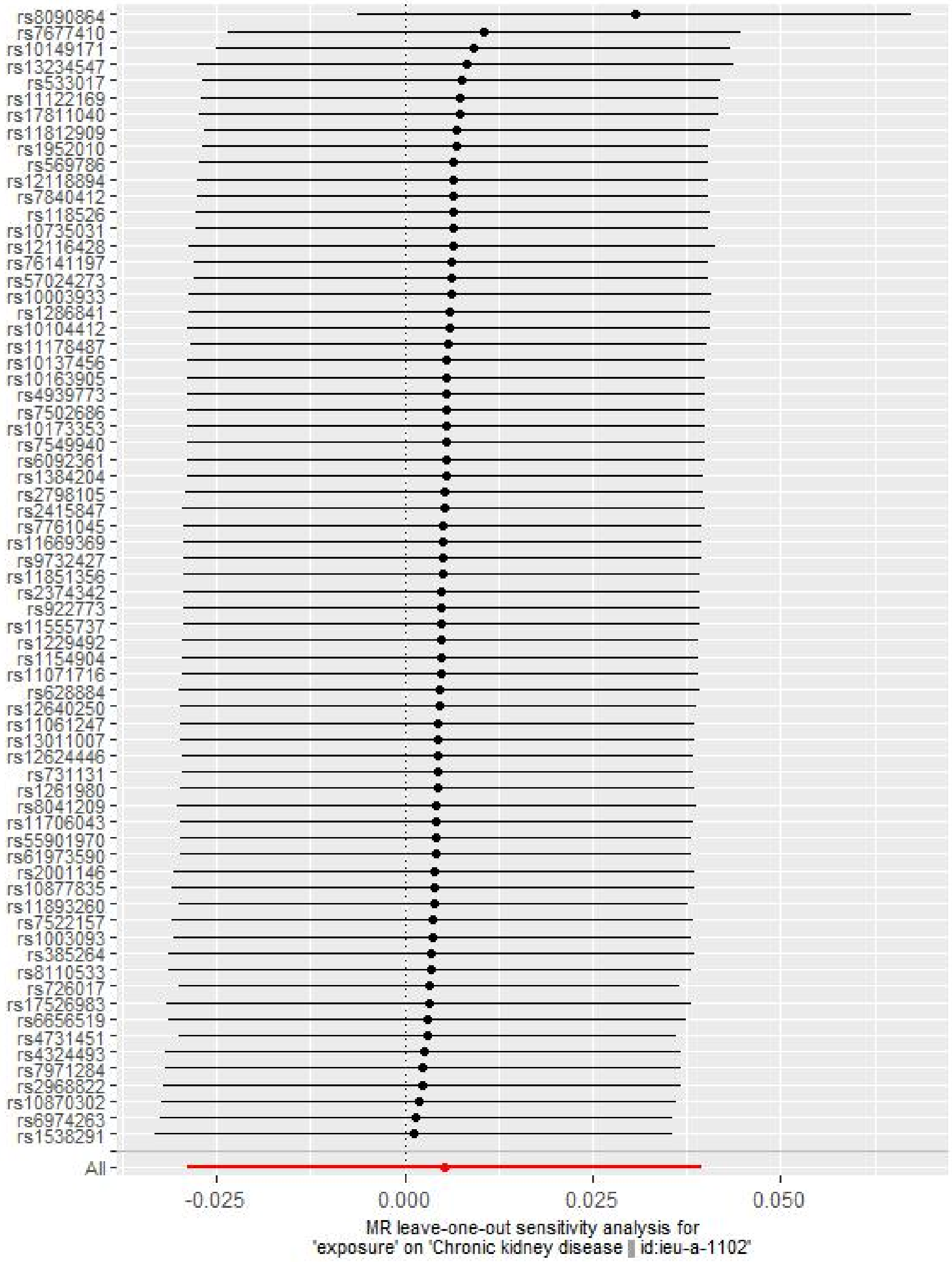

Figure 308: Funnel plots to visualize overall heterogeneity of Mendelian randomization (MR)

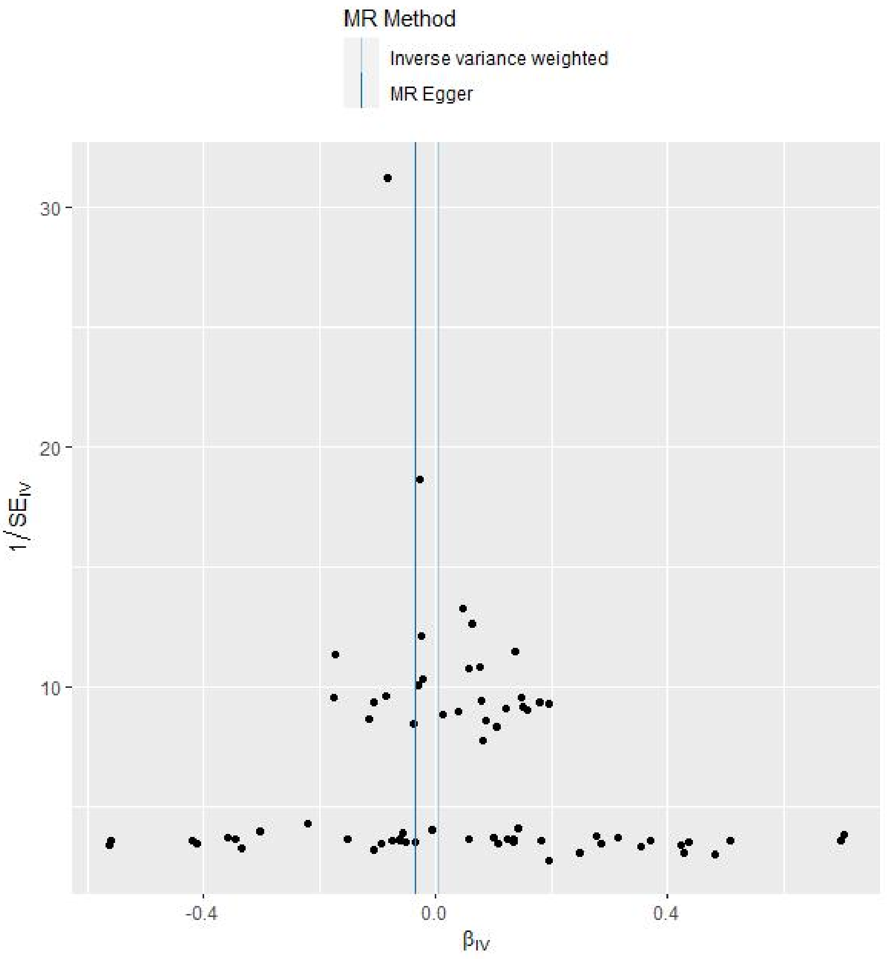

Figure 309: Leave-one-out plot to visualize causal effect of candida on the risk of systolic blood pressure when leaving one SNP out.

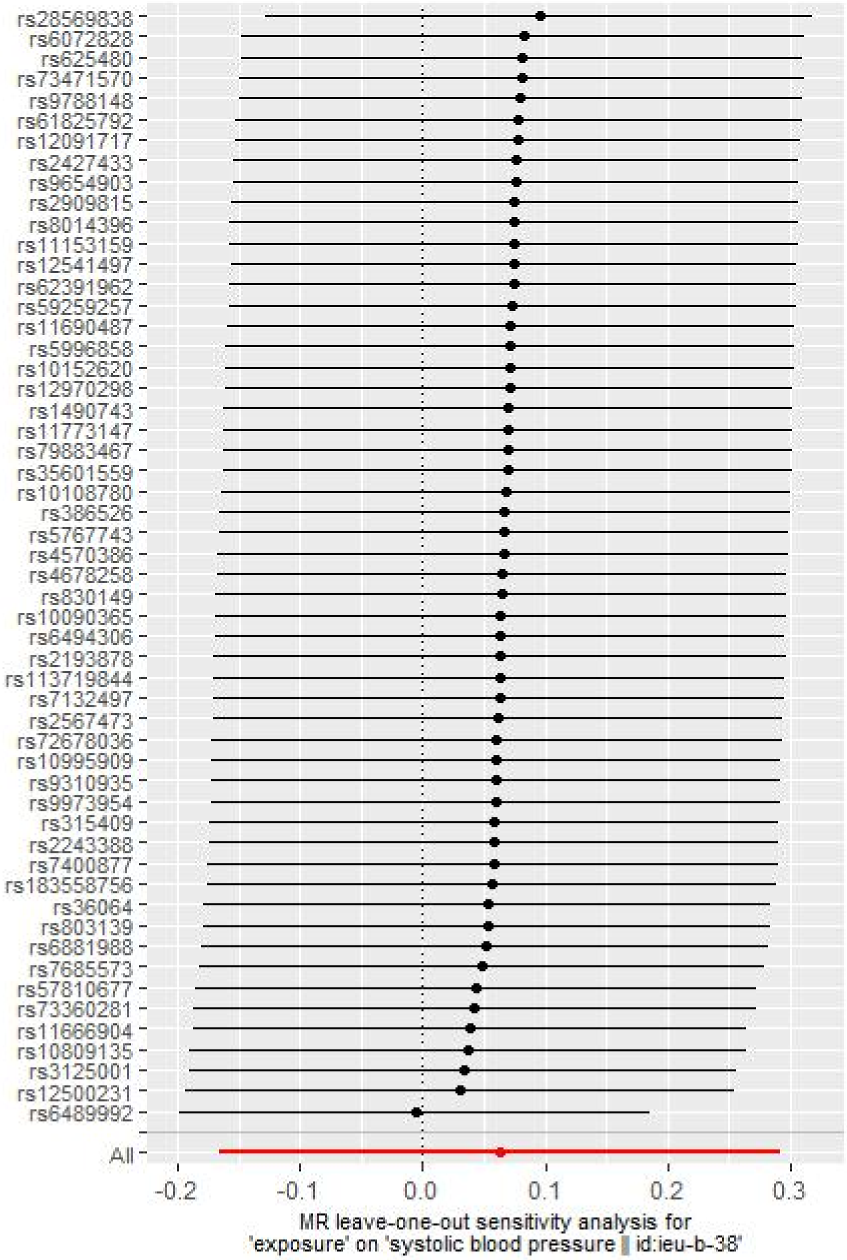

Figure 310: Funnel plots to visualize overall heterogeneity of Mendelian randomization (MR)

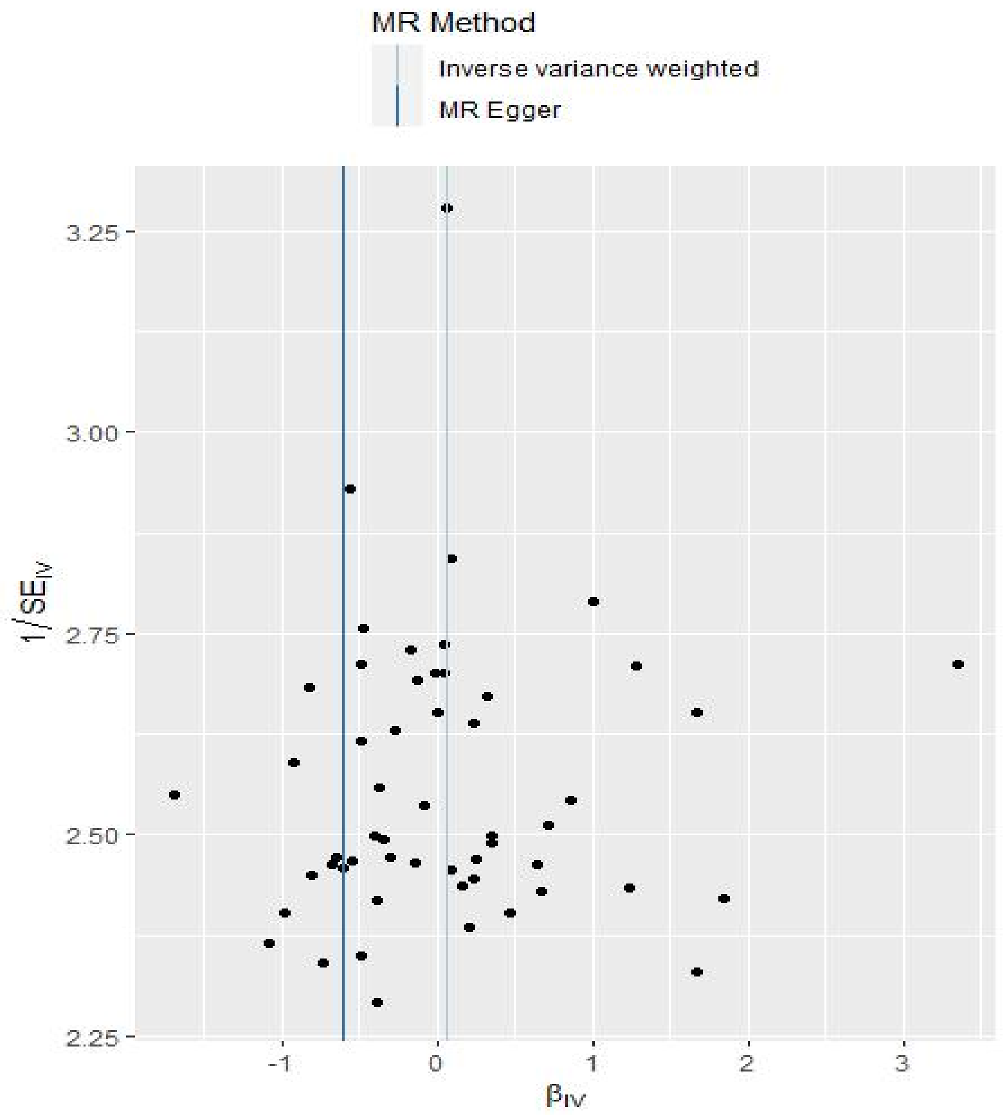

Figure 311: Leave-one-out plot to visualize causal effect of campylobacter on the risk of systolic blood pressure when leaving one SNP out.

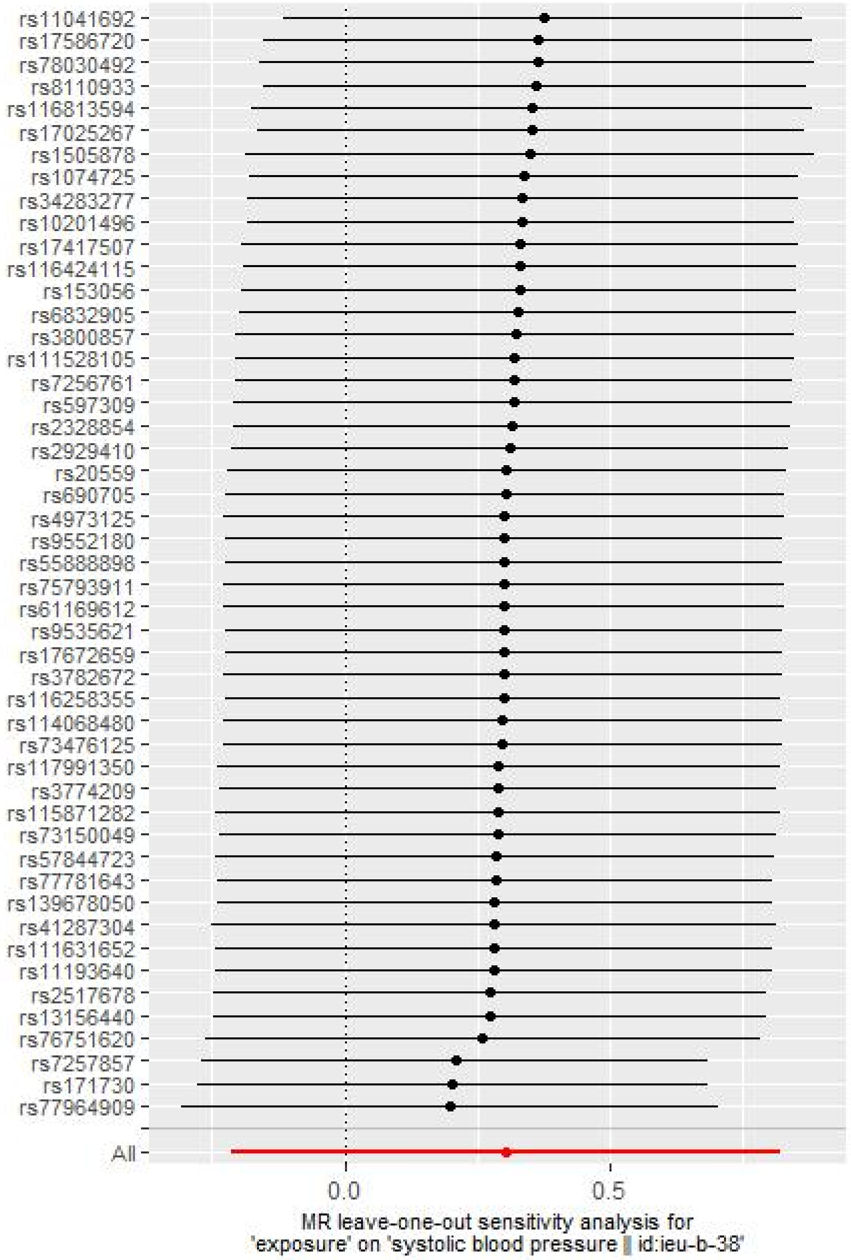

Figure 312: Funnel plots to visualize overall heterogeneity of Mendelian randomization (MR)

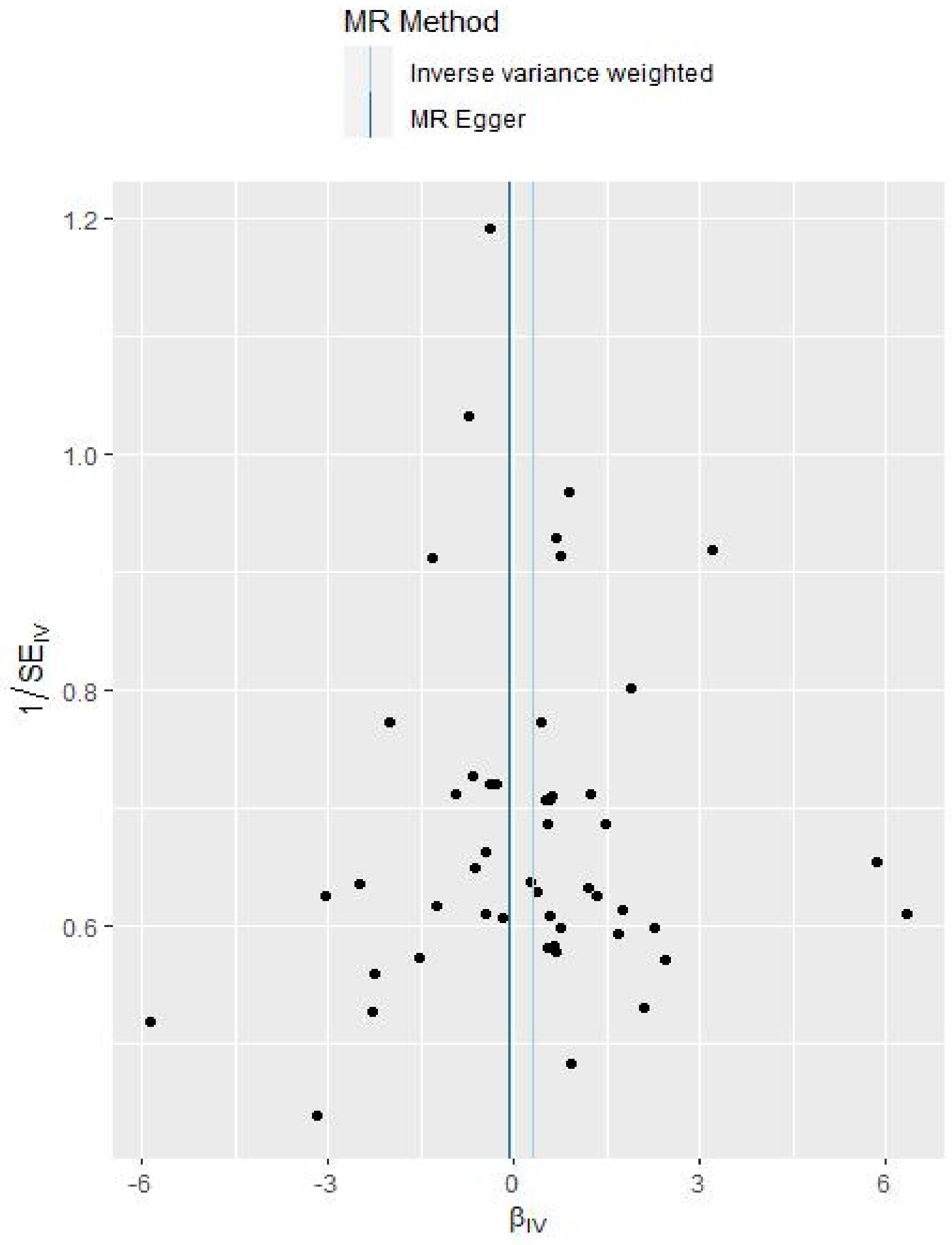

Figure 313: Leave-one-out plot to visualize causal effect of shigella on the risk of systolic blood pressure when leaving one SNP out.

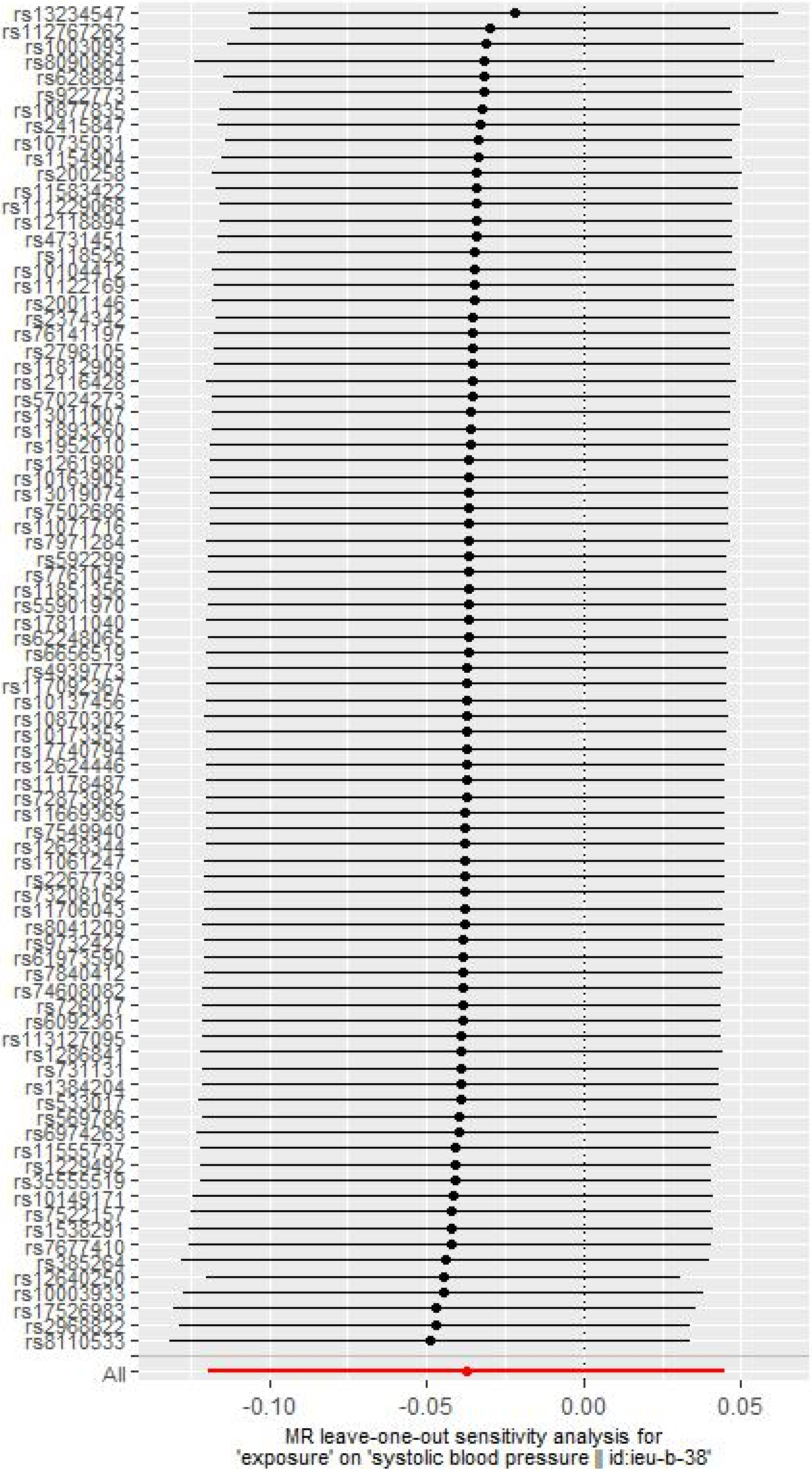

Figure 314: Funnel plots to visualize overall heterogeneity of Mendelian randomization (MR)

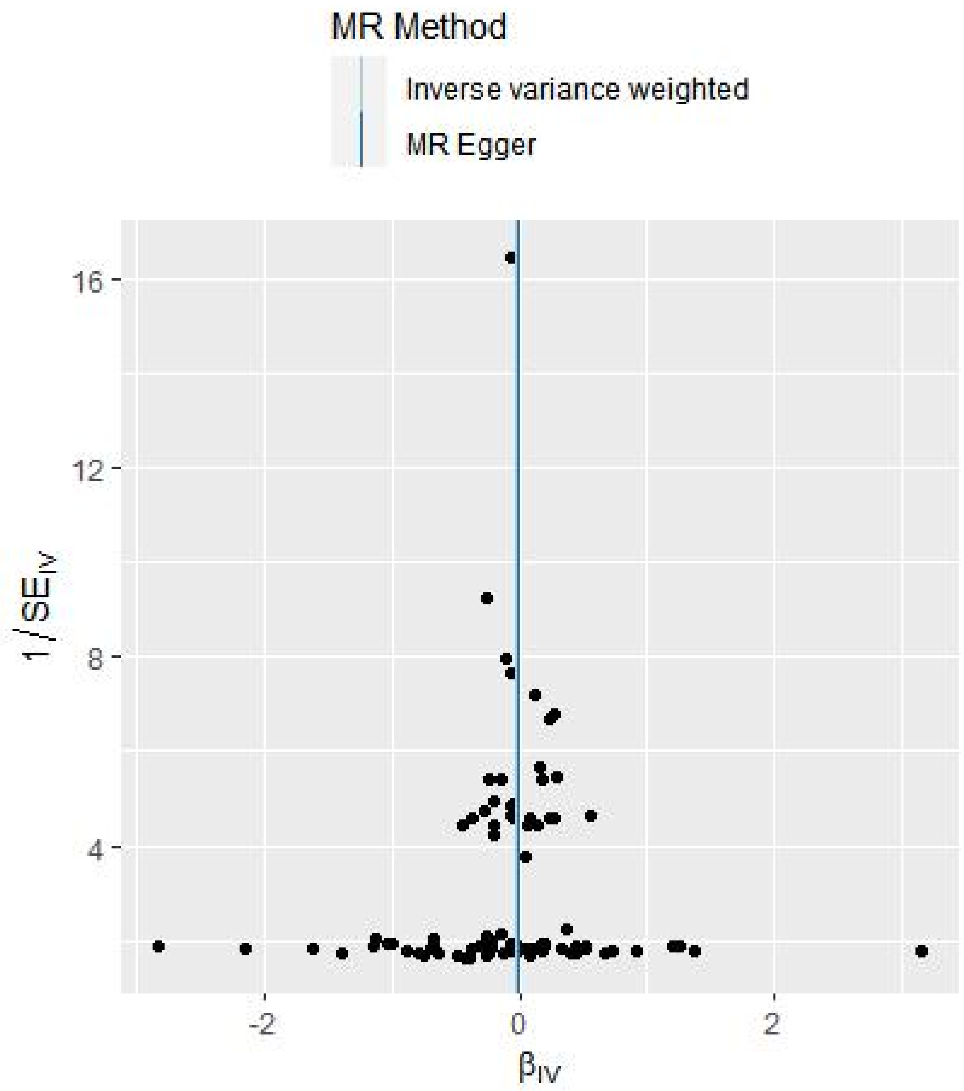

Figure 315: Leave-one-out plot to visualize causal effect of candida on the risk of diastolic blood pressure when leaving one SNP out.

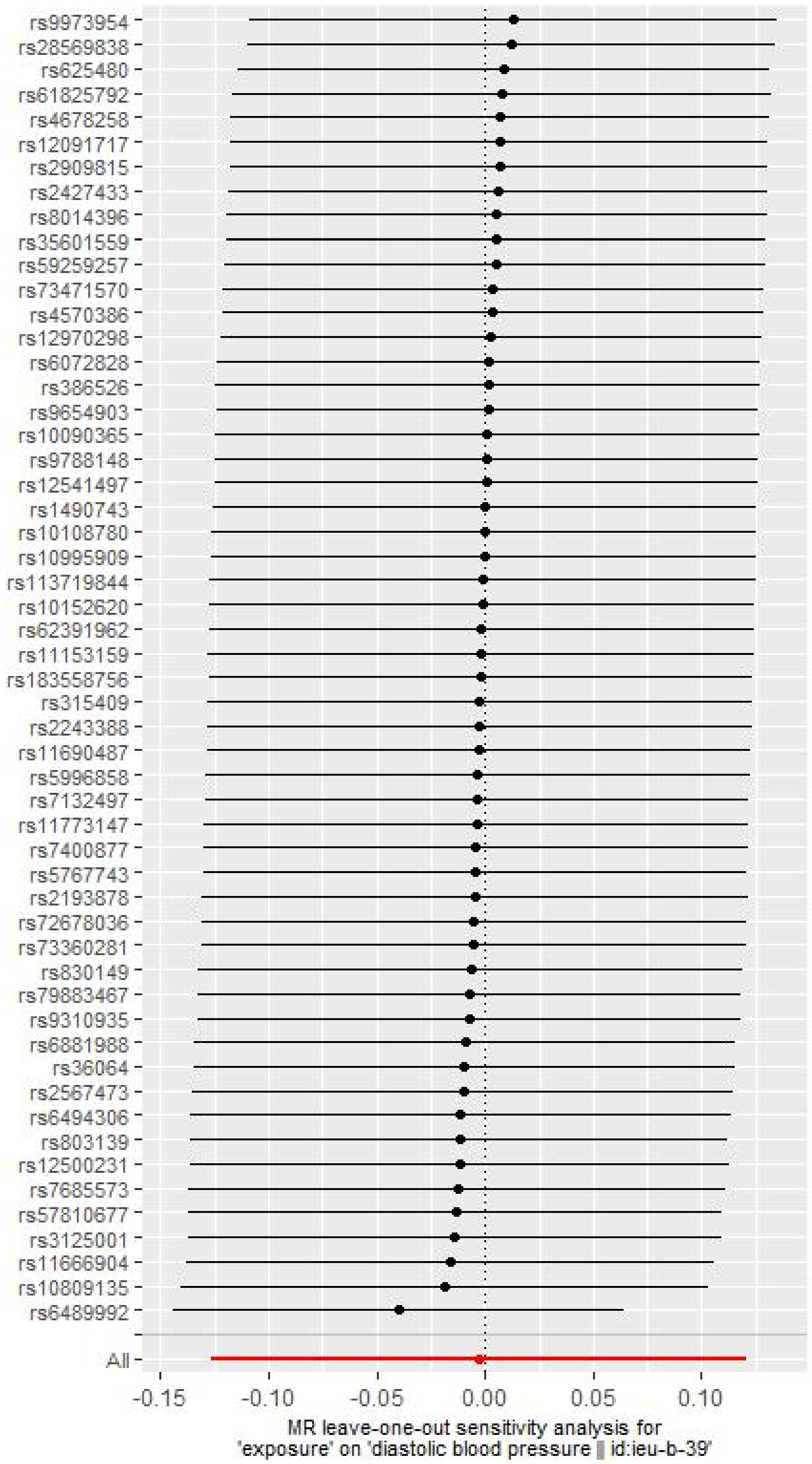

Figure 316: Funnel plots to visualize overall heterogeneity of Mendelian randomization (MR)

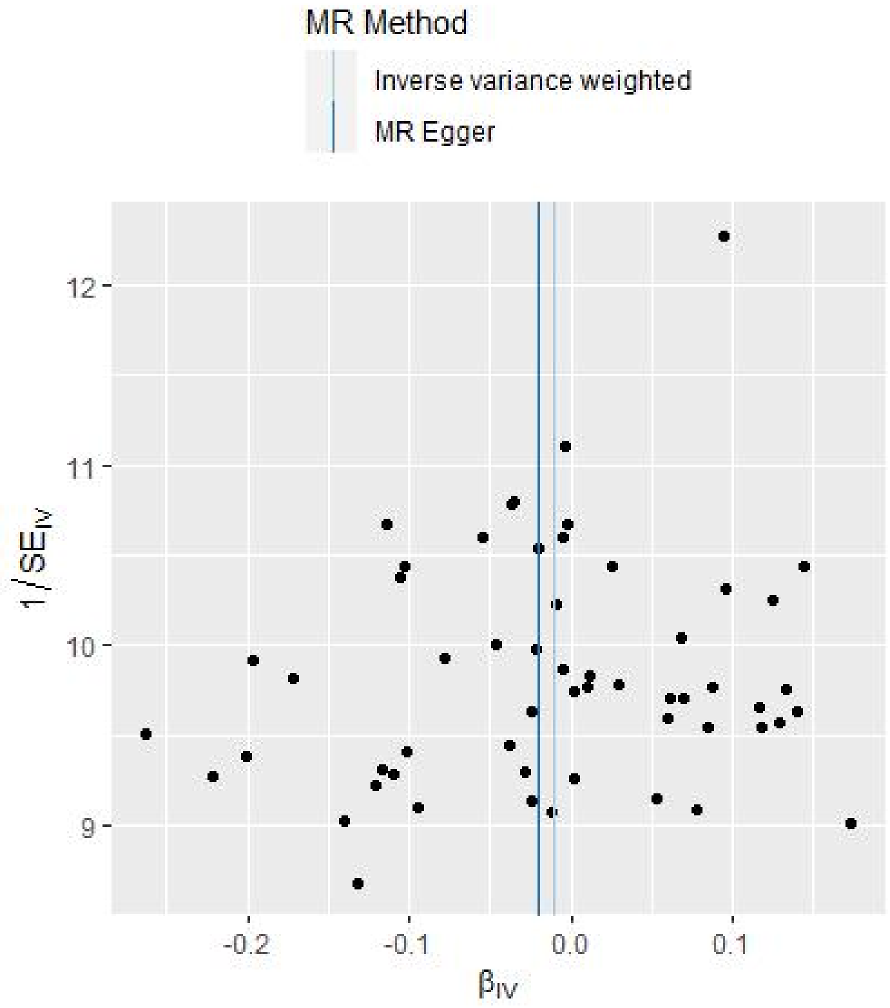

Figure 317: Leave-one-out plot to visualize causal effect of campylobacter on the risk of diastolic blood pressure when leaving one SNP out.

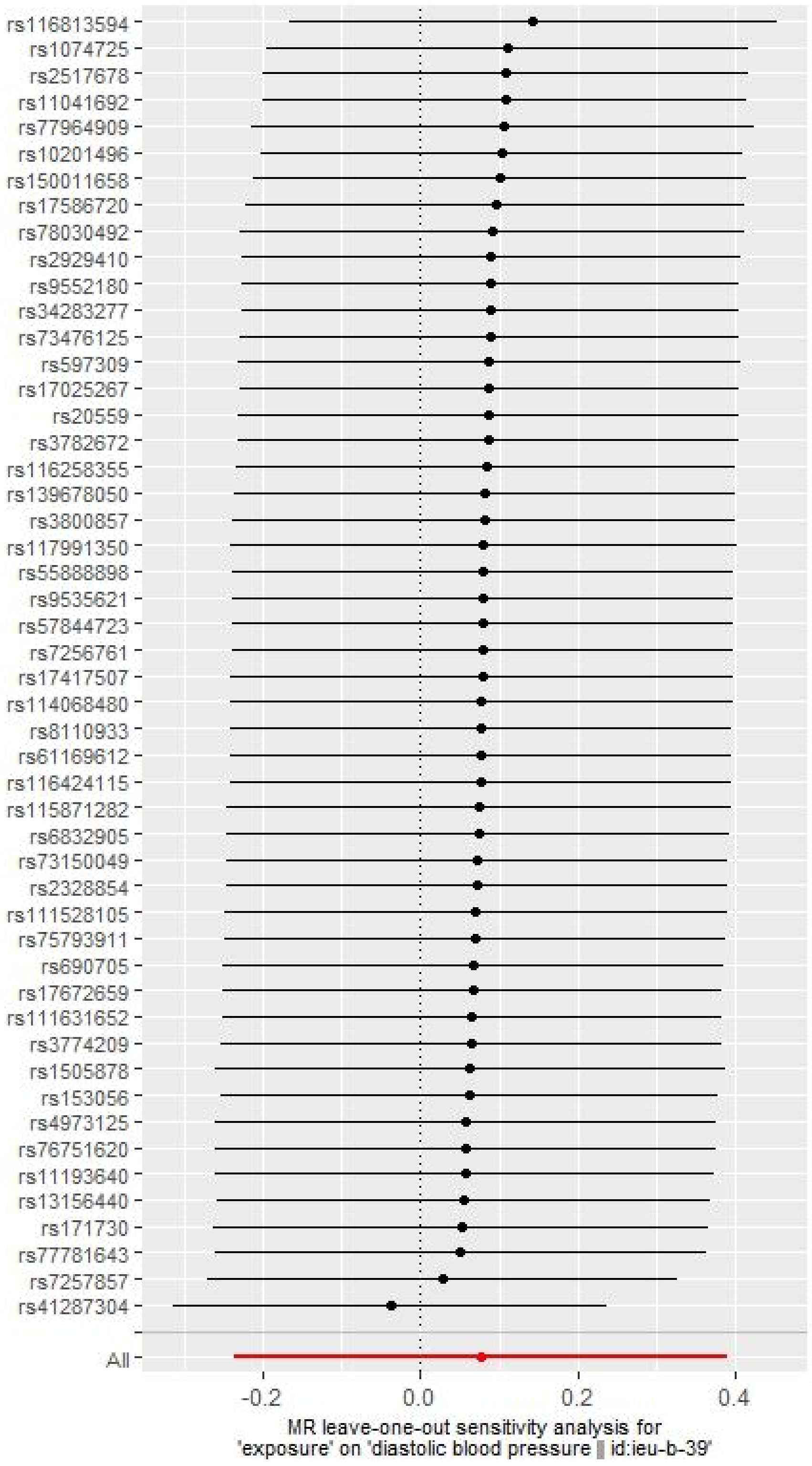

Figure 318: Funnel plots to visualize overall heterogeneity of Mendelian randomization (MR)

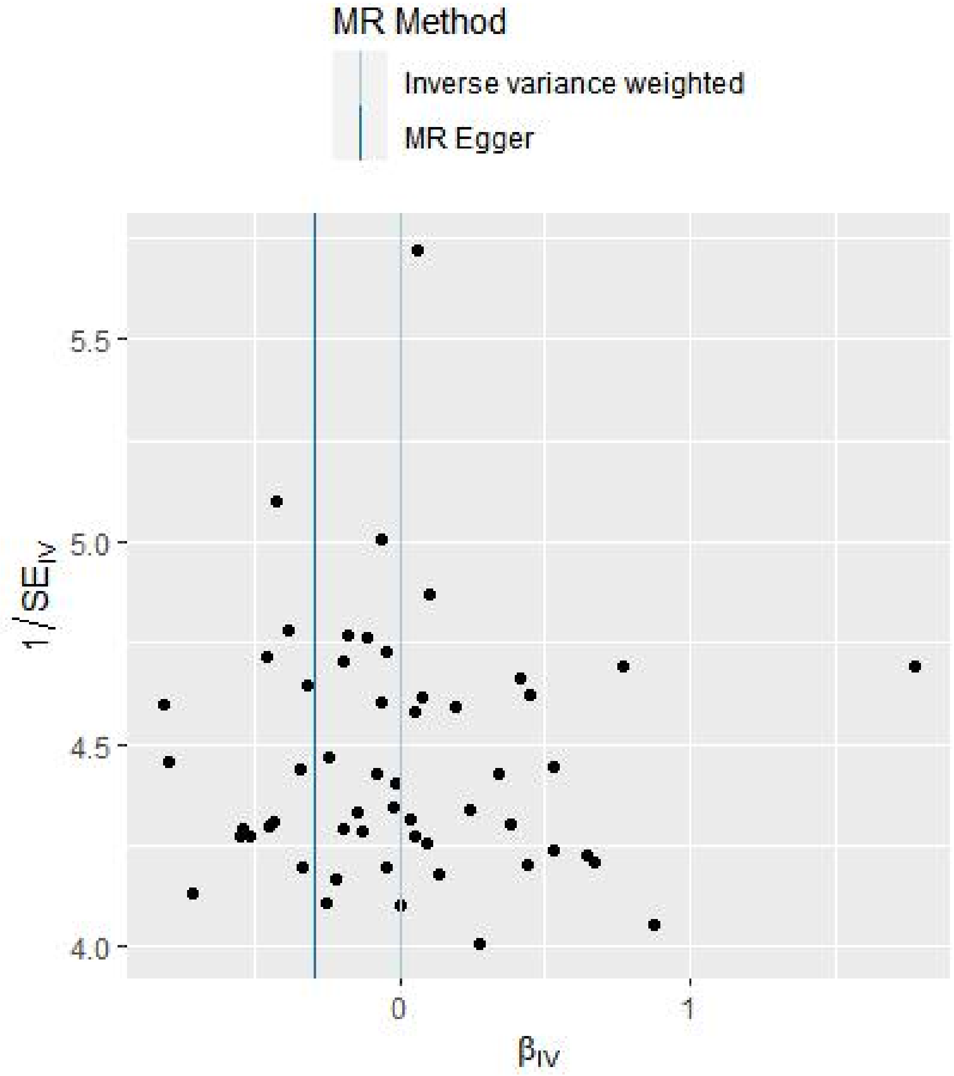

Figure 319: Leave-one-out plot to visualize causal effect of shigella on the risk of diastolic blood pressure when leaving one SNP out.

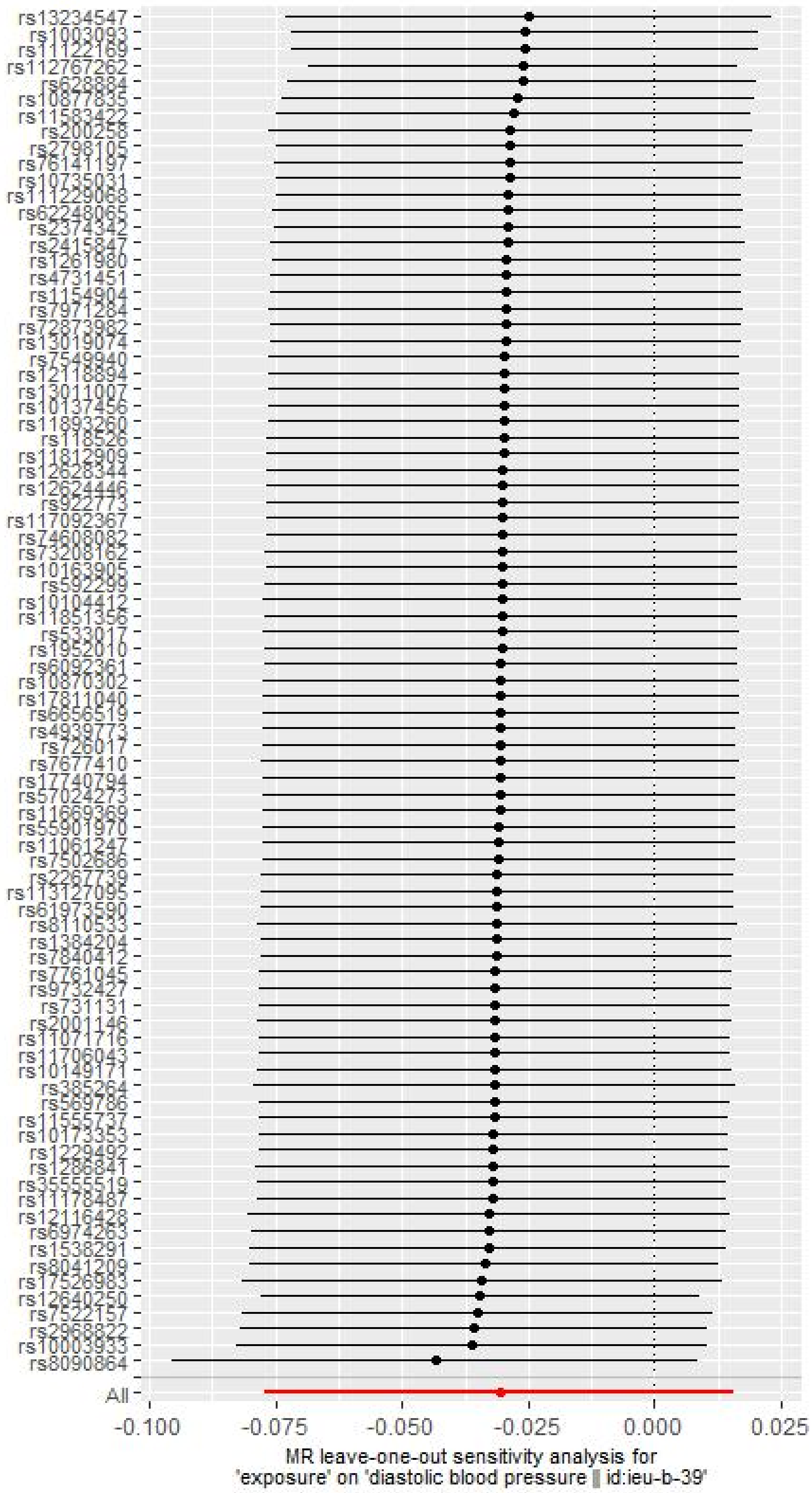

Figure 320: Funnel plots to visualize overall heterogeneity of Mendelian randomization (MR)

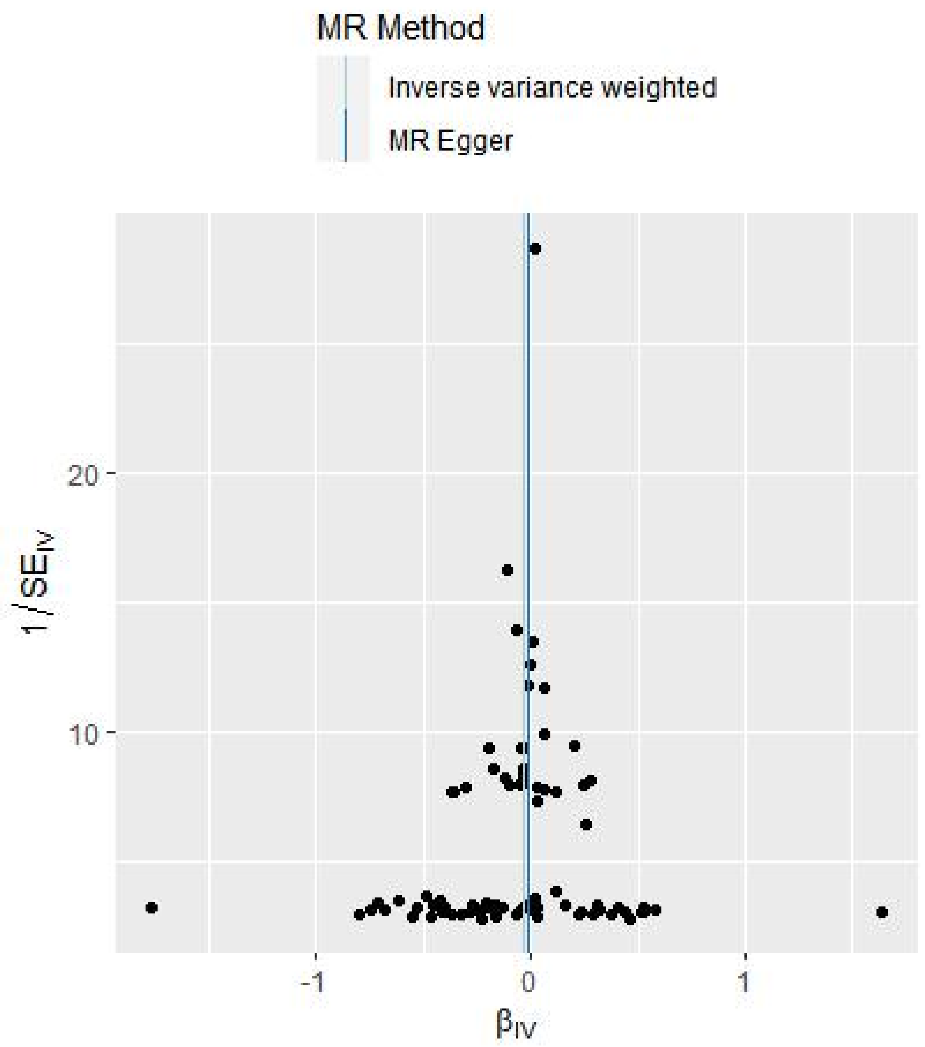

Figure 321: Leave-one-out plot to visualize causal effect of candida on the risk of diabetes when leaving one SNP out.

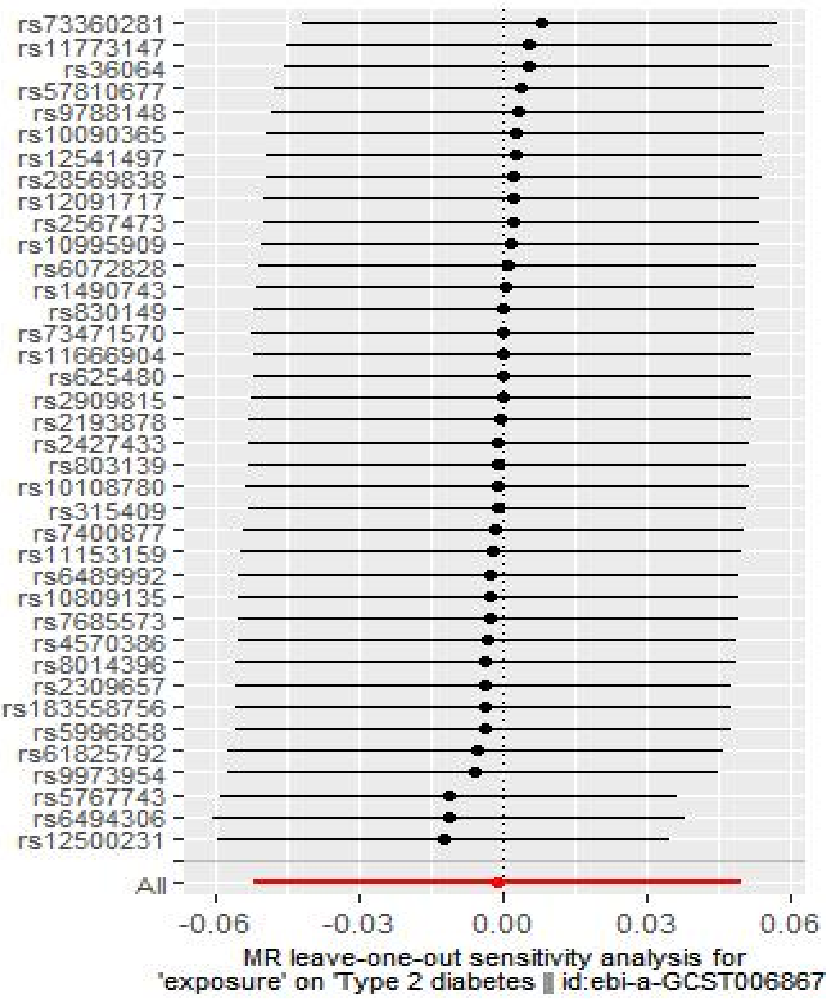

Figure 322: Funnel plots to visualize overall heterogeneity of Mendelian randomization (MR)

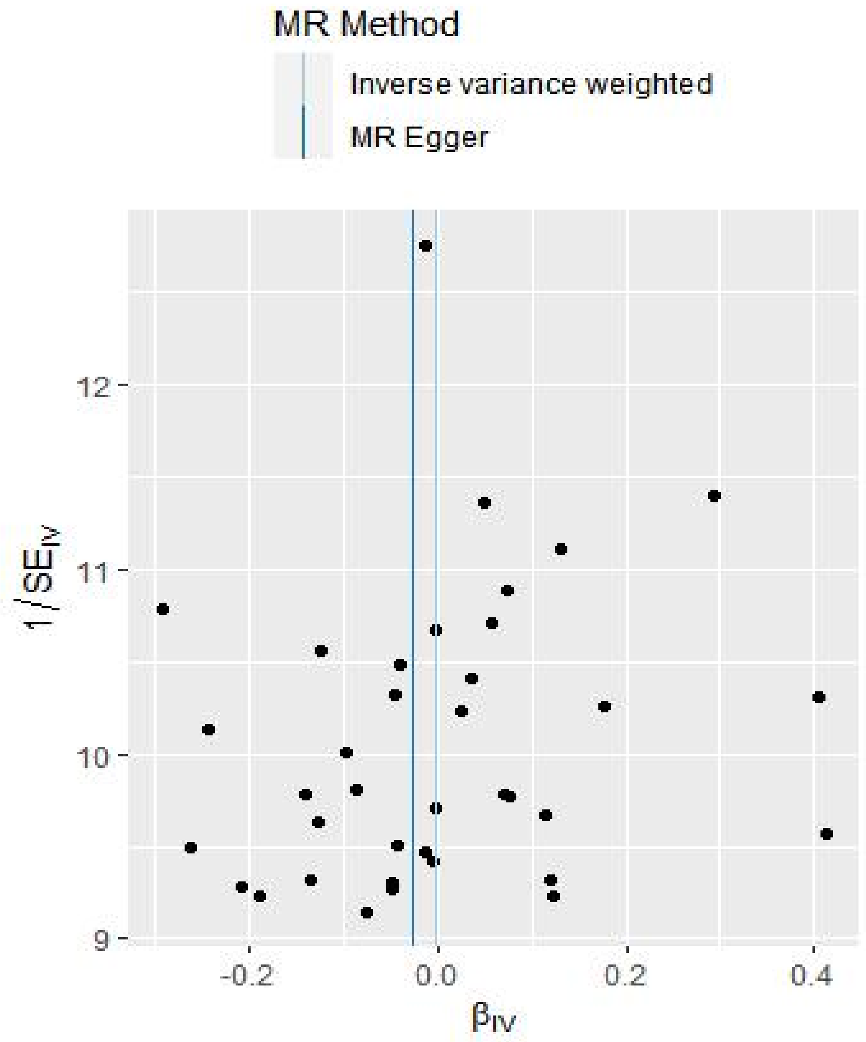

Figure 323: Leave-one-out plot to visualize causal effect of campylobacter on the risk of diabetes when leaving one SNP out.

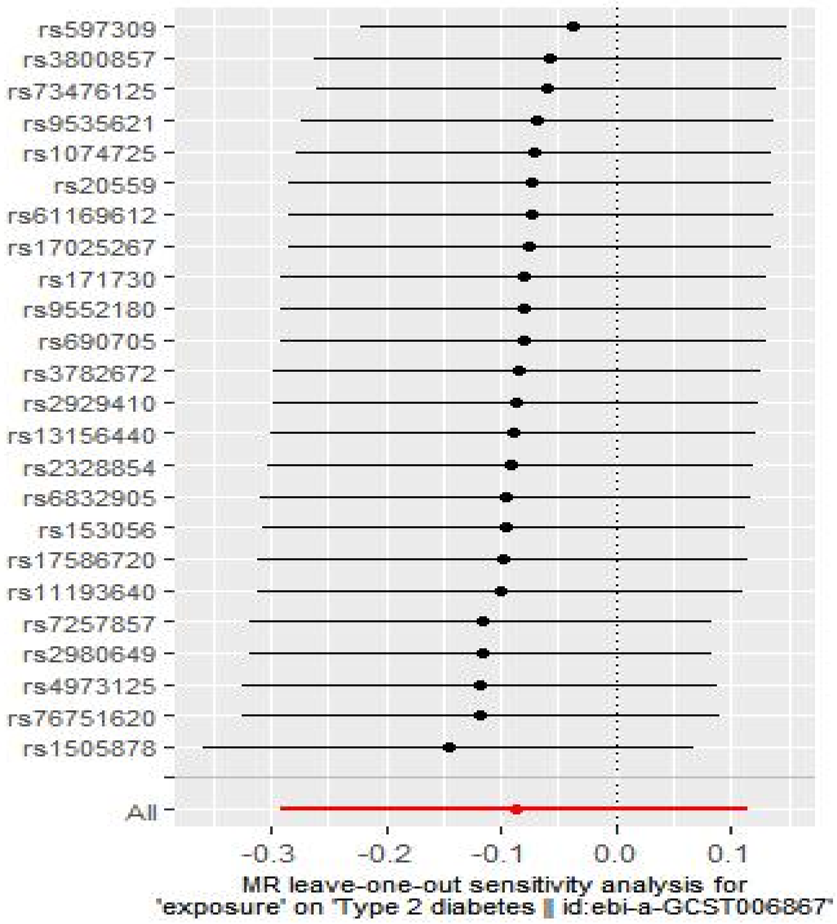

Figure 324: Funnel plots to visualize overall heterogeneity of Mendelian randomization (MR)

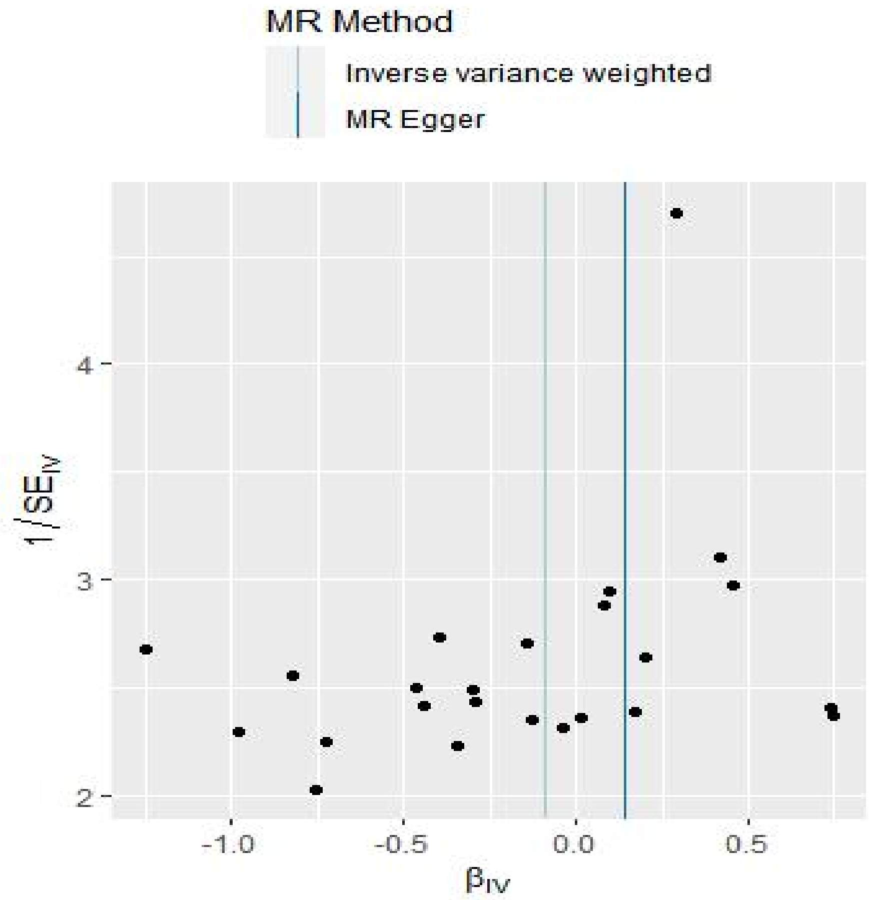

Figure 325: Leave-one-out plot to visualize causal effect of shigella on the risk of diabetes when leaving one SNP out.

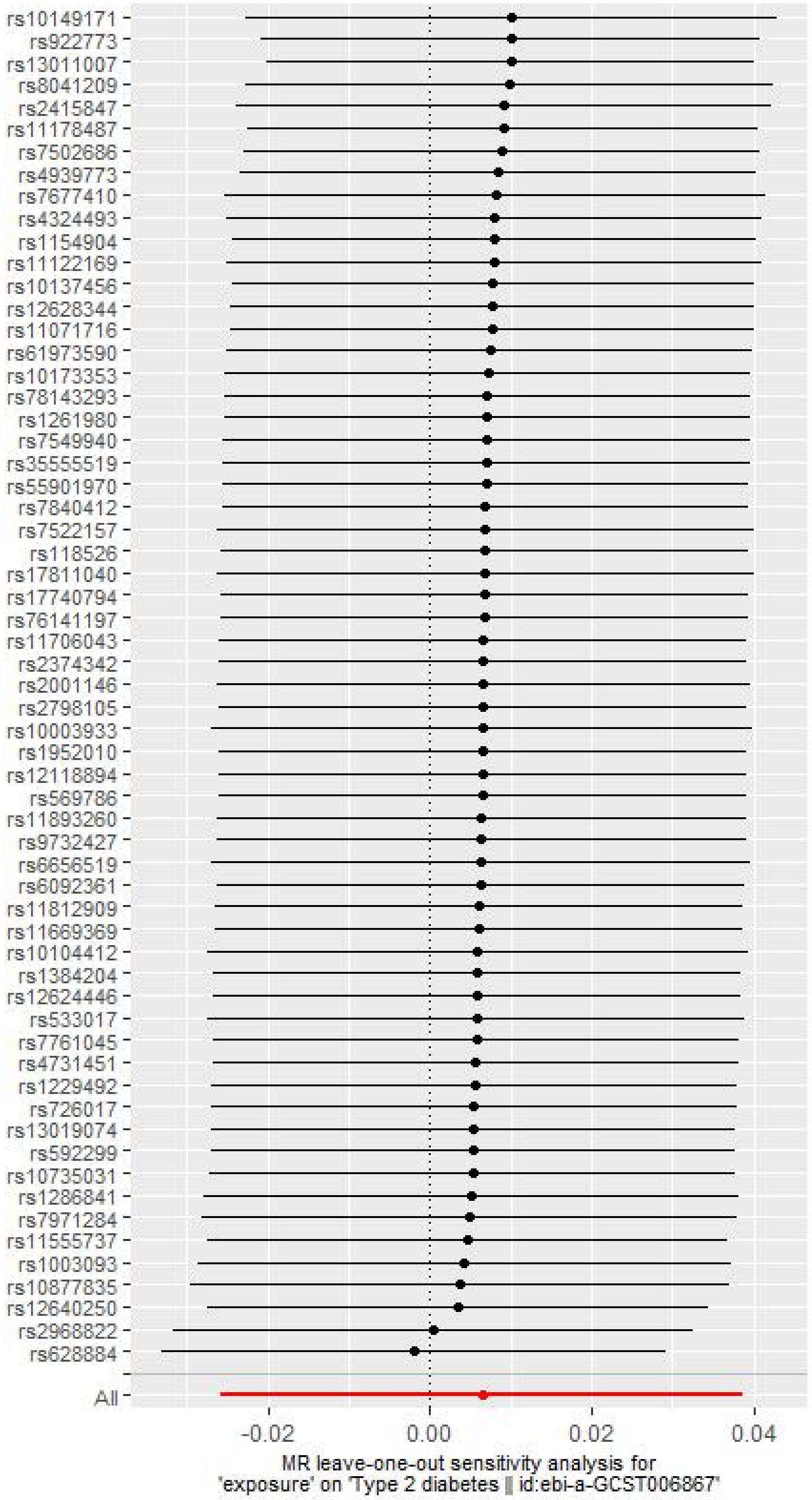

Figure 326: Funnel plots to visualize overall heterogeneity of Mendelian randomization (MR)

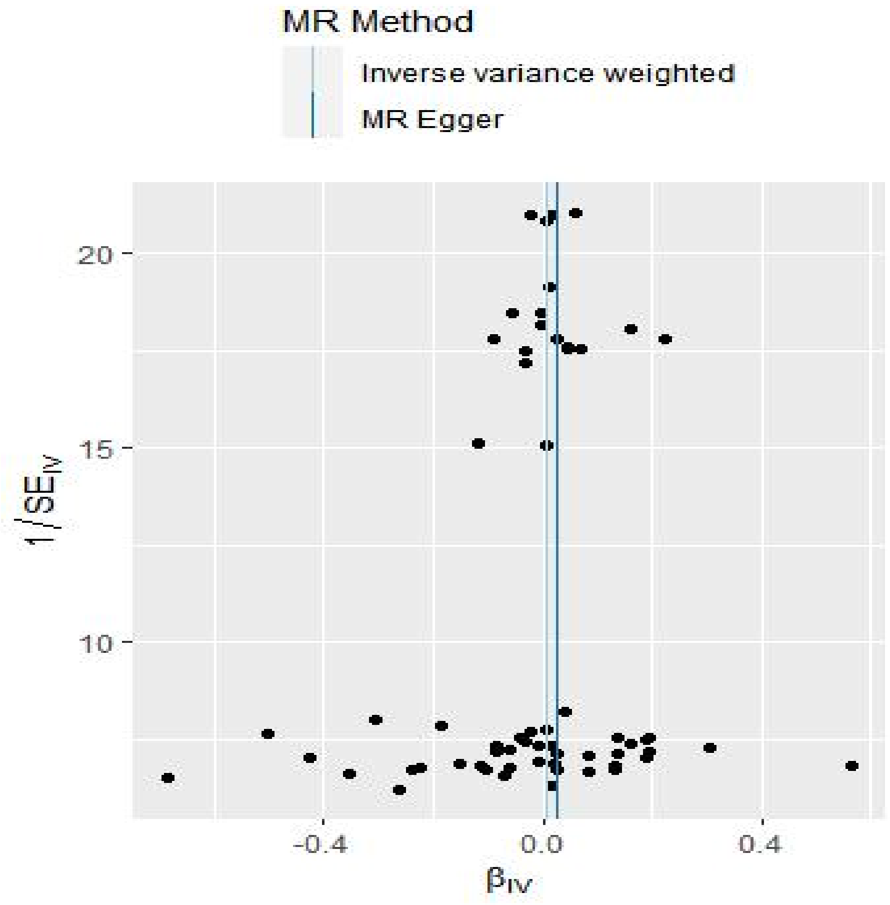

Figure 327: Leave-one-out plot to visualize causal effect of campylobacter on the risk of heart valve disease when leaving one SNP out.

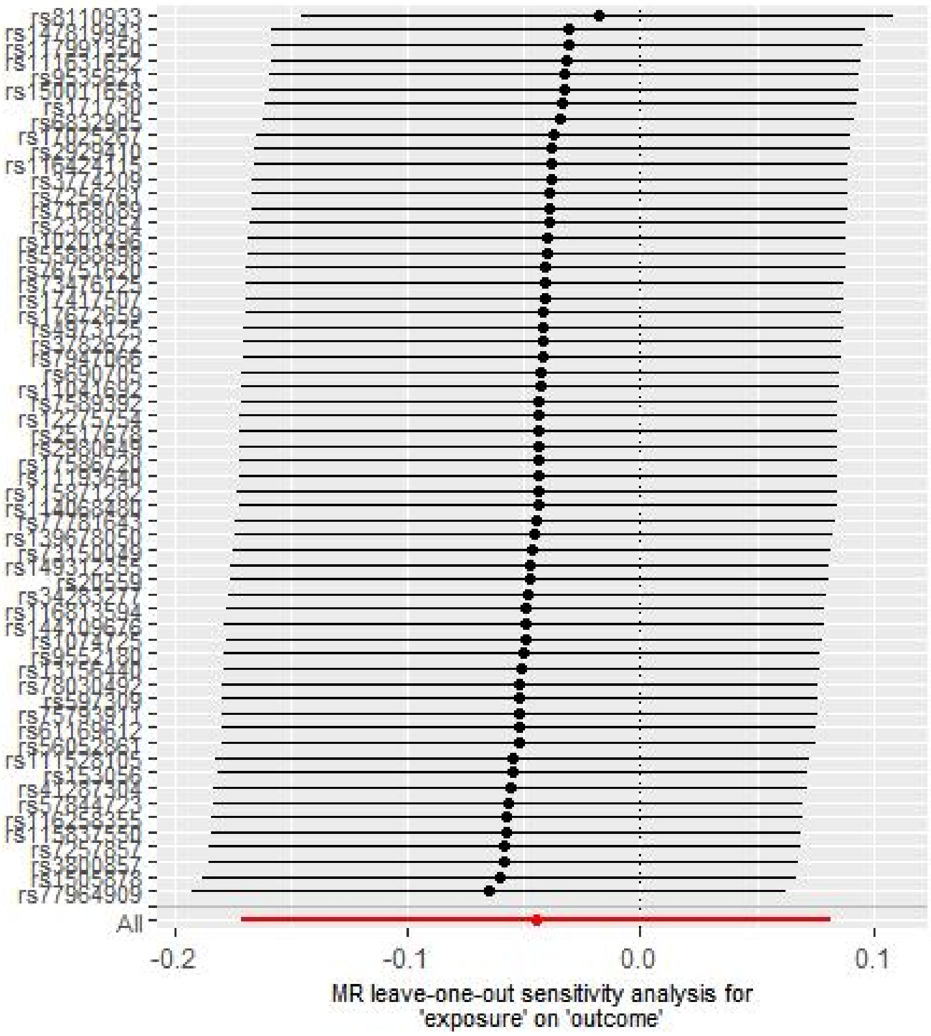

Figure 328: Funnel plots to visualize overall heterogeneity of Mendelian randomization (MR)

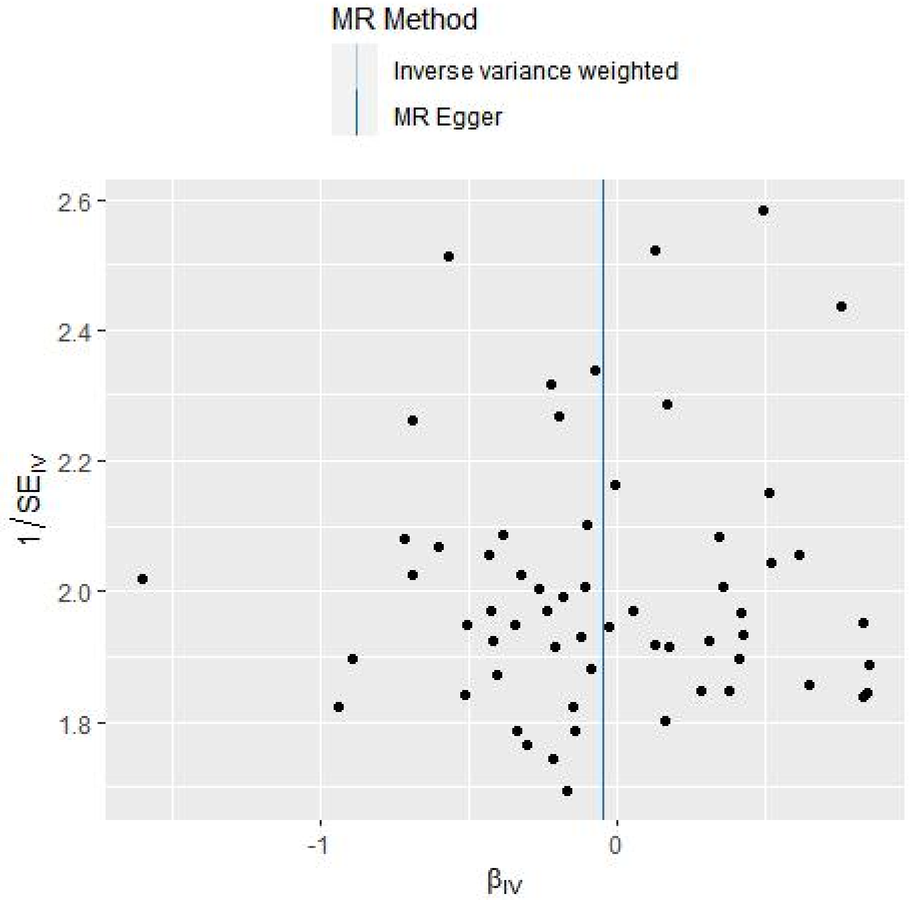

Figure 329: Leave-one-out plot to visualize causal effect of shigella on the risk of heart valve disease when leaving one SNP out.

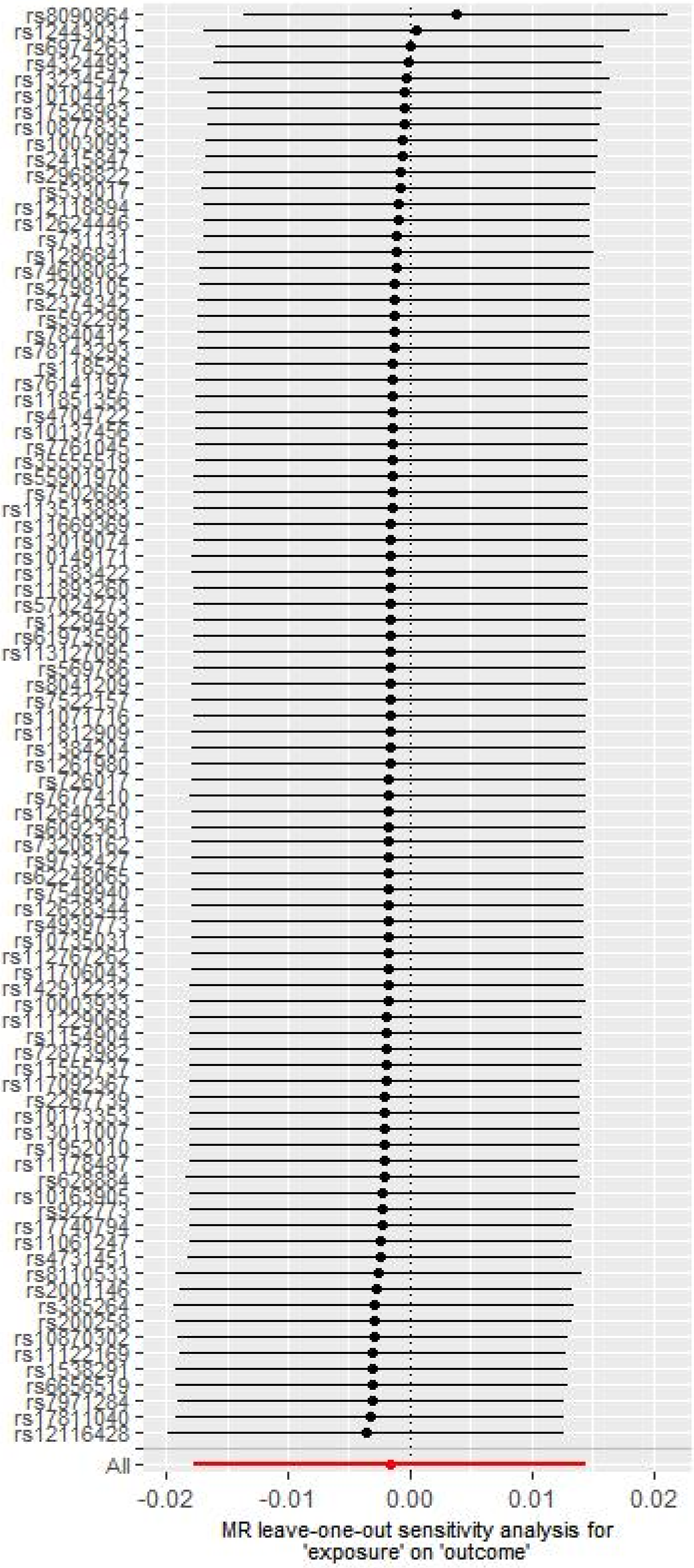

Figure 330: Funnel plots to visualize overall heterogeneity of Mendelian randomization (MR)

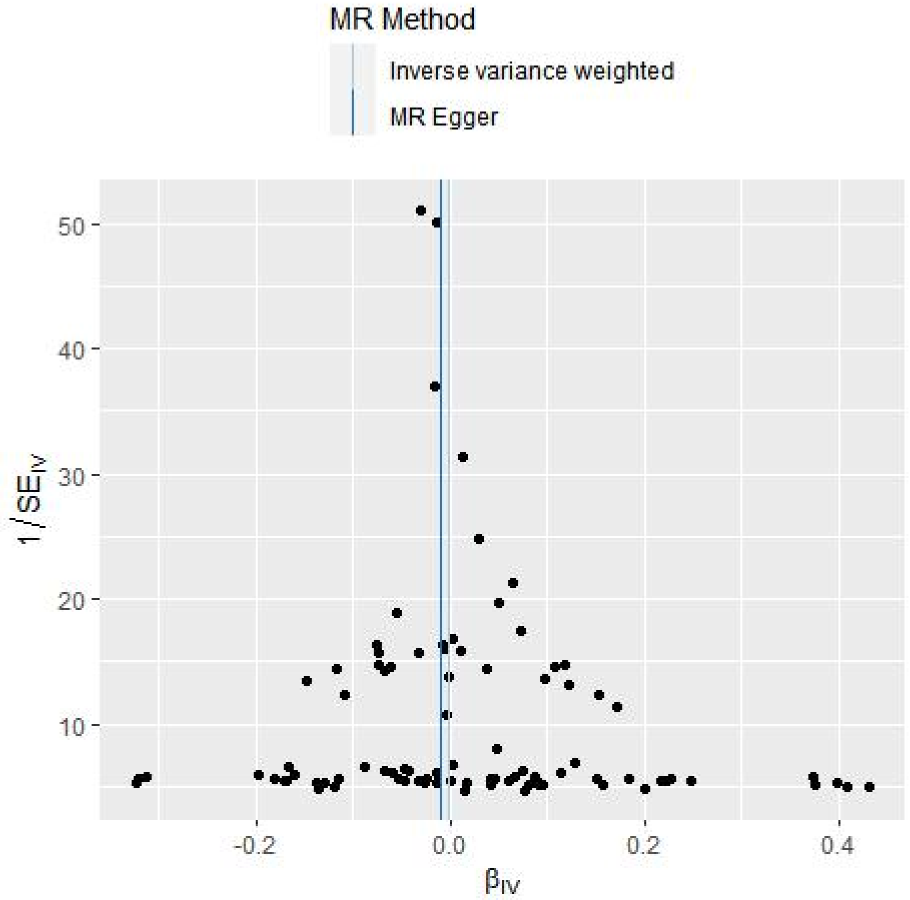

Figure 331: Leave-one-out plot to visualize causal effect of candida on the risk of heart valve disease when leaving one SNP out.

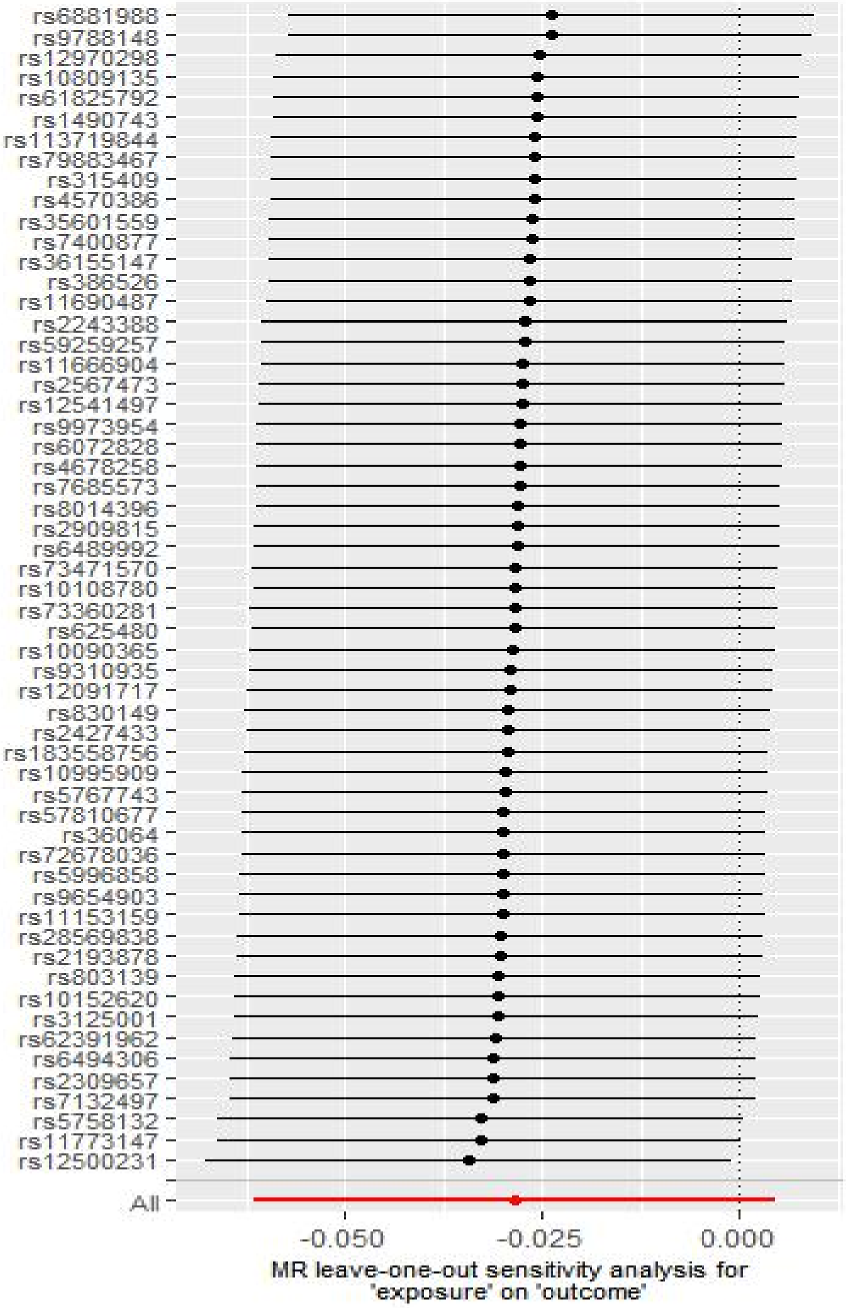

Figure 332: Funnel plots to visualize overall heterogeneity of Mendelian randomization (MR)

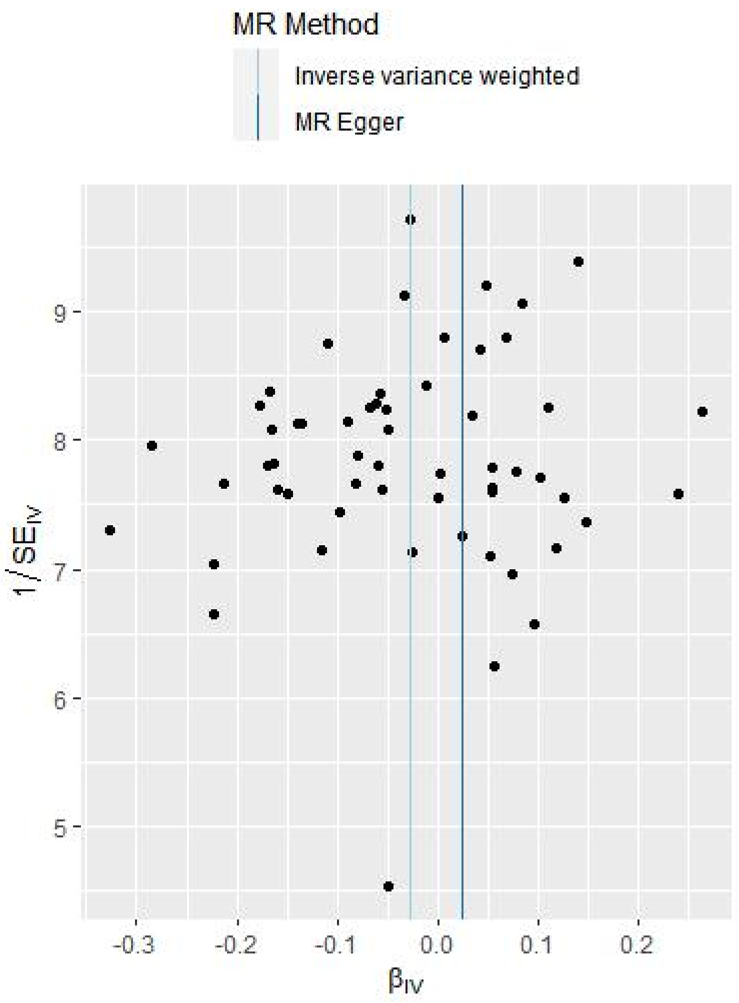

Figure 333: Leave-one-out plot to visualize causal effect of candida on the risk of myocardial infarction when leaving one SNP out.

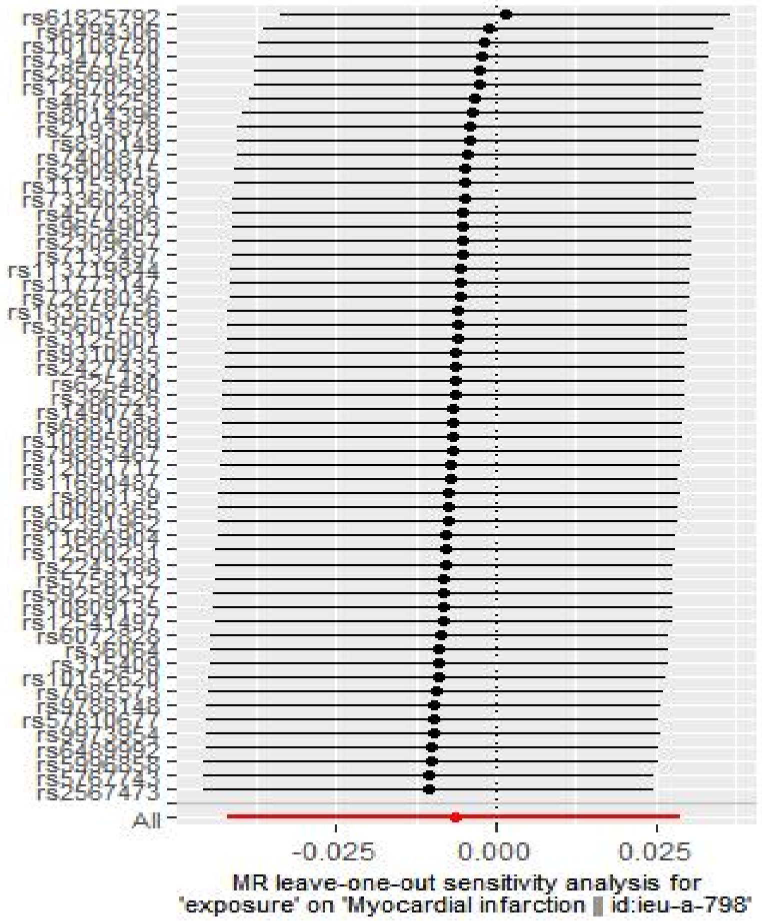

Figure 334: Funnel plots to visualize overall heterogeneity of Mendelian randomization (MR)

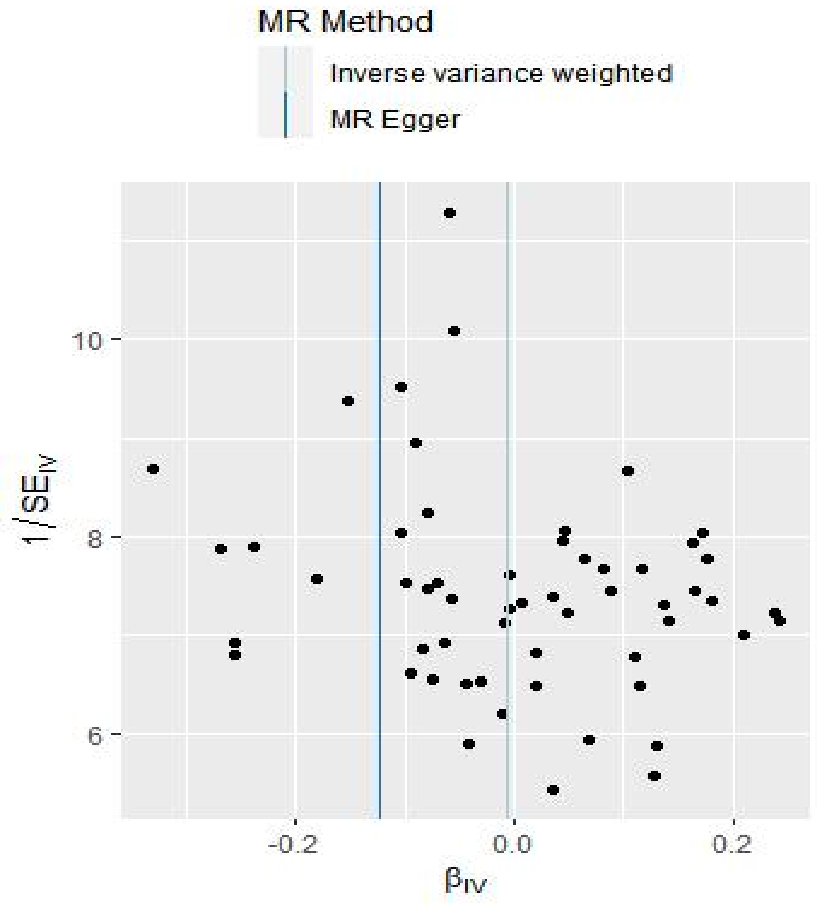

Figure 335: Leave-one-out plot to visualize causal effect of campylobacter on the risk of myocardial infarction when leaving one SNP out.

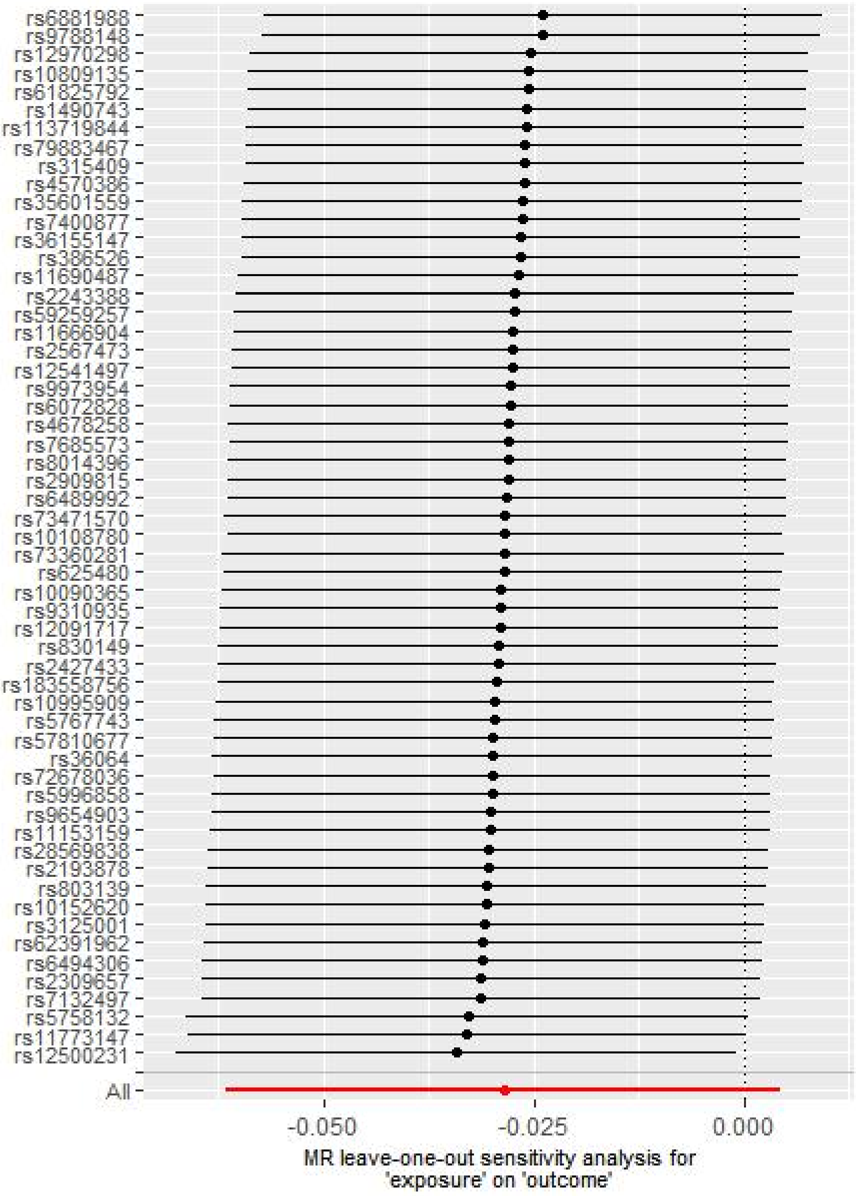

Figure 336: Funnel plots to visualize overall heterogeneity of Mendelian randomization (MR)

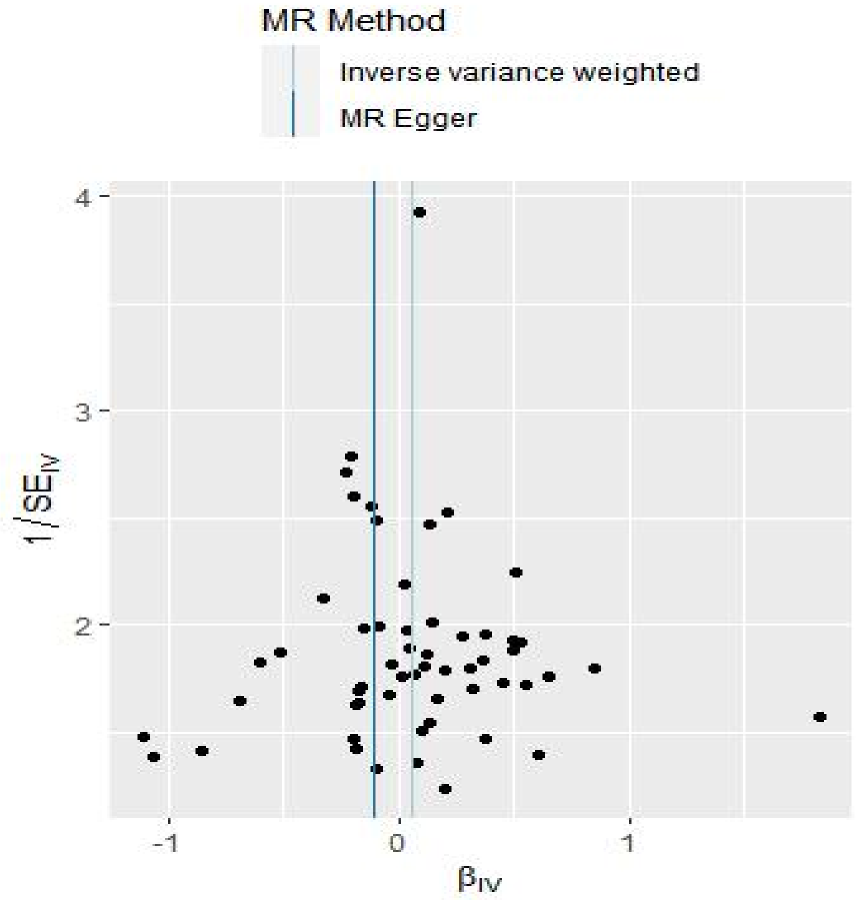

Figure 337: Leave-one-out plot to visualize causal effect of shigella on the risk of myocardial infarction when leaving one SNP out.

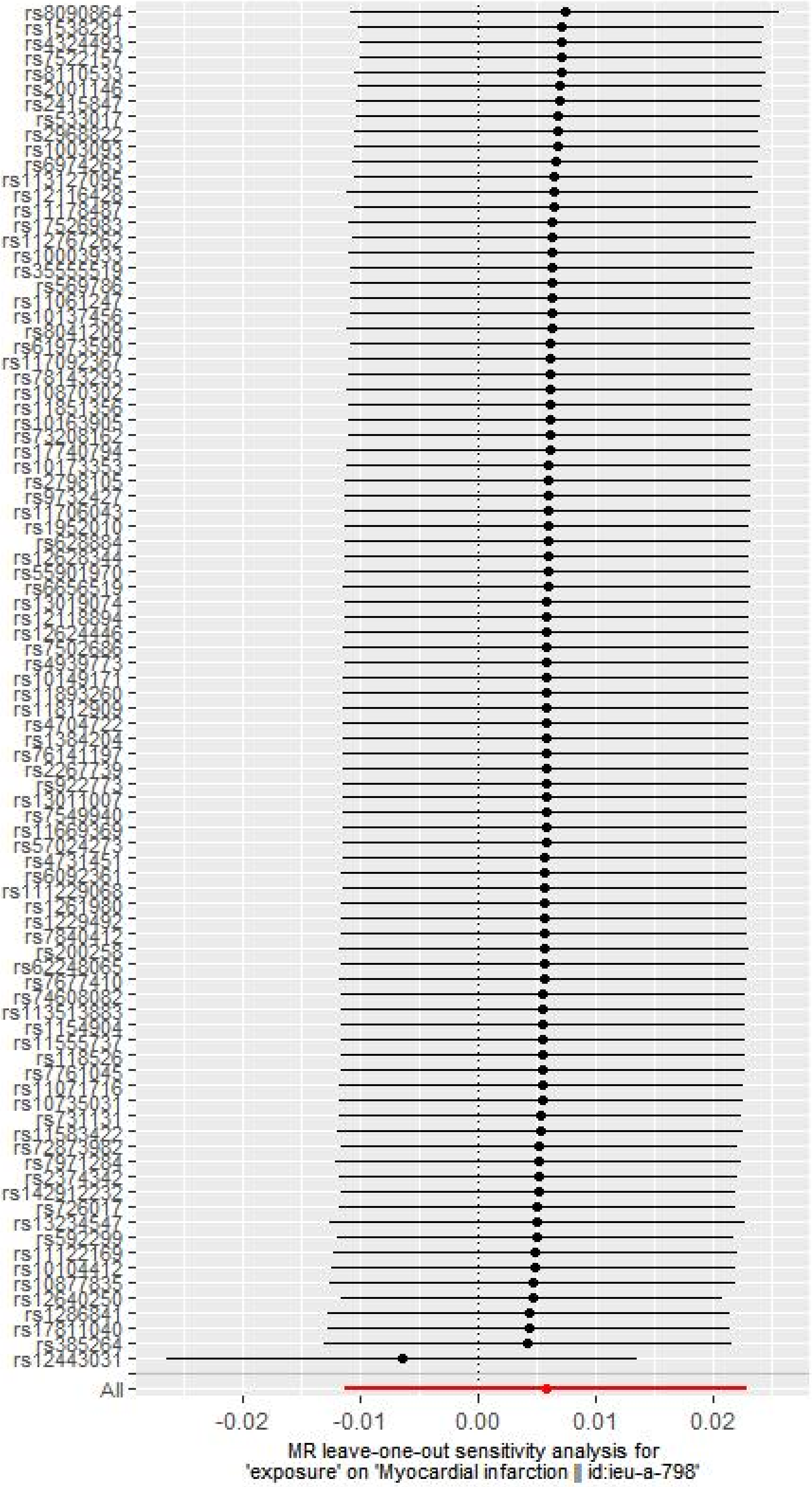

Figure 338: Funnel plots to visualize overall heterogeneity of Mendelian randomization (MR)

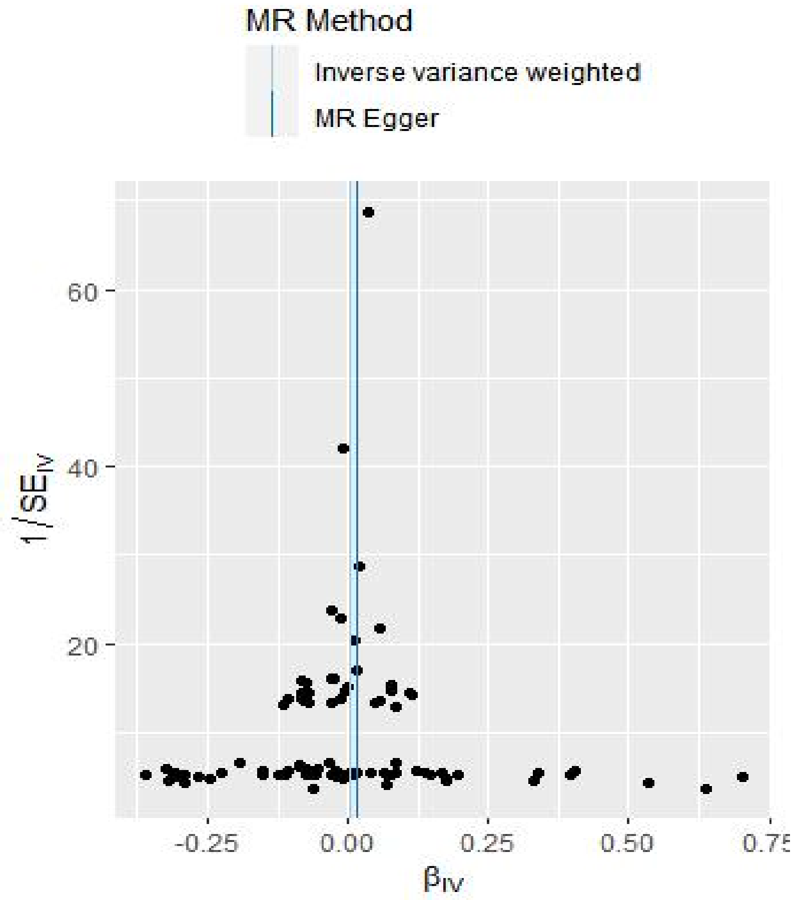

Figure 339: Leave-one-out plot to visualize causal effect of candida on the risk of myocarditis when leaving one SNP out.

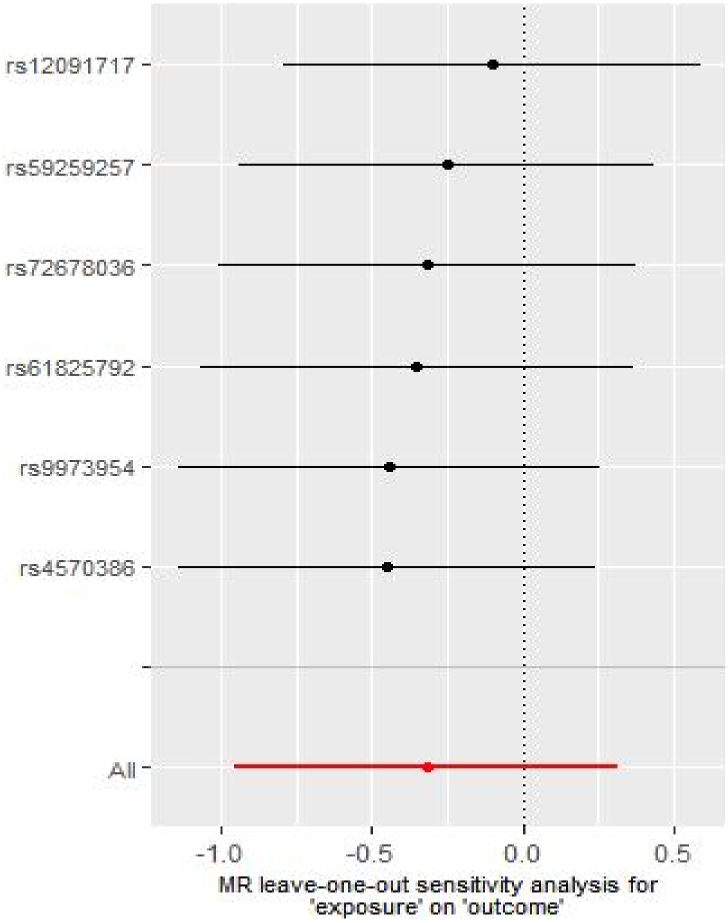

Figure 340: Funnel plots to visualize overall heterogeneity of Mendelian randomization (MR)

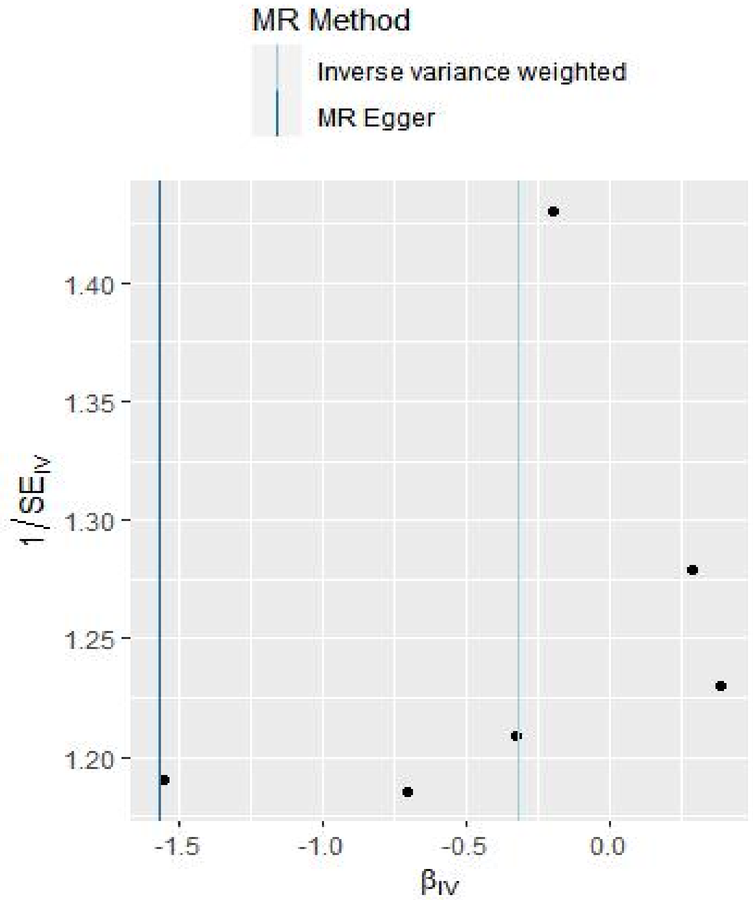

Figure 341: Leave-one-out plot to visualize causal effect of shigella on the risk of myocarditis when leaving one SNP out.

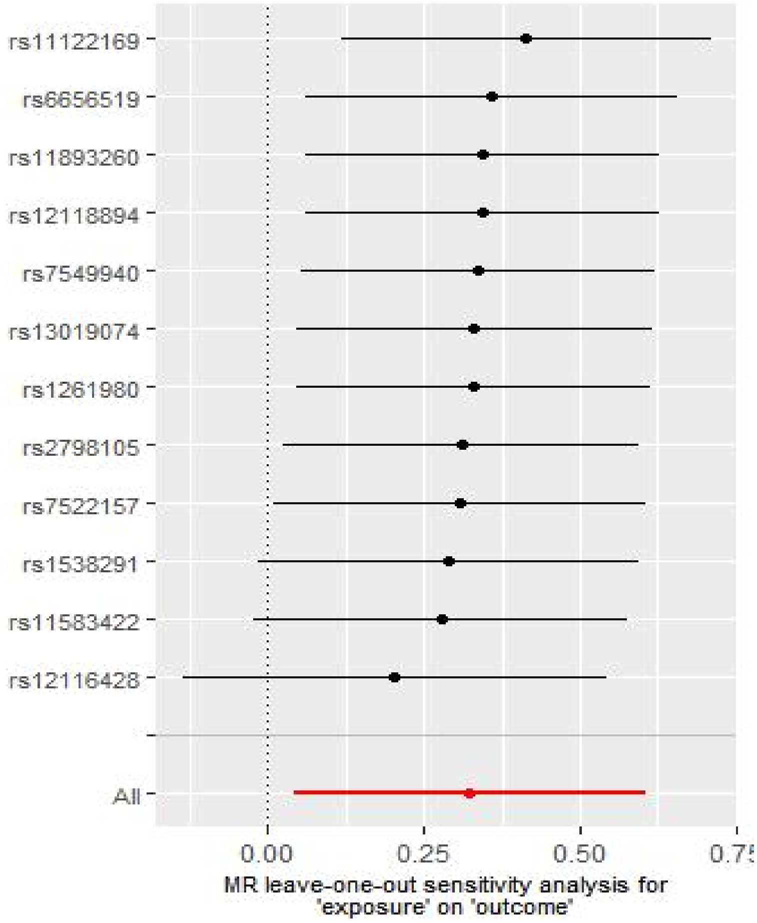

Figure 342: Funnel plots to visualize overall heterogeneity of Mendelian randomization (MR)

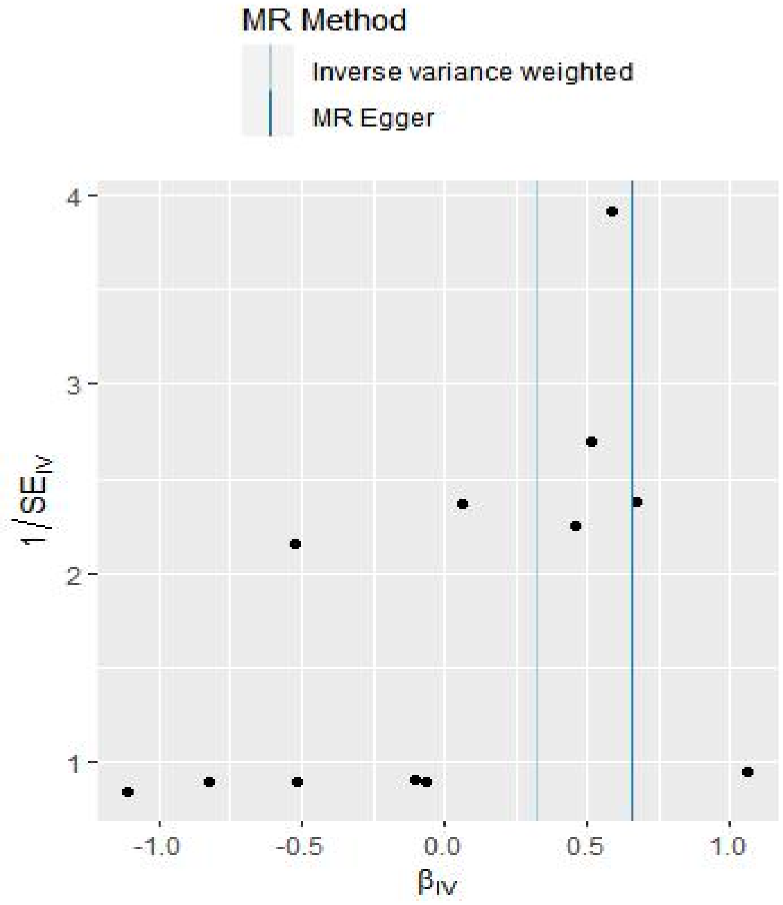

Figure 343: Leave-one-out plot to visualize causal effect of candida on the risk of heart failure when leaving one SNP out.

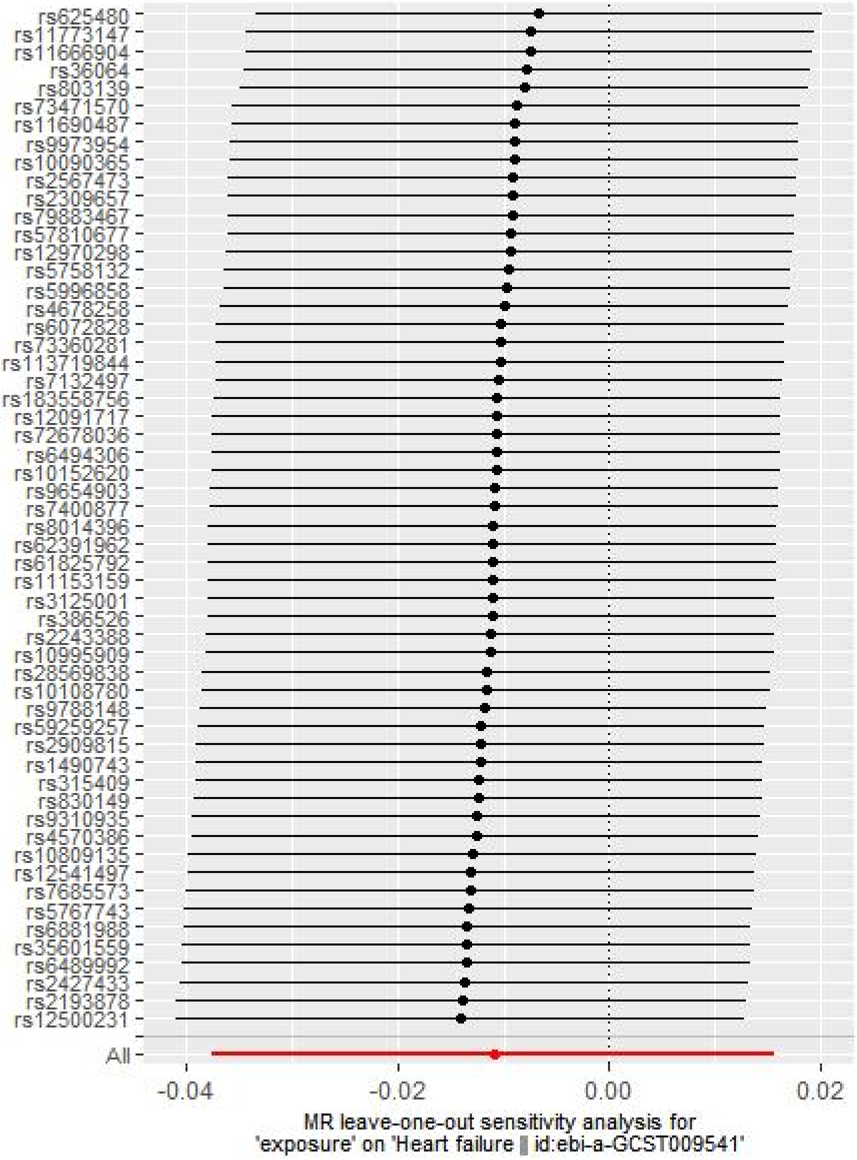

Figure 344: Funnel plots to visualize overall heterogeneity of Mendelian randomization (MR)

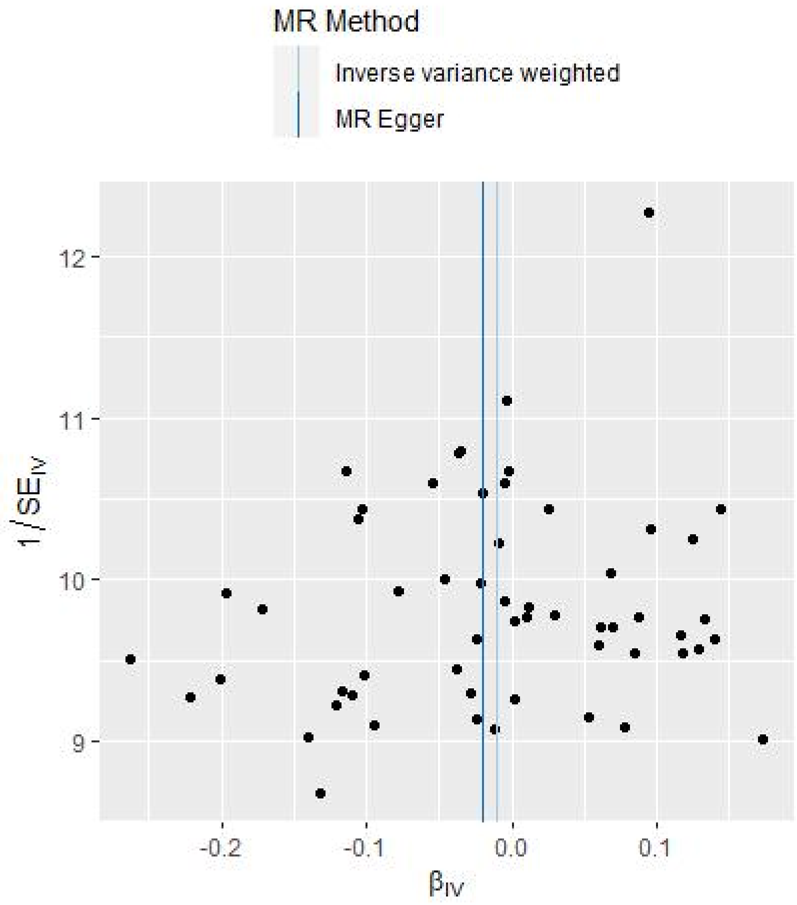

Figure 345: Leave-one-out plot to visualize causal effect of campylobacter on the risk of heart failure when leaving one SNP out.

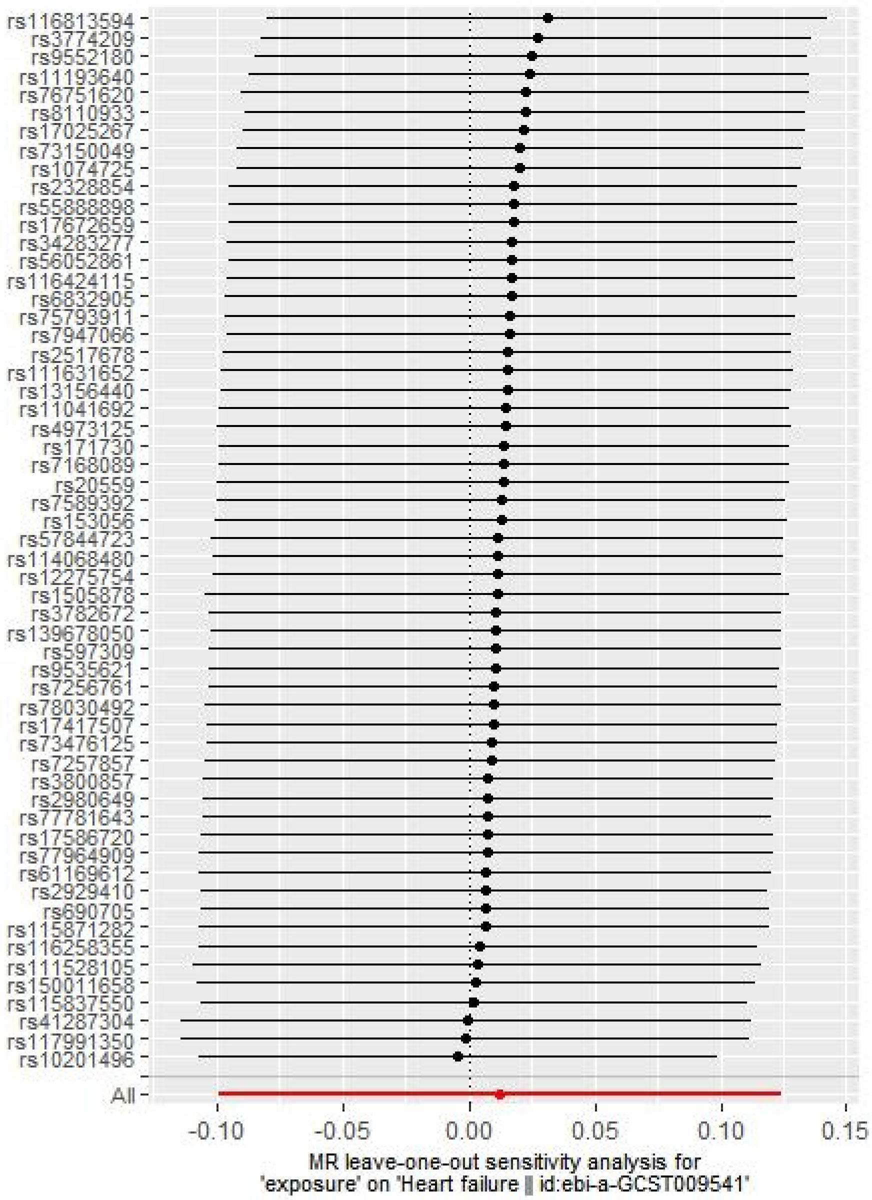

Figure 346: Funnel plots to visualize overall heterogeneity of Mendelian randomization (MR)

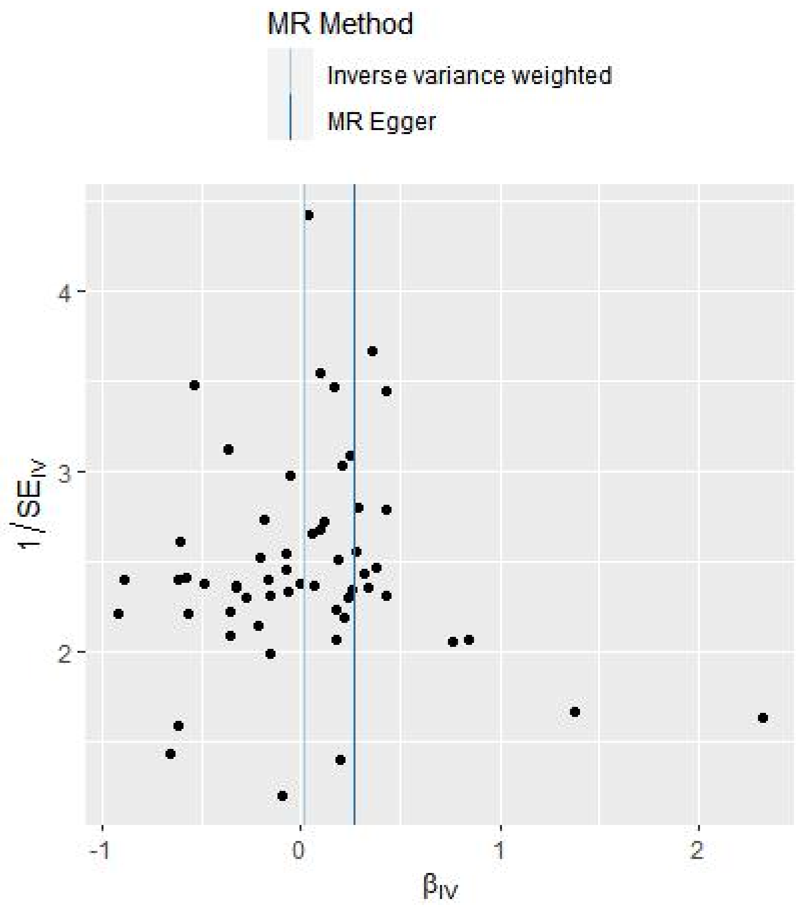

Figure 347: Leave-one-out plot to visualize causal effect of shigella on the risk of heart failure when leaving one SNP out.

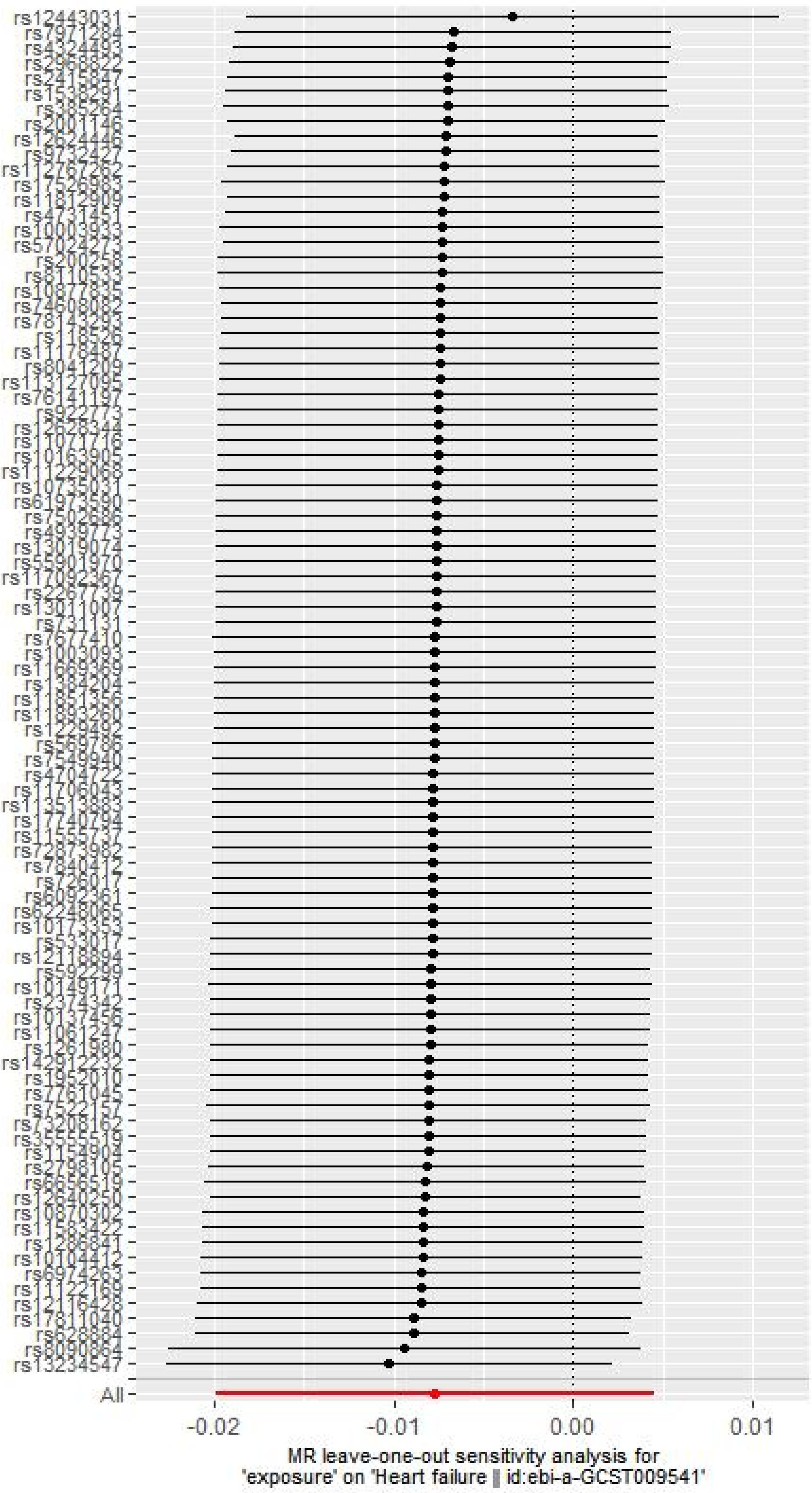

Figure 348: Funnel plots to visualize overall heterogeneity of Mendelian randomization (MR)

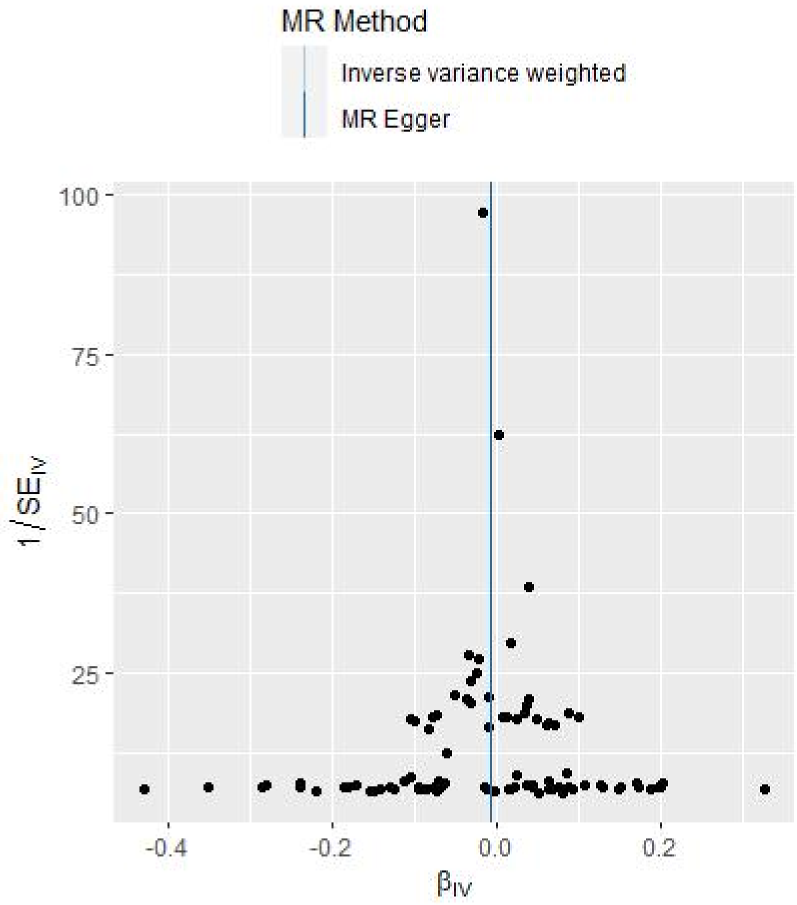

Supplement: Supplementary file 1 [file Data_Sheet_1.zip › Supplementary Material/Supplementary File 5.pdf]
